# Arene Ruthenium(II) Carboxylates for C─H Alkylations and Arylations at Near Room Temperature

**DOI:** 10.1002/anie.202508139

**Published:** 2025-10-22

**Authors:** Xiaoyan Hou, Zhipeng Lin, Takuya Michiyuki, Xuexue Chang, Lutz Ackermann

**Affiliations:** ^1^ Wöhler Research Institute for Sustainable Chemistry (WISCh) Georg‐August‐Universität Göttingen Tammannstraße 2 Göttingen 37077 Germany

**Keywords:** Alkylation, Ambient temperature, Kinetics, *Meta* C─H activation, Ruthenium

## Abstract

The long‐term pursuit for more efficient catalysts has stimulated the development of C─H activations under mild reaction conditions, with the overarching goal to improve their user‐friendly nature, selectivity, and synthetic utility. Herein, we report mild C─H alkylations enabled by inexpensive and most user‐friendly ruthenium carboxylate complexes. While these bench‐stable arene ruthenium carboxylate complexes catalyzed direct alkylations under ambient conditions, detailed kinetic studies demonstrated a high catalytic performance for the [Ru(O_2_CR)_2_(*p*‐cymene)] during the steady‐state catalytic process. Thus, temperature‐dependent kinetic Arrenhius‐plot analyses of the ruthenium‐catalyzed C─H alkylation revealed a comparable activation enthalpy for [Ru(O_2_CR)_2_(*p*‐cymene)] and [Ru(*t‐*BuCN)_5_(H_2_O)](BF_4_)_2_, hence, implying entropic factors to be of relevance. The robust arene ruthenium(II) carboxylate‐catalyzed C─H alkylation showed broad versatility under mild reaction conditions with respect to primary, secondary as well as tertiary alkyl bromides in a position‐divergent manner, reflecting a wide tolerance of valuable electrophilic functional groups for late‐stage functionalizations.

## Graphical abstract

The activity of arene ruthenium catalysts in C─H activation was suggested to depend on high reaction temperatures. In contrast, we show arene ruthenium‐catalyzed C─H activation at near to room temperature.

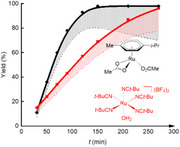

## Introduction

**Figure 1:**
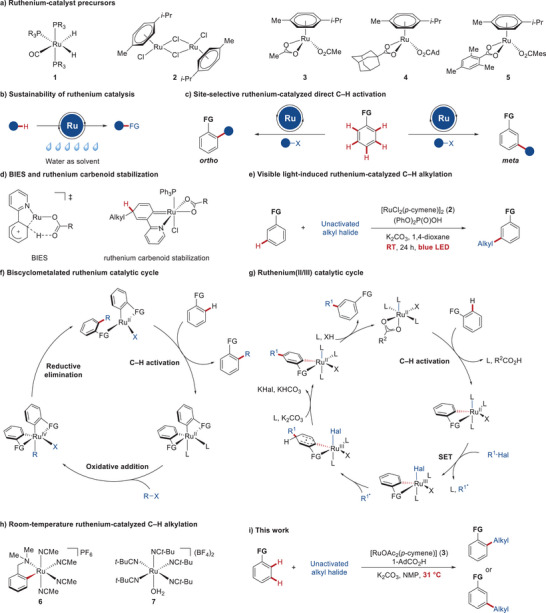
Ruthenium‐catalyzed C─H alkylation.

Over the last few decades, ruthenium(II)^[^ ^1^, ^2^ ^]^ and ruthenium(0)^[^ ^3^, ^4^, ^5^, ^6^ ^]^ catalyzed C─H activations^[^ ^7^, ^8^, ^9^, ^10^, ^11^, ^12^, ^13^, ^14^, ^15^, ^16^ ^]^ have emerged as a powerful tool for expedient access to C─C and C─X bonds (Figure 1a). Especially, the exploitation of air‐ and moisture‐stable, cost‐effective and user‐friendly ruthenium(II) complexes gained tremendous momentum toward milder, and more widely applicable conditions for C─H activations.^[^ ^17^, ^18^, ^19^, ^20^, ^21^, ^22^ ^]^ These ruthenium(II) catalysis not only offered remarkable sustainability through, among others, their full tolerance of water as reaction medium (Figure 1b),^[^ ^23^, ^24^, ^25^, ^26^ ^]^ but also allowed for the precise control^[^ ^27^, ^28^, ^29^, ^30^, ^31^, ^32^, ^33^, ^34^, ^35^, ^36^, ^37^, ^38^ ^]^ of *ortho*‐,^[^ ^39^, ^40^, ^41^ ^]^ *meta*‐,^[^ ^42^, ^43^, ^44^, ^45^, ^46^, ^47^, ^48^, ^49^, ^50^, ^51^, ^52^, ^53^, ^54^ ^]^ and *para*‐selectivity^[^ ^55^, ^56^, ^57^, ^58^, ^59^, ^60^ ^]^ via distinctive manifolds (Figure 1c,d), such as base‐assisted internal C─H activation^[^ ^61^, ^62^ ^]^ and ruthenium carbenoid stabilization.^[^ ^63^, ^64^, ^65^, ^66^ ^]^ Despite of key advances, ruthenium‐catalyzed C─H activations have been typically associated with high reaction temperatures (100–140 °C),^[^ ^67^, ^68^, ^69^, ^70^, ^71^ ^]^ while photochemical conditions enabled these reactions to proceed at room temperature (Figure 1e).^[^ ^72^, ^73^, ^74^, ^75^, ^76^, ^77^, ^78^, ^79^, ^80^ ^]^

The continued demand for efficient and selective catalysis has been reflected by a strong interest in room‐temperature ruthenium catalyzed C─H activations. In this regard, based on early findings on arene‐free ruthenium pre‐catalysts,^[^ ^72^, ^73^, ^74^, ^75^, ^76^, ^77^, ^78^, ^79^, ^80^ ^]^ and mechanistic studies (Figure 1f,g),^[^ ^81^, ^82^, ^83^, ^84^, ^85^ ^]^ pre‐activated cyclometalated ruthenium complexes (Figure 1h) have been identified for C─H alkylation^[^ ^86^, ^87^, ^88^, ^89^, ^90^, ^91^, ^92^ ^]^ at ambient temperature.^[^ ^93^, ^94^ ^]^ However, due to their air‐sensitivity, these complexes **6** require meticulous handling and storage, thus significantly limiting their utility for the practitioners. Recently, a bench‐stable ruthenium complex [Ru(*t‐*BuCN)_5_(H_2_O)](BF_4_)_2_ (**7**),^[^ ^95^, ^96^, ^97^, ^98^, ^99^ ^]^ comprising five pivalonitrile ligands, and two anionic tetrafluoroborates (Figure 1h), was used in C─H activations at 50 °C reaction temperature.^[^ ^100^ ^]^ However, a loading of 10–20 mol% of catalyst **7** was typically required, thus leading to 0.5–1.0 equivalents of pivalonitrile in the system. Based on the ligand field theory and the Tsuchida's spectrochemical series,^[^ ^101^, ^102^, ^103^ ^]^ the ligand field strength of pivalonitrile is only slightly weaker than that of pyridine. Therefore, we hypothesized whether the competing coordination between pivalonitrile and the substrate would inhibit the efficient formation of the active ruthenium intermediate.^[^ ^104^, ^105^ ^]^

We report, herein, a mild C─H activation using unactivated alkyl halides in the presence of the cost‐effective and most user‐friendly arene ruthenium(II) carboxylate as precatalyst (Figure 1i).^[^ ^62^, ^106^, ^107^ ^]^ Kinetic studies validated the power of the user‐friendly ruthenium carboxylate precatalyst under mild conditions. When compared with the reported catalyst **7**, the ruthenium carboxylates featured comparable overall reaction rates for representative substrates. Overall, the robust ruthenium(II) carboxylate catalyst enabled *ortho*‐, *meta*‐C─H alkylation and C─H arylation under mild conditions with the aid of diversified orienting groups, thus demonstrating promising compatibility with late‐stage C─H functionalization of bio‐relevant and thermally sensitive compounds.

## Results and Discussion

We commenced our studies on the ruthenium‐catalyzed C─H alkylations with 5.0 mol% of [RuCl_2_(*p*‐cymene)]_2_ (**2**), 1‐AdCO_2_H, K_2_CO_3_ in NMP at a reaction temperature of 45 °C, however, only 5% of the desired product **10** was obtained (Table 1, entry 1). Encouragingly, when using catalyst **3**, the reaction afforded the *mono*‐ and *di*‐alkylated product in 91% combined yield under mild reaction conditions (entry 2), showcasing an excellent reactivity similar to that of complex **7** (entry 3). This result highlighted the significant role of carboxylate ligand of the precatalyst on the catalytic efficacy. Different carboxylates on the ruthenium complexes did not significantly alter the performance (entries 4, 5). The replacement of the *p*‐cymene ligand with a hexamethylbenzene ligand diminished product formation (entry 6), while benzene‐complex [RuCl_2_(η^6^‐C_6_H_6_)]_2_ (**15**) showed high catalytic performance (entry 7), being indicative of a correlation between the arene dissociation and the catalyst's reactivity.^[^ ^108^ ^]^ Cationic ruthenium benzene complex, [Ru_2_Cl_3_(η^6^‐C_6_H_6_)_2_]PF_6_ (**16**), mirrored the catalytic efficacy at 45 °C, but gave a considerably lower product yield at 35 °C (entry 8). A different cationic ruthenium complex with acetonitrile ligands, namely [Ru(η^6^‐C_6_H_6_)(MeCN)_3_](BF_4_)_2_ (**17**), and a secondary phosphine oxide‐derived precatalyst were less effective (entries 9 and 10). An isocyanide complex proved ineffective (entry 11). Other ruthenium complexes, such as [Ru(*t‐*BuCN)_6_](SbF_6_)_2_ (**20**) and RuCl_3_∙3H_2_O (**21**), failed to furnish the C─H alkylation product at 45 °C (entries 12 and 13), while the addition of 20 mol% Zinc dust to **21** gave 66% of the mono‐alkylated product and 20% of *di*‐alkylated product at 35 °C.^[^ ^109^, ^110^, ^111^, ^112^, ^113^ ^]^ In addition, cationic monocyclometalated ruthenium complexes **11** and **12** enabled the direct alkylation (entries 14 and 15), giving the desired product in moderate yields. Generally, mono‐ and di‐functionalization proved to be viable, and the chemo‐selectivity was largely dependent on the overall conversion, while potassium phenylphosphonate as an additive was not beneficial (Table S1 and Figures S11,S12).^[^ ^93^ ^]^

**Table 1:** Catalytic efficacy of ruthenium complexes.^a)^

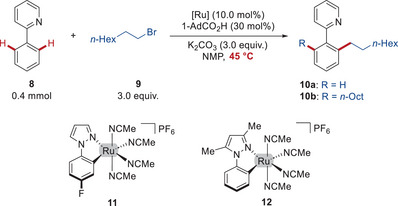		
Entry	Ruthenium complexes	Yield 10a and (10b)
1	[RuCl_2_(*p*‐cymene)]_2_, **2**	5% (trace)
2	[RuOAc_2_(*p*‐cymene)], **3**	61% (30%)^b)^
3	[Ru(*t‐*BuCN)_5_(H_2_O)](BF_4_)_2_, **7**	68% (21%)
4	[Ru(MesCO_2_)_2_(*p*‐cymene)], **5**	72% (11%)
5	[Ru(C_6_Me_5_CO_2_)_2_(*p*‐cymene)], **13**	73% (12%)
6	[Ru(MesCO_2_)_2_(C_6_Me_6_)], **14**	n.d.
7	[RuCl_2_(η^6^‐C_6_H_6_)]_2_, **15**	76% (21%)/80% (13%)^c)^
8	[Ru_2_Cl_3_(η^6^‐C_6_H_6_)_2_]PF_6_, **16**	79% (11%)/8% (3%)^c)^
9	[Ru(η^6^‐C_6_H_6_)(MeCN)_3_](BF_4_)_2_, **17**	n.d.
10	[RuCl_2_(*p*‐cymene)(*i‐*Pr_2_POH)], **18**	18% (trace)
11	[RuCl_2_(*p*‐cymene)(2,6*‐m‐*xyl‐NC)], **19**	Trace
12	[Ru(*t‐*BuCN)_6_](SbF_6_)_2_, **20**	n.d.
13	RuCl_3_ **·**3H_2_O, **21**	n.d./66% (20%)^c)^, ^d)^
14	**11**	54% (3%)
15	**12**	59% (4%)

^a)^ Standard reaction conditions: phenyl pyridine **8** (0.40 mmol), *n*‐octyl bromide **9** (1.20 mmol), ruthenium catalyst (10 mol% of ruthenium atoms), 1‐AdCO_2_H (30 mol%), K_2_CO_3_ (3.0 equiv.), NMP (2.0 mL), N_2_, 45 °C, 18 h. Yields were determined by ^1^H NMR using mesitylene (0.40 mmol) as internal standard. n.d. = not detected.

^b)^ Isolated yield.

^c)^ 35 °C.

^d)^ 20 mol% Zn dust.

**Figure 2:**
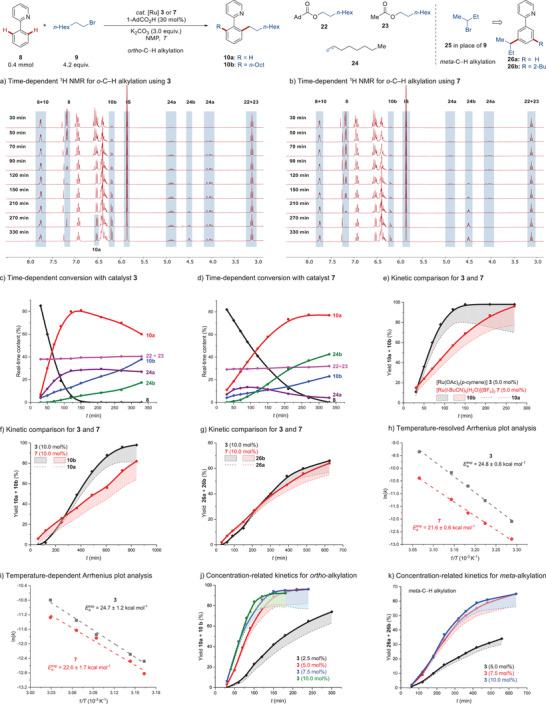
Kinetic studies on ruthenium‐catalyzed C─H alkylation. Reaction conditions: a), c), and e) **3** (5.0 mol%) at 45 °C. b), d), and e) **7** (5.0 mol%) at 45 °C. f) *ortho*‐Alkylation using **3** or **7** (10.0 mol%) at 31 °C. g) *meta*‐Alkylation using **3** or **7** (10.0 mol%) at 45 °C. h) *ortho*‐Alkylation using **3** or **7** (10.0 mol%). i) *meta*‐Alkylation using **3** or **7** (10.0 mol%). j) *ortho*‐Alkylation at 45 °C. k) *meta*‐Alkylation at 45 °C. See supporting information for more experimental details and results. *n*‐Octyl bromide **9** as substrate for *ortho*‐C─H alkylation, 2‐bromobutane **25** (5.5 equiv.) as substrate for *meta*‐C─H alkylation. The solid line corresponds to the combined yield, and the dashed line to the yield of mono‐alkylated products. The variance between the two curves (colored zone) accounts for the yield of di‐alkylated products.

In order to validate the mild nature of the effective ruthenium‐catalyzed C─H alkylation, detailed kinetic analyses were performed (Figure 2). The conversion of phenyl pyridine **8** and *n*‐octyl bromide **9** to the desired product **10** was monitored by ^1^H NMR spectroscopy. The expected disappearance of substrate **8** (7.76, 7.19 ppm) and the generation of mono‐ and di‐alkylated products **10a** (7.76 ppm) and **10b** (7.77, 6.20 ppm) are depicted in Figure 2a,b. The catalysts **3** and **7** both catalyzed the C─H alkylation at a temperature of 45 °C, giving a conversion of 99%. In addition, the formation of side products, such as esters **22** and **23** from the esterification of carboxylates with alkyl halides (3.12 ppm),^[^ ^114^, ^115^ ^]^ terminal octenes **24a** via *beta*‐hydride elimination (4.89, 4.05 ppm) and internal octenes **24b** via double bond chain walking^[^ ^116^ ^]^ (4.50 ppm) were observed for both ruthenium complexes. Here, it is important to note that terminal alkenes are not the intermediate for the alkylation, which was confirmed by control experiments using 1‐decene as a potential starting material under otherwise identical reaction conditions (Figure S7).

Figure 2c,d highlights the time‐resolved reaction profile of the *ortho*‐C─H alkylation with the precatalysts **3** and **7**. In an initial stage, an esterification of the carboxylate occurred. Hence, the in situ formation of the catalysts with 10 mol% of precatalyst **3** along with 20 mol% of additional 1‐AdCO_2_H proved beneficial (Figure S17). A comparison on the nature of the carboxylate additives led to an improved activity for AdCO_2_H versus MesCO_2_H, and a shorter induction period with 1‐AdCO_2_H as an additive (Figures S18 and S19).

Both catalysts **3** and **7** were not chemoselective with respect to the monoalkylation (Figure 2c,d), yielding around 20% of the dialkylated products after complete conversion of **8**. In addition, an alkene isomerization was indeed more pronounced for precatalyst **7**, resulting in a loss of catalytic efficiency on the C─H alkylation.

Next, when comparing the overall conversion to the mono‐alkylated product **10a**, including those converted to di‐alkylated product **10b** (Figure 2e), the reaction using **3** showcased a notably higher reaction rate on the steady state catalytic cycle and thus a shorter overall reaction time, albeit with a slightly longer induction period. Likewise, the inexpensive and user‐friendly ruthenium carboxylate complex **3** proved highly efficient at various catalyst loadings. Here, the reaction efficiency with 5.0 mol% of **3** exceeded that of 10.0 mol% of **7** (Figures S22 and S26). In addition, catalyst **3** demonstrated a higher reaction rate than complex **5** (Figure S16). The reaction kinetics for the ruthenium benzene complexes **15** and **16** were also probed, both of which led to lower reaction rates than the ruthenium carboxylates (Figure S21). Nevertheless, the power of the commercially‐available arene ruthenium family was further validated in the versatility studies below, where the [RuCl_2_(η^6^‐C_6_H_6_)]_2_ exhibited improved catalytic efficacy than well‐defined ruthenium carboxylates for select substrates (**45**, **48**).

The high efficacy of complex **3** on catalyzing C─H alkylations was observed at various mild reaction temperatures, including at an ambient temperature of 31 °C (Figures 2f and S23–27). Moreover, it is noteworthy that the induction period of precatalyst **3** in the direct *ortho*‐C─H alkylation became comparable to that of complex **7**, when slightly increasing the reaction temperature to only 53 °C. Hence, slightly elevated temperatures allow the use of user‐friendly, commercially‐available, and bench‐stable ruthenium carboxylate complexes without compromising their efficiency, rendering them highly attractive for real‐life application.

Next, when comparing the reaction profiles of ruthenium catalyzed *meta*‐C─H alkylation using 2‐bromobutane **25** as the substrate (Figure 2g), the steady state catalytic efficiency of **3** was slightly higher than that of **7**. The reactivity of secondary alkyl bromide was decreased as compared to primary alkyl halides, while alkene formation through *β*‐elimination was observed with secondary alkyl bromides (Figure S36).

Thereafter, to delineate the origin of different reaction rates for the two ruthenium complexes **3** and **7**, Arrhenius plots were employed to interrogate the thermodynamic activation parameters (Figure 2h). In the reaction profile, an approximately linear relationship between the chemical yield and the reaction time could be observed after the induction period of the catalyst (Figure S23). The slope of this linear plot was taken as the observed reaction rate constant *k*_obs_, which was further used for the Arrhenius plot. Consequently, the Arrhenius plots of the *ortho*‐C─H alkylation gave an activation barrier of 24.8 ± 0.8 kcal mol^−1^ for **3**, and 21.6 ± 0.6 kcal mol^−1^ for **7**. Likewise, the experimental activation energy for the *meta*‐C─H alkylation was 24.7 ± 1.2 kcal mol^−1^ for **3**, and 22.6 ± 1.7 kcal mol^−1^ for **7** (Figure 2i). The entropy‐controlled reaction rate highlights the significant role of ordering the reaction components to form the transition‐state species.^[^ ^117^ ^]^

Afterwards, the reaction kinetics of the C─H alkylation were studied with different catalyst loadings. The highly effective pre‐catalyst **3** allowed to decrease the catalyst loading to 7.5 mol% without affecting the reaction efficacy (Figure 2j), suggesting a saturation kinetics, and further to 2.5 mol%, albeit with a somewhat longer overall reaction time. Likewise, saturation kinetics were observed for the *meta*‐C─H alkylation (Figure 2k) within a concentration range of 7.5–10.0 mol%.

**Figure 3:**
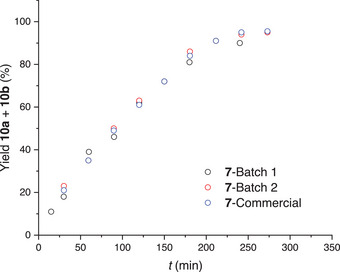
Kinetic analysis using 10.0 mol% of different catalyst **7**. Catalyst of batch 1 and 2 were synthesized by ourselves. See Supporting Information for more details.

When using commercially‐available but highly‐costed catalyst **7** ^[^ ^118^ ^]^ instead of those synthesized by ourselves, consistent kinetic profiles were obtained (Figure 3), validating the robustness of our kinetics comparisons.

**Figure 4:**
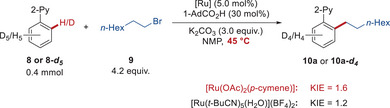
KIE studies.

In further mechanistic studies of the ruthenium‐catalyzed *ortho*‐C─H alkylation, parallel isotope experiments revealed a KIE value of 1.6 for **3**, and a KIE value of 1.2 for **7** (Figure 4). Intramolecular KIE analysis (Figure S64) disclosed the reversible nature of the C─H activation for both **3** and **7**.

**Scheme 1:**
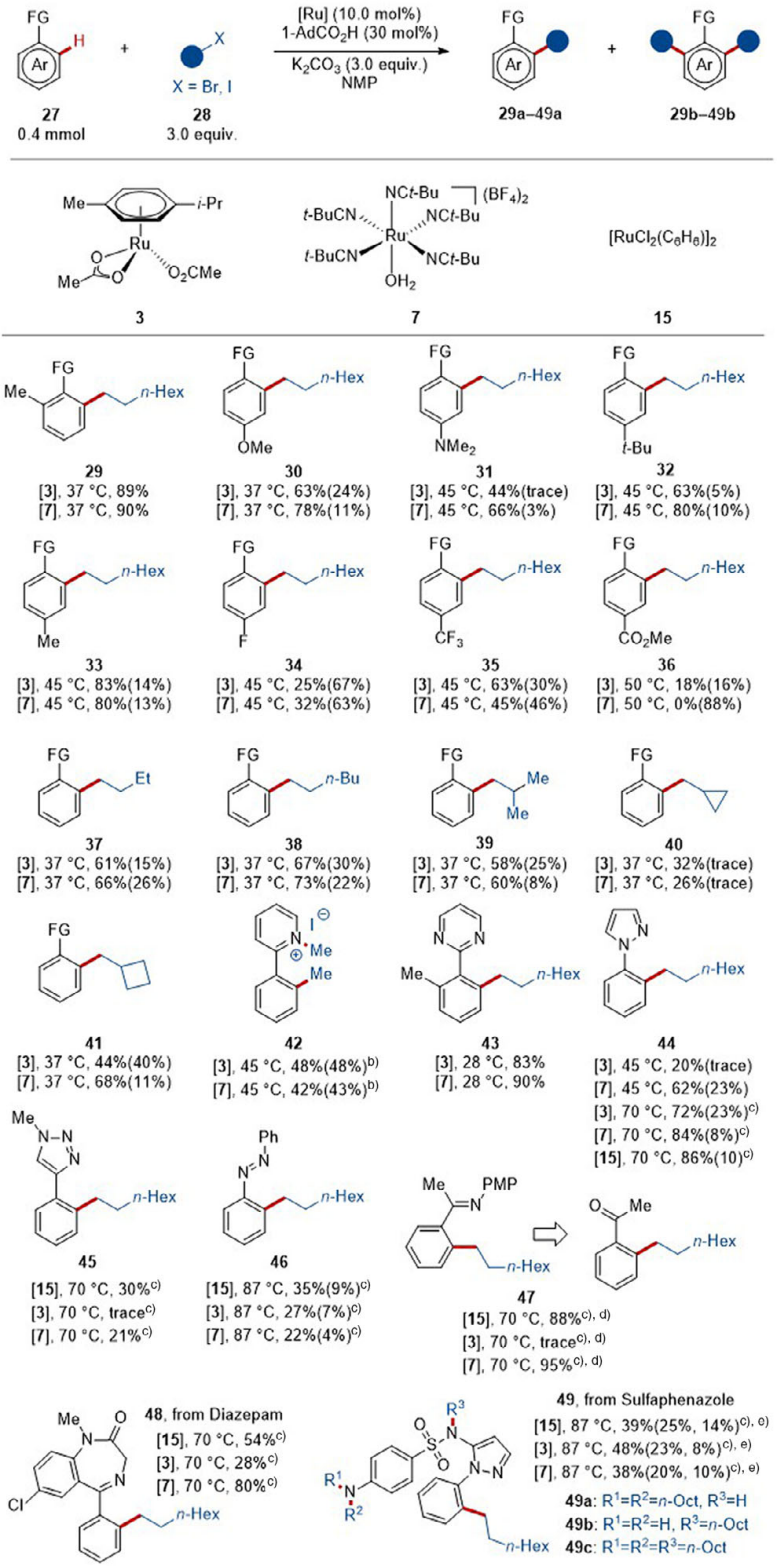
Scope of *ortho*‐C–H alkylation.^a)^ ^a)^ Standard reaction conditions, the reaction temperature used is indicated, the reaction yields are given as **10a** (**10b**) or **49a** (**49b**, **49c**), see Supporting Information for more experimental details. FG = 2‐Py. ^b)^ Iodomethane as substrate. ^c)^ 1,4‐dioxane as solvent. ^d)^ After treatment with HCl (2 M). ^e)^ Overall yield of several *ortho*‐alkylated products with different degrees of *N*‐alkylation.

Subsequently, we assessed the versatility and the robustness of the ruthenium‐catalyzed *o*‐alkylation at mild reaction temperatures (Scheme 1), which proceed through a ruthenium(II/IV) oxidative addition/reductive elimination manifold.^[^ ^64^ ^]^ Azinylarenes bearing electron‐donating or electron‐withdrawing groups (**27a**–**27h**) were well tolerated by both catalysts, giving both mono‐ and di‐alkylated^[^ ^119^ ^]^ products **29**–**36**. It is noteworthy that, for those substrates whose reactivity is lower than that of mono‐alkylated product, catalyst **3** preferred mono‐alkylation over di‐alkylation, thus giving improved mono/di ratio.^[^ ^119^ ^]^

Thereafter, direct C─H alkylations using different primary alkyl bromides were tested with the ruthenium carboxylate and aqua ruthenium catalyst (Scheme 1). Both catalysts **3** and **7** exhibited comparable levels of mono/di‐chemo‐selectivity under mild reaction conditions. Therefore, various primary alkyl bromides bearing linear (**28b** and **28c**) and branched alkyl chains (**28d**–**28f**) proceeded *ortho*‐selectively, affording the desired mono‐products **37a**–**41a** and the di‐products **37b**–**41b**. Cyclopropylmethyl bromide (**28e**) and cyclobutylmethyl bromide (**28f**) were feasible, however, the reactivity of cyclopropylmethyl bromide (**28e**) was notably lower than that of cyclobutylmethyl bromide (**28f**). o*rtho*‐C─H methylation using iodomethane **28g** as the methyl source proved viable under our standard conditions. It is noteworthy that the *N*‐methylated pyridine could be converted into pyrrole in one step according to a reported approach,^[^ ^119^ ^]^ thus increasing the synthetic utility of our *ortho*‐C─H alkylation strategy.

Next, the compatibility of the arene ruthenium‐catalyzed C─H alkylation with different orienting groups was investigated (Scheme 1). Here, diazines (**27j**) were suitable as orienting group, delivering the alkylated product **43** in excellent yield for both catalysts even at room temperature. Although C─H alkylation of phenyl pyrazole **27k** at 45 °C with **3** as catalyst resulted in a low yield, increasing the temperature to 70 °C led to comparable catalytic activities of catalysts **3** and **7**. Additionally, ruthenium benzene complex **15**, showed identical reactivity, thus catalyzing the alkylation in 86% yield (**44a**) at 70 °C. Furthermore, Lewis‐basic triazole (**27l**) and azobenzene (**27m**), were found to be amenable (**45** and **46**), with catalyst **15** showing superior performance as compared to **3** and **7**. Ketimine (**27n**) was tolerated under the catalysis with **7** and **15**, giving the *ortho*‐alkylated acetophenone **47** in excellent yield after acid treatment. Importantly, the robustness of the ruthenium‐catalyzed *o*‐alkylation was translated into late‐stage alkylation of drug compounds (**48** and **49**), such as diazepam (**27o**) and sulfaphenazole (**27p**).

**Scheme 2:**
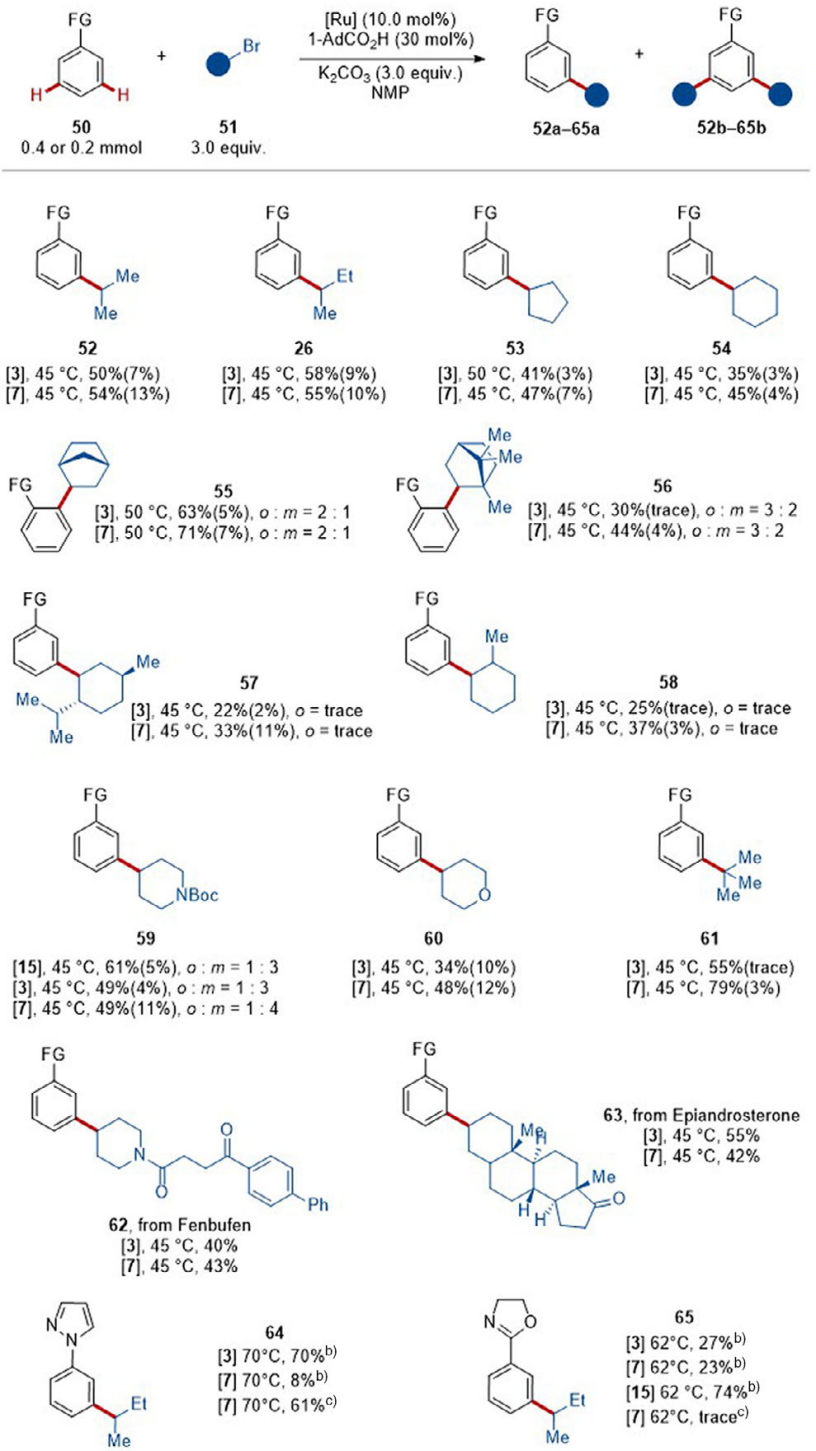
Scope of *meta*‐C─H alkylation.^a)^ ^a)^ Standard reaction condition, the reaction temperature used is indicated, the reaction yields are given as **10a** (**10b**), see Supporting Information for details on dialkylation and for more experimental details. FG = 2‐Py. ^b)^ P(4‐CF_3_‐C_6_H_4_)_3_ (10 mol%) instead of 1‐AdCO_2_H, 1,4‐dioxane as solvent. ^c)^ PhP(O)O_2_K_2_ (30 mol%), NMP as solvent.^[^ ^100^ ^]^

When using secondary alkyl bromides, *meta*‐substitution was favored for all catalysts (Scheme 2). The switch in site‐selectivity is due to the complementary nature of the underlying mechanisms, namely oxidative addition versus SET/XAT for the *ortho*‐ and *meta*‐selective functionalizations, respectively.^[^ ^64^, ^81^ ^]^ In addition, phosphine‐ligated ruthenium complexes can lead to the switch from *ortho*‐ to *meta*‐alkylations by catalyst control.^[^ ^63^ ^]^ Therefore, a variety of the secondary alkyl bromides **51** afforded *meta*‐mono‐alkylated products **52**–**65** with only minor amounts of di‐alkylated products. Importantly, bromonorbornanes favored *ortho*‐alkylation, due to their distinct stereoelectronic properties^[^ ^64^ ^]^ and gave products **55a** and **56a**, respectively.

**Scheme 3:**
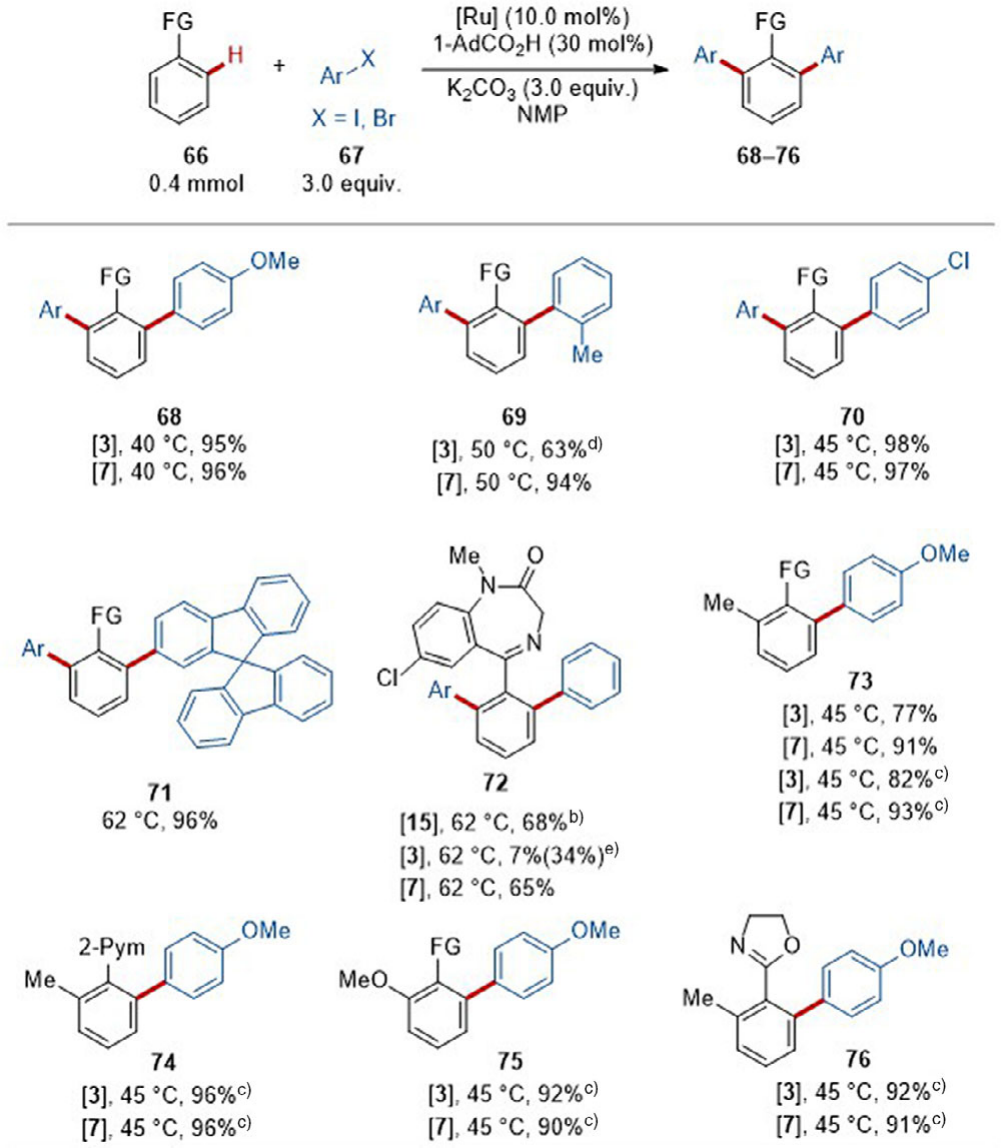
Scope of C–H arylation.^a)^ ^a)^ Standard reaction condition, the reaction temperature used is indicated, see Supporting Information for more experimental details. FG = 2‐Py. ^b)^ Around 2% of mono‐arylated product was detected by GC. ^c)^ 1,4‐dioxane as solvent. ^d)^ 34% of mono‐arylated product. ^e)^ Aryl iodide as substrate.

The catalytic performance of the inexpensive and user‐friendly ruthenium(II) carboxylates **3** proved to be comparable to the one of the complex **7**. Therefore, direct C─H alkylations with the enantiomerically enriched substrate (*S*)‐**51e** underwent a racemization process. Heteroatom‐contained secondary alkyl bromides, such as Boc‐protected 4‐bromopiperidine **51i** and 4‐bromotetrahydro‐2*H*‐pyran **51j** were converted (**59** and **60**). Tertiary alkyl bromide **51k** proved to be viable as well (**61**). Next, the robustness of the mild‐temperature ruthenium‐catalyzed C─H alkylation was validated by the C─H alkylation of complex and bio‐relevant molecules, such as fenbufen and epiandrosterone derivatives (**62** and **63**). Pyrazole and oxazoline were feasible orienting groups as well. Notably, with oxazoline (**65**) catalyst **3** proved to be more effective.

The performance of the ruthenium carboxylates **3** was further examined with C─H arylations at mild temperature (Scheme 3).^[^ ^120^, ^121^, ^122^, ^123^ ^]^ Electron‐rich, electron‐poor or sterically‐hindered *ortho*‐substituted aryl bromides (**67a**–**67c**) selectively gave the desired products **68**–**70** in good to excellent yields. Spirobifluorene **67d** was efficiently converted into diarylated product **71**. The power of the ruthenium‐catalyzed C─H arylations was validated with the late‐stage arylation of diazepam **67e** under mild conditions. Notably, while both catalysts **7** and **15** predominantly gave di‐arylation (**72a**)， catalyst **3** was capable to provide the mono‐arylated product (**72b**) as the main product, albeit in 34% yield.

Subsequently, our mild ruthenium carboxylate system was compared with our previously reported photo‐irradiated ruthenium approach with regard to arylation using the same substrates.^[^ ^124^ ^]^ As a result, all the reactions (**73**–**76**) exhibited high efficacy at 45 °C within the light‐shielding aluminum block. The key to success for the high performance of catalyst **3** is the use of 1‐AdCO_2_H, which is believed to facilitate the dissociation of the *p*‐cymene ligand, thus omitting the light activation condition.

## Conclusion

In summary, we have uncovered the potential of the most user‐friendly (arene)ruthenium(II) carboxylates catalyst for C─H activations under mild conditions. The arene ruthenium catalysis featured *ortho*‐selectivity for primary alkyl and aryl bromides, while *meta*‐selectivity was obtained for secondary and tertiary alkyl bromides. Benchmarking by kinetic analysis, Arrhenius‐plot, and isotope labeling experiments provided valuable insights into the performance of the room temperature C─H activations.

## Supporting Information

Experimental procedures and compound characterization data including ^1^H and ^13^C NMR spectra and kinetic analyses (PDF).

## Conflict of Interests

The authors declare no conflict of interest.

## Supporting information



Supporting Information

## Data Availability

The data that support the findings of this study are available in the supplementary material of this article.
