# Supplementary material for: Arene Ruthenium(II) Carboxylates for C─H Alkylations and Arylations at Near Room Temperature
Source: Angew Chem Int Ed Engl. 2025 Oct 22;64(48):e202508139. doi: 10.1002/anie.202508139 (PMC12643344; doi:10.1002/anie.202508139)
Supplement: Supplementary file 1 — Supporting Information [file ANIE-64-e202508139-s001.pdf]

Supporting Information

Arene Ruthenium(II) Carboxylates for C–H Alkylations at Near Ambient Temperature

Xiaoyan Hou,[a],‡ Zhipeng Lin,[a],‡ Takuya Michiyuki,[a] Xuexue Chang,[a] Lutz Ackermann[a],*

[a] Wöhler Research Institute for Sustainable Chemistry (WISCh)

Georg-August-Universität Göttingen, Tammannstraße 2, 37077, Göttingen, Germany

‡ These authors contributed equally.

Contents

1 General Remarks 3

2 General Procedures: Ruthenium-Catalyzed C–H Alkylation 4

3 Starting Material Syntheses 6

3.1 Synthesis of Phenyl Pyridine Derivatives 6

3.2 Synthesis of Secondary alkyl bromide 8

3.3 Synthesis of Diazepam 9

3.4 Preparation of $[Ru(t\text{-}BuCN)_5(H_2O)](BF_4)_2$ 10

3.5 Preparation of $[RuCl_2(\eta_6\text{-}C_6H_6)]_2$ 14

3.6 Preparation of $[Ru_2Cl_3(\eta_6\text{-}C_6H_6)_2]PF_6$ 15

3.7 Preparation of $[Ru(\eta_6\text{-}C_6H_6)(MeCN)_3](BF_4)_2$ 15

3.8 Preparation of $[Ru(AdCO_2)_2(p\text{-cymene})]$ 15

4 Supplementary Experiments 16

4.1 Hydroarylation with Unactivated Alkene 16

4.2 Photochemical C–H alkylation 16

4.3 C–H Alkylation Using Related Ruthenium Catalyst 16

4.4 Scope with cyclic and bicyclic secondary alkyl halides 17

4.5 Evaluation of $PhP(O)O_2K_2$ 18

4.6 Supplementary Scope 20

5 Kinetic Analysis 21

5.1 General Procedure for Kinetics Studies 21

5.2 Kinetic Profiles 22

5.3 Arrhenius Plot Analysis 27

5.3.1 Ruthenium-catalyzed *ortho*-C–H Alkylation 27

5.3.2 Ruthenium-catalyzed *meta*-C–H Alkylation 34

5.4 Substrate-Dependent Kinetic Analysis 41

5.5 KIE Analysis 54

5.5.1 Parallel Experiments 54

5.5.2 Intramolecular Competition Experiments 55

6 Characterization 57

7 NMR Spectrum 97

8 References 192

1 General Remarks

Catalytic reactions were carried out in a pre-dried glassware, phenyl pyridine derivatives, and alkyl bromide, and aryl bromides were used as obtained by commercial sources, if not noted otherwise. Ruthenium complexes **11**–**14**, **18**–**20**, ketimine **27n**, secondary alkyl bromide **51l** & **51m** were received as a gift. Other chemicals were obtained from commercial sources and were used without further purification. *N*-Methyl pyrrolidone (NMP) was stirred over calcium hydride for 24 h and then distilled under nitrogen at reduced pressure and stored over molecular sieves in the glove box. Potassium carbonate (VWR Chemicals), anhydrous grade, was fine powder and contained about 2% moisture as received. Yields refer to isolated compounds, estimated to be >95% pure as determined by ^{1}H-NMR, or refer to crude ^{1}H-NMR. Chromatography: Merck silica gel 60 (40–63 µm). NMR: Spectra were recorded on a Varian Unity 300, Mercury 300, Inova 500 or Bruker Avance III 300, Bruker Avance III HD 400 and Bruker Avance III HD 500 in the solvent indicated; chemical shifts (δ) are given in ppm relative to the residual solvent peak. M. p. Stuart® Melting Point Apparatus SMP3 melting point apparatus, values are uncorrected. All IR spectra were recorded on a Bruker FT-IR Alpha device. MS: EI-MS- and ESI-MS-spectra were recorded with Finnigan MAT 95, 70 eV and Finnigan LCQ; High resolution mass spectrometry (HR-MS) with APEX IV 7T FTICR.

2 General Procedures: Ruthenium-Catalyzed C–H Alkylation

General Procedure **A**: The reaction was carried out in a pre-dried 10 mL glass vial. In the glove box, phenyl pyridine derivatives (1.0 equiv.), alkyl bromides or aryl halides (3.0 equiv.), [RuOAc$_2$(*p*-cymene) (10 mol %)/[Ru(*t*-BuCN)$_5$(H$_2$O)](BF$_4$)$_2$ (10 mol %)/[RuCl$_2$(η$_6$-C$_6$H$_6$)]$_2$ (5 mol %), 1-AdCO$_2$H (30 mol%), K$_2$CO$_3$ (3.0 equiv.), NMP (1.0–2.0 mL) were placed in the vial and sealed with a PTFE screw cap and adhesive tape. The reaction was performed at *T* °C with a stirring rate of 500 rpm. After the reaction, the mixture was diluted with EtOAc (5.0 mL) and filtered through a short pad of Celite powder. The solvent was removed *in vacuo*. Mesitylene (1.0 equiv.) was added as the internal standard to determine the NMR yield. The crude mixture was purified by flash column chromatography on silica gel to yield the products.

NMP was removed through flash column chromatography, if not noted otherwise.

General Procedure **B**: The reaction was carried out in a pre-dried 10 mL glass vial. In the glove box, phenyl pyridine derivatives (1.0 equiv.), alkyl bromides or aryl halides (3.0 equiv.), [RuOAc$_2$(*p*-cymene)] (10 mol %)/[Ru(*t*-BuCN)$_5$(H$_2$O)](BF$_4$)$_2$ (10 mol %)/[RuCl$_2$(η$_6$-C$_6$H$_6$)]$_2$ (5 mol %), 1-AdCO$_2$H (30 mol%), K$_2$CO$_3$ (3.0 equiv.), 1,4-dioxane (1.0-2.0 mL) were placed in the vial and sealed with a PTFE screw cap and adhesive tape. The reaction was performed at *T* °C with a stirring rate of 500 rpm. After the reaction, the mixture was diluted with EtOAc (5.0 mL) and filtered through a short pad of Celite powder. The solvent was removed *in vacuo*. Mesitylene (1.0 equiv.) was added as the internal standard to determine the NMR yield. The crude mixture was purified by flash column chromatography on silica gel to yield the products.

General Procedure **C**: The reaction was carried out in a pre-dried 10 mL glass vial. In the glove box, phenyl pyridine derivatives (1.0 equiv.), alkyl bromides (3.0 equiv.), [RuOAc$_2$(*p*-cymene)] (10 mol %)/[Ru(*t*-BuCN)$_5$(H$_2$O)](BF$_4$)$_2$ (10 mol %)/[RuCl$_2$(η$_6$-C$_6$H$_6$)]$_2$ (5 mol %), P(4-CF$_3$-C$_6$H$_4$)$_3$ (10 mol%), K$_2$CO$_3$ (3.0 equiv.), 1,4-dioxane (1.0-2.0 mL) were placed in the vial and sealed with a PTFE screw cap and adhesive tape. The reaction was performed at *T* °C with a stirring rate of 500 rpm. After the reaction, the mixture was diluted with EtOAc (5.0 mL) and filtered through a short pad of Celite powder. The solvent was removed *in vacuo*. Mesitylene (1.0 equiv.) was added as the internal standard to determine the NMR yield. The crude mixture was purified by flash column chromatography on silica gel to yield the products.

General Procedure **D**: The reaction was carried out in a pre-dried 10 mL glass vial. In the glove box, phenyl pyridine derivatives (0.4 mmol, 1.0 equiv.), alkyl bromides (3.0 equiv.), [Ru(*t*-BuCN)$_5$(H$_2$O)](BF$_4$)$_2$ (10 mol %), PhP(O)O$_2$K$_2$ (30 mol%), K$_2$CO$_3$ (3.0 equiv.), NMP (2.0 mL) were placed in the vial and sealed with a PTFE

screw cap and adhesive tape. The reaction was performed at *T* °C with a stirring rate of 500 rpm. After the reaction, the mixture was diluted with EtOAc (5.0 mL) and filtered through a short pad of Celite powder. The solvent was removed *in vacuo*. Mesitylene (1.0 equiv.) was added as the internal standard to determine the NMR yield. The crude mixture was purified by flash column chromatography on silica gel to yield the products.

Note: To ensure reproducibility, catalytic reactions were set up in the glovebox and sealed with PVC tape, then transferred out of the glovebox and heated. Only those reactions using a temperature below 40 °C or using a catalyst loading less than 5 mol% were heated in the glove box.

To the best of our knowledge, all reported ruthenium-catalyzed C–H arylations with aryl halides are sensitive to oxygen.

3 Starting Material Syntheses

3.1 Synthesis of Phenyl Pyridine Derivatives

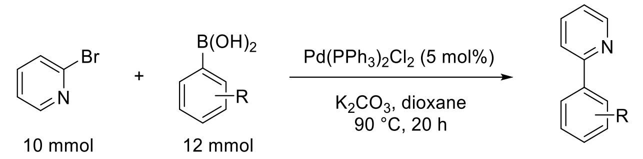

Phenyl pyridine derivatives **27a**–**27h** were synthesized according to a modified procedure from the literature.[1] 2-bromopyridine (10.0 mmol), the corresponding phenylboronic acid (12.0 mmol, 1.2 equiv.), bis(triphenylphosphine)palladium(II) dichloride (5.0 mol%), and K_2CO_3 (3.0 equiv.) were added in a flame-dried Schlenk flask. After degassing with three-times standard Schlenk technique, degassed dioxane (20 mL) was added, and the solution is stirred at 90 °C for 20 h. After the reaction, the resulting reaction mixture was diluted with ethyl acetate (30 mL) and filtered through a pad of Celite. Then the filtrate was removed the solvent in *vacuo*. Purification of the residue by flash column chromatography on silica gel affords the desired 2-aryl-substituted pyridines. If necessary, vacuum distillation was carried out to further purified the product.

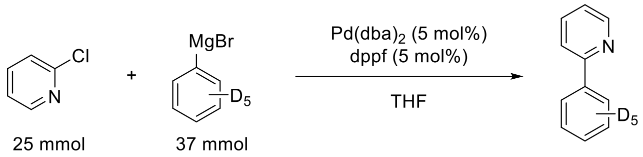

2-(Pentadeuteriophenyl)pyridine was synthesized according to a modified procedure from the literature.[2] Hence, the corresponding Grignard reagent was prepared from 1-bromopentadeuteriobenzene (6.0 g, 37.0 mmol) and magnesium turnings (960 mg, 40.0 mmol) in anhydrous THF (30 mL) in virtually quantitative yield (20–65 °C, 2 h). A second flask was charged with $Pd(dba)_2$ (725 mg, 1.25 mmol, 5 mol%), dppf (675 mg, 1.25 mmol, 5 mol%), anhydrous THF (75 mL), and the resulting mixture was stirred at ambient temperature for 20 min. 2-Chloropyridine (2.838 g, 2.35 mL, 25.0 mmol) was added in one portion, the reaction mixture was stirred at ambient temperature for 0.5 h and cooled to -40 °C. Under stirring, the Grignard reagent was added dropwise via syringe at this temperature over a period of 1 h, the reaction mixture was stirred for an additional 11 h at ambient temperature, poured into ice-cold mixture of sat. aq. NH_4Cl solution (100 mL) with sat. aq. NH_4OH solution (10 mL) and extracted with diethyl ether (3 × 60 mL). The combined extracts were washed with brine (50 mL), dried and concentrated under reduced pressure. Column chromatography of the residue on silica gel afforded **8**-[D_5] as a slightly yellow oil. If necessary, vacuum distillation was carried out to further purified the product.

^1H NMR (300 MHz, $CDCl_3$) δ 8.70 (dt, *J* = 4.8, 1.6 Hz, 1H), 7.82 – 7.70 (m, 2H), 7.23 (ddd, *J* = 6.2, 4.9, 2.4 Hz, 1H). ^{13}C NMR (75 MHz, $CDCl_3$) δ 157.5, 149.7, 136.8, 122.1, 120.6.

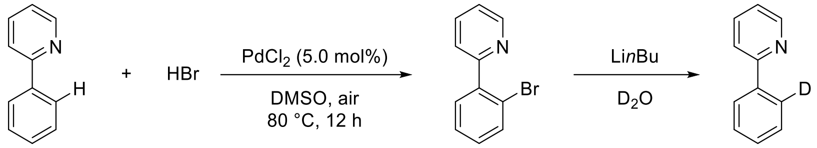

2-(2-Bromophenyl)pyridine was synthesized according to a modified procedure from the literature.[3] $PdCl_2$ (10 mol%) was added to a 25 mL oven-dried Schlenk tube with a stirring bar. 2-Phenylpyridine (6.7 mmol, 1.0 mL) was added followed by the addition of 20 mL of DMSO and 48% (*w*/*w*) HBr aqueous solution (1.1 mL). The mixture was heated to 80 °C under air for 36 h. Upon cooling, 20 mL of brine was added followed by extraction with ethyl acetate (20 mL). The aqueous layer was further extracted with ethyl acetate twice (2 × 20 mL) and the combined organic layer was dried over anhydrous sodium sulfate and filtered. The filtrate was concentrated under reduced pressure giving the resulting residue, which was purified by column chromatography to afford brominated product.

2-deuterio-2-phenylpyridine was synthesized according to a modified procedure from the literature.[4] To a solution of 2-(2-bromophenyl)pyridine (5.0 mmol) in dry THF (25 mL), *n*-butyl lithium (6.3 mL of 1.6 M in hexane, 10.0 mmol, 2.0 equiv.) was added dropwise at -40 °C under nitrogen. After stirring for 30 min, the reaction mixture was quenched with 5.0 mL of D_2O at -40 °C and stirred for 30 min. The reaction mixture was diluted with 40 mL of ethyl acetate and washed with 40 mL of brine. The organic layer was dried over Na_2SO_4 and concentrated under vacuum. The residue was purified by column chromatography on silica gel to give the product.

^1H NMR (300 MHz, $CDCl_3$) δ 8.73 – 8.65 (m, 1H), 8.06 – 7.93 (m, 1H), 7.82 – 7.68 (m, 2H), 7.55 – 7.37 (m, 3H), 7.28 – 7.19 (m, 1H).

3.2 Synthesis of Secondary alkyl bromide

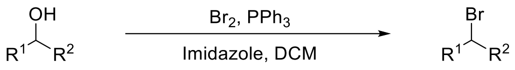

51g and **51h** were synthesized according to a modified procedure from the literature.[5]

Bromine (60 mmol, 3 mL) was added dropwise to a suspension of PPh_3 (60 mmol, 15.7 g) in DCM (100 mL) at 0 °C. The mixture was stirred for 10 min and a white solid precipitated from the mixture. And then imidazole (60 mmol, 4.0 g) was added to the mixture portion wise. Then, alcohol (50 mmol) was added slowly by syringe. The resulting mixture was allowed to warm to room temperature and stirred overnight. The reaction mixture was quenched with saturated aqueous $NaHCO_3$ and extracted with DCM. The organic layer was dried over anhydrous Na_2SO_4, filtered and concentrated. The residue was purified by flash column chromatography on silica gel and further by reduced pressure (35 °C for **51f**) to afford corresponding product.

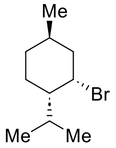

51f ((*1S*,*2S*,*4R*)-2-bromo-1-isopropyl-4-methylcyclohexane)

^{1}H NMR (300 MHz, $CDCl_3$) δ 4.72 – 4.62 (m, 1H), 2.25 – 2.10 (m, 1H), 2.08 – 1.85 (m, 1H), 1.78 – 1.68 (m, 2H), 1.60 – 1.27 (m, 3H), 1.04 – 0.70 (m, 11H).

^{13}C NMR (75 MHz, $CDCl_3$) δ 60.7, 49.3, 43.9, 34.8, 31.4, 26.8, 25.1, 21.8, 20.7, 20.1.

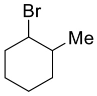

51g (1-bromo-2-methylcyclohexane)

^{1}H NMR (300 MHz, $CDCl_3$) δ 4.49 (q, *J* = 3.2 Hz, 1H), 2.19 – 1.20 (m, 9H), 0.99 (d, *J* = 6.3 Hz, 3H).

3.3 Synthesis of Diazepam

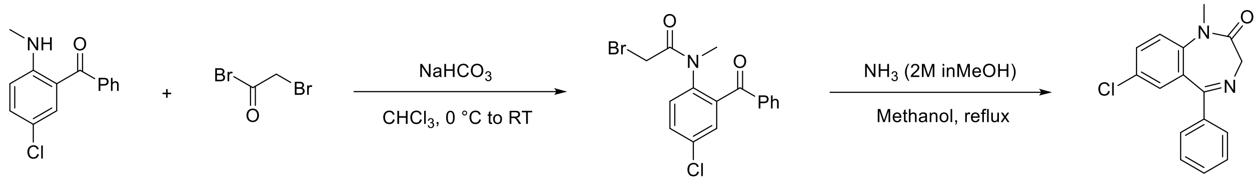

27o was synthesized according to a modified procedure from the literature.[6]

Sodium bicarbonate (22 mmol, 1.85 g) was added to (5-chloro-2-(methylamino)phenyl)(phenyl) methanone (10 mmol, 2.5 g) in chloroform (50 mL). The reaction was cooled to 0 °C, and bromoacetyl bromide (12 mmol, 1.0 mL) was added dropwise. The reaction was warmed to RT and stirred overnight. The reaction was quenched with cold water, and extracted with chloroform, washed with sat. sodium bicarbonate solution, dried over sodium sulfate, filtered, and concentrated in *vacuo*. The concentrate was dissolved in methanol (50 mL) and added 10 mL ammonia (2M in methanol) at 0 °C. The reaction mixture was gradually warmed to RT and refluxed overnight. The reaction was cooled, concentrated in vacuo, and purified by flash column chromatography.

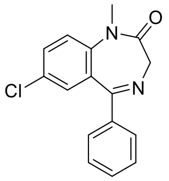

27o (Diazepam)

^{1}H NMR (300 MHz, $CDCl_3$) δ 7.63 – 7.54 (m, 2H), 7.49 (dd, *J* = 8.8, 2.5 Hz, 1H), 7.45 – 7.34 (m, 3H), 7.29 (d, *J* = 8.8 Hz, 1H), 7.26 (d, *J* = 2.5 Hz, 1H), 4.80 (d, *J* = 10.8 Hz, 1H), 3.76 (d, *J* = 10.7 Hz, 1H), 3.37 (s, 3H).

^{13}C NMR (75 MHz, $CDCl_3$) δ 169.9, 168.9, 142.6, 138.2, 131.5, 130.7, 130.0, 129.9, 129.5, 129.2, 128.4, 122.6, 57.0, 34.9.

3.4 Preparation of [Ru(*t*-BuCN)$_5$(H_2O)](BF_4)$_2$

[Ru(*t*-BuCN)$_5$(H_2O)](BF_4)$_2$ was synthesized according to a modified procedure from the literature.[7] Reaction was set up in an argon filled glovebox: $RuCl_3$·*n*H_2O (500 mg), zinc dust (500 mg) and pivalonitrile (12 mL). were loaded in an Ace pressure tube which was subsequently wrapped in Teflon tape and parafilm. The sealed tube was taken out of the glovebox and heated for 2 h at 115 °C behind a blast shield. The reaction mixture was cooled to room temperature and the pivalonitrile removed under reduced pressure. The resulting mixture was diluted with HPLC grade water (80 mL) and filtered through a small plug of Celite ensuring all solids were removed. $AgBF_4$ (2.8 g, 14.5 mmol, 2.5 equiv.) was added and the resulting solution and was allowed to stir at room temperature for 1 hour. The product was precipitated with Et_2O from a solution in CH_2Cl_2 5 times giving [Ru(*t*-BuCN)$_5$(H_2O)](BF_4)$_2$ as a fine light-yellow powder.

^{1}H NMR (400 MHz, CD_2Cl_2) δ 1.51 (s, 36H), 1.46 (s, 9H). **^{13}C NMR** (101 MHz, CD_2Cl_2) δ 136.2, 134.2, 31.2, 30.7, 28.7, 28.3. **^{19}F NMR** (377 MHz, CD_2Cl_2) δ -150.6, -150.7.

HR-MS (ESI): m/z calcd for $C_{25}H_{47}N_5ORuBF_4$ $[M]^+$: 622.2850, found: 622.2844.

M.p.: 182–183 °C (decomp.).

IR (ATR): ṽ = 3403, 2975, 2942, 2261, 1482, 1464, 1372, 1244, 1057, 991 cm^{-1}.

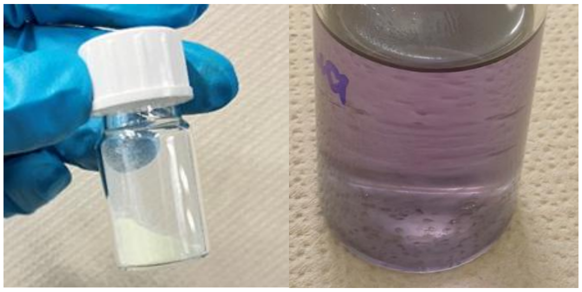

Figure S 1 Appearance of crystallization process of a batch of catalyst 7.

The RuAqua prepared by us have the same colour as those prepared in the *nature chemistry* paper (Figure S1). The purity of the RuAqua prepared by us is consistent with that reported, which is supported by the NMR characterizations (Figure S2 & S3).

The color of catalyst **7** solution in DCM/Et2O is light yellow and transparent, which is consistent with those reported in the Nature Chemistry paper as well. The solution for crystallization could turn light dark or light purple in a few minutes under air (Figure S1). Such a coloration of Catalyst **7** solution is likely attributable to minor impurities or catalyst degradation. Nevertheless, we successfully reproduced consistent reaction yields and kinetic profiles across different batches of Catalyst **7** (Figure S4). This suggests that trace impurities do not significantly impact reaction rates, thereby validating the reliability of our comparative analyses.

a year of storage was compared to a freshly made batch. These NMR spectra are shown below.

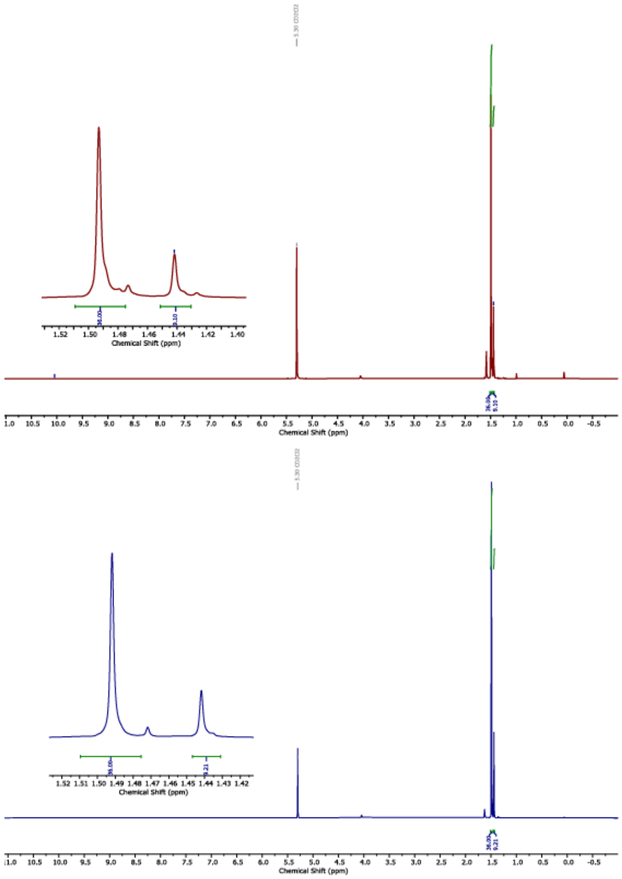

Figure 3 Air-stability of **RuAqua**. Top (red): new batch of **RuAqua**. Bottom (blue): over 1 year old batch of **RuAqua**.

Figure S 2 **Purity of reported RuAqua.**

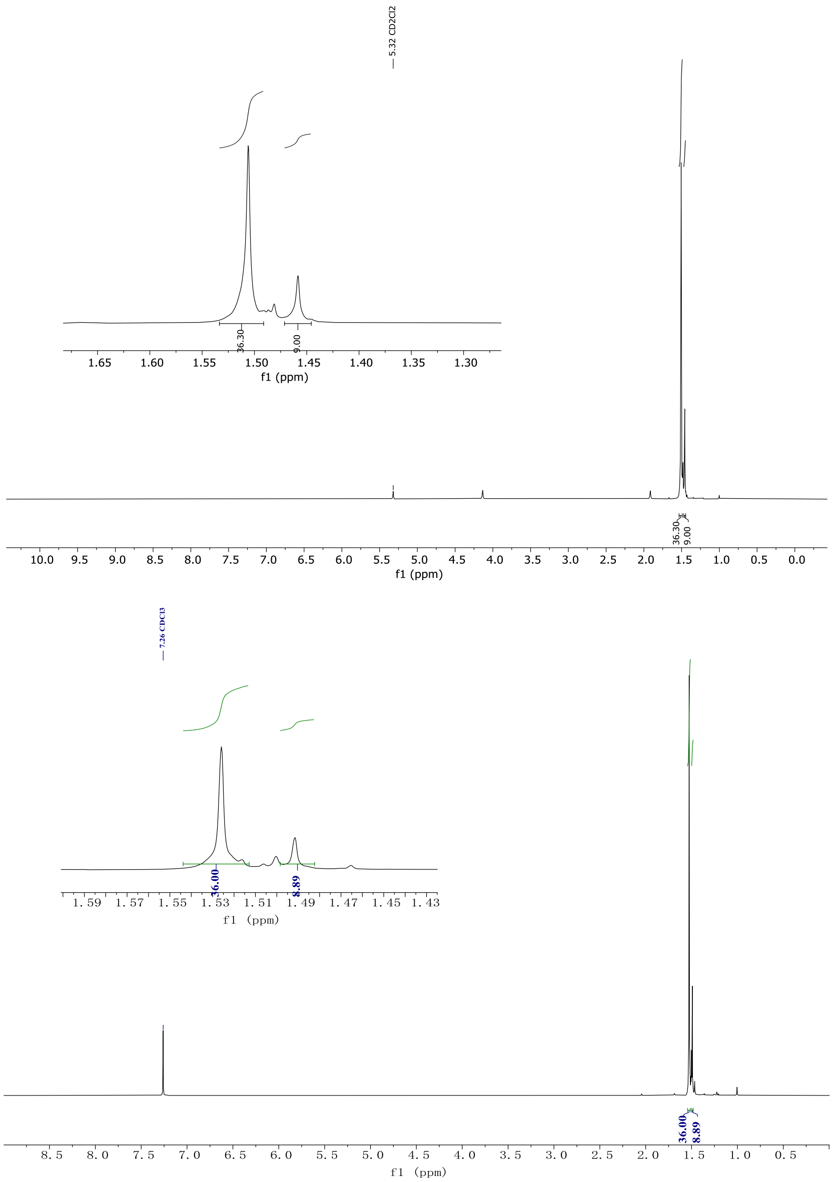

Figure S 3 **Purity of our prepared RuAqua from different batches.**

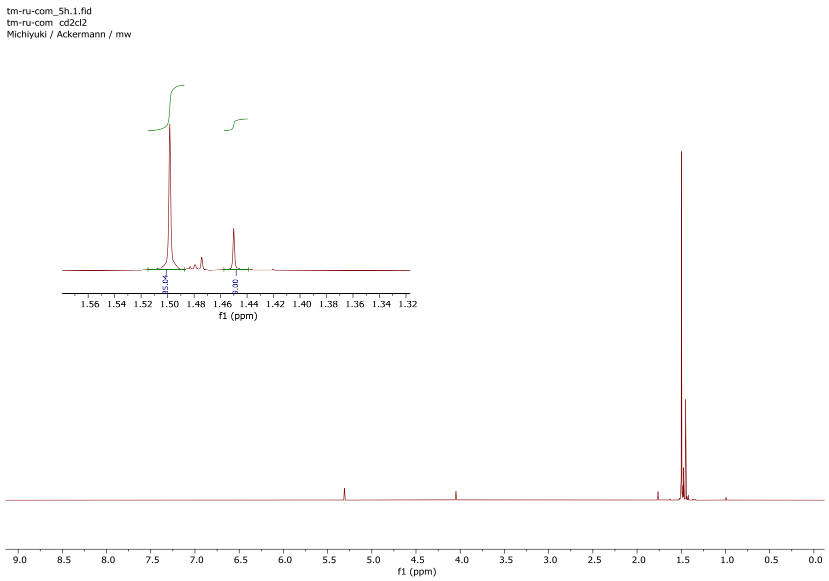

Figure S 4 Purity of commercially-available RuAqua.

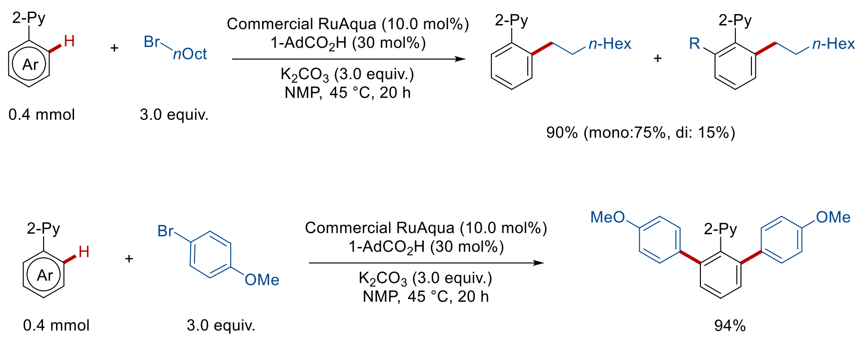

Figure S 5 Alkylation and arylation using commercial 7 as catalyst.

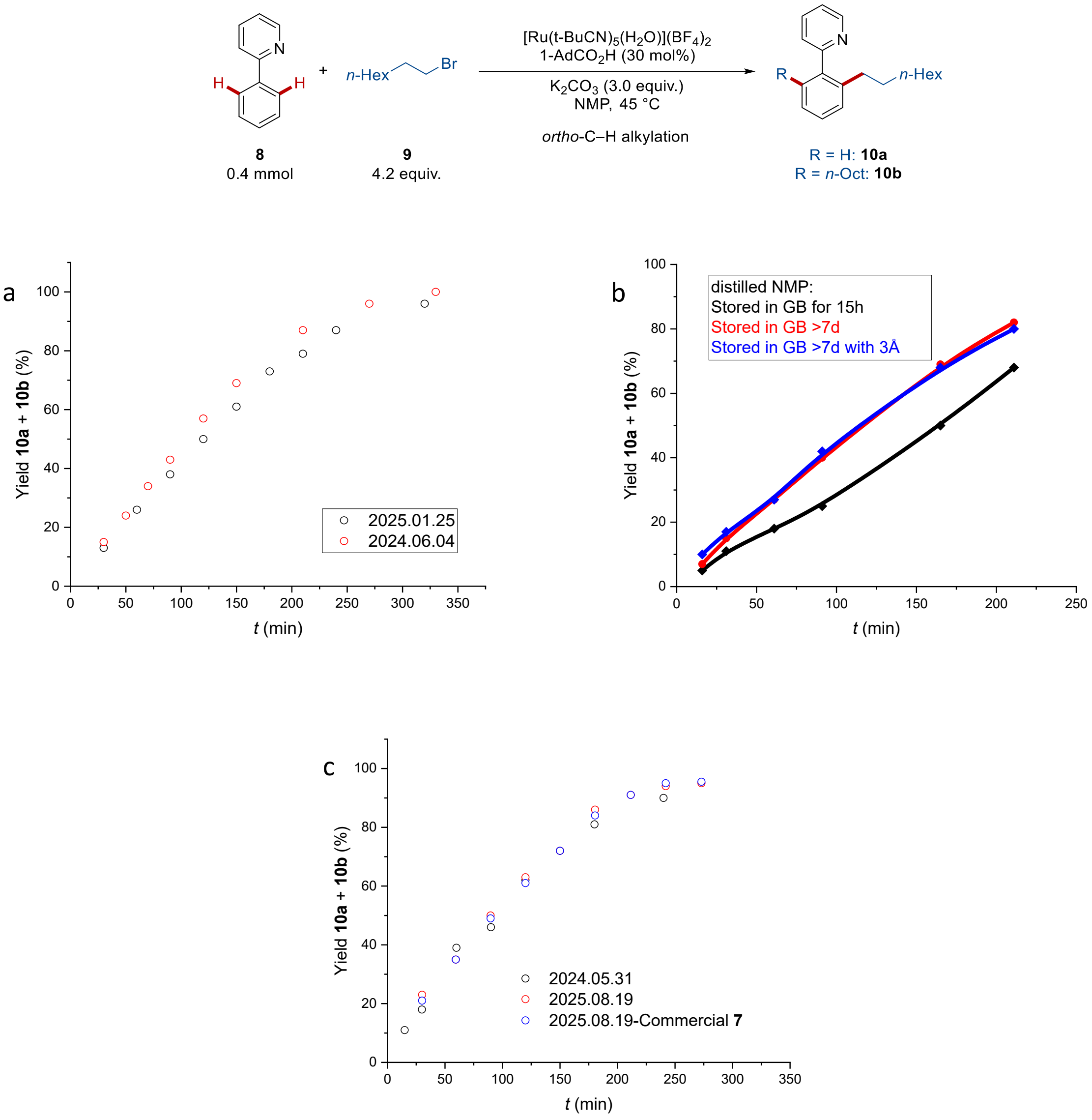

Figure S 6 a) Kinetic analysis using 5.0 mol% of RuAqua from different batches. b) Kinetic analysis using NMR with trace of oxygen. 7.5 mol% of RuAqua were used. The NMP were bubbled with nitrogen for 10 min before transferring into glovebox. c) Kinetic analysis using 10.0 mol% of RuAqua from different batches and commercially-available RuAqua.

3.5 Preparation of $[RuCl_2(\eta_6\text{-}C_6H_6)]_2$

$[RuCl_2(\eta_6\text{-}C_6H_6)]_2$ was synthesized according to a modified procedure from the literature.[8] Hydrated ruthenium trichloride (1.0 g) in ethanol 50 mL) was heated under reflux with cyclohexadiene (either 1,3-

or 1,4-) (5.0 mL) for 4 h. The brown precipitate was filtered off, washed with methanol, and dried in *vacuo*. A nitrogen atmosphere can be used but is not strictly necessary.

^{1}H NMR (300 MHz, $CDCl_3$) δ 5.97 (s, 6H). **^{13}C NMR** (75 MHz, $CDCl_3$) δ 87.6.

3.6 Preparation of [$Ru_2Cl_3(\eta_6\text{-}C_6H_6)_2$]$PF_6$

[$Ru_2Cl_3(\eta_6\text{-}C_6H_6)_2$]$PF_6$ was synthesized according to a modified procedure from the literature.[8] The complex [$RuCl_2(\eta_6\text{-}C_6H_6)$]$_2$, (0.2 g) was heated under reflux with water (10 mL) for 2 h. The orange solution was filtered and treated with a saturated aqueous solution of ammonium hexafluorophosphate (5.0 mL). After 3 days, the orange precipitate was filtered off, washed with water and methanol, and dried in *vacuo*.

3.7 Preparation of [$Ru(\eta_6\text{-}C_6H_6)(MeCN)_3$]$(BF_4)_2$

[$Ru(\eta_6\text{-}C_6H_6)(MeCN)_3$]$(BF_4)_2$ was synthesized according to a modified procedure from the literature.[8] The complex [$RuCl_2(\eta_6\text{-}C_6H_6)$]$_2$ (0.5 g) in acetonitrile (15 mL) was treated with $AgBF_4$ (0.9 g, 2.3 equiv.) and stirred at 35 °C for 2 h. Silver chloride was filtered off to give a clear yellow solution. After 2 days at 0 °C, yellow-orange crystals of the complex had formed.

^{1}H NMR (300 MHz, CD_3CN) δ 6.37 (s, 6H), 2.66 (s, 9H). **^{13}C NMR** (75 MHz, CD_3CN) δ 89.0, 4.5, 1.8. **^{19}F NMR** (282 MHz, CD_3CN) δ -151.7, -151.7.

3.8 Preparation of [$Ru(AdCO_2)_2$(*p*-cymene)]

[$Ru(AdCO_2)_2$(*p*-cymene)] was synthesized according to a modified procedure from the literature.[9] A suspension of [$RuCl_2$(*p*-cymene)]$_2$ (200 mg, 0.326 mmol), 1-$AdCO_2H$ (235 mg, 1.306 mmol) and K_2CO_3 (451 mg, 3.260 mmol) in PhMe (35 mL) was stirred under N_2 for 3 h at ambient temperature. The solvent was removed under *vacuo* and the remaining residue was dissolved in dry CH_2Cl_2 (50 mL) and the resulting suspension was filtered under N_2 through a short pad of Celite. The solvent was removed under *vacuo* to yield an orange solid as the product.

4 Supplementary Experiments

4.1 Hydroarylation with Unactivated Alkene

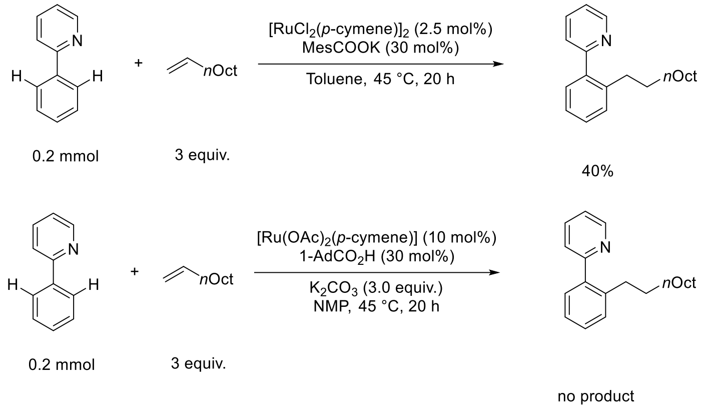

Figure S 7 Ruthenium Catalyzed Hydroarylation with Alkenes.

4.2 Photochemical C–H alkylation

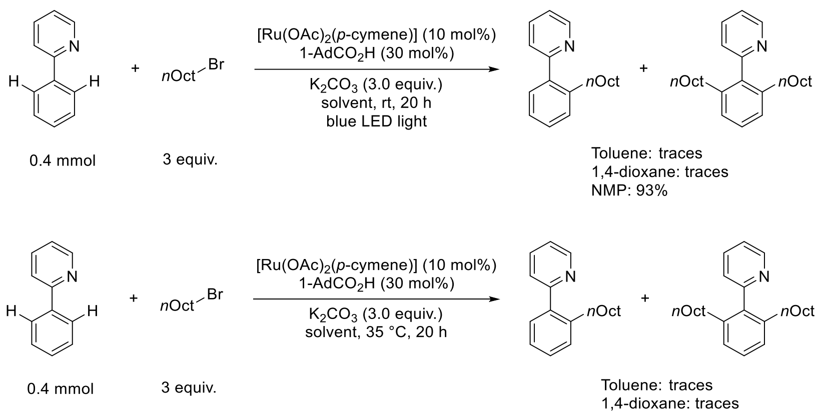

Figure S 8 Ru-catalyzed Direct C–H Alkylation under Photochemical Condition.

4.3 C–H Alkylation Using Related Ruthenium Catalyst

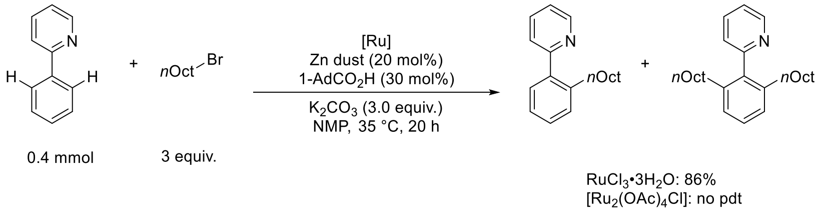

Figure S 9 Ru-catalyzed C–H alkylation Using Zn as Reductant.

4.4 Scope with cyclic and bicyclic secondary alkyl halides

Further, we tested additional substrates with similar stereoelectronic properties (Figure S10). Bromocyclopentane (**53**), bromocyclohexane (**54**) and its derivatives **57** and **58**, bromopiperidine (**59**), and bromo-tetrahydro-*2H*-pyran (**60**) gave the *meta*-products as the predominant products. *ortho*-Isomers of **53**, **54**, **57**, **58**, and **60** were only observed in trace amounts according to gas-chromatography analysis of the crude mixture. Solely bicyclic secondary alkyl bromides, such as bromonorbornane (**55**, **56**), gave significant amounts of the *ortho*-products likely due to their unique bicyclic nature.

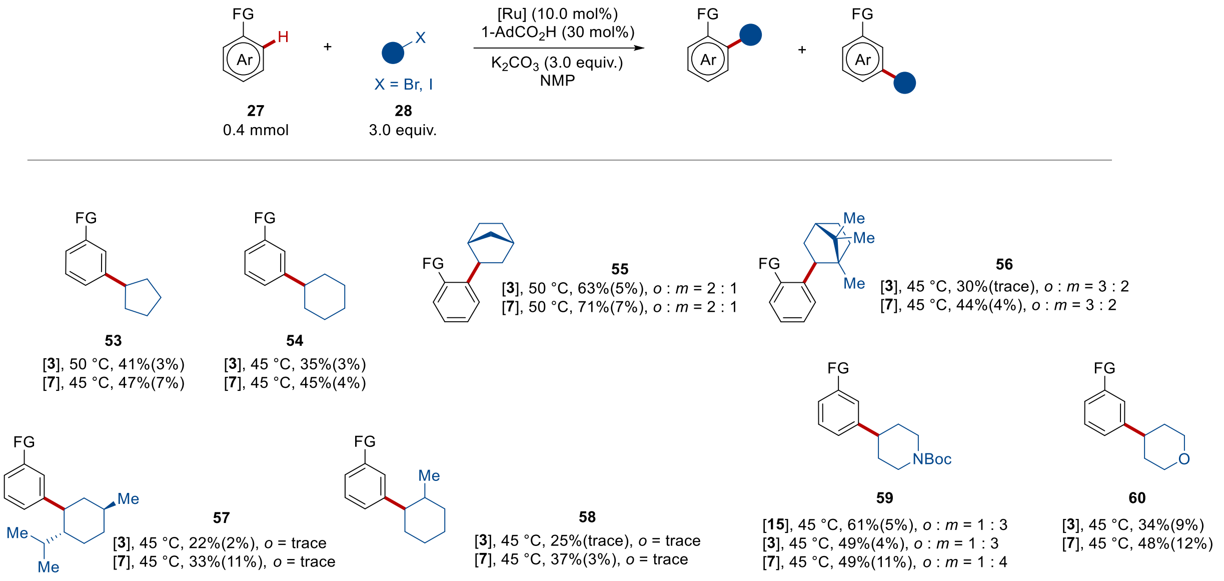

Figure S 10 Substrate Scope for cyclic and bicyclic secondary halides 28. The yield of di-alkylated product was indicated in parentheses.

4.5 Evaluation of $PhP(O)O_2K_2$

While both catalyst **3** and **7** gave mono- and di-alkylated products in excellent combined yields in the presence of either $PhP(O)O_2K_2$ or 1-$AdCO_2H$ (Table S1), there is no strong evidence for the relation between $PhP(O)O_2K_2$ and good mono/di ratio.[13] The mono/di selectivity is more dependent on reaction efficiency and reaction time. In addition, kinetic comparisons revealed that $PhP(O)O_2K_2$ has a strong impact on the activation of catalyst **3**, leading to a much longer induction period than that of 1-$AdCO_2H$ (Figure S11). In the case of catalyst **7**, both additives gave similar initial reaction rates, while the use of $PhP(O)O_2K_2$ resulted in a slightly inferior catalytic performance in the later stage of the reaction, but less dialkylated products (Figure S12). Nevertheless, the dialkylation products increase as the reaction time, of which the accumulation leads to a poor mono/di selectivity eventually.

$PhP(O)O_2K_2$ was not used in our kinetics studies for other reasons given below.

- The solubility of $PhP(O)O_2K_2$ in NMP is poor, 30 mol% of $PhP(O)O_2K_2$ did not fully dissolve in 2 mL of NMP (0.4 mmol reaction scale), making it difficult to prepare the stock solution for reproducible kinetic experiments.
- $PhP(O)O_2K_2$ contains aromatic protons, which could cause problems on the crude ^1H NMR integration.

Table S 1 Comparison of additives.

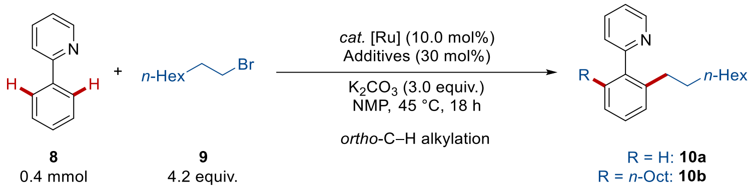

Entry	catalyst	additives	10a	10b	Overall yield
1	[Ru(OAc)$_2$(*p*-cymene)]	1-$AdCO_2H$	60%	31%	91%
2	[Ru(OAc)$_2$(*p*-cymene)]	$PhP(O)O_2K_2$	71%	12%	83%
3	[Ru(*t*-BuCN)$_5$(H_2O)](BF_4)$_2$	1-$AdCO_2H$	68%	21%	89%
4	[Ru(*t*-BuCN)$_5$(H_2O)](BF_4)$_2$	$PhP(O)O_2K_2$	63%	32%	95%

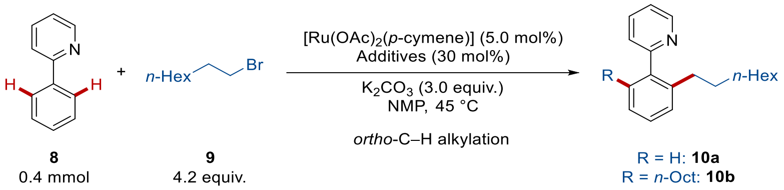

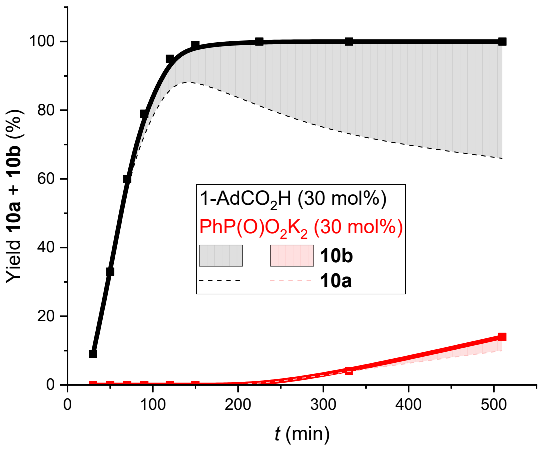

Figure S 11 Kinetic comparison of additives for catalyst 3.

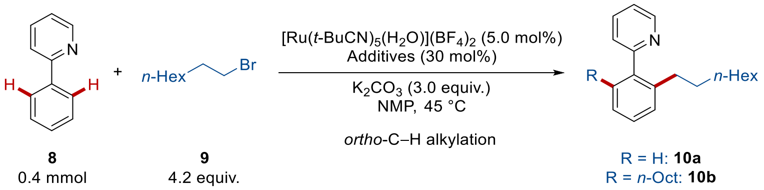

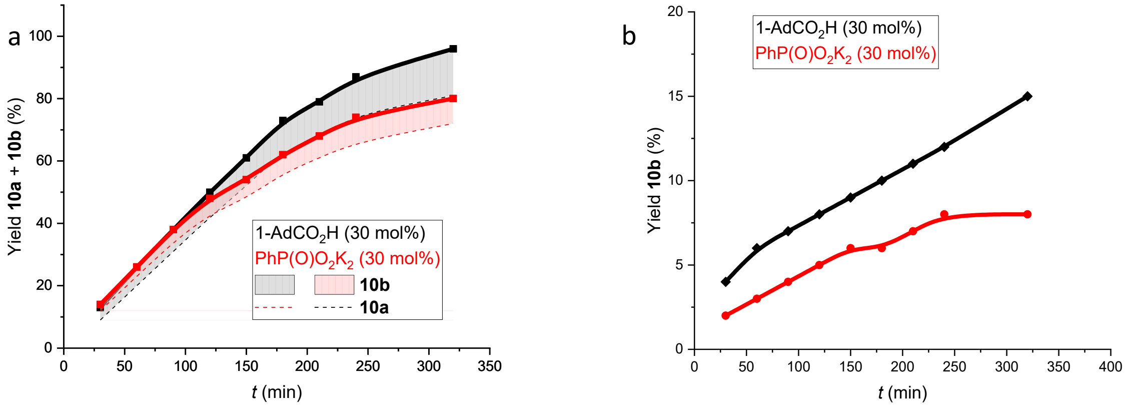

Figure S 12 a) Kinetic comparison of additives for catalyst 7. b) Comparison of di-alkylated products using different additives.

4.6 Supplementary Scope

Benzyl bromide and allyl bromide have been tried under 45 °C, both our catalyst and the [Ru(*t*-BuCN)$_5$(H$_2$O)](BF$_4$)$_2$ afforded the desired product in low yield (Figure S13). Activated alkyl halide like ethyl bromoacetate also didn't work under our condition.

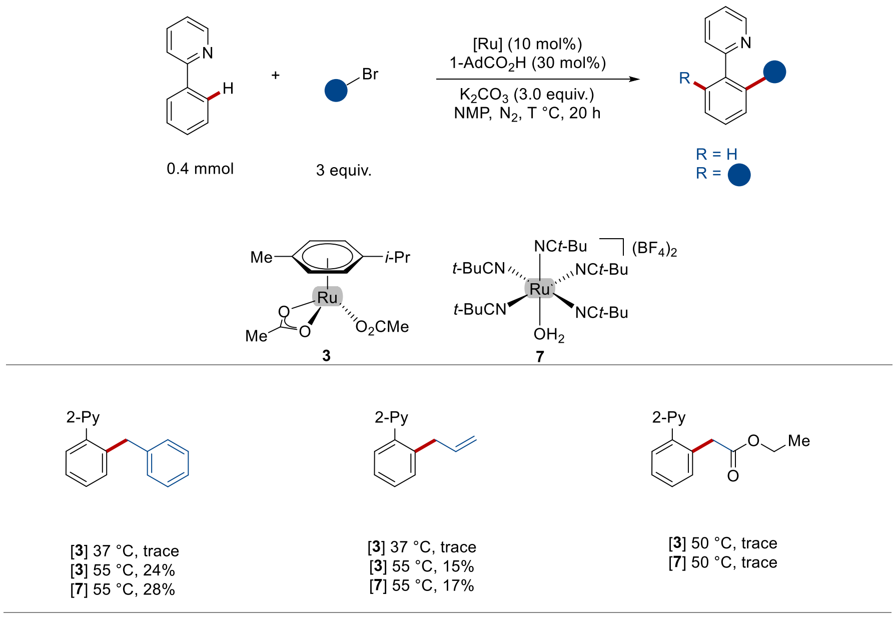

Figure S 13 Supplementary scope.

5 Kinetic Analysis

5.1 General Procedure for Kinetics Studies

Reaction conditions: **phenyl pyridine** (0.80 mmol), **mesitylene** (0.80 mmol), **octyl bromide** or **2-bromobutane** (0.60 mL), **[Ru(OAc)$_2$(*p*-cymene)]** or **[Ru(*t*-BuCN)$_5$(H$_2$O)](BF$_4$)$_2$** (10 mol %), **1-AdCO$_2$H** (30 mol %), NMP (4.0 mL) were mixed in the glovebox to form a homogeneous solution. Then 0.5 mL of this reaction mixture and K_2CO_3 (43.0 mg) was added to a vial with a stirring bar and sealed with a cap, the procedure was repeated 9 times to produce 9 reactions in the same conditions. The reaction vials were placed into a pre-heated alumina block (45 °C) and stirred with a rate of 500 rpm. The reaction temperature was monitored with an exogenous thermal meter. After specific time intervals, one reaction vial was transferred outside the glovebox. The reaction mixture was added with 0.5 mL $CDCl_3$ and filtered with a short pad of Celite, and then analyzed by ^{1}H NMR.

Reliability of the procedure were validated below (Figure S14 & S15).

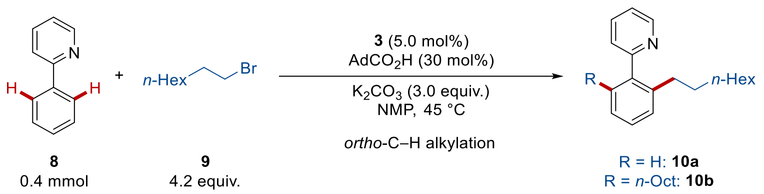

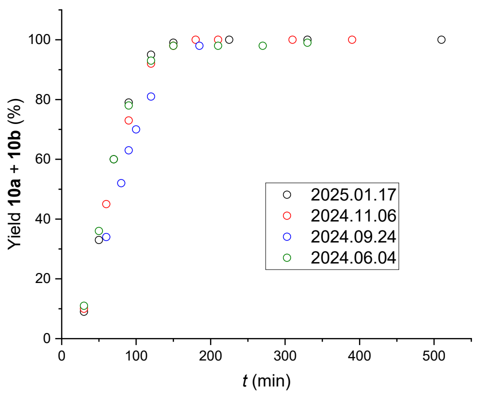

Figure S 14 Reaction profiles of different *ortho*-alkylation under the same conditions.

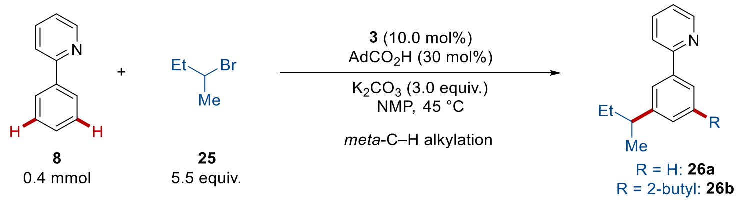

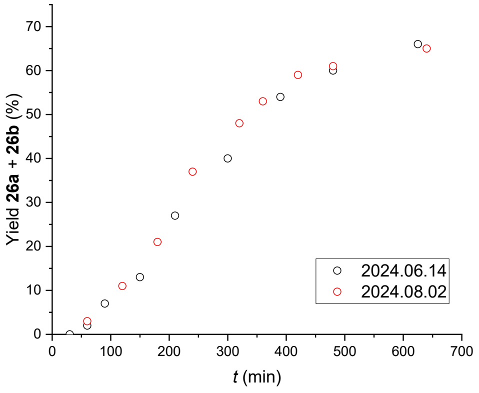

Figure S 15 Reaction profiles of different *meta*-alkylation under the same conditions.

5.2 Kinetic Profiles

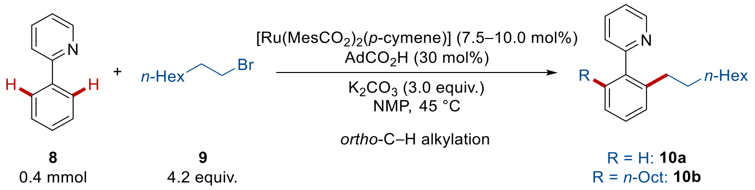

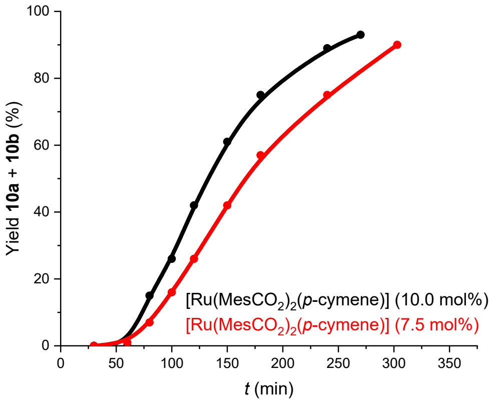

Figure S 16 Direct C–H Alkylation Using 5 as Catalyst.

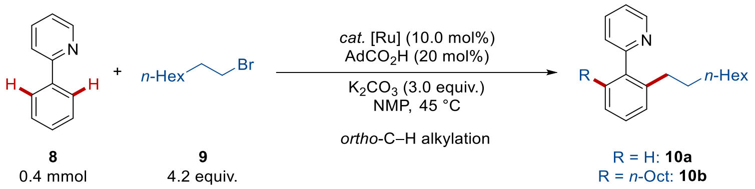

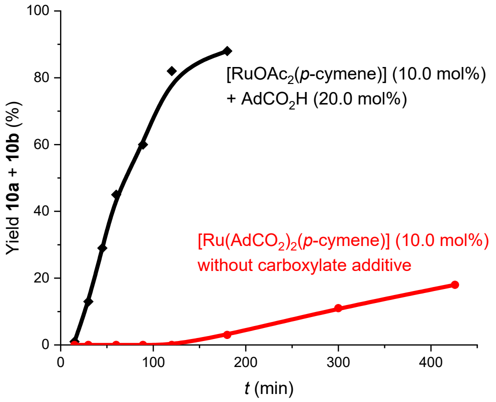

Figure S 17 **The Role of Carboxylate Additive in Direct C–H Alkylation.**

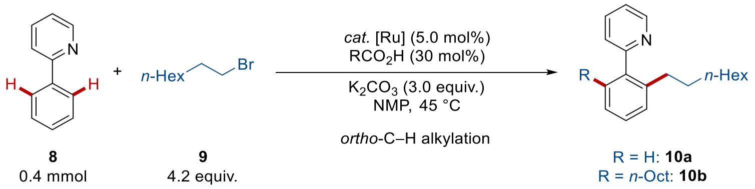

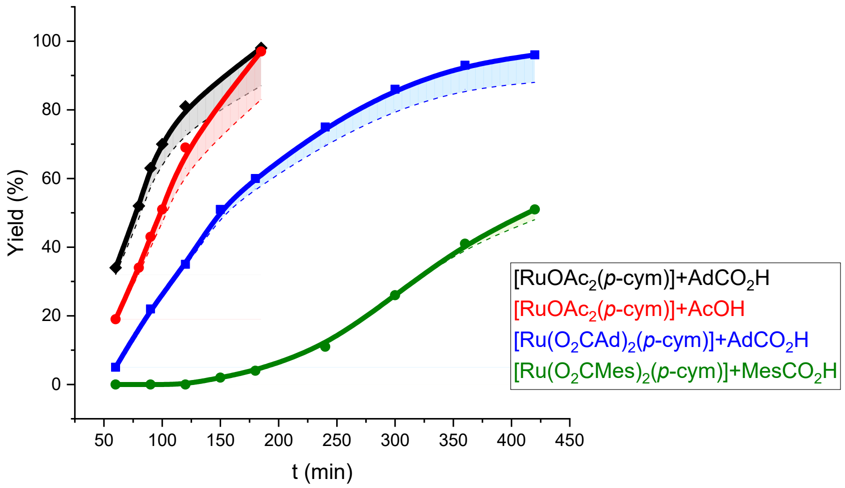

Figure S 18 **Evaluation of Different Carboxylates.** Reactivity order: AcOH > $AdCO_2H$ > $MesCO_2H$. The role of $AdCO_2H$ is to facilitate the dissociation of *p*-cymene from ruthenium catalyst.

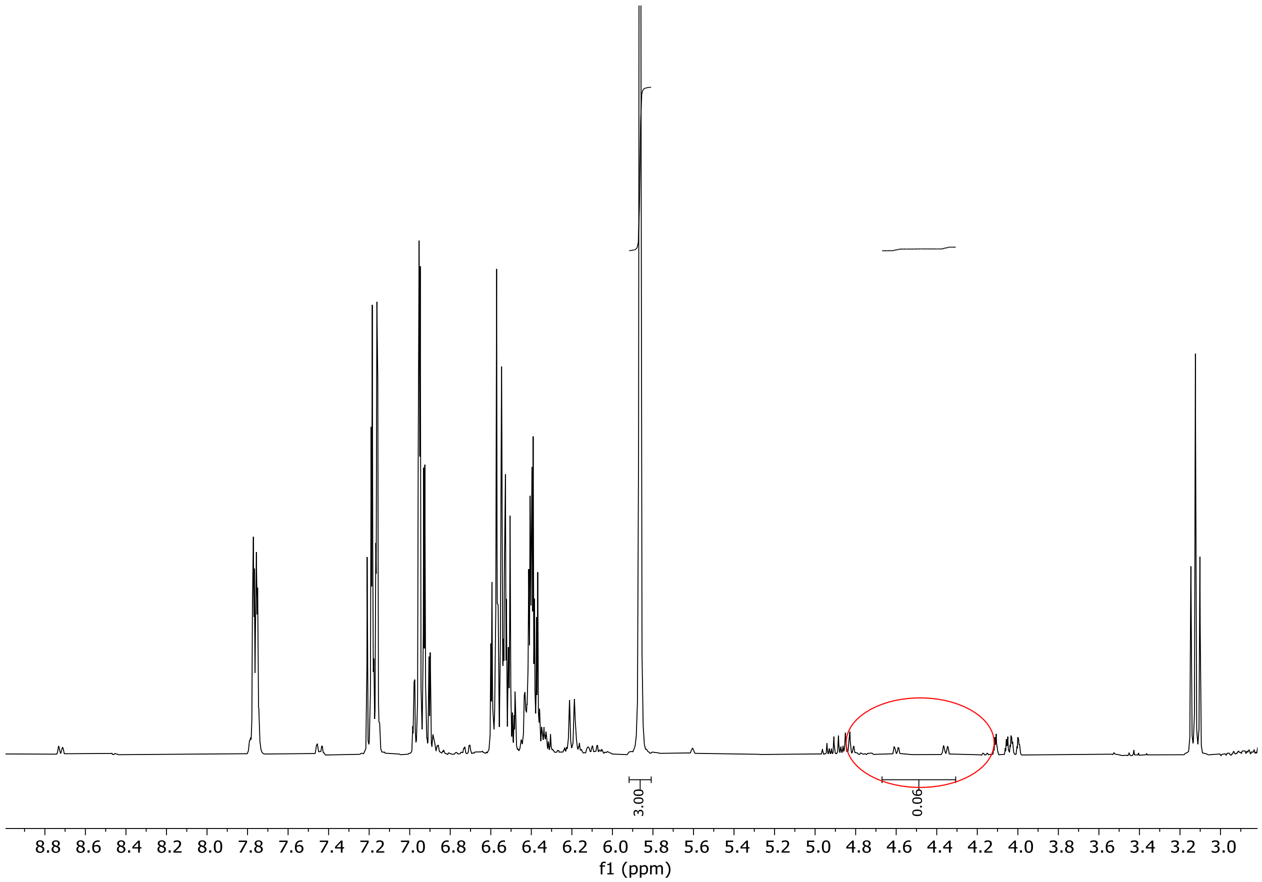

Figure S 19 **^{1}H NMR for Reaction Using AcOH as Additive.** Reaction time 1 hour. The characteristic peak of ruthenium-bound *p*-cymene was still observed, which was not found in the reaction using $AdCO_2H$ as an additive.

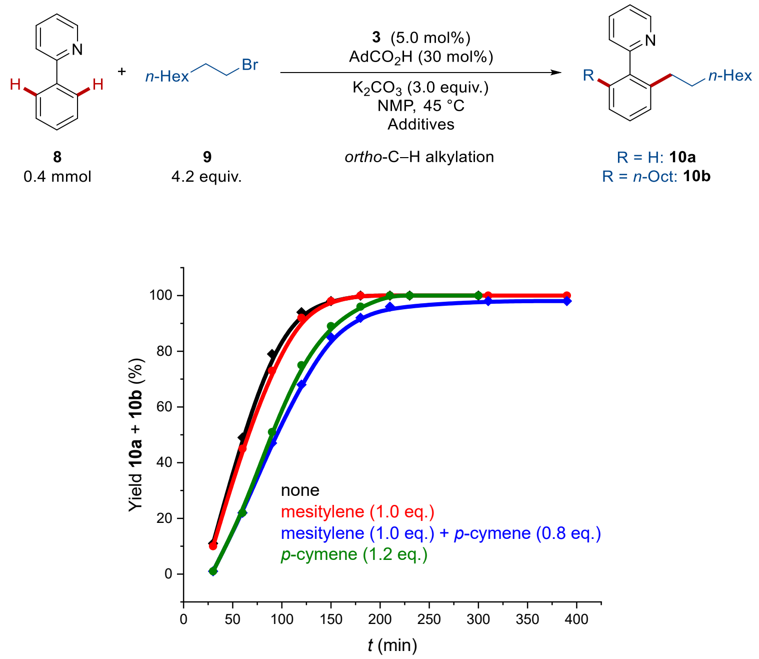

Figure S 20 **The effect of additional *p*-cymene.** The additional mesitylene as the internal standard didn't change the reaction profile, while the additional *p*-cymene lead to a longer induction period and slower reaction rate.

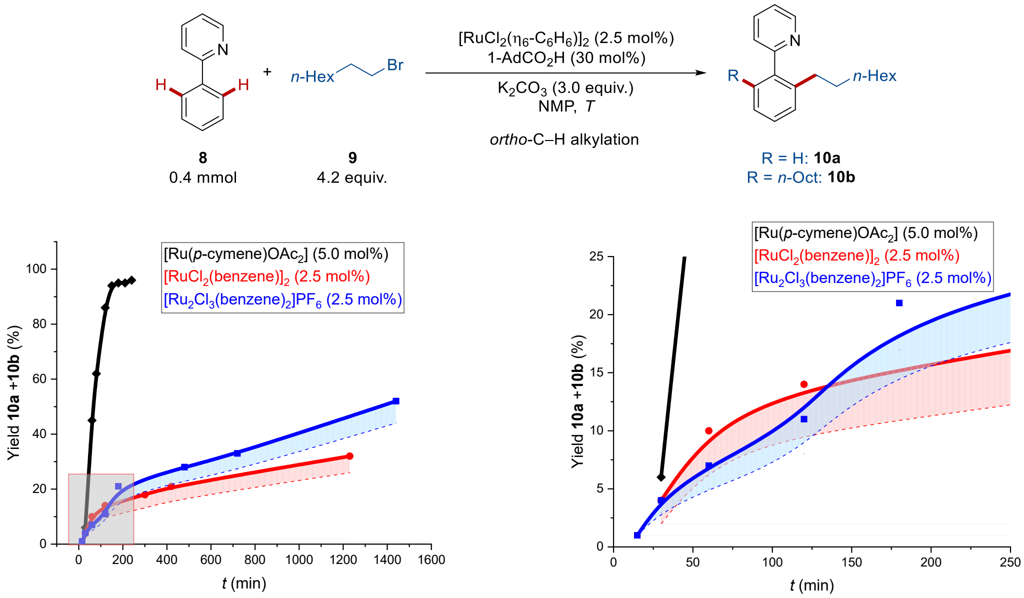

Figure S 21 **Reaction Kinetics for Ruthenium Benzene complexes.** The solubility of $[RuCl_2(\eta_6\text{-}C_6H_6)]_2$ in NMP is not good, making it unsuitable for kinetic analysis. Nevertheless, a shorter induction time for ruthenium-benzene complexes than for ruthenium carboxylates could be deduced from the reaction trends.

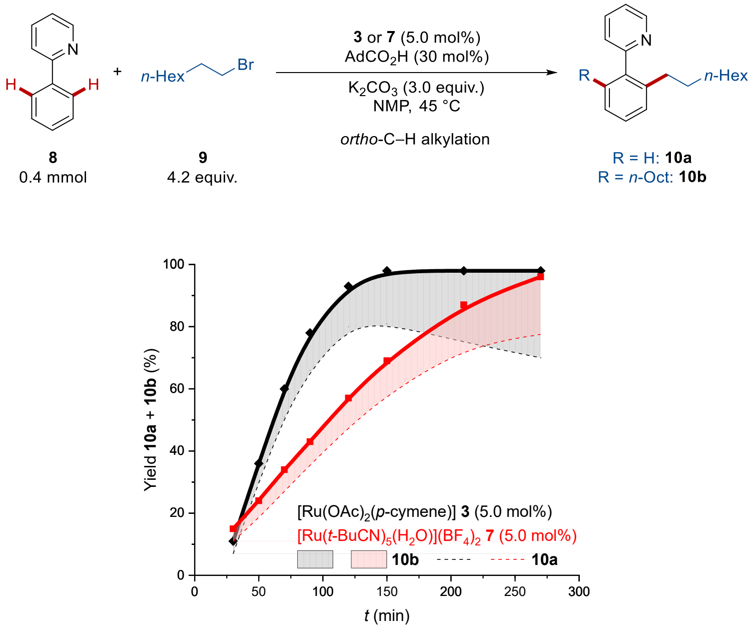

Figure S 22 Reaction Profiles Using 5 mol% of 3 and 7.

5.3 Arrhenius Plot Analysis

5.3.1 Ruthenium-catalyzed *ortho*-C–H Alkylation

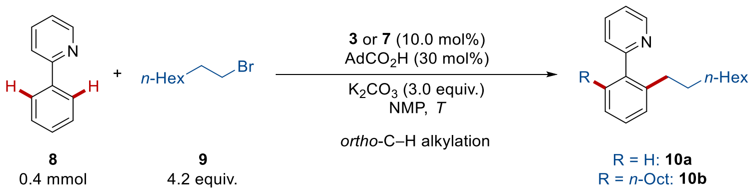

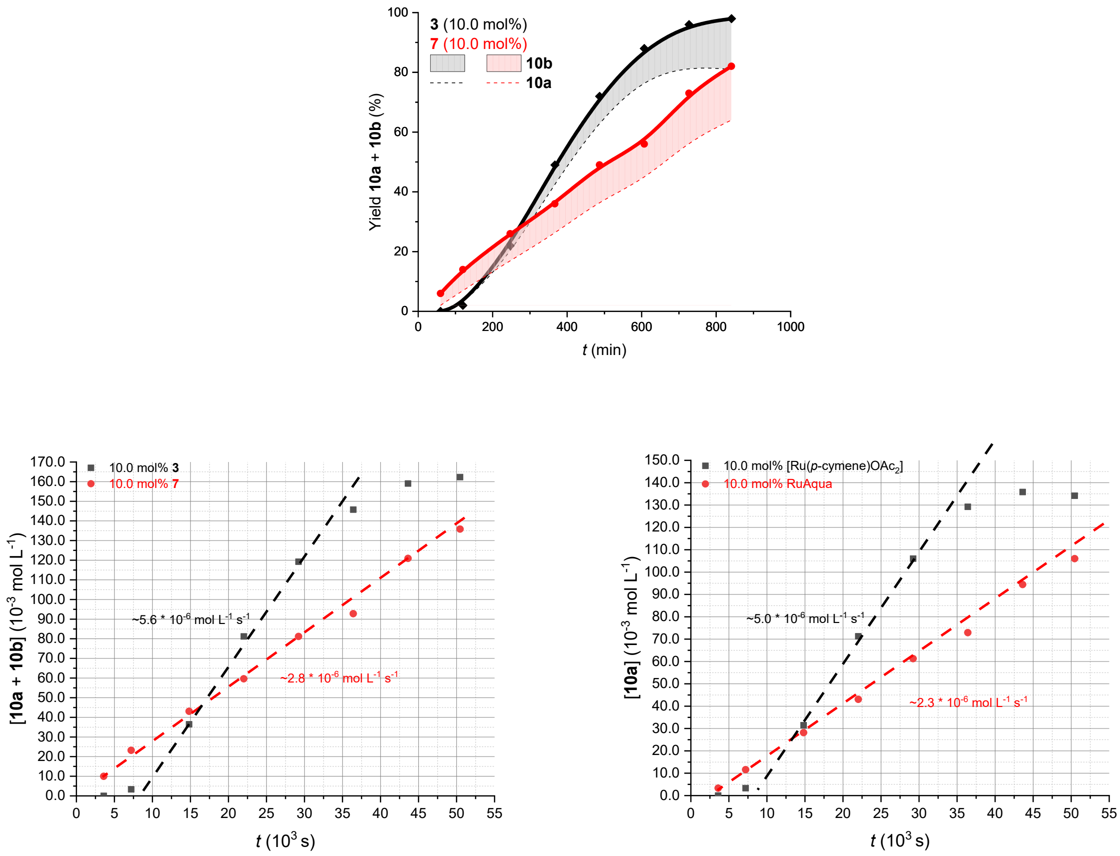

Figure S 23 Ruthenium-catalyzed *ortho*-C–H Alkylation at 31°C. Kinetic analyses were conducted respectively based on combined yield and yield of mono-alkylated product.

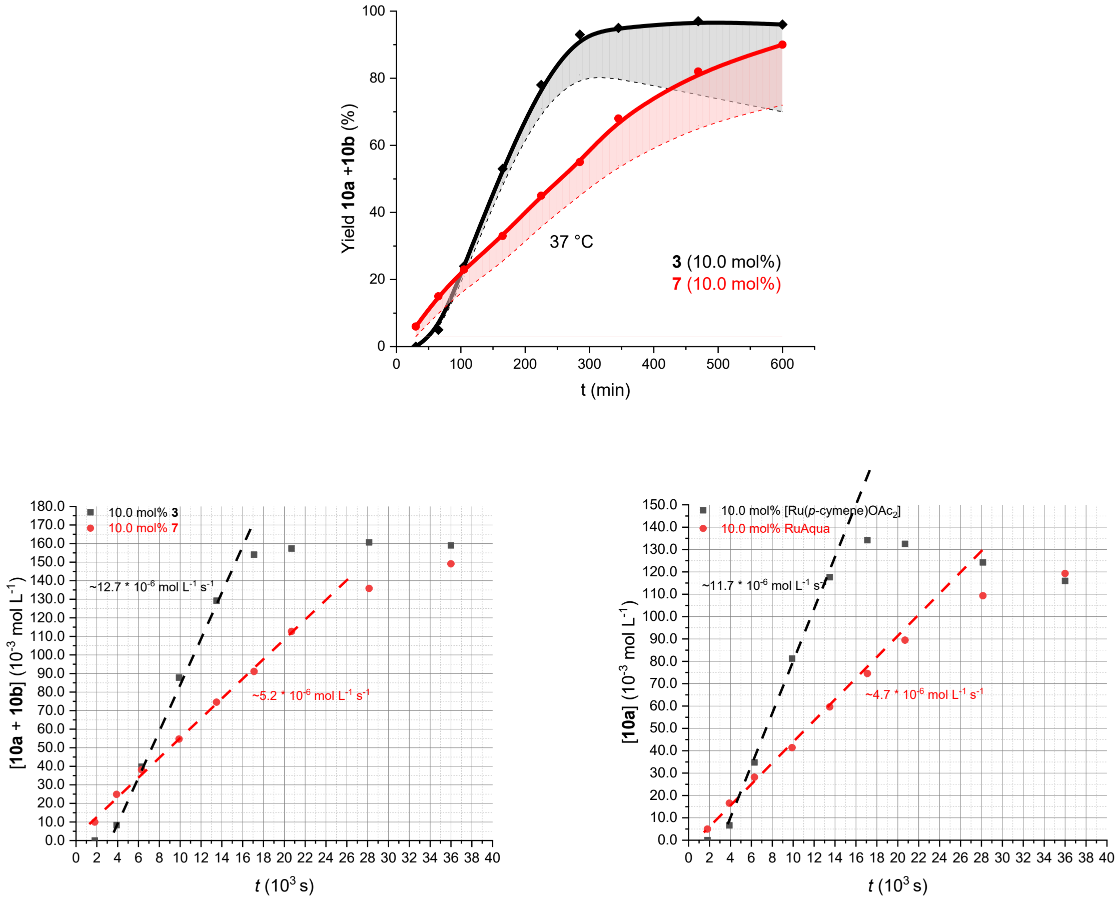

Figure S 24 **Ruthenium-catalyzed *ortho*-C–H Alkylation at 37°C.** Kinetic analyses were conducted respectively based on combined yield and yield of mono-alkylated product.

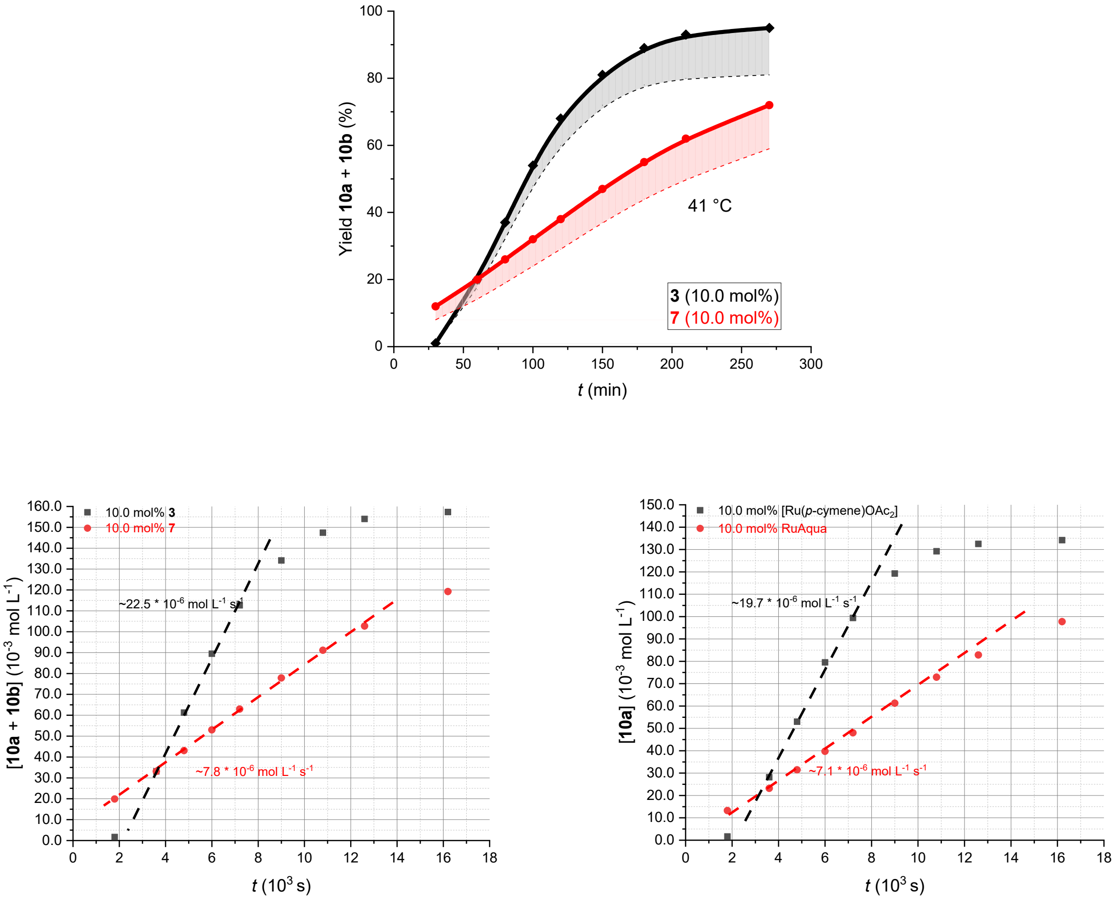

Figure S 25 **Ruthenium-catalyzed *ortho*-C–H Alkylation at 41°C.** Kinetic analyses were conducted respectively based on combined yield and yield of mono-alkylated product.

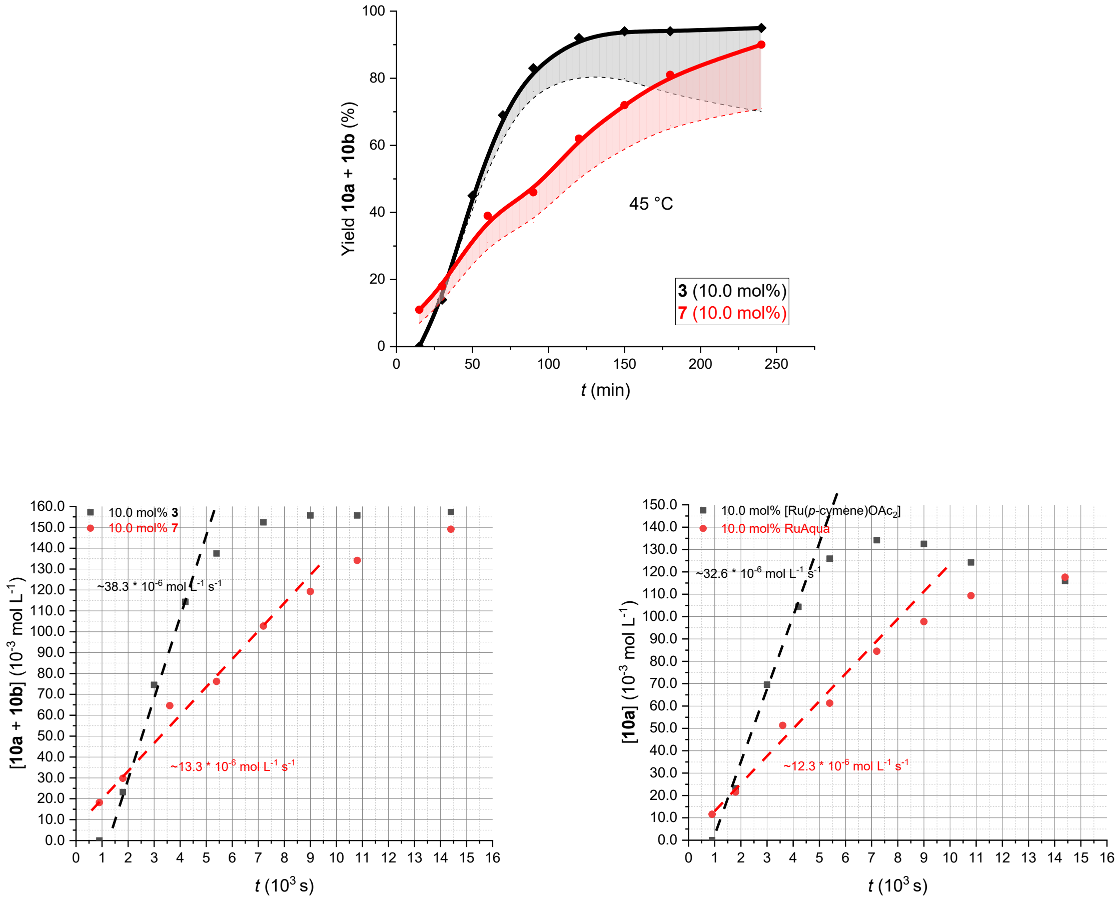

Figure S 26 **Ruthenium-catalyzed *ortho*-C–H Alkylation at 45°C.** Kinetic analyses were conducted respectively based on combined yield and yield of mono-alkylated product.

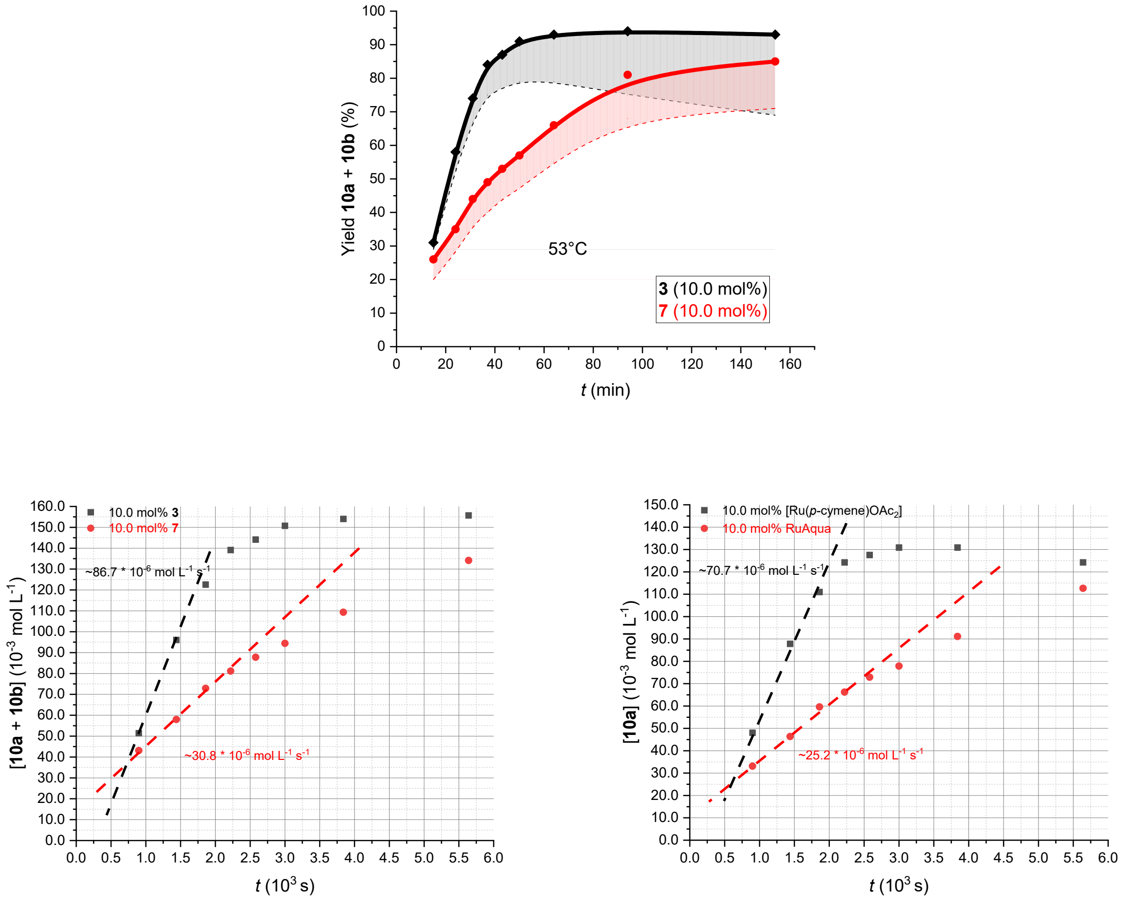

Figure S 27 Ruthenium-catalyzed *ortho*-C–H Alkylation at 53°C. Kinetic analyses were conducted respectively based on combined yield and yield of mono-alkylated product.

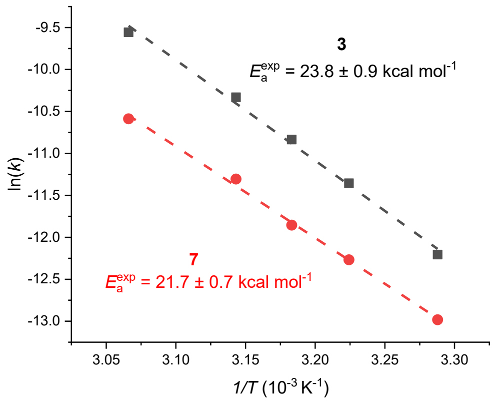

Figure S 28 Temperature-resolved Arrhenius plot analysis for *ortho* alkylation. Kinetic analyses were conducted based on the yield of **10a**.

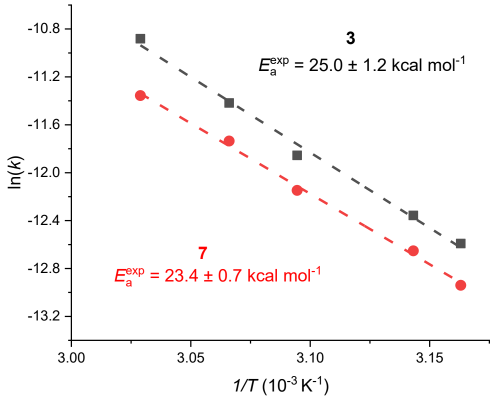

Figure S 29 Temperature-resolved Arrhenius plot analysis for *meta* alkylation. Kinetic analyses were conducted based on the yield of **26a**.

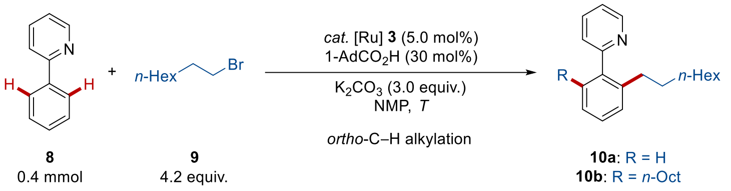

90 min, 80% conversion

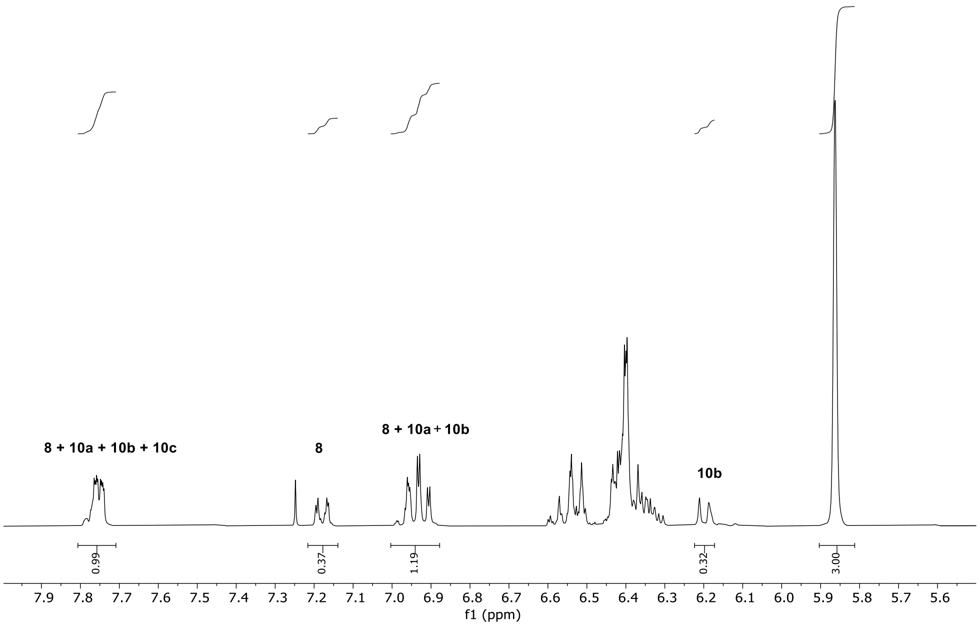

330 min, full conversion

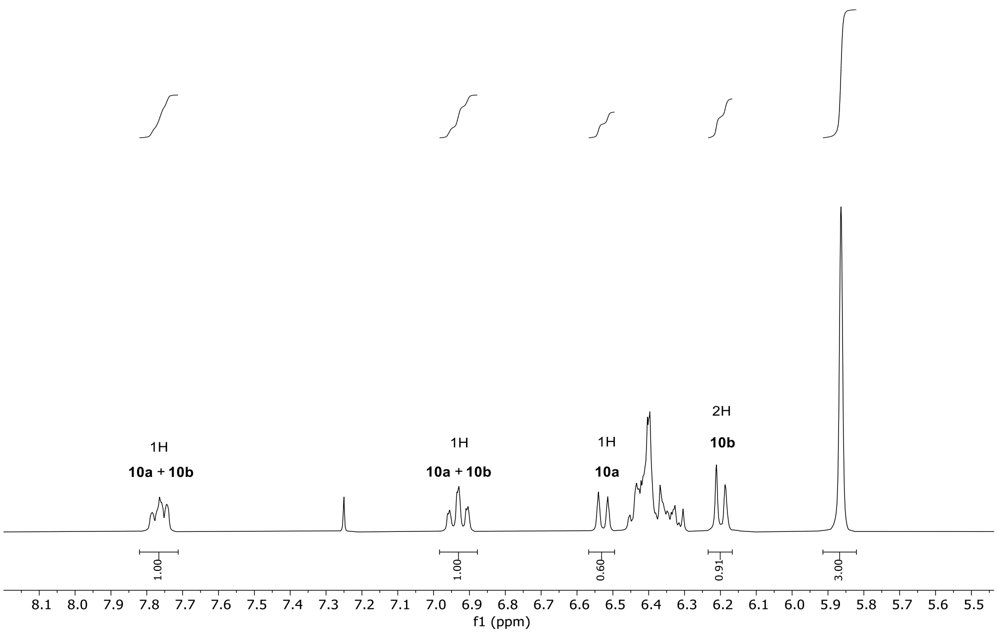

Figure S 30 Mass balance of *ortho*-alkylation.

5.3.2 Ruthenium-catalyzed *meta*-C–H Alkylation

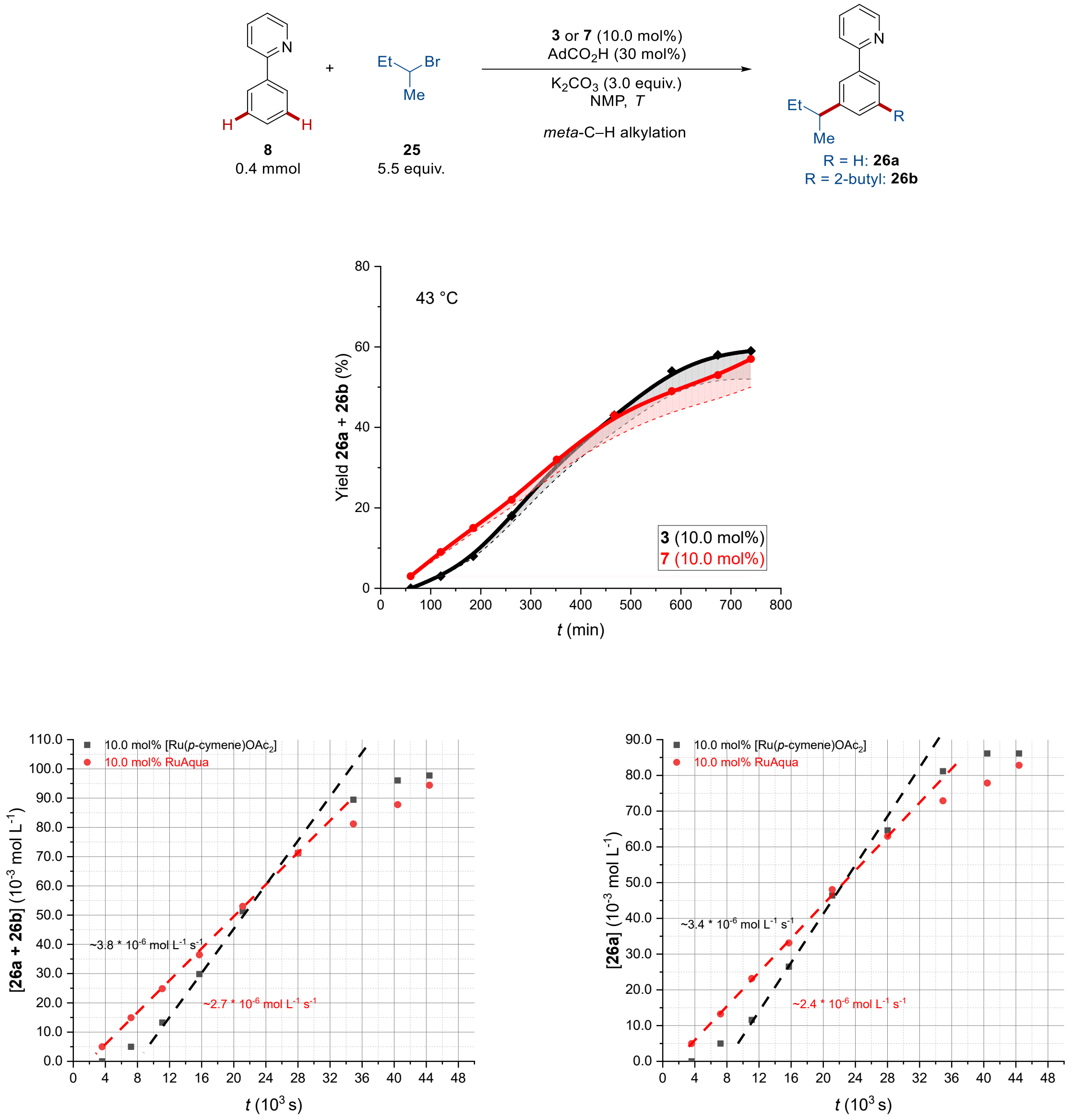

Figure S 31 Ruthenium-catalyzed *meta*-C–H Alkylation at 43°C. Kinetic analyses were conducted respectively based on combined yield and yield of mono-alkylated product.

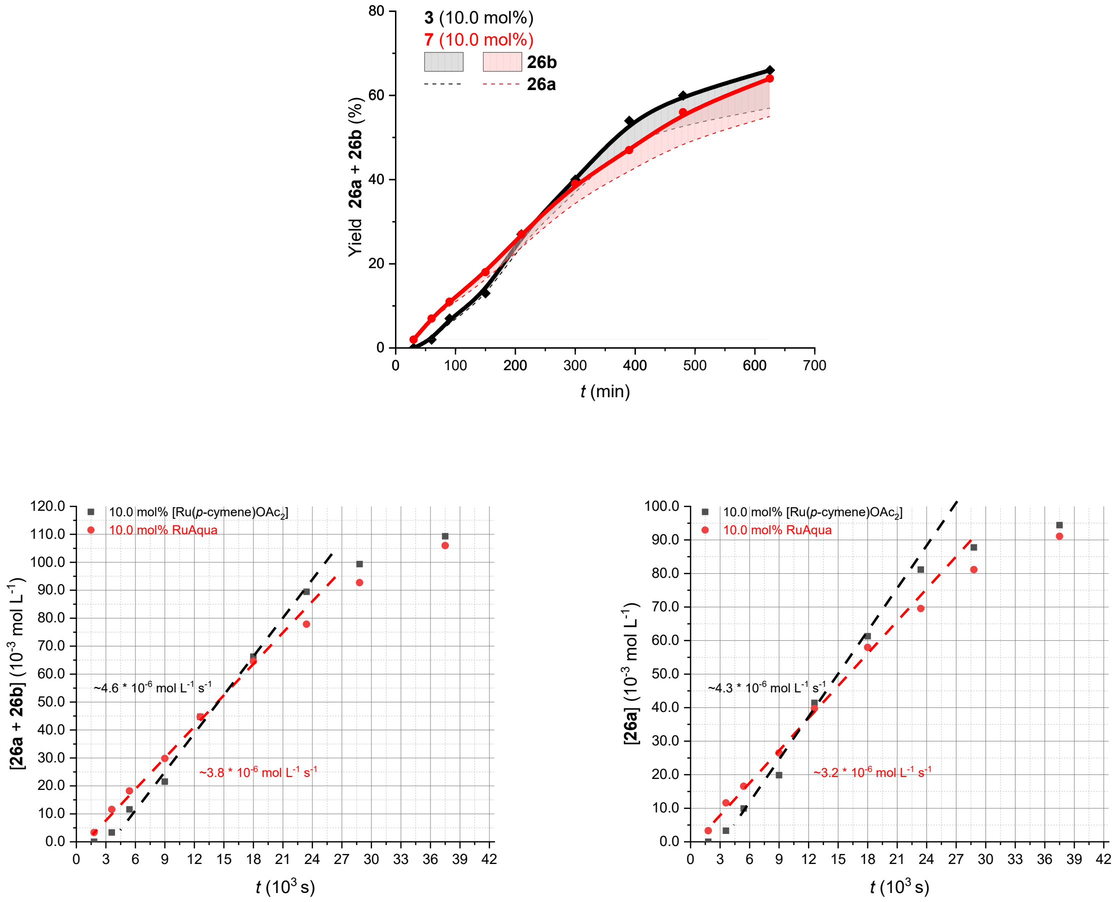

Figure S 32 **Ruthenium-catalyzed *meta*-C–H Alkylation at 45°C.** Kinetic analyses were conducted respectively based on combined yield and yield of mono-alkylated product.

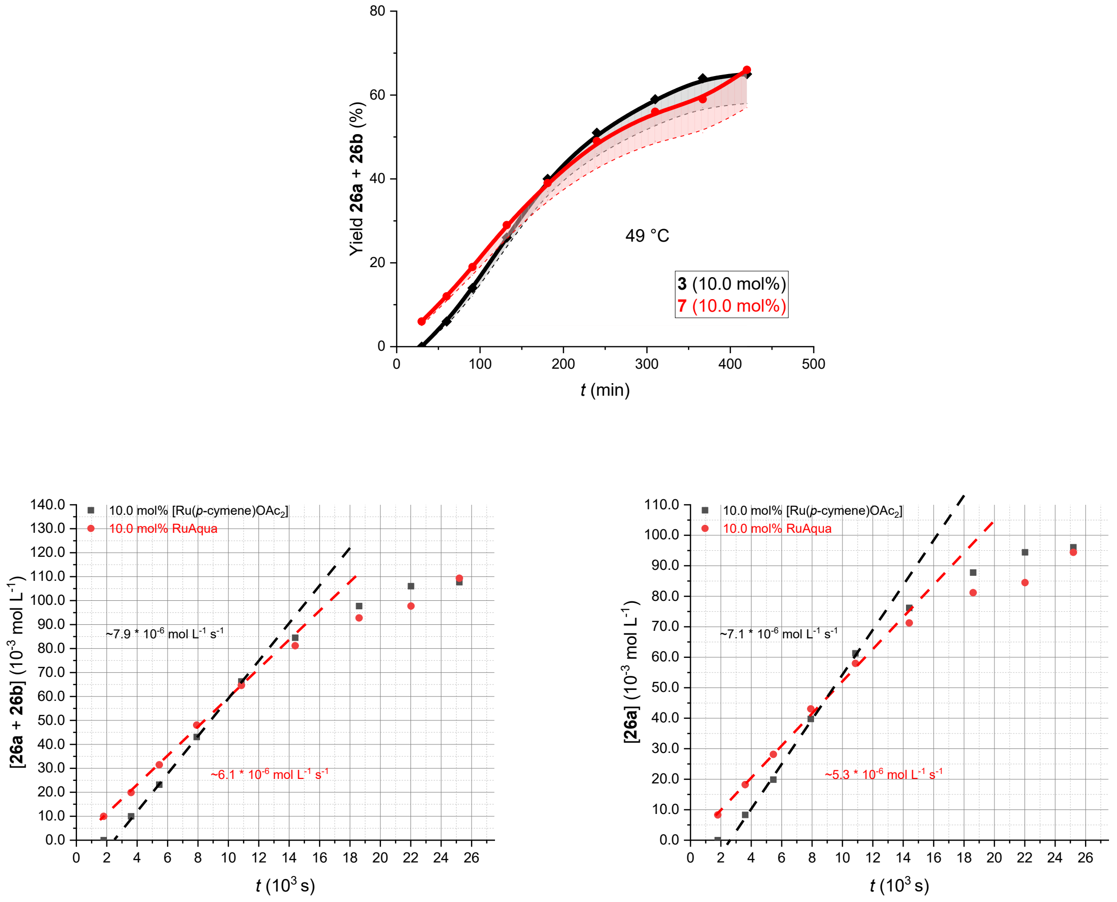

Figure S 33 Ruthenium-catalyzed *meta*-C–H Alkylation at 49°C. Kinetic analyses were conducted respectively based on combined yield and yield of mono-alkylated product.

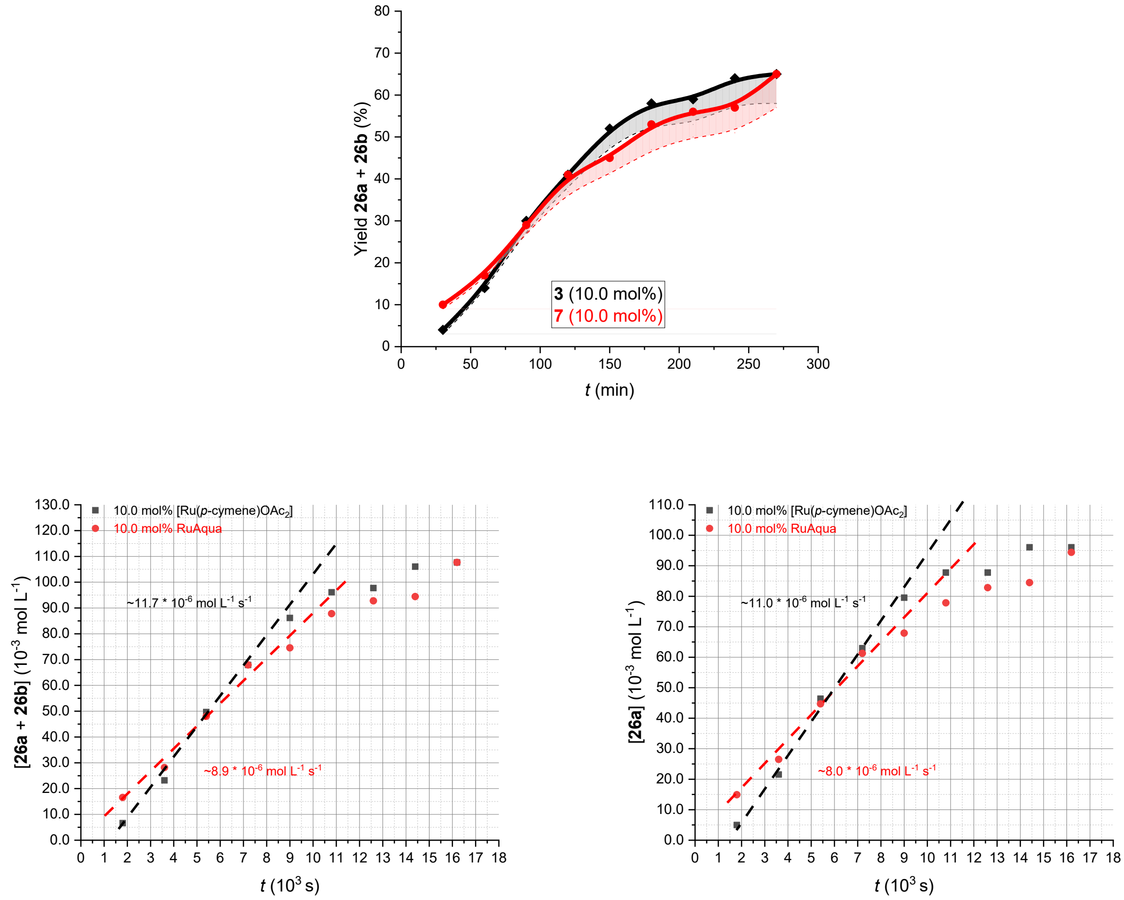

Figure S 34 **Ruthenium-catalyzed *meta*-C–H Alkylation at 53°C.** Kinetic analyses were conducted respectively based on combined yield and yield of mono-alkylated product.

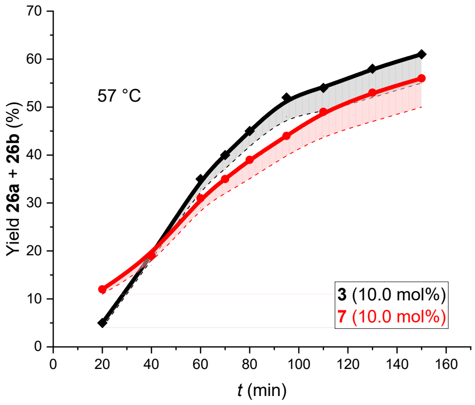

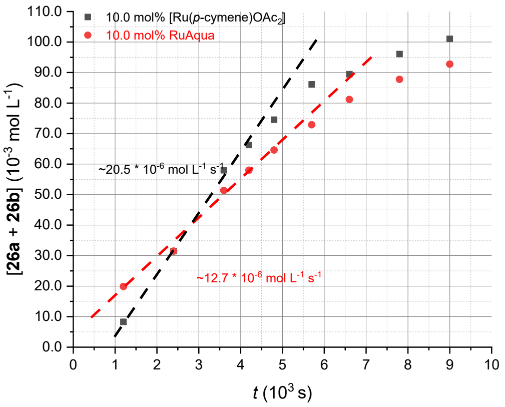

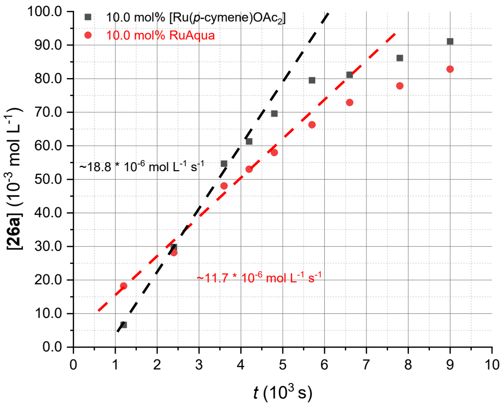

Figure S 35 Ruthenium-catalyzed *meta*-C–H Alkylation at 57°C. Kinetic analyses were conducted respectively based on combined yield and yield of mono-alkylated product.

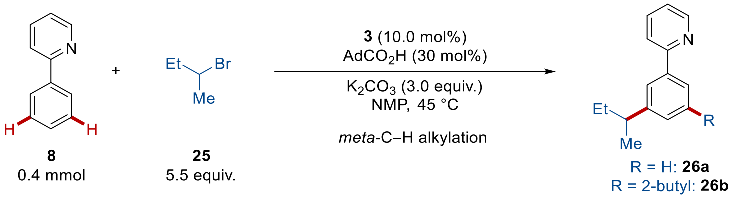

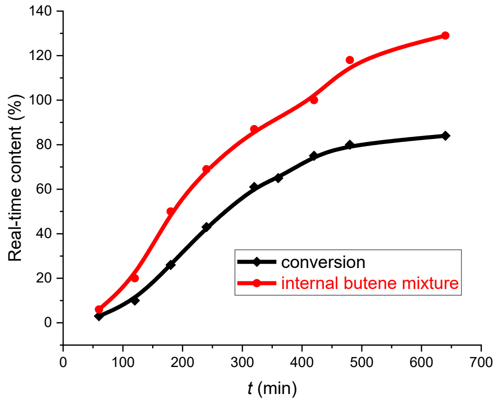

Figure S 36 ***β*****-Hydride Elimination for *meta*-C–H Alkylation.** Reaction condition: [Ru(OAc)$_2$(*p*-cymene)] (10 mol%), 45 °C.

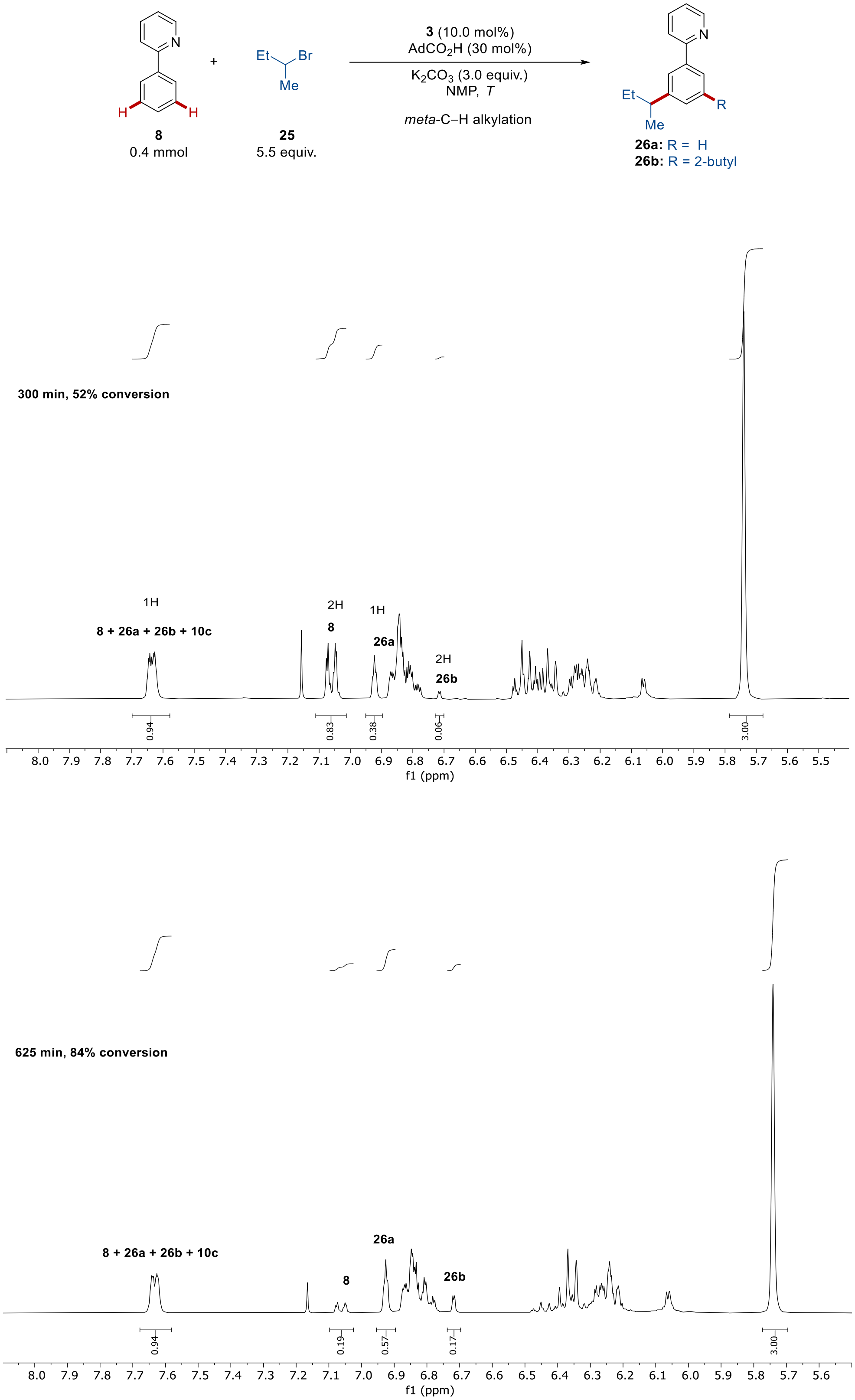

Figure S 37 Mass balance of *meta*-alkylation.

5.4 Substrate-Dependent Kinetic Analysis

Reaction conditions: **phenyl pyridine derivatives** (0.80 mmol), **mesitylene** (0.80 mmol), **octyl bromide** (0.60 mL), **[Ru(OAc)$_2$(*p*-cymene)]** or **[Ru(*t*-BuCN)$_5$(H$_2$O)](BF$_4$)$_2$** (5.0 mol %), **1-AdCO$_2$H** (30 mol %), NMP (4.0 mL) were mixed in the glovebox to form a homogeneous solution. Then 0.5 mL of this reaction mixture and K_2CO_3 (43.0 mg) was added to a vial with a stirring bar and sealed with a cap, the procedure was repeated 9 times to produce 9 reactions in the same conditions. The reaction vials were placed into a pre-heated alumina block (45 °C) and stirred with a rate of 500 rpm. The reaction temperature was monitored with an exogenous thermal meter. After specific time intervals, one reaction vial was transferred outside the glovebox. The reaction mixture was added with 0.5 mL $CDCl_3$ and filtered with a short pad of Celite, and then analyzed by ^{1}H NMR.

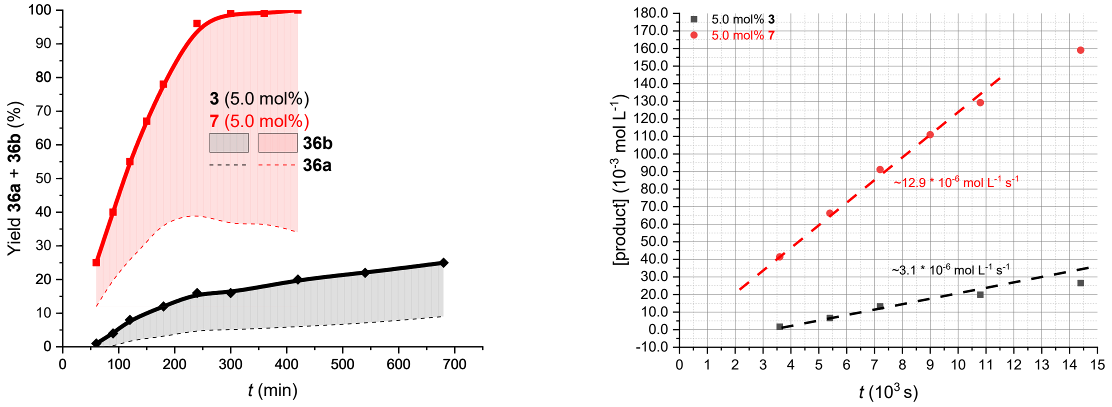

Figure S 38 **Ru-catalyzed *ortho*-C–H Alkylation Using Phenyl Pyridine Derivatives (*p*-CO$_2$Me).**

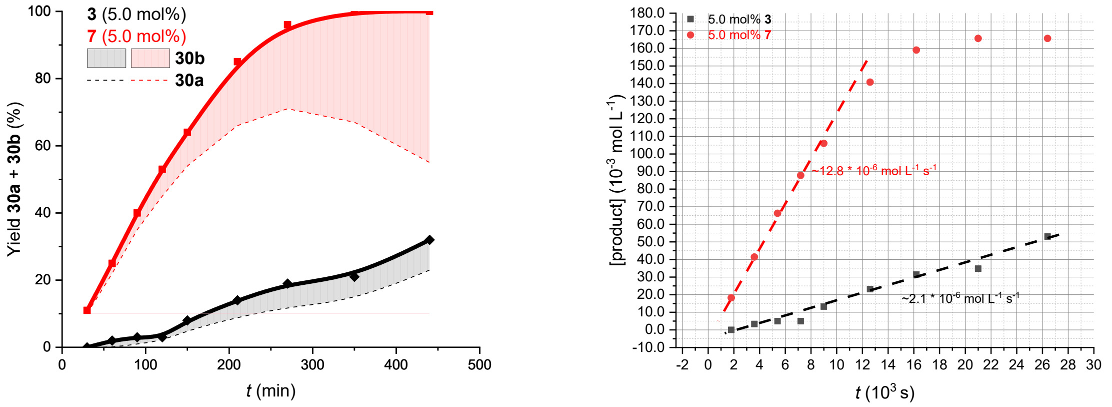

Figure S 39 **Ru-catalyzed *ortho*-C–H Alkylation Using Phenyl Pyridine Derivatives (*p*-OMe).**

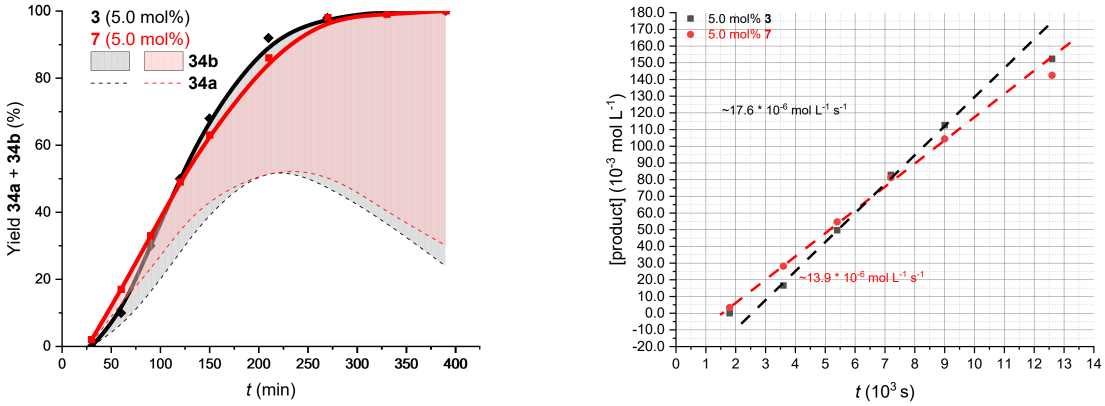

Figure S 40 Ru-catalyzed ***ortho*-C–H Alkylation Using Phenyl Pyridine Derivatives (*p*-F).**

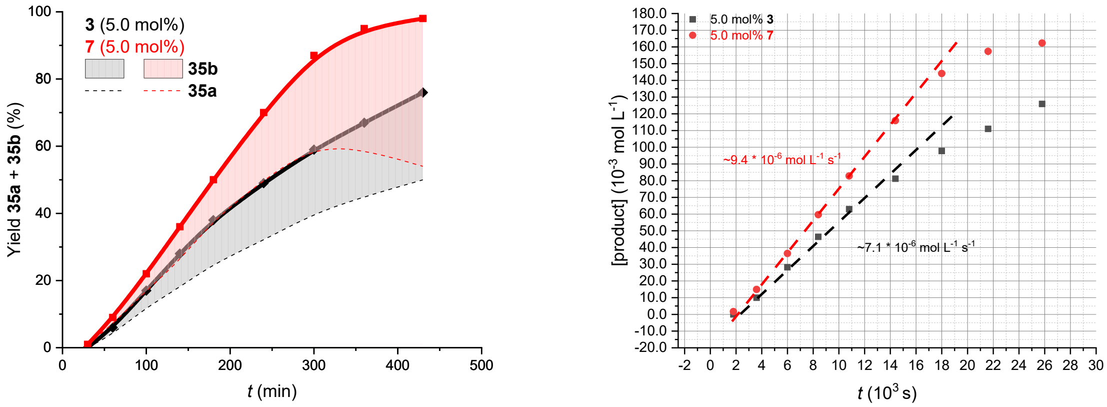

Figure S 41 Ru-catalyzed ***ortho*-C–H Alkylation Using Phenyl Pyridine Derivatives (*p*-CF_3).**

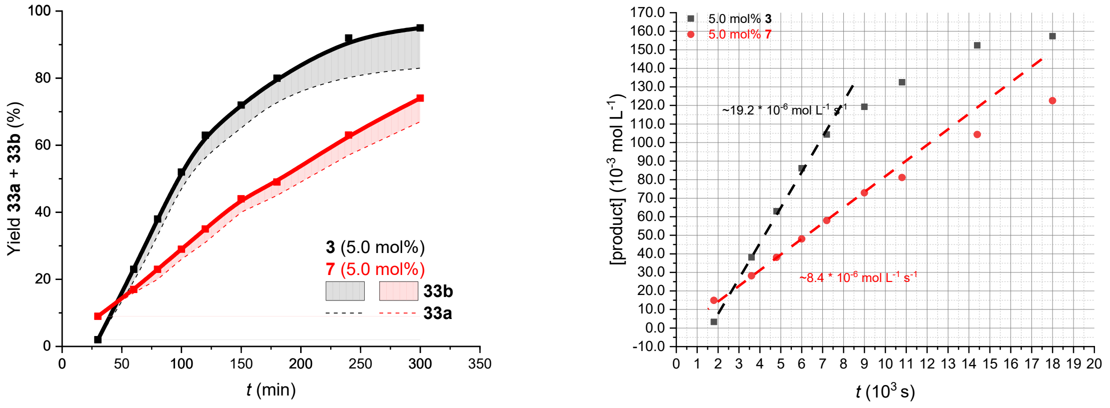

Figure S 42 Ru-catalyzed ***ortho*-C–H Alkylation Using Phenyl Pyridine Derivatives (*p*-Me).**

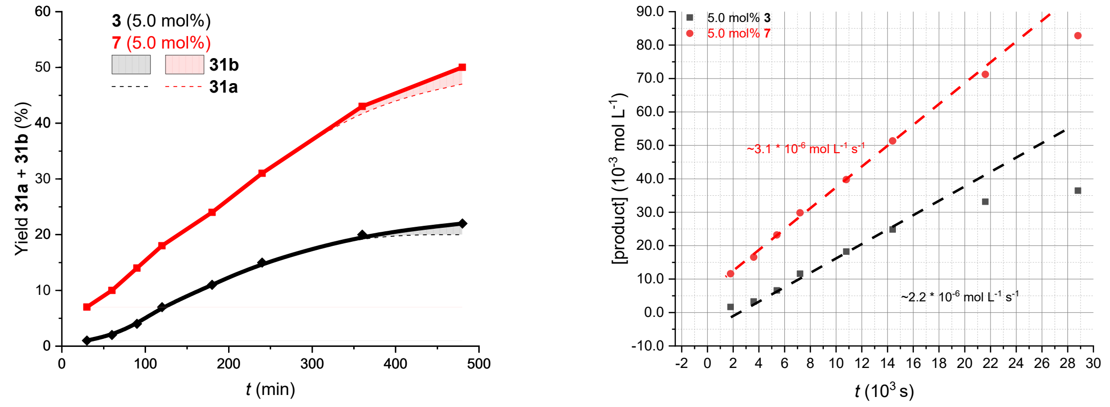

Figure S 43 Ru-catalyzed ***ortho*-C–H Alkylation Using Phenyl Pyridine Derivatives (*p*-NMe$_2$).**

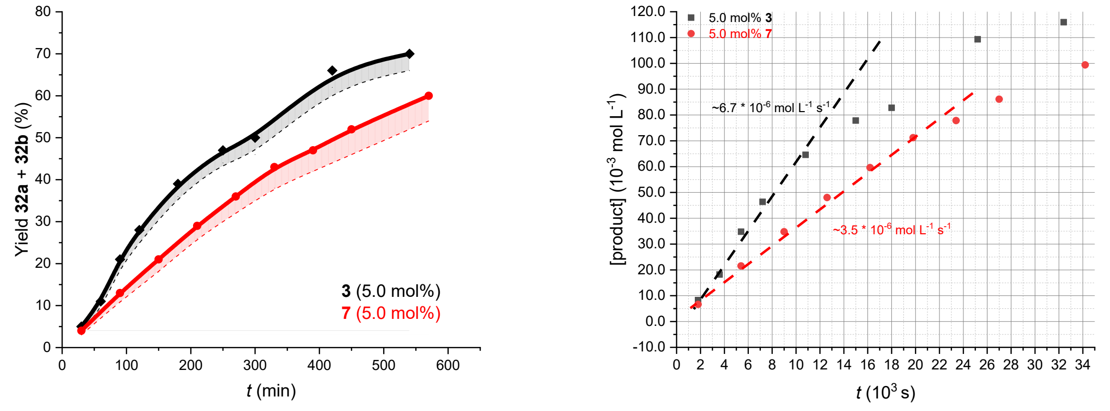

Figure S 44 Ru-catalyzed ***ortho*-C–H Alkylation Using Phenyl Pyridine Derivatives (*p*-*t*Bu).**

Table S 2 **Comparisons of reaction rates for different phenyl pyridine derivatives.**

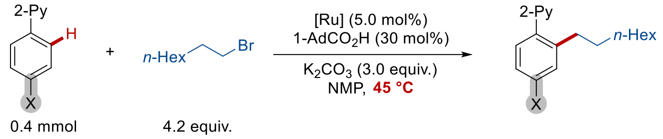

Substituents	Reaction rates (10^{-6} mol L^{-1} s^{-1}) [Ru(OAc)$_2$(*p*-cymene)]	Reaction rates (10^{-6} mol L^{-1} s^{-1}) [Ru(*t*-BuCN)$_5$(H_2O)](BF_4)$_2$
**–NMe$_2$**	2.2	3.1
**–OMe**	2.1	12.8
**–*t*Bu**	6.7	3.5
**–Me**	19.2	8.4
**–H**	33.8	12.5
**–F**	17.6	13.9
**–CO$_2$Me**	3.1	12.9
**–CF$_3$**	7.1	9.4

Reaction rates during steady state catalytic cycle for both catalysts have been summarized, with catalyst **3** showing a faster reaction rate in half of the examples (Table S2). RuAqua was able to maintain the same reaction kinetics over a wider range of substrates, and arene ruthenium catalyst **3** was recognized as having promising potential, with a reaction rate up to 33.8*10^{-6} mol L^{-1} s^{-1}. We assumed that the stronger interference resistance of catalyst **7** at the substituents was due to the strongly coordinating pivalonitrile ligands, whereas the potentially faster reaction rate of catalyst **3** was due to the less coordinating interference.

Table S 3 **Summary of mass balance.**

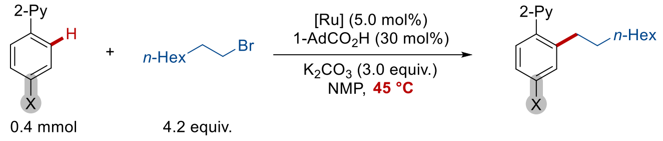

Substituents	Mass balance (SM + mono + di)			
	$[Ru(OAc)_2(p\text{-cymene})]$		$[Ru(t\text{-BuCN})_5(H_2O)](BF_4)_2$	
	Observed	Default	Observed	Default
**$-NMe_2$**	97%[a]	97%	97%[a]	96%
**−OMe**	97%	97%	96%	97%
**−*t*Bu**	103%[a]	100%	99%[a]	100%
**−Me**	99%	97%	99%	96%
**−H**	105%	100%	104%	103%
**−F**	99%	94%	97%	95%
**$-CO_2Me$**	101%	99%	101%	100%
**$-CF_3$**	100%	100%	98%	98%

Mass balances were obtained from the last data points of the profiles. See Figure S45–61 for more details. [a] Traces of di-alkylated products were observed.

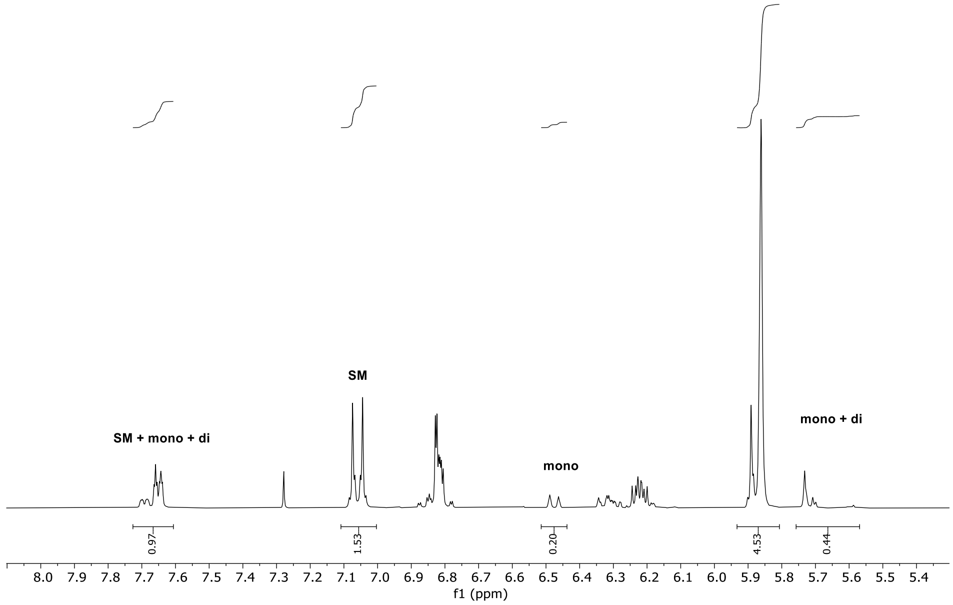

Figure S 45 **$[Ru(OAc)_2(p\text{-cymene})]$_$NMe_2$.** The peak of IS overlaps with the 2H peak of substrate.

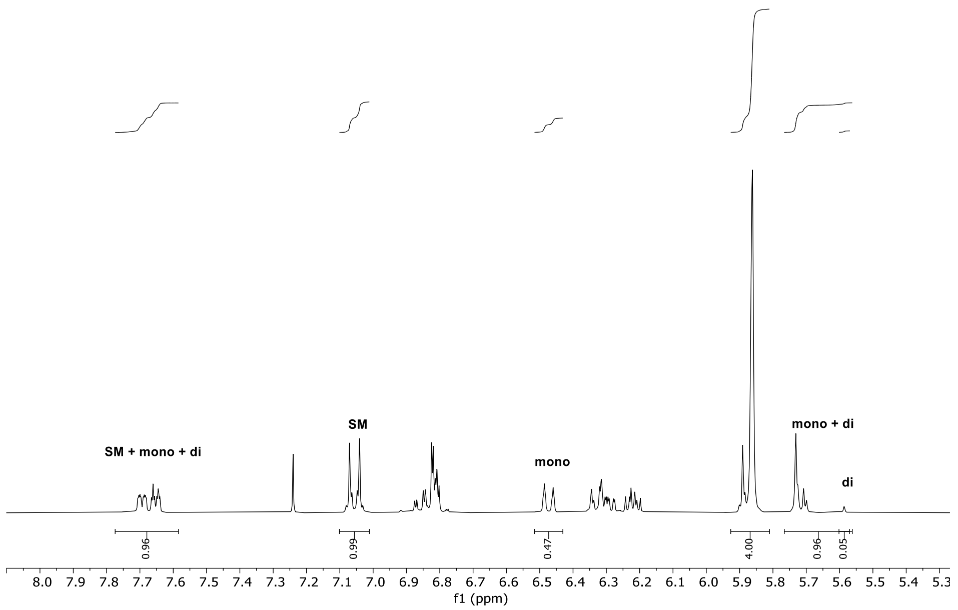

Figure S 46 **RuAqua_NMe$_2$.** The peak of IS overlaps with the 2H peak of substrate.

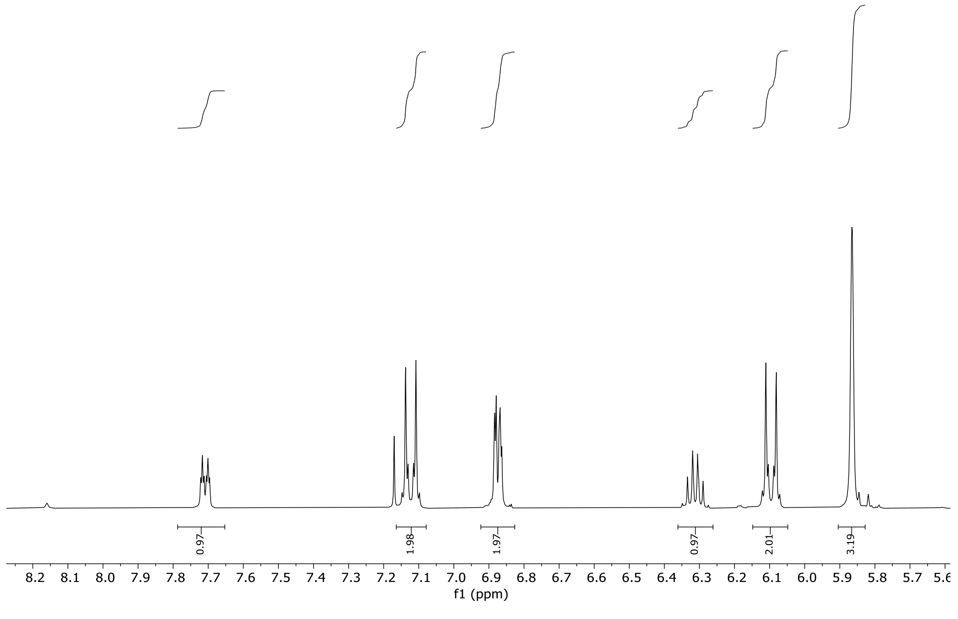

Figure S 47 **[Ru(OAc)$_2$(*p*-cymene)]_OMe_the first data point.** The peak of IS overlaps with unidentified peaks, therefore the yield was calculated by setting the integrals of the peak at 7.7 ppm to 0.97.

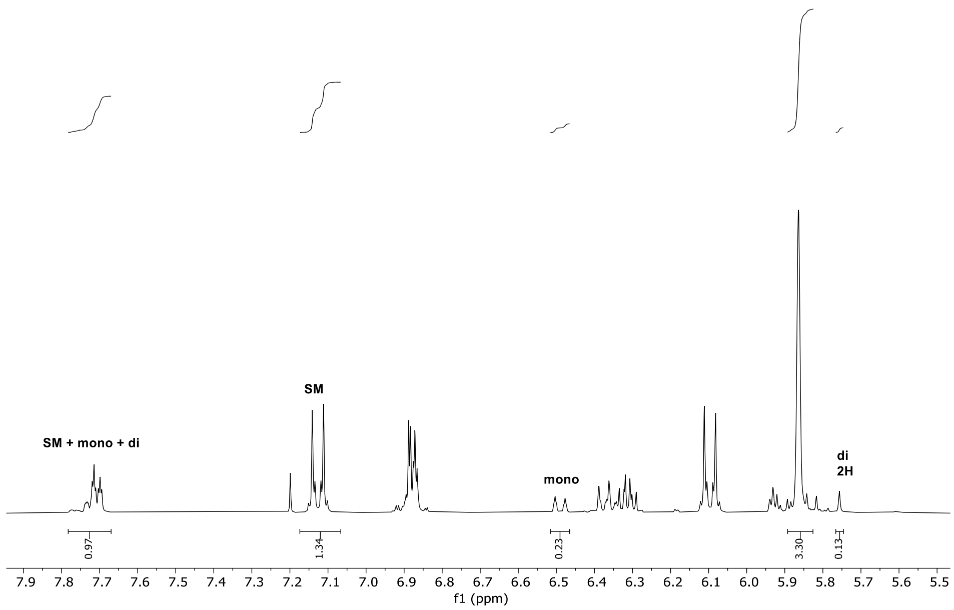

Figure S 48 **[Ru(OAc)$_2$(*p*-cymene)]_OMe.** The peak of IS overlaps with unidentified peaks, therefore the yield was calculated by setting the integrals of the peak at 7.7 ppm to 0.97.

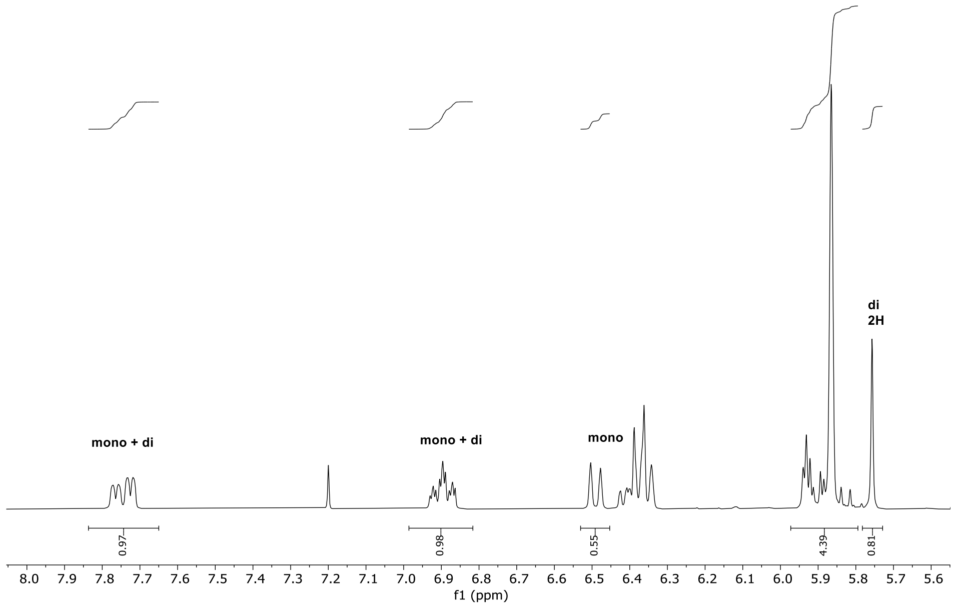

Figure S 49 **RuAqua_OMe.** The peak of IS overlaps with unidentified peaks, therefore the yield was calculated by setting the integrals of the peak at 7.7 ppm to 0.97.

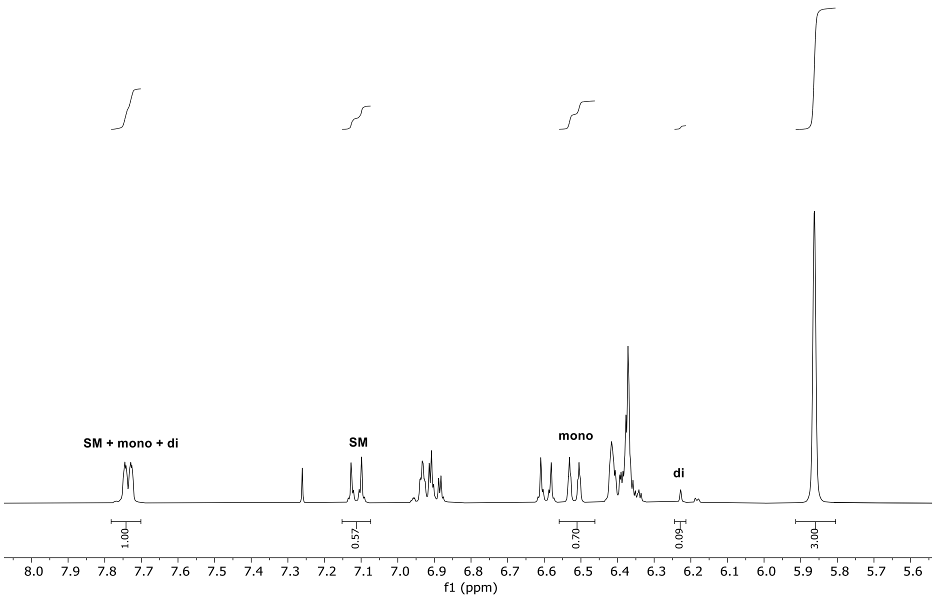

Figure S 50 **[Ru(OAc)$_2$(*p*-cymene)]_*t*Bu.**

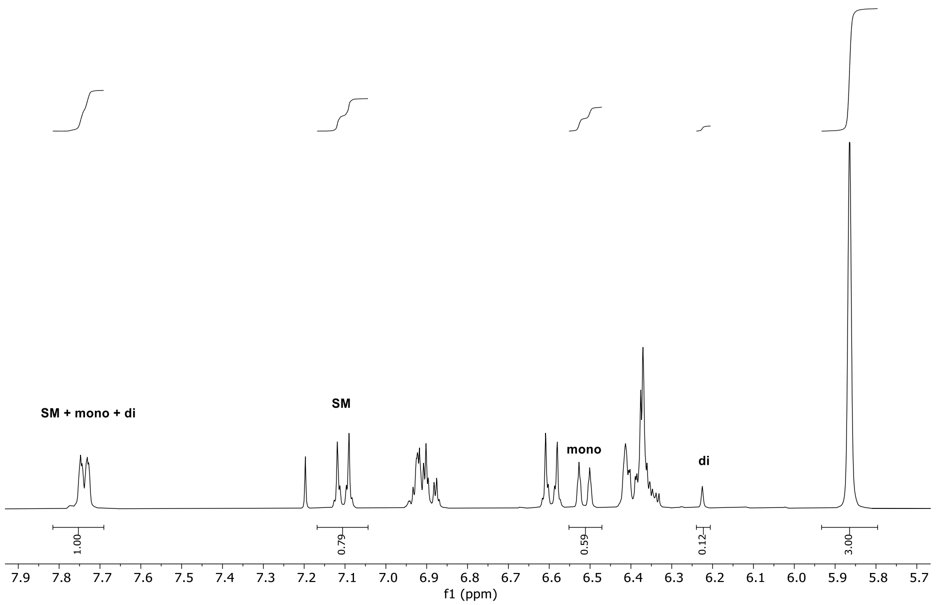

Figure S 51 **RuAqua_*t*Bu.**

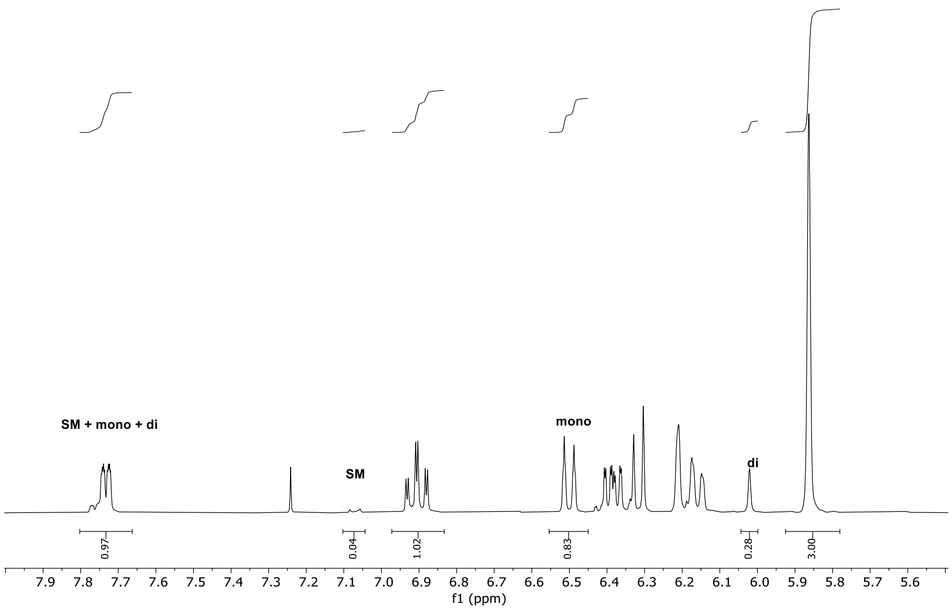

Figure S 52 [Ru(OAc)$_2$(*p*-cymene)]_Me.

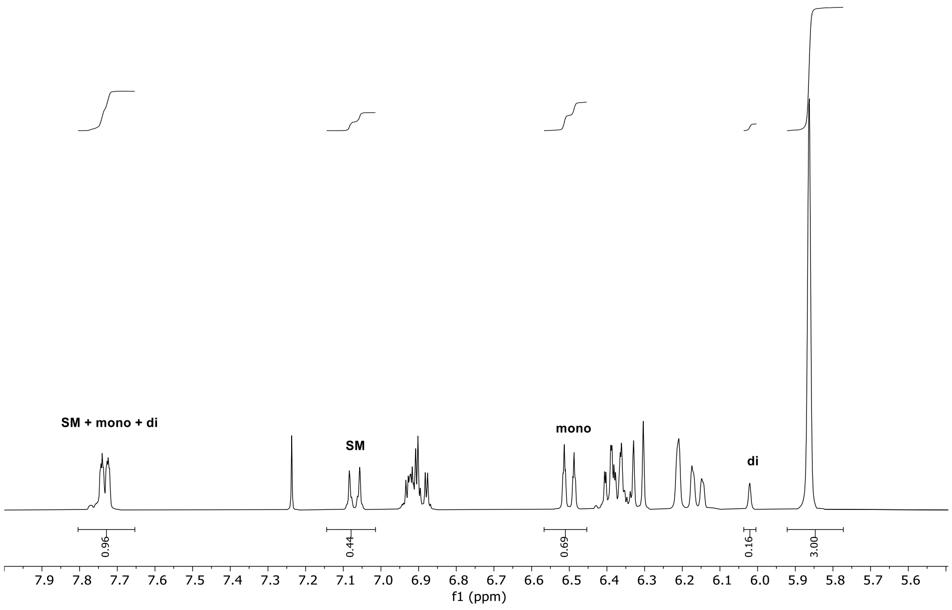

Figure S 53 RuAqua_Me.

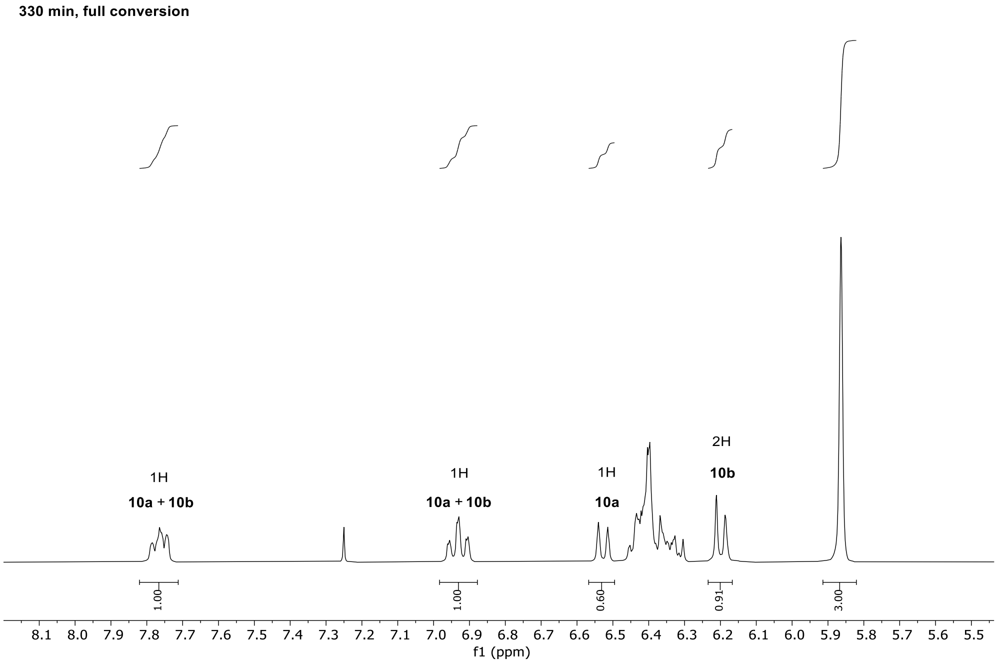

Figure S 54 **[Ru(OAc)$_2$(*p*-cymene)]_H.** The mass balance obtained from NMR integration is over 100% due to the 2H peak of **10b** at about 6.2 ppm overlaps with the other peaks.

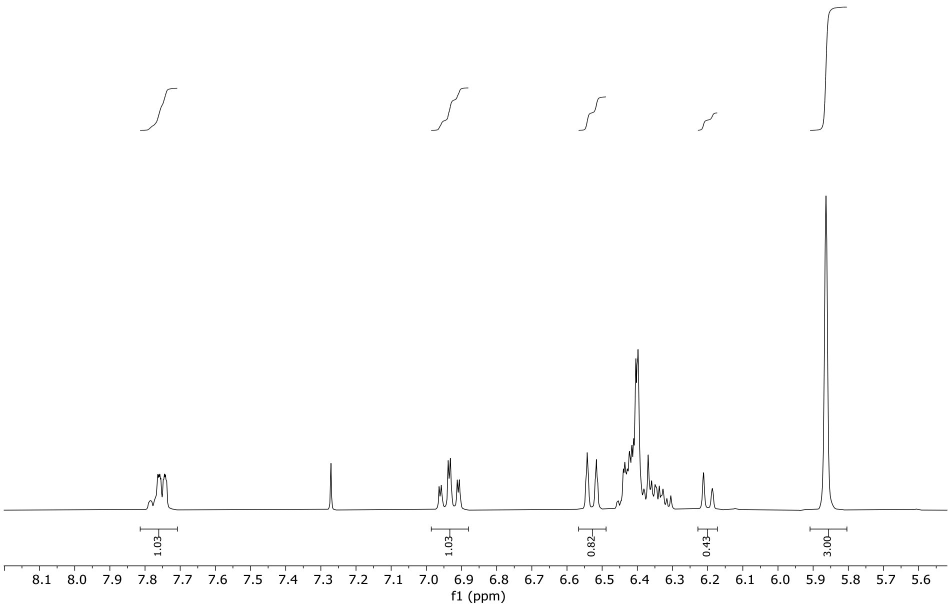

Figure S 55 **RuAqua_H.** The mass balance obtained from NMR integration is over 100% due to the 2H peak of **10b** at about 6.2 ppm overlaps with the other peaks.

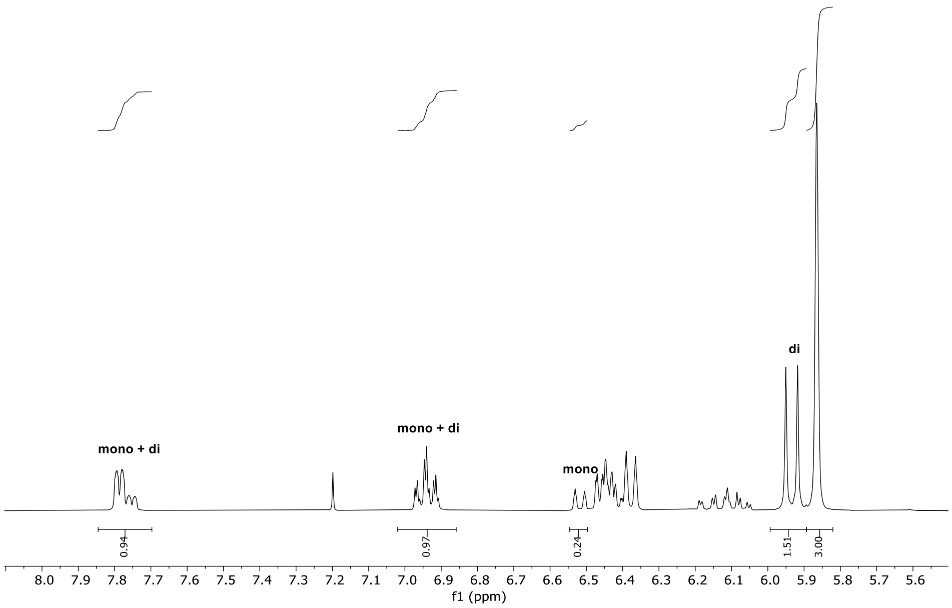

Figure S 56 [Ru(OAc)$_2$(*p*-cymene)]_F.

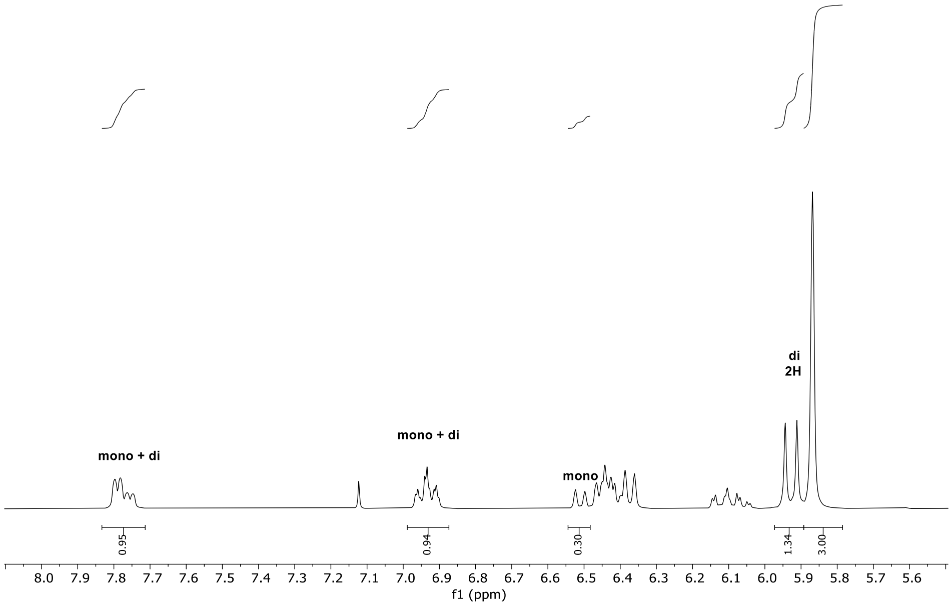

Figure S 57 RuAqua_F.

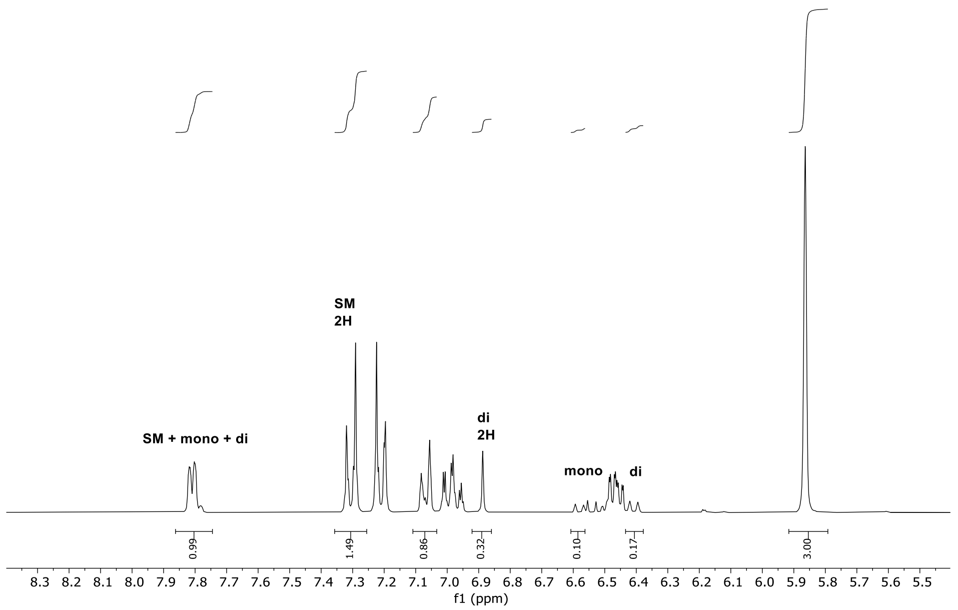

Figure S 58 **[Ru(OAc)$_2$(*p*-cymene)]_CO$_2$Me.**

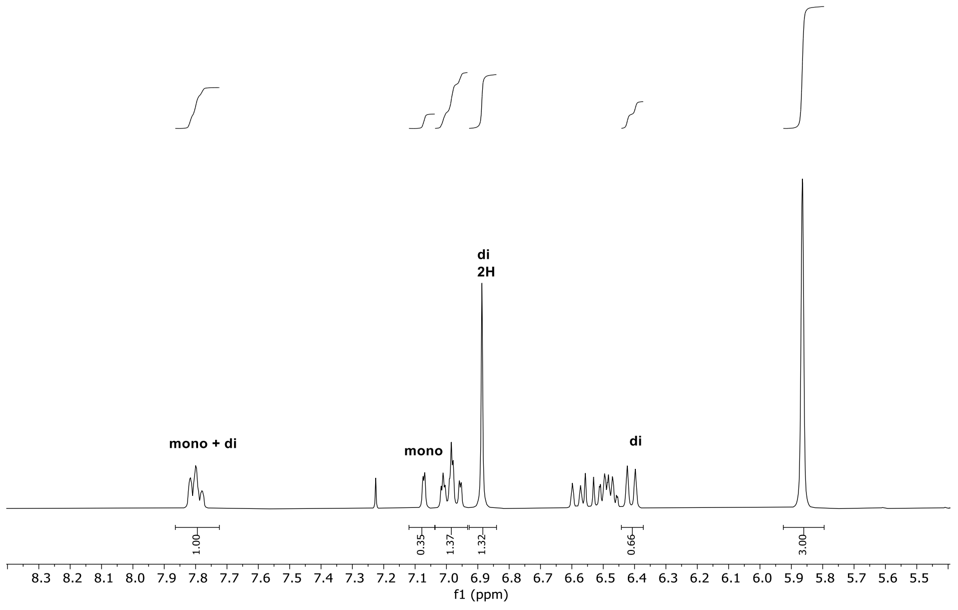

Figure S 59 **RuAqua_CO$_2$Me.**

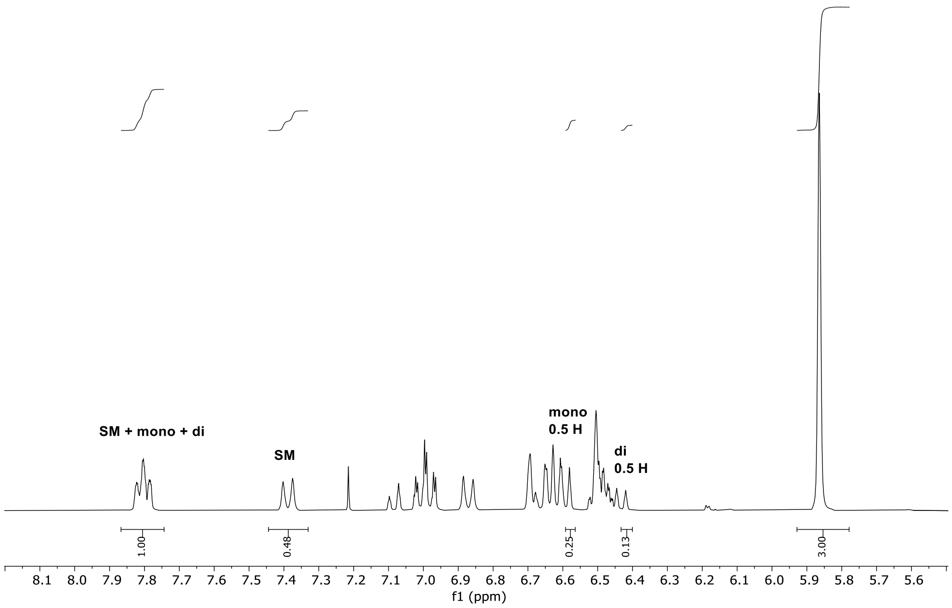

Figure S 60 **[Ru(OAc)$_2$(*p*-cymene)]_CF$_3$.**

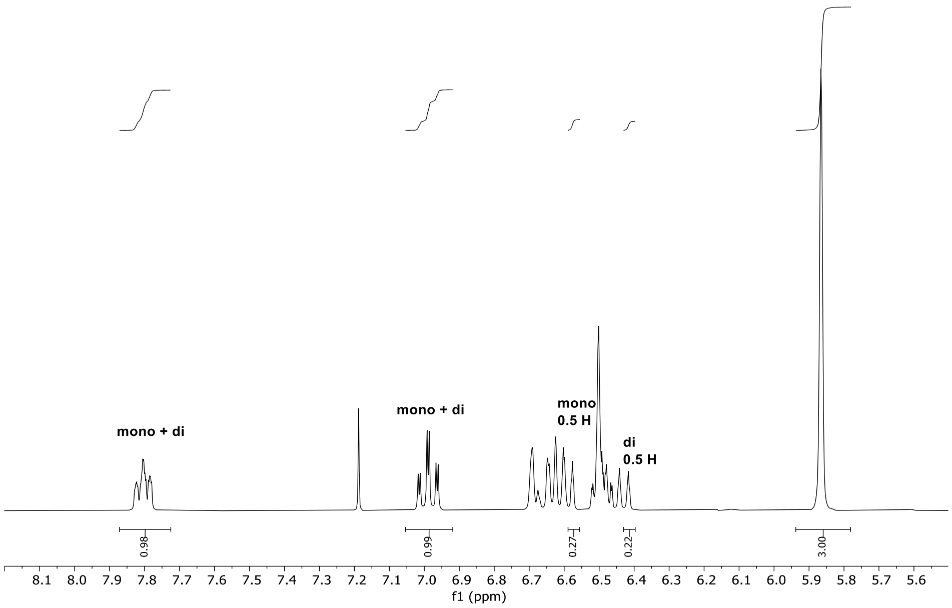

Figure S 61 **RuAqua_CF$_3$.**

5.5 KIE Analysis

5.5.1 Parallel Experiments

Reaction conditions: **phenyl pyridine 8 or 8-*d*$_5$** (0.80 mmol), **mesitylene** (0.80 mmol), **octyl bromide** (0.60 mL), **[Ru(OAc)$_2$(*p*-cymene)]** or **[Ru(*t*-BuCN)$_5$(H$_2$O)](BF$_4$)$_2$** (5.0 mol %), **1-AdCO$_2$H** (30 mol %), NMP (4.0 mL) were mixed in the glovebox to form a homogeneous solution. Then 0.5 mL of this reaction mixture and K_2CO_3 (43.0 mg) was added to a vial with a stirring bar and sealed with a cap, the procedure was repeated 9 times to produce 9 reactions in the same conditions. The reaction vials were placed into a pre-heated alumina block (45 °C) and stirred with a rate of 500 rpm. The reaction temperature was monitored with an exogenous thermal meter. After specific time intervals, one reaction vial was transferred outside the glovebox. The reaction mixture was added with 0.5 mL $CDCl_3$ and filtered with a short pad of Celite, and then analyzed by ^{1}H NMR.

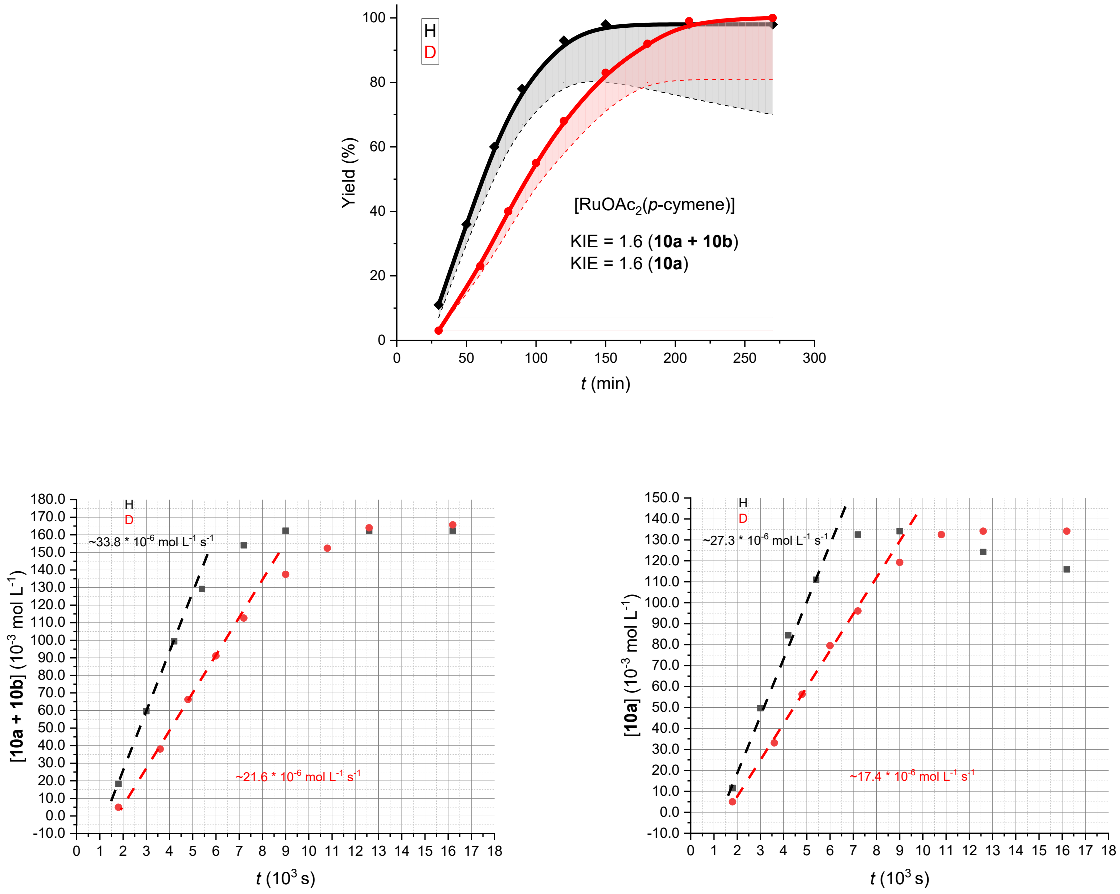

Figure S 62 **KIE Analysis for 3.** Kinetic analyses were conducted respectively based on combined yield and yield of mono-alkylated product.

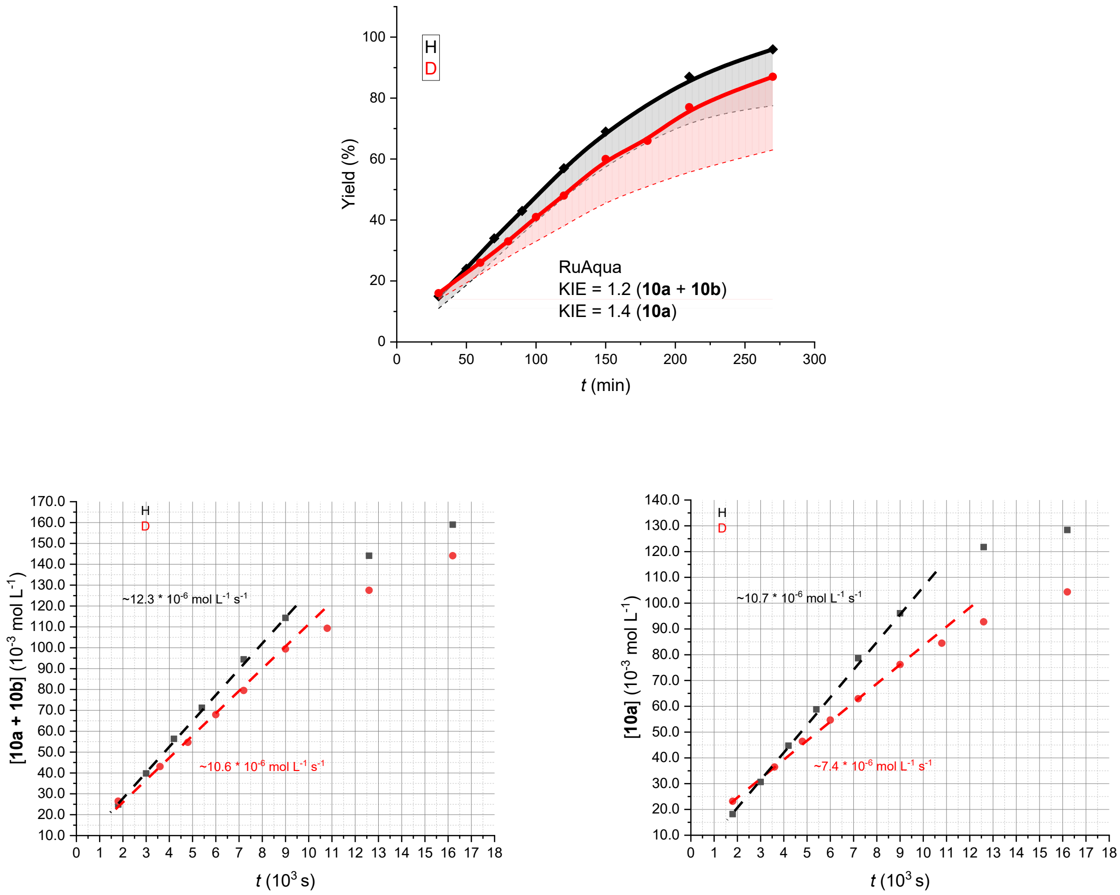

Figure S 63 **KIE Analysis for 7.** Kinetic analyses were conducted respectively based on combined yield and yield of mono-alkylated product.

5.5.2 Intramolecular Competition Experiments

Reaction conditions: **phenyl pyridine 8 and 8-*d*$_1$** (0.20 mmol each), **octyl bromide**, **[Ru(OAc)$_2$(*p*-cymene)]** or **[Ru(*t*-BuCN)$_5$(H$_2$O)](BF$_4$)$_2$** (5.0 mol%), **1-AdCO$_2$H** (30 mol%), K_2CO_3 (166.0 mg), NMP (2.0 mL) were added into a 10 mL glass vial in the glove box. The reaction was performed at 45 °C with a stirring rate of 500 rpm for 1.5 h. After the reaction, the mixture was diluted with EtOAc (5.0 mL) and filtered through a short pad of Celite powder. The filtrate was removed solvent in vacuo. The crude mixture was purified by flash column chromatography on silica gel to obtain the products and recovered starting material for further ^{1}H NMR analysis.

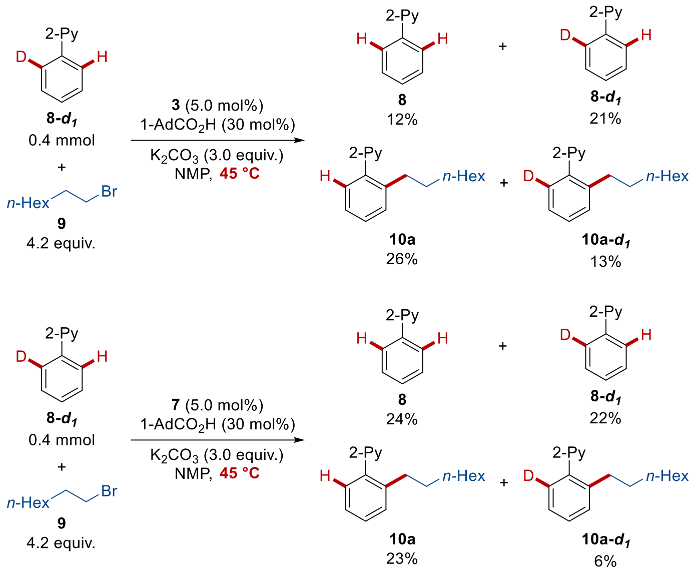

Figure S 64 KIE Studies-Intramolecular Experiments.

6 Characterization

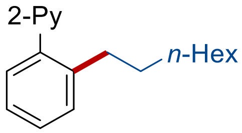

2-(2-octylphenyl)pyridine (10a)

The general procedure **A** was followed using 2-phenylpyridine (**8**) (62.1 mg, 0.4 mmol), *n*-octyl bromide (**9**) (207.3 µL, 1.20 mmol), and [Ru(*p*-cymene)OAc_2] (**3**) (14.1 mg, 10 mol%) in NMP (2.0 mL). The reaction was stirred at 45 °C for 18 h under N_2 atmosphere. Purification by column chromatography (*n*-hexane/EtOAc = 20:1) yielded **10** (**10a**: 65.0 mg, 61%; **10b**: 45.7 mg, 30%), as a colorless oil. The characterization data is in accordance with those reported in the literature.[9]

10a:

^{1}H NMR (300 MHz, $CDCl_3$) δ 8.61 (ddd, *J* = 4.9, 1.9, 1.0 Hz, 1H), 7.65 (td, *J* = 7.7, 1.9 Hz, 1H), 7.37 – 7.09 (m, 6H), 2.70 – 2.55 (m, 2H), 1.44 – 1.29 (m, 2H), 1.25 – 1.00 (m, 10H), 0.78 (t, *J* = 6.9 Hz, 3H).

^{13}C NMR (75 MHz, $CDCl_3$) δ 160.4 (C_q), 149.1 (CH), 140.8 (C_q), 140.4 (C_q), 136.1 (CH), 129.7 (CH), 129.7 (CH), 128.3 (CH), 125.7 (CH), 124.1 (CH), 121.6 (CH), 33.0 (CH_2), 31.9 (CH_2), 31.3 (CH_2), 29.5 (CH_2), 29.3 (CH_2), 29.2 (CH_2), 22.7 (CH_2), 14.1 (CH_3).

IR (ATR): $\tilde{\nu}$ = 3059, 2926, 2856, 1586, 1562, 1468, 1377, 795, 751 cm^{-1}.

HR-MS (ESI): m/z calcd for $C_{19}H_{25}N_1$ $[M+H]^+$: 268.2060, found: 268.2070.

10b:

^{1}H NMR (300 MHz, $CDCl_3$) δ 8.68 – 8.58 (m, 1H), 7.66 (td, *J* = 7.7, 1.8 Hz, 1H), 7.24 – 7.13 (m, 3H), 7.04 (d, *J* = 7.6 Hz, 2H), 2.29 – 2.12 (m, 4H), 1.31 (q, *J* = 7.7 Hz, 4H), 1.24 – 0.98 (m, 20H), 0.78 (t, *J* = 6.9 Hz, 6H).

^{13}C NMR (75 MHz, $CDCl_3$) δ 159.7 (C_q), 149.3 (CH), 140.8 (C_q), 139.9 (C_q), 135.7 (CH), 127.9 (CH), 126.5 (CH), 125.0 (CH), 121.6 (CH), 33.6 (CH_2), 31.9 (CH_2), 31.1 (CH_2), 29.5 (CH_2), 29.2 (CH_2), 29.1 (CH_2), 22.7 (CH_2), 14.1 (CH_3).

IR (ATR): $\tilde{\nu}$ = 2922, 2853, 1603, 1584, 1458, 1423, 1375, 1153, 749 cm^{-1}.

HR-MS (ESI): m/z calcd for $C_{27}H_{41}N_1$ $[M+H]^+$: 380.3312, found: 380.3325.

The general procedure **A** was followed using 2-phenylpyridine (**8**) (62.1 mg, 0.4 mmol), *n*-octyl bromide (**9**) (207.3 µL, 1.20 mmol), and $[Ru(t\text{-}BuCN)_5(H_2O)](BF_4)_2$ (**7**) (28.3 mg, 10 mol%) in NMP (2.0 mL). The reaction was stirred at 45 °C for 18 h under N_2 atmosphere. Crude ^{1}H NMR using mesitylene (0.40 mmol) as internal standard gave product **10a** (68%), and **10b** (21%).

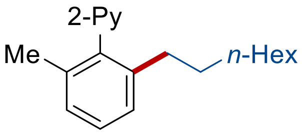

2-(2-methyl-6-octylphenyl)pyridine (29)

The general procedure **A** was followed using 2-(*o*-tolyl)pyridine (**27a**) (67.7 mg, 0.4 mmol), *n*-octyl bromide (**9**) (207.3 µL, 1.20 mmol), and [Ru(OAc)$_2$(*p*-cymene)] (**3**) (14.1 mg, 10 mol%) in NMP (2.0 mL). The reaction was stirred at 37 °C for 20 h under N_2 atmosphere. Purification by column chromatography (*n*-hexane/EtOAc = 20:1) yielded **29** (100.2 mg, 89%), as a colorless oil.

^{1}H NMR (400 MHz, $CDCl_3$) δ 8.75 – 8.69 (m, 1H), 7.74 (td, *J* = 7.7, 1.9 Hz, 1H), 7.27 – 7.19 (m, 3H), 7.15 – 7.07 (m, 2H), 2.33 (t, *J* = 8.0 Hz, 2H), 2.03 (s, 3H), 1.48 – 1.32 (m, 2H), 1.31 – 1.07 (m, 10H), 0.86 (t, *J* = 7.1 Hz, 3H).

^{13}C NMR (101 MHz, $CDCl_3$) δ 159.9 (C_q), 149.6 (CH), 140.8 (C_q), 140.2 (C_q), 136.1 (CH), 135.9 (C_q), 128.0 (CH), 127.5 (CH), 126.7 (CH), 124.8 (CH), 121.7 (CH), 33.5 (CH_2), 31.9 (CH_2), 31.2 (CH_2), 29.5 (CH_2), 29.3 (CH_2), 29.2 (CH_2), 22.7 (CH_2), 20.4 (CH_3), 14.2 (CH_3).

IR (ATR): ṽ = 2953, 2923, 2853, 1583, 1562, 1460, 1422, 1026, 749 cm^{-1}.

HR-MS (ESI): m/z calcd for $C_{20}H_{27}N_1$ $[M+H]^+$: 282.2216, found: 282.2224.

The general procedure **A** was followed using 2-(*o*-tolyl)pyridine (**27a**) (33.9 mg, 0.2 mmol) and *n*-octyl bromide (**9**) (103.7 µL, 0.6 mmol), and [Ru(*t*-BuCN)$_5$(H$_2$O)](BF$_4$)$_2$ (**7**) (14.0 mg, 10 mol%) in NMP (1.0 mL). The reaction was stirred at 37 °C for 20 h under N_2 atmosphere. Crude ^{1}H NMR using mesitylene (0.20 mmol) as internal standard gave product **29** (90%).

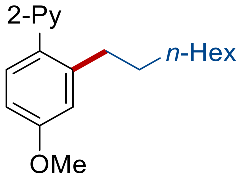

2-(4-methoxy-2-octylphenyl)pyridine (30a)

The general procedure **A** was followed using 2-(4-methoxyphenyl)pyridine (**27b**) (74.1 mg, 0.4 mmol), *n*-octyl bromide (**9**) (207.3 µL, 1.20 mmol), and [Ru(OAc)$_2$(*p*-cymene)] (**3**) (14.1 mg, 10 mol%) in NMP (2.0 mL). The reaction was stirred at 37 °C for 20 h under N_2 atmosphere. Purification by column chromatography (*n*-hexane/EtOAc = 10:1) yielded **30** (**30a**: 75.0 mg, 63%; **30b**: 39.4 mg, 24%), as a colorless oil.

30a:

^{1}H NMR (400 MHz, $CDCl_3$) δ 8.65 (ddd, *J* = 4.9, 1.9, 0.9 Hz, 1H), 7.69 (td, *J* = 7.7, 1.9 Hz, 1H), 7.34 (dt, *J* = 7.8, 1.1 Hz, 1H), 7.29 (d, *J* = 8.4 Hz, 1H), 7.19 (ddd, *J* = 7.6, 4.9, 1.2 Hz, 1H), 6.84 (d, *J* = 2.6 Hz, 1H), 6.80 (dd, *J* = 8.4, 2.7 Hz, 1H), 3.83 (s, 3H), 2.74 – 2.66 (m, 2H), 1.54 – 1.38 (m, 2H), 1.31 – 1.12 (m, 10H), 0.85 (t, *J* = 7.0 Hz, 3H).

^{13}C NMR (101 MHz, $CDCl_3$) δ 160.2 (C_q), 159.6 (C_q), 149.1 (CH), 142.6 (C_q), 136.1 (CH), 133.3 (C_q), 131.1 (CH), 124.2 (CH), 121.3 (CH), 115.3 (CH), 111.0 (CH), 55.3 (CH_3), 33.3 (CH_2), 31.9 (CH_2), 31.3 (CH_2), 29.5 (CH_2), 29.3 (CH_2), 29.2 (CH_2), 22.7 (CH_2), 14.2 (CH_3).

IR (ATR): ṽ = 2956, 2924, 2853, 1607, 1587, 1466, 1281, 1238, 787 cm^{-1}.

HR-MS (ESI): m/z calcd for $C_{20}H_{27}N_1O_1$ $[M+H]^+$: 298.2165, found: 298.2172.

30b:

^{1}H NMR (400 MHz, $CDCl_3$) δ 8.71 – 8.66 (m, 1H), 7.70 (td, *J* = 7.6, 1.9 Hz, 1H), 7.23 (ddd, *J* = 7.4, 3.7, 1.9 Hz, 2H), 6.66 (s, 2H), 3.82 (s, 3H), 2.28 (t, *J* = 8.0 Hz, 4H), 1.51 – 1.31 (m, 4H), 1.29 – 1.06 (m, 20H), 0.85 (t, *J* = 7.1 Hz, 6H).

^{13}C NMR (101 MHz, $CDCl_3$) δ 159.8 (C_q), 159.2 (C_q), 149.4 (CH), 142.5 (C_q), 135.8 (CH), 133.1 (C_q), 125.6 (CH), 121.5 (CH), 112.0 (CH), 55.3 (CH_3), 33.9 (CH_2), 32.0 (CH_2), 31.1 (CH_2), 29.6 (CH_2), 29.3 (CH_2), 29.2 (CH_2), 22.8 (CH_2), 14.2 (CH_3).

IR (ATR): ṽ = 2955, 2922, 2853, 1603, 1584, 1458, 1423, 1313, 1153, 749 cm^{-1}.

HR-MS (ESI): m/z calcd for $C_{28}H_{43}N_1O_1$ $[M+H]^+$: 410.3417, found: 410.3425.

The general procedure **A** was followed using 2-(4-methoxyphenyl)pyridine (**27b**) (74.1 mg, 0.4 mmol), *n*-octyl bromide (**9**) (207.3 µL, 1.20 mmol), and [Ru(*t*-BuCN)$_5$(H_2O)](BF_4)$_2$ (**7**) (28.3 mg, 10 mol%) in NMP (2.0 mL). The reaction was stirred at 37 °C for 20 h under N_2 atmosphere. Crude ^{1}H NMR using mesitylene (0.40 mmol) as internal standard gave product **30a** (78%) and **30b** (11%).

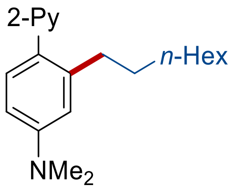

***N*,*N*-dimethyl-3-octyl-4-(pyridin-2-yl)aniline (31a)**

The general procedure **A** was followed using *N*,*N*-dimethyl-4-(pyridin-2-yl)aniline (**27c**) (79.3 mg, 0.4 mmol), *n*-octyl bromide (**9**) (207.3 µL, 1.20 mmol), and [Ru(*p*-cymene)OAc$_2$] (**3**) (14.1 mg, 10 mol%) in NMP (2.0 mL). The reaction was stirred at 45 °C for 20 h under N_2 atmosphere. Purification by column chromatography (*n*-hexane/EtOAc = 5:1) yielded **31** (**31a**: 54.3 mg, 44%; **31b**: traces).

^{1}H NMR (300 MHz, $CDCl_3$) δ 8.65 (ddd, *J* = 4.9, 1.9, 0.9 Hz, 1H), 7.68 (td, *J* = 7.7, 1.9 Hz, 1H), 7.40 – 7.23 (m, 2H), 7.16 (ddd, *J* = 7.5, 4.9, 1.2 Hz, 1H), 6.71 – 6.61 (m, 2H), 2.99 (s, 6H), 2.81 – 2.70 (m, 2H), 1.55 – 1.39 (m, 2H), 1.35 – 1.16 (m, 10H), 0.87 (t, *J* = 6.8 Hz, 3H).

^{13}C NMR (75 MHz, $CDCl_3$) δ 160.7 (C_q), 150.6 (C_q), 149.0 (CH), 141.8 (C_q), 135.8 (CH), 130.9 (CH), 129.1 (C_q), 124.0 (CH), 120.7 (CH), 113.7 (CH), 110.2 (CH), 40.6 (CH_3), 33.6 (CH_2), 31.9 (CH_2), 31.6 (CH_2), 29.6 (CH_2), 29.3 (CH_2), 29.2 (CH_2), 22.7 (CH_2), 14.1 (CH_3).

IR (ATR): ṽ = 2953, 2924, 2853, 1608, 1585, 1467, 1427, 1356, 785, 747 cm^{-1}.

HR-MS (ESI): m/z calcd for $C_{21}H_{30}N_2$ $[M+H]^+$: 311.2482, found: 311.2486.

The general procedure **A** was followed using *N*,*N*-dimethyl-4-(pyridin-2-yl)aniline (**27c**) (79.3 mg, 0.4 mmol), *n*-octyl bromide (**9**) (207.3 µL, 1.20 mmol), and $[Ru(t\text{-}BuCN)_5(H_2O)](BF_4)_2$ (**7**) (28.3 mg, 10 mol%) in NMP (2.0 mL). The reaction was stirred at 45 °C for 20 h under N_2 atmosphere. Crude ^{1}H NMR using mesitylene (0.40 mmol) as internal standard gave *mono*-alkylated product **31a** (66%) and *di*-alkylated product **31b** (3%).

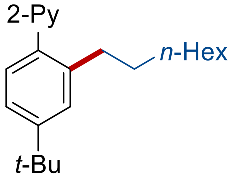

2-(4-(*tert*-butyl)-2-octylphenyl)pyridine (32a)

The general procedure **A** was followed using 2-(4-(*tert*-butyl)phenyl)pyridine (**27d**) (86.0 µL, 0.4 mmol) and *n*-octyl bromide (**9**) (207.3 µL, 1.20 mmol), and $[Ru(p\text{-cymene})OAc_2]$ (**3**) (14.1 mg, 10 mol%) in NMP (2.0 mL). The reaction was stirred at 45 °C for 20 h under N_2 atmosphere. Purification by column chromatography (*n*-hexane/EtOAc = 20:1) yielded **32** (**32a**: 82.1 mg, 63%; **32b**: 5% from crude ^{1}H NMR).

^{1}H NMR (300 MHz, $CDCl_3$) δ 8.68 (ddd, *J* = 4.9, 1.9, 1.0 Hz, 1H), 7.72 (td, *J* = 7.7, 1.9 Hz, 1H), 7.39 (dt, *J* = 7.8, 1.1 Hz, 1H), 7.34 – 7.28 (m, 3H), 7.22 (ddd, *J* = 7.5, 4.9, 1.2 Hz, 1H), 2.82 – 2.67 (m, 2H), 1.55 – 1.40 (m, 2H), 1.37 (s, 9H), 1.32 – 1.12 (m, 10H), 0.88 (t, *J* = 6.8 Hz, 3H).

^{13}C NMR (75 MHz, $CDCl_3$) δ 160.5 (C_q), 151.1 (C_q), 149.1 (CH), 140.3 (C_q), 137.5 (C_q), 136.0 (CH), 129.5 (CH), 126.8 (CH), 124.0 (CH), 122.8 (CH), 121.4 (CH), 34.6 (C_q), 33.3 (CH_2), 31.9 (CH_2), 31.5 (CH_2), 31.4 (CH_3), 29.5 (CH_2), 29.3 (CH_2), 29.2 (CH_2), 22.7 (CH_2), 14.2 (CH_3).

IR (ATR): ṽ = 2960, 2923, 2856, 1587, 1026, 828, 786, 747 cm^{-1}.

HR-MS (ESI): m/z calcd for $C_{23}H_{33}N_1$ $[M+H]^+$: 324.2686, found: 324.2689.

The general procedure **A** was followed using 2-(4-(*tert*-butyl)phenyl)pyridine (**27d**) (86.0 µL, 0.4 mmol) and *n*-octyl bromide (**9**) (207.3 µL, 1.20 mmol), and $[Ru(t\text{-}BuCN)_5(H_2O)](BF_4)_2$ (**7**) (28.3 mg, 10 mol%) in NMP

(2.0 mL). The reaction was stirred at 45 °C for 20 h under N_2 atmosphere. Crude ^{1}H NMR using mesitylene (0.40 mmol) as internal standard gave product **32a** (80%) and **32b** (10%).

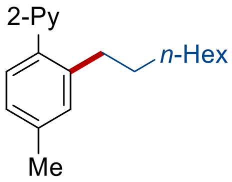

2-(4-methyl-2-octylphenyl)pyridine (33a)

The general procedure **A** was followed using 2-(*p*-tolyl)pyridine (**27e**) (67.7 mg, 0.4 mmol), *n*-octyl bromide (**9**) (207.3 µL, 1.20 mmol), and [Ru(*p*-cymene)OAc$_2$] (**3**) (14.1 mg, 10 mol%) in NMP (2.0 mL). The reaction was stirred at 45 °C for 18 h under N_2 atmosphere. Purification by column chromatography (*n*-hexane/EtOAc = 20:1) yielded **33** (**33a**: 93.4 mg, 83%; **33b**: 22.0 mg, 14%).

33a:

^{1}H NMR (300 MHz, CDCl$_3$) δ 8.58 (ddd, *J* = 4.9, 1.8, 0.9 Hz, 1H), 7.62 (td, *J* = 7.7, 1.9 Hz, 1H), 7.27 (dt, *J* = 7.8, 1.1 Hz, 1H), 7.20 – 7.07 (m, 2H), 7.02 (d, *J* = 1.7 Hz, 1H), 6.98 (dd, *J* = 7.7, 1.1 Hz, 1H), 2.67 – 2.50 (m, 2H), 2.29 (s, 3H), 1.43 – 1.28 (m, 2H), 1.23 – 1.02 (m, 10H), 0.77 (t, *J* = 6.9 Hz, 3H).

^{13}C NMR (75 MHz, CDCl$_3$) δ 160.4 (C$_q$), 149.1 (CH), 140.7 (C$_q$), 137.9 (C$_q$), 137.6 (C$_q$), 136.0 (CH), 130.5 (CH), 129.7 (CH), 126.5 (CH), 124.1 (CH), 121.4 (CH), 32.9 (CH$_2$), 31.9 (CH$_2$), 31.4 (CH$_2$), 29.5 (CH$_2$), 29.3 (CH$_2$), 29.2 (CH$_2$), 22.7 (CH$_2$), 21.3 (CH$_3$), 14.1 (CH$_3$).

IR (ATR): ṽ = 2955, 2922, 2853, 1614, 1586, 1467, 1426, 1026, 828, 786, 747 cm^{-1}.

HR-MS (ESI): m/z calcd for $C_{20}H_{27}N_1$ [M+H]$^+$: 282.2216, found: 282.2226.

33b:

^{1}H NMR (300 MHz, CDCl$_3$) δ 8.73 – 8.64 (m, 1H), 7.71 (td, *J* = 7.7, 1.9 Hz, 1H), 7.28 – 7.18 (m, 2H), 6.92 (s, 2H), 2.34 (s, 3H), 2.25 (t, *J* = 8.0 Hz, 4H), 1.44 – 1.33 (m, 4H), 1.28 – 1.05 (m, 20H), 0.85 (t, *J* = 6.9 Hz, 6H).

^{13}C NMR (75 MHz, CDCl$_3$) δ 159.9 (C$_q$), 149.3 (CH), 140.7 (C$_q$), 137.3 (C$_q$), 137.1 (C$_q$), 135.6 (CH), 127.3 (CH), 125.2 (CH), 121.4 (CH), 33.5 (CH$_2$), 31.9 (CH$_2$), 31.2 (CH$_2$), 29.6 (CH$_2$), 29.2 (CH$_2$), 29.1 (CH$_2$), 22.7 (CH$_2$), 21.3 (CH$_3$), 14.1 (CH$_3$).

IR (ATR): ṽ = 2954, 2922, 2854, 1610, 1586, 1458, 1425, 1377, 1025, 792, 749 cm^{-1}.

HR-MS (ESI): m/z calcd for $C_{28}H_{43}N_1$ [M+H]$^+$: 394.3468, found: 394.3480.

The general procedure **A** was followed using 2-(*p*-tolyl)pyridine (**27e**) (67.7 mg, 0.4 mmol) and *n*-octyl bromide (**9**) (207.3 µL, 1.20 mmol), and [Ru(*t*-BuCN)$_5$(H$_2$O)](BF$_4$)$_2$ (**7**) (28.3 mg, 10 mol%) in NMP (2.0 mL). The reaction was stirred at 45 °C for 20 h under N_2 atmosphere. Crude ^{1}H NMR using mesitylene (0.40 mmol) as internal standard gave product **33a** (80%) and **33b** (13%).

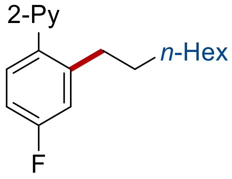

2-(4-fluoro-2-octylphenyl)pyridine (34a)

The general procedure **A** was followed using 2-(4-fluorophenyl)pyridine (**27f**) (69.3 mg, 0.4 mmol), *n*-octyl bromide (**9**) (207.3 µL, 1.20 mmol), and [Ru(*p*-cymene)OAc$_2$] (**3**) (7.1 mg, 5.0 mol%) in NMP (2.0 mL). The reaction was stirred at 45 °C for 10 h under N_2 atmosphere. Purification by column chromatography (*n*-hexane/EtOAc = 20:1) yielded **34** (**34a**: 28.5 mg, 25%; **34b**:106.3 mg, 67%), as a colorless oil.

34a:

^{1}H NMR (500 MHz, CDCl$_3$) δ 8.67 (ddd, *J* = 4.9, 1.9, 1.0 Hz, 1H), 7.73 (td, *J* = 7.7, 1.8 Hz, 1H), 7.34 (dt, *J* = 7.8, 1.1 Hz, 1H), 7.30 (dd, *J* = 8.5, 6.0 Hz, 1H), 7.24 (ddd, *J* = 7.6, 4.9, 1.2 Hz, 1H), 7.00 (dd, *J* = 10.1, 2.7 Hz, 1H), 6.94 (td, *J* = 8.4, 2.7 Hz, 1H), 2.72 – 2.64 (m, 2H), 1.50 – 1.40 (m, 2H), 1.29 – 1.13 (m, 10H), 0.85 (t, *J* = 7.1 Hz, 3H).

^{13}C NMR (126 MHz, CDCl$_3$) δ 162.8 (d, *J* = 246.7 Hz, C$_q$), 159.5 (C$_q$), 149.3 (CH), 143.61 (d, *J* = 7.5 Hz, C$_q$), 136.56 (d, *J* = 3.0 Hz, C$_q$), 136.3 (CH), 131.52 (d, *J* = 8.6 Hz, CH), 124.3 (CH), 121.8 (CH), 116.21 (d, *J* = 20.8 Hz, CH), 112.7 (d, *J* = 21.3 Hz, CH), 33.1 (d, *J* = 1.2 Hz, CH$_2$), 31.9 (CH$_2$), 31.0 (CH$_2$), 29.4 (CH$_2$), 29.3 (CH$_2$), 29.2 (CH$_2$), 22.8 (CH$_2$), 14.2 (CH$_3$).

^{19}F NMR (471 MHz, CDCl$_3$) δ -114.42 (td, *J* = 9.3, 6.2 Hz).

IR (ATR): ṽ = 2957, 2925, 2854, 1609, 1589, 1467, 1428, 1219, 787 cm^{-1}.

HR-MS (ESI): m/z calcd for $C_{19}H_{24}F_1N_1$ [M+H]$^+$: 286.1966, found: 286.1979.

34b:

^{1}H NMR (400 MHz, CDCl$_3$) δ 8.70 (ddd, *J* = 4.9, 1.9, 1.0 Hz, 1H), 7.73 (td, *J* = 7.6, 1.9 Hz, 1H), 7.28 – 7.24 (m, 1H), 7.22 (dt, *J* = 7.8, 1.1 Hz, 1H), 6.81 (d, *J* = 9.7 Hz, 2H), 2.26 (ddd, *J* = 8.6, 6.9, 4.1 Hz, 4H), 1.46 – 1.29 (m, 4H), 1.29 – 1.09 (m, 20H), 0.85 (t, *J* = 7.1 Hz, 6H).

^{13}C NMR (101 MHz, CDCl$_3$) δ 162.5 (d, *J* = 244.7 Hz, C$_q$), 159.1 (C$_q$), 149.6 (CH), 143.5 (d, *J* = 7.7 Hz, C$_q$), 136.0 (CH), 136.0 (C$_q$), 125.4 (CH), 121.9 (CH), 113.1 (d, *J* = 20.9 Hz, CH), 113.1 (d, *J* = 20.9 Hz, CH), 33.7 (d, *J* = 1.7 Hz, CH$_2$), 32.0 (CH$_2$), 30.8 (CH$_2$), 29.5 (CH$_2$), 29.3 (CH$_2$), 29.2 (CH$_2$), 22.8 (CH$_2$), 14.2 (CH$_3$).

^{19}F NMR (282 MHz, CDCl$_3$) δ -115.27 (t, *J* = 9.8 Hz).

IR (ATR): ṽ =2957, 2923, 2854, 1745, 1454, 1260, 946, 750 cm^{-1}.

HR-MS (ESI): m/z calcd for $C_{27}H_{40}F_1N_1$ [M+H]$^+$: 398.3218, found: 398.3224.

The general procedure **A** was followed using 2-(4-fluorophenyl)pyridine (**27f**) (69.3 mg, 0.4 mmol) and *n*-octyl bromide (**9**) (207.3 µL, 1.20 mmol), and [Ru(*t*-BuCN)$_5$(H$_2$O)](BF$_4$)$_2$ (**7**) (14.2 mg, 5.0 mol%) in NMP (2.0 mL). The reaction was stirred at 45 °C for 10 h under N_2 atmosphere. Crude ^{1}H NMR using mesitylene (0.40 mmol) as internal standard gave mono-alkylated product **34a** (32%) and di-alkylated product **34b** (63%).

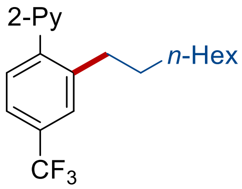

2-(2-octyl-4-(trifluoromethyl)phenyl)pyridine (35a)

The general procedure **A** was followed using 2-(4-(trifluoromethyl)phenyl)pyridine (**27g**) (89.3 mg, 0.4 mmol) and *n*-octyl bromide (**9**) (207.3 µL, 1.20 mmol), and [Ru(*p*-cymene)OAc$_2$] (**3**) (7.1 mg, 5.0 mol%) in NMP (2.0 mL). The reaction was stirred at 45 °C for 10 h under N_2 atmosphere. Purification by column chromatography (*n*-hexane/EtOAc = 20:1) yielded **35** (**35a**: 84.4 mg, 63%; **35b**: 53.4mg, 30%), as a colorless oil.

35a:

^{1}H NMR (300 MHz, CDCl$_3$) δ 8.70 (ddd, *J* = 4.9, 1.9, 1.0 Hz, 1H), 7.77 (td, *J* = 7.7, 1.8 Hz, 1H), 7.59 – 7.49 (m, 2H), 7.44 (dt, *J* = 8.0, 0.7 Hz, 1H), 7.37 (dt, *J* = 7.9, 1.1 Hz, 1H), 7.29 (ddd, *J* = 7.6, 4.9, 1.2 Hz, 1H), 2.79 – 2.68 (m, 2H), 1.54 – 1.38 (m, 2H), 1.34 – 1.11 (m, 10H), 0.86 (t, *J* = 6.9 Hz, 3H).

^{13}C NMR (75 MHz, CDCl$_3$) δ 159.0 (C$_q$), 149.4 (CH), 143.7 (q, *J* = 1.4 Hz, C$_q$), 141.9 (C$_q$), 136.3 (CH), 130.3 (q, *J* = 32.2 Hz, C$_q$), 130.2 (CH), 126.5 (q, *J* = 3.7 Hz, CH), 124.3 (q, *J* = 271.9 Hz, C$_q$), 124.0 (CH), 122.5 (q, *J* = 3.9 Hz, CH), 122.2 (CH), 32.9 (CH$_2$), 31.8 (CH$_2$), 31.0 (CH$_2$), 29.4 (CH$_2$), 29.2 (CH$_2$), 29.1 (CH$_2$), 22.6 (CH$_2$), 14.1 (CH$_3$).

^{19}F NMR (282 MHz, CDCl$_3$) δ -62.5.

IR (ATR): ṽ =2957, 2925, 2854, 1588, 1468, 1328, 1163, 1119, 1080, 837, 791, 748 cm^{-1}.

HR-MS (ESI): m/z calcd for $C_{20}H_{24}F_3N_1$ [M+H]$^+$: 336.1934, found: 336.1928.

35b:

^{1}H NMR (300 MHz, CDCl$_3$) δ 8.72 (ddd, *J* = 4.9, 1.8, 1.0 Hz, 1H), 7.77 (td, *J* = 7.7, 1.8 Hz, 1H), 7.36 (s, 2H), 7.30 (ddd, *J* = 7.6, 4.9, 1.2 Hz, 1H), 7.23 (dt, *J* = 7.7, 1.1 Hz, 1H), 2.42 – 2.23 (m, 4H), 1.40 (td, *J* = 8.8, 4.5 Hz, 4H), 1.24 – 1.04 (m, 20H), 0.85 (t, *J* = 6.9 Hz, 6H).

^{13}C NMR (75 MHz, CDCl$_3$) δ 158.4 (C$_q$), 149.6 (CH), 143.2 (q, *J* = 1.2 Hz, C$_q$), 141.9 (C$_q$), 136.0 (CH), 130.1 (d, *J* = 31.9 Hz, C$_q$), 124.6 (CH), 124.4 (d, *J* = 272.2 Hz, C$_q$), 123.2 (q, *J* = 3.7 Hz, CH), 122.1 (CH), 33.5 (CH$_2$), 31.8 (CH$_2$), 30.8 (CH$_2$), 29.4 (CH$_2$), 29.1 (CH$_2$), 29.1 (CH$_2$), 22.6 (CH$_2$), 14.1 (CH$_3$).

^{19}F NMR (282 MHz, CDCl$_3$) δ -62.5.

IR (ATR): ṽ = 2957, 2925, 2854, 1741, 1586, 1467, 1358, 1218, 1159, 1122, 886, 750 cm^{-1}.

HR-MS (ESI): m/z calcd for $C_{28}H_{40}F_3N_1$ $[M+H]^+$: 448.3186, found: 448.3175.

The general procedure **A** was followed using 2-(4-(trifluoromethyl)phenyl)pyridine (**27g**) (89.3 mg, 0.4 mmol) and *n*-octyl bromide (**9**) (207.3 µL, 1.20 mmol), and $[Ru(t\text{-}BuCN)_5(H_2O)](BF_4)_2$ (**7**) (14.2 mg, 5.0 mol%) in NMP (2.0 mL). The reaction was stirred at 45 °C for 10 h under N_2 atmosphere. Crude ^{1}H NMR using mesitylene (0.40 mmol) as internal standard gave mono-alkylated product **35a** (45%) and di-alkylated product **35b** (46%).

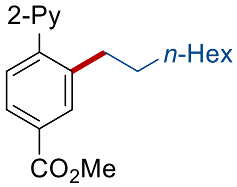

methyl 3-octyl-4-(pyridin-2-yl)benzoate (36a)

The general procedure **A** was followed using methyl 4-(pyridin-2-yl)benzoate (**27h**) (85.3 mg, 0.4 mmol), *n*-octyl bromide (**9**) (207.3 µL, 1.20 mmol), and $[Ru(p\text{-cymene})OAc_2]$ (**3**) (14.1 mg, 10 mol%) in NMP (2.0 mL). The reaction was stirred at 50 °C for 24 h under N_2 atmosphere. Purification by column chromatography (*n*-hexane/EtOAc = 10:1) yielded **36** (**36a**: 23.5 mg, 18% and **36b**: 28.0 mg, 16%), as a colorless oil.

36a:

^{1}H NMR (500 MHz, $CDCl_3$) δ 8.69 (ddd, *J* = 4.9, 1.8, 1.0 Hz, 1H), 7.98 (dd, *J* = 1.8, 0.5 Hz, 1H), 7.91 (dd, *J* = 8.0, 1.8 Hz, 1H), 7.76 (td, *J* = 7.7, 1.8 Hz, 1H), 7.42 – 7.36 (m, 2H), 7.28 (ddd, *J* = 7.6, 4.9, 1.2 Hz, 1H), 3.93 (s, 3H), 2.77 – 2.67 (m, 2H), 1.50 – 1.40 (m, 2H), 1.32 – 1.14 (m, 10H), 0.85 (t, *J* = 7.1 Hz, 3H).

^{13}C NMR (126 MHz, $CDCl_3$) δ 167.2 (C_q), 159.4 (C_q), 149.4 (CH), 144.8 (C_q), 141.4 (C_q), 136.4 (CH), 131.1 (CH), 130.0 (CH), 129.9 (C_q), 127.0 (CH), 124.1 (CH), 122.3 (CH), 52.2 (CH_3), 33.0 (CH_2), 31.9 (CH_2), 31.2 (CH_2), 29.5 (CH_2), 29.3 (CH_2), 29.2 (CH_2), 22.8 (CH_2), 14.2 (CH_3).

IR (ATR): ṽ =2954, 2924, 2854, 1720, 1585, 1467, 1428, 1289, 1247, 1195, 1110, 764 cm^{-1}.

HR-MS (ESI): m/z calcd for $C_{21}H_{27}N_1O_2$ $[M+H]^+$: 326.2115, found: 326.2122.

36b:

^{1}H NMR (400 MHz, $CDCl_3$) δ 8.71 (ddd, *J* = 4.9, 1.8, 1.0 Hz, 1H), 7.79 (s, 2H), 7.75 (td, *J* = 7.7, 1.8 Hz, 1H), 7.28 (ddd, *J* = 7.6, 4.9, 1.2 Hz, 1H), 7.22 (dt, *J* = 7.8, 1.1 Hz, 1H), 3.92 (s, 3H), 2.31 (td, *J* = 8.1, 2.4 Hz, 4H), 1.49 – 1.30 (m, 4H), 1.28 – 1.06 (m, 20H), 0.85 (t, *J* = 7.1 Hz, 6H).

^{13}C NMR (101 MHz, $CDCl_3$) δ 167.5 (C_q), 158.9 (C_q), 149.6 (CH), 144.4 (C_q), 141.5 (C_q), 136.0 (CH), 129.6 (C_q), 127.8 (CH), 124.8 (CH), 122.1 (CH), 52.2 (CH_3), 33.6 (CH_2), 31.9 (CH_2), 31.1 (CH_2), 29.6 (CH_2), 29.3 (CH_2), 29.2 (CH_2), 22.8 (CH_2), 14.2 (CH_3).

IR (ATR): ṽ = 2922, 2853, 1721, 1585, 1435, 1209, 1125, 770, 750 cm^{-1}.

HR-MS (ESI): m/z calcd for $C_{29}H_{43}N_1O_2$ $[M+H]^+$: 438.3367, found: 438.3377.

The general procedure **A** was followed using methyl 4-(pyridin-2-yl)benzoate (**27h**) (85.3 mg, 0.4 mmol) and *n*-octyl bromide (**9**) (207.3 µL, 1.20 mmol), and $[Ru(t\text{-}BuCN)_5(H_2O)](BF_4)_2$ (**7**) (28.3 mg, 10 mol%) in NMP (2.0 mL). The reaction was stirred at 50 °C for 24 h under N_2 atmosphere. Crude ^1H NMR using mesitylene (0.40 mmol) as internal standard gave mono-alkylated product **31a** (0%) and di-alkylated product **31b** (88%).

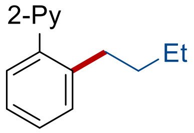

2-(2-butylphenyl)pyridine (37a)

The general procedure **A** was followed using 2-phenylpyridine (**8**) (62.1 mg, 0.4 mmol) and *n*-butyl bromide (**28b**) (129.7 µL, 1.20 mmol), and $[Ru(p\text{-}cymene)OAc_2]$ (**3**) (14.1 mg, 10 mol%) in NMP (2.0 mL). The reaction was stirred at 37 °C for 20 h under N_2 atmosphere. Purification by column chromatography (*n*-hexane/EtOAc = 20:1) yielded **37** (**37a**: 51.5 mg, 61%; **37b**: 16.0 mg, 15%), as a colorless oil. The characterization data is in accordance with those reported in the literature.[14]

37a:

^{1}H NMR (600 MHz, $CDCl_3$) δ 8.72 – 8.67 (m, 1H), 7.74 (td, *J* = 7.7, 1.7 Hz, 1H), 7.38 (dt, *J* = 7.8, 1.0 Hz, 1H), 7.36 – 7.29 (m, 3H), 7.28 – 7.22 (m, 2H), 2.75 – 2.67 (m, 2H), 1.49 – 1.38 (m, 2H), 1.22 (h, *J* = 7.4 Hz, 2H), 0.79 (t, *J* = 7.4 Hz, 3H).

^{13}C NMR (151 MHz, $CDCl_3$) δ 160.4 (C_q), 149.2 (CH), 140.8 (C_q), 140.4 (C_q), 136.2 (CH), 129.8 (CH), 129.8 (CH), 128.4 (CH), 125.8 (CH), 124.2 (CH), 121.7 (CH), 33.6 (CH_2), 32.7 (CH_2), 22.6 (CH_2), 13.9 (CH_3).

IR (ATR): ṽ =2956, 2929, 2857, 1737, 1586, 1468, 1425, 1234, 749 cm^{-1}.

HR-MS (ESI): m/z calcd for $C_{15}H_{17}N_1$ $[M+H]^+$: 212.1434, found: 212.1438.

37b:

^{1}H NMR (500 MHz, $CDCl_3$) δ 8.71 – 8.65 (m, 1H), 7.71 (td, *J* = 7.7, 1.8 Hz, 1H), 7.27 – 7.20 (m, 3H), 7.09 (d, *J* = 7.6 Hz, 2H), 2.28 (td, *J* = 8.0, 2.9 Hz, 4H), 1.44 – 1.28 (m, 4H), 1.13 (h, *J* = 7.4 Hz, 4H), 0.72 (t, *J* = 7.3 Hz, 6H).

^{13}C NMR (126 MHz, $CDCl_3$) δ 159.8 (C_q), 149.4 (CH), 140.8 (C_q), 140.0 (C_q), 135.9 (CH), 128.0 (CH), 126.6 (CH), 125.2 (CH), 121.7 (CH), 33.4 (CH_2), 33.3 (CH_2), 22.7 (CH_2), 13.9 (CH_3).

IR (ATR): ṽ =2956, 2929, 2859, 1584, 1462, 1423, 1024, 789, 751 cm^{-1}.

HR-MS (ESI): m/z calcd for $C_{19}H_{25}N_1$ $[M+H]^+$: 268.2060, found: 268.2066.

The general procedure **A** was followed using 2-phenylpyridine (**8**) (31.1 mg, 0.2 mmol) and *n*-butyl bromide (**28b**) (64.9 µL, 0.6 mmol), and [Ru(*t*-BuCN)$_5$(H$_2$O)](BF$_4$)$_2$ (**7**) (14.0 mg, 10 mol%) in NMP (1.0 mL). The reaction was stirred at 37 °C for 20 h under N_2 atmosphere. Crude ^{1}H NMR using mesitylene (0.20 mmol) as internal standard gave product **37a** (66%) and **37b** (26%).

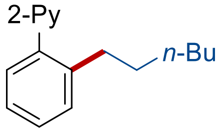

2-(2-hexylphenyl)pyridine (38a)

The general procedure **A** was followed using 2-phenylpyridine (**8**) (62.1 mg, 0.4 mmol), *n*-hexyl bromide (**28c**) (168.4 µL, 1.20 mmol), and [Ru(*p*-cymene)OAc$_2$] (**3**) (14.1 mg, 10 mol%) in NMP (2.0 mL). The reaction was stirred at 37 °C for 20 h under N_2 atmosphere. Purification by column chromatography (*n*-hexane/EtOAc = 20:1) yielded **38** (**38a**: 64.1 mg, 67%; **38b**: 30% from crude ^{1}H NMR), as a colorless oil. The characterization data is in accordance with those reported in the literature.[15]

38a:

^{1}H NMR (300 MHz, CDCl$_3$) δ 8.59 (ddd, *J* = 4.9, 1.9, 1.0 Hz, 1H), 7.62 (td, *J* = 7.7, 1.8 Hz, 1H), 7.33 – 7.08 (m, 6H), 2.67 – 2.55 (m, 2H), 1.36 (dtd, *J* = 9.9, 7.2, 5.6 Hz, 2H), 1.21 – 0.96 (m, 6H), 0.81 – 0.66 (m, 3H).

^{13}C NMR (75 MHz, CDCl$_3$) δ 160.4 (C$_q$), 149.2 (CH), 140.9 (C$_q$), 140.4 (C$_q$), 136.1 (CH), 129.8 (CH), 129.8 (CH), 128.3 (CH), 125.8 (CH), 124.2 (CH), 121.6 (CH), 33.0 (CH$_2$), 31.6 (CH$_2$), 31.3 (CH$_2$), 29.2 (CH$_2$), 22.6 (CH$_2$), 14.1 (CH$_3$).

IR (ATR): ṽ =2956, 2926, 2854, 1585, 1469, 1424, 1024, 794, 746 cm^{-1}.

HR-MS (ESI): m/z calcd for $C_{17}H_{21}N_1$ [M+H]$^+$: 240.1747, found: 240.1752.

The general procedure **A** was followed using 2-phenylpyridine (**8**) (31.1 mg, 0.2 mmol) and *n*-hexyl bromide (**28c**) (84.2 µL, 0.6 mmol), and [Ru(*t*-BuCN)$_5$(H$_2$O)](BF$_4$)$_2$ (**7**) (14.0 mg, 10 mol%) in NMP (1.0 mL). The reaction was stirred at 37 °C for 20 h under N_2 atmosphere. Crude ^{1}H NMR using mesitylene (0.20 mmol) as internal standard gave *mono*-alkylated product **38a** (73%) and *di*-alkylated product **38b** (22%).

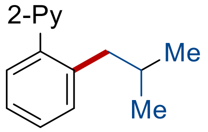

2-(2-isobutylphenyl)pyridine (39a)

The general procedure **A** was followed using 2-phenylpyridine (**8**) (62.1 mg, 0.4 mmol), 1-bromo-2-methylpropane (**28d**) (130.5 µL, 1.20 mmol), and [Ru(*p*-cymene)OAc$_2$] (**3**) (14.1 mg, 10 mol%) in NMP (2.0

mL). The reaction was stirred at 37 °C for 20 h under N_2 atmosphere. Purification by column chromatography (*n*-hexane/EtOAc = 20:1) yielded **39** (**39a**: 49.0 mg, 58%; **39b**: 26.7 mg, 25%), as a colorless oil. The characterization data is in accordance with those reported in the literature.[16]

39a:

^{1}H NMR (400 MHz, $CDCl_3$) δ 8.73 (ddd, *J* = 4.9, 1.9, 1.0 Hz, 1H), 7.77 (td, *J* = 7.7, 1.8 Hz, 1H), 7.41 (dt, *J* = 7.8, 1.1 Hz, 1H), 7.40 – 7.34 (m, 2H), 7.33 – 7.26 (m, 3H), 2.69 (d, *J* = 7.2 Hz, 2H), 1.66 (dp, *J* = 13.6, 6.8 Hz, 1H), 0.77 (d, *J* = 6.6 Hz, 6H).

^{13}C NMR (101 MHz, $CDCl_3$) δ 160.7 (C_q), 149.2 (CH), 140.8 (C_q), 139.7 (C_q), 136.1 (CH), 130.6 (CH), 129.9 (CH), 128.1 (CH), 125.9 (CH), 124.4 (CH), 121.6 (CH), 42.1 (CH_2), 29.9 (CH), 22.5 (CH_3).

IR (ATR): ṽ =2953, 2867, 1586, 1562, 1468, 1425, 751 cm^{-1}.

HR-MS (ESI): m/z calcd for $C_{15}H_{17}N_1$ $[M+H]^+$: 212.1434, found: 212.1442.

39b:

^{1}H NMR (400 MHz, $CDCl_3$) δ 8.70 (ddd, *J* = 4.9, 1.7, 0.9 Hz, 1H), 7.72 – 7.72 (m, 1H), 7.23 (ddt, *J* = 8.9, 6.4, 1.7 Hz, 3H), 7.08 (d, *J* = 7.6 Hz, 2H), 2.28 – 2.14 (m, 4H), 1.60 (dp, *J* = 13.5, 6.8 Hz, 2H), 0.71 (dd, *J* = 6.6, 3.8 Hz, 12H).

^{13}C NMR (101 MHz, $CDCl_3$) δ 159.9 (C_q), 149.3 (CH), 140.7 (C_q), 139.6 (C_q), 135.7 (CH), 127.7 (CH), 127.4 (CH), 125.6 (CH), 121.6 (CH), 42.9 (CH_2), 29.5(CH), 22.8 (CH_3), 22.5 (CH_3).

IR (ATR): ṽ =2953, 2927, 2867, 1583, 1465, 1424, 1365, 789, 772, 751 cm^{-1}.

HR-MS (ESI): m/z calcd for $C_{19}H_{25}N_1$ $[M+H]^+$: 268.2060, found: 268.2064.

The general procedure **A** was followed using 2-phenylpyridine (**8**) (31.1 mg, 0.2 mmol) and 1-bromo-2-methylpropane (**28d**) (65.3 µL, 0.6 mmol), and $[Ru(t\text{-BuCN})_5(H_2O)](BF_4)_2$ (**7**) (14.0 mg, 10 mol%) in NMP (1.0 mL). The reaction was stirred at 37 °C for 20 h under N_2 atmosphere. Crude ^{1}H NMR using mesitylene (0.20 mmol) as internal standard gave *mono*-alkylated product **39a** (60%) and *di*-alkylated product **39b** (8%).

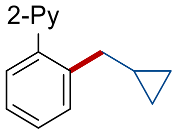

2-(2-(cyclopropylmethyl)phenyl)pyridine (40a)

The general procedure **A** was followed using 2-phenylpyridine (**8**) (62.1 mg, 0.4 mmol), (bromomethyl)cyclopropane (**28e**) (116.4 µL, 1.20 mmol), and [Ru(*p*-cymene)OAc_2] (**3**) (14.1 mg, 10 mol%) in NMP (2.0 mL). The reaction was stirred at 37 °C for 20 h under N_2 atmosphere. Purification by column

chromatography (n-hexane/EtOAc = 20:1) yielded **40** (**40a**: 26.8 mg, 32%; **40b**: traces), as a colorless oil. The characterization data is in accordance with those reported in the literature.[13]

^{1}H NMR (300 MHz, $CDCl_3$) δ 8.64 (ddd, J = 5.0, 1.9, 0.9 Hz, 1H), 7.70 (td, J = 7.7, 1.8 Hz, 1H), 7.48 – 7.41 (m, 1H), 7.38 – 7.30 (m, 2H), 7.30 – 7.16 (m, 3H), 2.60 (d, J = 6.8 Hz, 2H), 0.90 – 0.72 (m, 1H), 0.43 – 0.30 (m, 2H), 0.04 – -0.07 (m, 2H).

^{13}C NMR (75 MHz, $CDCl_3$) δ 160.5 (C_q), 149.2 (CH), 140.4 (C_q), 140.2 (C_q), 136.3 (CH), 129.8 (CH), 129.5 (CH), 128.4 (CH), 126.1 (CH), 124.4 (CH), 121.8 (CH), 37.5 (CH_2), 11.7 (CH), 4.9 (CH_2), 4.9 (CH_2).

IR (ATR): ṽ =3062, 3003, 2921, 1586, 1562, 1469, 1425, 1024, 750 cm^{-1}.

HR-MS (ESI): m/z calcd for $C_{15}H_{15}N_1$ $[M+H]^+$: 210.1277, found: 210.1280.

The general procedure **A** was followed using 2-phenylpyridine (**8**) (31.1 mg, 0.2 mmol) and (bromomethyl)cyclopropane (**28e**) (58.2 µL, 0.6 mmol), and $[Ru(t\text{-}BuCN)_5(H_2O)](BF_4)_2$ (**7**) (14.2 mg, 10 mol%) in NMP (1.0 mL). The reaction was stirred at 37 °C for 20 h under N_2 atmosphere. Crude ^{1}H NMR using mesitylene (0.20 mmol) as internal standard gave *mono*-alkylated product **40a** (26%) and *di*-alkylated product **40b** (traces).

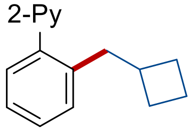

2-(2-(cyclobutylmethyl)phenyl)pyridine (41a)

The general procedure **A** was followed using 2-phenylpyridine (**8**) (62.1 mg, 0.4 mmol) and (bromomethyl)cyclobutane (**28f**) (134.9 µL, 1.20 mmol), and $[Ru(p\text{-cymene})OAc_2]$ (**3**) (14.1 mg, 10 mol%) in NMP (2.0 mL). The reaction was stirred at 37 °C for 20 h under N_2 atmosphere. Purification by column chromatography (n-hexane/EtOAc = 20:1) yielded **41** (**41a**: 39.9 mg, 44%; **41b**: 46.6 mg, 40%), as a colorless oil.

41a:

^{1}H NMR (400 MHz, $CDCl_3$) δ 8.67 (ddd, J = 4.9, 1.8, 1.0 Hz, 1H), 7.72 (td, J = 7.7, 1.9 Hz, 1H), 7.35 (dt, J = 7.8, 1.1 Hz, 1H), 7.30 (td, J = 7.7, 1.9 Hz, 2H), 7.26 – 7.20 (m, 3H), 2.81 (d, J = 7.5 Hz, 2H), 2.39 (hept, J = 7.8 Hz, 1H), 1.94 – 1.81 (m, 2H), 1.77 – 1.63 (m, 2H), 1.59 – 1.47 (m, 2H).

^{13}C NMR (101 MHz, $CDCl_3$) δ 160.6 (C_q), 149.2 (CH), 140.6 (C_q), 139.2 (C_q), 136.2 (CH), 129.9 (CH), 129.7 (CH), 128.2 (CH), 125.9 (CH), 124.4 (CH), 121.7 (CH), 39.8 (CH_2), 37.0 (CH), 28.5 (CH_2), 28.5 (CH_2), 18.4 (CH_2).

IR (ATR): ṽ = 2971, 2929, 2854,1586, 1469, 1441, 1424, 1240, 1023, 746 cm^{-1}.

HR-MS (ESI): m/z calcd for $C_{16}H_{17}N_1$ $[M+H]^+$: 224.1434, found: 224.1438.

41b:

^{1}**H NMR** (400 MHz, $CDCl_3$) δ 8.70 (ddd, *J* = 4.9, 1.8, 0.9 Hz, 1H), 7.71 (td, *J* = 7.7, 1.8 Hz, 1H), 7.26 – 7.23 (m, 1H), 7.22 – 7.17 (m, 2H), 7.05 (d, *J* = 7.6 Hz, 2H), 2.46 – 2.29 (m, 6H), 1.94 – 1.83 (m, 4H), 1.76 – 1.61 (m, 4H), 1.58 – 1.44 (m, 4H).

^{13}**C NMR** (101 MHz, $CDCl_3$) δ 159.8 (C_q), 149.4 (CH), 140.1 (C_q), 139.0 (C_q), 135.9 (CH), 127.7 (CH), 126.4 (CH), 126.4 (CH), 125.2 (CH), 121.7 (CH), 40.4 (CH_2), 36.5 (CH), 28.6 (CH_2), 28.6 (CH_2), 18.4 (CH_2).

IR (ATR): ṽ =2971, 2955, 2928, 2853, 1582, 1562, 1448, 1424, 1024, 789, 750 cm^{-1}.

HR-MS (ESI): m/z calcd for $C_{21}H_{25}N_1$ $[M+H]^+$: 292.2060, found: 292.2068.

The general procedure **A** was followed using 2-phenylpyridine (**8**) (31.1 mg, 0.2 mmol) and (bromomethyl)cyclobutane (**28f**) (67.5 µL, 0.6 mmol), and [Ru(*t*-BuCN)$_5$(H_2O)](BF_4)$_2$ (**7**) (14.0 mg, 10 mol%) in NMP (1.0 mL). The reaction was stirred at 37 °C for 20 h under N_2 atmosphere. Crude ^{1}H NMR using mesitylene (0.20 mmol) as internal standard gave *mono*-alkylated product **41a** (68%) and *di*-alkylated product **41b** (11%).

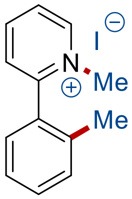

1-methyl-2-(*o*-tolyl)pyridin-1-ium iodide (42)

The general procedure **A** was followed using 2-phenylpyridine (**8**) (62.1 mg, 0.4 mmol), iodomethane (**28g**) (100.0 µL, 1.50 mmol), and [Ru(*p*-cymene)OAc$_2$] (**3**) (14.1 mg, 10 mol%) in NMP (2.0 mL). The reaction was stirred at 45 °C for 20 h under N_2 atmosphere. Purification by column chromatography (DCM/MeOH = 10:1) yielded **42** (123.6 mg, 96 %, **42a**: **42b**= 1 : 1). The characterization data is in accordance with those reported in the literature.[17]

^{1}**H NMR** (300 MHz, $CDCl_3$) δ 9.97 (d, *J* = 6.1 Hz, 1H), 9.77 (d, *J* = 6.1 Hz, 1H), 8.70 (td, *J* = 7.9, 1.5 Hz, 1H), 8.63 (td, *J* = 7.8, 1.4 Hz, 1H), 8.36 – 8.20 (m, 2H), 7.88 – 7.76 (m, 2H), 7.55 – 7.46 (m, 2H), 7.44 – 7.36 (m, 3H), 7.25 – 7.20 (m, 2H), 4.26 (s, 3H), 4.20 (s, 3H), 2.14 (s, $3H^{mono}$), 2.04 (s, $6H^{di}$).

^{13}**C NMR** (75 MHz, $CDCl_3$) δ 155.5 (C_q), 155.5 (C_q), 148.6 (CH), 148.0 (CH), 146.3 (CH), 145.7 (CH), 135.7 (C_q), 135.6 (C_q), 131.7 (CH), 131.6 (CH), 131.3 (CH), 130.7 (C_q), 130.4 (C_q), 129.6 (CH), 129.4 (CH), 129.0 (CH), 128.8 (CH), 127.8 (CH), 127.7 (CH), 127.2 (CH), 47.5 (CH_3), 46.7 (CH_3), 20.5 (CH_3), 20.1 (CH_3).

IR (ATR): ṽ = 3454, 2921, 2853, 1625, 1459, 1377, 1266, 768 cm^{-1}.

HR-MS (ESI): m/z calcd for $C_{13}H_{14}N$ $[M]^+$: 184.1121, found: 184.1127.

HR-MS (ESI): m/z calcd for $C_{14}H_{16}N$ $[M]^+$: 198.1277, found: 198.1282.

The general procedure **A** was followed using 2-phenylpyridine (**8**) (62.1 mg, 0.4 mmol) and iodomethane (**28g**) (100.0 µL, 1.50 mmol), and [Ru(*t*-BuCN)$_5$(H$_2$O)](BF$_4$)$_2$ (**7**) (28.3 mg, 10 mol%) in NMP (2.0 mL). The reaction was stirred at 45 °C for 20 h under N_2 atmosphere. Crude ^{1}H NMR using mesitylene (0.40 mmol) as internal standard gave *mono*-alkylated product **42a** (42%) and *di*-alkylated product **42b** (43%).

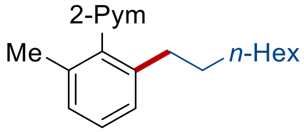

2-(2-methyl-6-octylphenyl)pyrimidine (43)

The general procedure **A** was followed using 2-(*o*-tolyl)pyrimidine (**27j**) (68.1 mg, 0.4 mmol), *n*-octyl bromide (**9**) (207.3 µL, 1.20 mmol), and [Ru(*p*-cymene)OAc$_2$] (**3**) (14.1 mg, 10 mol%) in NMP (2.0 mL). The reaction was stirred at 28 °C for 20 h under N_2 atmosphere. Purification by column chromatography (*n*-hexane/EtOAc = 20:1) yielded **43** (93.8 mg, 83%), as a colorless oil.

^{1}H NMR (400 MHz, CDCl$_3$) δ 8.84 (d, *J* = 4.9 Hz, 2H), 7.25 – 7.20 (m, 2H), 7.10 (dddd, *J* = 8.8, 7.4, 1.3, 0.7 Hz, 2H), 2.40 – 2.30 (m, 2H), 2.05 (s, 3H), 1.46 – 1.34 (m, 2H), 1.29 – 1.06 (m, 10H), 0.84 (t, *J* = 7.1 Hz, 3H).

^{13}C NMR (101 MHz, CDCl$_3$) δ 168.6 (C$_q$), 157.0 (CH), 157.0 (CH), 140.3 (C$_q$), 138.8 (C$_q$), 135.4 (C$_q$), 128.5 (CH), 127.7 (CH), 126.8 (CH), 118.9 (CH), 33.5 (CH$_2$), 31.9 (CH$_2$), 31.0 (CH$_2$), 29.5 (CH$_2$), 29.3 (CH$_2$), 29.1 (CH$_2$), 22.7 (CH$_2$), 19.9 (CH$_3$), 14.2 (CH$_3$).

IR (ATR): ṽ = 2956, 2923, 2853, 1567, 1555, 819, 778, 755 cm^{-1}.

HR-MS (ESI): m/z calcd for $C_{19}H_{26}N_2$ [M+H]$^+$: 283.2169, found: 283.2175.

The general procedure **A** was followed using 2-(*o*-tolyl)pyrimidine (**27j**) (34.1 mg, 0.2 mmol) and *n*-octyl bromide (**9**) (103.7 µL, 0.6 mmol), and [Ru(*t*-BuCN)$_5$(H$_2$O)](BF$_4$)$_2$ (**7**) (14.0 mg, 10 mol%) in NMP (1.0 mL). The reaction was stirred at 28 °C for 24 h under N_2 atmosphere. Crude ^{1}H NMR using mesitylene (0.20 mmol) as internal standard gave product **43** (90%).

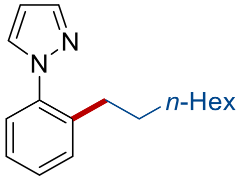

1-(2-octylphenyl)-1H-pyrazole (44a)

The general procedure **B** was followed using 1-phenyl-1*H*-pyrazole (**27k**) (57.7 mg, 0.4 mmol) and *n*-octyl bromide (**9**) (207.3 µL, 1.20 mmol), and [RuCl$_2$(benzene)]$_2$ **15** (10.0 mg, 5 mol%) in 1,4-dioxane (2.0 mL). The reaction was stirred at 70 °C for 20 h under N_2 atmosphere. Purification by column chromatography

(*n*-hexane/EtOAc = 30:1) yielded **44** (**44a**: 88.2 mg, 86%; **44b**: 14.7 mg, 10%), as a colorless oil. The characterization data is in accordance with those reported in the literature.[7]

41a:

^{1}H NMR (300 MHz, $CDCl_3$) δ 7.74 – 7.68 (m, 1H), 7.57 (dd, *J* = 2.3, 0.7 Hz, 1H), 7.37 – 7.26 (m, 4H), 6.43 (t, *J* = 2.1 Hz, 1H), 2.59 – 2.48 (m, 2H), 1.48 – 1.34 (m, 2H), 1.31 – 1.16 (m, 10H), 0.86 (t, *J* = 6.8 Hz, 3H).

^{13}C NMR (75 MHz, $CDCl_3$) δ 140.3 (CH), 139.8 (C_q), 139.2 (C_q), 130.9 (CH), 130.4 (CH), 128.7 (CH), 126.8 (CH), 126.6 (CH), 106.2 (CH), 32.0 (CH_2), 31.4 (CH_2), 30.7 (CH_2), 29.6 (CH_2), 29.4 (CH_2), 29.3 (CH_2), 22.8 (CH_2), 14.2 (CH_3).

IR (ATR): ṽ = 2955, 2924, 2853, 1581, 1456, 1380, 938, 749 cm^{-1}.

HR-MS (ESI): m/z calcd for $C_{17}H_{24}N_2$ $[M+H]^+$: 257.2012, found: 257.2023.

41b:

^{1}H NMR (300 MHz, $CDCl_3$) δ 7.71 (dd, *J* = 1.9, 0.7 Hz, 1H), 7.44 (dd, *J* = 2.3, 0.7 Hz, 1H), 7.30 (dd, *J* = 8.2, 7.0 Hz, 1H), 7.14 (d, *J* = 7.6 Hz, 2H), 6.43 (t, *J* = 2.1 Hz, 1H), 2.21 (td, *J* = 8.0, 3.3 Hz, 4H), 1.39 (d, *J* = 13.9 Hz, 4H), 1.34 – 1.16 (m, 20H), 0.86 (t, *J* = 6.8 Hz, 6H).

^{13}C NMR (75 MHz, $CDCl_3$) δ 141.2 (C_q), 139.8 (CH), 138.6 (C_q), 131.6 (CH), 129.2 (CH), 127.2 (CH), 105.7 (CH), 32.0 (CH_2), 31.4 (CH_2), 31.2 (CH_2), 29.7 (CH_2), 29.4 (CH_2), 29.3 (CH_2), 22.8 (CH_2), 14.2 (CH_3).

IR (ATR): ṽ = 2954, 2923, 2853, 1583, 1459, 1379, 938, 750 cm^{-1}.

HR-MS (ESI): m/z calcd for $C_{25}H_{40}N_2$ $[M+H]^+$: 369.3264, found: 369.3268.

The general procedure **A** was followed using 1-phenyl-1*H*-pyrazole (**27k**) (57.7 mg, 0.4 mmol) and *n*-octyl bromide (**9**) (207.3 µL, 1.20 mmol), and [Ru(*p*-cymene)OAc_2] (**3**) (14.1 mg, 10 mol%) in NMP (2.0 mL). The reaction was stirred at 45 °C for 20 h under N_2 atmosphere. Crude ^{1}H NMR using mesitylene (0.40 mmol) as internal standard gave *mono*-alkylated product **44a** (20%) and *di*-alkylated product **44b** (traces).

The general procedure **A** was followed using 1-phenyl-1*H*-pyrazole (**27k**) (57.7 mg, 0.4 mmol) and *n*-octyl bromide (**9**) (207.3 µL, 1.20 mmol), and $[Ru(t\text{-}BuCN)_5(H_2O)](BF_4)_2$ (**7**) (28.3 mg, 10 mol%) in NMP (2.0 mL). The reaction was stirred at 45 °C for 20 h under N_2 atmosphere. Crude ^{1}H NMR using mesitylene (0.40 mmol) as internal standard gave *mono*-alkylated product **44a** (62%) and *di*-alkylated product **44b** (23%).

The general procedure **B** was followed using 1-phenyl-1*H*-pyrazole (**27k**) (57.7 mg, 0.4 mmol) and *n*-octyl bromide (**9**) (207.3 µL, 1.20 mmol), and [Ru(*p*-cymene)OAc_2] (**3**) (14.1 mg, 10 mol%) in 1,4-dioxane (2.0 mL). The reaction was stirred at 70 °C for 20 h under N_2 atmosphere. Crude ^{1}H NMR using mesitylene (0.40 mmol) as internal standard gave *mono*-alkylated product **44a** (72%) and *di*-alkylated product **44b** (23%).

The general procedure **B** was followed using 1-phenyl-1*H*-pyrazole (**27k**) (57.7 mg, 0.4 mmol) and *n*-octyl bromide (**9**) (207.3 µL, 1.20 mmol), and $[Ru(t\text{-}BuCN)_5(H_2O)](BF_4)_2$ (**7**) (28.3 mg, 10 mol%) in 1,4-dioxane (2.0

mL). The reaction was stirred at 70 °C for 20 h under N_2 atmosphere. Crude ^1H NMR using mesitylene (0.40 mmol) as internal standard gave *mono*-alkylated product **44a** (84%) and *di*-alkylated product **44b** (8%).

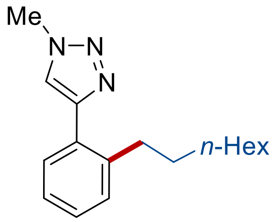

1-methyl-4-(2-octylphenyl)-*1H*-1,2,3-triazole (45a)

The general procedure **B** was followed using 1-methyl-4-phenyl-1H-1,2,3-triazole (**27l**) (31.8 mg, 0.2 mmol), *n*-octyl bromide (**9**) (103.7 µL, 0.6 mmol), and $[RuCl_2(benzene)]_2$ (**15**) (5.0 mg, 5 mol%) in 1,4-dioxane (1.0 mL). The reaction was stirred at 70 °C for 24 h under N_2 atmosphere. Purification by column chromatography (*n*-hexane/EtOAc = 10:1) yielded **45** (**45a**:16.3 mg, 30 %; **45b**: trace).

^{1}H NMR (300 MHz, $CDCl_3$) δ 7.54 – 7.46 (m, 2H), 7.25 – 7.13 (m, 3H), 4.09 (s, 3H), 2.74 – 2.63 (m, 2H), 1.55 – 1.39 (m, 2H), 1.28 – 1.11 (m, 10H), 0.83 – 0.77 (m, 3H).

^{13}C NMR (75 MHz, $CDCl_3$) δ 147.7 (C_q), 141.1 (C_q), 129.9 (CH), 129.8 (CH), 129.7 (C_q), 128.4 (CH), 126.0 (CH), 122.7 (CH), 36.8 (CH_3), 33.7 (CH_2), 32.0 (CH_2), 31.2 (CH_2), 29.7 (CH_2), 29.6 (CH_2), 29.4 (CH_2), 22.8 (CH_2), 14.2 (CH_3).

IR (ATR): ṽ = 2953, 2924, 2853, 2164, 1466, 1224, 975, 762 cm^{-1}.

HR-MS (ESI): m/z calcd for $C_{17}H_{25}N_3$ $[M+H]^+$: 272.2121, found: 272.2131.

The general procedure **B** was followed using 1-methyl-4-phenyl-1H-1,2,3-triazole (**27l**) (31.8 mg, 0.2 mmol) and *n*-octyl bromide (**9**) (103.7 µL, 0.6 mmol), and [Ru(*p*-cymene)OAc_2] (**3**) (7.1 mg, 10 mol%) in 1,4-dioxane (1.0 mL). The reaction was stirred at 70 °C for 24 h under N_2 atmosphere. Crude ^1H NMR using mesitylene (0.20 mmol) as internal standard gave product **45** (trace) and **45b** (trace).

The general procedure **B** was followed using 1-methyl-4-phenyl-1H-1,2,3-triazole (**27l**) (31.8 mg, 0.2 mmol) and *n*-octyl bromide (**9**) (103.7 µL, 0.6 mmol), and $[Ru(t\text{-}BuCN)_5(H_2O)](BF_4)_2$ (**7**) (14.0 mg, 10 mol%) in 1,4-dioxane (1.0 mL). The reaction was stirred at 70 °C for 24 h under N_2 atmosphere. Crude ^1H NMR using mesitylene (0.20 mmol) as internal standard gave product **45a** (21%) and **45b** (trace).

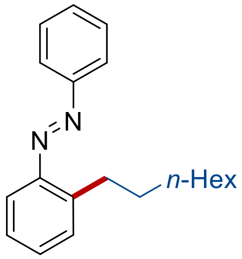

(*E*)-1-(2-octylphenyl)-2-phenyldiazene (46a)

The general procedure **B** was followed using (*E*)-1,2-diphenyldiazene (**27m**) (36.5 mg, 0.2 mmol) and *n*-octyl bromide (**9**) (103.7 µL, 0.6 mmol), and [$RuCl_2$(benzene)]$_2$ (**15**) (5.0 mg, 5 mol%) in 1,4-dioxane (1.0 mL). The reaction was stirred at 87 °C for 48 h under N_2 atmosphere. Purification by column chromatography (*n*-hexane/EtOAc = 50:1) yielded **46** (**46a**: 20.6 mg, 35%, **46b**: 9% from crude ^{1}H NMR). The characterization data is in accordance with those reported in the literature.[18]

^{1}H NMR (300 MHz, $CDCl_3$) δ 7.97 – 7.91 (m, 2H), 7.67 (dd, *J* = 8.0, 1.3 Hz, 1H), 7.57 – 7.45 (m, 3H), 7.43 – 7.33 (m, 2H), 7.32 – 7.25 (m, 1H), 3.21 – 3.10 (m, 2H), 1.77 – 1.61 (m, 2H), 1.44 – 1.22 (m, 10H), 0.91 – 0.83 (m, 3H).

^{13}C NMR (75 MHz, $CDCl_3$) δ 153.1 (C_q), 150.5 (C_q), 143.1 (C_q), 131.1 (CH), 130.9 (CH), 130.6 (CH), 129.2 (CH), 126.6 (CH), 123.1 (CH), 115.4 (CH), 32.3 (CH_2), 32.0 (CH_2), 31.6 (CH_2), 29.6 (CH_2), 29.6 (CH_2), 29.4 (CH_2), 22.8 (CH_2), 14.3 (CH_3).

IR (ATR): ṽ = 3062, 2954, 2923, 2854, 1599, 1453, 1152, 772, 739, 687 cm^{-1}.

HR-MS (ESI): m/z calcd for $C_{20}H_{26}N_2$ [M+H]$^+$: 295.2169, found: 295.2171.

The general procedure **B** was followed using (*E*)-1,2-diphenyldiazene (**27m**) (36.5 mg, 0.2 mmol) and *n*-octyl bromide (**9**) (103.7 µL, 0.6 mmol), and [Ru(*p*-cymene)OAc_2] (**3**) (7.1 mg, 10 mol%) in 1,4-dioxane (1.0 mL). The reaction was stirred at 87 °C for 48 h under N_2 atmosphere. Crude ^{1}H NMR using mesitylene (0.20 mmol) as internal standard gave product **46a** (27%) and **46b** (7%).

The general procedure **B** was followed using (*E*)-1,2-diphenyldiazene (**27m**) (36.5 mg, 0.2 mmol) and *n*-octyl bromide (**9**) (103.7 µL, 0.6 mmol), and [Ru(*t*-BuCN)$_5$(H_2O)](BF_4)$_2$ (**7**) (14.0 mg, 10 mol%) in 1,4-dioxane (1.0 mL). The reaction was stirred at 87 °C for 48 h under N_2 atmosphere. Crude ^{1}H NMR using mesitylene (0.20 mmol) as internal standard gave product **46a** (23%) and **46b** (4%).

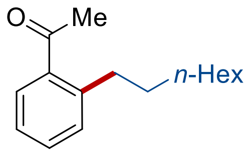

1-(2-octylphenyl)ethan-1-one (**47a**)

The general procedure **B** was followed using (*E*)-N-(4-methoxyphenyl)-1-phenylethan-1-imine (**27n**) (45.0 mg, 0.2 mmol) and *n*-octyl bromide (**9**) (103.7 µL, 0.6 mmol), and [$RuCl_2$(benzene)]$_2$ (**15**) (5.0 mg, 5 mol%) in 1,4-dioxane (1.0 mL). The reaction was stirred at 70 °C for 24 h under N_2 atmosphere. At ambient temperature, HCl (2 N, 3.0 mL) was added, and the resulting mixture was stirred for additional 3 h, and then extracted with EtOAc (3 × 20 mL). The combined organic layers were dried over Na_2SO_4 and

concentrated in vacuo. Purification by column chromatography (*n*-hexane/EtOAc = 50:1) yielded **47** (**47a**: 40.8 mg, 88 %; **47b**: trace). The characterization data is in accordance with those reported in the literature.[7]

^{1}H NMR (300 MHz, $CDCl_3$) δ 7.61 (dd, *J* = 8.0, 1.5 Hz, 1H), 7.42 – 7.34 (m, 1H), 7.28 – 7.21 (m, 2H), 2.89 – 2.78 (m, 2H), 2.57 (s, 3H), 1.60 – 1.49 (m, 2H), 1.37 – 1.22 (m, 10H), 0.91 – 0.83 (m, 3H).

^{13}C NMR (75 MHz, $CDCl_3$) δ 202.5 (C_q), 143.0 (C_q), 138.2 (C_q), 131.4 (CH), 131.3 (CH), 129.0 (CH), 125.7 (CH), 34.1 (CH_2), 32.0 (CH_2), 30.1 (CH_3), 29.8 (CH_2), 29.6 (CH_2), 29.4 (CH_2), 22.8 (CH_2), 14.3 (CH_3).

IR (ATR): ṽ = 2955, 2925, 2854, 1687, 1465, 1354, 1248, 955, 758 cm^{-1}.

HR-MS (ESI): m/z calcd for $C_{16}H_{24}O_1$ $[M+H]^+$: 233.1900, found: 233.1901.

The general procedure **B** was followed using (*E*)-N-(4-methoxyphenyl)-1-phenylethan-1-imine (**27n**) (90.0 mg, 0.4 mmol) and *n*-octyl bromide (**9**) (207 µL, 1.2 mmol), and [Ru(*p*-cymene)OAc_2] (**3**) (14.1 mg, 10 mol%) in 1,4-dioxane (2.0 mL). The reaction was stirred at 70 °C for 24 h under N_2 atmosphere. Crude ^{1}H NMR using mesitylene (0.40 mmol) as internal standard gave product **47a** (trace) and **47b**(trace).

The general procedure **B** was followed using (*E*)-N-(4-methoxyphenyl)-1-phenylethan-1-imine (**27n**) (45.0 mg, 0.2 mmol) and *n*-octyl bromide (**9**) (103.7 µL, 0.6 mmol), and $[Ru(t\text{-BuCN})_5(H_2O)](BF_4)_2$ (**7**) (14.0 mg, 10 mol%) in 1,4-dioxane (1.0 mL). The reaction was stirred at 70 °C for 24 h under N_2 atmosphere. Crude ^{1}H NMR using mesitylene (0.20 mmol) as internal standard gave product **47a** (95%) and **47b**(trace).

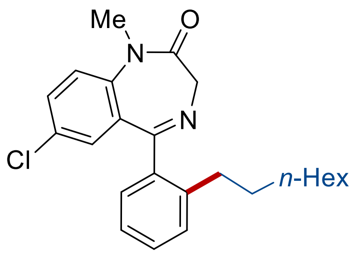

7-chloro-1-methyl-5-(2-octylphenyl)-1,3-dihydro-*2H*-benzo[*e*][1,4]diazepin-2-one (48a)

The general procedure **B** was followed using 7-chloro-1-methyl-5-phenyl-1,3-dihydro-2H-benzo[e][1,4]diazepin-2-one (**27o**) (57.0 mg, 0.2 mmol), *n*-octyl bromide (**9**) (103.7 µL, 0.6 mmol), and $[RuCl_2(benzene)]_2$ (**15**) (5.0 mg, 5 mol%) in 1,4-dioxane (1.0 mL). The reaction was stirred at 70 °C for 42 h under N_2 atmosphere. Purification by column chromatography (*n*-hexane/EtOAc = 10:1) yielded **48** (**48a**: 42.3 mg, 54%; **48b**: trace). The characterization data is in accordance with those reported in the literature.[7]

^{1}H NMR (300 MHz, $CDCl_3$) δ 7.39 (dd, *J* = 8.8, 2.5 Hz, 1H), 7.33 – 7.24 (m, 2H), 7.22 (s, 1H), 7.20 – 7.16 (m, 1H), 7.15 – 7.10 (m, 1H), 6.98 (d, *J* = 2.5 Hz, 1H), 4.78 (d, *J* = 10.9 Hz, 1H), 3.73 (d, *J* = 10.9 Hz, 1H), 3.36 (s, 3H), 2.34 – 2.00 (m, 2H), 1.26 – 0.99 (m, 12H), 0.78 (t, *J* = 6.9 Hz, 3H).

^{13}C NMR (75 MHz, $CDCl_3$) δ 171.1 (C_q), 169.8 (C_q), 141.6 (C_q), 141.2 (C_q), 138.5 (C_q), 132.0 (C_q), 131.5 (CH), 130.1 (CH), 130.0 (CH), 129.9 (CH), 129.6 (C_q), 129.3 (CH), 126.1 (CH), 122.5 (CH), 56.9 (CH_2), 34.9 (CH_3), 33.6 (CH_2), 31.9 (CH_2), 31.0 (CH_2), 29.8 (CH_2), 29.4 (CH_2), 29.3 (CH_2), 22.7 (CH_2), 14.2 (CH_3).

IR (ATR): ṽ = 2924, 2854, 1678, 1613, 1480, 1400, 1341, 1318, 1129, 911, 821, 730 cm^{-1}.

HR-MS (ESI): m/z calcd for $C_{24}H_{29}Cl_1N_2O_1$ $[M+H]^+$: 397.2041, found: 397.2053.

The general procedure **B** was followed using 7-chloro-1-methyl-5-phenyl-1,3-dihydro-2H-benzo[e][1,4]diazepin-2-one (**27o**) (57.0 mg, 0.2 mmol), *n*-octyl bromide (**9**) (103.7 µL, 0.6 mmol), and [Ru(*p*-cymene)OAc_2] (**3**) (7.1 mg, 10 mol%) in 1,4-dioxane (1.0 mL). The reaction was stirred at 70 °C for 46 h under N_2 atmosphere. Crude ^{1}H NMR using mesitylene (0.20 mmol) as internal standard gave product **48a** (28%) and **48b** (trace).

The general procedure **B** was followed using 7-chloro-1-methyl-5-phenyl-1,3-dihydro-2H-benzo[e][1,4]diazepin-2-one (**27o**) (57.0 mg, 0.2 mmol), *n*-octyl bromide (**9**) (103.7 µL, 0.6 mmol), and $[Ru(t\text{-}BuCN)_5(H_2O)](BF_4)_2$ (**7**) (14.0 mg, 10 mol%) in 1,4-dioxane (1.0 mL). The reaction was stirred at 70 °C for 46 h under N_2 atmosphere. Crude ^{1}H NMR using mesitylene (0.20 mmol) as internal standard gave product **48a** (80%) and **48b** (trace).

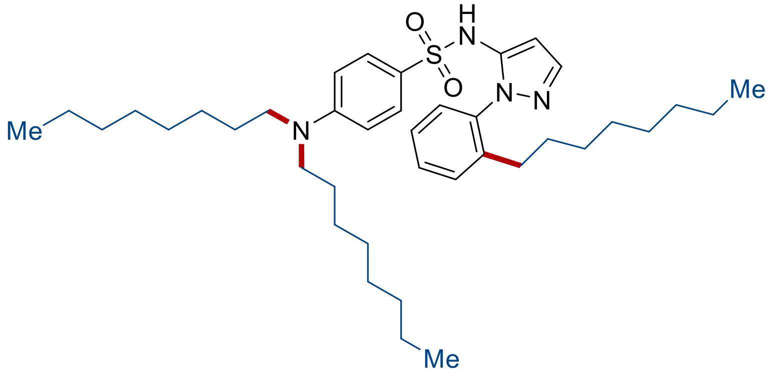

4-(dioctylamino)-*N*-(1-(2-octylphenyl)-*1H*-pyrazol-5-yl)benzenesulfonamide (49a)

The general procedure **A** was followed using 4-amino-N-(1-phenyl-1H-pyrazol-5-yl)benzenesulfonamide (**27p**) (31.5 mg, 0.1 mmol) and *n*-octyl bromide (**26f**) (207.3 µL, 1.2 mmol), and $[RuCl_2(benzene)]_2$ (**15**) (5.0 mg, 5.0 mol%) in 1,4-dioxane (1.0 mL). The reaction was stirred at 87 °C for 24 h under N_2 atmosphere. Purification by column chromatography (*n*-hexane/EtOAc = 5:1) yielded **49** (49a: 25.4 mg, 39%, 49b: 13.3 mg, 25%, 49c: 14% (from crude NMR)).

^{1}H NMR (300 MHz, $CDCl_3$) δ 7.61 (d, *J* = 2.0 Hz, 1H), 7.51 – 7.43 (m, 2H), 7.43 – 7.29 (m, 3H), 7.26 – 7.19 (m, 1H), 6.59 – 6.49 (m, 2H), 6.06 (d, *J* = 2.0 Hz, 1H), 3.23 – 3.02 (m, 4H), 2.34 – 2.21 (m, 2H), 1.73 – 1.51 (m, 4H), 1.47 – 0.96 (m, 38H), 0.93 – 0.78 (m, 9H).

^{13}**C NMR** (75 MHz, $CDCl_3$) δ 152.1 (C_q), 140.9 (C_q), 139.2 (C_q), 138.9 (CH), 137.0 (C_q), 130.4 (CH), 129.5 (CH), 129.1 (CH), 128.4 (CH), 125.9 (CH), 124.4 (C_q), 111.3 (CH), 103.5 (CH), 52.1 (CH_2), 43.4 (CH_2), 31.9 (CH_2), 31.8 (CH_2), 31.7 (CH_2), 31.2 (CH_2), 30.5 (CH_2), 29.9 (CH_2), 29.4 (CH_2), 29.3 (CH_2), 29.3 (CH_2), 29.2 (CH_2), 29.2 (CH_2), 29.1 (CH_2), 28.0 (CH_2), 27.1 (CH_2), 26.5 (CH_2), 22.7 (CH_2), 22.7 (CH_2), 22.6 (CH_2), 14.1 (CH_3), 14.1 (CH_3), 14.1 (CH_3).

IR (ATR): ṽ = 2954, 2925, 2855, 1599, 1458, 1348, 1154, 1094, 763 cm^{-1}.

HR-MS (ESI): m/z calcd for $C_{39}H_{62}N_4O_2S_1$ $[M+H]^+$: 651.4666, found: 651.4662.

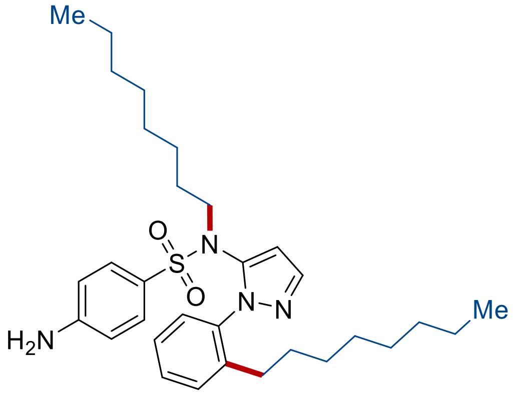

4-amino-*N*-octyl-*N*-(1-(2-octylphenyl)-*1H*-pyrazol-5-yl)benzenesulfonamide (49b)

^{1}**H NMR** (300 MHz, $CDCl_3$) δ 7.61 (d, *J* = 2.0 Hz, 1H), 7.49 – 7.44 (m, 2H), 7.42 – 7.30 (m, 3H), 7.26 – 7.20 (m, 1H), 6.68 – 6.61 (m, 2H), 6.04 (d, *J* = 2.0 Hz, 1H), 4.18 (s, 2H), 3.20 – 3.09 (m, 2H), 2.33 – 2.22 (m, 2H), 1.66 – 1.49 (m, 4H), 1.36 – 1.08 (m, 24H), 0.91 – 0.74 (m, 6H).

^{13}**C NMR** (75 MHz, $CDCl_3$) δ 150.9 (C_q), 141.0 (C_q), 139.0 (C_q), 138.9 (CH), 136.9 (C_q), 130.4 (CH), 129.6 (CH), 129.1 (CH), 128.3 (CH), 126.4 (C_q), 125.9 (CH), 113.8 (CH), 103.5 (CH), 63.1 (CH_2), 52.2 (CH_2), 32.8 (CH_2), 31.9 (CH_2), 31.7 (CH_2), 31.2 (CH_2), 30.5 (CH_2), 29.9 (CH_2), 29.4 (CH_2), 29.3 (CH_2), 29.1 (CH_2), 29.1 (CH_2), 28.0 (CH_2), 26.5 (CH_2), 22.7 (CH_2), 22.6 (CH_2), 14.1 (CH_3), 14.1 (CH_3).

IR (ATR): ṽ = 3382, 2925, 2854, 1738, 1631, 1595, 1372, 1235, 1158, 1044, 765 cm^{-1}.

HR-MS (ESI): m/z calcd for $C_{31}H_{46}N_4O_2S_1$ $[M+H]^+$: 539.3414, found: 539.3411.

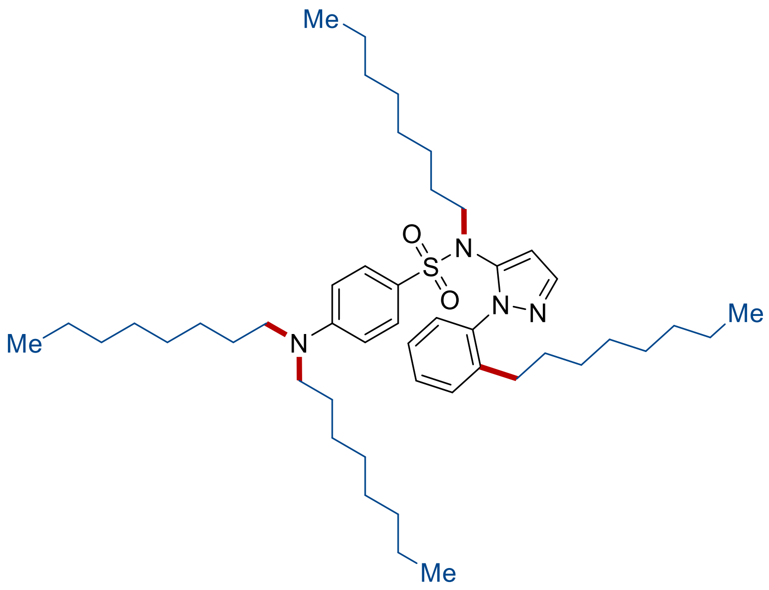

4-(dioctylamino)-*N*-octyl-*N*-(1-(2-octylphenyl)-*1H*-pyrazol-5-yl)benzenesulfonamide (49c)

Clean NMR spectrum for **49c** were not obtained.

The general procedure **B** was followed using 4-amino-N-(1-phenyl-1H-pyrazol-5-yl)benzenesulfonamide (**27p**) (31.5 mg, 0.1 mmol) and *n*-octyl bromide (**26f**) (414.6 µL, 2.4 mmol), and [Ru(*p*-cymene)OAc_2] (**3**) (7.1 mg, 20 mol%) in 1,4-dioxane (1.0 mL). The reaction was stirred at 87 °C for 24 h under N_2 atmosphere. Crude ^{1}H NMR using mesitylene (0.10 mmol) as internal standard gave product **49a** (48%), **49b** (23%), **49c** (8%).

The general procedure **B** was followed using 4-amino-N-(1-phenyl-1H-pyrazol-5-yl)benzenesulfonamide (**27o**) (31.5 mg, 0.1 mmol) and *n*-octyl bromide (**26f**) (414.6 µL, 2.4 mmol), and [Ru(*t*-BuCN)$_5$(H_2O)](BF_4)$_2$ (**7**) (14.0 mg, 20 mol%) in 1,4-dioxane (1.0 mL). The reaction was stirred at 87 °C for 24 h under N_2 atmosphere. Crude ^{1}H NMR using mesitylene (0.10 mmol) as internal standard gave product **49a** (38%), **49b** (20%), **49c** (10%).

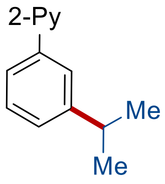

2-(3-isopropylphenyl)pyridine (52a)

The general procedure **A** was followed using 2-phenylpyridine (**8**) (31.1 mg, 0.2 mmol) and 2-bromopropane (**51a**) (56.4 µL, 0.6 mmol), and [Ru(*p*-cymene)OAc_2] (**3**) (7.1 mg, 10 mol%) in NMP (1.0 mL). The reaction was stirred at 45 °C for 44 h under N_2 atmosphere. Purification by column chromatography (*n*-hexane/EtOAc = 20:1) yielded **52** (**52a**: 19.8 mg, 50%, **52b**: 7% from crude ^{1}H NMR), as a colorless oil. The characterization data is in accordance with those reported in the literature.[19]

^{1}**H NMR** (400 MHz, $CDCl_3$) δ 8.73 – 8.68 (m, 1H), 7.88 (t, *J* = 1.9 Hz, 1H), 7.79 – 7.70 (m, 3H), 7.41 (t, *J* = 7.6 Hz, 1H), 7.32 – 7.28 (m, 1H), 7.22 (ddd, *J* = 6.0, 4.8, 2.5 Hz, 1H), 3.02 (hept, *J* = 6.9 Hz, 1H), 1.33 (s, 3H), 1.31 (s, 3H).

^{13}**C NMR** (101 MHz, $CDCl_3$) δ 158.0 (C_q), 149.8 (CH), 149.6 (C_q), 139.6 (C_q), 136.8 (CH), 128.9 (CH), 127.2 (CH), 125.4 (CH), 124.6 (CH), 122.1 (CH), 120.9 (CH), 34.4(CH), 24.2 (CH_3), 24.2 (CH_3).

IR (ATR): ṽ =2985, 1737, 1448, 1373, 1234, 1044, 938, 847, 786 cm^{-1}.

HR-MS (ESI): m/z calcd for $C_{14}H_{15}N_1$ $[M+H]^+$: 198.1277, found: 198.1283.

The general procedure **A** was followed using 2-phenylpyridine (**8**) (31.1 mg, 0.2 mmol) and 2-bromopropane (**51a**) (56.4 µL, 0.6 mmol), and $[Ru(t\text{-}BuCN)_5(H_2O)](BF_4)_2$ (**7**) (14.0 mg, 10 mol%) in NMP (1.0 mL). The reaction was stirred at 45 °C for 44 h under N_2 atmosphere. Crude ^{1}H NMR using mesitylene (0.20 mmol) as internal standard gave product **52a** (54%), and **52b** (13%).

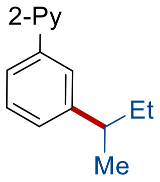

2-(3-(*sec*-butyl)phenyl)pyridine (26a)

The general procedure **A** was followed using 2-phenylpyridine (**8**) (62.1 mg, 0.4 mmol), 2-bromobutane (**25** or **51b**) (131.0 µL, 1.20 mmol), and [Ru(*p*-cymene)OAc_2] (**3**) (14.1 mg, 10 mol%) in NMP (2.0 mL). The reaction was stirred at 45 °C for 20 h under N_2 atmosphere. Purification by column chromatography (*n*-hexane/EtOAc = 20:1) yielded product **26a** (49.0 mg, 58%) and **26b** (9%, crude ^{1}H NMR yield). The characterization data is in accordance with those reported in the literature.[19]

^{1}**H NMR** (500 MHz, $CDCl_3$) δ 8.70 (dt, *J* = 4.9, 1.4 Hz, 1H), 7.84 (t, *J* = 1.8 Hz, 1H), 7.78 (ddd, *J* = 7.7, 1.9, 1.1 Hz, 1H), 7.76 – 7.71 (m, 2H), 7.40 (t, *J* = 7.7 Hz, 1H), 7.25 (dt, *J* = 7.8, 1.4 Hz, 1H), 7.23 – 7.19 (m, 1H), 2.71 (h, *J* = 7.0 Hz, 1H), 1.73 – 1.60 (m, 2H), 1.30 (d, *J* = 7.0 Hz, 3H), 0.86 (t, *J* = 7.4 Hz, 3H).

^{13}**C NMR** (126 MHz, $CDCl_3$) δ 158.0 (C_q), 149.8 (CH), 148.4 (C_q), 139.5 (C_q), 136.8 (CH), 128.8 (CH), 127.8 (CH), 126.0 (CH), 124.6 (CH), 122.1 (CH), 120.8 (CH), 42.0 (CH), 31.3 (CH_2), 22.0 (CH_3), 12.5 (CH_3).

IR (ATR): ṽ =2959, 2929, 2872, 1584, 1564, 1461, 1434, 1415, 1152, 770 cm^{-1}.

HR-MS (ESI): m/z calcd for $C_{15}H_{17}N_1$ $[M+H]^+$: 212.1434, found: 212.1440.

The general procedure **A** was followed using 2-phenylpyridine (**8**) (62.1 mg, 0.4 mmol) and 2-bromobutane (**25** or **51b**) (131.0 µL, 1.20 mmol), and $[Ru(t\text{-}BuCN)_5(H_2O)](BF_4)_2$ (**7**) (28.3 mg, 10 mol%) in NMP (2.0 mL). The reaction was stirred at 45 °C for 20 h under N_2 atmosphere. Crude ^{1}H NMR using mesitylene (0.40 mmol) as internal standard gave product **26a** (55%), and **26b** (10%).

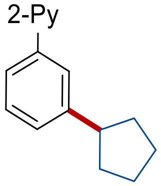

2-(3-cyclopentylphenyl)pyridine (53a)

The general procedure **A** was followed using 2-phenylpyridine (**8**) (62.1 mg, 0.4 mmol) and bromocyclopentane (**51c**) (128.7 µL, 1.20 mmol), and [Ru(*p*-cymene)OAc$_2$] (**3**) (14.1 mg, 10 mol%) in NMP (2.0 mL). The reaction was stirred at 45 °C for 24 h under N_2 atmosphere. Purification by column chromatography (*n*-hexane/EtOAc = 20:1) yielded **53** (**53a**: 36.6 mg, 41%; **53b**: 3% from crude ^{1}H NMR), as a colorless oil. The characterization data is in accordance with those reported in the literature.[19]

^{1}H NMR (500 MHz, $CDCl_3$) δ 8.72 – 8.68 (m, 1H), 7.90 (td, *J* = 1.8, 0.9 Hz, 1H), 7.78 – 7.75 (m, 1H), 7.75 – 7.70 (m, 2H), 7.40 (td, *J* = 7.7, 0.5 Hz, 1H), 7.33 – 7.30 (m, 1H), 7.22 (ddd, *J* = 6.4, 4.8, 2.1 Hz, 1H), 3.09 (tt, *J* = 9.1, 7.3 Hz, 1H), 2.17 – 2.08 (m, 2H), 1.90 – 1.80 (m, 2H), 1.74 – 1.63 (m, 4H).

^{13}C NMR (126 MHz, $CDCl_3$) δ 158.0 (C_q), 149.8 (CH), 147.2 (C_q), 139.5 (C_q), 136.8 (CH), 128.8 (CH), 127.9 (CH), 126.0 (CH), 124.5 (CH), 122.1 (CH), 120.8 (CH), 46.2 (CH), 34.8 (CH_2), 34.8 (CH_2), 25.7 (CH_2), 25.7 (CH_2).

IR (ATR): ṽ = 2955, 2867, 1585, 1564, 1462, 1434, 1152, 991, 772 cm^{-1}.

HR-MS (ESI): m/z calcd for $C_{16}H_{17}N_1$ $[M+H]^+$: 224.1434, found: 224.1438.

The general procedure **A** was followed using 2-phenylpyridine (**8**) (62.1 mg, 0.4 mmol) and bromocyclopentane (**51c**) (128.7 µL, 1.20 mmol), and [Ru(*t*-BuCN)$_5$(H$_2$O)](BF$_4$)$_2$ (**7**) (28.3 mg, 10 mol%) in NMP (2.0 mL). The reaction was stirred at 45 °C for 24 h under N_2 atmosphere. Crude ^{1}H NMR using mesitylene (0.40 mmol) as internal standard gave product **53a** (47%), and **53b** (7%).

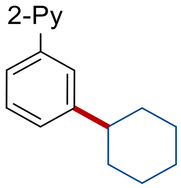

2-(3-cyclohexylphenyl)pyridine (54a)

The general procedure **A** was followed using 2-phenylpyridine (**8**) (62.1 mg, 0.4 mmol), bromocyclohexane (**51d**) (147.8 µL, 1.20 mmol), and [Ru(*p*-cymene)OAc$_2$] (**3**) (14.1 mg, 10 mol%) in NMP (2.0 mL). The reaction was stirred at 55 °C for 20 h under N_2 atmosphere. Purification by column chromatography (*n*-hexane/EtOAc = 20:1) yielded **54a** (45.6 mg, 48%), as a colorless oil. The characterization data is in accordance with those reported in the literature.[19]

^{1}H NMR (500 MHz, $CDCl_3$) δ 8.72 – 8.68 (m, 1H), 7.88 (tt, *J* = 1.9, 0.6 Hz, 1H), 7.77 (ddd, *J* = 7.7, 1.9, 1.2 Hz, 1H), 7.74 – 7.71 (m, 2H), 7.40 (td, *J* = 7.7, 0.5 Hz, 1H), 7.29 – 7.26 (m, 1H), 7.22 (ddd, *J* = 5.7, 4.8, 2.8 Hz, 1H), 2.61 (tt, *J* = 11.9, 3.4 Hz, 1H), 1.97 – 1.73 (m, 5H), 1.56 – 1.22 (m, 5H).

^{13}C NMR (126 MHz, $CDCl_3$) δ 158.0 (C_q), 149.8 (CH), 148.8 (C_q), 139.5 (C_q), 136.8 (CH), 128.8 (CH), 127.6 (CH), 125.7 (CH), 124.6 (CH), 122.1 (CH), 120.8 (CH), 44.9 (CH), 34.6 (CH_2), 27.1 (CH_2), 26.3 (CH_2).

IR (ATR): ṽ = 2982, 2927, 2852, 1736, 1585, 1462, 1372, 1234, 1044, 773 cm^{-1}.

HR-MS (ESI): m/z calcd for $C_{17}H_{19}N_1$ $[M+H]^+$: 238.1590, found: 238.1599.

The general procedure **A** was followed using 2-phenylpyridine (**8**) (31.1 mg, 0.2 mmol) and bromocyclohexane (**51d**) (73.9 µL, 0.6 mmol), and [Ru(*p*-cymene)OAc_2] (**3**) (7.1 mg, 10 mol%) in NMP (1.0 mL). The reaction was stirred at 45 °C for 44 h under N_2 atmosphere. Crude ^{1}H NMR using mesitylene (0.20 mmol) as internal standard gave product **54a** (35%), and **54b** (3%).

The general procedure **A** was followed using 2-phenylpyridine (**8**) (31.1 mg, 0.2 mmol) and bromocyclohexane (**51d**) (73.9 µL, 0.6 mmol), and $[Ru(t\text{-}BuCN)_5(H_2O)](BF_4)_2$ (**7**) (14.0 mg, 10 mol%) in NMP (1.0 mL). The reaction was stirred at 45 °C for 44 h under N_2 atmosphere. Crude ^{1}H NMR using mesitylene (0.20 mmol) as internal standard gave product **54a** (45%), and **54b** (4%).

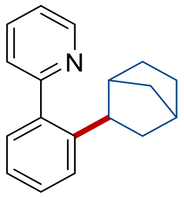

2-(2-((2R)-bicyclo[2.2.1]heptan-2-yl)phenyl)pyridine (55a-*ortho*)

The general procedure **A** was followed using 2-phenylpyridine (**8**) (62.1 mg, 0.4 mmol) and *exo*-2-bromonorbornane (**51e**) (140.1 µL, 1.20 mmol), and [Ru(*p*-cymene)OAc_2] (**3**) (14.1 mg, 10 mol%) in NMP (2.0 mL). The reaction was stirred at 50 °C for 20 h under N_2 atmosphere. Purification by column chromatography (*n*-hexane/EtOAc = 20:1) yielded **55** (**55a**: 63%, 62.8 mg, *o* : *m* = 41.8 mg : 20.9 mg, **55b**: 6.87 mg, 5%).

55a- *ortho*:

^{1}H NMR (400 MHz, $CDCl_3$) δ 8.73 – 8.64 (m, 1H), 7.79 – 7.67 (m, 1H), 7.45 – 7.32 (m, 3H), 7.31 – 7.19 (m, 3H), 3.52 – 3.36 (m, 1H, rotamer), 3.05 – 2.84 (m, 1H), 2.46 – 2.06 (m, 2H), 1.78 – 0.98 (m, 8H).

^{13}C NMR (101 MHz, $CDCl_3$) δ 160.9 (C_q), 149.1 (CH), 145.3 (C_q), 140.8 (C_q), 136.0 (CH), 129.8 (CH), 128.3 (CH), 125.7 (CH), 125.3 (CH), 124.5 (CH), 121.6 (CH), 43.3 (CH), 42.7 (CH), 40.1 (CH_2), 36.9 (CH), 36.5 (CH_2), 30.4 (CH_2), 28.7 (CH_2).

IR (ATR): ṽ = 3058, 2947, 2868, 1585, 1562, 1468, 1424, 1149, 1024, 796, 748 cm^{-1}.

HR-MS (ESI): m/z calcd for $C_{18}H_{19}N_1$ $[M+H]^+$: 250.1590, found: 250.1595.

55a-*meta*:

^{1}H NMR (400 MHz, $CDCl_3$) δ 8.70 (dt, *J* = 5.2, 1.3 Hz, 1H), 7.86 (t, *J* = 1.9 Hz, 1H), 7.77 – 7.69 (m, 2H), 7.39 (t, *J* = 7.7 Hz, 1H), 7.31 – 7.26 (m, 1H), 7.24 – 7.16 (m, 2H), 2.84 (dd, *J* = 8.9, 5.8 Hz, 1H), 2.46 – 2.32 (m, 2H), 1.87 – 1.69 (m, 2H), 1.43 – 1.08 (m, 6H).

^{13}C NMR (101 MHz, $CDCl_3$) δ 157.9 (C_q), 149.7 (CH), 148.2 (C_q), 139.3 (C_q), 136.7 (CH), 128.6 (CH), 127.6 (CH), 126.0 (CH), 124.1 (CH), 122.0 (CH), 120.8 (CH), 47.5 (CH), 43.1 (CH), 39.2 (CH_2), 36.9 (CH), 36.2 (CH_2), 30.7 (CH_2), 28.9 (CH_2).

IR (ATR): ṽ = 3058, 2947, 2868, 1585, 1562, 1468, 1424, 1149, 1024, 796, 748 cm^{-1}.

HR-MS (ESI): m/z calcd for $C_{18}H_{19}N_1$ $[M+H]^+$: 250.1590, found: 250.1595.

55b (a mixture):

^{1}H NMR (400 MHz, $CDCl_3$) δ 8.73 – 8.65 (m, 1H), 7.78 – 7.68 (m, 1H), 7.62 (d, *J* = 1.7 Hz, 1H), 7.38 – 7.28 (m, 1H), 7.24 – 7.15 (m, 2H), 7.14 (d, *J* = 2.0 Hz, 1H), 2.94 – 2.69 (m, 2H), 2.46 – 2.21 (m, 4H), 1.87 – 0.78 (m, 16H).

^{13}C NMR (101 MHz, $CDCl_3$) δ 161.3, 158.4, 149.6, 149.1, 147.9, 144.4, 142.2, 140.5, 139.3, 136.6, 135.9, 128.7, 126.8, 126.8, 125.6, 124.5, 123.1, 121.8, 121.4, 120.9, 47.6, 46.9, 43.2, 43.2, 43.1, 42.9, 42.9, 42.9, 40.1, 40.1, 39.2, 39.0, 39.0, 36.9, 36.8, 36.5, 36.5, 36.3, 36.1, 30.7, 30.6, 30.6, 30.4, 29.7, 29.0, 29.0, 28.7.

IR (ATR): ṽ = 2947, 2867, 1739, 1585, 1564, 1472, 1453, 1238, 1047, 784, 746 cm^{-1}.

HR-MS (ESI): m/z calcd for $C_{25}H_{29}N_1$ $[M+H]^+$: 344.2373, found: 344.2376.

The general procedure **A** was followed using 2-phenylpyridine (**8**) (62.1 mg, 0.4 mmol) and *exo*-2-bromonorbornane (**51e**) (140.1 µL, 1.20 mmol), and $[Ru(t\text{-}BuCN)_5(H_2O)](BF_4)_2$ (**7**) (28.3 mg, 10 mol%) in NMP (2.0 mL). The reaction was stirred at 50 °C for 20 h under N_2 atmosphere. Crude ^{1}H NMR using mesitylene (0.40 mmol) as internal standard gave product **55a** (71%, *o* : *m* = 2 : 1), and **55b** (7%).

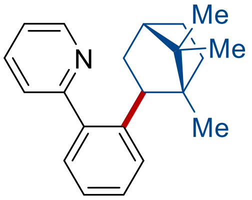

2-(2-((1R,2S,4S)-1,7,7-trimethylbicyclo[2.2.1]heptan-2-yl)phenyl)pyridine (**56a-*ortho***)

The general procedure **A** was followed using 2-phenylpyridine (**8**) (62.1 mg, 0.4 mmol), (1S,4S)-2-bromo-1,7,7-trimethylbicyclo[2.2.1]heptane (**51f**) (260.0 mg, 1.20 mmol), and $[Ru(p\text{-cymene})OAc_2]$ (**3**) (14.1 mg, 10 mol%) in NMP (2.0 mL). The reaction was stirred at 45 °C for 20 h under N_2 atmosphere. Purification by

column chromatography (*n*-hexane/EtOAc = 20:1) yielded **56** (**56a**: *o* + *m* = 21 mg + 14 mg = 18% + 12%, **56b**: trace), as a colorless oil.

56a- *ortho*:

^{1}H NMR (300 MHz, $CDCl_3$) δ 8.66 (ddd, *J* = 4.9, 1.9, 0.9 Hz, 1H), 7.71 (td, *J* = 7.7, 1.9 Hz, 1H), 7.50 (dd, *J* = 7.9, 1.3 Hz, 1H), 7.44 – 7.37 (m, 1H), 7.36 – 7.25 (m, 3H), 7.21 (ddd, *J* = 7.6, 4.9, 1.2 Hz, 1H), 3.97 (ddd, *J* = 11.7, 5.7, 2.4 Hz, 1H), 2.23 (dddd, *J* = 13.1, 11.7, 4.5, 3.3 Hz, 1H), 1.89 – 1.66 (m, 2H), 1.59 – 1.46 (m, 2H), 1.39 (ddd, *J* = 12.0, 9.2, 4.4 Hz, 1H), 1.15 – 1.02 (m, 1H), 0.80 (s, 3H), 0.77 (s, 3H).

^{13}C NMR (75 MHz, $CDCl_3$) δ 161.5 (C_q), 148.8 (CH), 142.6 (C_q), 140.5 (C_q), 136.0 (CH), 130.2 (CH), 129.3 (CH), 127.7 (CH), 125.8 (CH), 125.2 (CH), 121.5 (CH), 51.0 (C_q), 50.3 (C_q), 45.6 (CH), 42.9 (CH), 35.6 (CH_2), 28.7 (CH_2), 28.6 (CH_2), 19.9 (CH_3), 18.4 (CH_3), 14.4 (CH_3).

IR (ATR): ṽ = 3057, 2949, 2882, 1585, 1468, 1389, 1023, 796, 750 cm^{-1}.

HR-MS (ESI): m/z calcd for $C_{21}H_{25}N_1$ $[M+H]^+$: 292.2060, found: 292.2064.

56a-*meta*

^{1}H NMR (300 MHz, $CDCl_3$) δ 8.74 – 8.64 (m, 1H), 7.94 – 7.67 (m, 4H), 7.47 – 7.27 (m, 2H), 7.24 – 7.18 (m, 1H), 3.28 – 2.95 (m, 1H), 2.52 – 2.11 (m, 1H), 1.99 – 1.56 (m, 4H), 1.51 – 1.30 (m, 2H), 1.06 (s, 1.5H), 0.95 (s, 1.5H), 0.85 (s, 3H), 0.82 (s, 1.5H), 0.77 (s, 1.5H). (A mixture of two rotamers)

^{13}C NMR (75 MHz, $CDCl_3$) δ 158.0, 158.0, 149.7, 149.7, 143.9, 143.1, 138.9, 138.7, 136.7, 129.8, 129.8, 128.2, 128.2, 128.1, 127.95, 124.6, 124.2, 121.9, 121.9, 120.7, 120.7, 52.5, 50.2, 50.0, 49.9, 49.8, 48.0, 45.7, 45.5, 40.6, 34.4, 33.5, 28.5, 28.5, 27.6, 21.4, 20.3, 19.9, 18.7, 14.8, 14.5. (A mixture of two rotamers)

IR (ATR): ṽ = 2951, 2876, 1584, 1564, 1460, 1433, 1387, 768, 743 cm^{-1}.

HR-MS (ESI): m/z calcd for $C_{21}H_{25}N_1$ $[M+H]^+$: 292.2060, found: 292.2062.

56b was obtained as a mixture.

The general procedure **A** was followed using 2-phenylpyridine (**8**) (62.1 mg, 0.4 mmol), (1S,4S)-2-bromo-1,7,7-trimethylbicyclo[2.2.1]heptane (**51f**) (260.0 mg, 1.20 mmol), and $[Ru(t\text{-}BuCN)_5(H_2O)](BF_4)_2$ (**7**) (28.3 mg, 10 mol%) in NMP (2.0 mL). The reaction was stirred at 45 °C for 20 h under N_2 atmosphere. Crude ^{1}H NMR using mesitylene (0.40 mmol) as internal standard gave product **56a** (44%, *o* + *m* = 26% + 18%), and **56b** (4%).

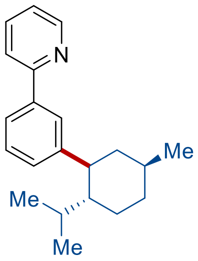

2-(3-((1S,2R,5S)-2-isopropyl-5-methylcyclohexyl)phenyl)pyridine (57a)

The general procedure **A** was followed using 2-phenylpyridine (**8**) (62.1 mg, 0.4 mmol), (1R,4S)-2-bromo-1-isopropyl-4-methylcyclohexane (**51g**) (263.0 mg, 1.20 mmol), and [Ru(*p*-cymene)OAc_2] (**3**) (14.1 mg, 10 mol%) in NMP (2.0 mL). The reaction was stirred at 45 °C for 18 h under N_2 atmosphere. Purification by column chromatography (*n*-hexane/EtOAc = 20:1) yielded **57** (**57a**: 25.8 mg, 22%, *o* + *m* = trace + 22%, **57b**: 2% from crude ^{1}H NMR), as a colorless oil.

57a-*meta*:

^{1}H NMR (600 MHz, $CDCl_3$) δ 8.75 – 8.66 (m, 1H), 7.99 – 7.16 (m, 7H), 3.38 – 2.45 (m, 1H), 2.06 – 1.12 (m, 8H), 1.11 – 0.94 (m, 1H), 0.93 – 0.84 (m, 3H), 0.83 – 0.78 (m, 3H), 0.75 – 0.23 (m, 3H).

^{13}C NMR (126 MHz, $CDCl_3$) δ 158.0 (C_q), 149.8 (CH), 147.3 (C_q), 139.6 (C_q), 136.8 (CH), 128.8 (CH), 124.6 (CH), 122.1 (CH), 120.8 (CH), 48.31, 47.4 (CH), 45.5 (CH), 35.4 (CH_2), 33.4 (CH_2), 27.6 (CH), 24.7 (CH), 22.7 (CH_3), 21.7 (CH_3), 15.6 (CH_3).

IR (ATR): ṽ = 2955, 2915, 2868, 1585, 1565, 1462, 1367, 773, 701 cm^{-1}.

HR-MS (ESI): m/z calcd for $C_{21}H_{27}N_1$ $[M+H]^+$: 294.2216, found: 294.2216.

The general procedure **A** was followed using 2-phenylpyridine (**8**) (62.1 mg, 0.4 mmol), (1R,4S)-2-bromo-1-isopropyl-4-methylcyclohexane (**51g**) (263.0 mg, 1.20 mmol), and [Ru(*p*-cymene)OAc_2] (**3**) (14.1 mg, 10 mol%) in NMP (2.0 mL). The reaction was stirred at 45 °C for 18 h under N_2 atmosphere. Crude ^{1}H NMR using mesitylene (0.40 mmol) as internal standard gave product **57a** (33%, *o* + *m* = trace + 33%), and **57b** (11%).

57b was obtained as a mixture.

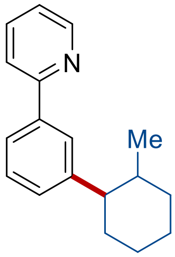

2-(3-((1R)-2-methylcyclohexyl)phenyl)pyridine (58a-*meta*)

The general procedure **A** was followed using 2-phenylpyridine (**8**) (62.1 mg, 0.4 mmol), 1-bromo-2-methylcyclohexane (**51h**) (211.2 mg, 1.20 mmol), and [Ru(*p*-cymene)OAc$_2$] (**3**) (14.1 mg, 10 mol%) in NMP (2.0 mL). The reaction was stirred at 45 °C for 18 h under N_2 atmosphere. Purification by column chromatography (*n*-hexane/EtOAc = 20:1) yielded **58** (**58a**: 25.0 mg, 25%, *o* + *m* = trace + 25%, **58b**: trace), as a colorless oil.

58a-*meta*:

^{1}H NMR (300 MHz, $CDCl_3$) δ 8.70 (dq, *J* = 4.9, 1.1 Hz, 1H), 7.85 – 7.80 (m, 1H), 7.80 – 7.75 (m, 1H), 7.75 – 7.69 (m, 2H), 7.39 (t, *J* = 7.6 Hz, 1H), 7.25 – 7.18 (m, 2H), 2.19 (td, *J* = 11.3, 3.3 Hz, 1H), 1.91 – 1.21 (m, 8H), 1.20 – 1.03 (m, 1H), 0.70 (d, *J* = 6.4 Hz, 3H).

^{13}C NMR (75 MHz, $CDCl_3$) δ 158.0 (C_q), 149.8 (CH), 147.6 (C_q), 139.4 (C_q), 136.8 (CH), 128.8 (CH), 128.3 (CH), 126.4 (CH), 124.6 (CH), 122.1 (CH), 120.8 (CH), 52.8 (CH), 37.8 (CH), 35.9 (CH_2), 35.8 (CH_2), 27.1 (CH_2), 26.8 (CH_2), 21.0 (CH_3).

IR (ATR): $\tilde{v}$ = 3062, 2921, 2851, 1585, 1565, 1461, 1434, 1152, 990, 770 cm^{-1}.

HR-MS (ESI): m/z calcd for $C_{18}H_{21}N_1$ $[M+H]^+$: 252.1747, found: 252.1746.

[Ru(*p*-cymene)OAc$_2$] (**3**) selectively gave one rotamer, while [Ru(*t*-BuCN)$_5$(H$_2$O)](BF$_4$)$_2$ (**7**) gave two rotamers in a ratio of 1 : 3.

The general procedure **A** was followed using 2-phenylpyridine (**8**) (62.1 mg, 0.4 mmol), 1-bromo-2-methylcyclohexane (**51h**) (211.2 mg, 1.20 mmol), and [Ru(*t*-BuCN)$_5$(H$_2$O)](BF$_4$)$_2$ (**7**) (28.3 mg, 10 mol%) in NMP (2.0 mL). The reaction was stirred at 45 °C for 18 h under N_2 atmosphere. Crude ^{1}H NMR using mesitylene (0.40 mmol) as internal standard gave product **58a** (37%, *o* + *m* = trace + 37%), and **58b** (3%).

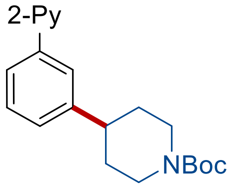

tert-Butyl 4-(3-(pyridin-2-yl)phenyl)piperidine-1-carboxylate (59a-_meta_)

The general procedure **A** was followed using 2-phenylpyridine (**8**) (62.1 mg, 0.4 mmol), *tert*-butyl 4-bromopiperidine-1-carboxylate (**51i**) (317.0 mg, 1.20 mmol), and [RuCl$_2$(benzene)]$_2$ (**15**) (10.0 mg, 5.0 mol%) in NMP (2.0 mL). The reaction was stirred at 45 °C for 20 h under N_2 atmosphere. Purification by column chromatography (*n*-hexane/EtOAc = 20:1) yielded **59** (**59a**: 82.5 mg, 61%, *o* : *m* = 1 : 3, **59b**: 11.1 mg, 5%), as a colorless oil. The characterization data is in accordance with those reported in the literature.[19]

59a-*meta*:

^{1}H NMR (300 MHz, $CDCl_3$) δ 8.72 – 8.66 (m, 1H), 7.88 (t, *J* = 1.8 Hz, 1H), 7.78 (ddd, *J* = 7.6, 1.8, 1.1 Hz, 1H), 7.76 – 7.68 (m, 2H), 7.40 (t, *J* = 7.7 Hz, 1H), 7.28 – 7.17 (m, 2H), 4.26 (d, *J* = 13.1 Hz, 2H), 2.91 – 2.63 (m, 3H), 2.01 – 1.80 (m, 2H), 1.70 (qd, *J* = 12.6, 4.3 Hz, 2H), 1.48 (s, 9H).

^{13}C NMR (75 MHz, $CDCl_3$) δ 157.5 (C_q), 154.9 (C_q), 149.7 (CH), 146.4 (C_q), 139.6 (C_q), 136.8 (CH), 128.9 (CH), 127.4 (CH), 125.6 (CH), 125.0 (CH), 122.1 (CH), 120.7 (CH), 79.4 (C_q), 44.4 (CH_2), 42.9 (CH), 33.2 (CH_2), 28.5 (CH_3).

IR (ATR): ṽ = 2975, 2933, 2848, 1686, 1585, 1462, 1423, 1365, 1229, 1171, 1121, 774 cm^{-1}.

HR-MS (ESI): m/z calcd for $C_{21}H_{26}N_2O_2$ $[M+H]^+$: 339.2067, found: 339.2055.

59b:

^{1}H NMR (300 MHz, $CDCl_3$) δ 8.69 (ddd, *J* = 4.8, 1.8, 1.0 Hz, 1H), 7.79 – 7.68 (m, 2H), 7.67 (d, *J* = 1.6 Hz, 2H), 7.23 (ddd, *J* = 6.6, 4.8, 1.7 Hz, 1H), 7.10 (t, *J* = 1.7 Hz, 1H), 4.26 (d, *J* = 12.9 Hz, 4H), 2.94 – 2.64 (m, 6H), 1.94 – 1.82 (m, 4H), 1.78 – 1.59 (m, 4H), 1.49 (s, 18H).

^{13}C NMR (75 MHz, $CDCl_3$) δ 154.3 (C_q), 149.1 (CH), 146.1 (C_q), 139.3 (C_q), 136.2 (CH), 125.5 (CH), 123.1 (CH), 121.6 (CH), 120.2 (CH), 78.9 (C_q), 42.4 (CH), 32.7 (CH_2), 28.0 (CH_3).

The general procedure **A** was followed using 2-phenylpyridine (**8**) (62.1 mg, 0.4 mmol), *tert*-butyl 4-bromopiperidine-1-carboxylate (**51i**) (317.0 mg, 1.20 mmol), and [Ru(*p*-cymene)OAc_2] (**3**) (14.1 mg, 10 mol%) in NMP (2.0 mL). The reaction was stirred at 45 °C for 20 h under N_2 atmosphere. Crude ^{1}H NMR using mesitylene (0.40 mmol) as internal standard gave *mono*-alkylated product **59a** (49%, *o* : *m* = 1 : 3) and *di*-alkylated product **59b** (4%).

The general procedure **A** was followed using 2-phenylpyridine (**8**) (62.1 mg, 0.4 mmol), *tert*-butyl 4-bromopiperidine-1-carboxylate (**51i**) (317.0 mg, 1.20 mmol), and $[Ru(t\text{-}BuCN)_5(H_2O)](BF_4)_2$ (**7**) (28.3 mg, 10 mol%) in NMP (2.0 mL). The reaction was stirred at 45 °C for 20 h under N_2 atmosphere. Crude ^{1}H NMR using mesitylene (0.40 mmol) as internal standard gave *mono*-alkylated product **59a** (49% *o* : *m* = 1 : 4) and *di*-alkylated product **59b** (11%).

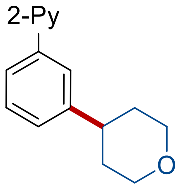

2-(3-(tetrahydro-*2H*-pyran-4-yl)phenyl)pyridine (60)

The general procedure **A** was followed using 2-phenylpyridine (**8**) (62.1 mg, 0.4 mmol) and 4-bromotetrahydro-*2H*-pyran (**51j**) (135.0 µL, 1.20 mmol), and [Ru(*p*-cymene)OAc_2] (**3**) (14.1 mg, 10 mol%) in NMP (2.0 mL). The reaction was stirred at 45 °C for 44 h under N_2 atmosphere. Purification by column

chromatography (*n*-hexane/EtOAc = 20:1) yielded **60** (**60a**: 32.6 mg, 34%; **60b**: 12.9 mg, 10%), as a colorless oil. The characterization data is in accordance with those reported in the literature.[20]

60a:

^{1}H NMR (500 MHz, $CDCl_3$) δ 8.71 (ddd, *J* = 4.8, 1.8, 1.0 Hz, 1H), 7.90 (tt, *J* = 1.8, 0.6 Hz, 1H), 7.79 (ddd, *J* = 7.7, 1.8, 1.1 Hz, 1H), 7.76 (ddd, *J* = 8.0, 7.1, 1.8 Hz, 1H), 7.74 – 7.71 (m, 1H), 7.46 – 7.41 (m, 1H), 7.29 (dddd, *J* = 7.6, 1.7, 1.1, 0.5 Hz, 1H), 7.24 (ddd, *J* = 7.1, 4.8, 1.5 Hz, 1H), 4.17 – 4.05 (m, 2H), 3.55 (td, *J* = 11.7, 2.3 Hz, 2H), 2.94 – 2.80 (m, 1H), 1.93 – 1.88 (m, 2H), 1.86 – 1.80 (m, 2H).

^{13}C NMR (126 MHz, $CDCl_3$) δ 157.7 (C_q), 149.8 (CH), 146.6 (C_q), 139.8 (C_q), 136.9 (CH), 129.1 (CH), 127.4 (CH), 125.8 (CH), 125.1 (CH), 122.3 (CH), 120.8 (CH), 68.6 (CH_2), 68.6 (CH_2), 41.9 (CH), 34.1 (CH_2), 34.1 (CH_2).

IR (ATR): ṽ = 2935, 2914, 2849, 1709,1584, 1565, 1462, 1435, 1237, 1129, 1085, 773 cm^{-1}.

HR-MS (ESI): m/z calcd for $C_{16}H_{17}N_1O_1$ $[M+H]^+$: 240.1383, found: 240.1392.

60b:

^{1}H NMR (300 MHz, $CDCl_3$) δ 8.75 – 8.64 (m, 1H), 7.79 – 7.67 (m, 3H), 7.38 – 7.20 (m, 2H), 7.19 – 7.12 (m, 1H), 4.18 – 3.93 (m, 4H), 3.64 – 3.25 (m, 4H), 2.94 – 2.70 (m, 2H), 2.03 – 1.69 (m, 8H).

^{13}C NMR (75 MHz, $CDCl_3$) δ 157.8 (C_q), 149.8 (CH), 146.8 (C_q, 2C), 140.0 (C_q), 136.9 (CH), 126.0 (CH), 123.8 (CH, 2C), 122.2 (CH), 120.9 (CH), 68.6 (CH_2, 4C), 41.9 (CH, 2C),34.2 (CH_2, 4C).

IR (ATR): ṽ = 3050, 2934, 2842, 1584, 1565, 1462, 1435, 1237, 1130, 1086, 773 cm^{-1}.

HR-MS (ESI): m/z calcd for $C_{21}H_{25}N_1O_2$ $[M+H]^+$: 324.1958, found: 324.1949.

The general procedure **A** was followed using 2-phenylpyridine (**8**) (62.1 mg, 0.4 mmol) and 4-bromotetrahydro-*2H*-pyran (**51j**) (135.0 µL, 1.20 mmol), and $[Ru(t\text{-}BuCN)_5(H_2O)](BF_4)_2$ (**7**) (28.3 mg, 10 mol%) in NMP (2.0 mL). The reaction was stirred at 45 °C for 44 h under N_2 atmosphere. Crude ^{1}H NMR using mesitylene (0.40 mmol) as internal standard gave *mono*-alkylated product **60a** (48%) and *di*-alkylated product **60b** (12%).

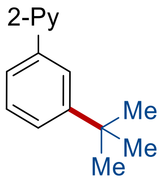

2-(3-(*tert*-butyl)phenyl)pyridine (61a)

The general procedure **A** was followed using 2-phenylpyridine (**8**) (62.1 mg, 0.4 mmol) and 2-bromo-2-methylpropane (**51k**) (135.0 µL, 1.20 mmol), and $[Ru(p\text{-}cymene)OAc_2]$ (**3**) (14.1 mg, 10 mol%) in NMP (2.0 mL). The reaction was stirred at 53 °C for 20 h under N_2 atmosphere. Purification by column

chromatography (*n*-hexane/EtOAc = 20:1) yielded **61** (**61a**: 38.9 mg, 46%; **61b**: trace), as a colorless oil. The characterization data is in accordance with those reported in the literature.[21]

61a:

^{1}H NMR (500 MHz, $CDCl_3$) δ 8.71 (dt, *J* = 4.9, 1.4 Hz, 1H), 8.05 (t, *J* = 1.9 Hz, 1H), 7.78 – 7.71 (m, 3H), 7.47 (dt, *J* = 7.9, 1.6 Hz, 1H), 7.42 (t, *J* = 7.7 Hz, 1H), 7.22 (ddd, *J* = 6.7, 4.8, 2.0 Hz, 1H), 1.40 (s, 9H).

^{13}C NMR (126 MHz, $CDCl_3$) δ 158.2 (C_q), 151.8 (C_q), 149.8 (CH), 139.4 (C_q), 136.8 (CH), 128.6 (CH), 126.2 (CH), 124.3 (CH), 124.1 (CH), 122.0 (CH), 120.9 (CH), 35.0 (C_q), 31.5 (CH_3).

IR (ATR): $\tilde{\nu}$ = 2983, 2966, 1736, 1585, 1462, 1372, 1234, 1044, 775 cm^{-1}.

HR-MS (ESI): m/z calcd for $C_{15}H_{17}N_1$ $[M+H]^+$: 212.1434, found: 212.1443.

The general procedure **A** was followed using 2-phenylpyridine (**8**) (31.1 mg, 0.2 mmol) and 2-bromo-2-methylpropane (**51k**) (67.5 µL, 0.6 mmol), and [Ru(*p*-cymene)OAc_2] (**3**) (7.1 mg, 10 mol%) in NMP (1.0 mL). The reaction was stirred at 45 °C for 44 h under N_2 atmosphere. Crude ^{1}H NMR using mesitylene (0.20 mmol) as internal standard gave *mono*-alkylated product **61a** (55%) and *di*-alkylated product **61b** (traces).

The general procedure **A** was followed using 2-phenylpyridine (**8**) (31.1 mg, 0.2 mmol) and 2-bromo-2-methylpropane (**51k**) (67.5 µL, 0.6 mmol), and $[Ru(t\text{-}BuCN)_5(H_2O)](BF_4)_2$ (**7**) (14.0 mg, 10 mol%) in NMP (1.0 mL). The reaction was stirred at 45 °C for 44 h under N_2 atmosphere. Crude ^{1}H NMR using mesitylene (0.20 mmol) as internal standard gave *mono*-alkylated product **61a** (79%) and *di*-alkylated product **61b** (3%).

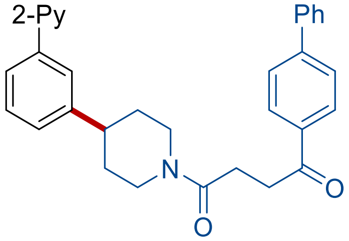

1-([1,1'-biphenyl]-4-yl)-4-(4-(3-(pyridin-2-yl)phenyl)piperidin-1-yl)butane-1,4-dione (62a)

The general procedure **A** was followed using 2-phenylpyridine (**8**) (31.1 mg, 0.2 mmol) and 1-([1,1'-biphenyl]-4-yl)-4-(4-bromopiperidin-1-yl)butane-1,4-dione (**51l**) (240.2 mg, 0.6 mmol), and [Ru(*p*-cymene)OAc_2] (**3**) (7.1 mg, 10 mol%) in NMP (1.0 mL). The reaction was stirred at 45 °C for 48 h under N_2 atmosphere. Purification by column chromatography (*n*-hexane/EtOAc = 20:1) yielded **62** (**62a**: 38.0 mg, 40%; **62b**: trace), as a colorless oil.

^{1}H NMR (400 MHz, $CDCl_3$) δ 8.73 – 8.67 (m, 1H), 8.16 – 8.08 (m, 2H), 7.90 (t, *J* = 1.8 Hz, 1H), 7.81 (ddd, *J* = 7.8, 1.9, 1.2 Hz, 1H), 7.80 – 7.66 (m, 4H), 7.67 – 7.60 (m, 2H), 7.52 – 7.36 (m, 4H), 7.31 – 7.20 (m, 2H), 4.81 (ddt, *J* = 13.4, 4.5, 2.2 Hz, 1H), 4.17 (ddt, *J* = 13.6, 4.6, 2.3 Hz, 1H), 3.42 (td, *J* = 6.6, 1.5 Hz, 2H), 3.21 (td, *J* = 13.1, 2.7 Hz, 1H), 2.88 (t, *J* = 6.6 Hz, 2H), 2.69 (td, *J* = 13.0, 2.9 Hz, 1H), 2.04 – 1.91 (m, 2H), 1.86 – 1.71 (m, 2H), 1.69 (s, 1H).

^{13}C NMR (101 MHz, $CDCl_3$) δ 199.1 (C_q), 170.2 (C_q), 157.6 (C_q), 149.8 (CH), 146.0 (C_q), 145.8 (C_q), 140.1 (C_q), 139.8 (C_q), 136.9 (CH), 135.8 (C_q), 129.1 (CH), 129.1 (CH), 128.9 (CH), 128.3 (CH), 127.5 (CH), 127.4 (CH), 127.4 (CH), 125.7 (CH), 125.2 (CH), 122.3 (CH), 120.8 (CH), 46.3 (CH_2), 43.1 (CH), 42.8 (CH_2), 33.9 (CH_2), 33.9 (CH_2), 33.0 (CH_2), 27.5 (CH_2).

IR (ATR): ṽ = 2924, 2853, 1735, 1682, 1640, 1447, 1372, 1243, 1045, 774 cm^{-1}.

HR-MS (ESI): m/z calcd for $C_{32}H_{30}N_2O_2$ $[M+H]^+$: 475.2380, found: 475.2398.

The general procedure **A** was followed using 2-phenylpyridine (**8**) (31.1 mg, 0.2 mmol) and 1-([1,1'-biphenyl]-4-yl)-4-(4-bromopiperidin-1-yl)butane-1,4-dione (**51l**) (240.2 mg, 0.6 mmol), and [Ru(*t*-BuCN)$_5$(H_2O)](BF_4)$_2$ (**7**) (14.0 mg, 10 mol%) in NMP (1.0 mL). The reaction was stirred at 45 °C for 48 h under N_2 atmosphere. Crude ^{1}H NMR using mesitylene (0.20 mmol) as internal standard gave *mono*-alkylated product **62a** (43%) and *di*-alkylated product **62b** (trace).

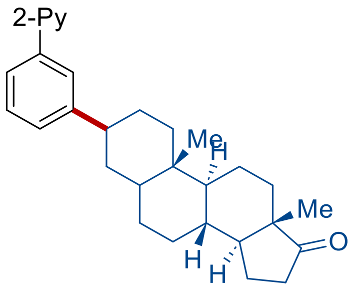

(*3S,8R,9S,10S,13S,14S*)-10,13-dimethyl-3-(3-(pyridin-2-yl)phenyl)hexadecahydro-*17H*-cyclopenta[*a*]phenanthren-17-one (63a)

The general procedure **A** was followed using 2-phenylpyridine (**8**) (31.1 mg, 0.2 mmol) and (8R,9S,10S,13S,14S)-3-bromo-10,13-dimethylhexadecahydro-17H-cyclopenta[a]phenanthren-17-one (**51m**) (212.4 mg, 0.6 mmol), and [Ru(*p*-cymene)OAc$_2$] (**3**) (7.1 mg, 10 mol%) in NMP (1.0 mL). The reaction was stirred at 45 °C for 48 h under N_2 atmosphere. Purification by column chromatography (*n*-hexane/EtOAc = 20:1) yielded **63** (**63a**: 47.0 mg, 55%; **63b**: trace), as a colorless oil.

^{1}H NMR (400 MHz, $CDCl_3$) δ 8.73 – 8.67 (m, 1H), 7.88 (t, *J* = 1.9 Hz, 1H), 7.79 – 7.70 (m, 3H), 7.43 – 7.36 (m, 1H), 7.29 (dt, *J* = 7.6, 1.6 Hz, 1H), 7.25 – 7.19 (m, 1H), 2.67 (ddd, *J* = 16.5, 11.0, 4.5 Hz, 1H), 2.49 – 2.39 (m, 1H), 2.14 – 2.03 (m, 1H), 2.01 – 1.90 (m, 1H), 1.88 – 1.67 (m, 6H), 1.62 – 1.45 (m, 5H), 1.40 – 1.23 (m, 6H), 1.19 – 0.99 (m, 2H), 0.93 (s, 3H), 0.88 (s, 3H).

^{13}C NMR (101 MHz, $CDCl_3$) δ 221.7 (C_q), 158.0 (C_q), 149.8 (CH), 148.2 (C_q), 139.6 (C_q), 136.8 (CH), 128.8 (CH), 127.6 (CH), 125.7 (CH), 124.7 (CH), 122.1 (CH), 120.9 (CH), 54.9 (CH), 51.7 (CH), 48.0 (C_q), 47.3 (CH), 45.0 (CH), 39.0 (CH_2), 36.6 (CH_2), 36.1 (C_q), 36.0 (CH_2), 35.3 (CH), 31.8 (CH_2), 31.1 (CH_2), 30.0 (CH_2), 28.7 (CH_2), 21.9 (CH_2), 20.5 (CH_2), 14.0 (CH_3), 12.7 (CH_3).

IR (ATR): ṽ = 2916, 2856, 1739, 1585, 1471, 1371, 1242, 1044, 750 cm^{-1}.

HR-MS (ESI): m/z calcd for $C_{30}H_{37}N_1O_1$ $[M+H]^+$: 428.2948, found: 428.2956.

The general procedure **A** was followed using 2-phenylpyridine (**8**) (31.1 mg, 0.2 mmol) and (8R,9S,10S,13S,14S)-3-bromo-10,13-dimethylhexadecahydro-17H-cyclopenta[a]phenanthren-17-one (**51m**) (212.4 mg, 0.6 mmol), and $[Ru(t\text{-}BuCN)_5(H_2O)](BF_4)_2$ (**7**) (14.0 mg, 10 mol%) in NMP (1.0 mL). The reaction was stirred at 45 °C for 48 h under N_2 atmosphere. Crude ^{1}H NMR using mesitylene (0.20 mmol) as internal standard gave alkylated product **63** (42%).

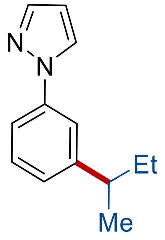

(*R*)-1-(3-(sec-butyl)phenyl)-1H-pyrazole (64a)

The general procedure **C** was followed using 1-phenyl-1*H*-pyrazole (**27j**) (57.7 mg, 0.4 mmol) and 2-bromobutane (**25**) (131.0 µL, 1.20 mmol), and [Ru(*p*-cymene)OAc_2] (**3**) (14.1 mg, 10 mol%) in 1,4-dioxane (2.0 mL). The reaction was stirred at 70 °C for 40 h under N_2 atmosphere. Purification by column chromatography (*n*-hexane/EtOAc = 30:1) yielded **64** (**64a**: 56.1 mg, 70%; **64b**: trace), as a colorless oil. The characterization data is in accordance with those reported in the literature.[11]

^{1}H NMR (300 MHz, $CDCl_3$) δ 7.92 (dd, *J* = 2.5, 0.7 Hz, 1H), 7.75 – 7.70 (m, 1H), 7.56 (t, *J* = 2.0 Hz, 1H), 7.46 (ddd, *J* = 8.0, 2.3, 1.1 Hz, 1H), 7.36 (t, *J* = 7.8 Hz, 1H), 7.12 (dt, *J* = 7.6, 1.5 Hz, 1H), 6.46 (dd, *J* = 2.5, 1.8 Hz, 1H), 2.68 (h, *J* = 7.1 Hz, 1H), 1.70 – 1.58 (m, 2H), 1.28 (d, *J* = 6.9 Hz, 3H), 0.85 (t, *J* = 7.4 Hz, 3H).

^{13}C NMR (101 MHz, $CDCl_3$) δ 149.6 (C_q), 141.0 (CH), 140.4 (C_q), 129.4 (CH), 127.0 (CH), 125.4 (CH), 118.4 (CH), 116.8 (CH), 107.5 (CH), 41.9 (CH), 31.2 (CH_2), 21.9 (CH_3), 12.4 (CH_3).

IR (ATR): ṽ = 2960, 1609, 1591, 1519, 1392, 1042, 945, 787, 745 cm^{-1}.

HR-MS (ESI): m/z calcd for $C_{13}H_{16}N_2$ $[M+H]^+$: 201.1386, found: 201.1392.

The general procedure **C** was followed using 1-phenyl-1*H*-pyrazole (**50b**) (57.7 mg, 0.4 mmol) and 2-bromobutane (**25**) (131.0 µL, 1.20 mmol), and $[Ru(t\text{-}BuCN)_5(H_2O)](BF_4)_2$ (**7**) (28.3 mg, 10 mol%) in 1,4-dioxane (2.0 mL). The reaction was stirred at 70 °C for 40 h under N_2 atmosphere. Crude ^{1}H NMR using mesitylene (0.40 mmol) as internal standard gave *mono*-alkylated product **64a** (8%).

The general procedure **D** was followed using 1-phenyl-1*H*-pyrazole (**50b**) (57.7 mg, 0.4 mmol) and 2-bromobutane (**25**) (131.0 µL, 1.20 mmol), and $[Ru(t\text{-}BuCN)_5(H_2O)](BF_4)_2$ (**7**) (28.3 mg, 10 mol%) in NMP (2.0 mL). The reaction was stirred at 70 °C for 48 h under N_2 atmosphere. Crude ^{1}H NMR using mesitylene (0.40 mmol) as internal standard gave *mono*-alkylated product **64a** (61%).

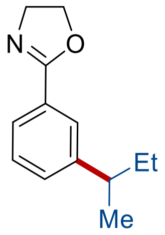

(*R*)-2-(3-(sec-butyl)phenyl)-4,5-dihydrooxazole (65a)

The general procedure **C** was followed using 2-phenyl-4,5-dihydrooxazole (**50c**) (29.5 mg, 0.2 mmol) and 2-bromobutane (**25**) (65.5 µL, 0.6 mmol), and **15** (5.0 mg, 5 mol%) in 1,4-dioxane (1.0 mL). The reaction was stirred at 62 °C for 48 h under N_2 atmosphere. Purification by column chromatography (*n*-hexane/EtOAc = 3:1) yielded **65** (**65a**: 30.1 mg, 74%; **65b**: trace), as a colorless oil.

65a:

^{1}H NMR (300 MHz, $CDCl_3$) δ 7.78 (d, *J* = 1.7 Hz, 1H), 7.75 (dt, *J* = 7.1, 1.8 Hz, 1H), 7.39 – 7.24 (m, 2H), 4.43 (t, *J* = 9.5 Hz, 2H), 4.06 (t, *J* = 9.5 Hz, 2H), 2.64 (h, *J* = 7.0 Hz, 1H), 1.61 (p, *J* = 7.3 Hz, 2H), 1.25 (d, *J* = 7.0 Hz, 3H), 0.81 (t, *J* = 7.4 Hz, 3H).

^{13}C NMR (75 MHz, $CDCl_3$) δ 165.1 (C_q), 148.0 (C_q), 130.4 (CH), 128.4 (CH), 127.8 (C_q), 127.0 (CH), 125.9 (CH), 67.7 (CH_2), 55.0 (CH_2), 41.8 (CH), 31.2 (CH_2), 21.9 (CH_3), 12.4 (CH_3).

IR (ATR): ṽ = 2951, 2835, 1608, 1580, 1457, 1441, 1248, 1031, 830, 747 cm^{-1}.

HR-MS (ESI): m/z calcd for $C_{13}H_{17}N_1O_1$ $[M+H]^+$: 204.1383, found: 204.1393.

The general procedure **C** was followed using 2-phenyl-4,5-dihydrooxazole (**50c**) (29.5 mg, 0.2 mmol) and 2-bromobutane (**25**) (65.5 µL, 0.6 mmol), and [Ru(*p*-cymene)OAc_2] (**3**) (7.1 mg, 10 mol%) in 1,4-dioxane (1.0 mL). The reaction was stirred at 62 °C for 48 h under N_2 atmosphere. Crude ^{1}H NMR using mesitylene (0.20 mmol) as internal standard gave *mono*-alkylated product **65a** (27%) and **65b** (trace).

The general procedure **C** was followed using 2-phenyl-4,5-dihydrooxazole (**50c**) (29.5 mg, 0.2 mmol) and 2-bromobutane (**25**) (65.5 µL, 0.6 mmol), and $[Ru(t\text{-}BuCN)_5(H_2O)](BF_4)_2$ (**7**) (14.0 mg, 10 mol%) in 1,4-dioxane (1.0 mL). The reaction was stirred at 62 °C for 48 h under N_2 atmosphere. Crude ^{1}H NMR using mesitylene (0.20 mmol) as internal standard gave *mono*-alkylated product **65a** (23%) and **65b** (trace).

The general procedure **D** was followed using 2-phenyl-4,5-dihydrooxazole (**50c**) (58.9 mg, 0.4 mmol) and 2-bromobutane (**25**) (131.0 µL, 1.20 mmol), and $[Ru(t\text{-}BuCN)_5(H_2O)](BF_4)_2$ (**7**) (28.3 mg, 10 mol%) in 1,4-dioxane (2.0 mL). The reaction was stirred at 62 °C for 48 h under N_2 atmosphere. Crude ^{1}H NMR using mesitylene (0.40 mmol) as internal standard gave *mono*-alkylated product **65a** (trace) and **65b** (trace).

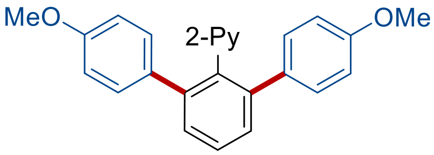

2-(4,4''-dimethoxy-[1,1':3',1''-terphenyl]-2'-yl)pyridine (68)

The general procedure **A** was followed using 2-phenylpyridine (**8**) (62.1 mg, 0.4 mmol) and 1-bromo-4-methoxybenzene (**67a**) (149.7 µL, 1.20 mmol), and [Ru(*p*-cymene)OAc$_2$] (**3**) (14.1 mg, 10 mol%) in NMP (2.0 mL). The reaction was stirred at 40 °C for 20 h under N_2 atmosphere. Purification by column chromatography (*n*-hexane/EtOAc = 10:1) yielded **68** (139.6 mg, 95%), as a colorless solid. The characterization data is in accordance with those reported in the literature.[22]

^{1}H NMR (400 MHz, $CDCl_3$) δ 8.35 (ddd, *J* = 4.9, 1.9, 1.0 Hz, 1H), 7.48 (dd, *J* = 8.5, 6.7 Hz, 1H), 7.42 – 7.38 (m, 2H), 7.33 (td, *J* = 7.7, 1.8 Hz, 1H), 7.03 – 6.98 (m, 4H), 6.93 (ddd, *J* = 7.5, 4.9, 1.2 Hz, 1H), 6.88 (dt, *J* = 7.8, 1.1 Hz, 1H), 6.72 – 6.66 (m, 4H), 3.74 (s, 6H).

^{13}C NMR (101 MHz, $CDCl_3$) δ 159.4 (C_q), 158.2 (C_q), 148.7 (CH), 141.5 (C_q), 138.5 (C_q), 135.2 (CH), 134.2 (C_q), 134.2 (C_q), 130.8 (CH), 129.3 (CH), 128.3 (CH), 126.9 (CH), 120.9 (CH), 113.2 (CH), 55.3 (CH_3).

IR (ATR): ṽ = 2931, 2835, 1608, 1580, 1511, 1457, 1441, 1248, 1230, 1184, 1031, 830, 805, 742 cm^{-1}.

HR-MS (ESI): m/z calcd for $C_{25}H_{21}N_1O_2$ $[M+H]^+$: 368.1645, found: 368.1656.

The general procedure **A** was followed using 2-phenylpyridine (**8**) (62.1 mg, 0.4 mmol) and 1-bromo-4-methoxybenzene (**67a**) (149.7 µL, 1.20 mmol), and [Ru(*t*-BuCN)$_5$(H$_2$O)](BF$_4$)$_2$ (**7**) (28.3 mg, 10 mol%) in NMP (2.0 mL). The reaction was stirred at 40 °C for 20 h under N_2 atmosphere. Crude ^{1}H NMR using mesitylene (0.40 mmol) as internal standard gave product **68** (96%).

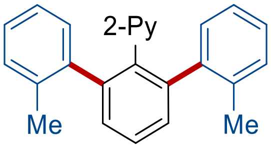

2-(2,2''-dimethyl-[1,1':3',1''-terphenyl]-2'-yl)pyridine (69)

The general procedure **A** was followed using 2-phenylpyridine (**8**) (62.1 mg, 0.4 mmol) and 1-bromo-2-methylbenzene (**67b**) (143.7 µL, 1.20 mmol), and [Ru(*p*-cymene)OAc$_2$] (**3**) (14.1 mg, 10 mol%) in NMP (2.0 mL). The reaction was stirred at 50 °C for 20 h under N_2 atmosphere. Purification by column chromatography (*n*-hexane/EtOAc = 20:1) yielded **69** (84.6 mg, 63%), as a colorless solid. The characterization data is in accordance with those reported in the literature.[23]

^{1}H NMR (500 MHz, $CDCl_3$) δ 8.16 (dt, *J* = 4.7, 2.0 Hz, 1H), 7.51 – 7.44 (m, 1H), 7.31 (dd, *J* = 7.6, 2.4 Hz, 2H), 7.19 – 7.10 (m, 2H), 7.08 – 6.95 (m, 7H), 6.76 (dddd, *J* = 11.4, 5.6, 3.0, 1.3 Hz, 2H), 2.10 (s, 3H), 2.03 (s, 3H).

^{13}C NMR (126 MHz, $CDCl_3$) δ 158.6 (C_q), 158.6 (C_q), 148.2 (CH), 141.5 (C_q), 141.4 (C_q), 141.3 (C_q), 139.6 (C_q), 139.5 (C_q), 136.1 (C_q), 135.9 (C_q), 134.3 (CH), 130.8 (CH), 130.4 (CH), 129.5 (CH), 129.5 (CH), 129.2 (CH), 129.2 (CH), 127.6 (CH), 126.9 (CH), 126.9 (CH), 125.7 (CH), 124.9 (CH), 124.9 (CH), 120.7 (CH), 20.6 (CH_3), 20.5 (CH_3).

IR (ATR): ṽ = 3061, 3015, 2925, 1738, 1588, 1562, 1454, 1417, 759, 747 cm^{-1}.

HR-MS (ESI): m/z calcd for $C_{25}H_{21}N_1$ $[M+H]^+$: 336.1747, found: 336.1757.

The general procedure **A** was followed using 2-phenylpyridine (**8**) (62.1 mg, 0.4 mmol) and 1-bromo-2-methylbenzene (**67b**) (143.7 µL, 1.20 mmol), and [Ru(*t*-BuCN)$_5$(H_2O)](BF_4)$_2$ (**7**) (28.3 mg, 10 mol%) in NMP (2.0 mL). The reaction was stirred at 50 °C for 20 h under N_2 atmosphere. Crude ^{1}H NMR using mesitylene (0.40 mmol) as internal standard gave product **69** (94%).

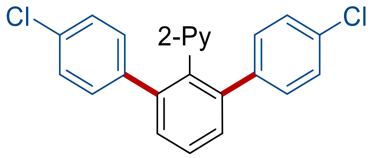

2-(4,4''-dichloro-[1,1':3',1''-terphenyl]-2'-yl)pyridine (70)

The general procedure **A** was followed using 2-phenylpyridine (**8**) (62.1 mg, 0.4 mmol) and 1-bromo-4-chlorobenzene (**67c**) (231.8 mg, 1.20 mmol), and [Ru(*p*-cymene)OAc_2] (**3**) (14.1 mg, 10 mol%) in NMP (2.0 mL). The reaction was stirred at 45 °C for 20 h under N_2 atmosphere. Purification by column chromatography (*n*-hexane/EtOAc = 20:1) yielded **70** (147.5 mg, 98%), as a colorless solid. The characterization data is in accordance with those reported in the literature.[23]

^{1}H NMR (500 MHz, $CDCl_3$) δ 8.35 (ddd, *J* = 4.9, 1.8, 1.0 Hz, 1H), 7.52 (dd, *J* = 8.3, 7.1 Hz, 1H), 7.42 (dd, *J* = 7.7, 0.6 Hz, 2H), 7.35 (td, *J* = 7.7, 1.8 Hz, 1H), 7.15 – 7.10 (m, 4H), 7.04 – 7.00 (m, 4H), 6.97 (ddd, *J* = 7.6, 4.9, 1.2 Hz, 1H), 6.85 (dt, *J* = 7.8, 1.1 Hz, 1H).

^{13}C NMR (126 MHz, $CDCl_3$) δ 158.5 (C_q), 149.0 (CH), 140.9 (C_q), 140.0 (C_q), 138.6 (C_q), 135.4 (CH), 132.7 (C_q), 131.0 (CH), 129.8 (CH), 128.5 (CH), 128.0 (CH), 126.8 (CH), 121.4 (CH).

IR (ATR): ṽ = 2985, 1737, 1580, 1491, 1452, 1372, 1235, 1088, 1014, 825, 799 cm^{-1}.

HR-MS (ESI): m/z calcd for $C_{23}H_{15}Cl_2N_1$ $[M+H]^+$: 376.0654, found: 376.0670.

The general procedure **A** was followed using 2-phenylpyridine (**8**) (62.1 mg, 0.4 mmol) and 1-bromo-4-chlorobenzene (**67c**) (231.8 mg, 1.20 mmol), and [Ru(*t*-BuCN)$_5$(H_2O)](BF_4)$_2$ (**7**) (28.3 mg, 10 mol%) in NMP (2.0 mL). The reaction was stirred at 45 °C for 20 h under N_2 atmosphere. Crude ^{1}H NMR using mesitylene (0.40 mmol) as internal standard gave product **70** (97%).

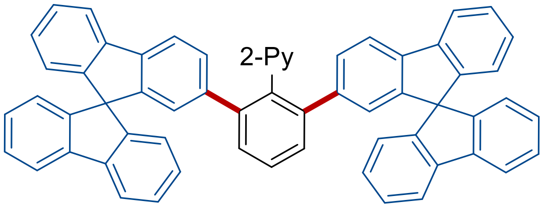

2-(2,6-di(9,9'-spirobi[fluoren]-2-yl)phenyl)pyridine (71)

The general procedure **A** was followed using 2-phenylpyridine (**8**) (31.1 mg, 0.2 mmol) and 2-bromo-9,9'-spirobi[fluorene] (**67d**) (237.2 mg, 0.6 mmol), and [Ru(*p*-cymene)OAc$_2$] (**3**) (7.1 mg, 10 mol%) in NMP (1.0 mL). The reaction was stirred at 62 °C for 24 h under N_2 atmosphere. Purification by column chromatography (*n*-hexane/EtOAc = 20:1) yielded **71** (150.4 mg, 96%), as a colorless liquid.

^{1}H NMR (300 MHz, $CDCl_3$) δ 7.65 – 7.49 (m, 9H), 7.21 – 7.06 (m, 11H), 6.88 (tt, *J* = 7.6, 1.5 Hz, 6H), 6.60 – 6.46 (m, 3H), 6.36 (d, *J* = 7.6 Hz, 4H), 6.28 (d, *J* = 7.8 Hz, 1H), 6.25 – 6.17 (m, 1H), 6.05 (d, *J* = 1.6 Hz, 2H).

^{13}C NMR (75 MHz, $CDCl_3$) δ 157.6 (C_q), 149.1 (C_q), 148.5 (C_q), 148.0 (C_q), 147.5 (CH), 141.6 (C_q), 141.6 (C_q), 141.5 (C_q), 141.2 (C_q), 139.9 (C_q), 138.4 (C_q), 134.0 (CH), 129.3 (CH), 129.0 (CH), 127.9 (CH), 127.8 (CH), 127.7 (CH), 127.5 (CH), 126.0 (CH), 125.7 (CH), 124.0 (CH), 124.0 (CH), 121.2 (CH), 119.9 (CH), 119.8 (CH), 119.4 (CH), 65.7 (C_q).

IR (ATR): ṽ = 3061, 2924, 1602, 1445, 1411, 905, 725, 621 cm^{-1}.

HR-MS (ESI): m/z calcd for $C_{61}H_{37}N_1$ $[M+H]^+$: 784.2999, found: 784.2994.

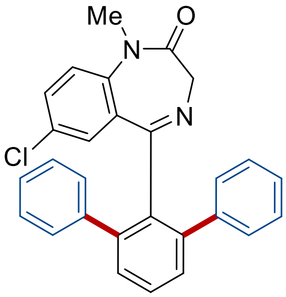

5-([1,1':3',1''-terphenyl]-2'-yl)-7-chloro-1-methyl-1,3-dihydro-2H-benzo[e][1,4]diazepin-2-one (72a)

The general procedure **B** was followed using 7-chloro-1-methyl-5-phenyl-1,3-dihydro-2H-benzo[e][1,4]diazepin-2-one (**27n**) (114.0 mg, 0.4 mmol) and bromobenzene (**67e**) (126 µL, 1.20 mmol), and [RuCl$_2$(benzene)]$_2$ (**15**) (10.0 mg, 5 mol%) in 1,4-dioxane (2.0 mL). The reaction was stirred at 62 °C for 24 h under N_2 atmosphere. Purification by column chromatography (*n*-hexane/EtOAc = 5:1) yielded **72** (*di*-arylated product **72a**: 118.8 mg, 68%; mono-arylated product **72b**: trace), as a colorless solid.

72a:

^{1}H NMR (300 MHz, $CDCl_3$) δ 7.59 – 7.47 (m, 2H), 7.44 – 7.23 (m, 6H), 7.20 (dd, *J* = 8.8, 2.5 Hz, 1H), 7.16 – 7.03 (m, 4H), 7.02 – 6.92 (m, 2H), 6.77 (d, *J* = 8.8 Hz, 1H), 4.46 (d, *J* = 10.8 Hz, 1H), 3.31 (d, *J* = 10.8 Hz, 1H), 2.92 (s, 3H).

^{13}C NMR (75 MHz, $CDCl_3$) δ 169.1 (C_q), 168.4 (C_q), 142.7 (C_q), 142.3 (C_q), 141.6 (C_q), 141.3 (C_q), 141.0 (C_q), 136.8 (C_q), 132.0 (C_q), 131.0 (CH), 130.0 (CH), 129.6 (CH), 129.4 (CH), 129.2 (CH), 129.2 (CH), 128.8 (C_q), 128.7 (CH), 128.0 (CH), 127.8 (CH), 127.0 (CH), 126.6 (CH), 121.8 (CH), 56.2 (CH_2), 35.3 (CH_3).

IR (ATR): ṽ = 3057, 1671, 1617, 1481, 1399, 1342, 1317, 1130, 909, 729 cm^{-1}.

HR-MS (ESI): m/z calcd for $C_{28}H_{21}Cl_1N_2O_1$ $[M+H]^+$: 437.1415, found: 437.1429.

72b:

^{1}H NMR (300 MHz, $CDCl_3$) δ 7.76 – 7.70 (m, 1H), 7.59 – 7.46 (m, 2H), 7.31 – 7.27 (m, 1H), 7.14 (dd, *J* = 8.8, 2.5 Hz, 1H), 7.08 (ddt, *J* = 5.4, 3.9, 2.1 Hz, 3H), 7.00 – 6.93 (m, 2H), 6.79 (dd, *J* = 5.6, 3.1 Hz, 2H), 4.82 (d, *J* = 11.0 Hz, 1H), 3.68 (d, *J* = 10.9 Hz, 1H), 3.12 (s, 3H).

^{13}C NMR (75 MHz, $CDCl_3$) δ 171.4 (C_q), 169.5 (C_q), 142.1 (C_q), 141.6 (C_q), 141.2 (C_q), 138.5 (C_q), 131.6 (C_q), 130.9 (CH), 130.5 (CH), 130.4 (CH), 130.2 (CH), 128.8 (CH), 128.8 (C_q), 128.5 (CH), 128.0 (CH), 127.8 (CH), 126.7 (CH), 121.6 (CH), 56.9 (CH_2), 35.1 (CH_3).

IR (ATR): ṽ = 3058, 2923, 2853, 1674, 1612, 1484, 1401, 1319, 1130, 1075, 818, 751 cm^{-1}.

HR-MS (ESI): m/z calcd for $C_{22}H_{17}Cl_1N_2O_1$ $[M+H]^+$: 361.1102, found: 361.1100.

The general procedure **B** was followed using 7-chloro-1-methyl-5-phenyl-1,3-dihydro-2H-benzo[e][1,4]diazepin-2-one (**27n**) (114.0 mg, 0.4 mmol), bromobenzene (**67e**) (126 µL, 1.20 mmol), and [Ru(*p*-cymene)OAc_2] (**3**) (14.1 mg, 10 mol%) in 1,4-dioxane (2.0 mL). The reaction was stirred at 62 °C for 24 h under N_2 atmosphere. Crude ^{1}H NMR using mesitylene (0.40 mmol) as internal standard gave *di*-arylated product **72a** (7%) and *mono*-arylated product **72b** (34%).

The general procedure **B** was followed using 7-chloro-1-methyl-5-phenyl-1,3-dihydro-2H-benzo[e][1,4]diazepin-2-one (**27n**) (114.0 mg, 0.4 mmol), bromobenzene (**67e**) (126 µL, 1.20 mmol), and $[Ru(t\text{-}BuCN)_5(H_2O)](BF_4)_2$ (**7**) (28.3 mg, 10 mol%) in 1,4-dioxane (2.0 mL). The reaction was stirred at 62 °C for 24 h under N_2 atmosphere. Crude ^{1}H NMR using mesitylene (0.40 mmol) as internal standard gave product **72a** (65%) and *mono*-arylated product **72b** (trace).

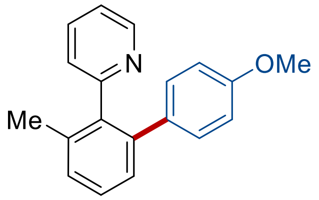

2-(4'-methoxy-3-methyl-[1,1'-biphenyl]-2-yl)pyridine (**73**)

The general procedure **A** was followed using 2-(*o*-tolyl)pyridine (**27a**) (33.9 mg, 0.2 mmol), 1-iodo-4-methoxybenzene (**67f**) (140.4 mg, 0.6 mmol), and [Ru(*p*-cymene)OAc_2] (**3**) (7.1 mg, 10 mol%) in NMP (1.0

mL). The reaction was stirred at 45 °C for 20 h under N_2 atmosphere. Purification by column chromatography (n-hexane/EtOAc = 10:1) yielded **73** (45.1 mg, 82%), as a viscous colorless oil.

^{1}H NMR (300 MHz, $CDCl_3$) δ 8.64 (ddd, *J* = 4.9, 1.9, 1.0 Hz, 1H), 7.46 (td, *J* = 7.7, 1.8 Hz, 1H), 7.38 – 7.31 (m, 1H), 7.30 – 7.22 (m, 2H), 7.09 (ddd, *J* = 7.6, 4.9, 1.2 Hz, 1H), 7.03 – 6.96 (m, 2H), 6.89 (dt, *J* = 7.8, 1.1 Hz, 1H), 6.72 – 6.64 (m, 2H), 3.73 (s, 3H), 2.17 (s, 3H).

^{13}C NMR (75 MHz, $CDCl_3$) δ 159.9 (C_q), 158.2 (C_q), 149.0 (CH), 140.9 (C_q), 139.4 (C_q), 136.8 (C_q), 135.9 (CH), 134.2 (C_q), 130.8 (CH), 129.2 (CH), 128.1 (CH), 127.7 (CH), 125.7 (CH), 121.4 (CH), 113.2 (CH), 55.2 (CH_3), 20.6 (CH_3).

IR (ATR): ṽ = 3057, 2931, 2834, 1609, 1511, 1460, 1246, 1179, 1031, 834, 795 cm^{-1}.

HR-MS (ESI): m/z calcd for $C_{19}H_{17}N_1O_1$ $[M+H]^+$: 276.1383, found: 276.1383.

The general procedure **A** was followed using 2-(*o*-tolyl)pyridine (**27a**) (33.9 mg, 0.2 mmol), 1-iodo-4-methoxybenzene (**67f**) (140.4 mg, 0.6 mmol), and $[Ru(t\text{-}BuCN)_5(H_2O)](BF_4)_2$ (**7**) (14.2 mg, 10 mol%) in NMP (1.0 mL). The reaction was stirred at 45 °C for 20 h under N_2 atmosphere. Crude ^{1}H NMR using mesitylene (0.20 mmol) as internal standard gave the product **73** (93%).

The general procedure **A** was followed using 2-(*o*-tolyl)pyridine (**27a**) (33.9 mg, 0.2 mmol), 1-bromo-4-methoxybenzene (**67a**) (74.9 µL, 0.6 mmol), and [Ru(*p*-cymene)OAc_2] (**3**) (7.1 mg, 10 mol%) in NMP (1.0 mL). The reaction was stirred at 45 °C for 20 h under N_2 atmosphere. Crude ^{1}H NMR using mesitylene (0.20 mmol) as internal standard gave the product **73** (77%).

The general procedure **A** was followed using 2-(*o*-tolyl)pyridine (**27a**) (33.9 mg, 0.2 mmol), 1-bromo-4-methoxybenzene (**67a**) (74.9 µL, 0.6 mmol), and $[Ru(t\text{-}BuCN)_5(H_2O)](BF_4)_2$ (**7**) (14.2 mg, 10 mol%) in NMP (1.0 mL). The reaction was stirred at 45 °C for 20 h under N_2 atmosphere. Crude ^{1}H NMR using mesitylene (0.20 mmol) as internal standard gave the product **73** (91%).

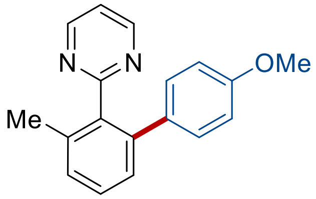

2-(4'-methoxy-3-methyl-[1,1'-biphenyl]-2-yl)pyrimidine (**74**)

The general procedure **A** was followed using 2-(*o*-tolyl)pyrimidine (**27i**) (68.1 mg, 0.4 mmol), 1-iodo-4-methoxybenzene (**67f**) (280.8 mg, 1.20 mmol), and [Ru(*p*-cymene)OAc_2] (**3**) (14.1 mg, 10 mol%) in NMP (2.0 mL). The reaction was stirred at 45 °C for 20 h under N_2 atmosphere. Purification by column chromatography (n-hexane/EtOAc = 10:1) yielded **74** (106.0 mg, 96%), as a white solid.

^{1}H NMR (300 MHz, $CDCl_3$) δ 8.67 (d, *J* = 4.9 Hz, 2H), 7.36 (dd, *J* = 8.0, 7.1 Hz, 1H), 7.29 – 7.22 (m, 2H), 7.07 (t, *J* = 4.9 Hz, 1H), 7.05 – 6.99 (m, 2H), 6.73 – 6.65 (m, 2H), 3.72 (s, 3H), 2.19 (s, 3H).

^{13}C NMR (75 MHz, $CDCl_3$) δ 168.5 (C_q), 158.2 (C_q), 156.7 (CH), 140.6 (C_q), 138.4 (C_q), 136.0 (C_q), 134.1 (C_q), 130.2 (CH), 129.1 (CH), 128.6 (CH), 127.7 (CH), 118.5 (CH), 113.3 (CH), 55.2 (CH_3), 20.1 (CH_3).

IR (ATR): ṽ = 2958, 2930, 2836, 1736, 1554, 1512, 1402, 1243, 1178, 1030, 909, 787 cm^{-1}.

HR-MS (ESI): m/z calcd for $C_{18}H_{16}N_2O_1$ $[M+H]^+$: 277.1335, found: 277.1330.

The general procedure **A** was followed using 2-(*o*-tolyl)pyrimidine (**27i**) (68.1 mg, 0.4 mmol), 1-iodo-4-methoxybenzene (**67f**) (280.8 mg, 1.20 mmol), and $[Ru(t\text{-}BuCN)_5(H_2O)](BF_4)_2$ (**7**) (28.3 mg, 10 mol%) in NMP (2.0 mL). The reaction was stirred at 45 °C for 20 h under N_2 atmosphere. Crude ^{1}H NMR using mesitylene (0.40 mmol) as internal standard gave the product **74** (96%).

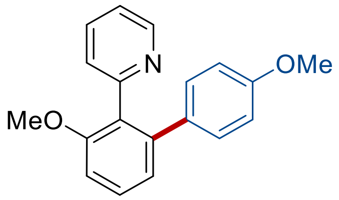

2-(3,4'-dimethoxy-[1,1'-biphenyl]-2-yl)pyridine (**75**)

The general procedure **A** was followed using 2-(2-methoxyphenyl)pyridine (**66a**) (74.1 mg, 0.4 mmol), 1-iodo-4-methoxybenzene (**67f**) (280.8 mg, 1.20 mmol), and [Ru(*p*-cymene)OAc_2] (**3**) (14.1 mg, 10 mol%) in NMP (2.0 mL). The reaction was stirred at 45 °C for 20 h under N_2 atmosphere. Purification by column chromatography (n-hexane/EtOAc = 10:1) yielded **75** (106.4 mg, 92%), as a white solid.

^{1}H NMR (300 MHz, $CDCl_3$) δ 8.60 (ddd, *J* = 4.9, 1.9, 1.0 Hz, 1H), 7.48 (td, *J* = 7.7, 1.8 Hz, 1H), 7.43 – 7.35 (m, 1H), 7.11 – 6.93 (m, 6H), 6.72 – 6.64 (m, 2H), 3.76 (s, 3H), 3.71 (s, 3H).

^{13}C NMR (75 MHz, $CDCl_3$) δ 158.2 (C_q), 157.3 (C_q), 157.1 (C_q), 148.8 (CH), 142.4 (C_q), 135.5 (CH), 133.5 (C_q), 130.6 (CH), 129.1 (CH), 129.0 (C_q), 126.3 (CH), 122.5 (CH), 121.3 (CH), 113.1 (CH), 109.7 (CH), 55.9 (CH_3), 55.1 (CH_3).

IR (ATR): ṽ = 3061, 2935, 2835, 1735, 1584, 1513, 1464, 1291, 1239, 1174, 1122, 1021, 833, 797 cm^{-1}.

HR-MS (ESI): m/z calcd for $C_{19}H_{17}N_1O_2$ $[M+H]^+$: 292.1332, found: 292.1334.

The general procedure **A** was followed using 2-(2-methoxyphenyl)pyridine (**66a**) (74.1 mg, 0.4 mmol), 1-iodo-4-methoxybenzene (**67f**) (280.8 mg, 1.20 mmol), and $[Ru(t\text{-}BuCN)_5(H_2O)](BF_4)_2$ (**7**) (28.3 mg, 10 mol%) in NMP (2.0 mL). The reaction was stirred at 45 °C for 20 h under N_2 atmosphere. Crude ^{1}H NMR using mesitylene (0.40 mmol) as internal standard gave the product **75** (96%).

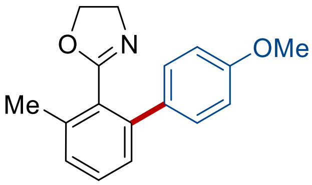

2-(4'-methoxy-3-methyl-[1,1'-biphenyl]-2-yl)-4,5-dihydrooxazole (**76**)

The general procedure **A** was followed using 2-(o-tolyl)-4,5-dihydrooxazole (**66b**) (64.5 mg, 0.4 mmol), 1-iodo-4-methoxybenzene (**67f**) (280.8 mg, 1.20 mmol), and [Ru(*p*-cymene)OAc_2] (**3**) (14.1 mg, 10 mol%) in NMP (2.0 mL). The reaction was stirred at 45 °C for 20 h under N_2 atmosphere. Purification by column chromatography (n-hexane/EtOAc = 10:1) yielded **76** (106.4 mg, 92%), as a colorless liquid.

^{1}H NMR (300 MHz, $CDCl_3$) δ 7.40 – 7.30 (m, 3H), 7.23 – 7.15 (m, 2H), 6.95 – 6.87 (m, 2H), 4.16 (td, *J* = 9.4, 1.0 Hz, 2H), 3.88 (td, *J* = 9.4, 1.0 Hz, 2H), 3.83 (s, 3H), 2.41 (s, 3H).

^{13}C NMR (75 MHz, $CDCl_3$) δ 164.7 (C_q), 158.9 (C_q), 141.7 (C_q), 137.5 (C_q), 133.8 (C_q), 129.6 (CH), 129.5 (CH), 128.7 (CH), 128.2 (C_q), 127.3 (CH), 113.6 (CH), 67.3 (CH_2), 55.3 (CH_3), 55.2 (CH_3), 19.9 (CH_2).

IR (ATR): ṽ = 2959, 2930, 2835, 1662, 1610, 1512, 1460, 1246, 1178, 1032, 936 cm^{-1}.

HR-MS (ESI): m/z calcd for $C_{17}H_{17}N_1O_2$ $[M+H]^+$: 268.1332, found: 268.1335.

The general procedure **A** was followed using 2-(o-tolyl)-4,5-dihydrooxazole (**66b**) (64.5 mg, 0.4 mmol), 1-iodo-4-methoxybenzene (**67f**) (280.8 mg, 1.20 mmol), and [Ru(*t*-BuCN)$_5$(H_2O)](BF_4)$_2$ (**7**) (28.3 mg, 10 mol%) in NMP (2.0 mL). The reaction was stirred at 45 °C for 20 h under N_2 atmosphere. Crude ^{1}H NMR using mesitylene (0.40 mmol) as internal standard gave the product **76** (91%).

7 NMR Spectrum

^{1}H NMR of **10a**, 300 MHz, $CDCl_3$, 25 °C.

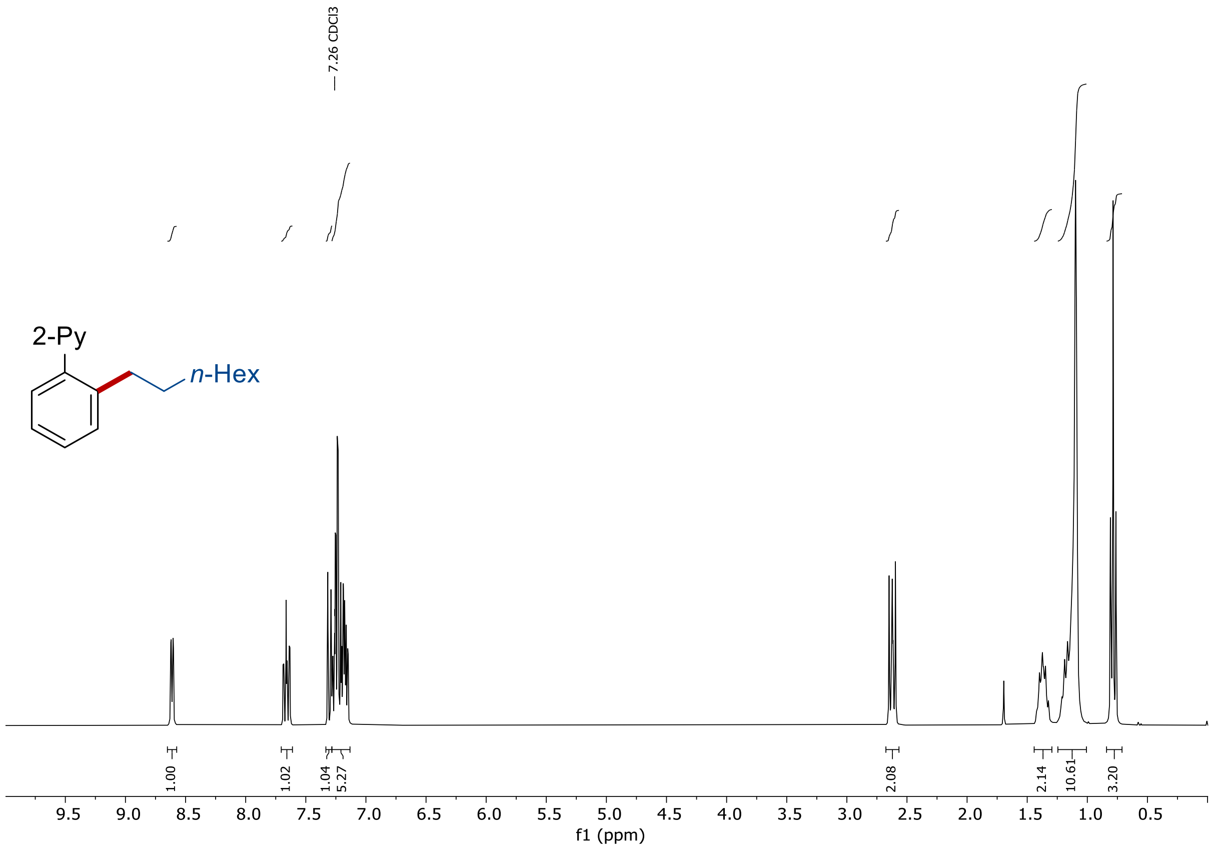

2-Py
n-Hex
— 7.26 CDCl3
1.00
1.02
1.04
5.27
2.08
2.14
10.61
3.20
9.5
9.0
8.5
8.0
7.5
7.0
6.5
6.0
5.5
5.0
4.5
4.0
3.5
3.0
2.5
2.0
1.5
1.0
0.5
f1 (ppm)

^{13}C NMR of **10a**, 75 MHz, $CDCl_3$, 25 °C.

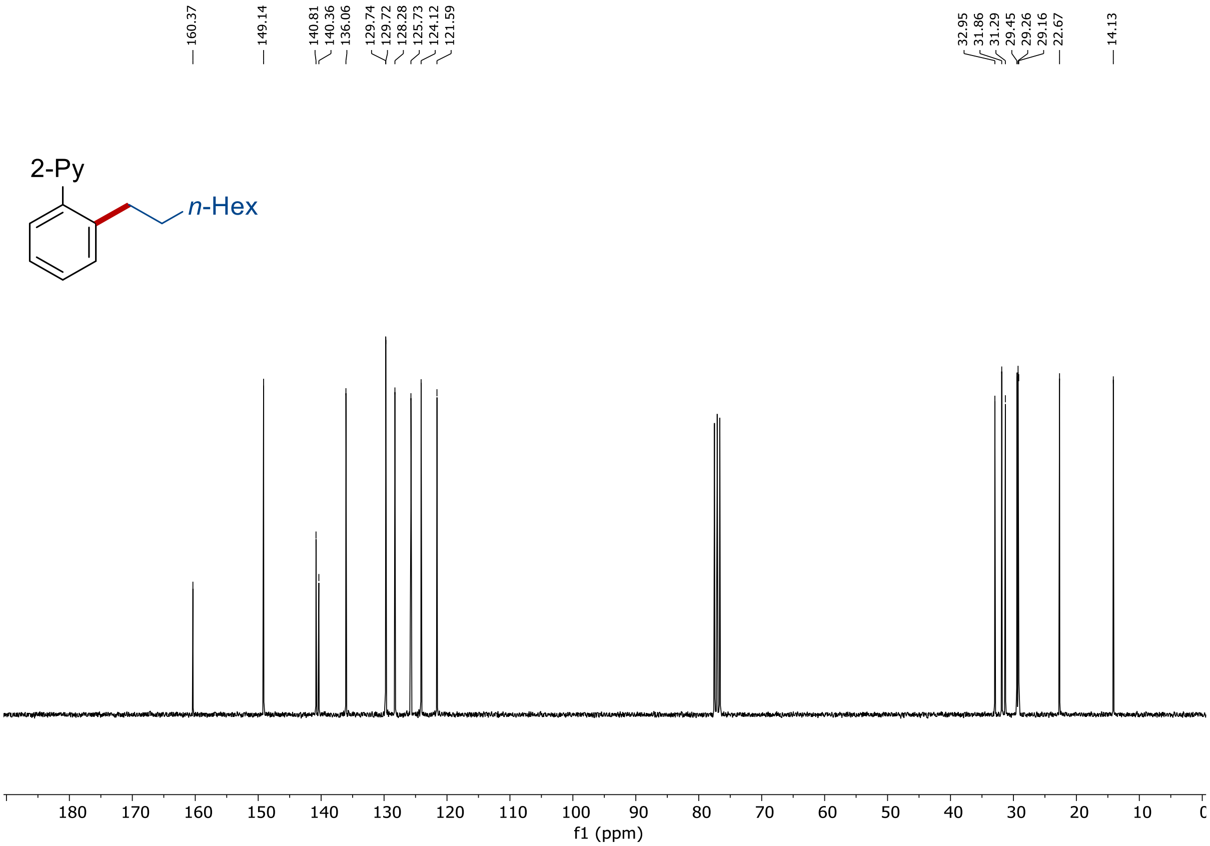

^{1}H NMR of **10b**, 300 MHz, $CDCl_3$, 25 °C.

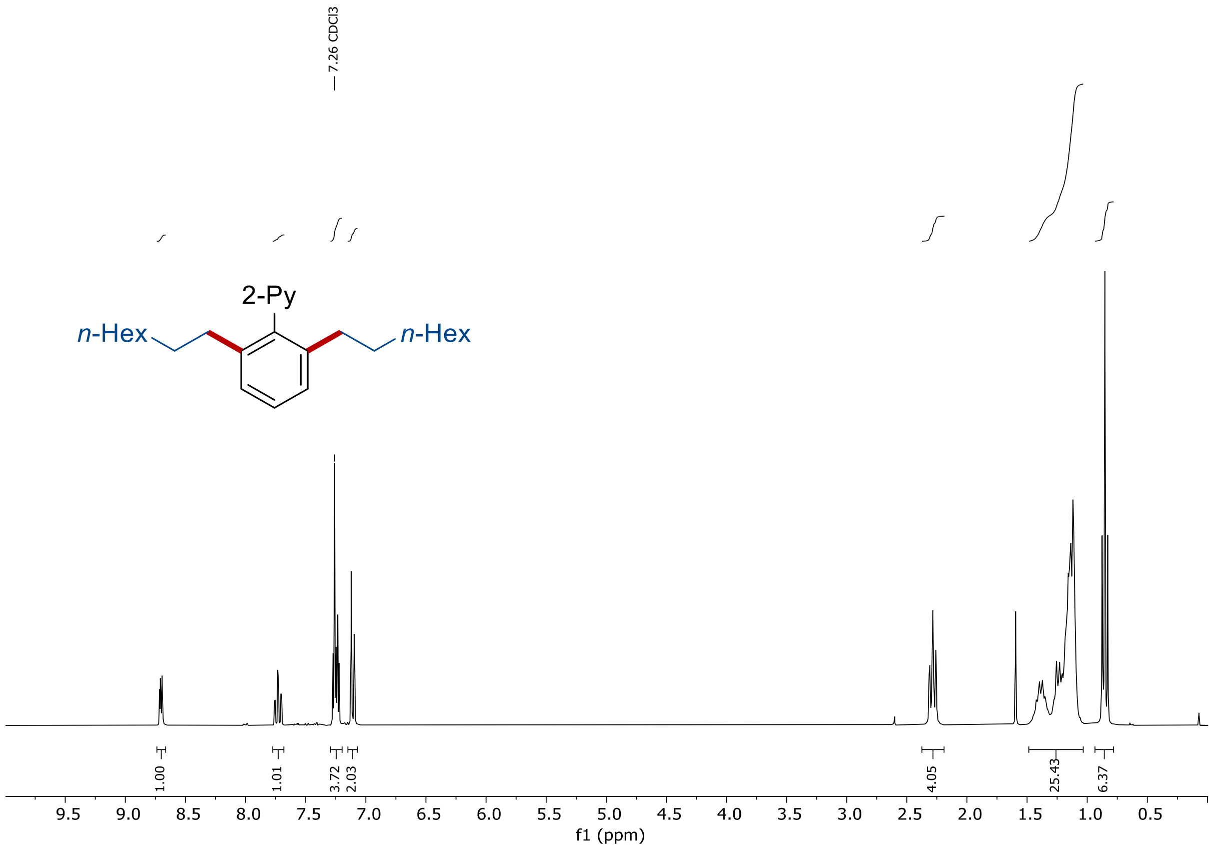

^{13}C NMR of **10b**, 75 MHz, $CDCl_3$, 25 °C.

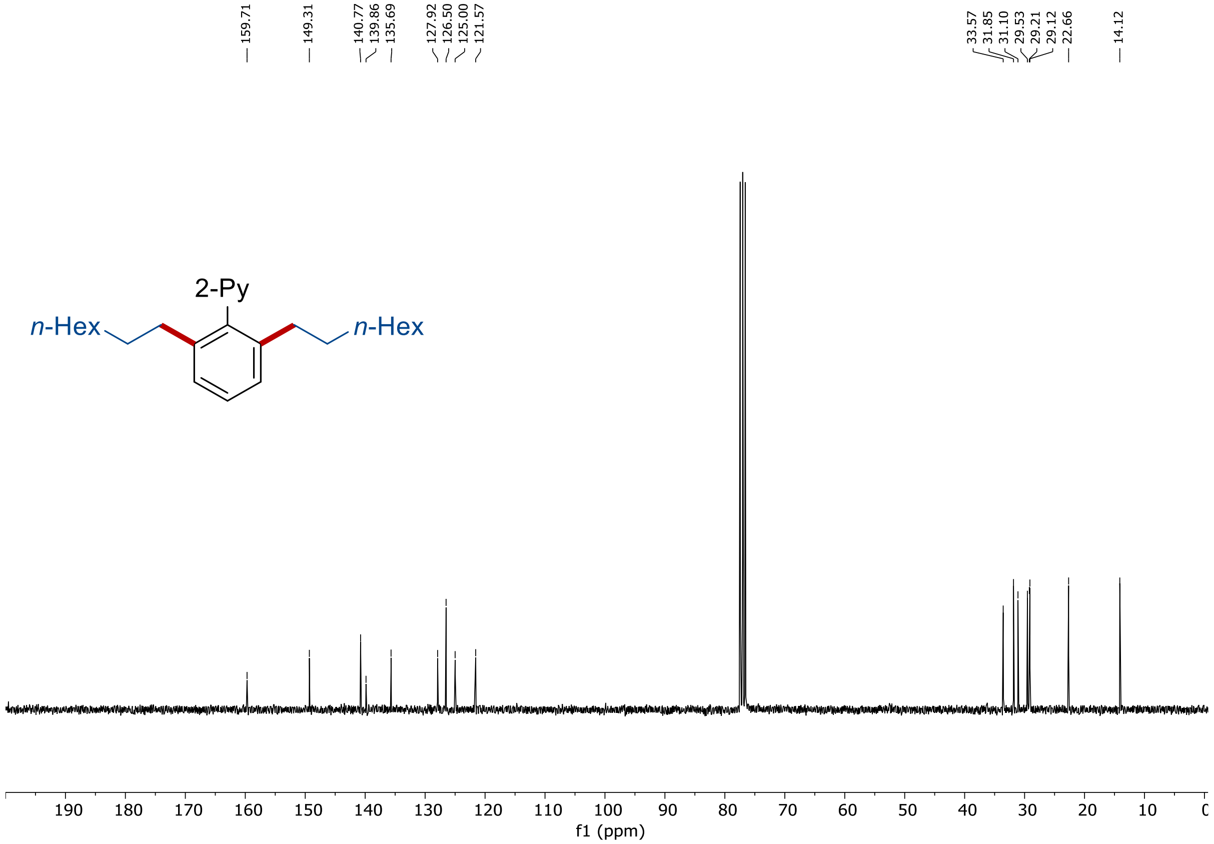

^{1}H NMR of **10a-D₅**, 300 MHz, CDCl₃, 25 °C.

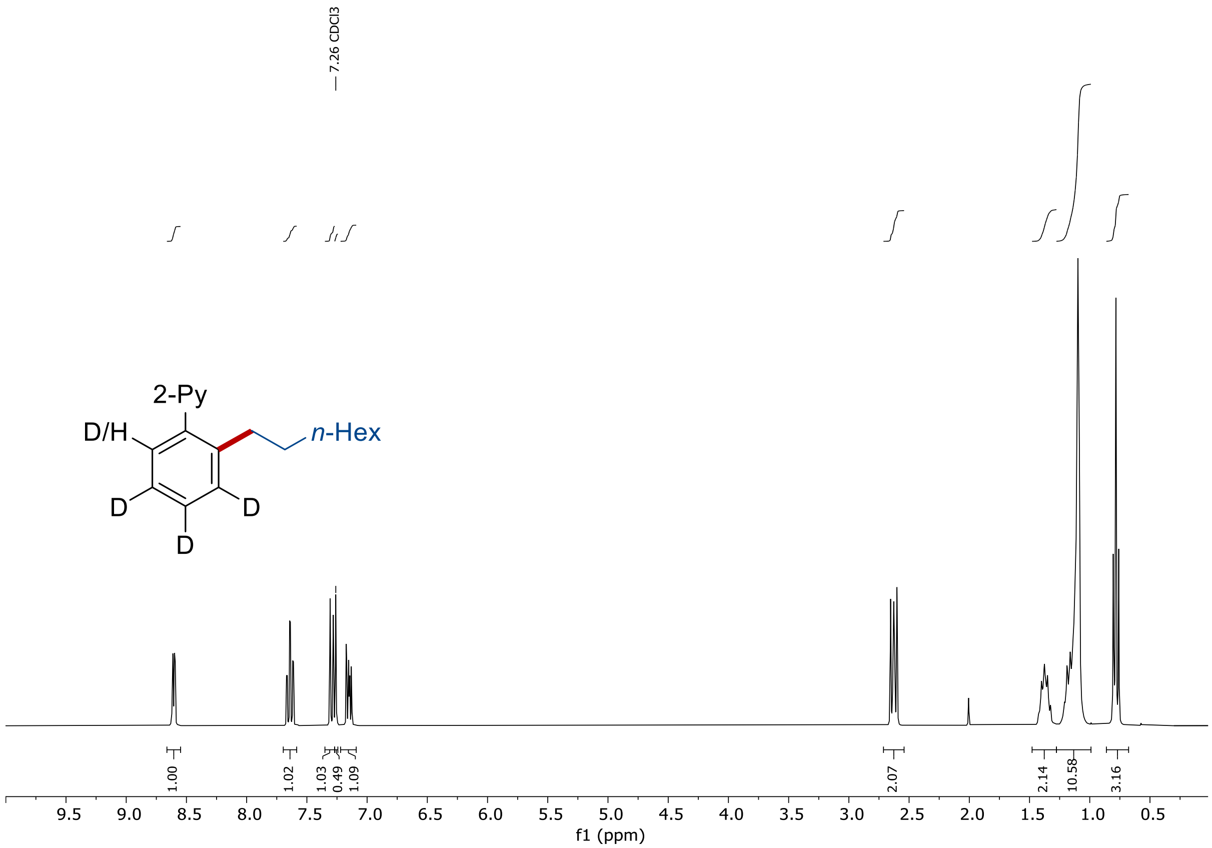

^{13}C NMR of **10a-D₅**, 75 MHz, CDCl₃, 25 °C.

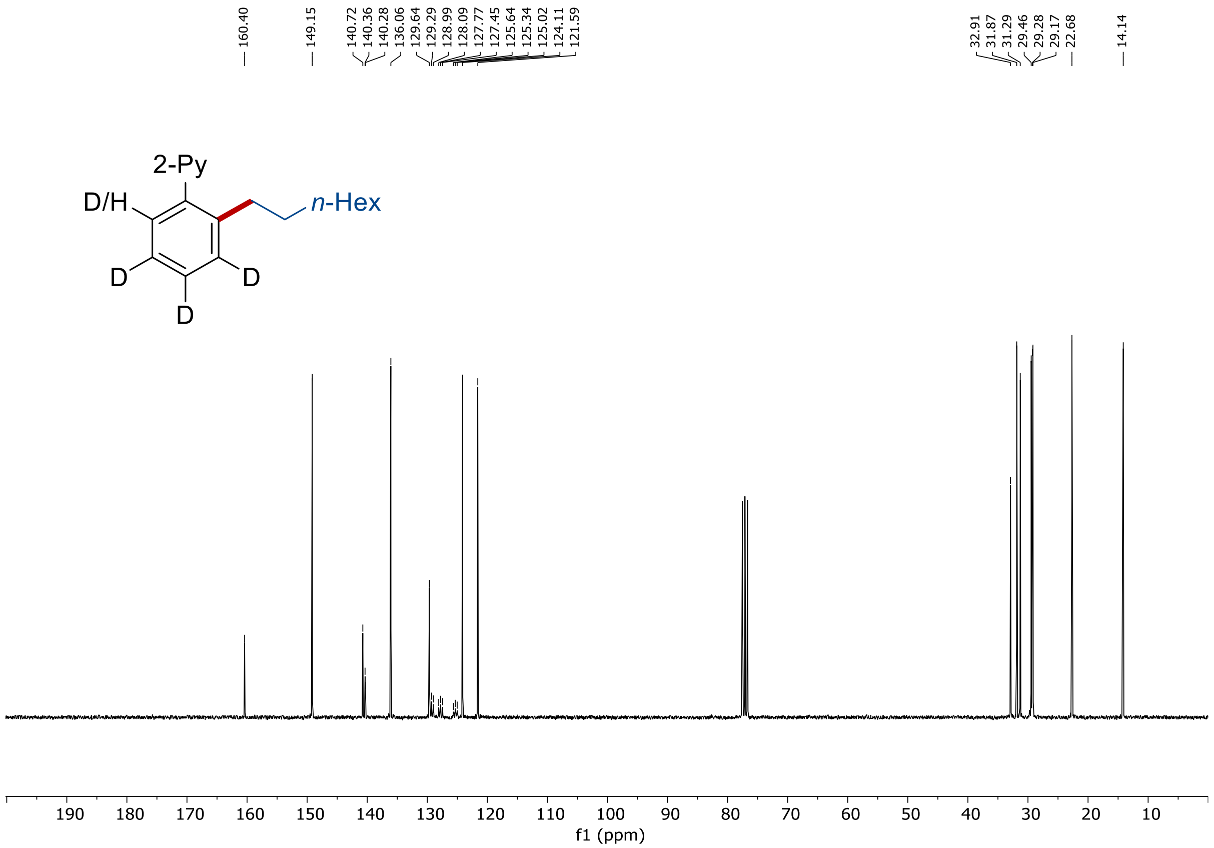

^{1}H NMR of **10b-D$_3$**, 300 MHz, $CDCl_3$, 25 °C.

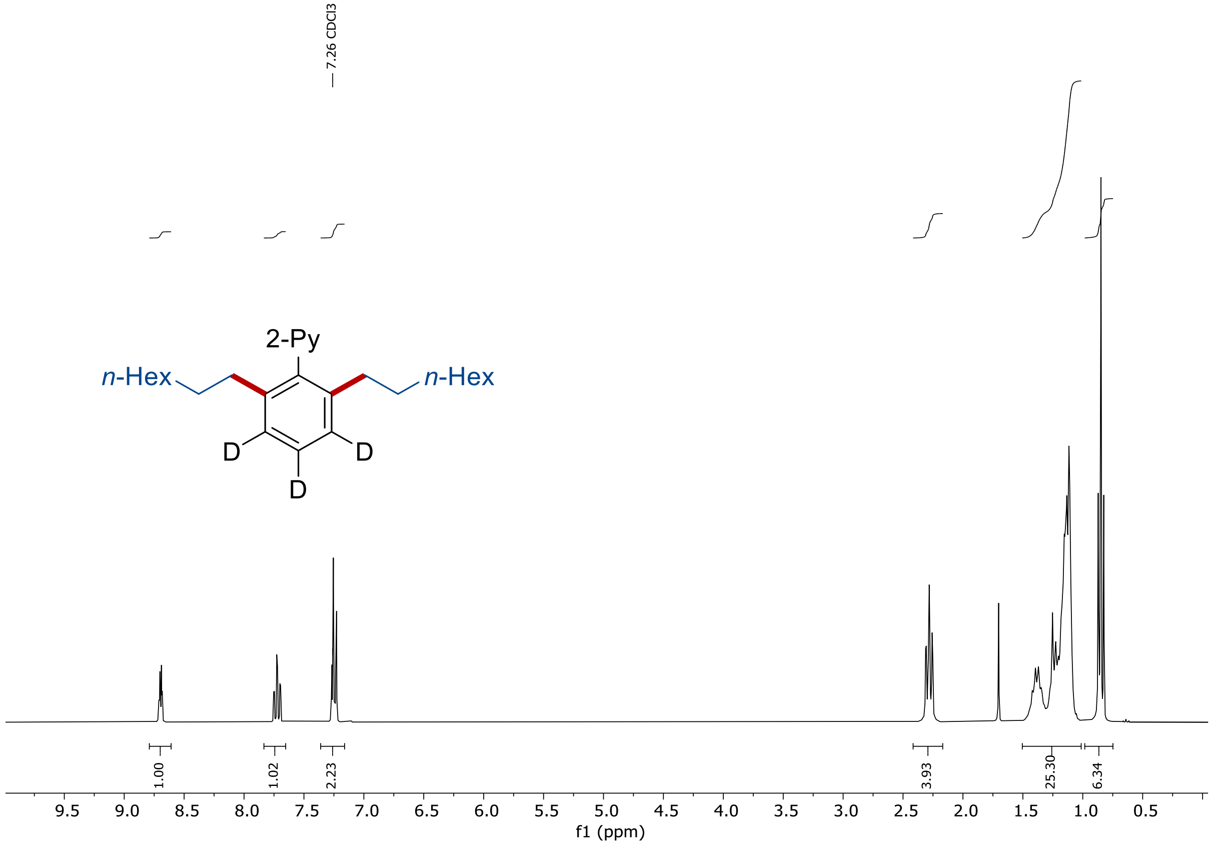

^{13}C NMR of **10b-D$_3$**, 75 MHz, $CDCl_3$, 25 °C.

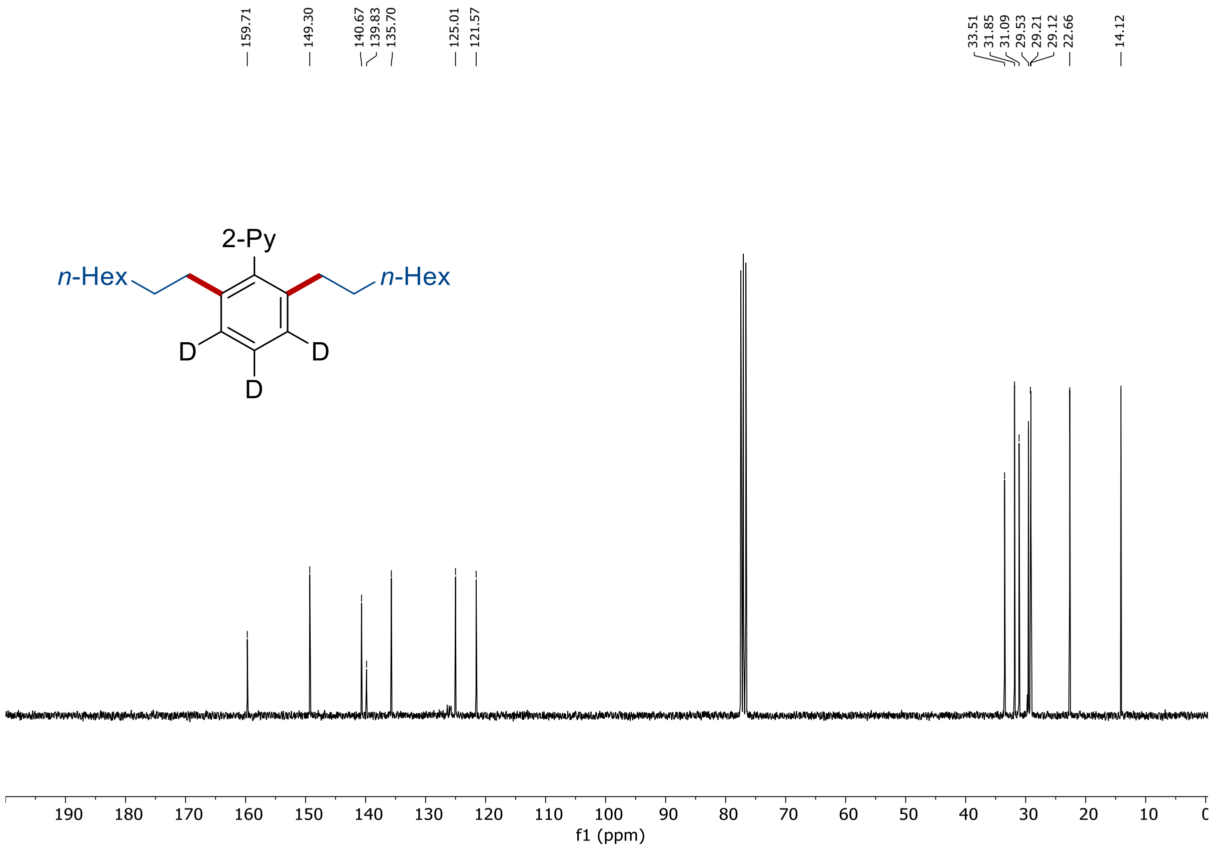

^{1}H NMR of **29**, 400 MHz, $CDCl_3$, 25 °C.

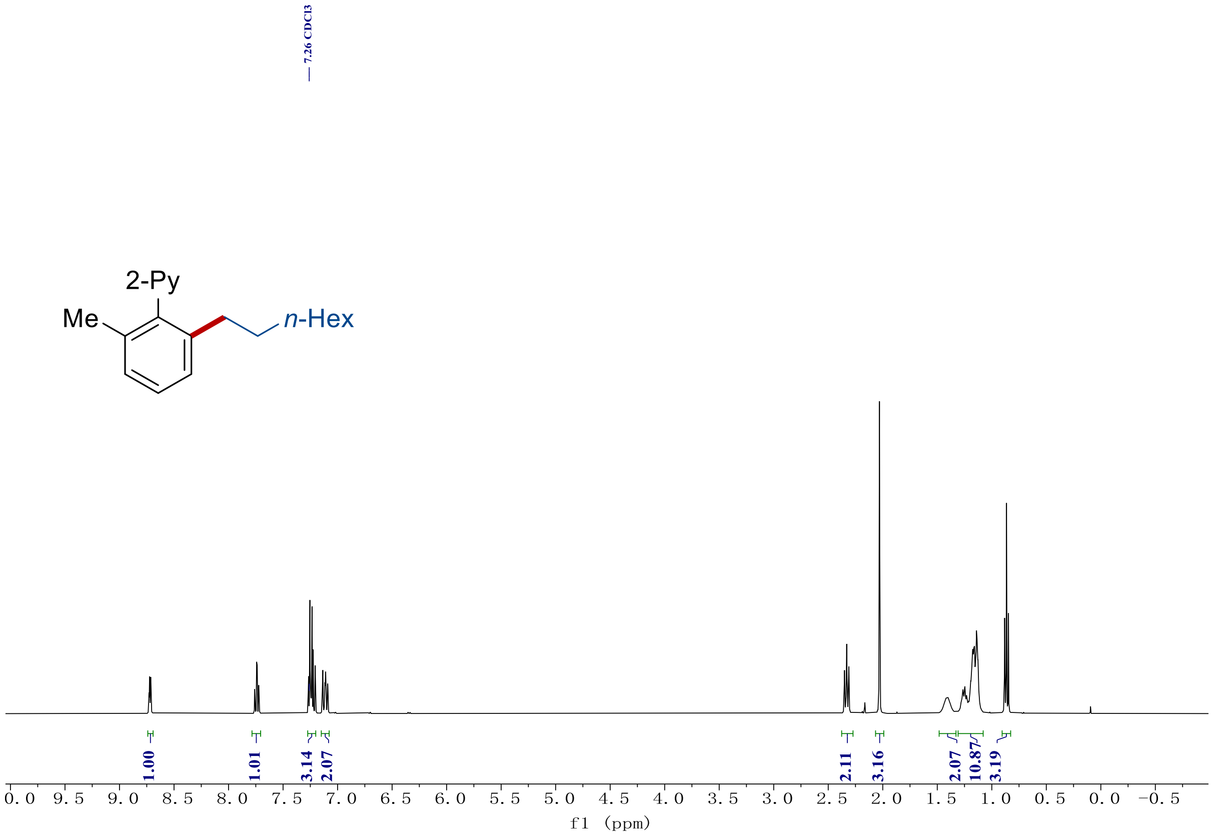

^{13}C NMR of **29**, 101 MHz, $CDCl_3$, 25 °C.

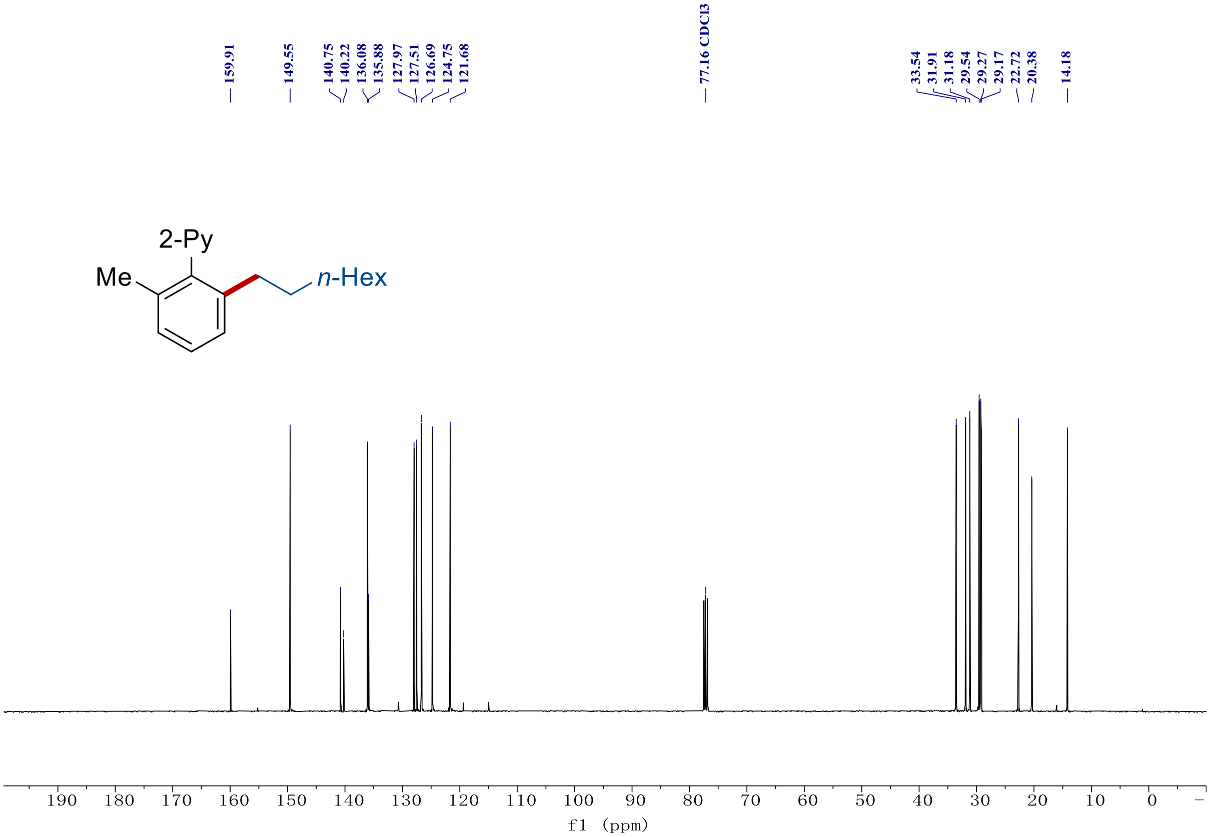

^{1}H NMR of **30a**, 400 MHz, $CDCl_3$, 25 °C.

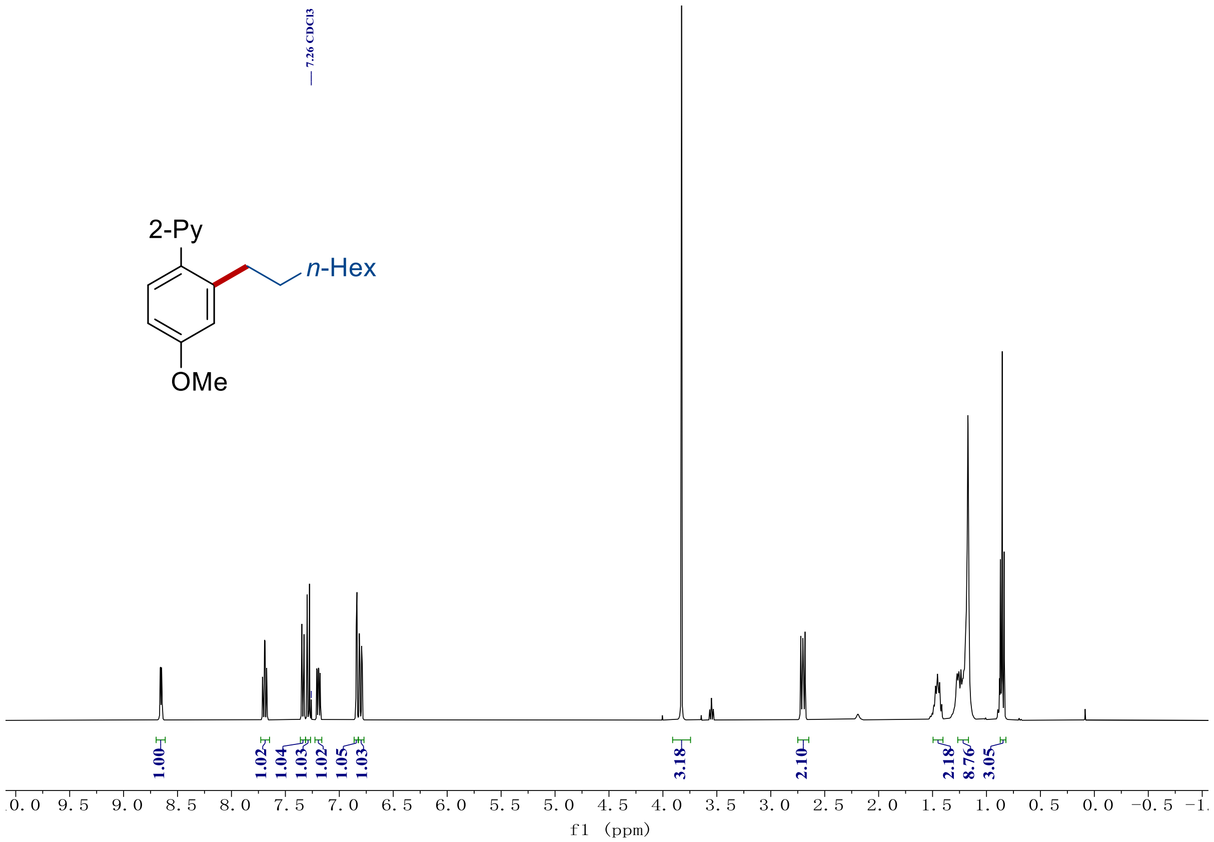

^{13}C NMR of **30a**, 101 MHz, $CDCl_3$, 25 °C.

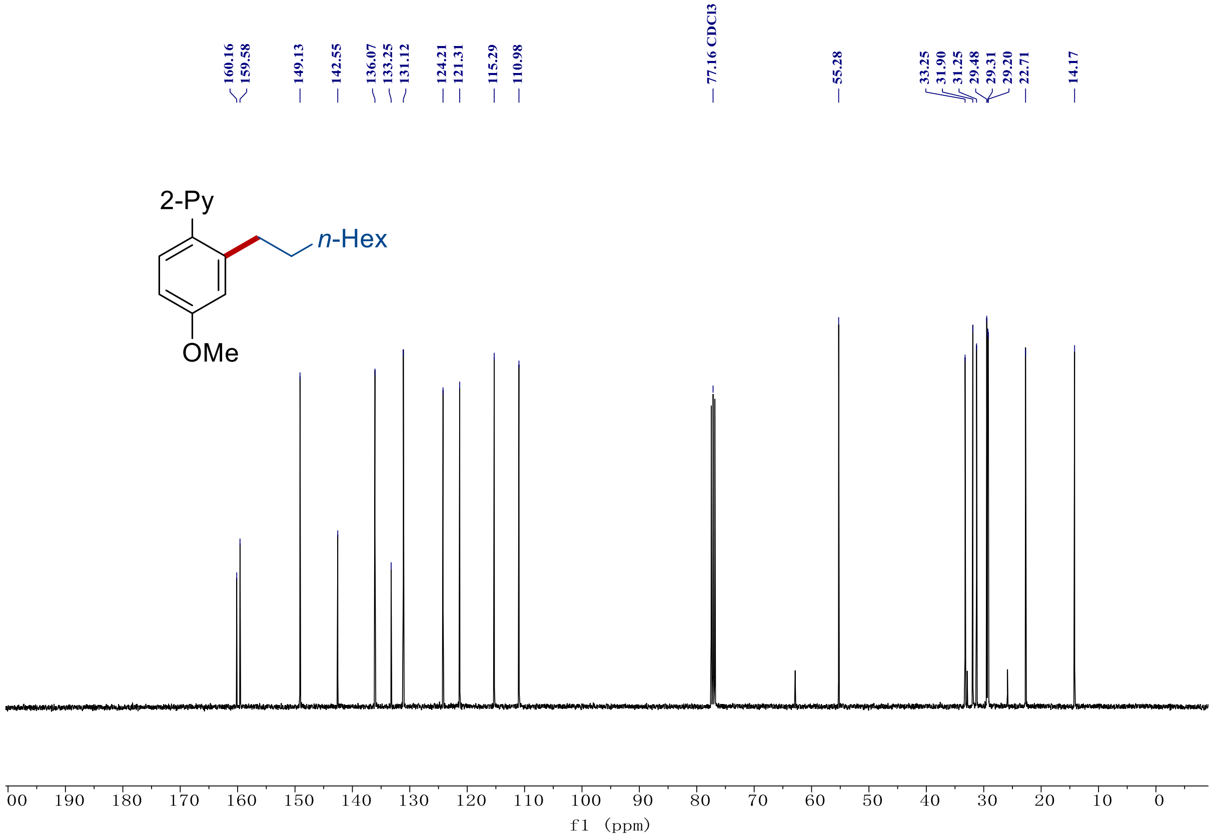

^{1}H NMR of **30b**, 400 MHz, $CDCl_3$, 25 °C.

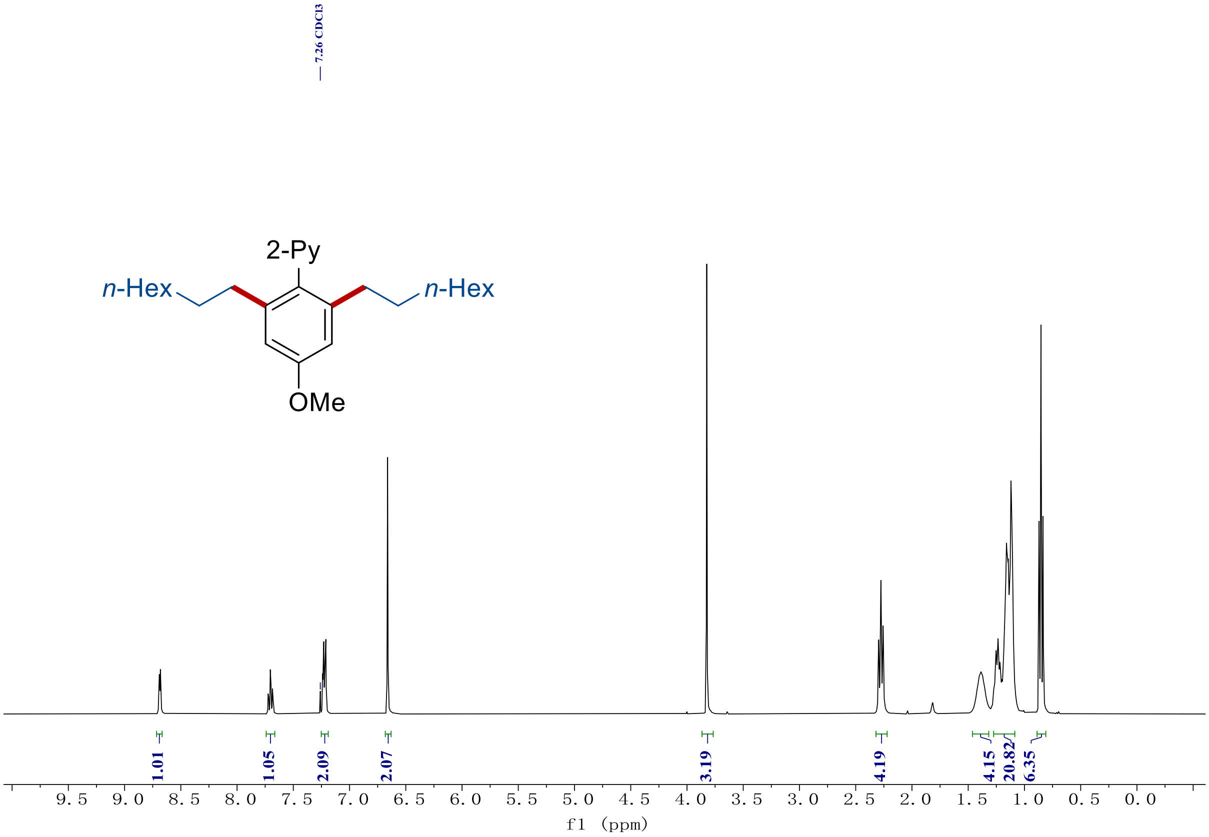

^{13}C NMR of **30b**, 101 MHz, $CDCl_3$, 25 °C.

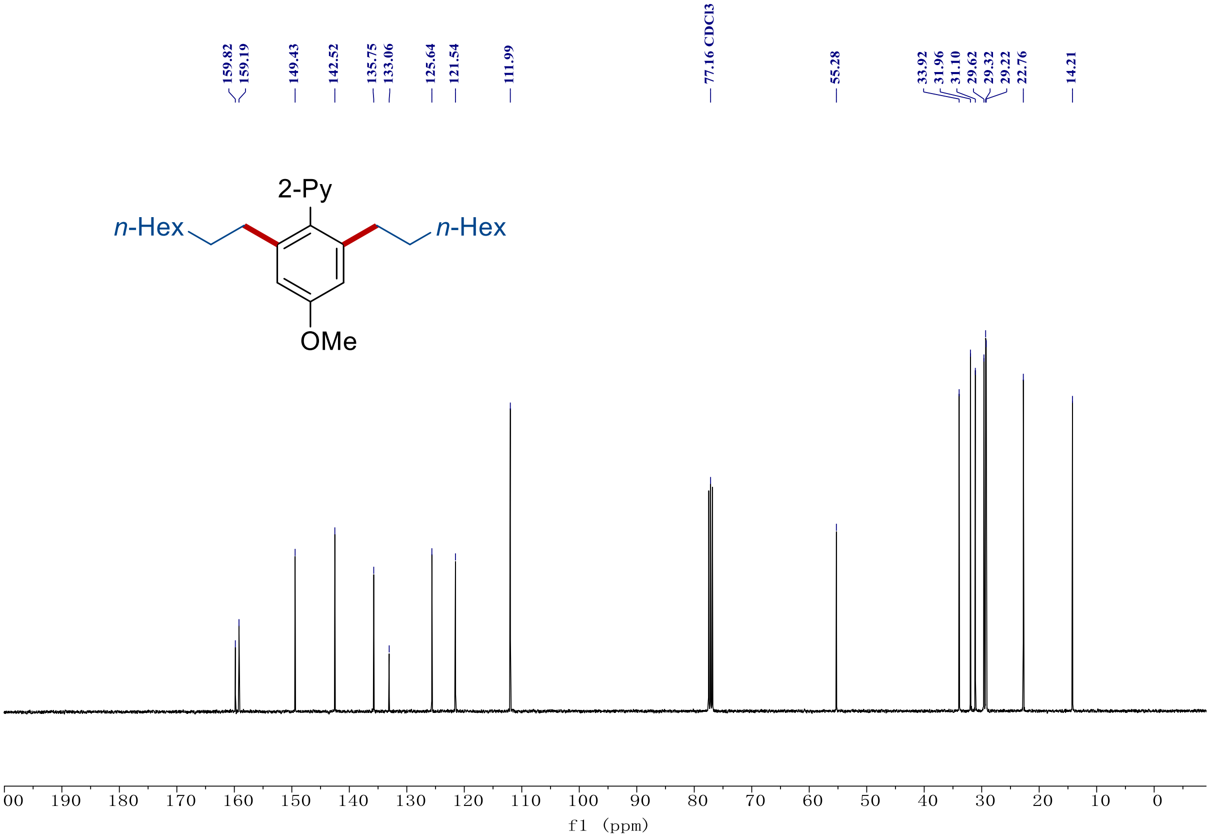

^{1}H NMR of **31a**, 300 MHz, $CDCl_3$, 25 °C.

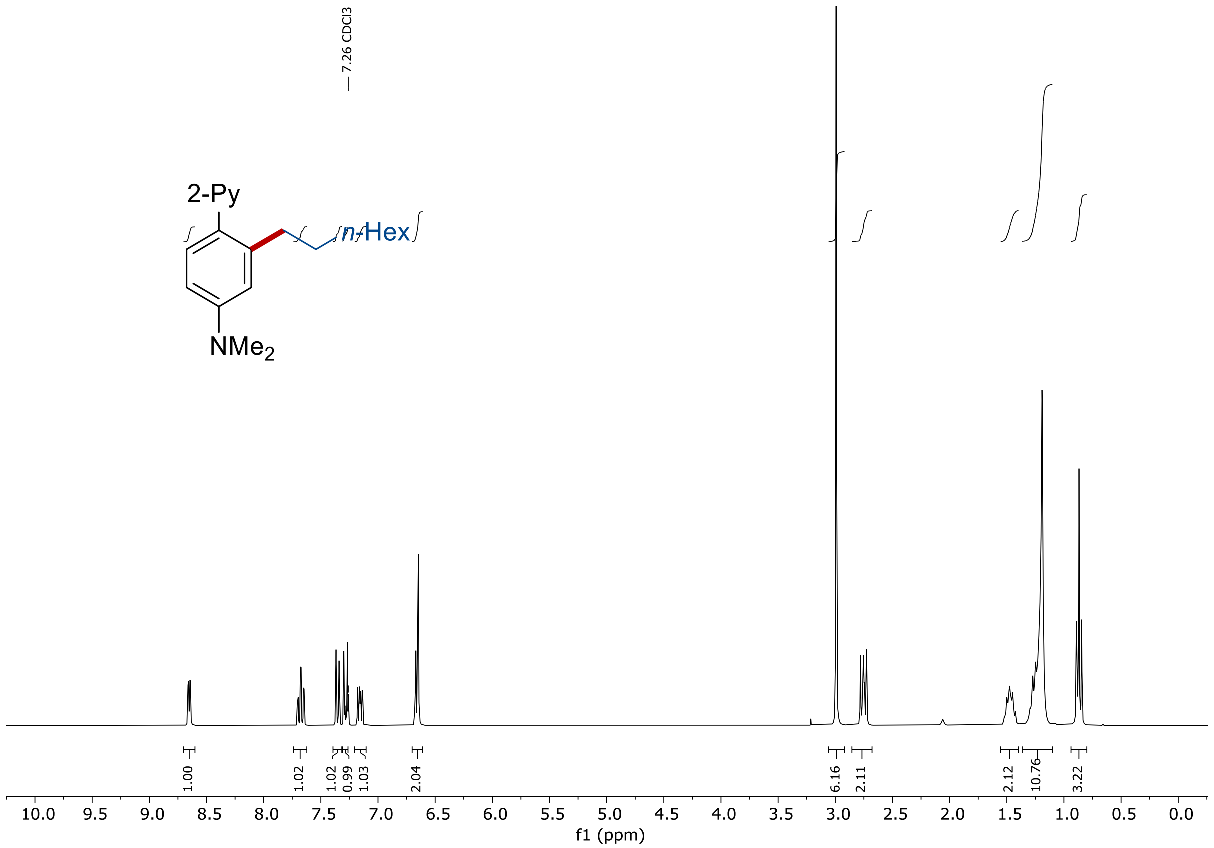

^{13}C NMR of **31a**, 75 MHz, $CDCl_3$, 25 °C.

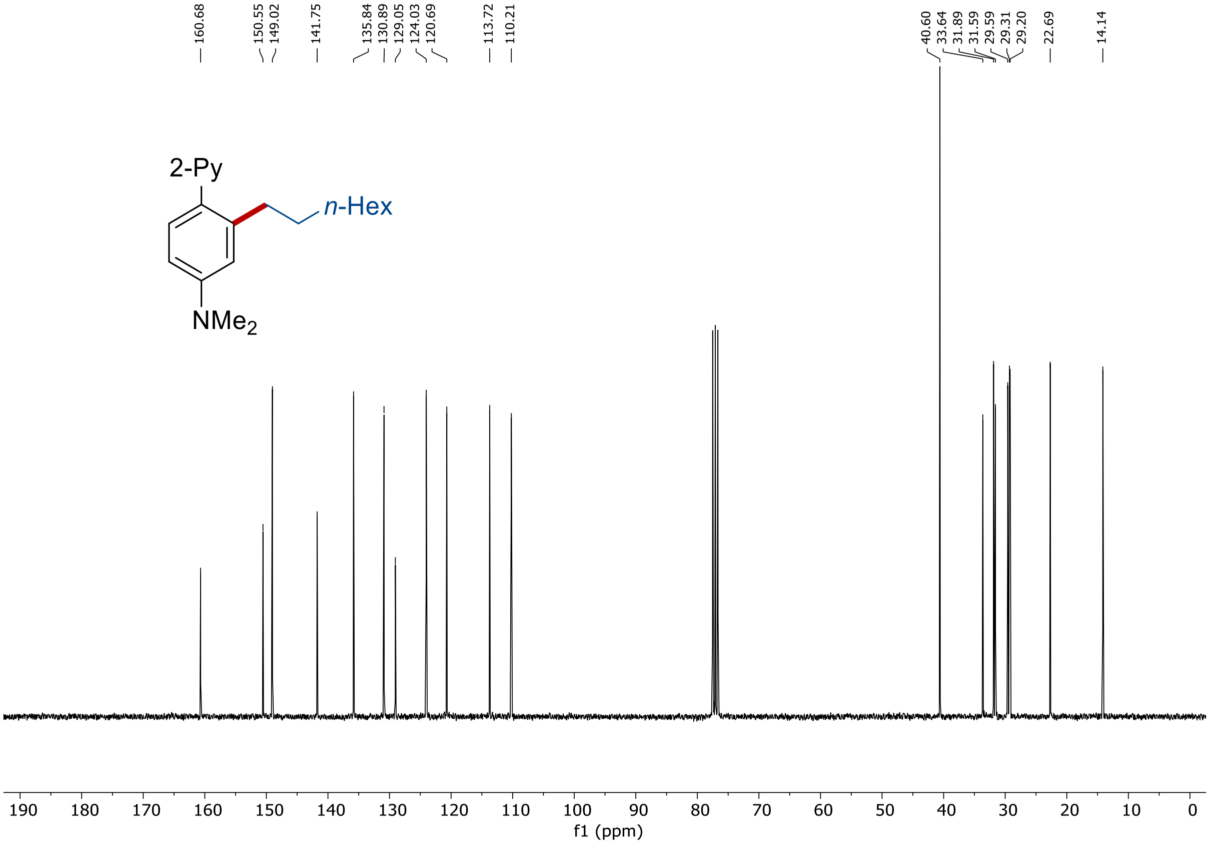

^{1}H NMR of **32a**, 300 MHz, $CDCl_3$, 25 °C.

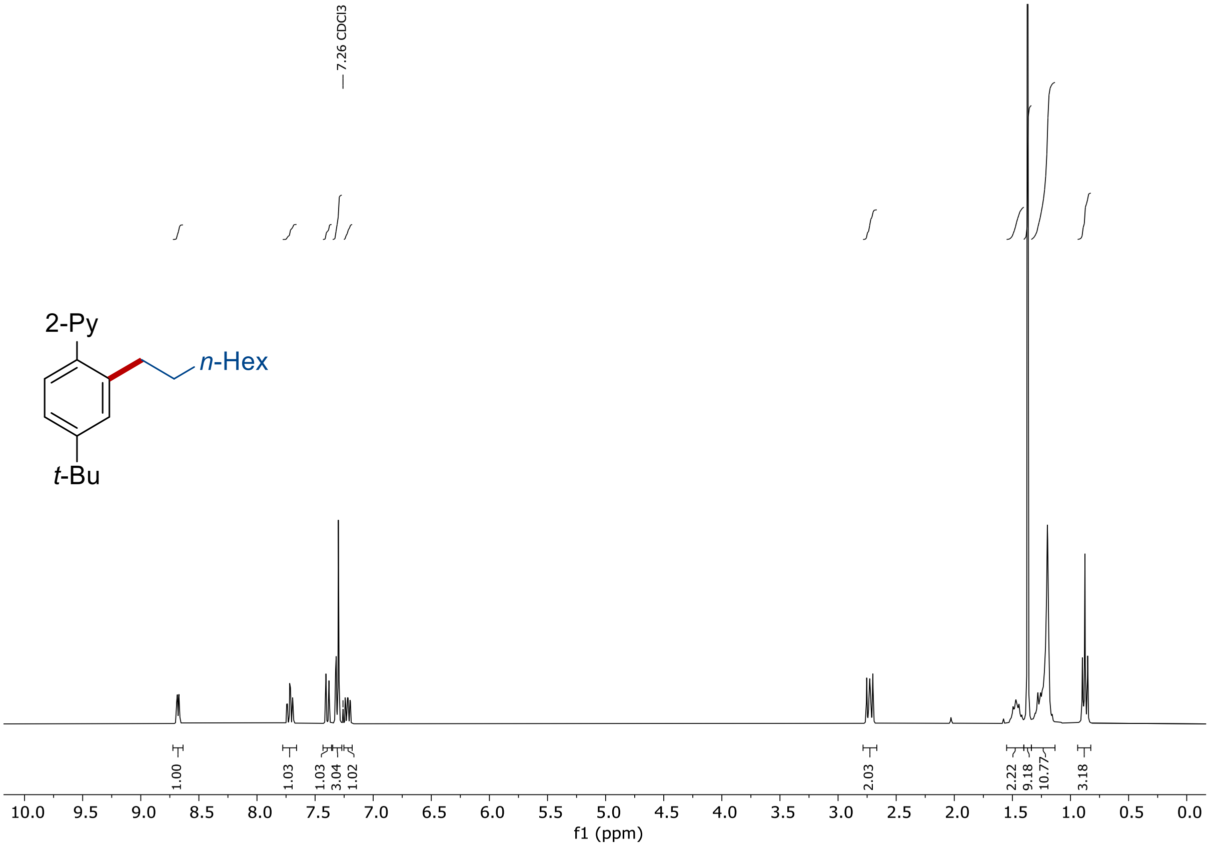

^{13}C NMR of **32a**, 75 MHz, $CDCl_3$, 25 °C.

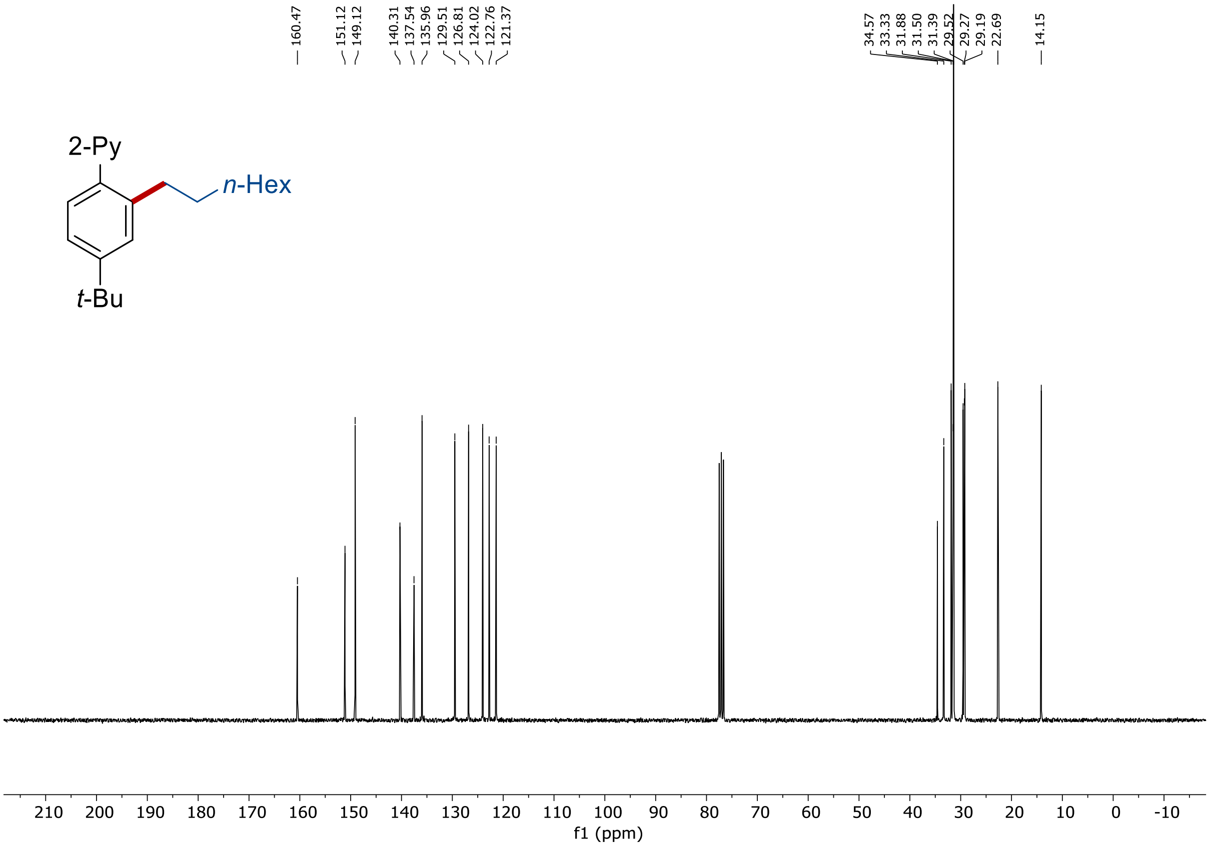

^{1}H NMR of **33a**, 300 MHz, $CDCl_3$, 25 °C.

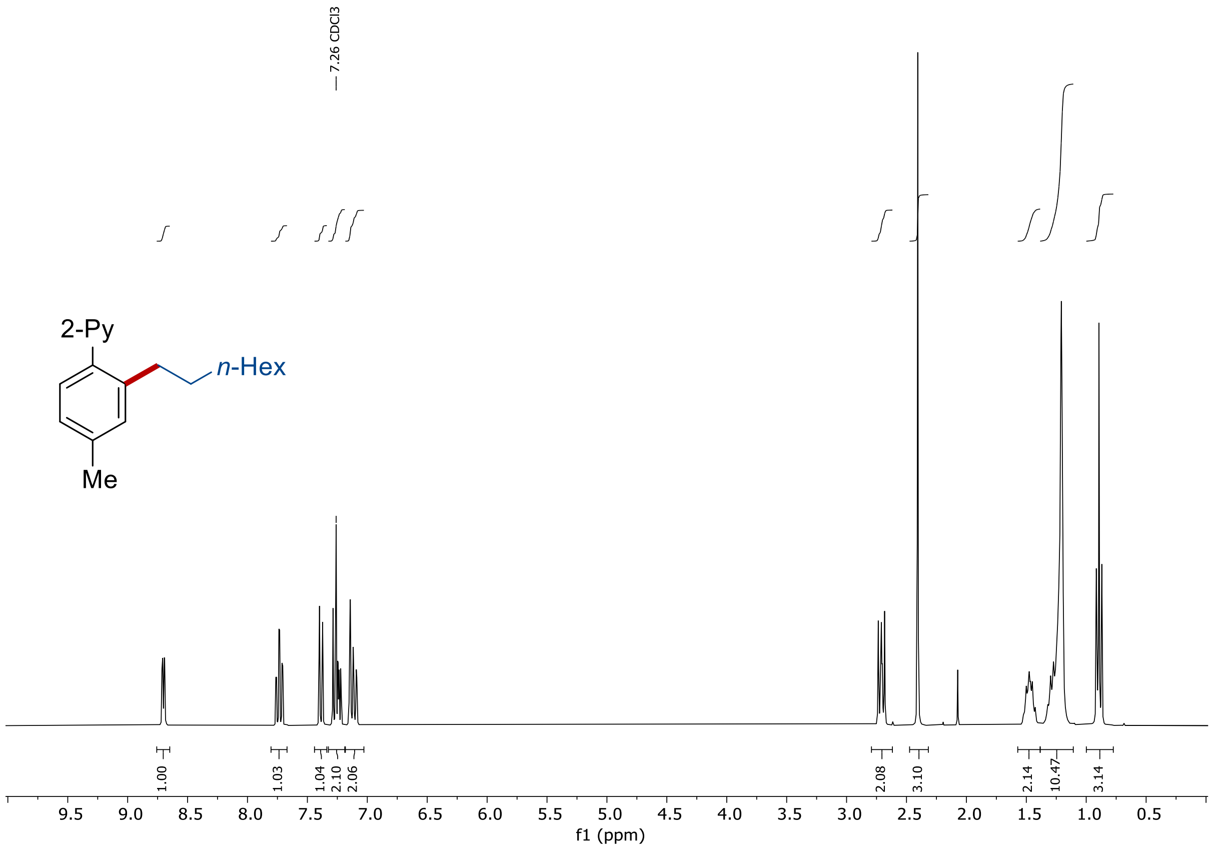

^{13}C NMR of **33a**, 75 MHz, $CDCl_3$, 25 °C.

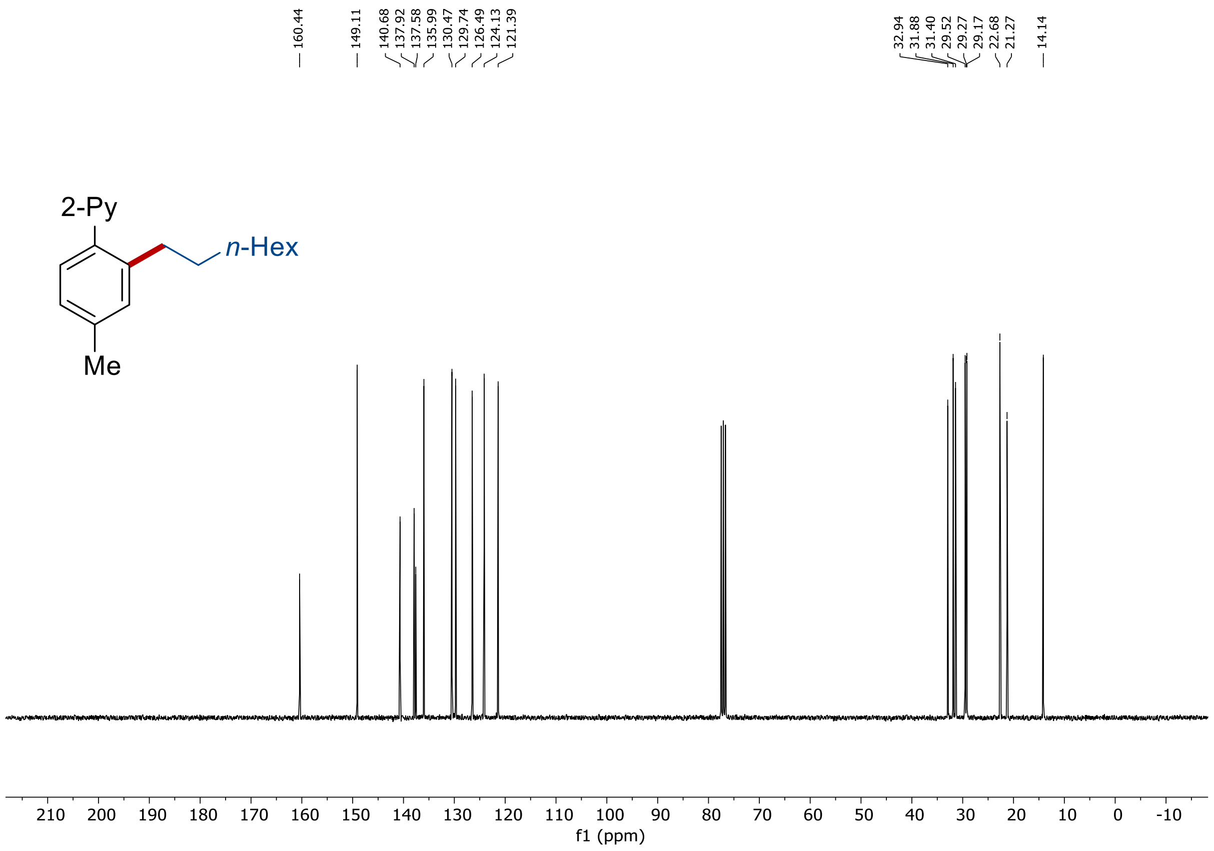

^{1}H NMR of **33b**, 300 MHz, $CDCl_3$, 25 °C.

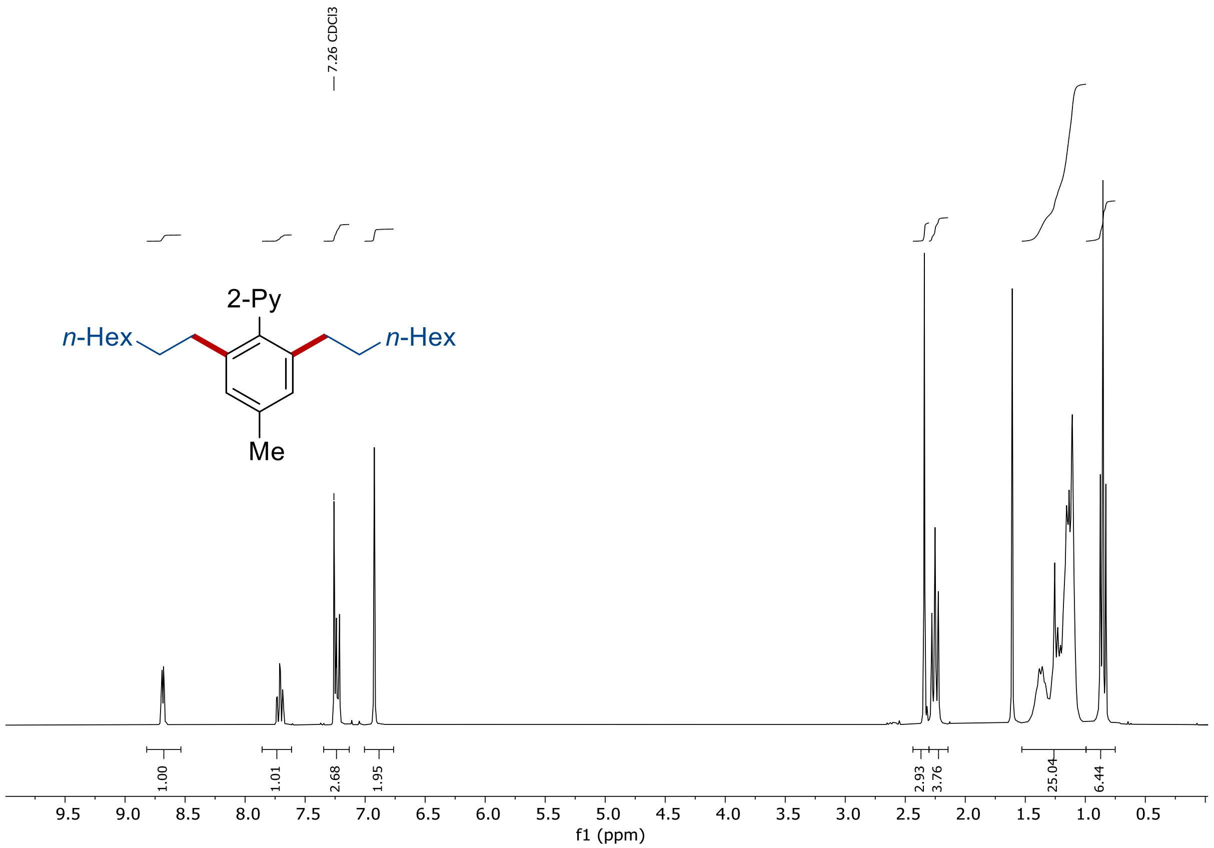

^{13}C NMR of **33b**, 75 MHz, $CDCl_3$, 25 °C.

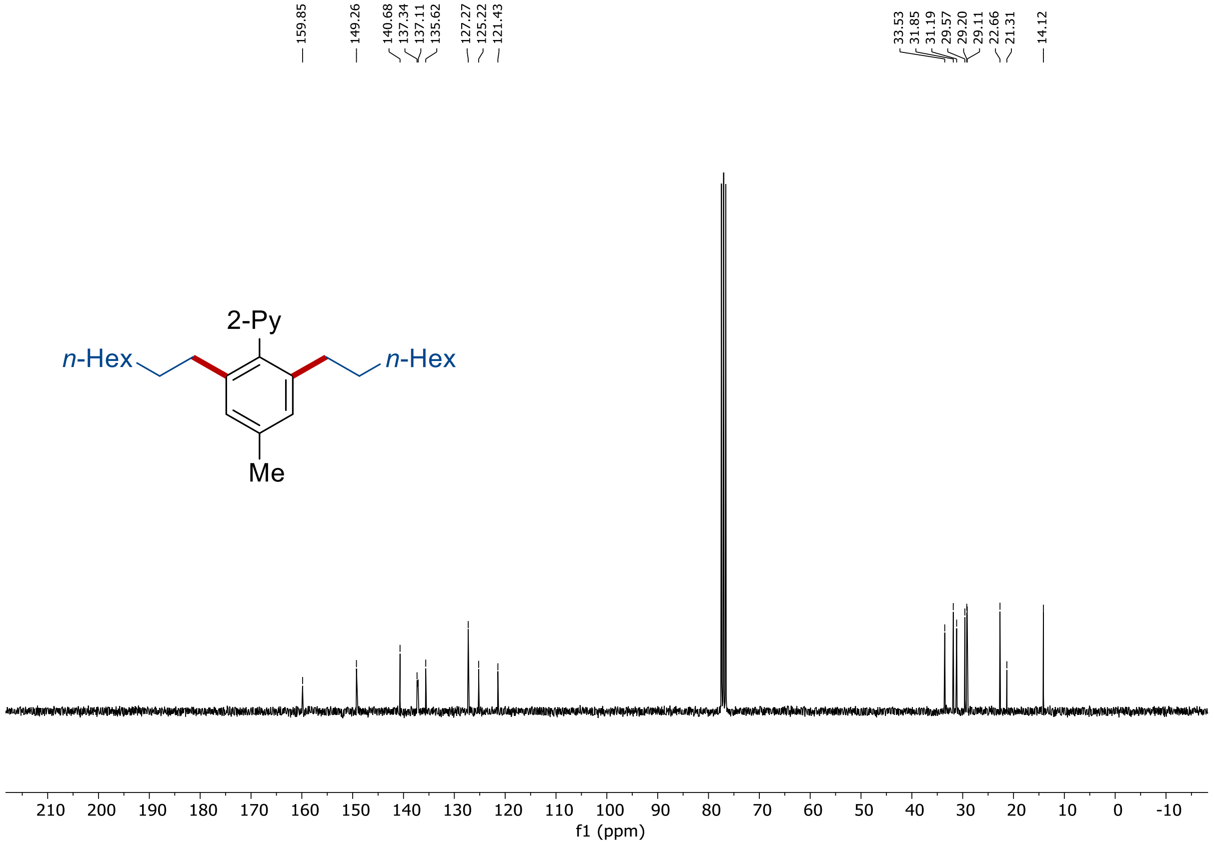

^{1}H NMR of **34a**, 500 MHz, $CDCl_3$, 25 °C.

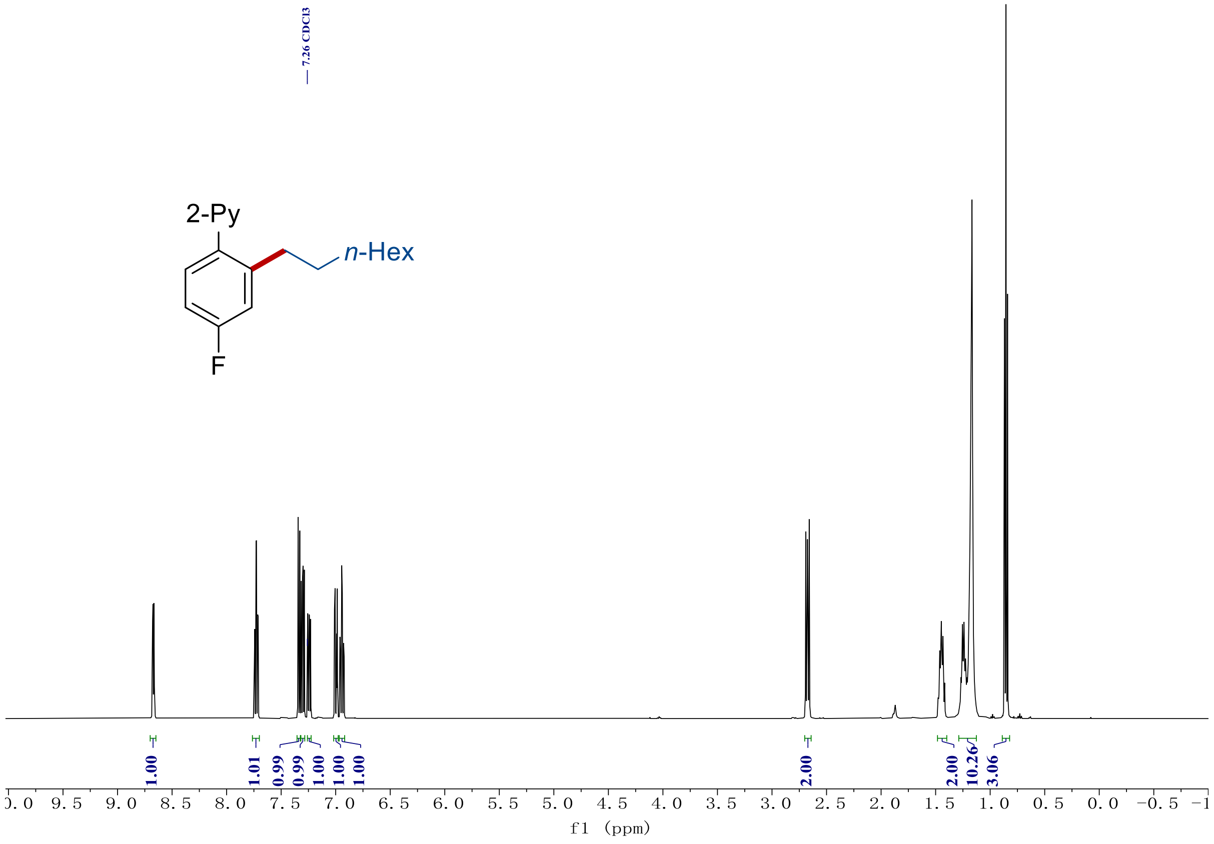

^{13}C NMR of **34a**, 126 MHz, $CDCl_3$, 25 °C.

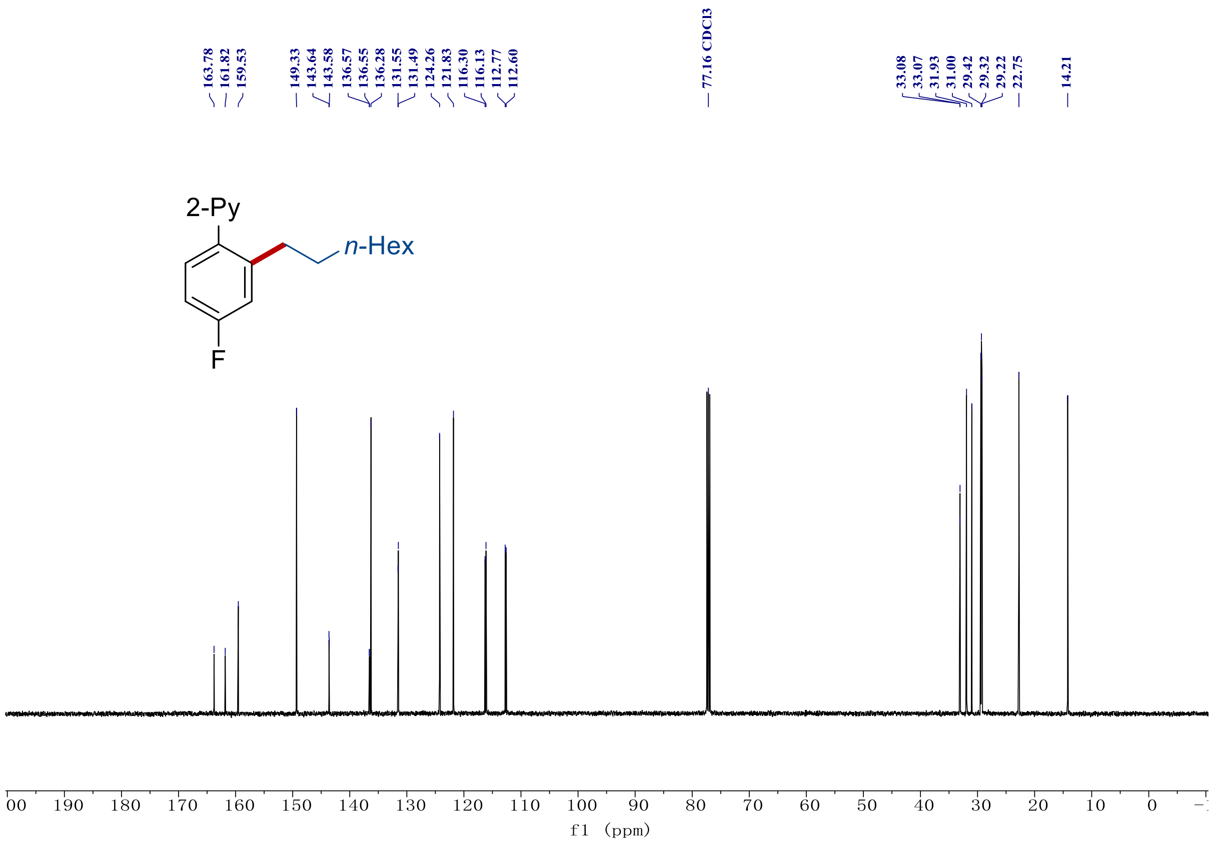

^{19}F NMR of **34a**, 471 MHz, $CDCl_3$, 25 °C.

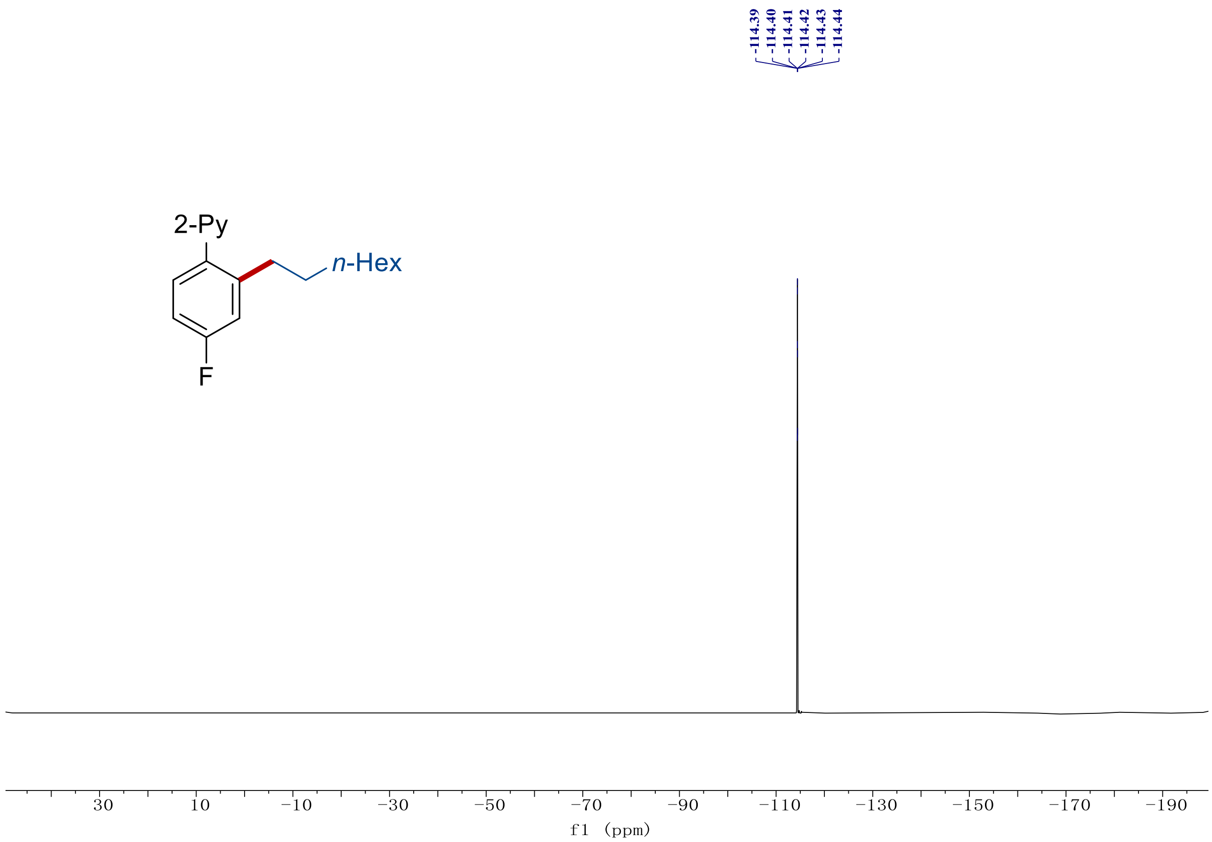

^{1}H NMR of **34b**, 400 MHz, $CDCl_3$, 25 °C.

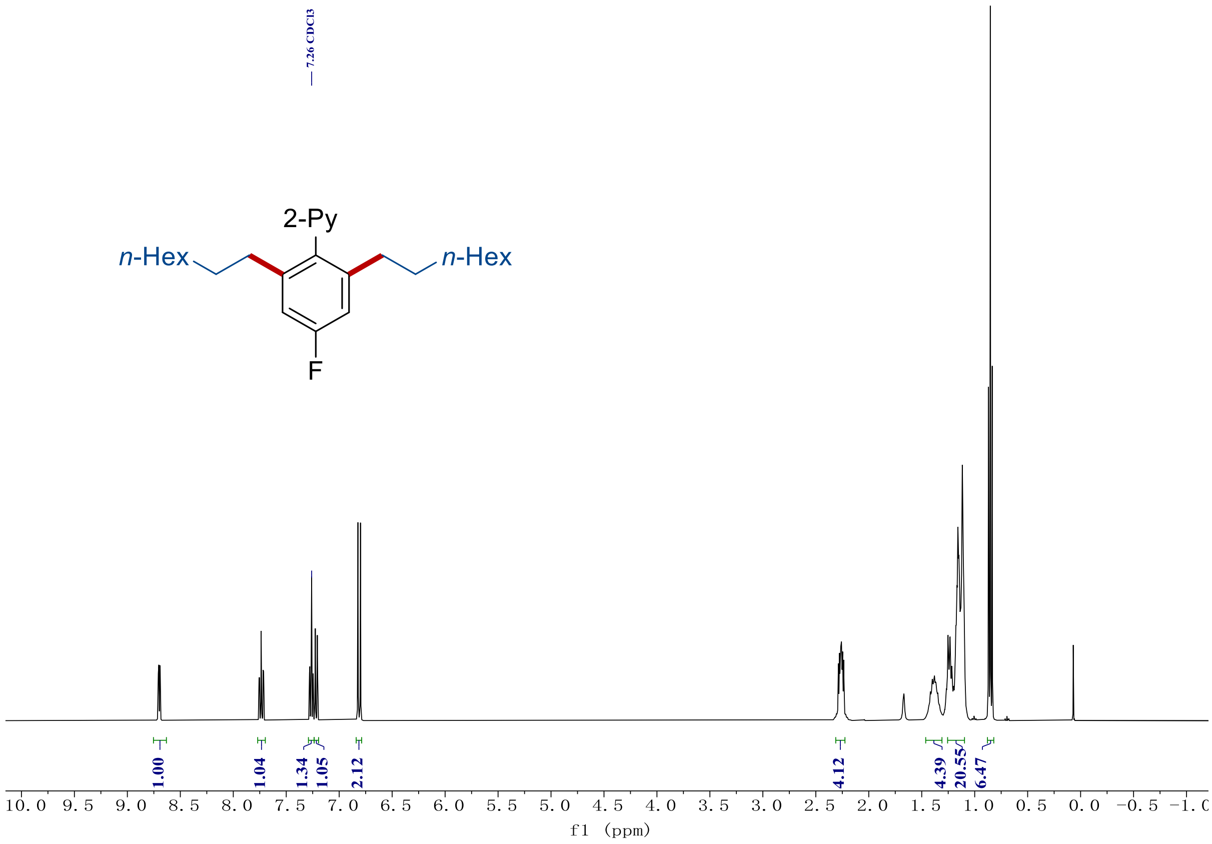

^{13}C NMR of **34b**, 101 MHz, $CDCl_3$, 25 °C.

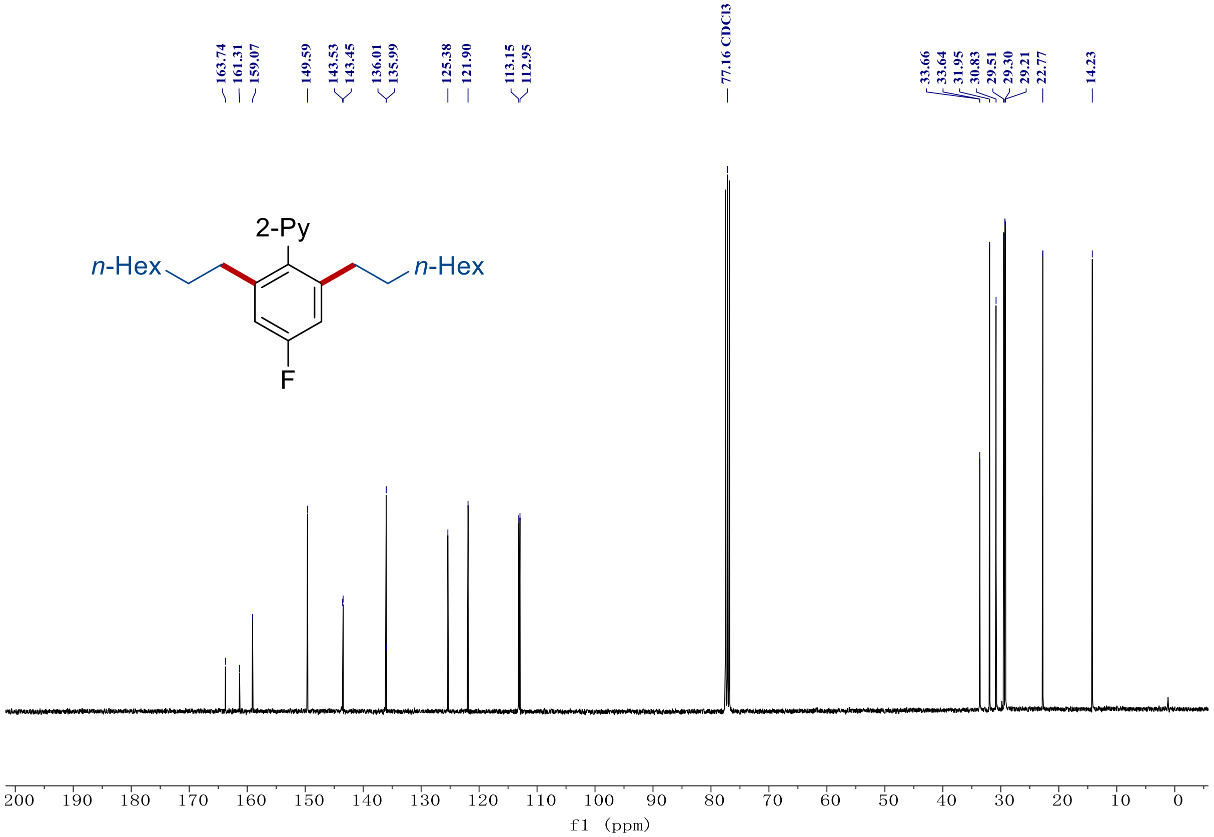

^{19}F NMR of **34b**, 282 MHz, $CDCl_3$, 25 °C.

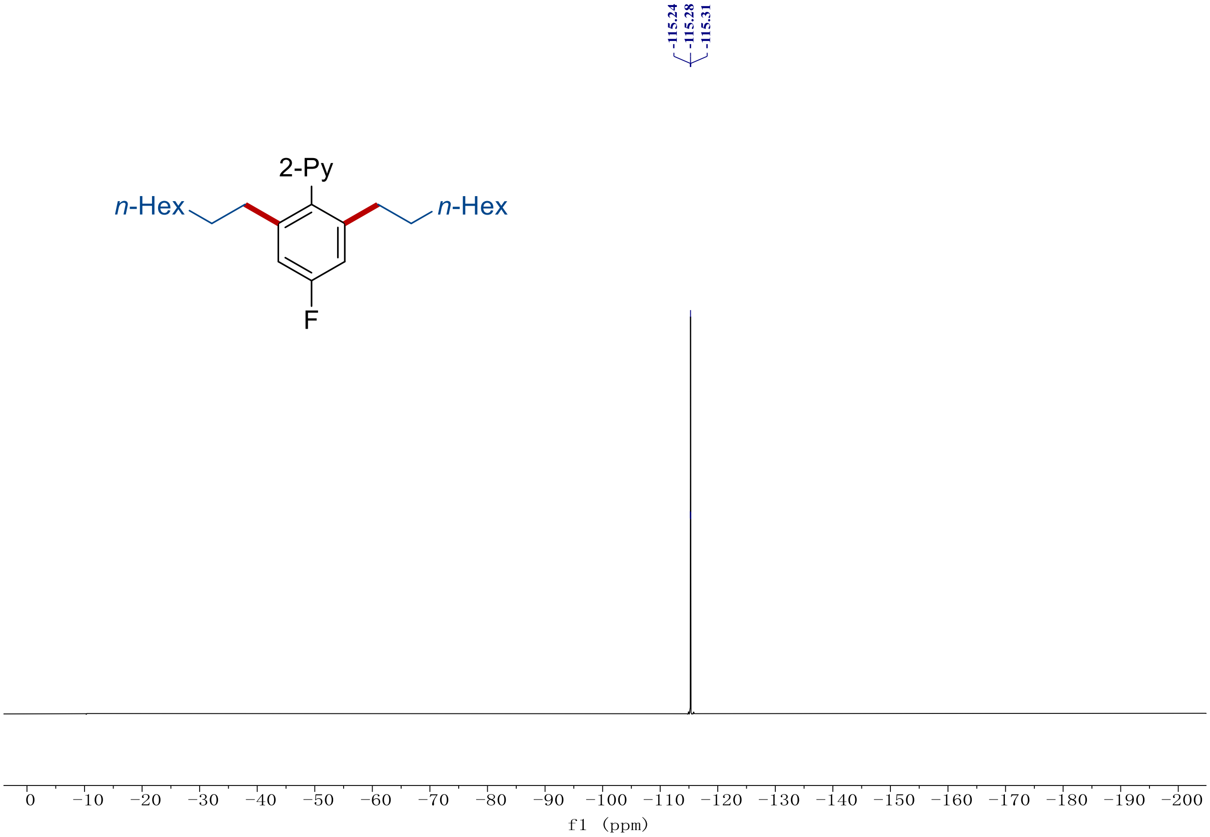

^{1}H NMR of **35a**, 300 MHz, $CDCl_3$, 25 °C.

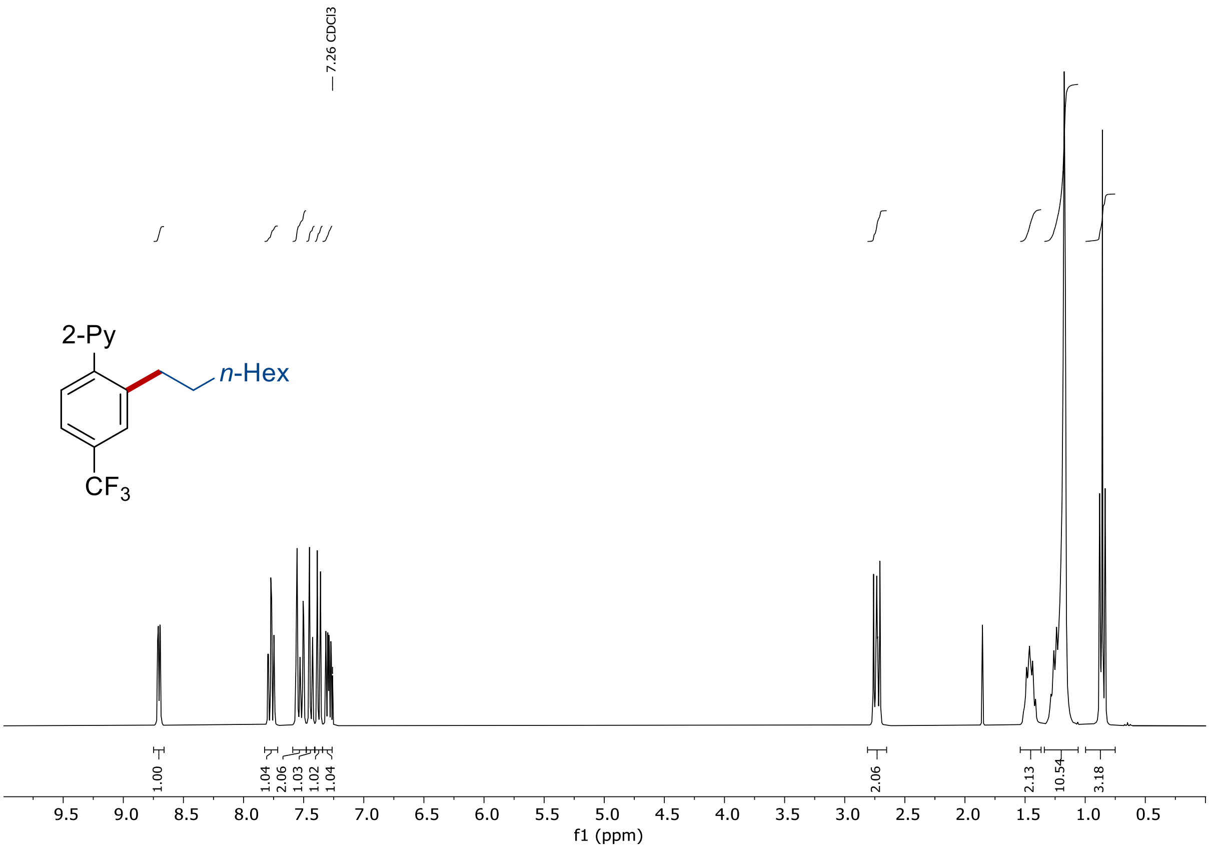

^{13}C NMR of **35a**, 75 MHz, $CDCl_3$, 25 °C.

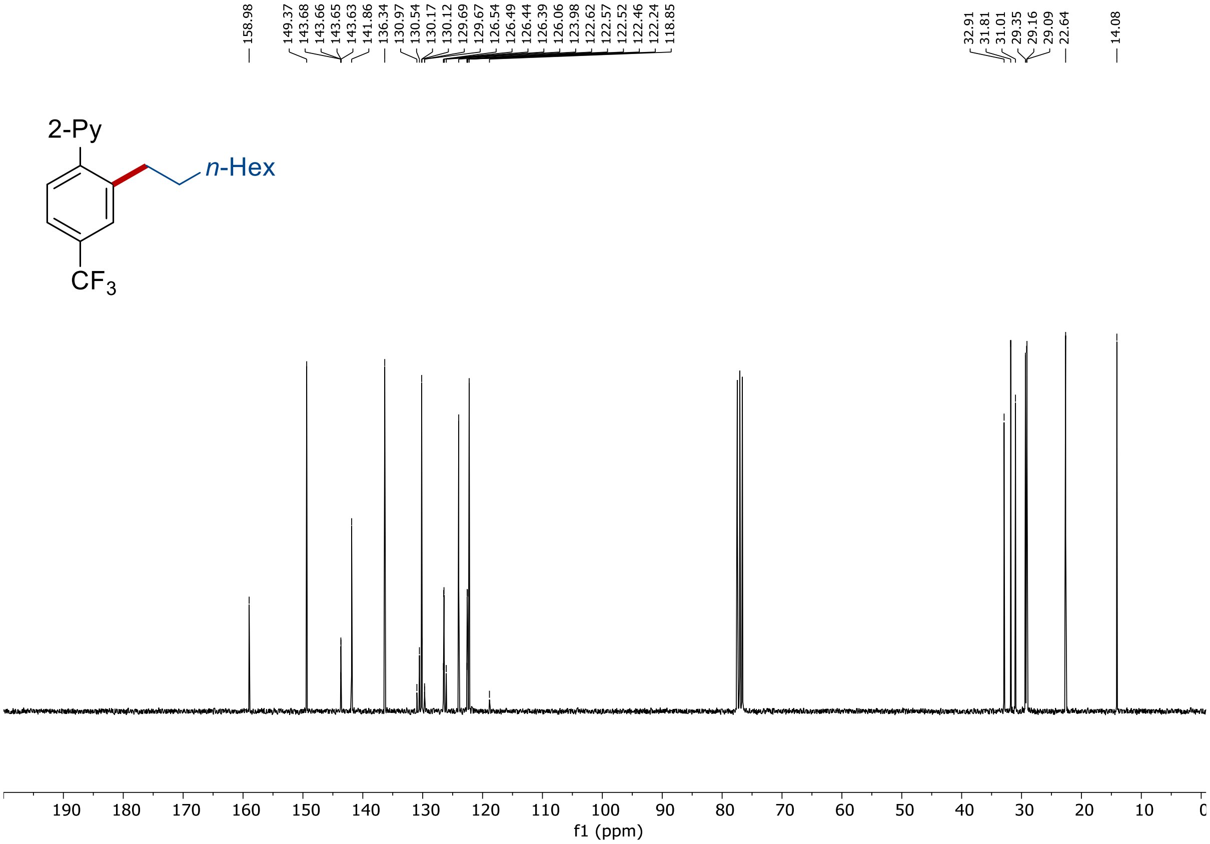

^{19}F NMR of **35a**, 282 MHz, $CDCl_3$, 25 °C.

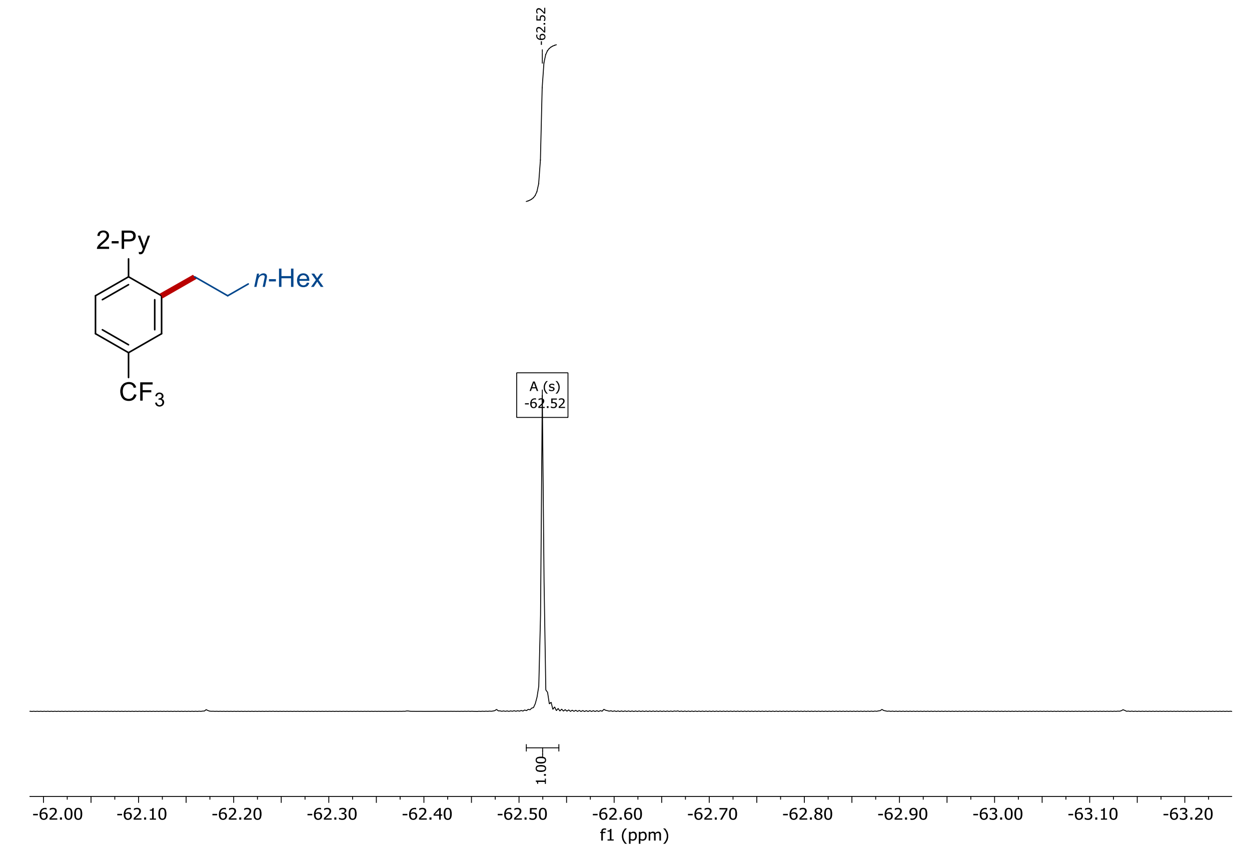

^{1}H NMR of **35b**, 300 MHz, $CDCl_3$, 25 °C.

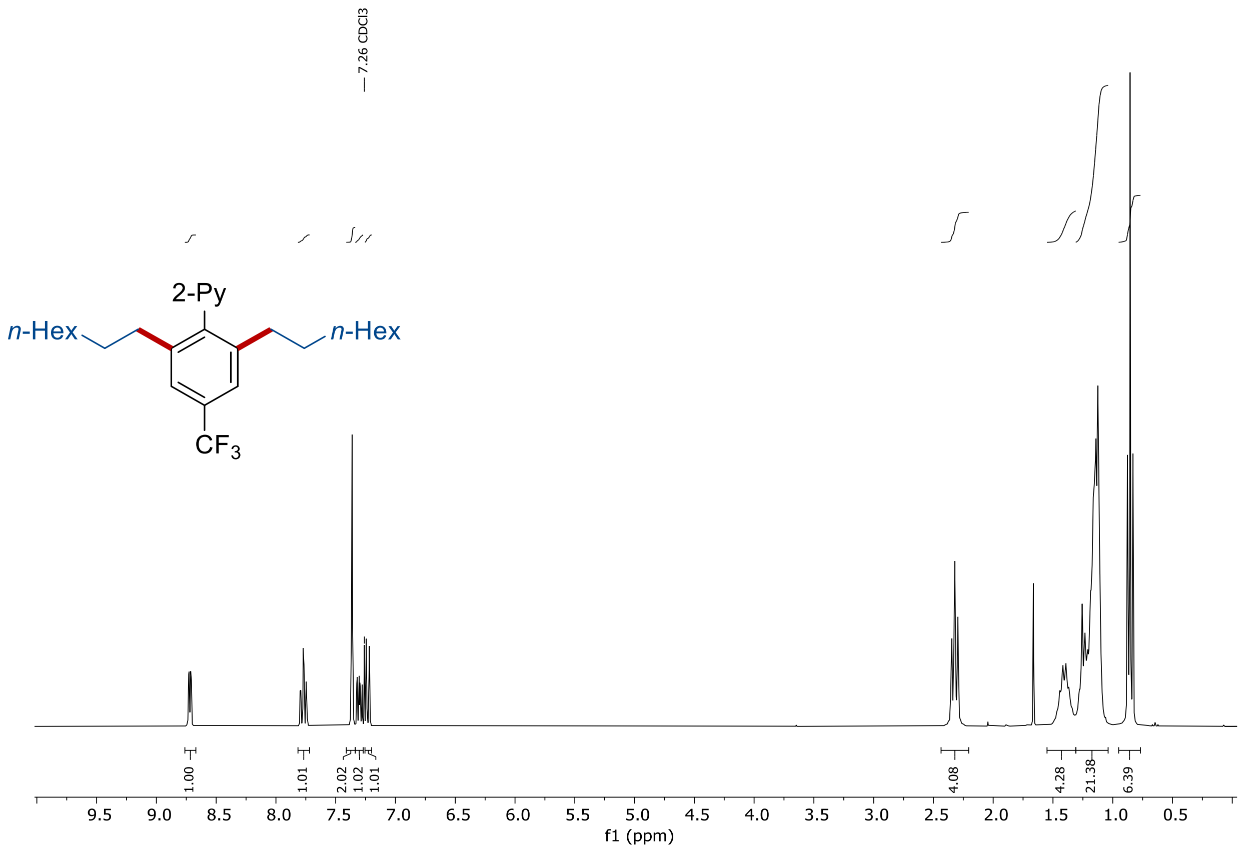

^{13}C NMR of **35b**, 75 MHz, $CDCl_3$, 25 °C.

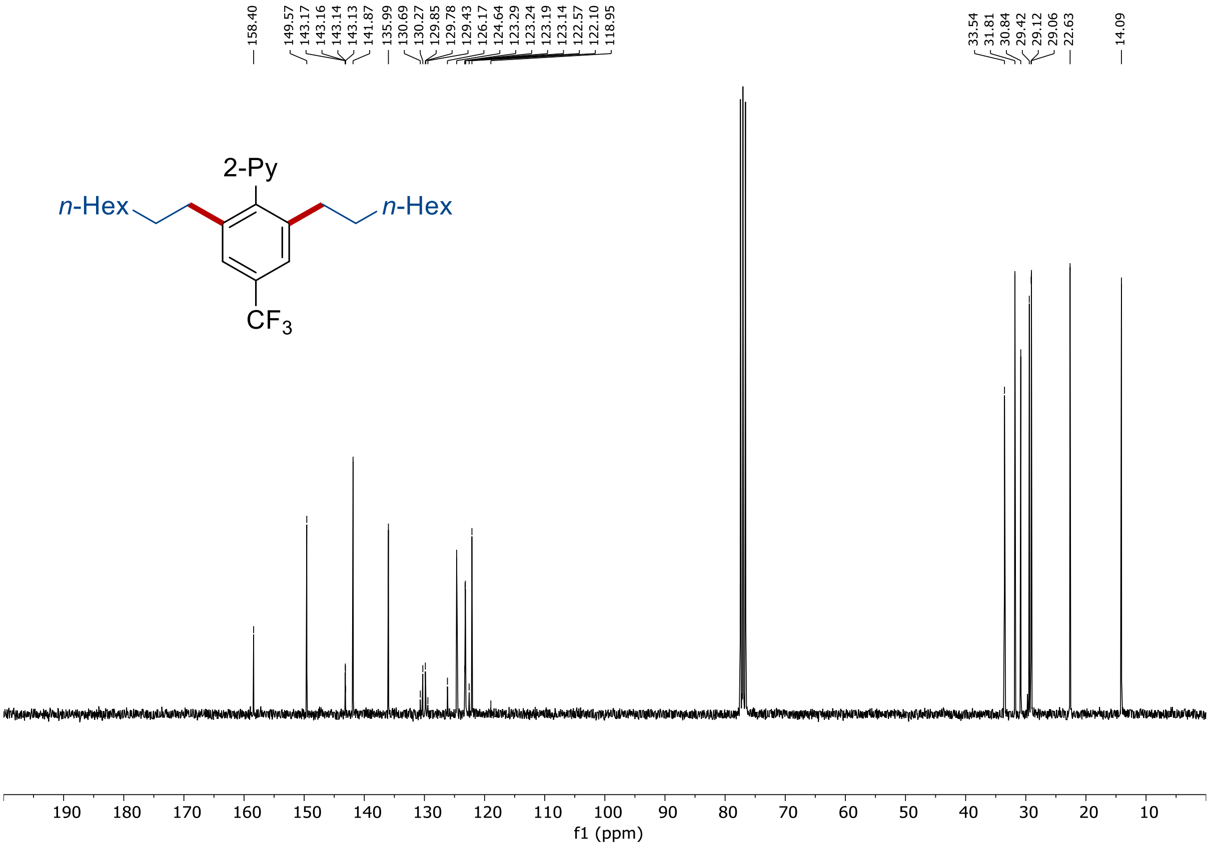

^{19}F NMR of **35b**, 282 MHz, $CDCl_3$, 25 °C.

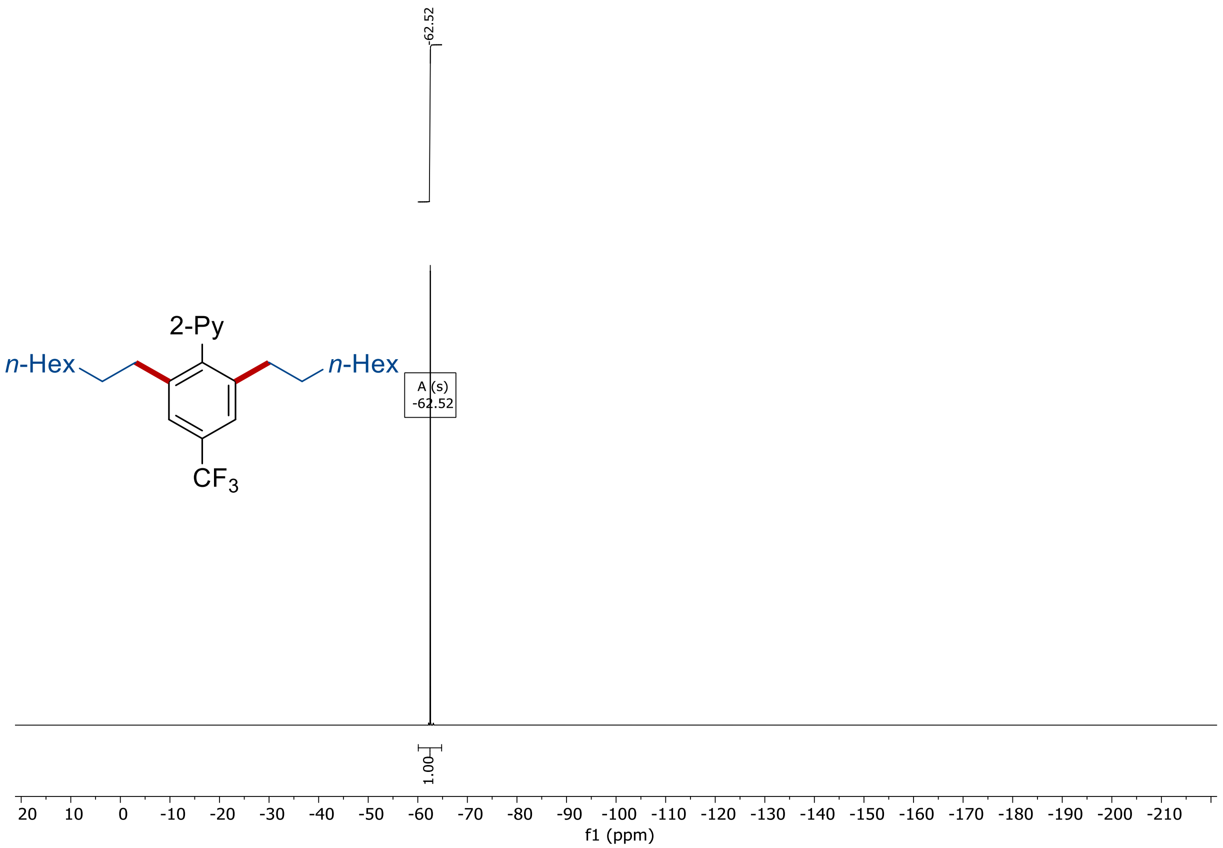

^{1}H NMR of **36a**, 500 MHz, $CDCl_3$, 25 °C.

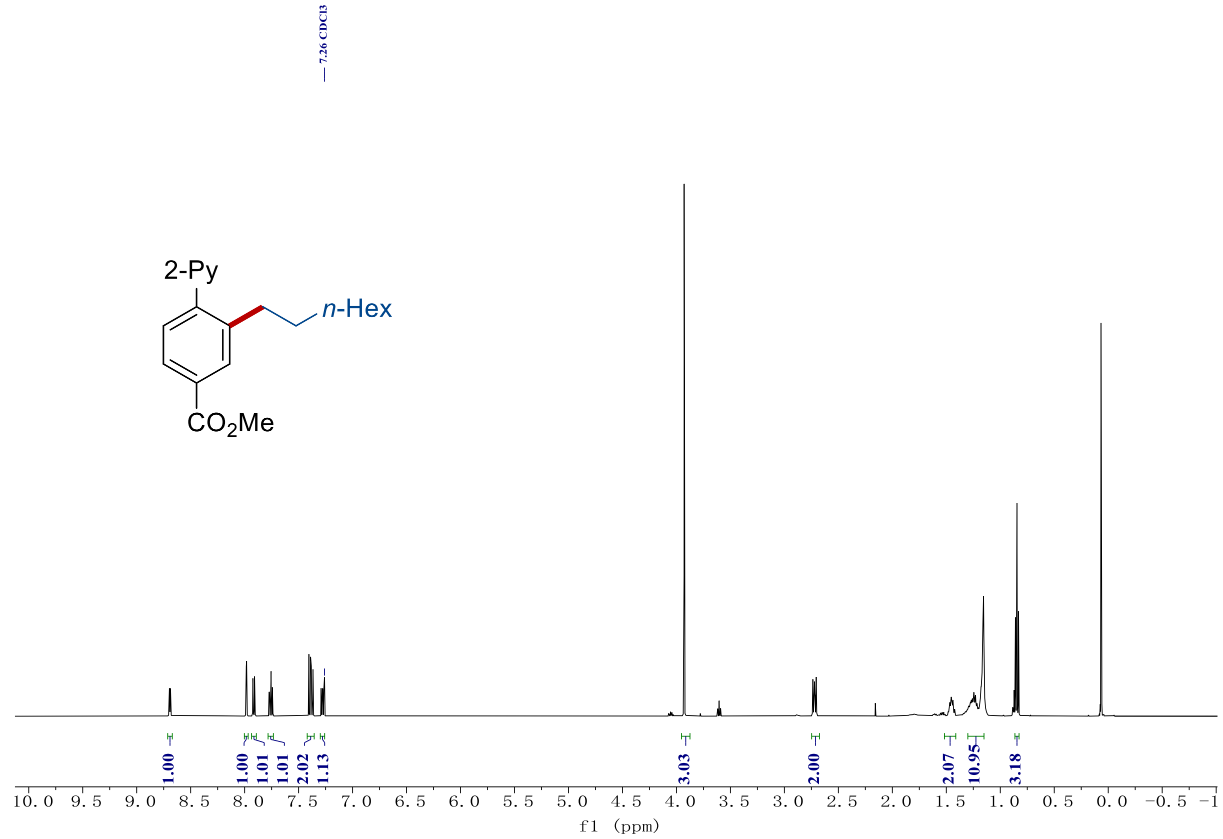

^{13}C NMR of **36a**, 126 MHz, $CDCl_3$, 25 °C.

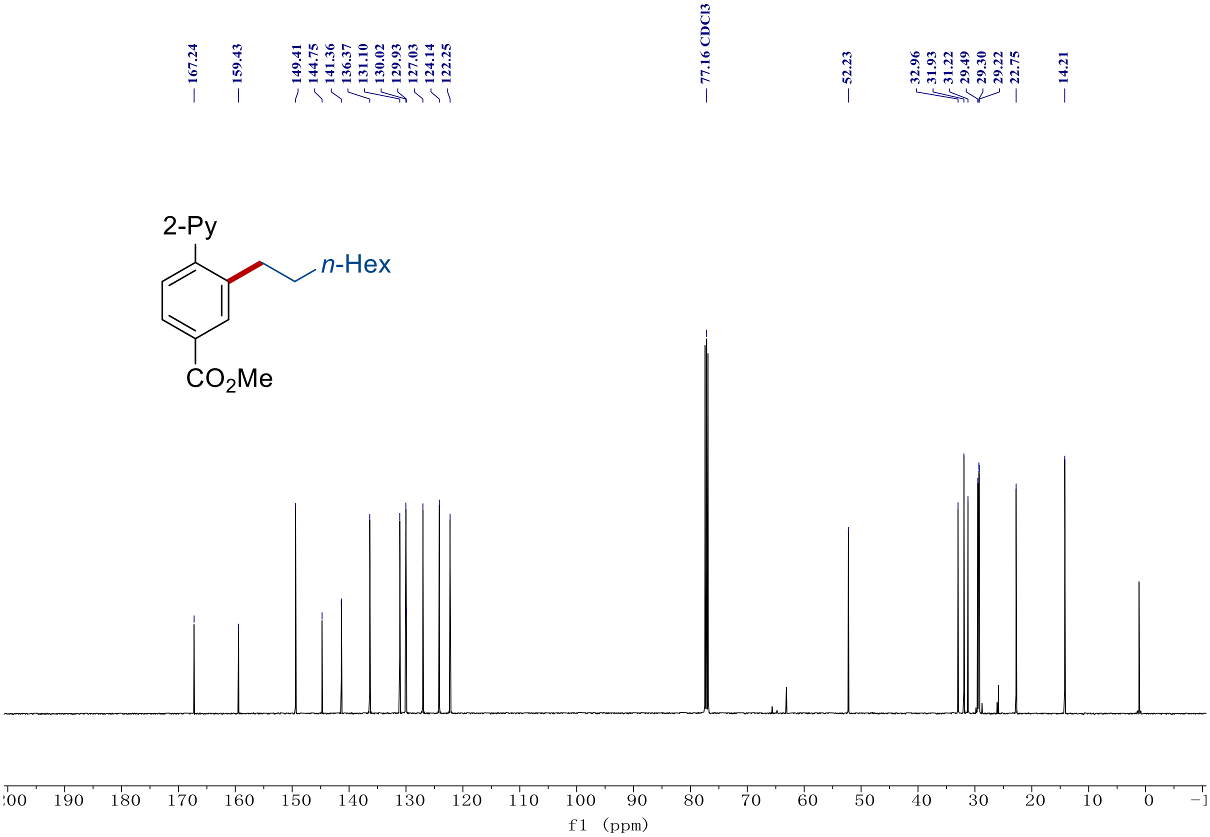

^{1}H NMR of **36b**, 400 MHz, $CDCl_3$, 25 °C.

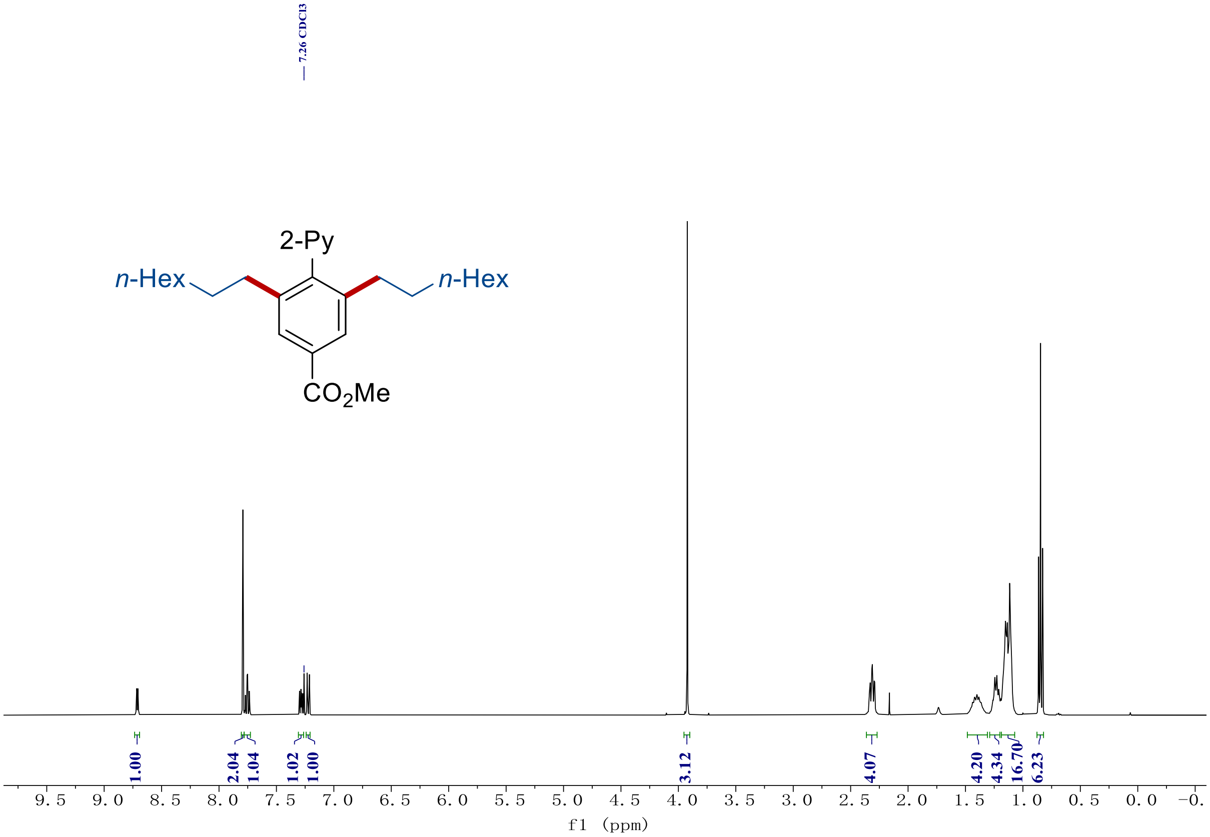

^{13}C NMR of **36b**, 101 MHz, $CDCl_3$, 25 °C.

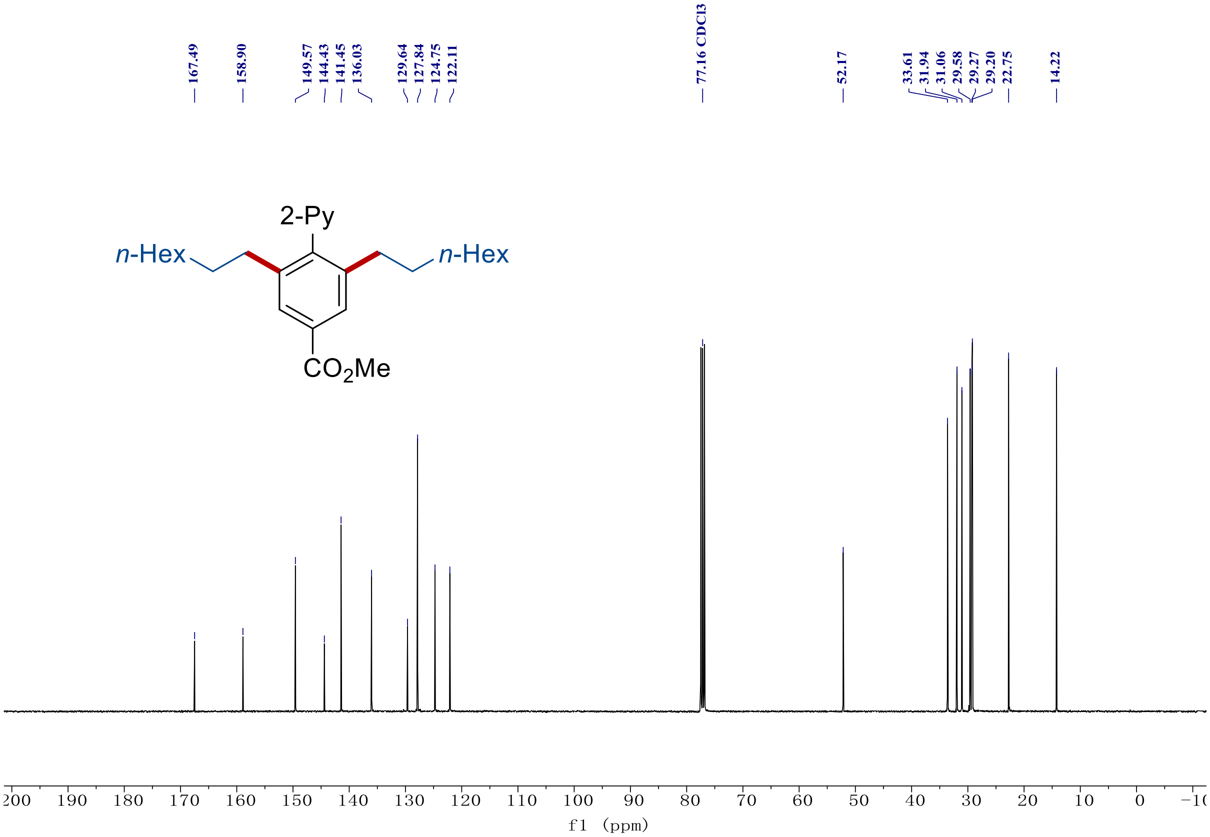

^{1}H NMR of **37a**, 600 MHz, $CDCl_3$, 25 °C.

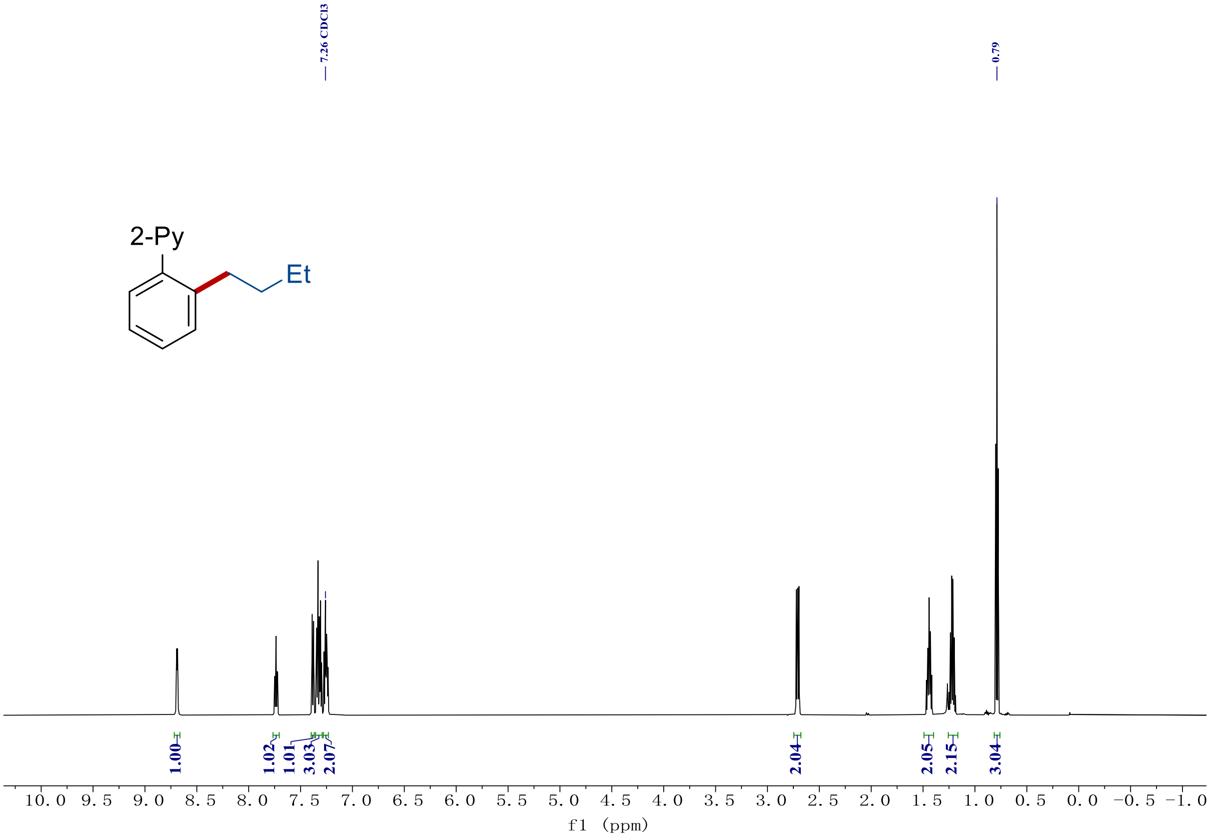

^{13}C NMR of **37a**, 151 MHz, $CDCl_3$, 25 °C.

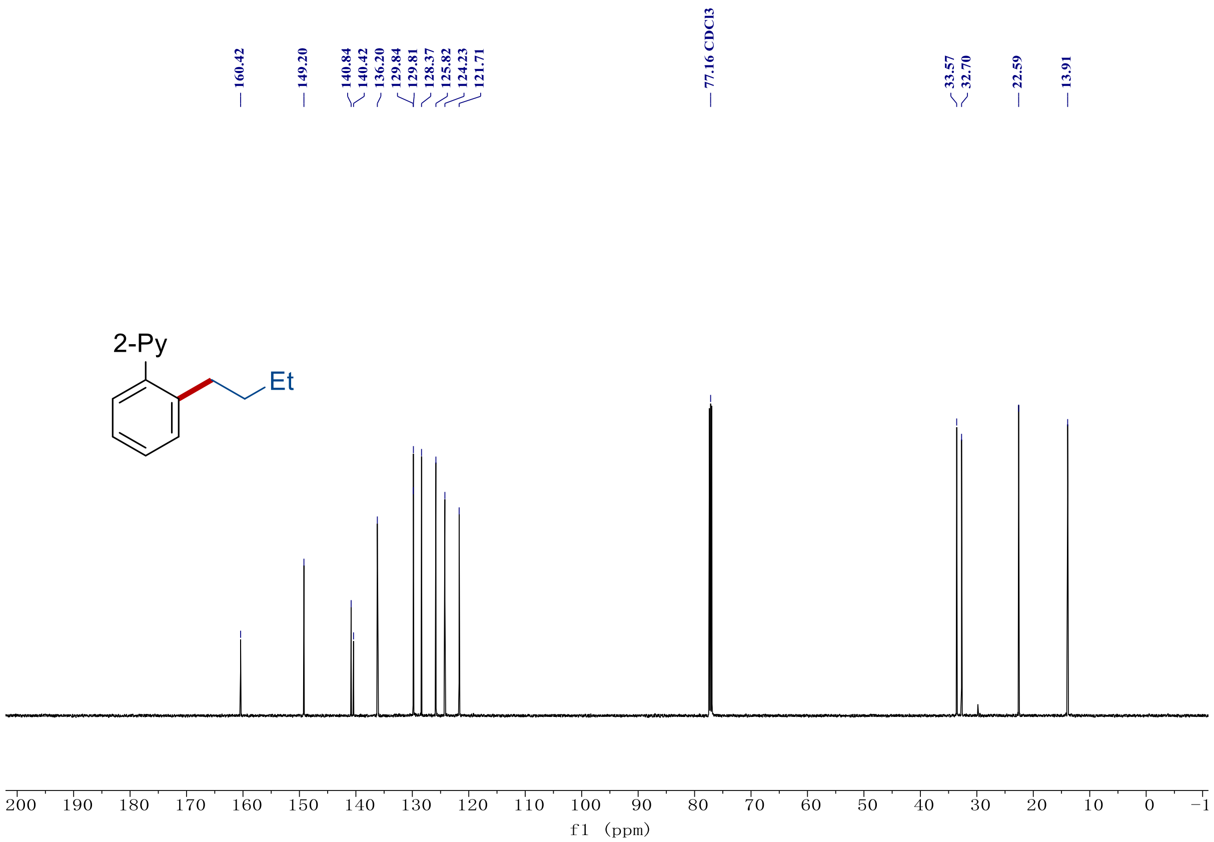

^{1}H NMR of **37b**, 500 MHz, $CDCl_3$, 25 °C.

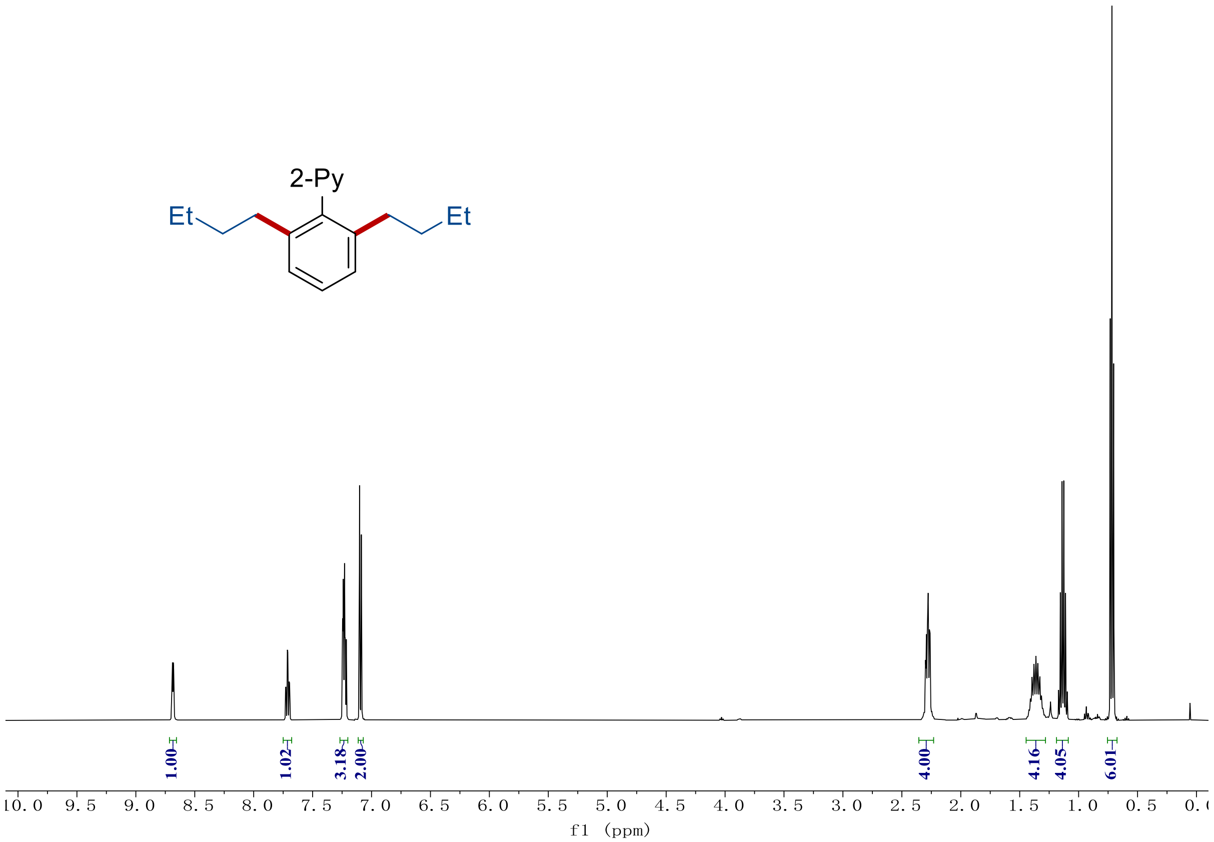

^{13}C NMR of **37b**, 126 MHz, $CDCl_3$, 25 °C.

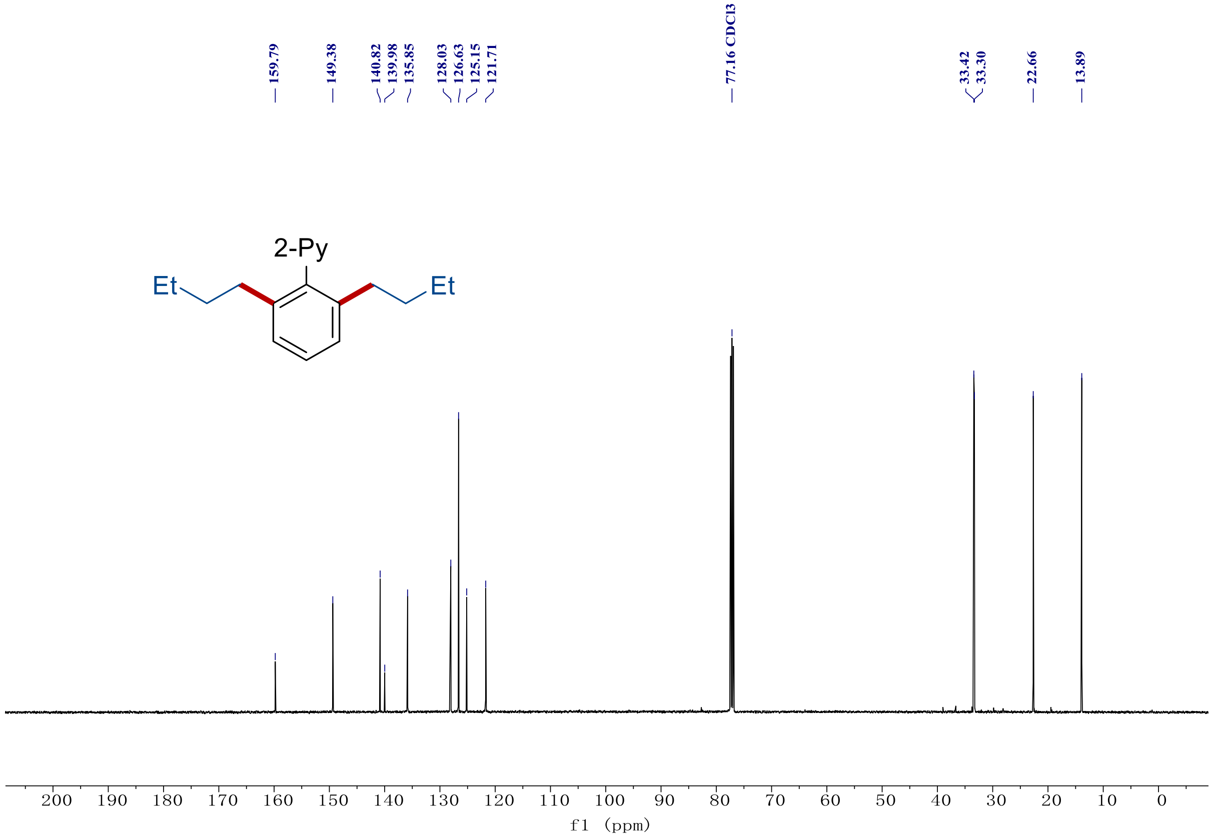

^{1}H NMR of **38a**, 300 MHz, $CDCl_3$, 25 °C.

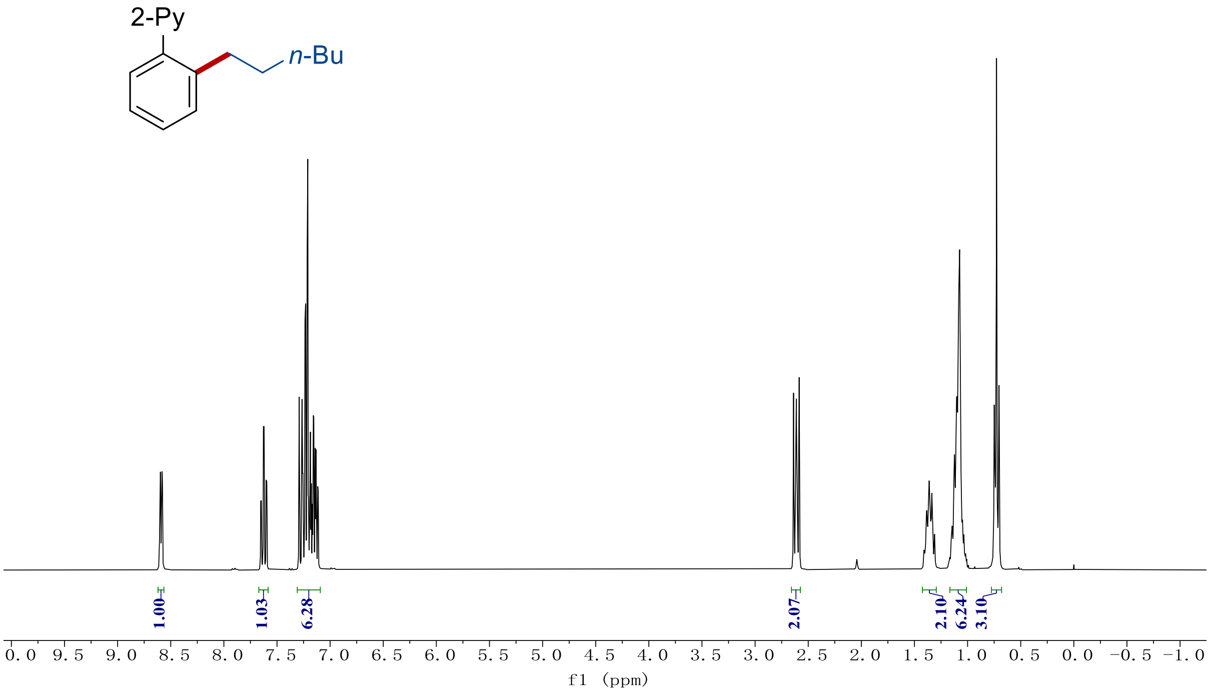

^{13}C NMR of **38a**, 75 MHz, $CDCl_3$, 25 °C.

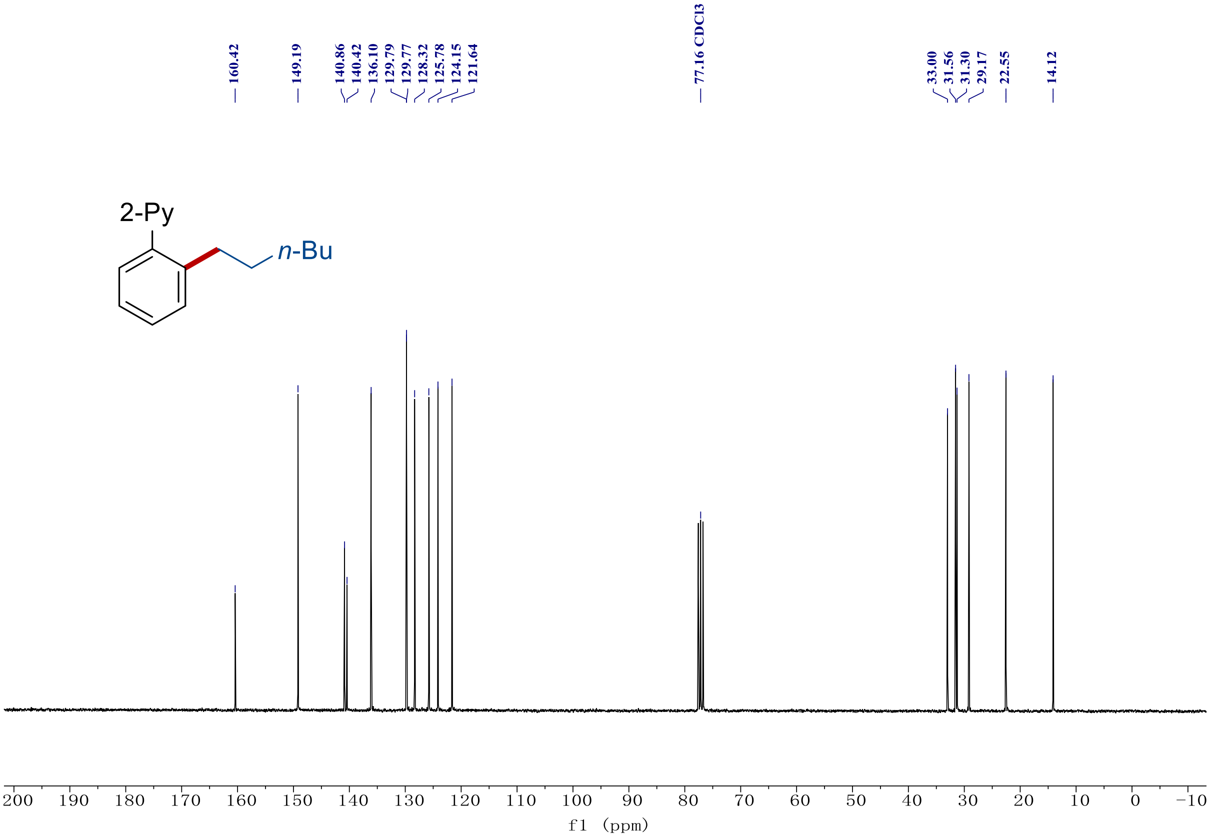

^{1}H NMR of **39a**, 400 MHz, $CDCl_3$, 25 °C.

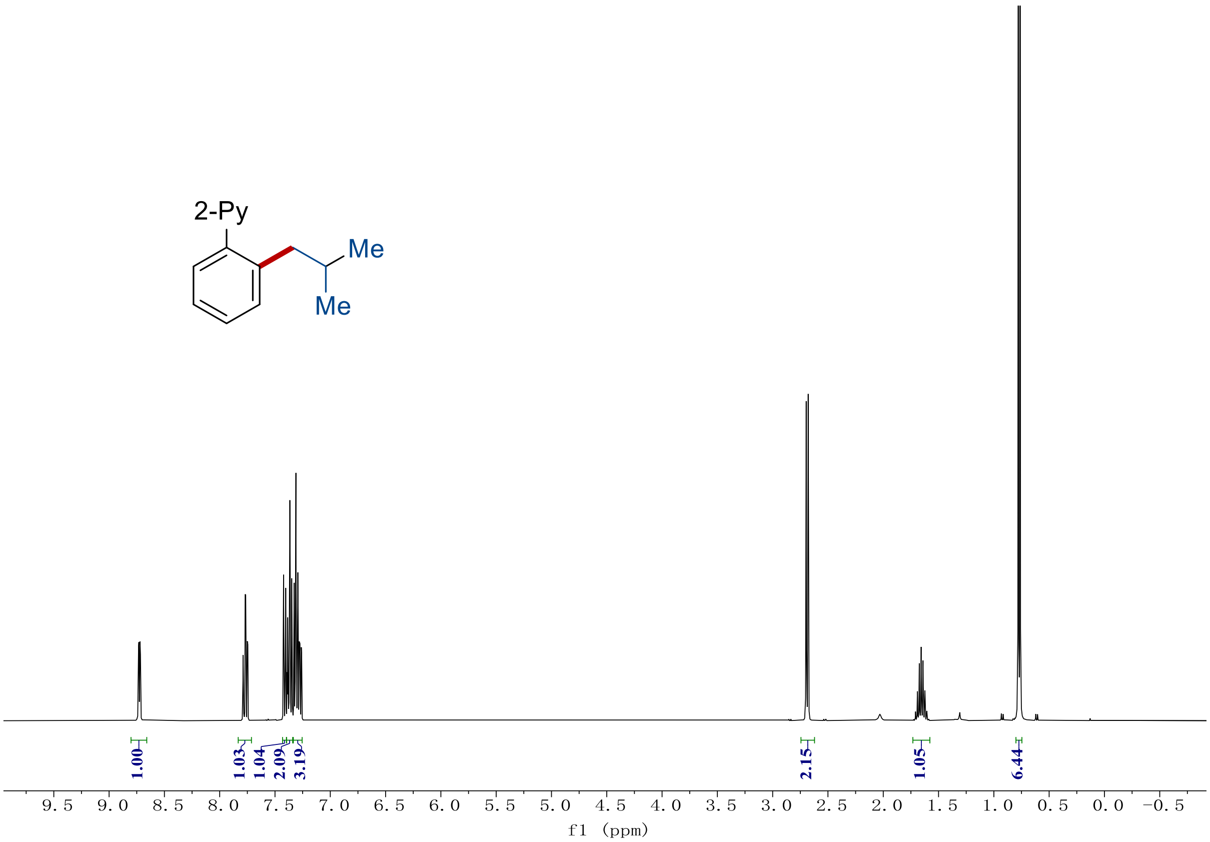

^{13}C NMR of **39a**, 101 MHz, $CDCl_3$, 25 °C.

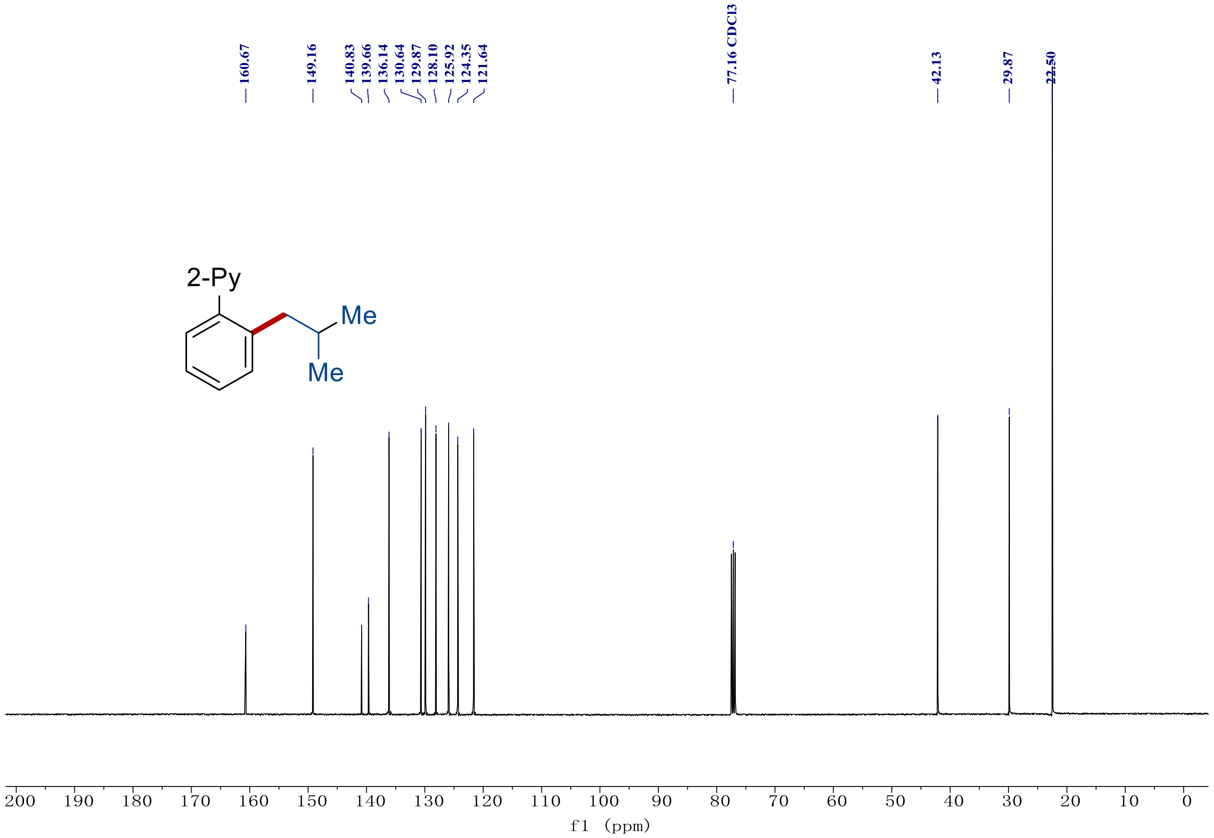

^{1}H NMR of **39b**, 400 MHz, $CDCl_3$, 25 °C.

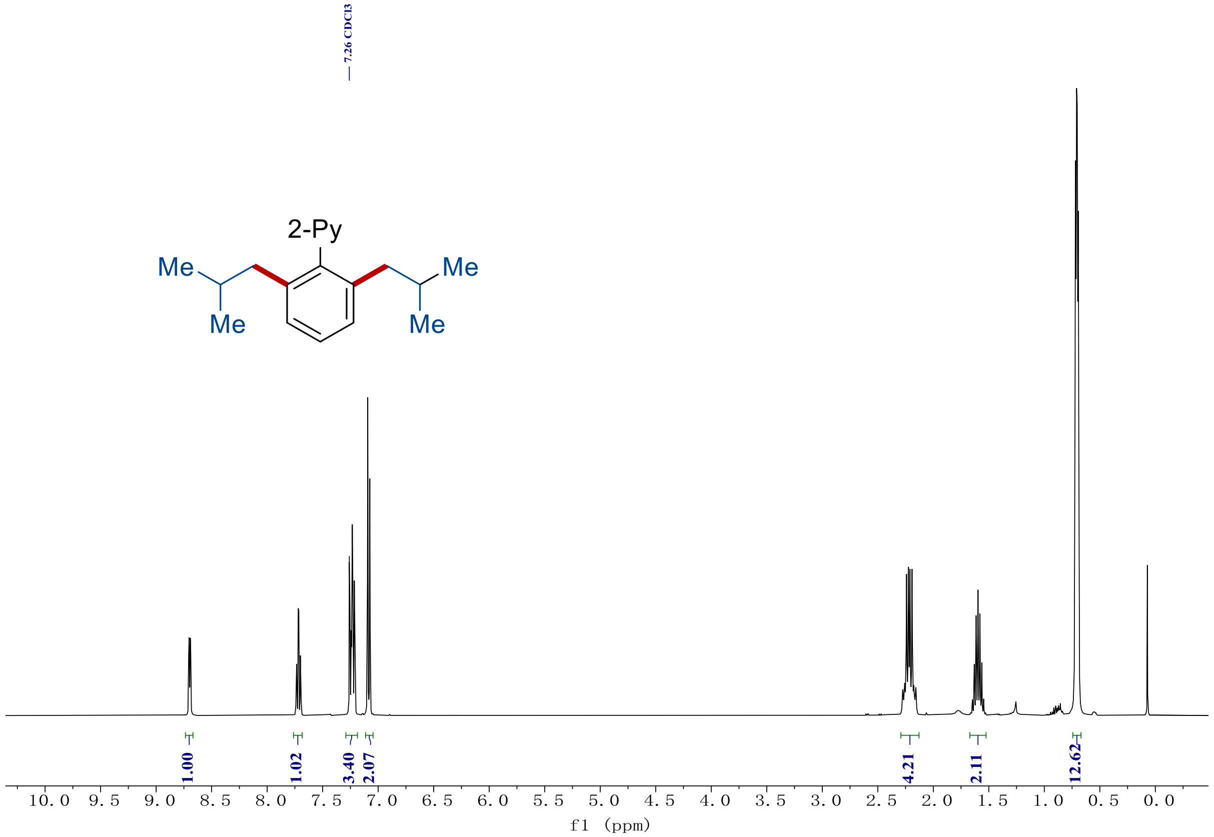

^{13}C NMR of **39b**, 101 MHz, $CDCl_3$, 25 °C.

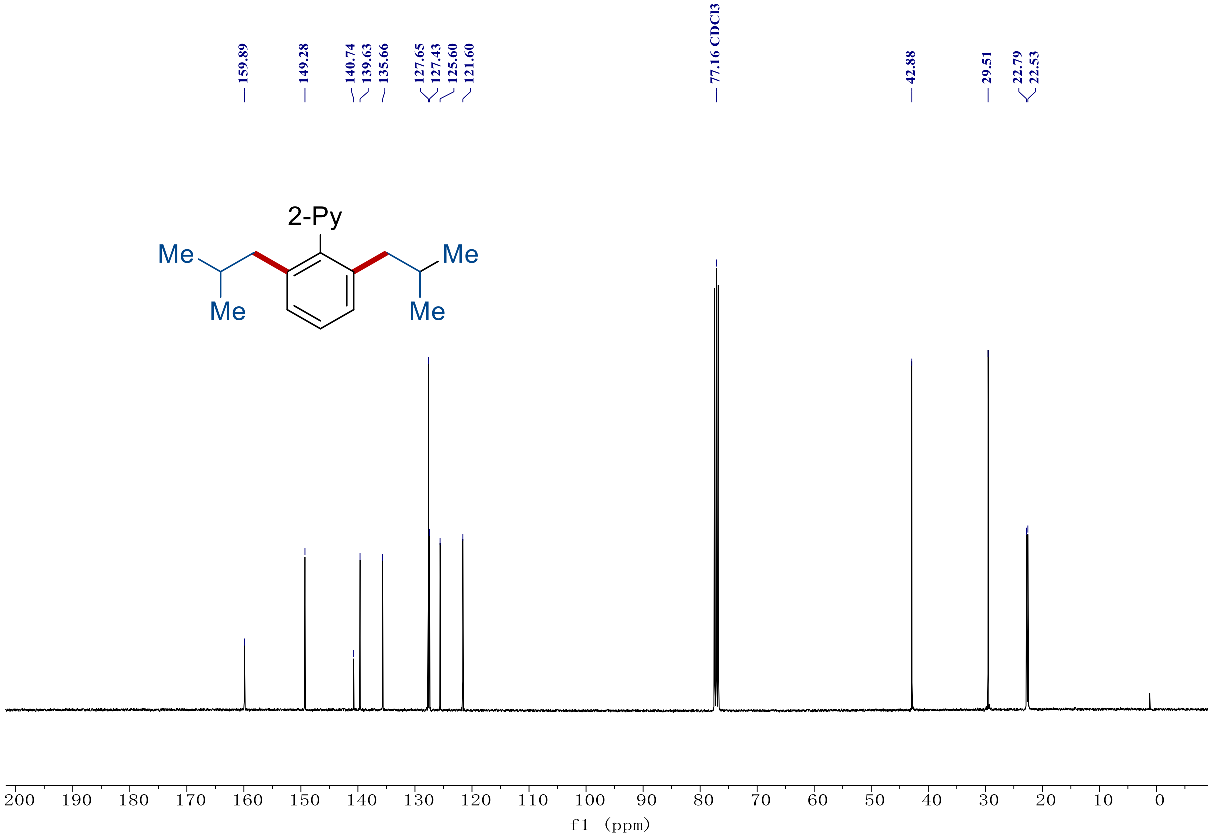

^{1}H NMR of **40a**, 300 MHz, $CDCl_3$, 25 °C.

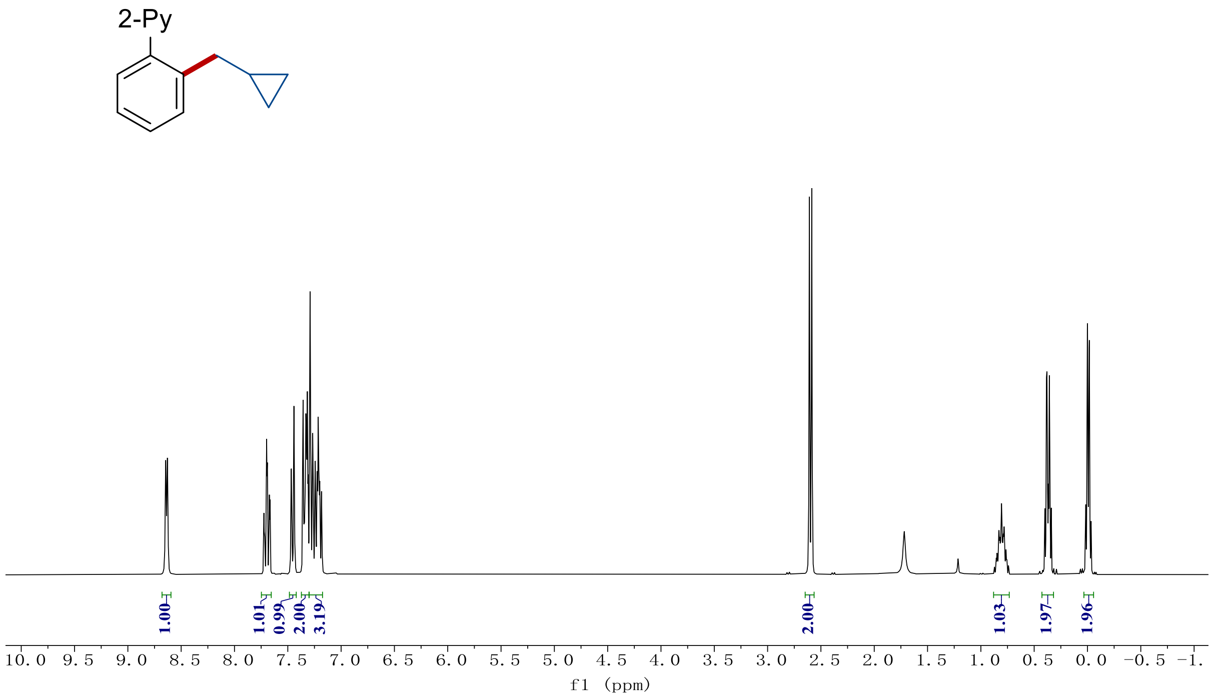

^{13}C NMR of **40a**, 75 MHz, $CDCl_3$, 25 °C.

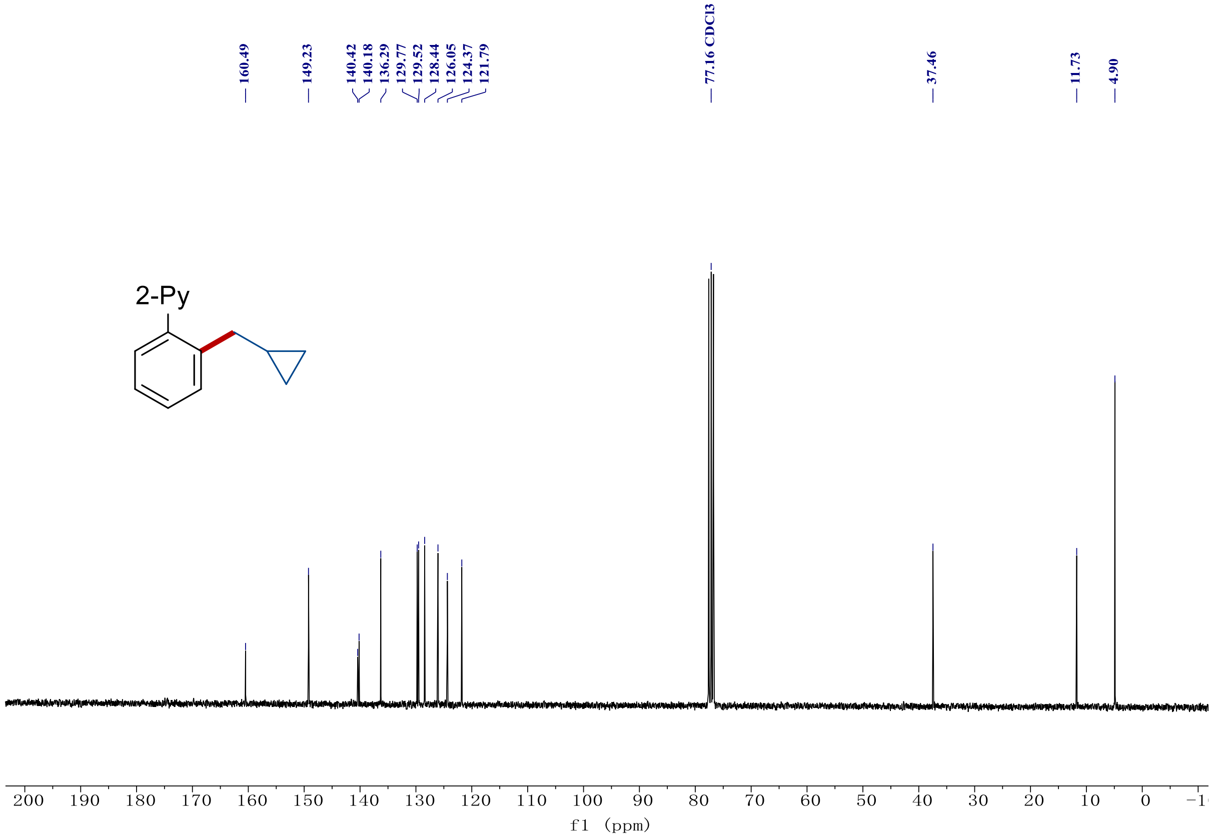

^{1}H NMR of **41a**, 400 MHz, $CDCl_3$, 25 °C.

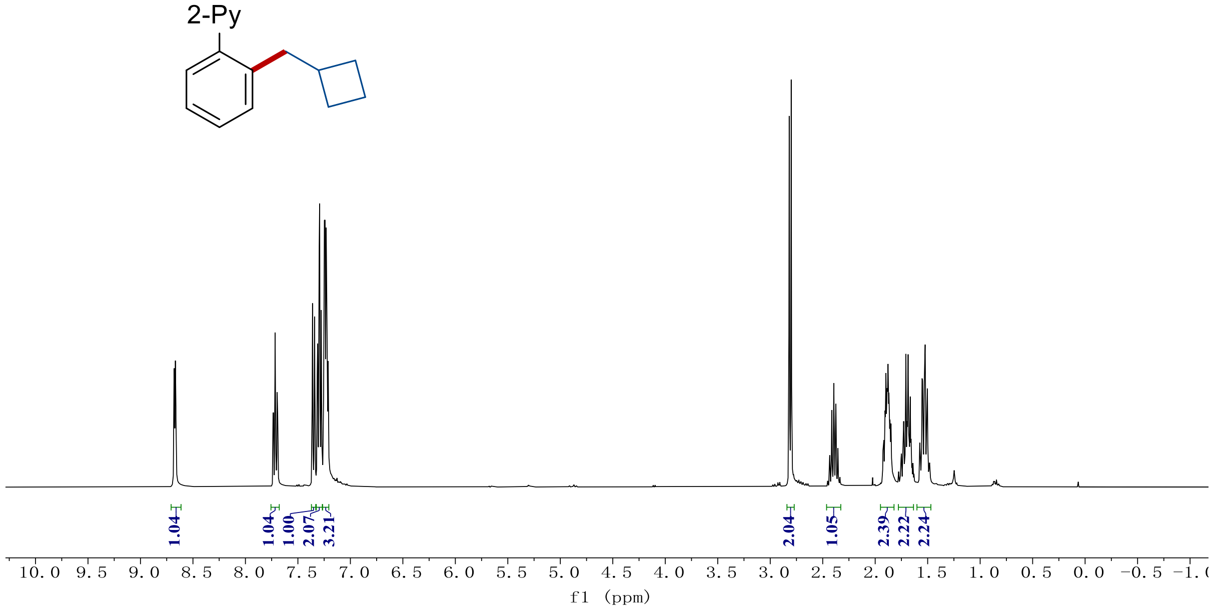

^{13}C NMR of **41a**, 101 MHz, $CDCl_3$, 25 °C.

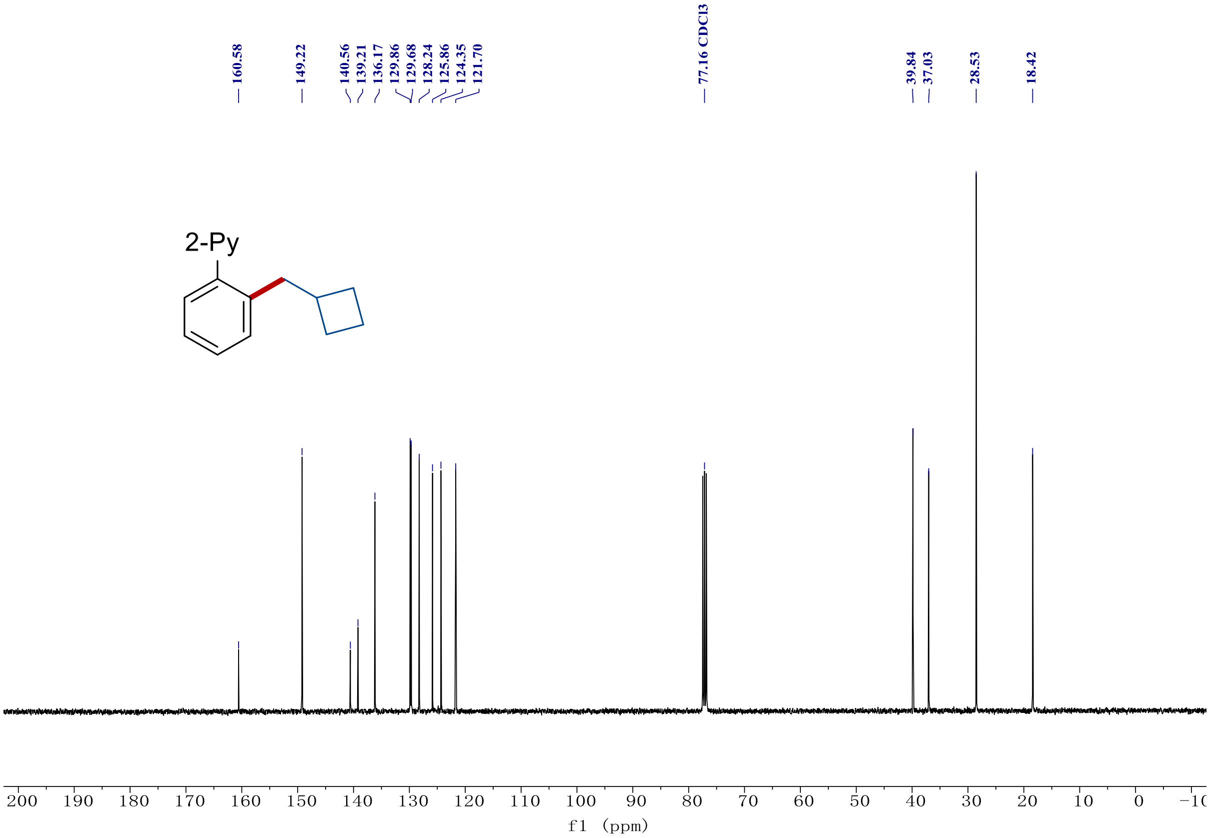

^{1}H NMR of **41b**, 400 MHz, $CDCl_3$, 25 °C.

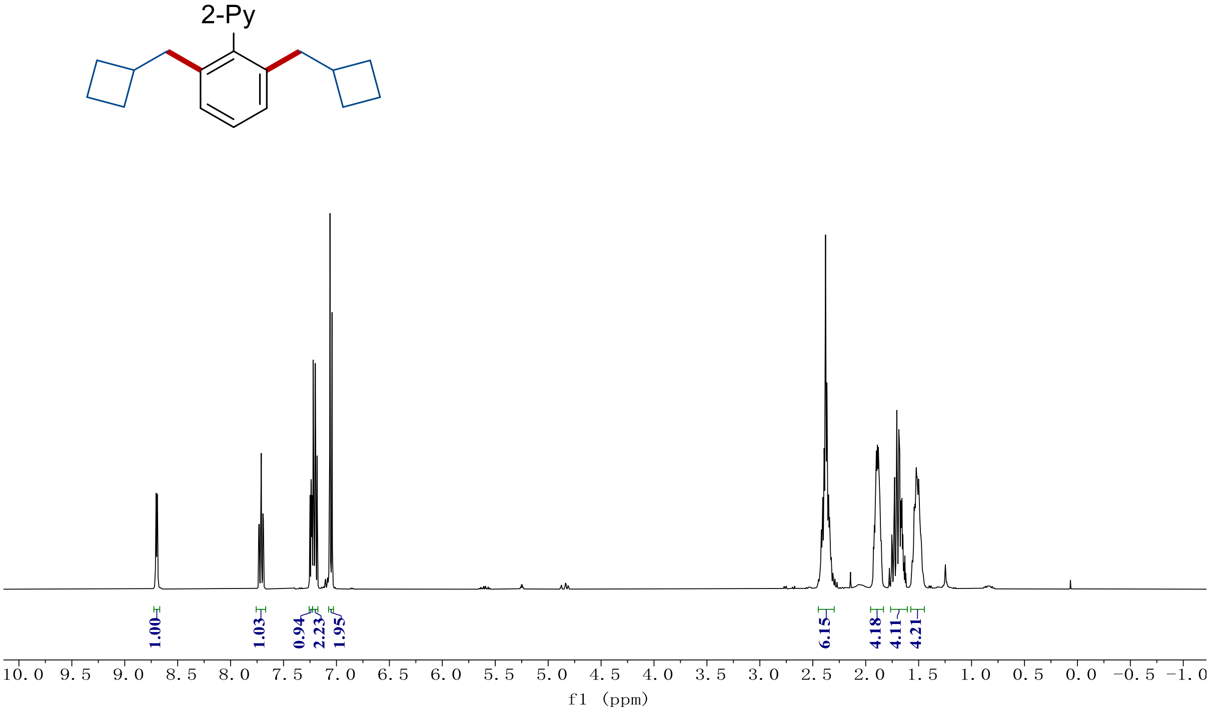

^{13}C NMR of **41b**, 101 MHz, $CDCl_3$, 25 °C.

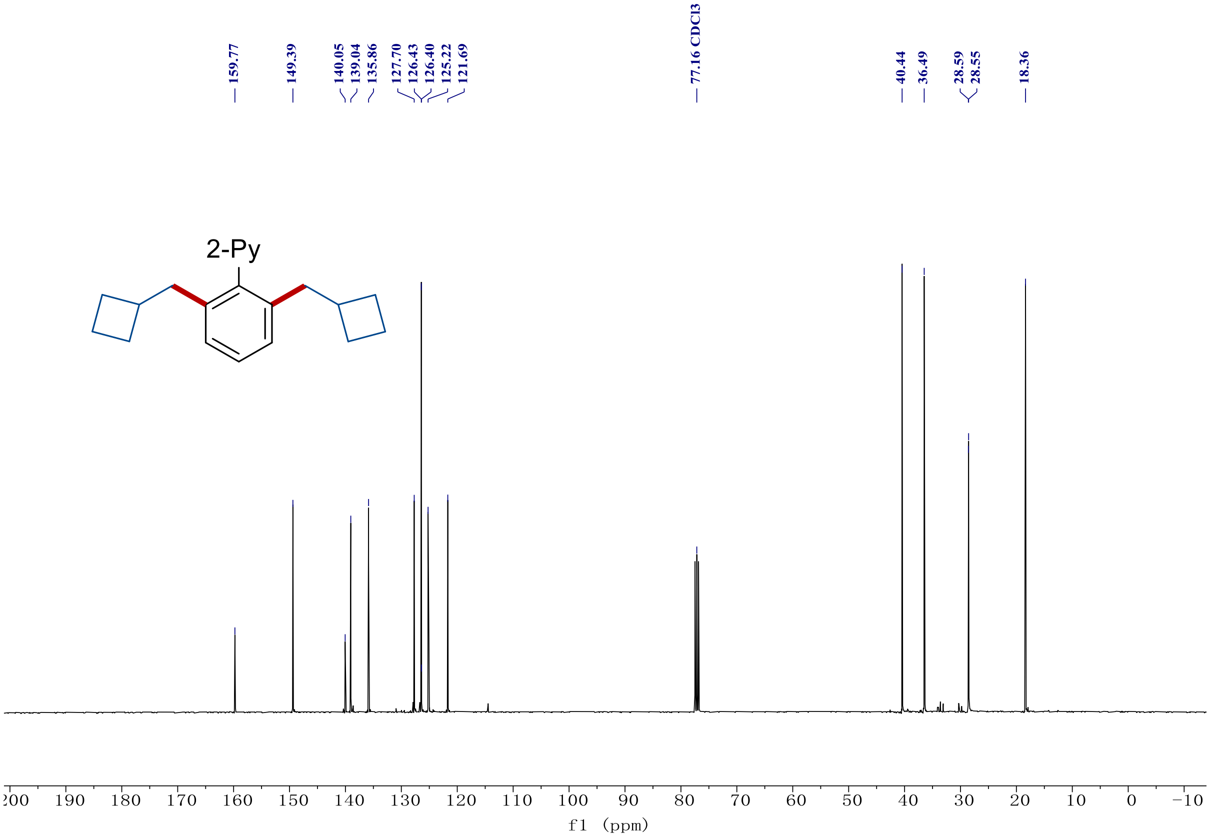

^{1}H NMR of **42**, 300 MHz, $CDCl_3$, 25 °C.

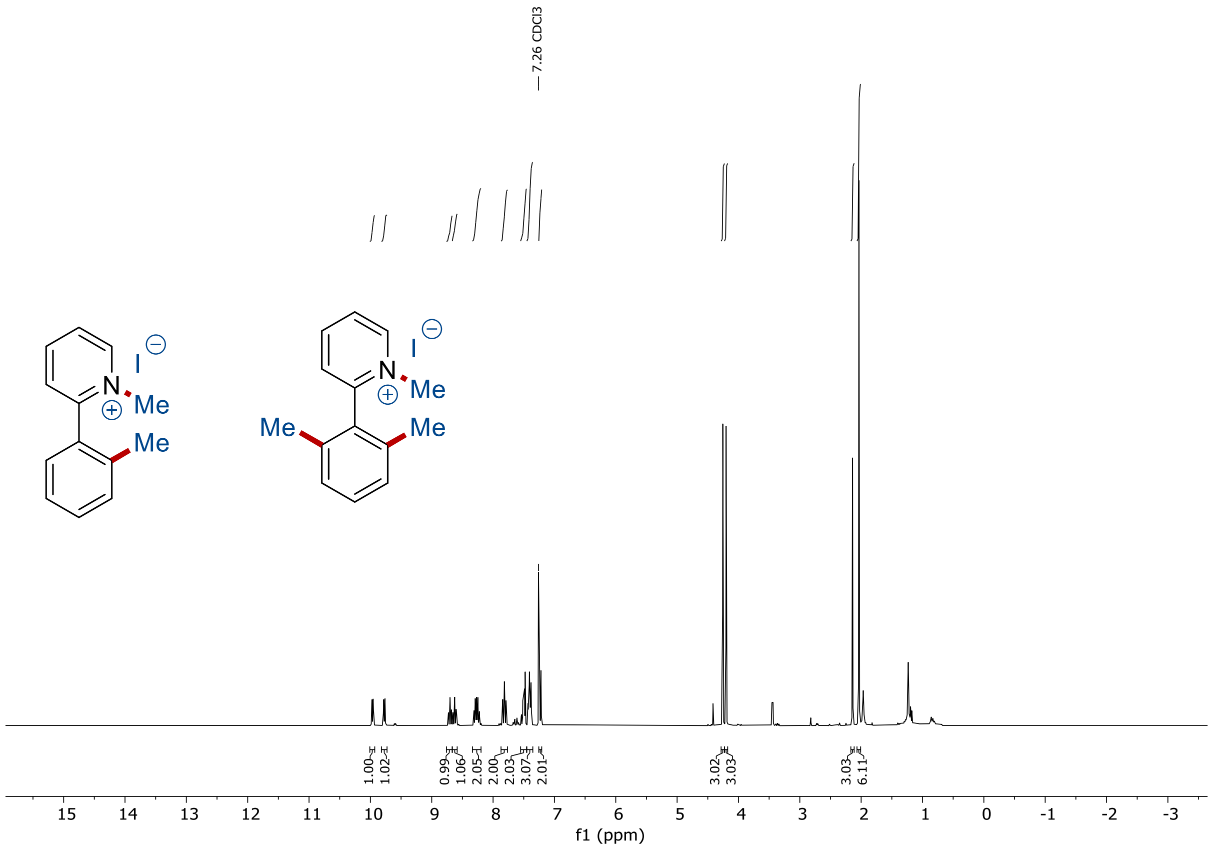

^{13}C NMR of **42**, 75 MHz, $CDCl_3$, 25 °C.

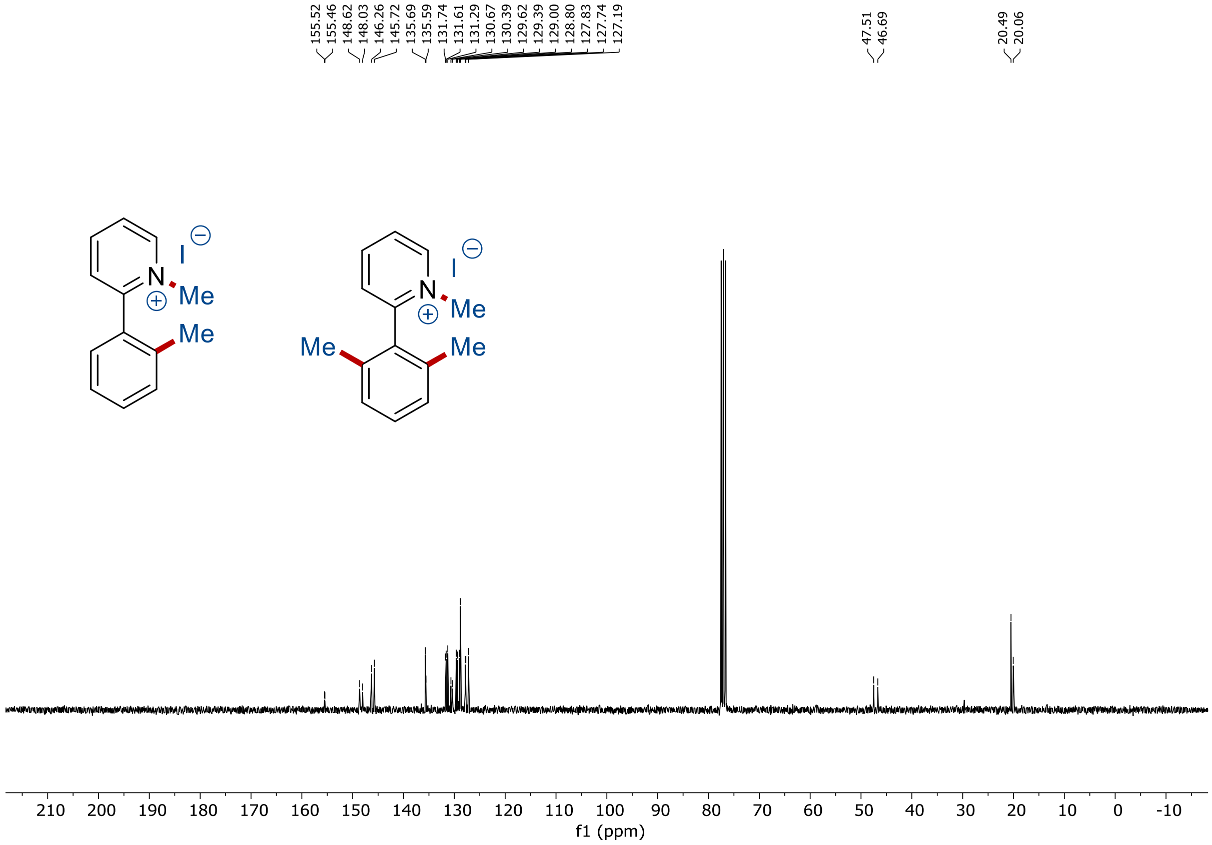

^{1}H NMR of **43**, 400 MHz, $CDCl_3$, 25 °C.

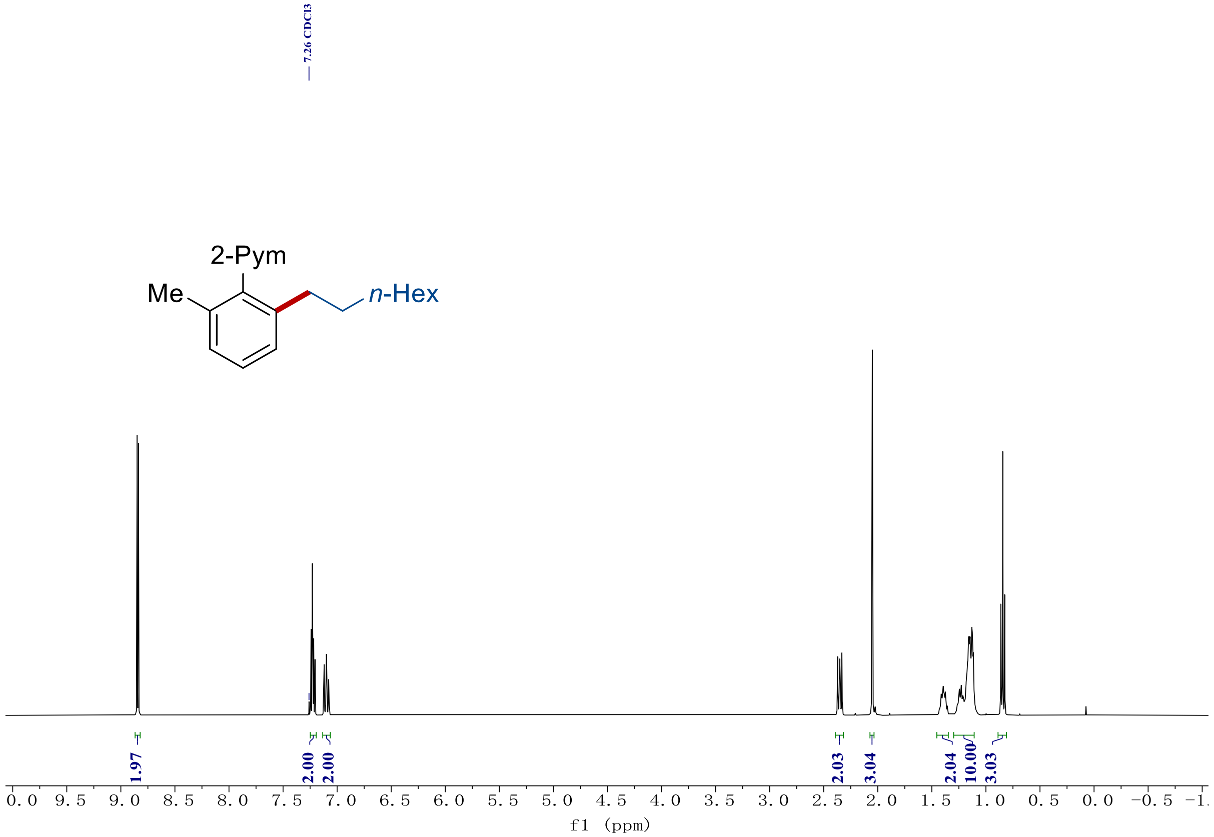

^{13}C NMR of **43**, 101 MHz, $CDCl_3$, 25 °C.

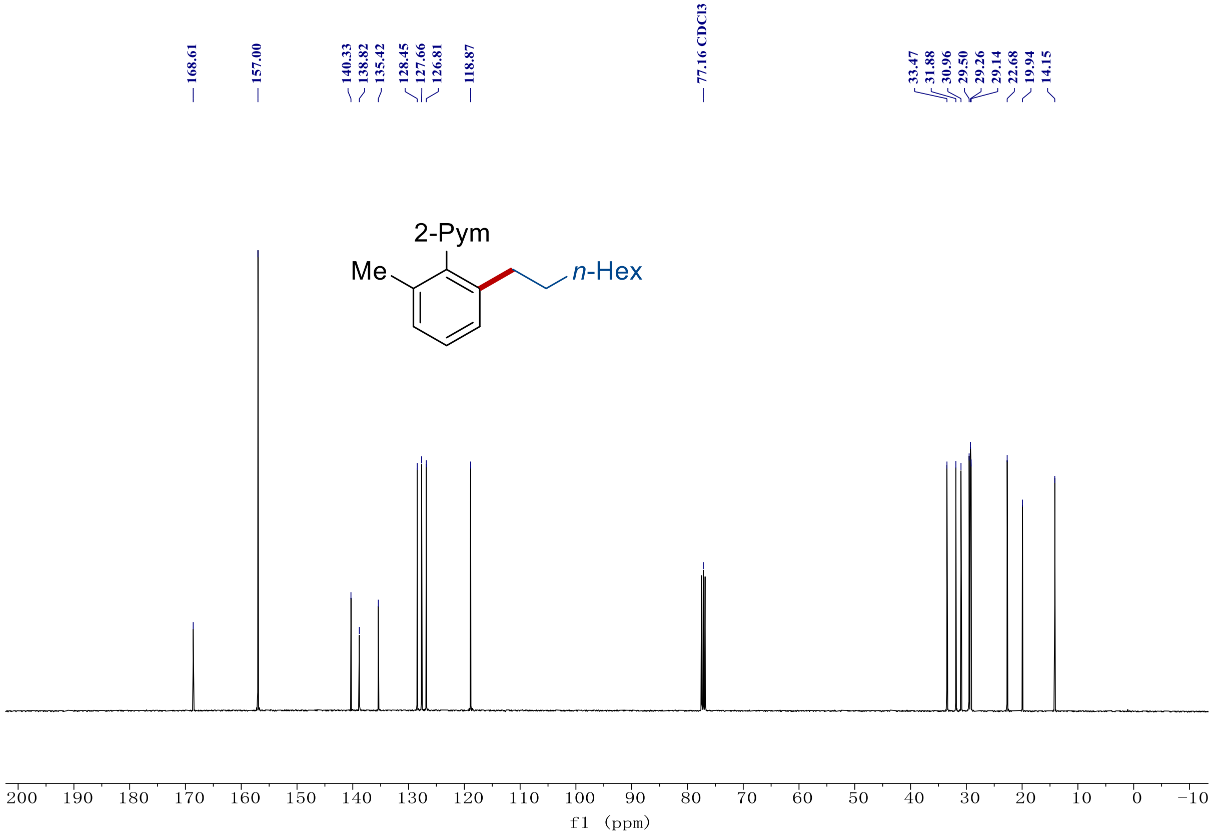

^{1}H NMR of **44a**, 300 MHz, $CDCl_3$, 25 °C.

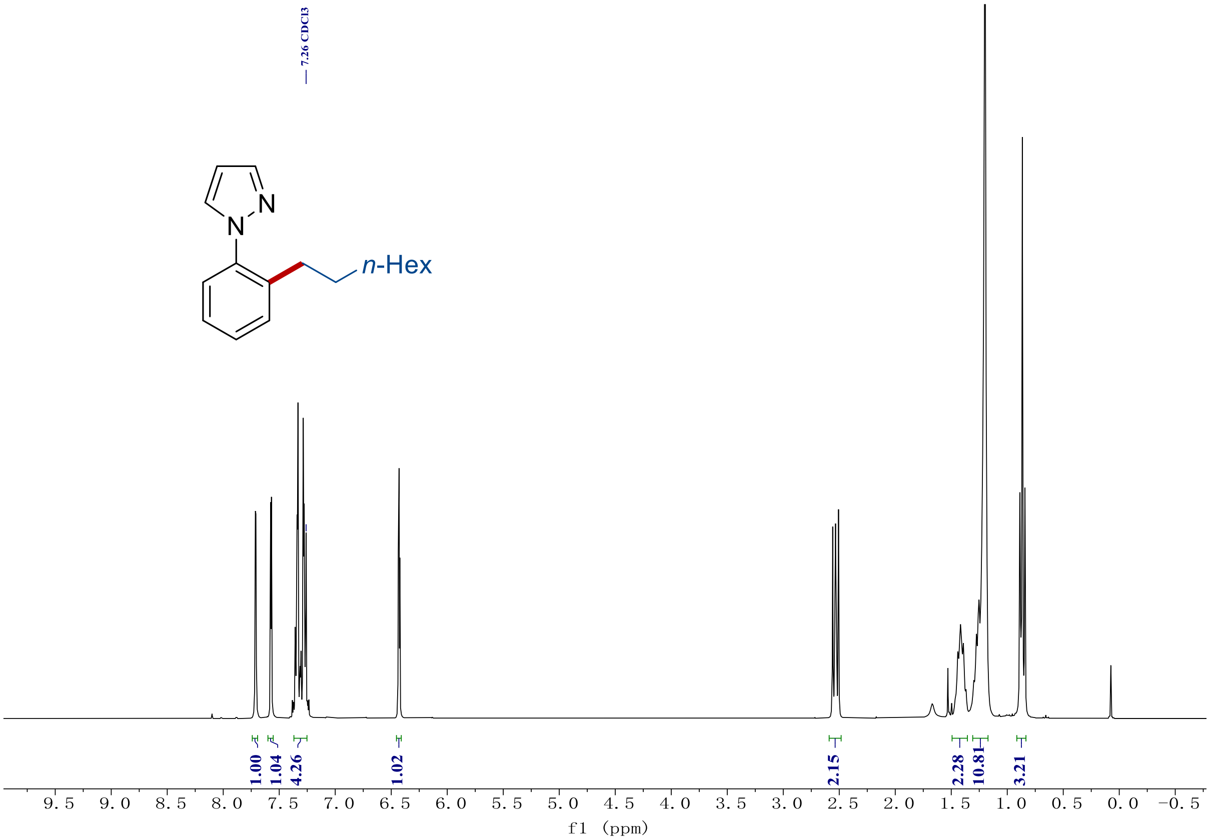

^{13}C NMR of **44a**, 75 MHz, $CDCl_3$, 25 °C.

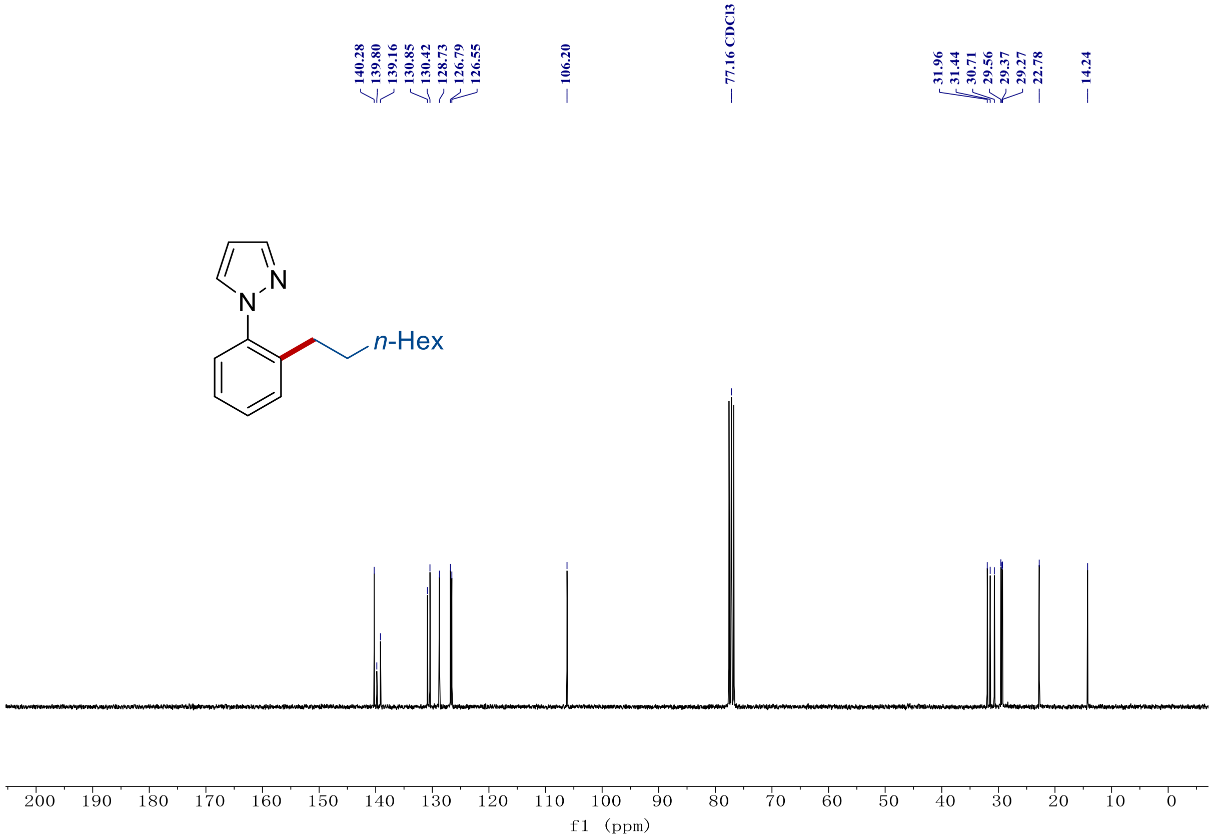

^{1}H NMR of **44b**, 300 MHz, $CDCl_3$, 25 °C.

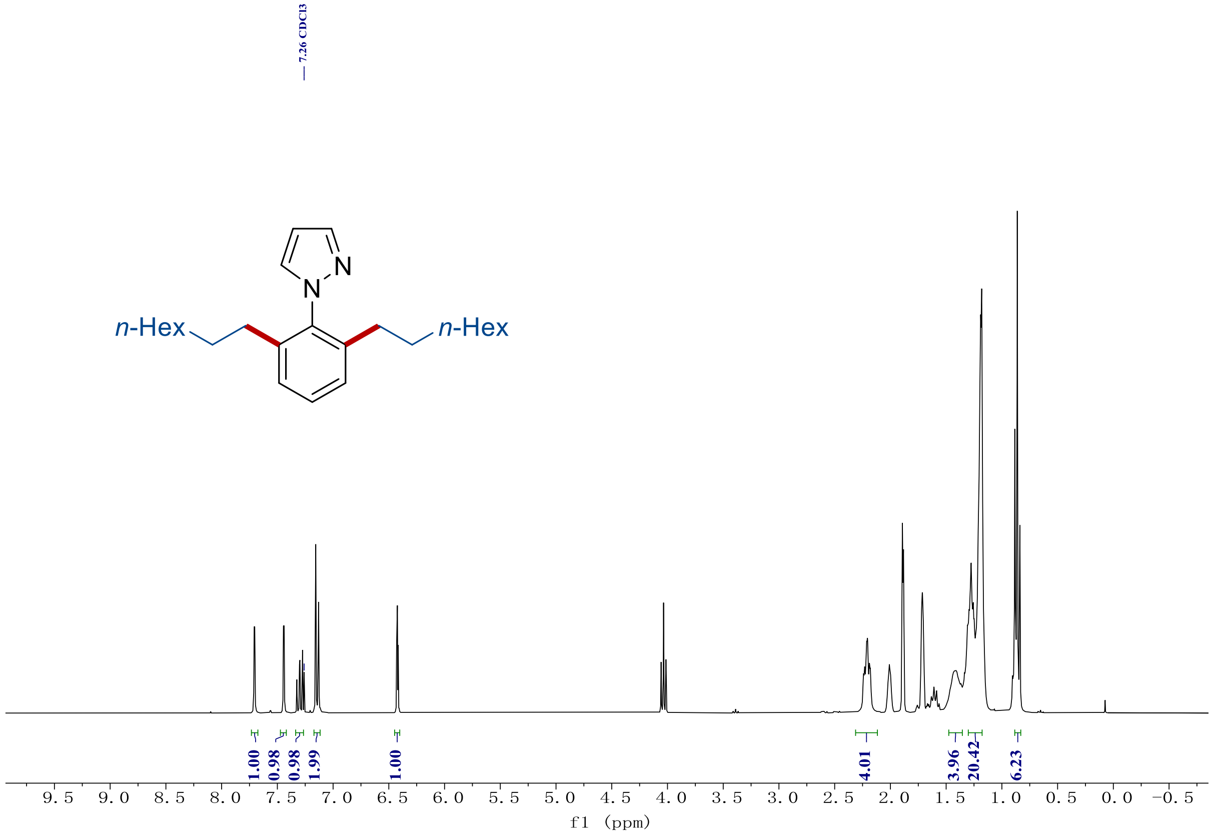

^{13}C NMR of **44b**, 75 MHz, $CDCl_3$, 25 °C.

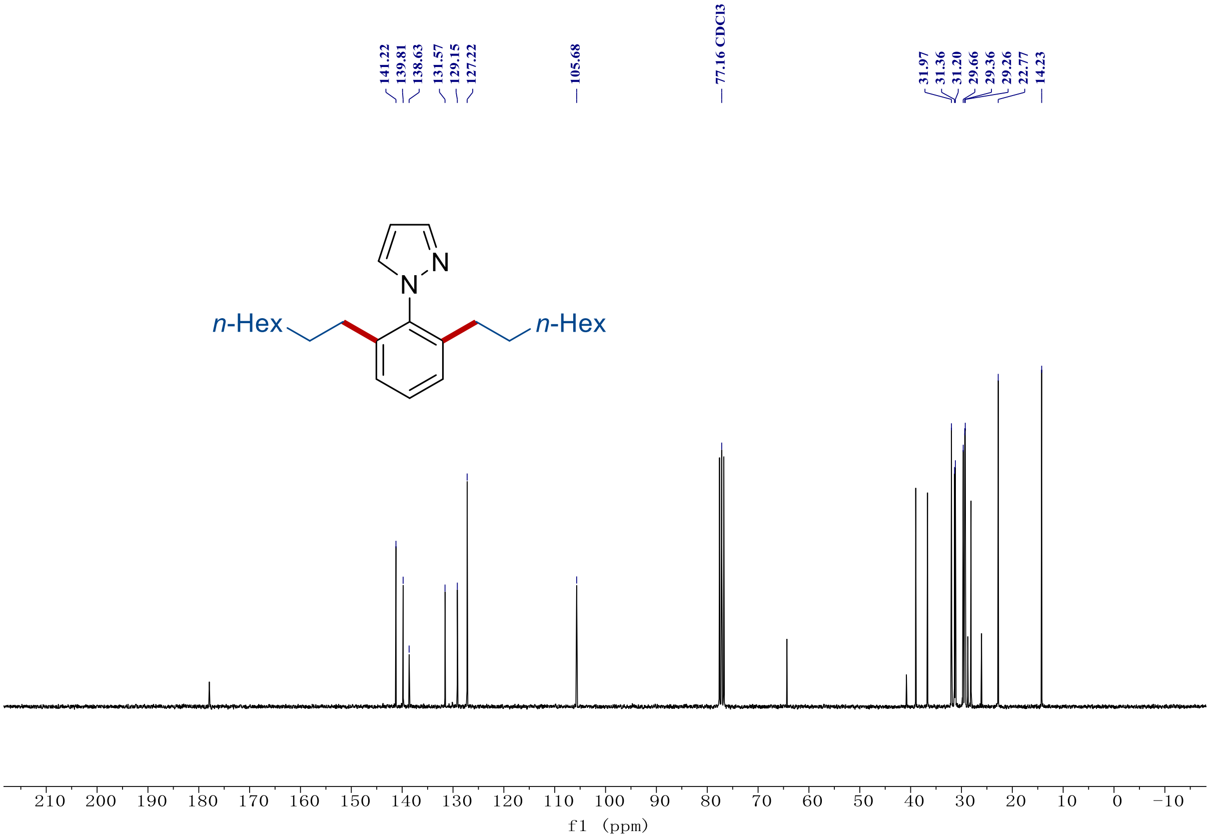

^{1}H NMR of **45a**, 300 MHz, $CDCl_3$, 25 °C.

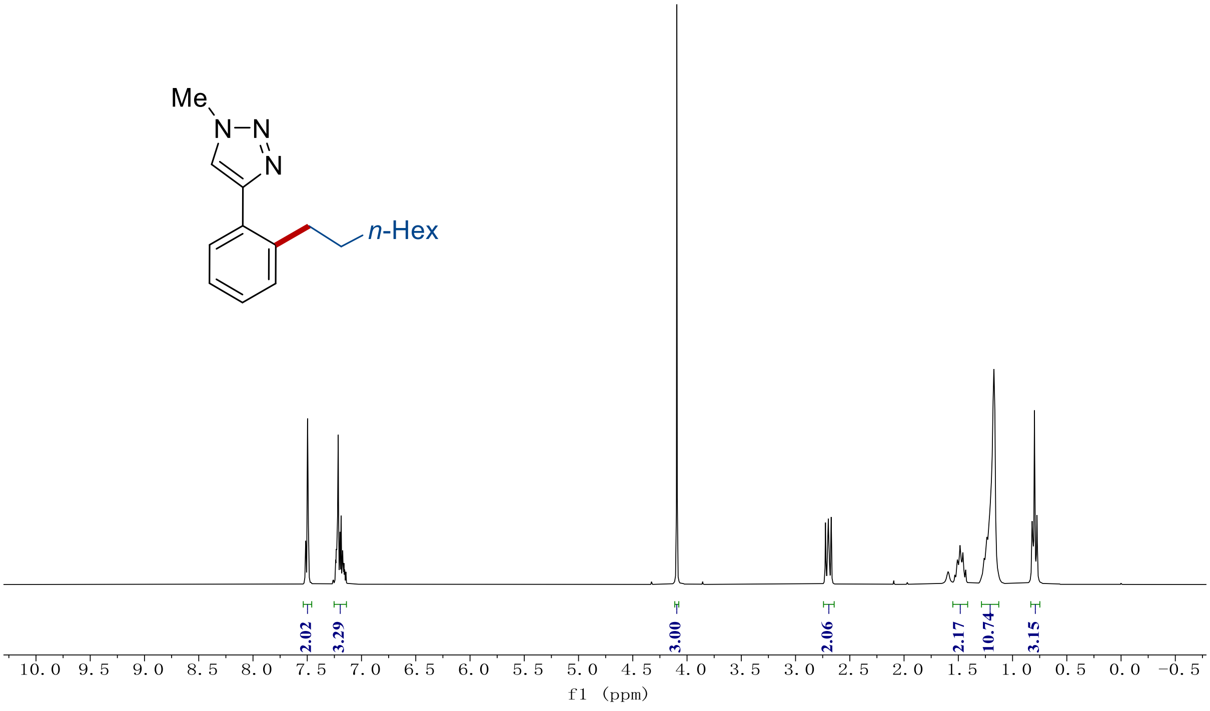

^{13}C NMR of **45a**, 75 MHz, $CDCl_3$, 25 °C.

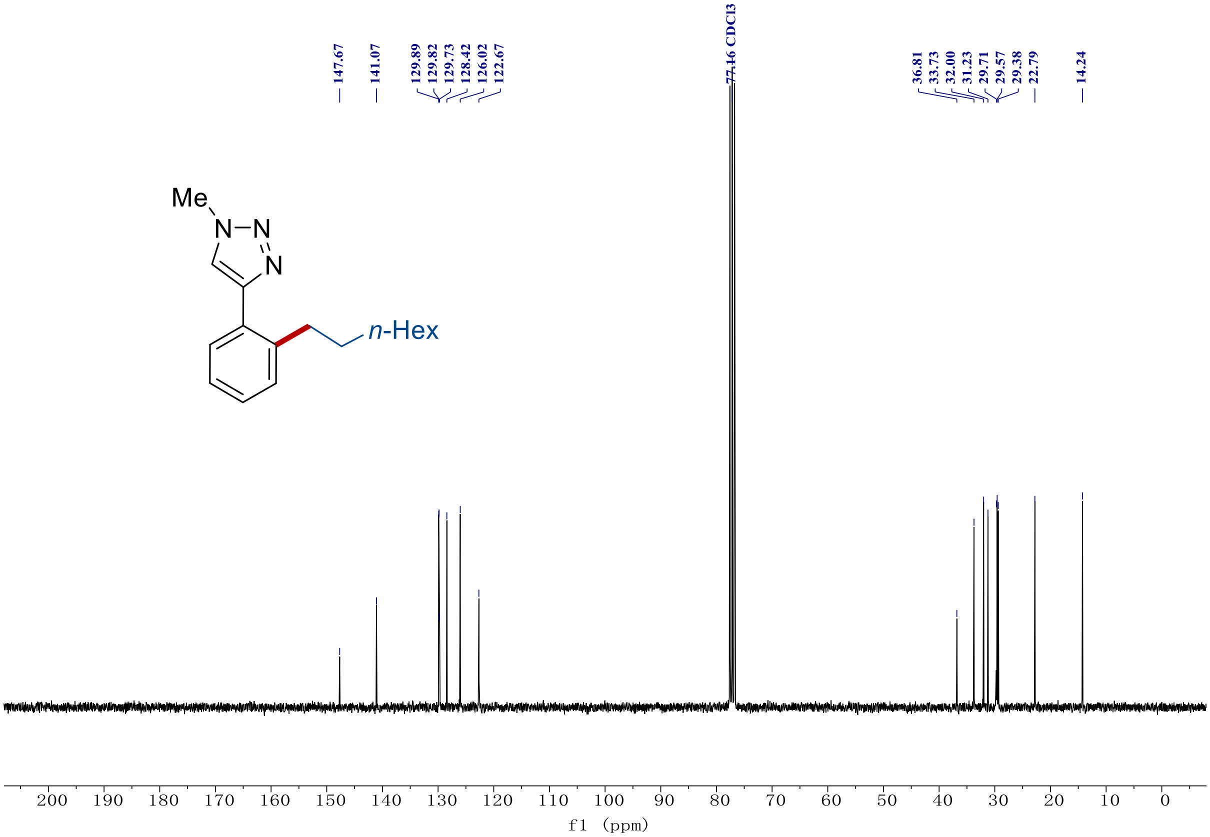

^{1}H NMR of **46a**, 300 MHz, $CDCl_3$, 25 °C.

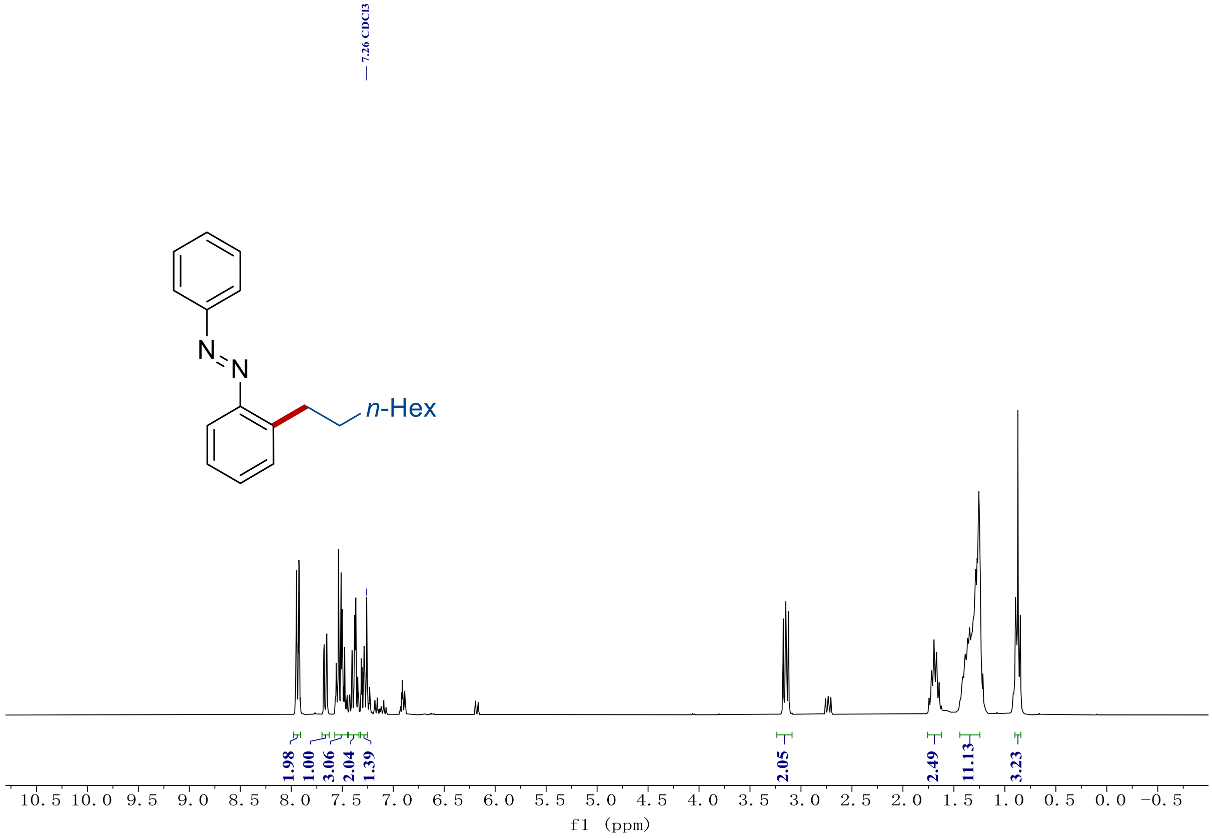

^{13}C NMR of **46a**, 75 MHz, $CDCl_3$, 25 °C.

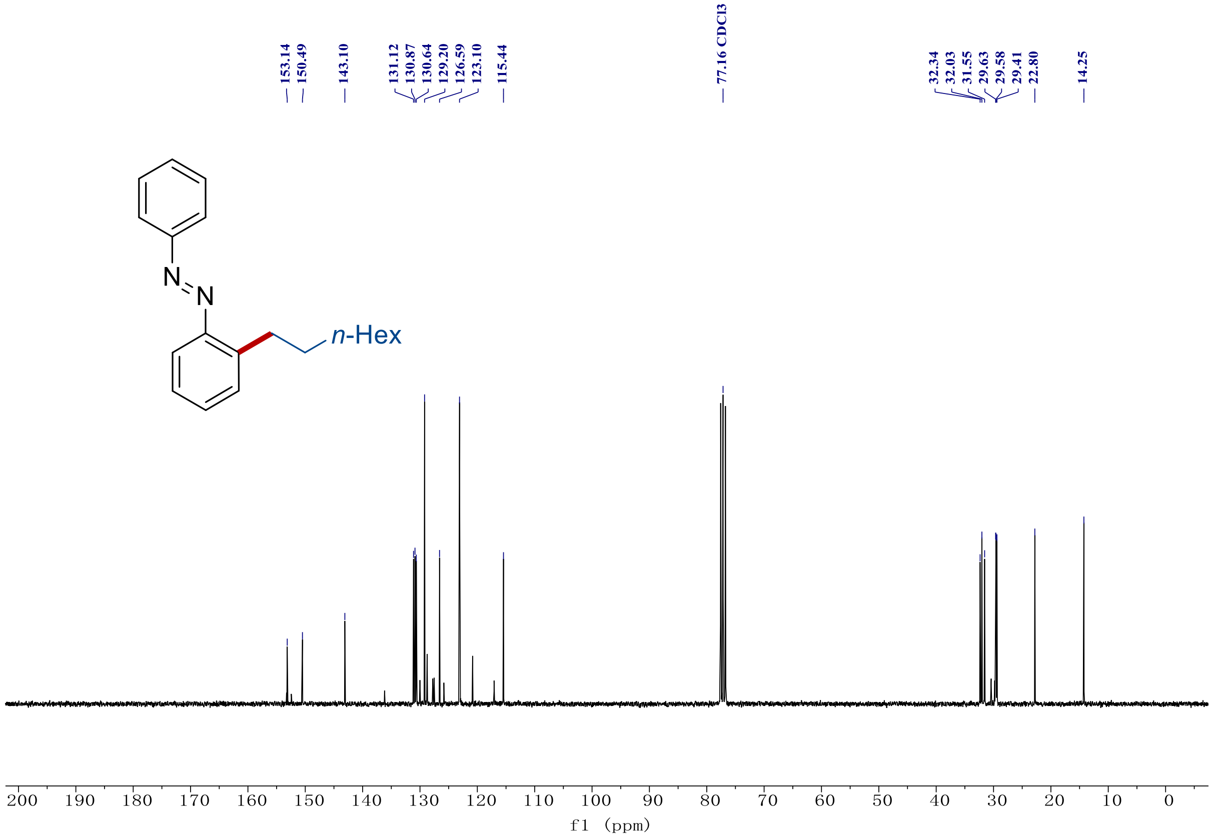

^{1}H NMR of **47a**, 300 MHz, $CDCl_3$, 25 °C.

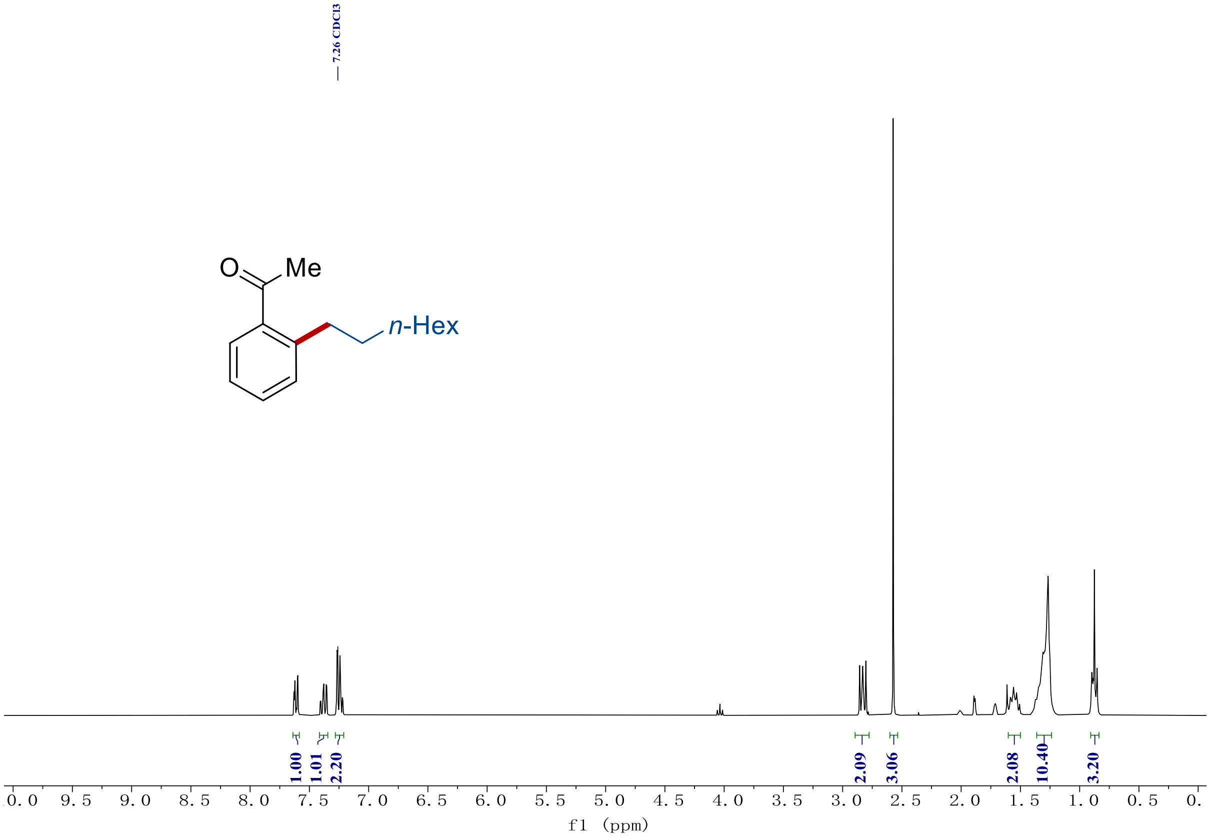

^{13}C NMR of **47a**, 75 MHz, $CDCl_3$, 25 °C.

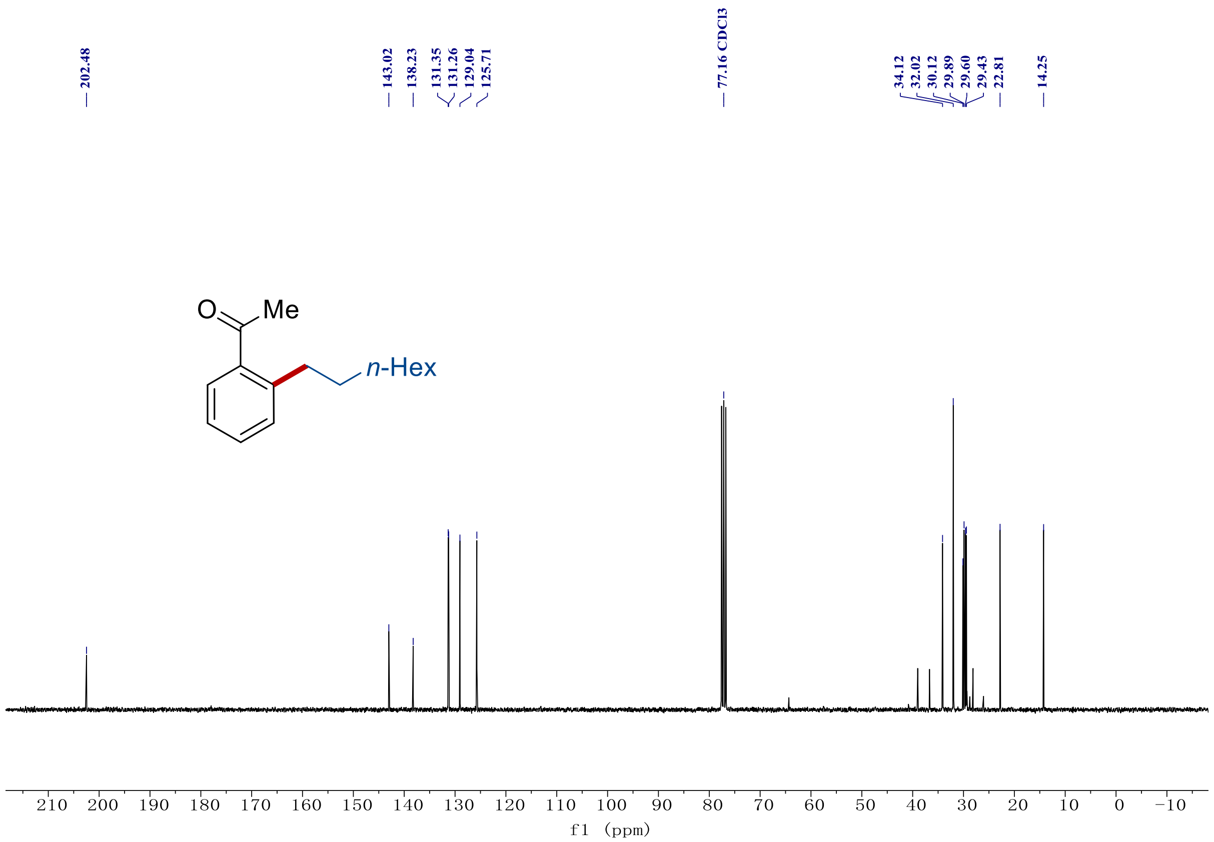

^{1}H NMR of **48a**, 300 MHz, $CDCl_3$, 25 °C.

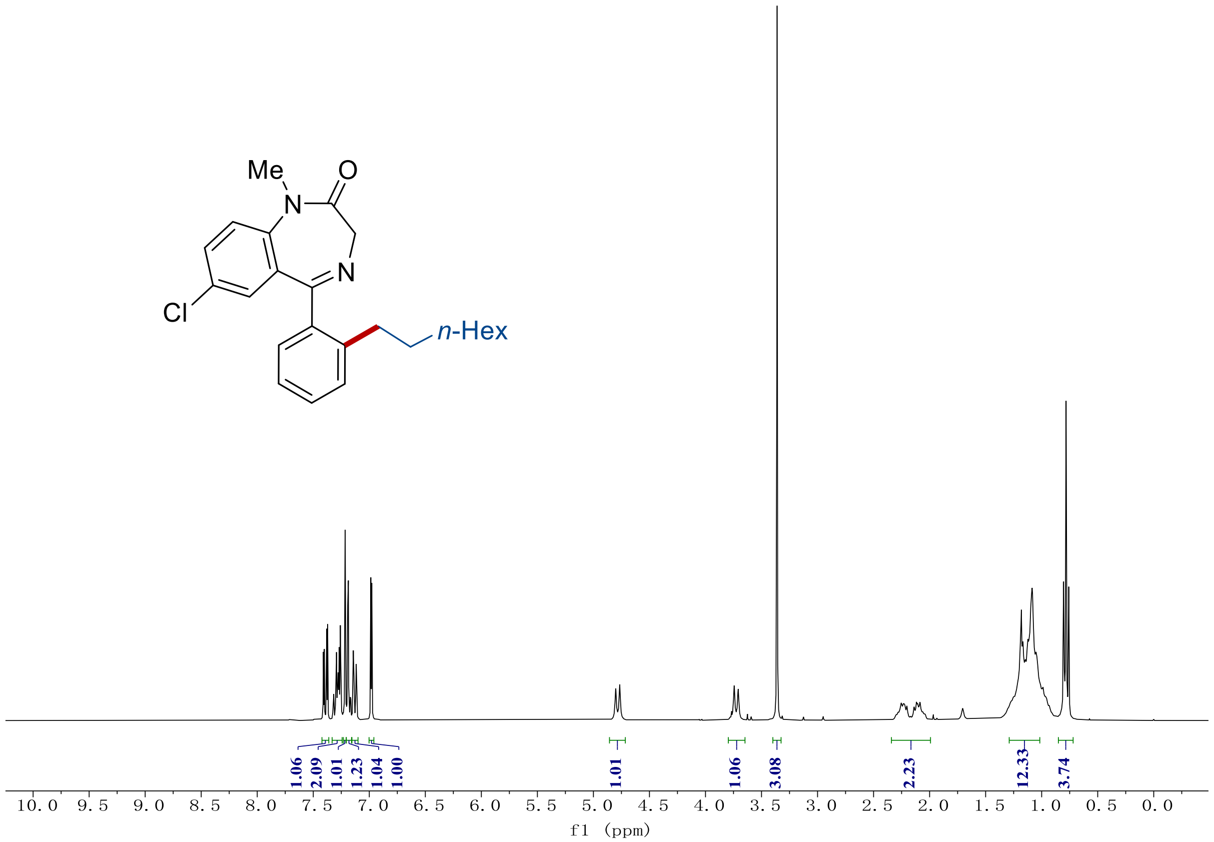

^{13}C NMR of **48a**, 75 MHz, $CDCl_3$, 25 °C.

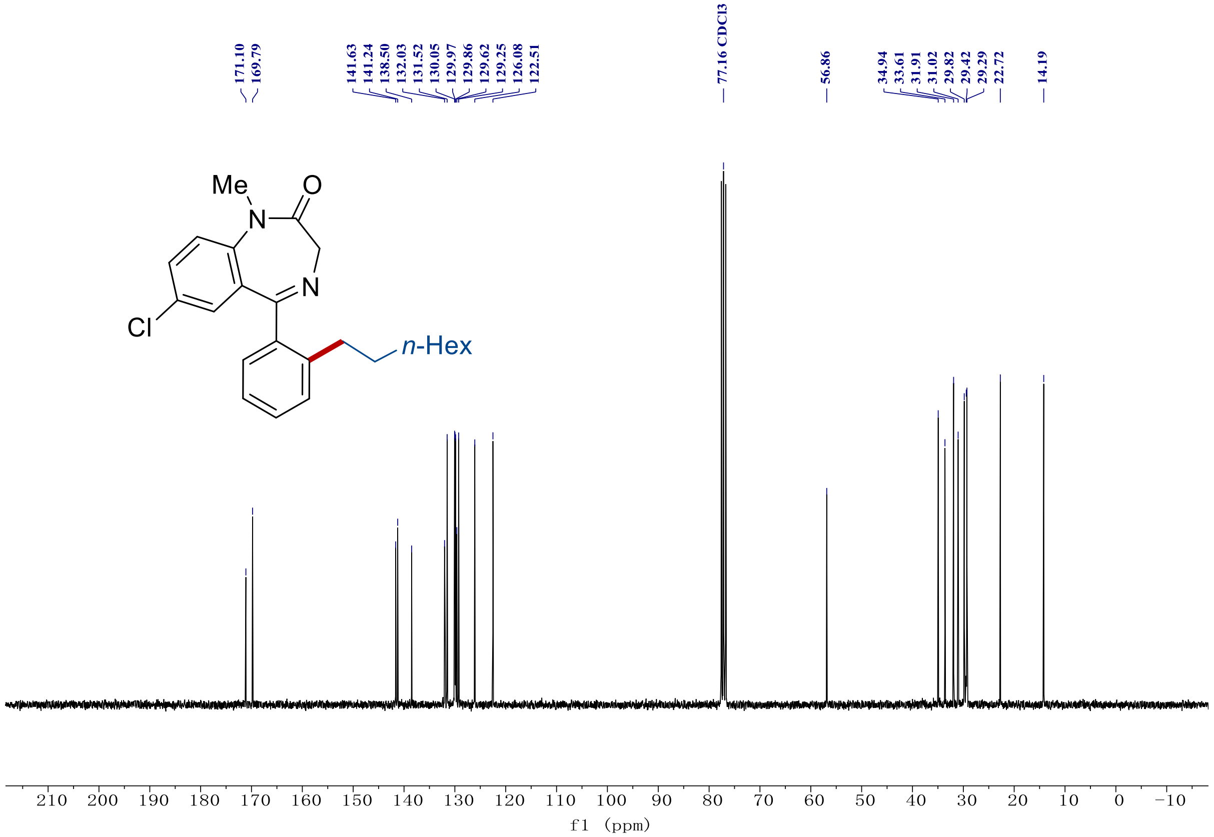

^{1}H NMR of **49a**, 300 MHz, $CDCl_3$, 25 °C.

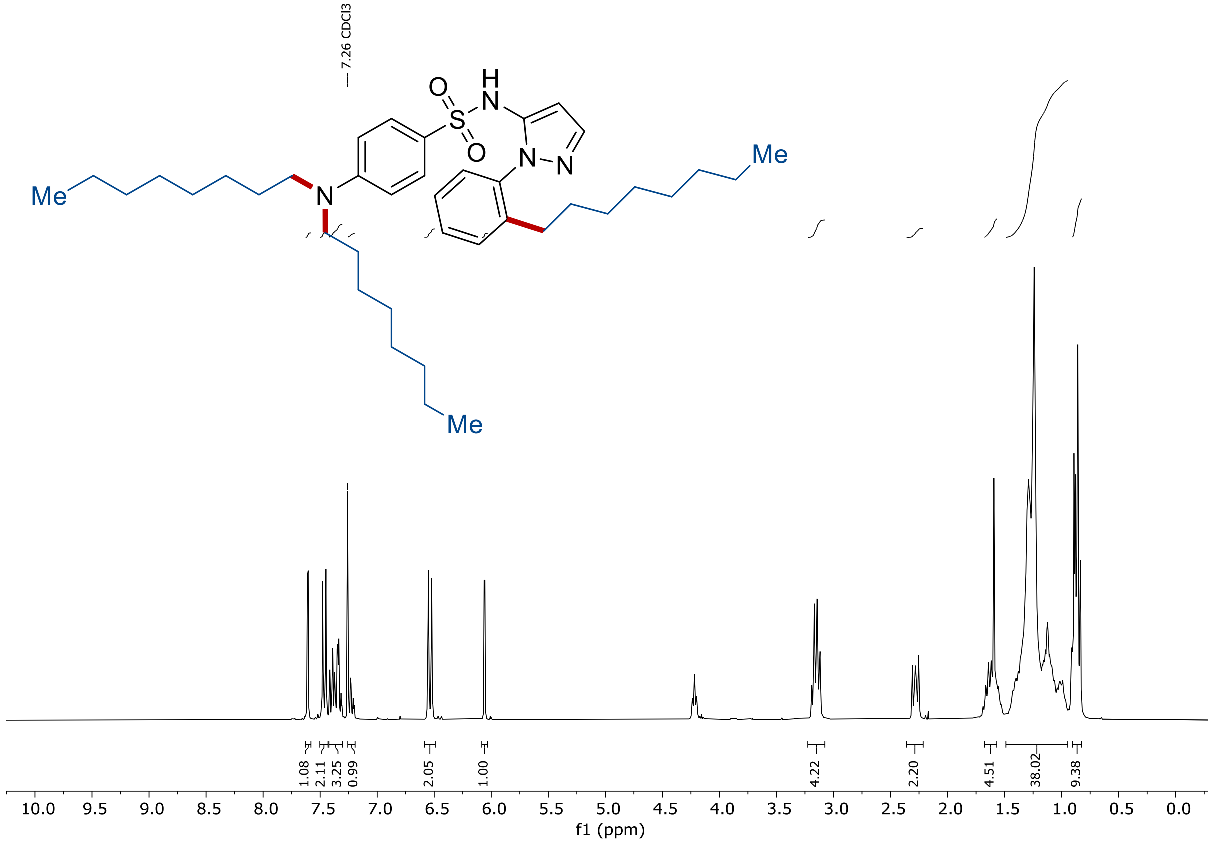

^{13}C NMR of **49a**, 75 MHz, $CDCl_3$, 25 °C.

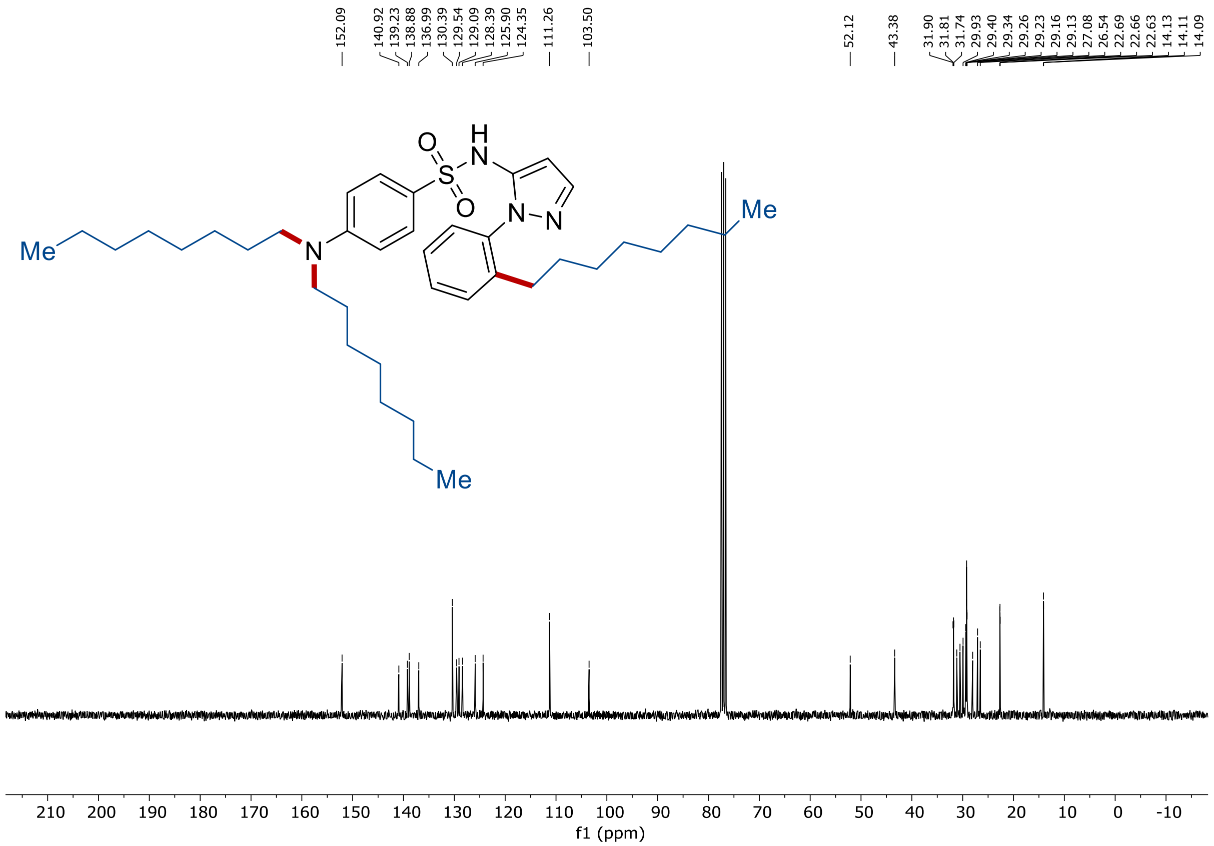

^{1}H NMR of **49b**, 300 MHz, $CDCl_3$, 25 °C.

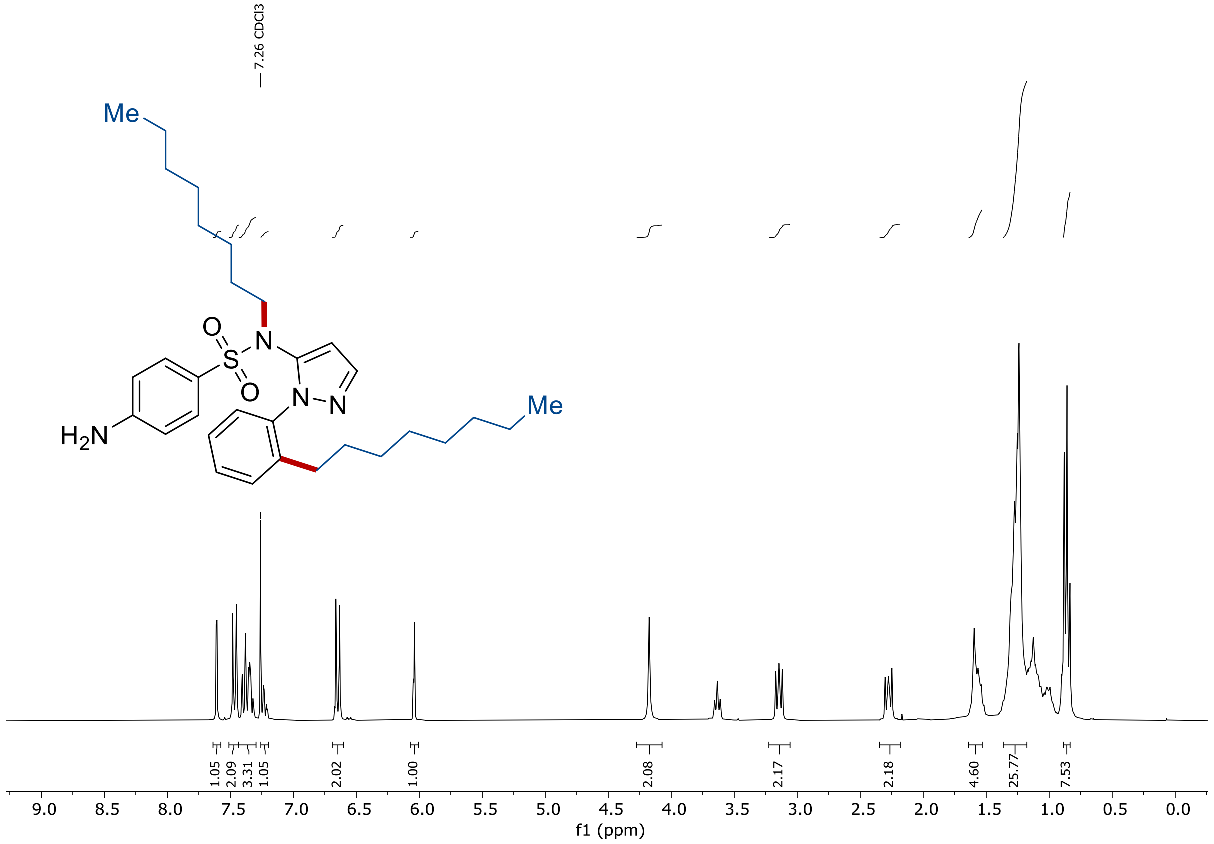

^{13}C NMR of **49b**, 75 MHz, $CDCl_3$, 25 °C.

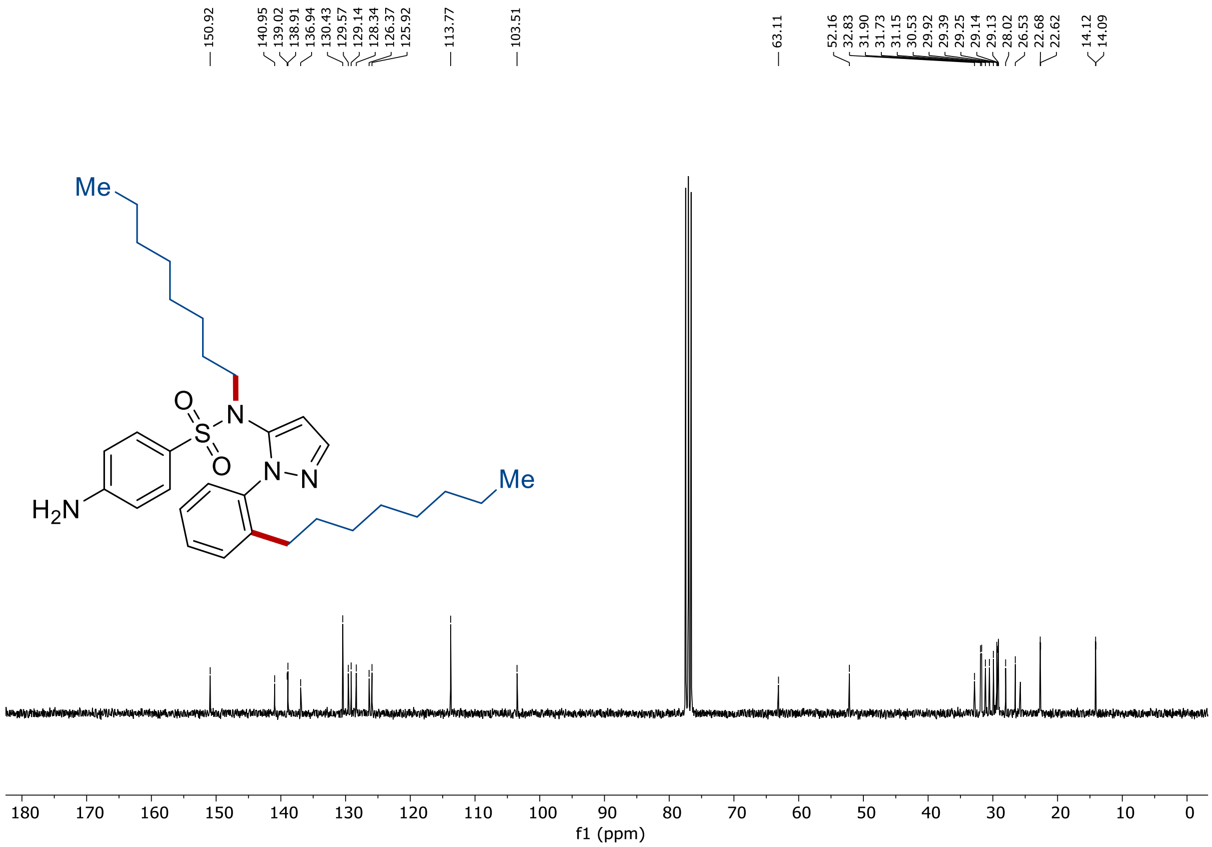

^{1}H NMR of **52a**, 400 MHz, $CDCl_3$, 25 °C.

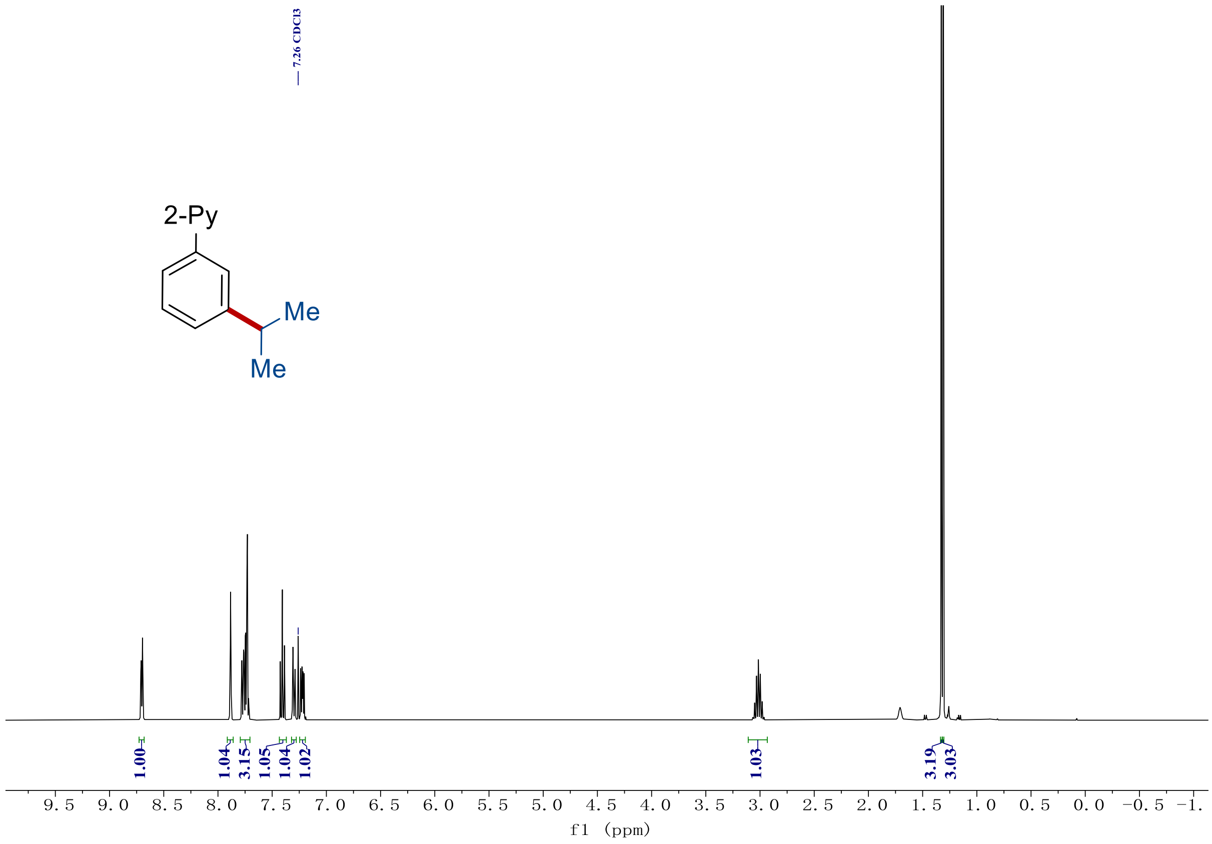

^{13}C NMR of **52a**, 101 MHz, $CDCl_3$, 25 °C.

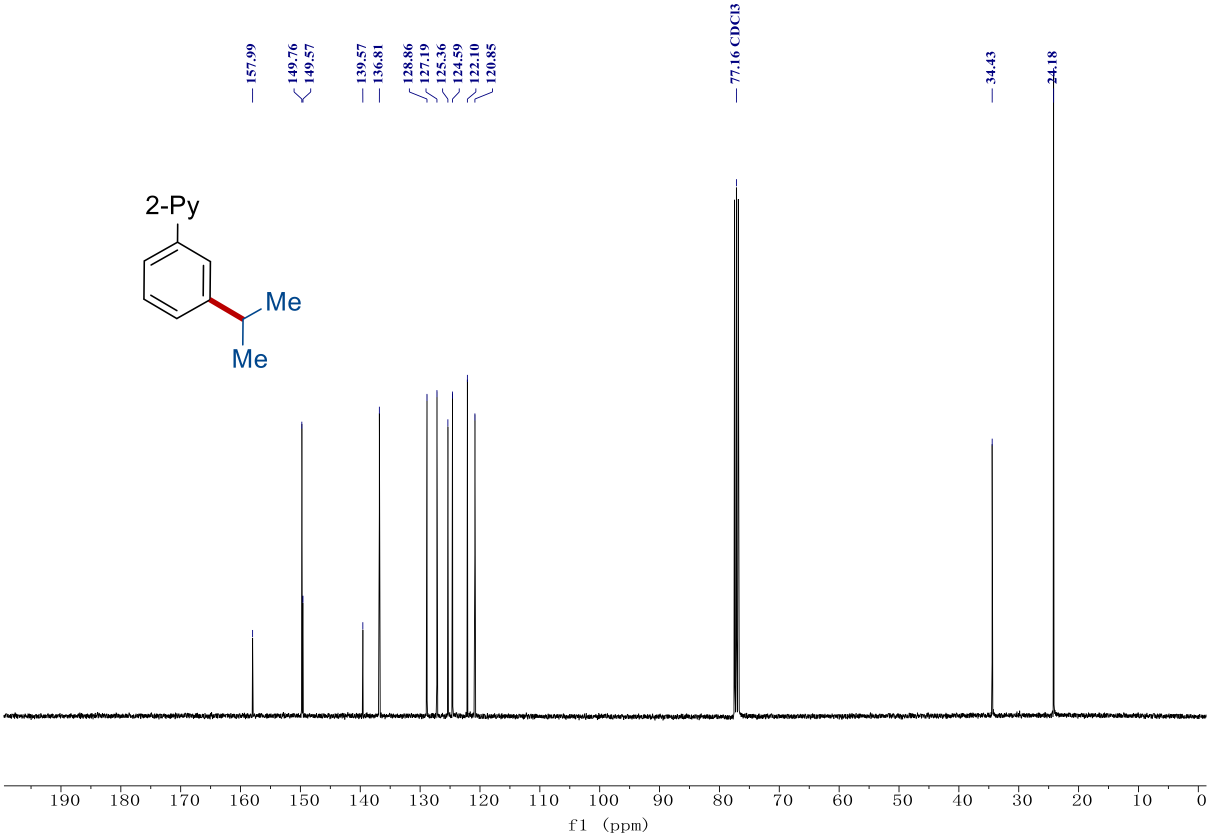

^{1}H NMR of **26a**, 500 MHz, $CDCl_3$, 25 °C.

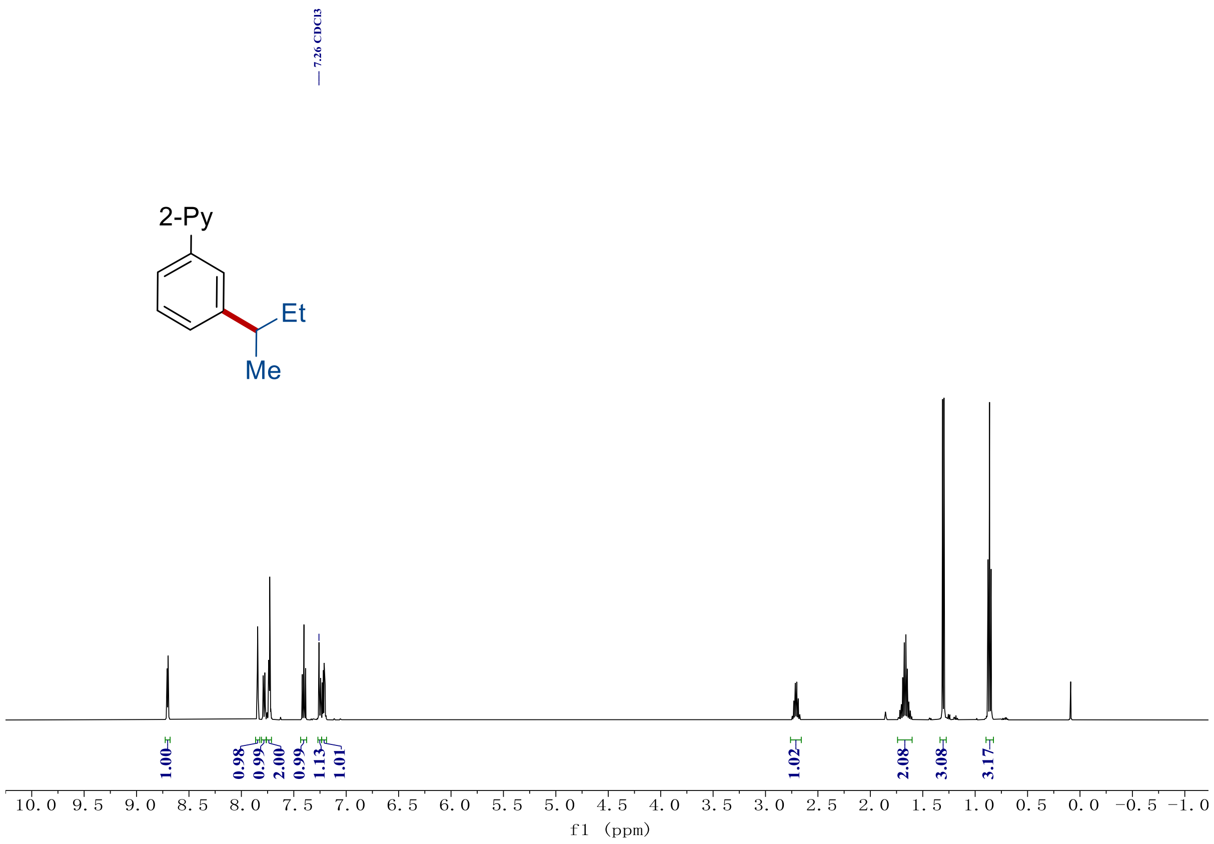

^{13}C NMR of **26a**, 126 MHz, $CDCl_3$, 25 °C.

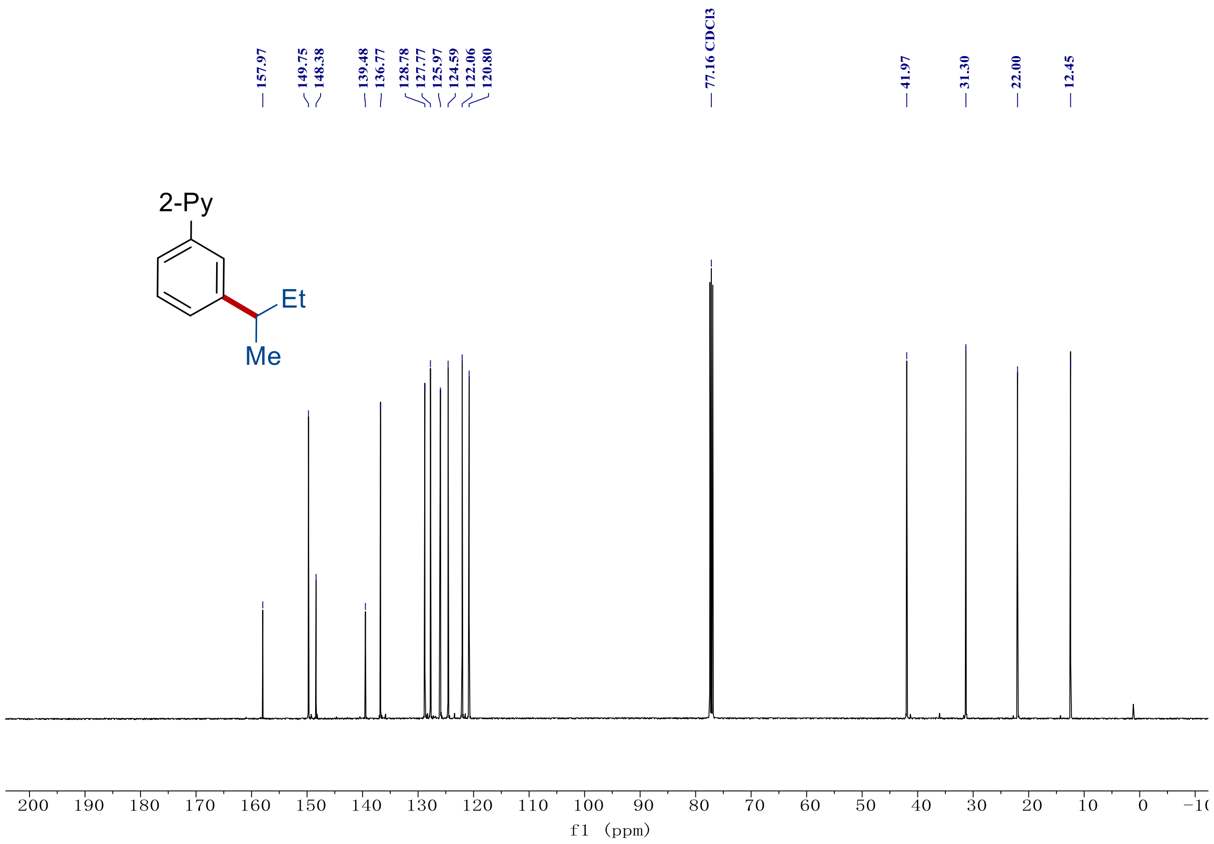

^{1}H NMR of **53a**, 500 MHz, $CDCl_3$, 25 °C.

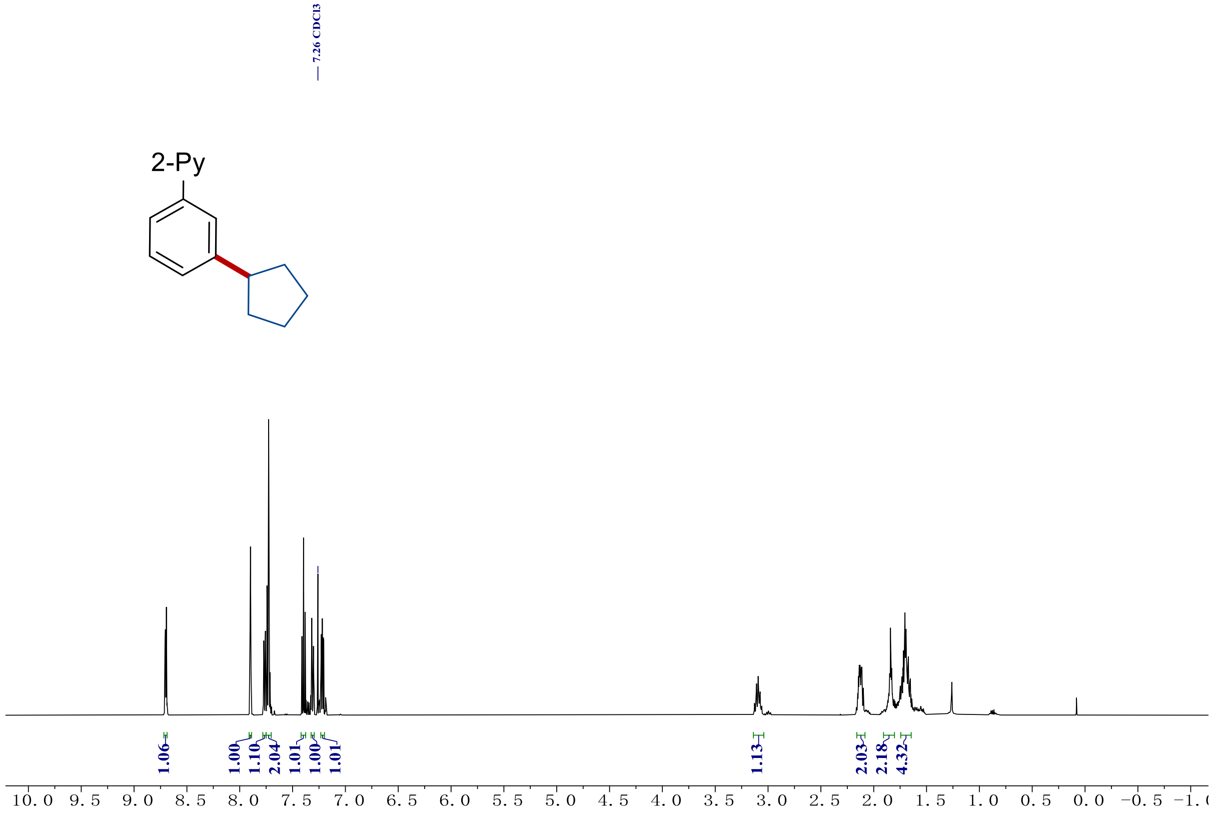

^{13}C NMR of **53a**, 126 MHz, $CDCl_3$, 25 °C.

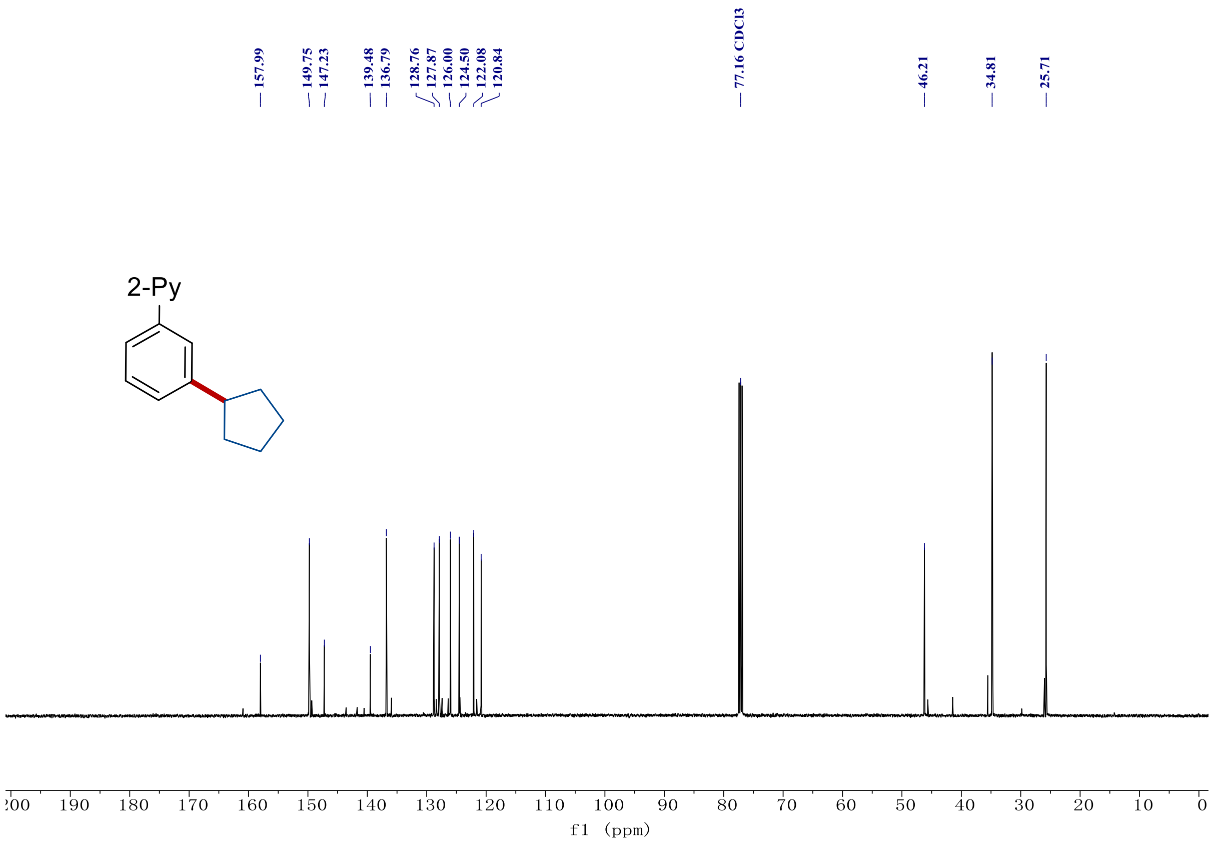

^{1}H NMR of **54a**, 500 MHz, $CDCl_3$, 25 °C.

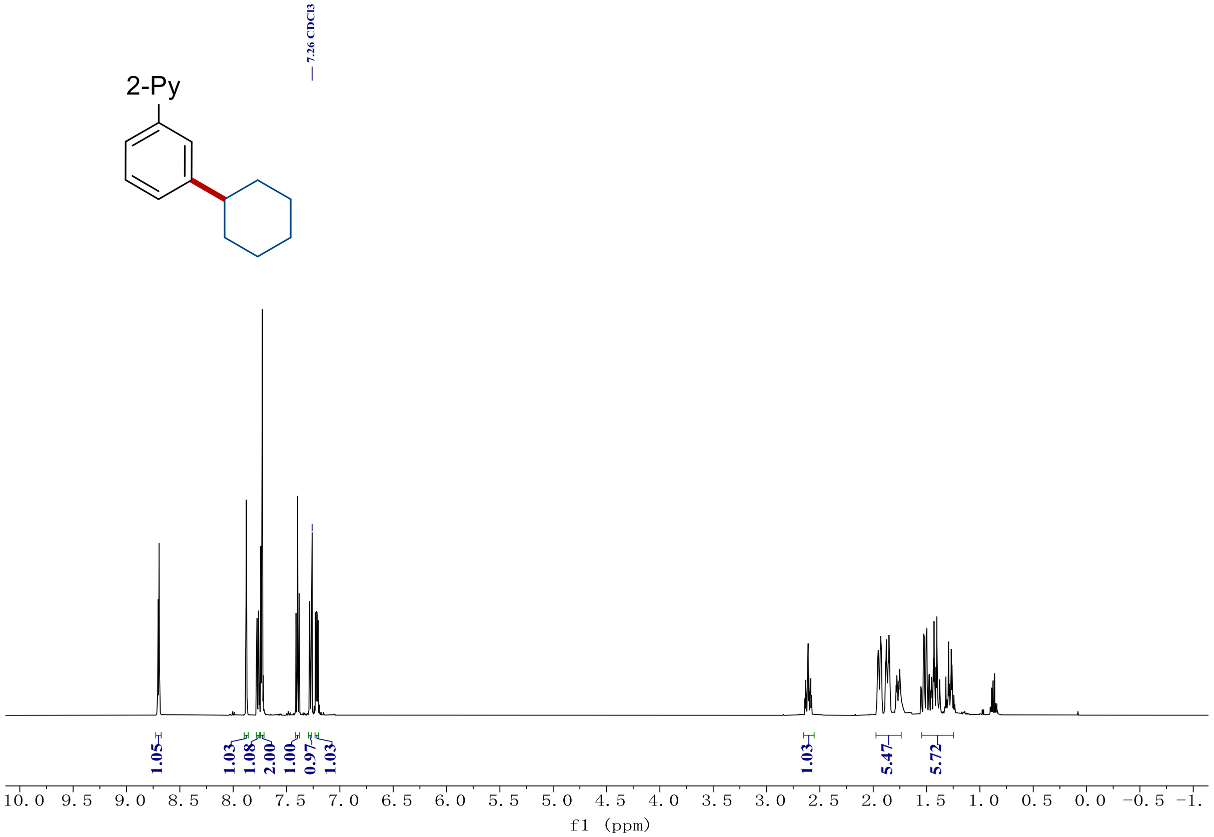

^{13}C NMR of **54a**, 126 MHz, $CDCl_3$, 25 °C.

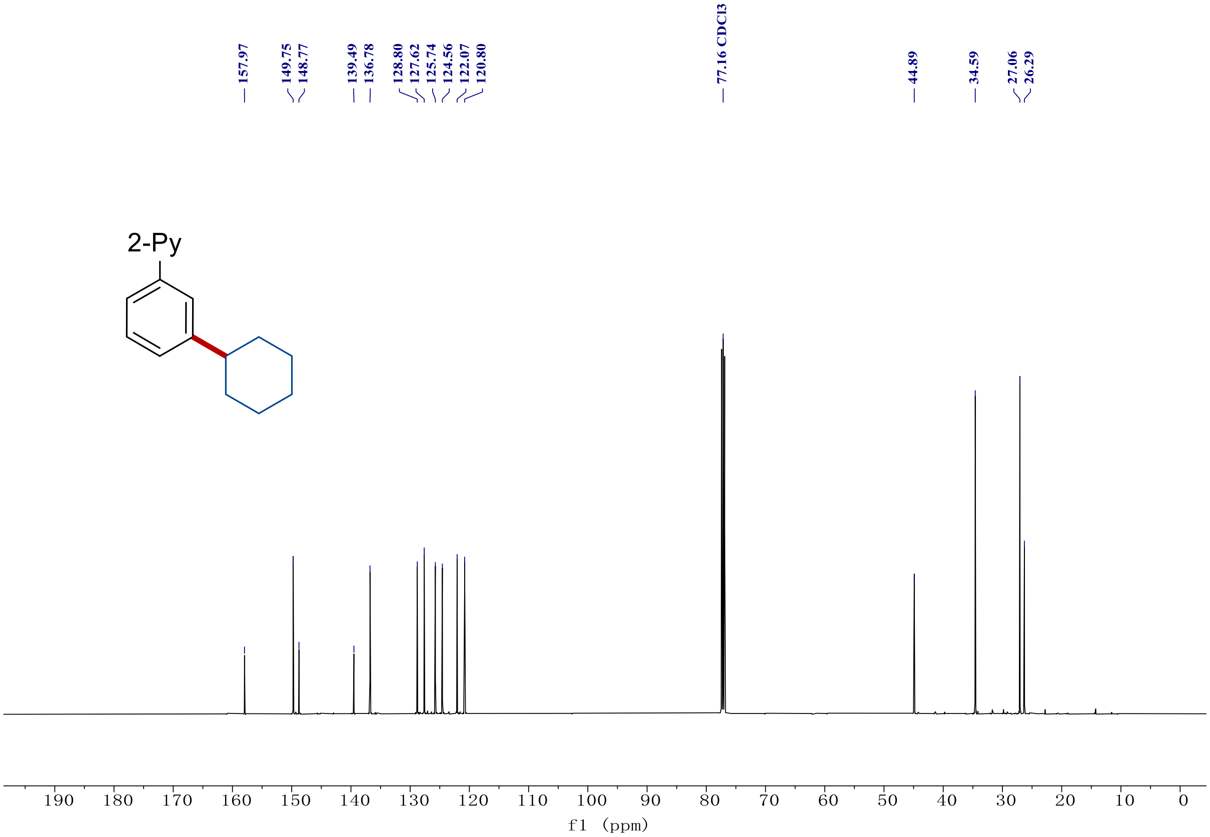

^{1}H NMR of **55a-*meta***, 400 MHz, $CDCl_3$, 25 °C.

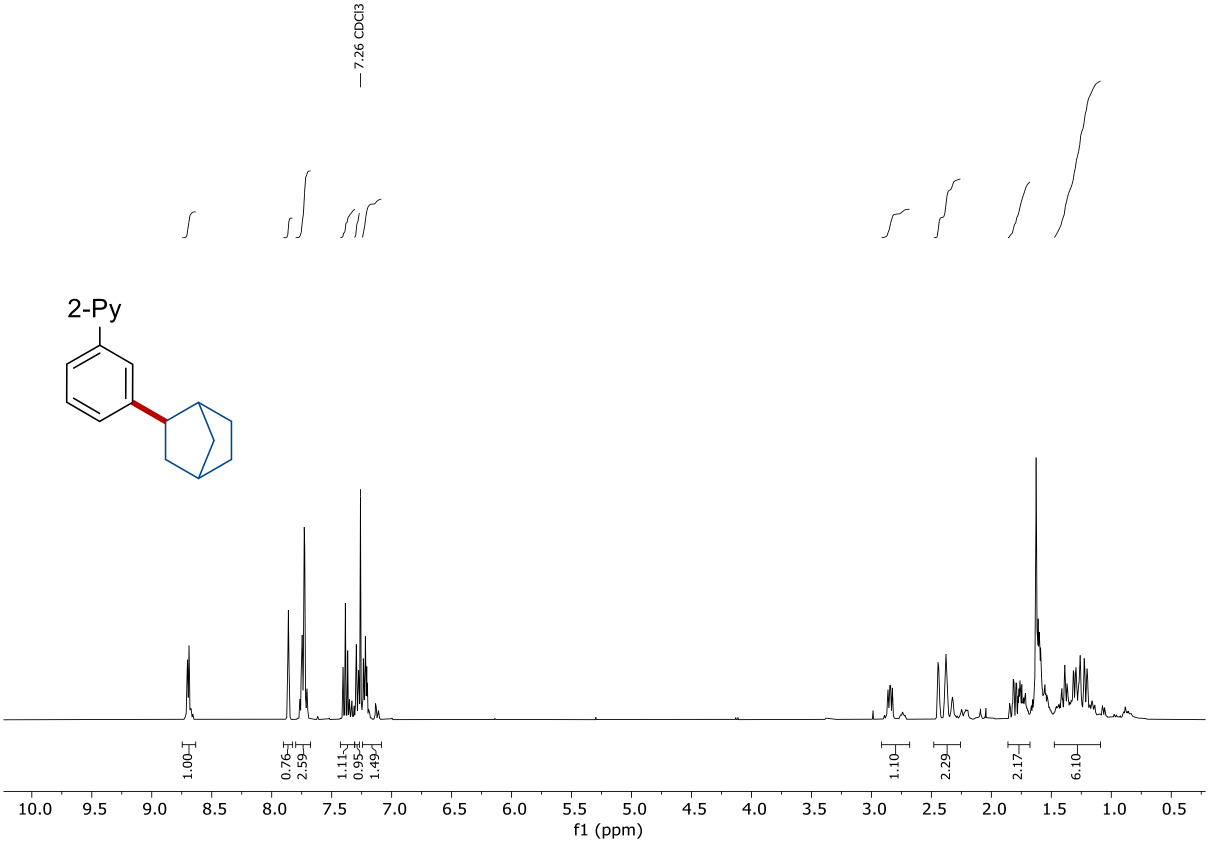

^{13}C NMR of **55a-*meta***, 101 MHz, $CDCl_3$, 25 °C.

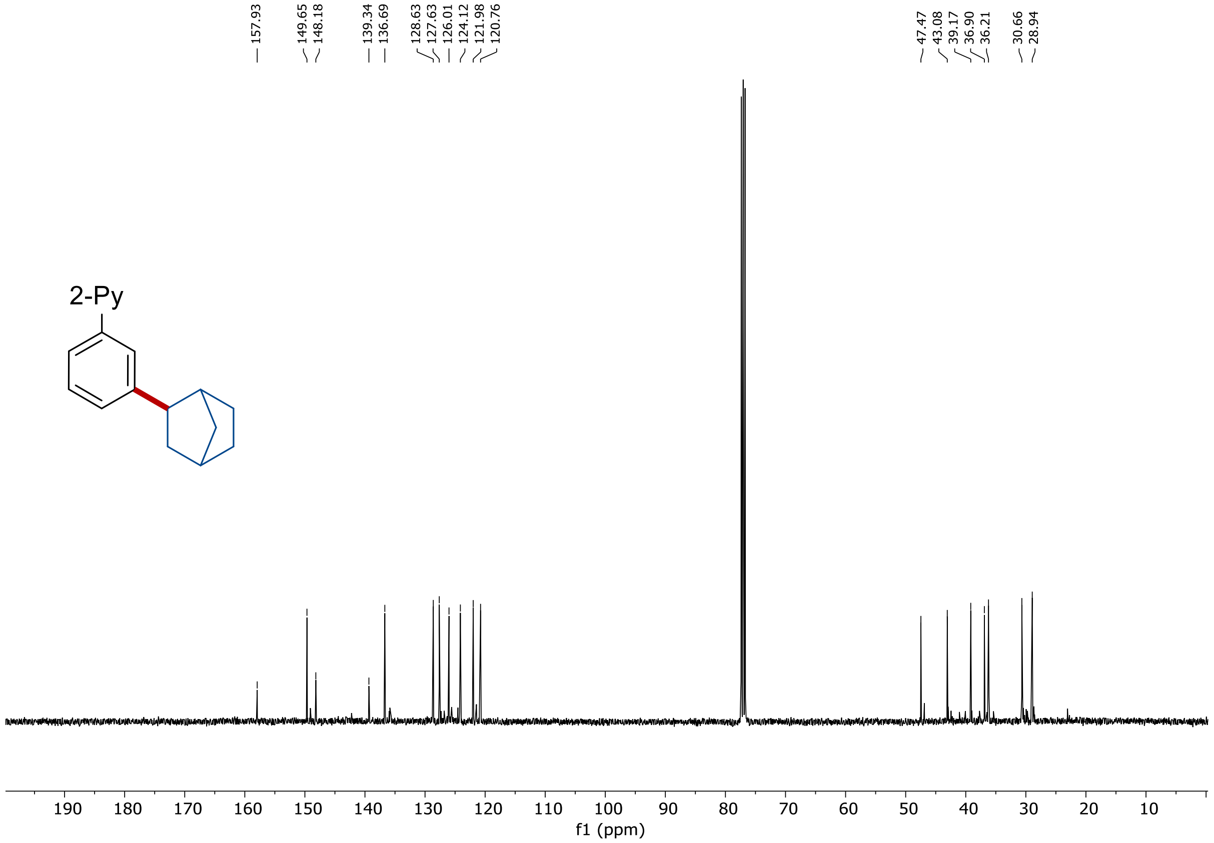

^{1}H NMR of **55a-*ortho***, 400 MHz, $CDCl_3$, 25 °C.

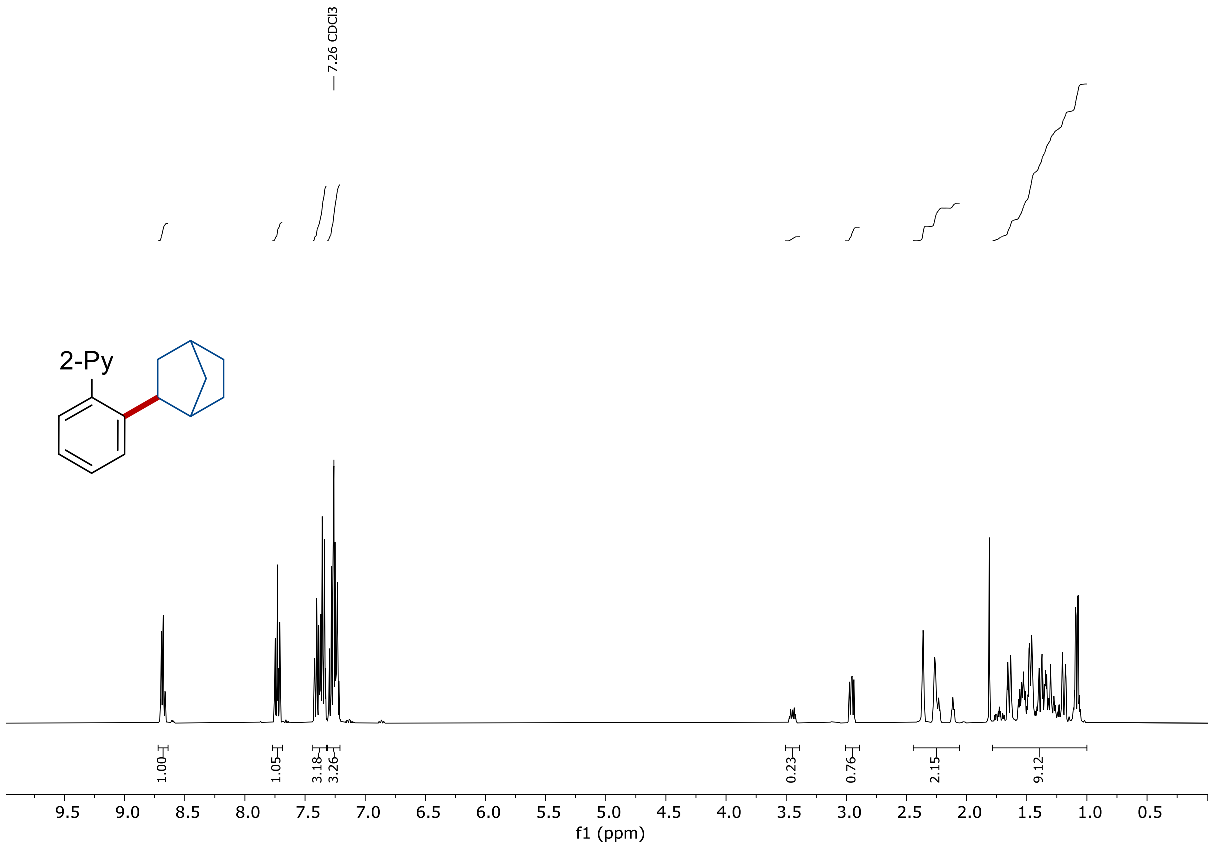

^{13}C NMR of **55a-*ortho***, 101 MHz, $CDCl_3$, 25 °C.

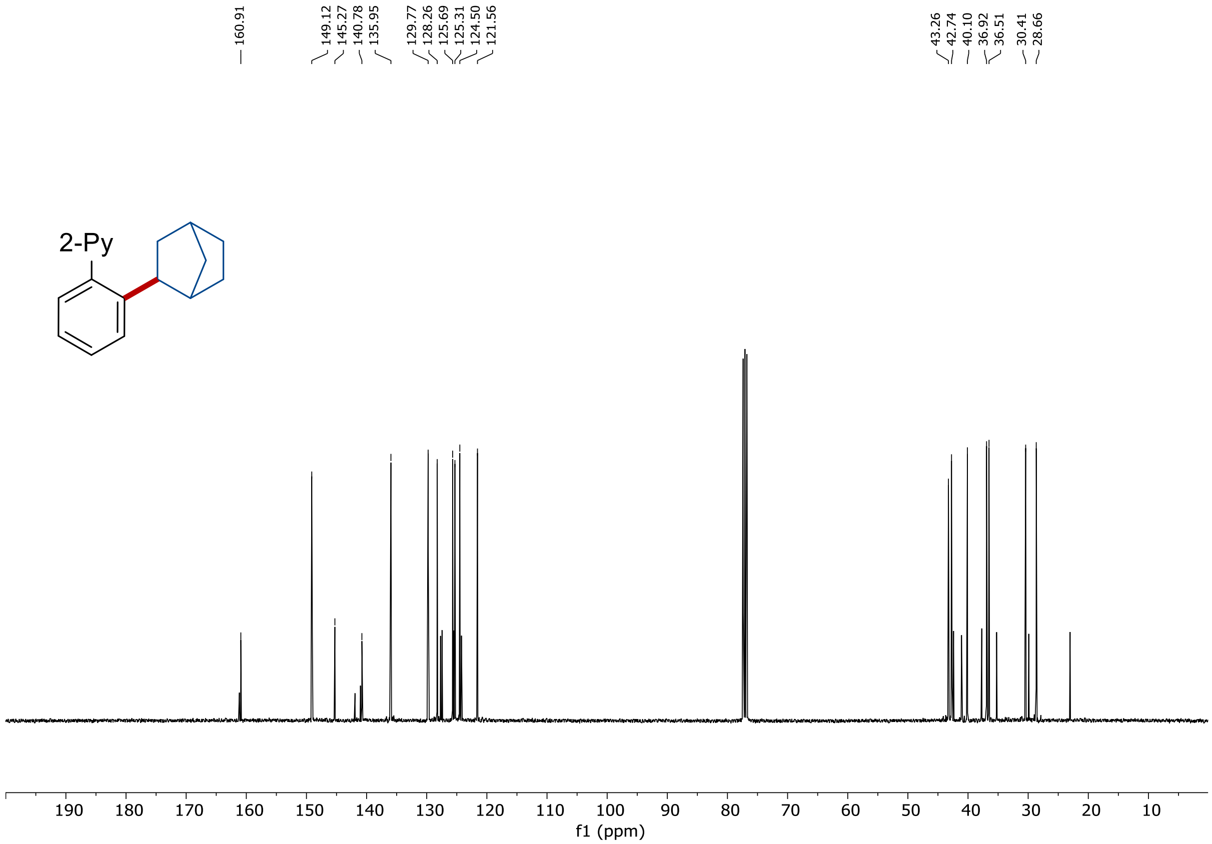

^{1}H NMR of **55b**, 400 MHz, $CDCl_3$, 25 °C.

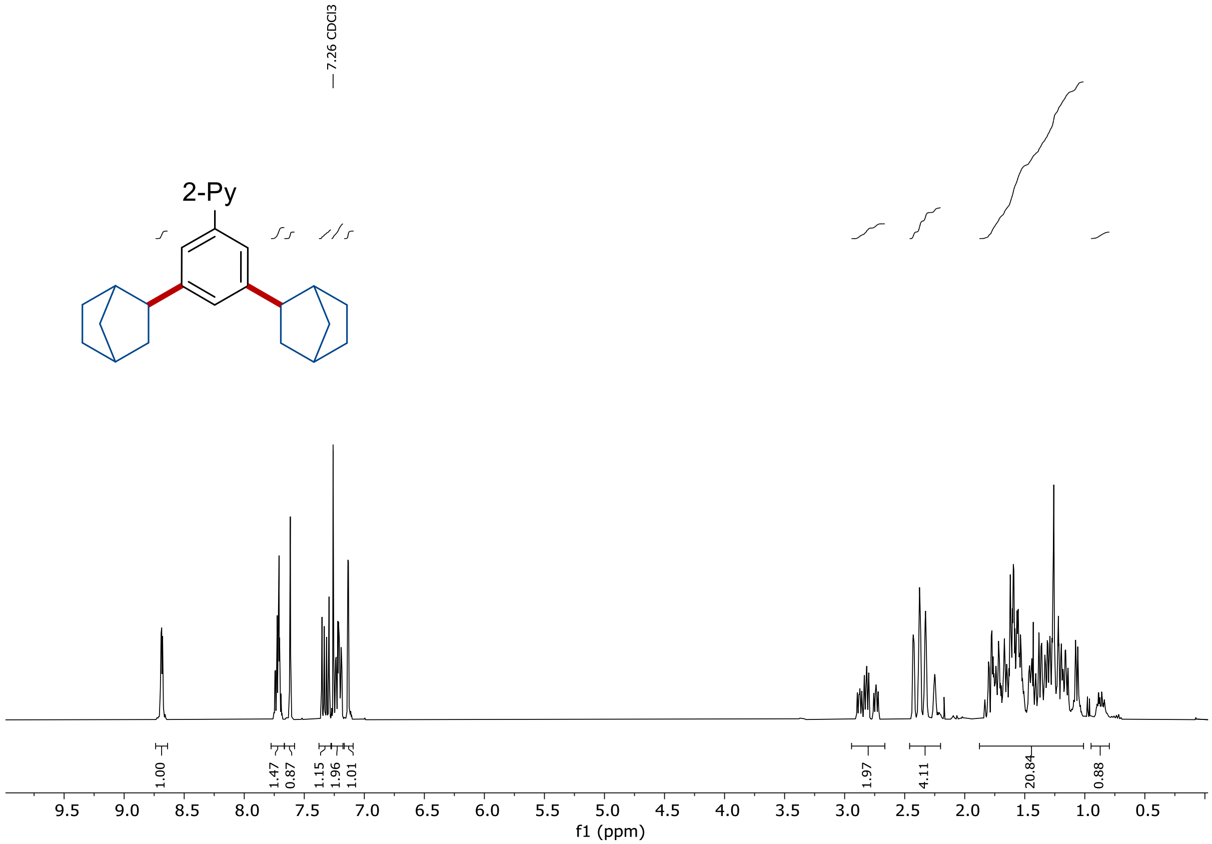

^{13}C NMR of **55b**, 101 MHz, $CDCl_3$, 25 °C.

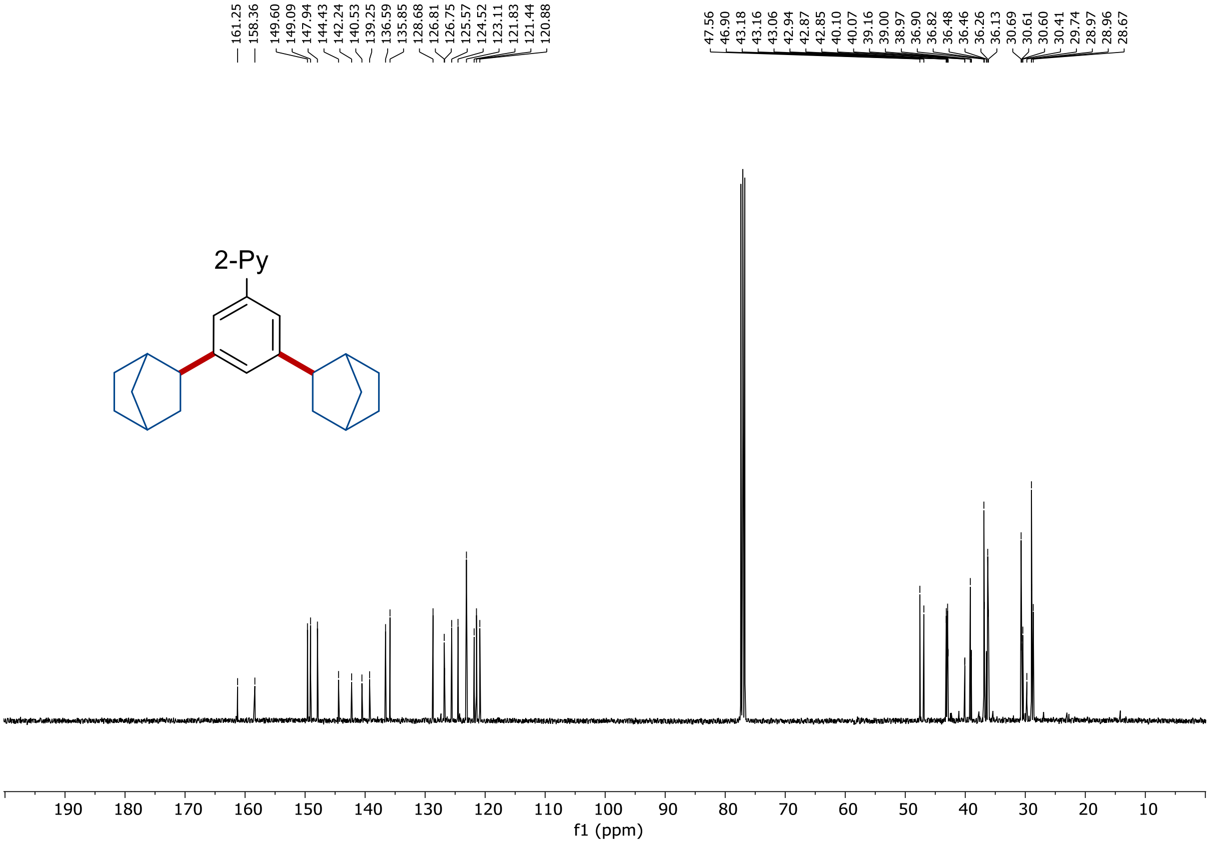

^{1}H NMR of **56a-*ortho***, 300 MHz, $CDCl_3$, 25 °C.

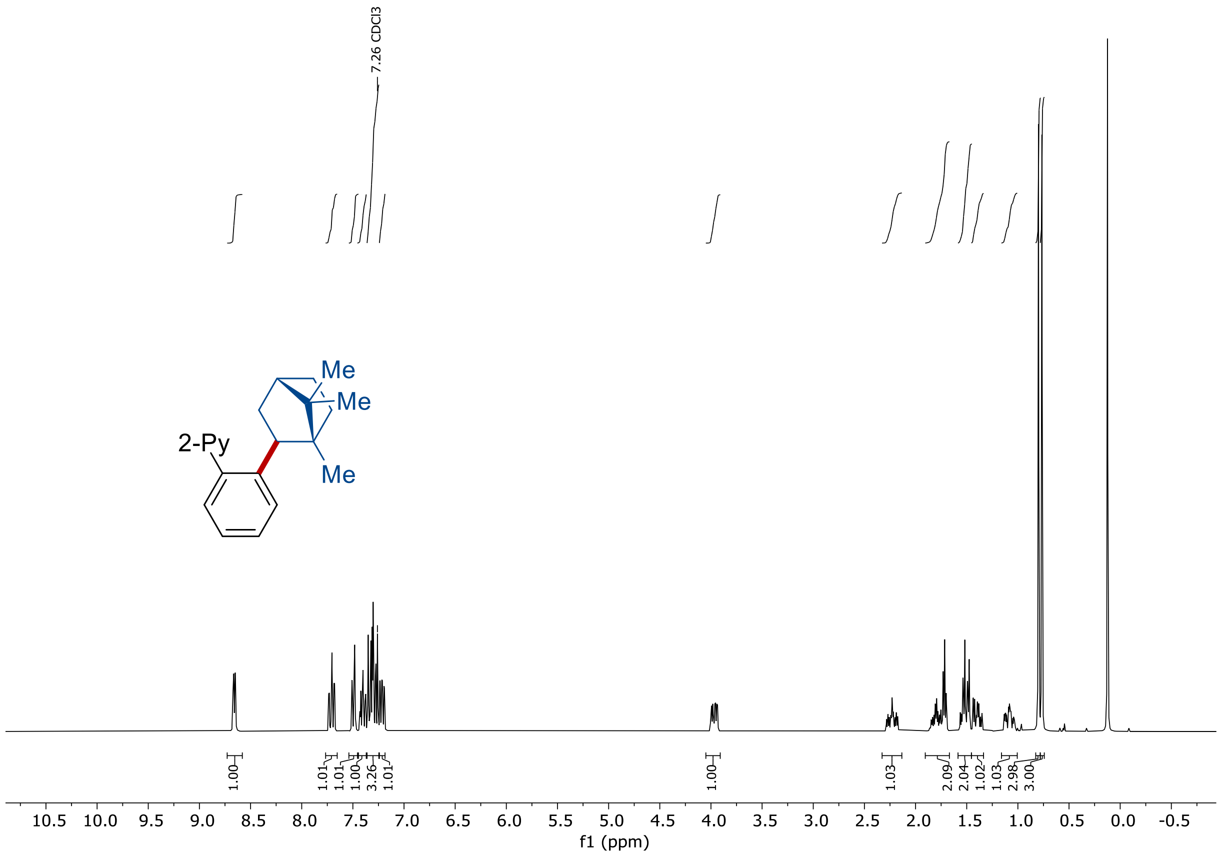

^{13}C NMR of **56-*ortho***, 75 MHz, $CDCl_3$, 25 °C.

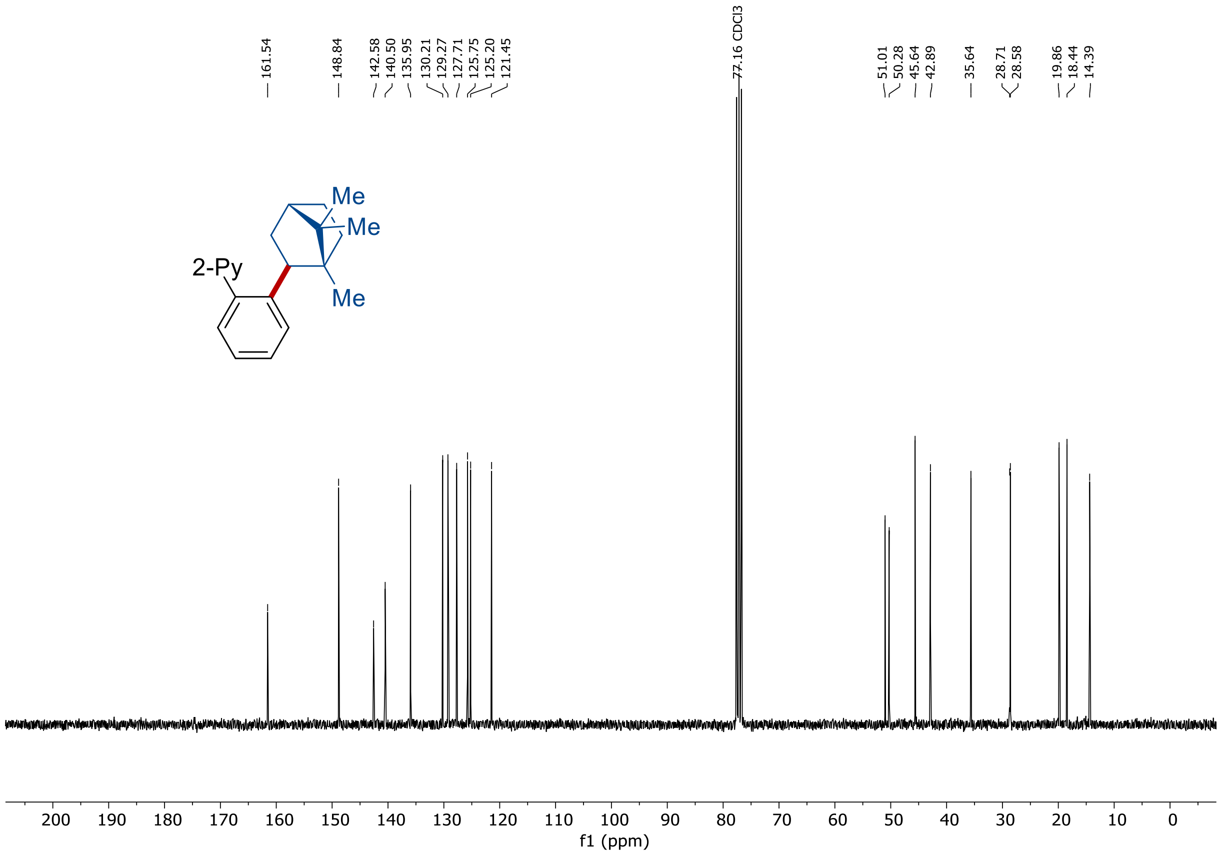

^{1}H NMR of **56a-*meta***, 300 MHz, $CDCl_3$, 25 °C.

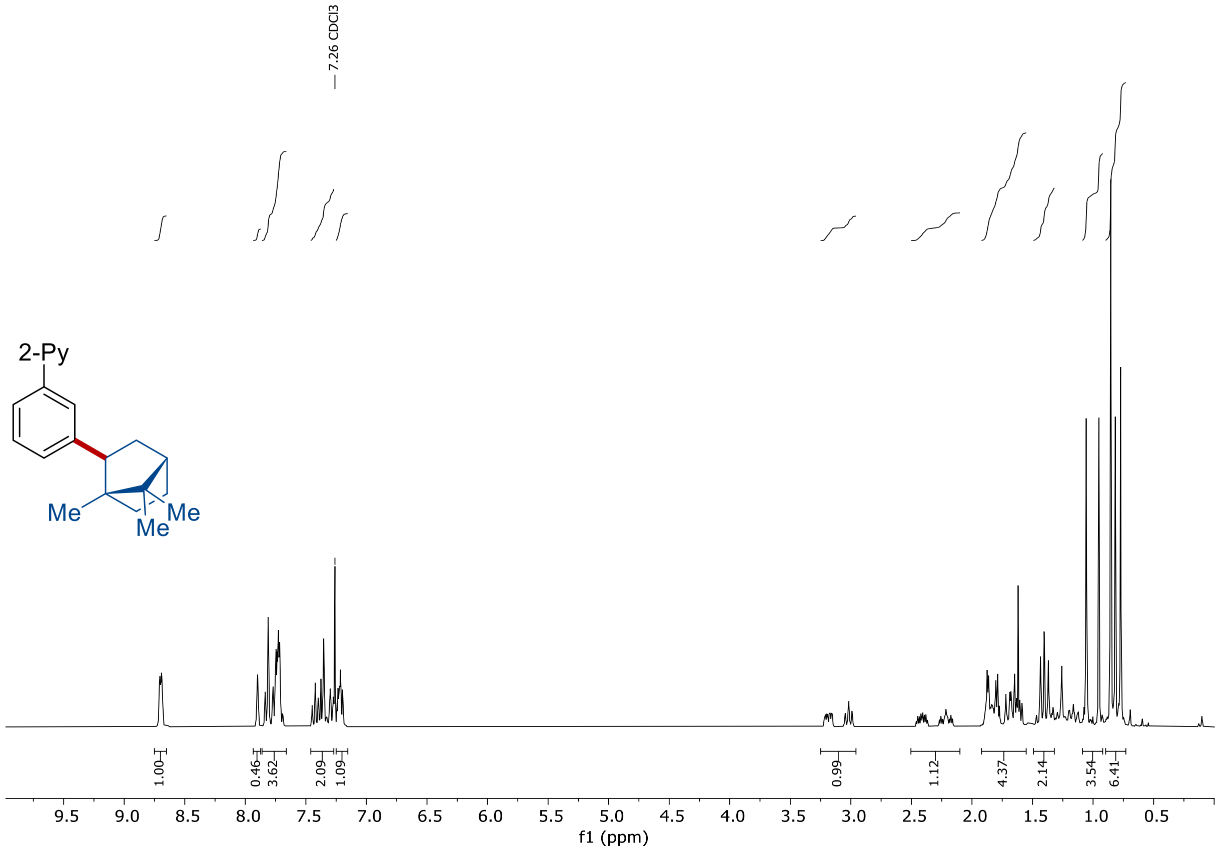

^{13}C NMR of **56a-*meta***, 75 MHz, $CDCl_3$, 25 °C.

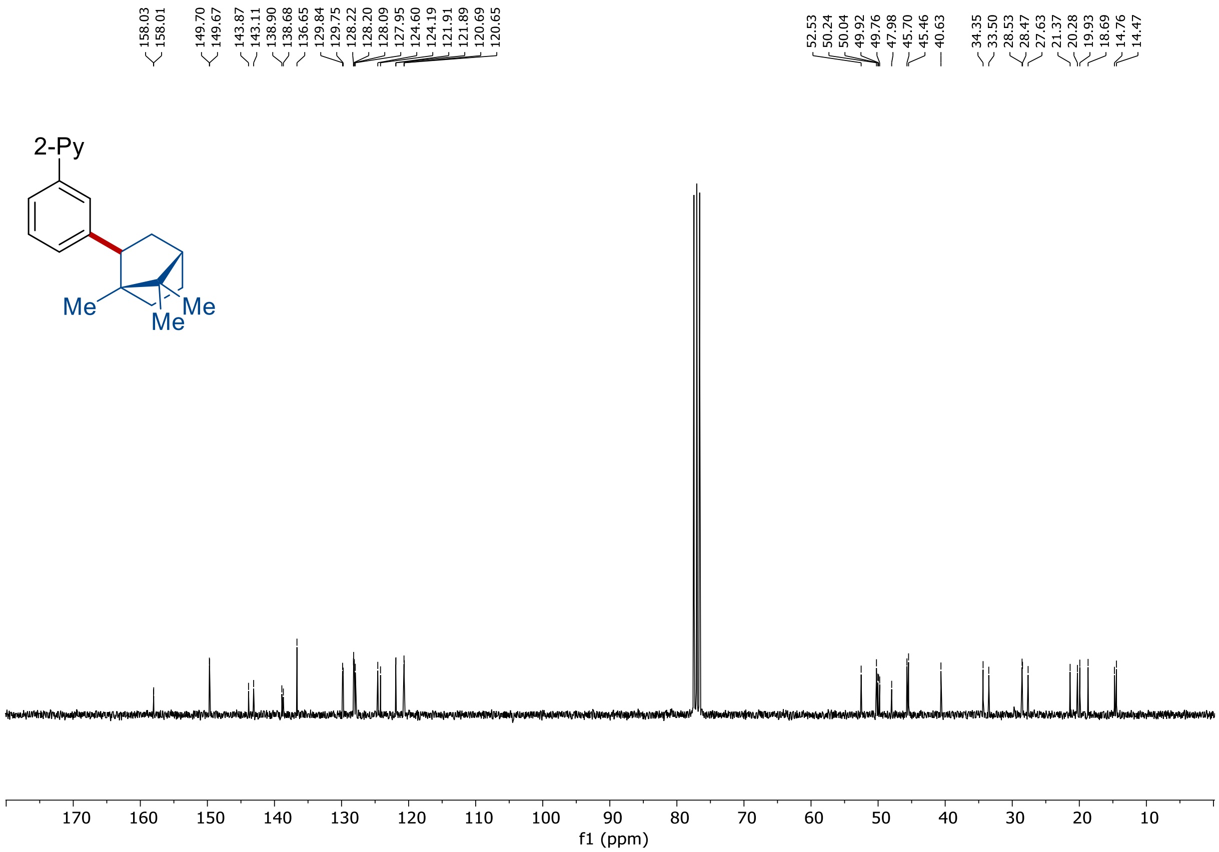

^{1}H NMR of **56b**, 300 MHz, $CDCl_3$, 25 °C.

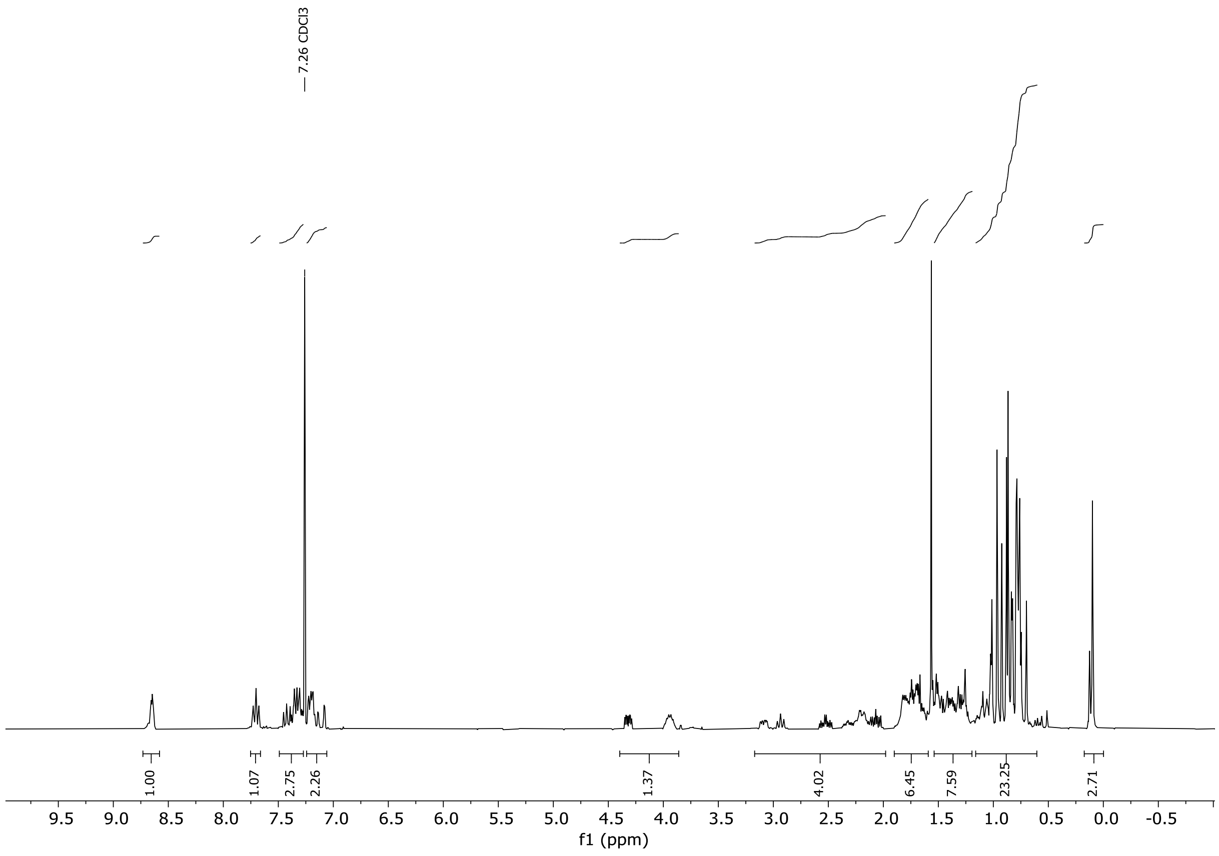

^{1}H NMR of **57a-*meta***, 600 MHz, $CDCl_3$, 25 °C.

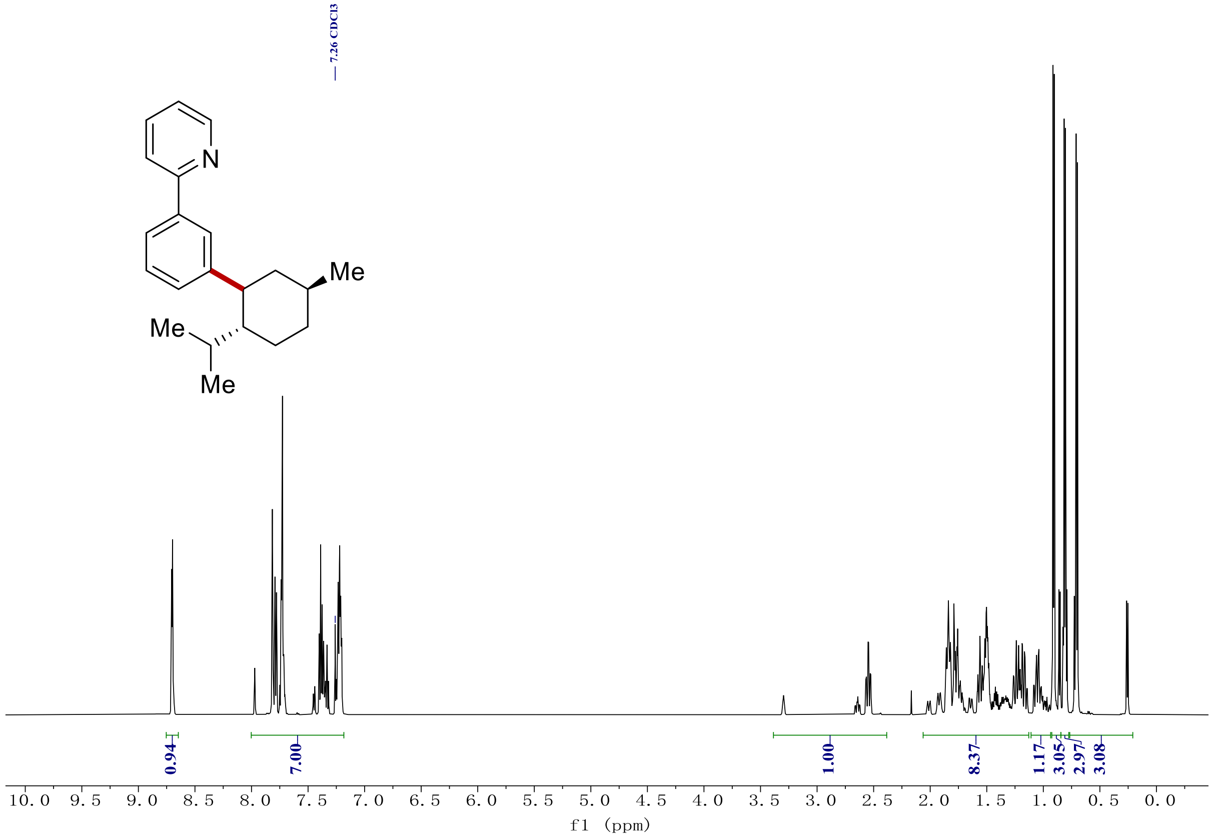

^{13}C NMR of **57a-*meta***, 126 MHz, $CDCl_3$, 25 °C.

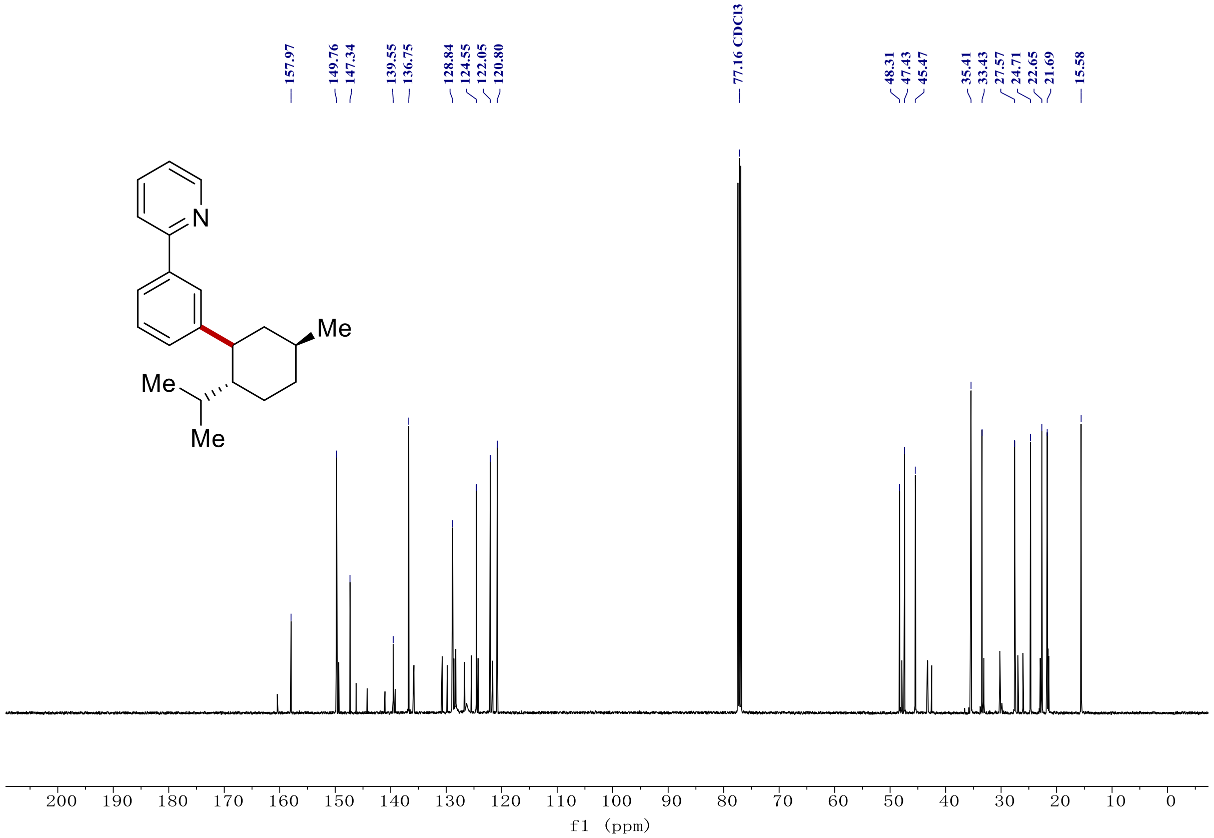

^{1}H NMR of **57b**, 300 MHz, $CDCl_3$, 25 °C.

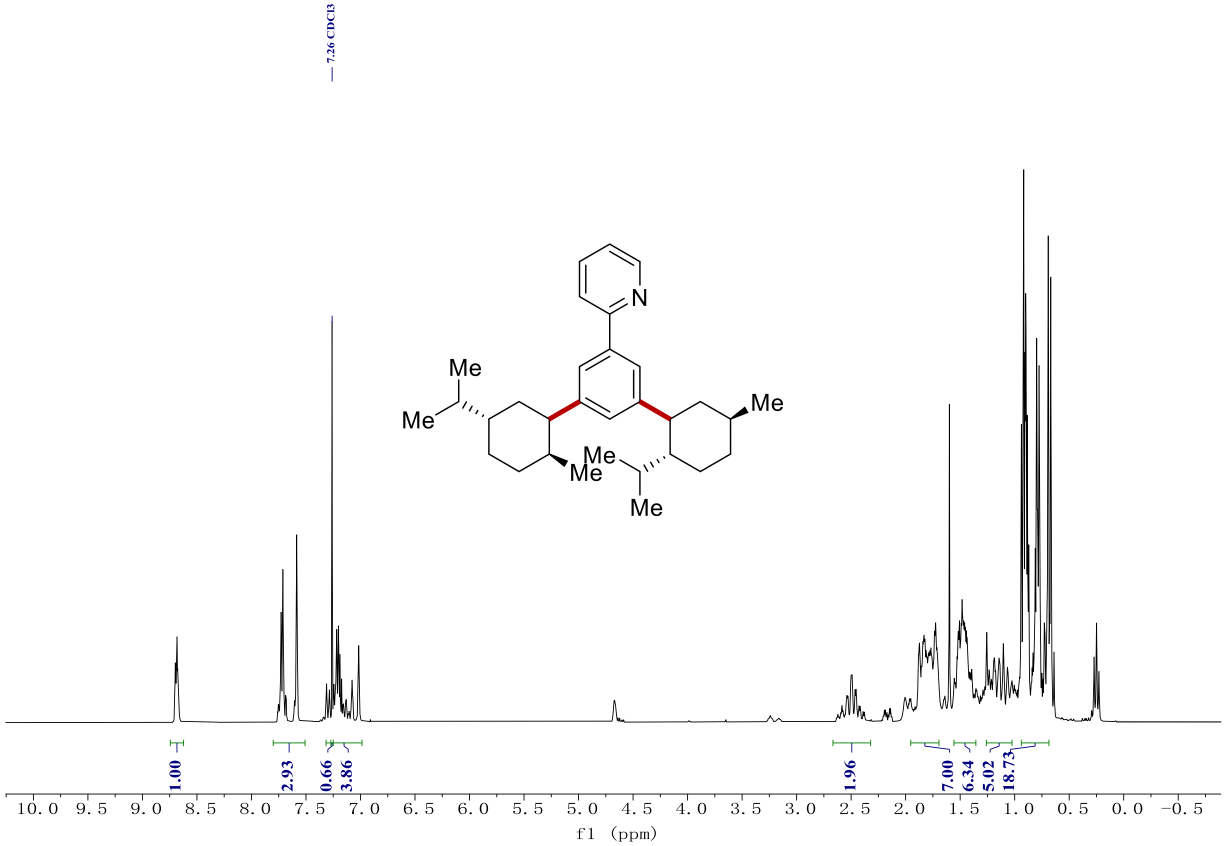

^{1}H NMR of **58a-*meta***, 300 MHz, $CDCl_3$, 25 °C.

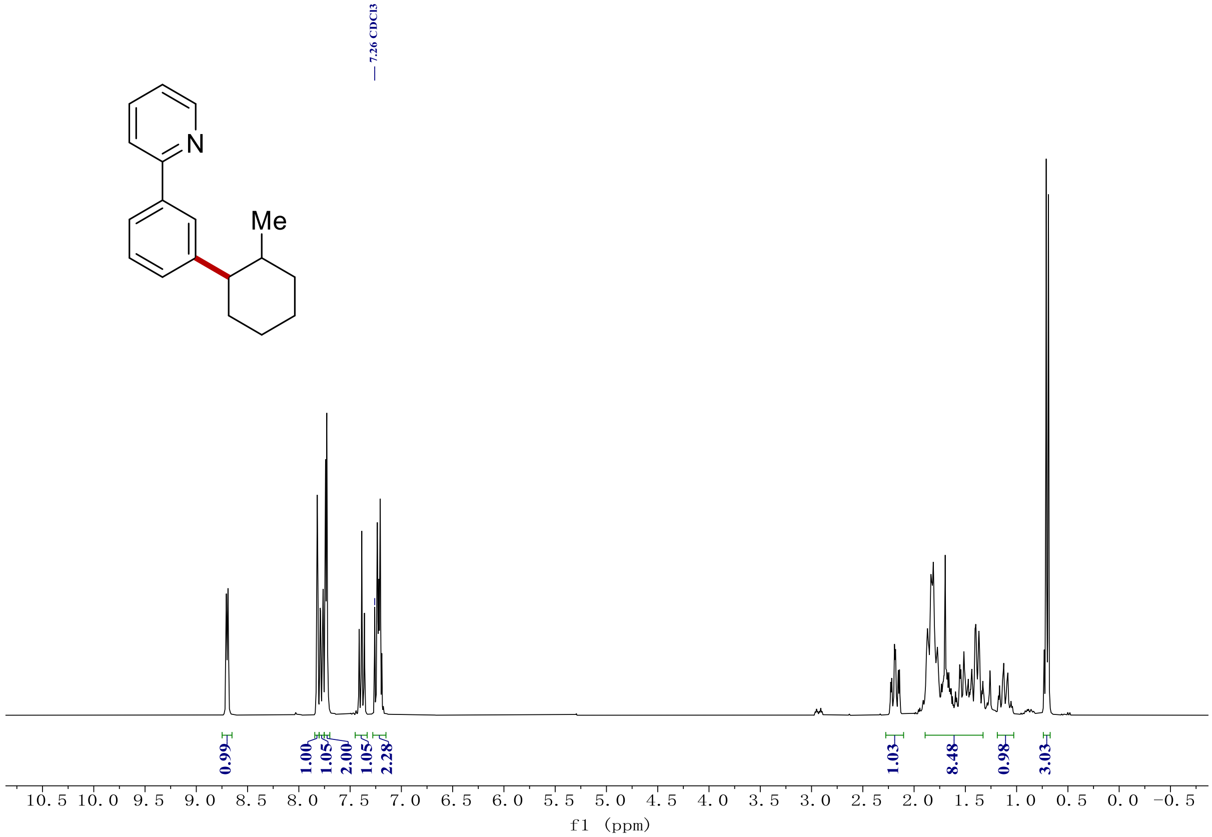

^{13}C NMR of **58a-*meta***, 75 MHz, $CDCl_3$, 25 °C.

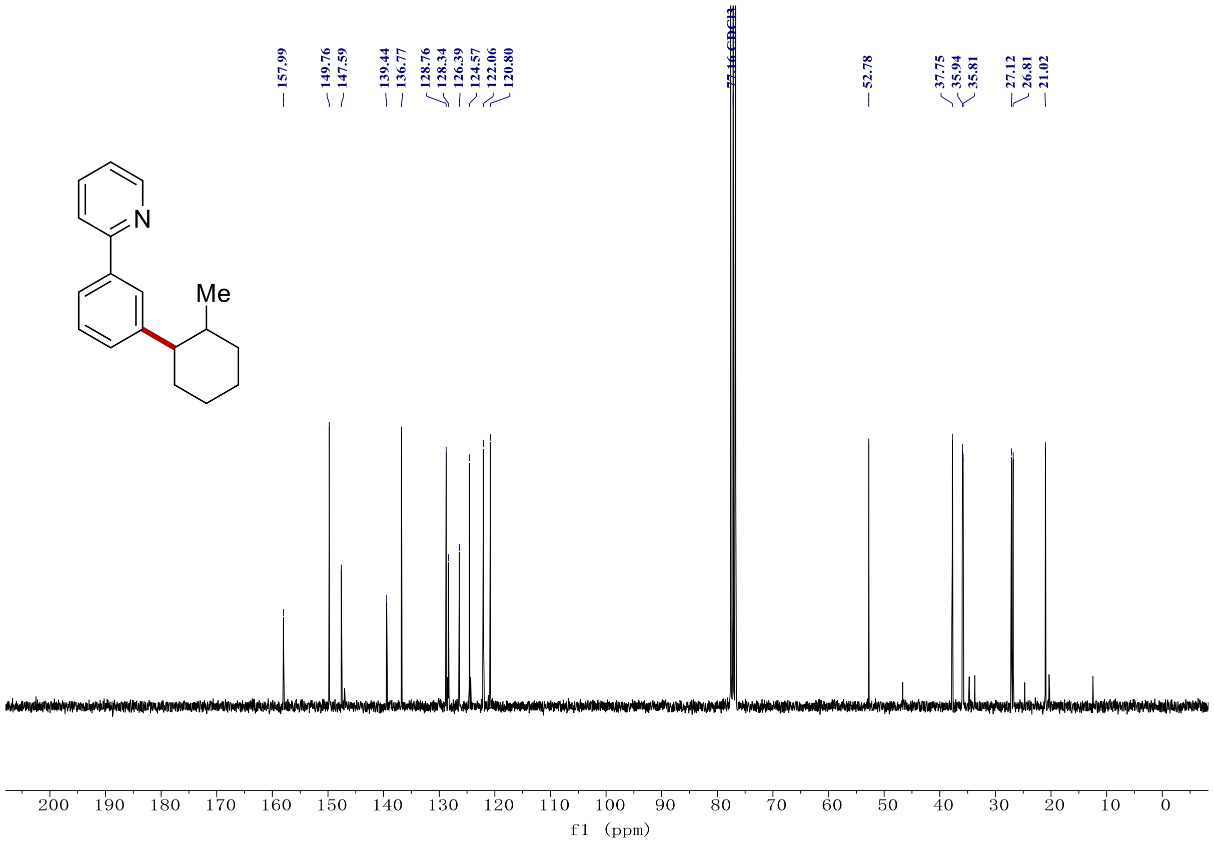

^{1}H NMR of **59a-*meta***, 300 MHz, $CDCl_3$, 25 °C.

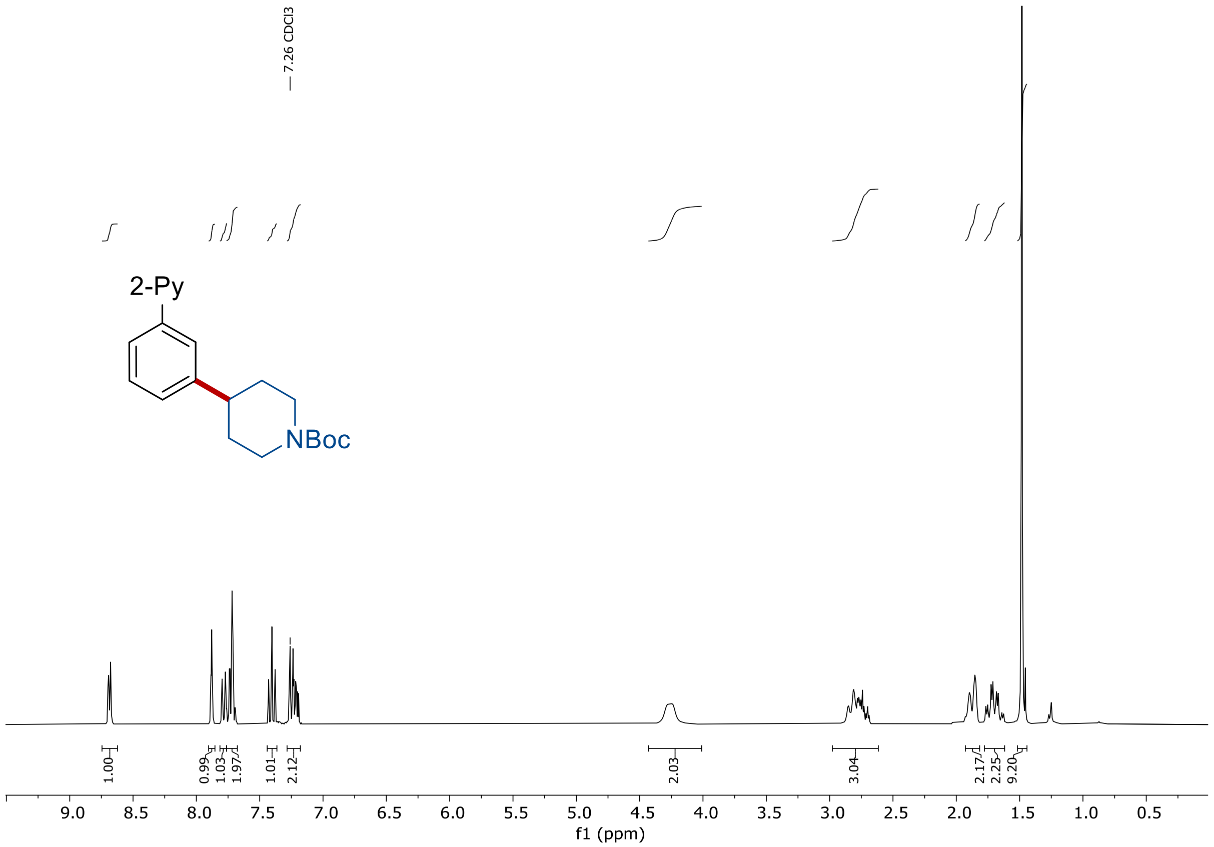

^{13}C NMR of **59a-*meta***, 75 MHz, $CDCl_3$, 25 °C.

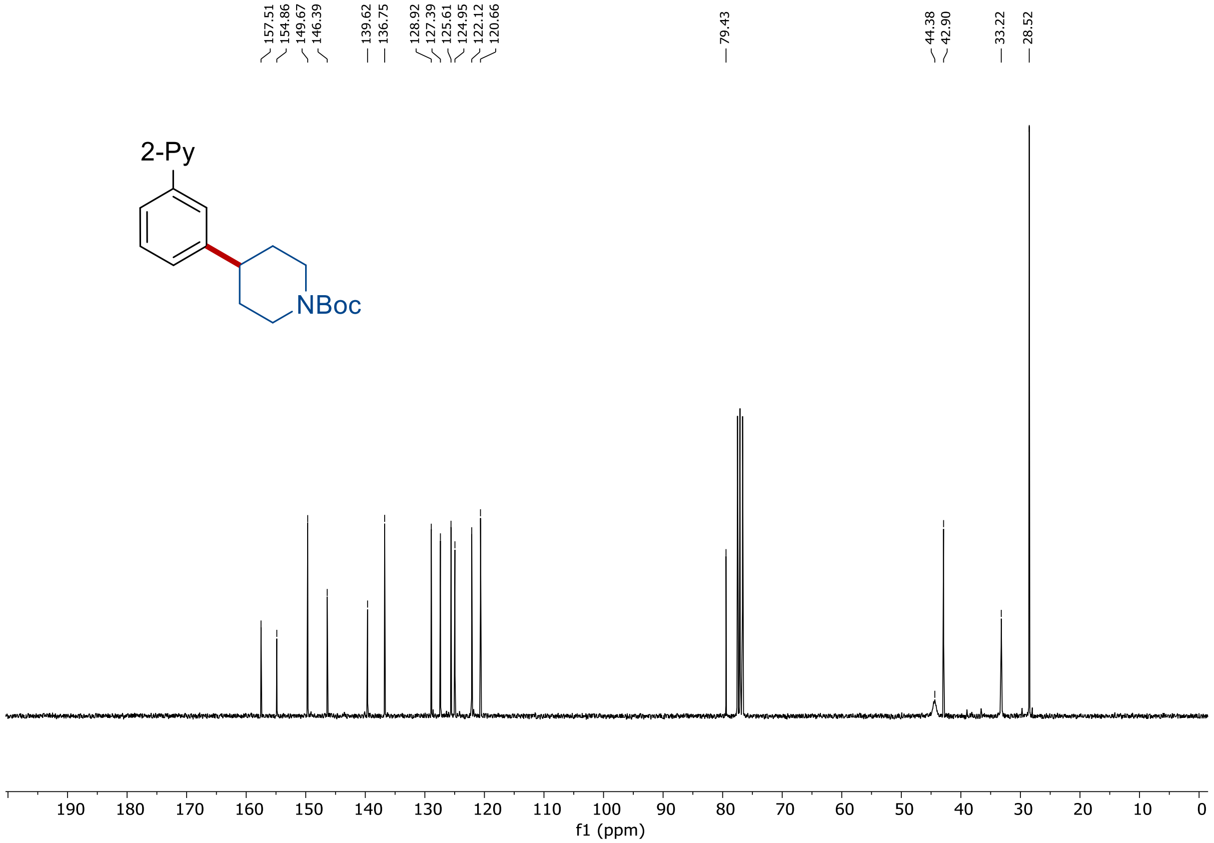

^{1}H NMR of **59a** (a mixture of *o*- and *m*-product), 300 MHz, $CDCl_3$, 25 °C.

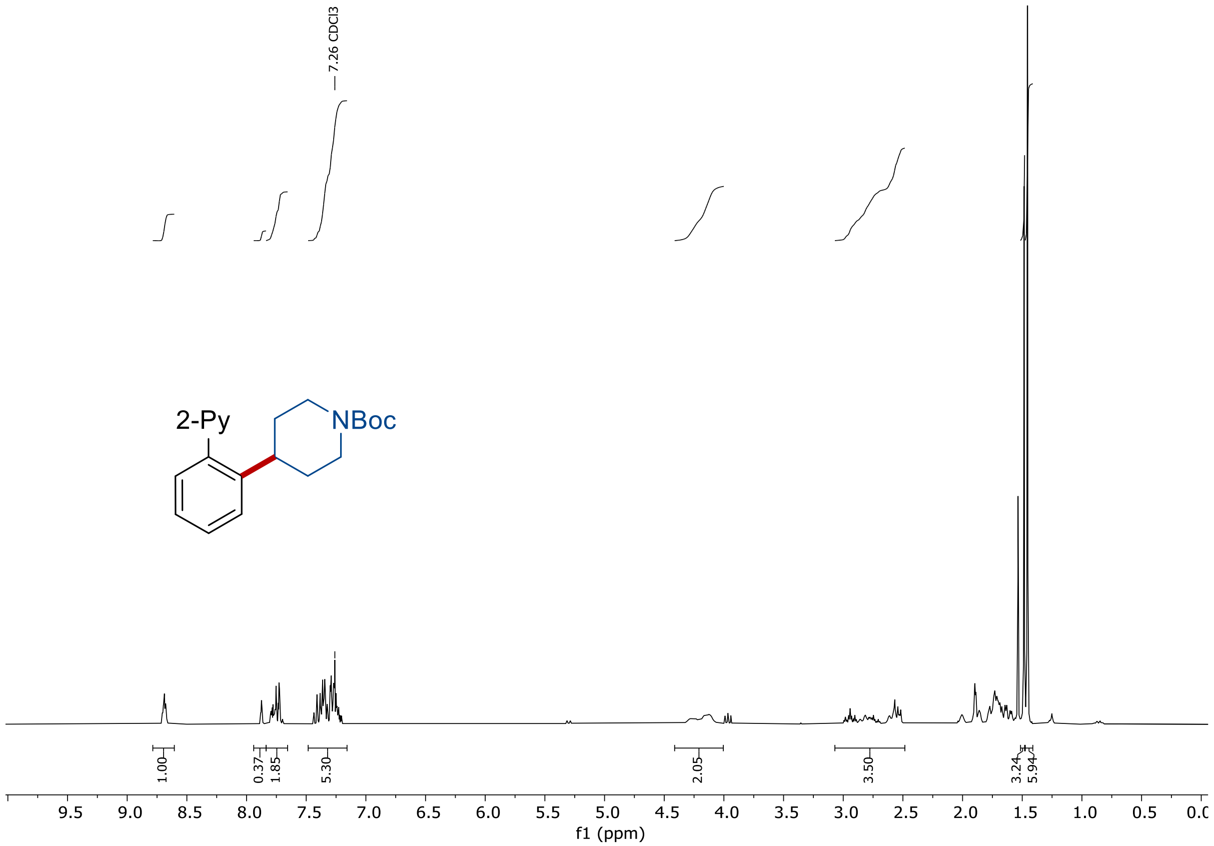

^{1}H NMR of **59b**, 300 MHz, $CDCl_3$, 25 °C.

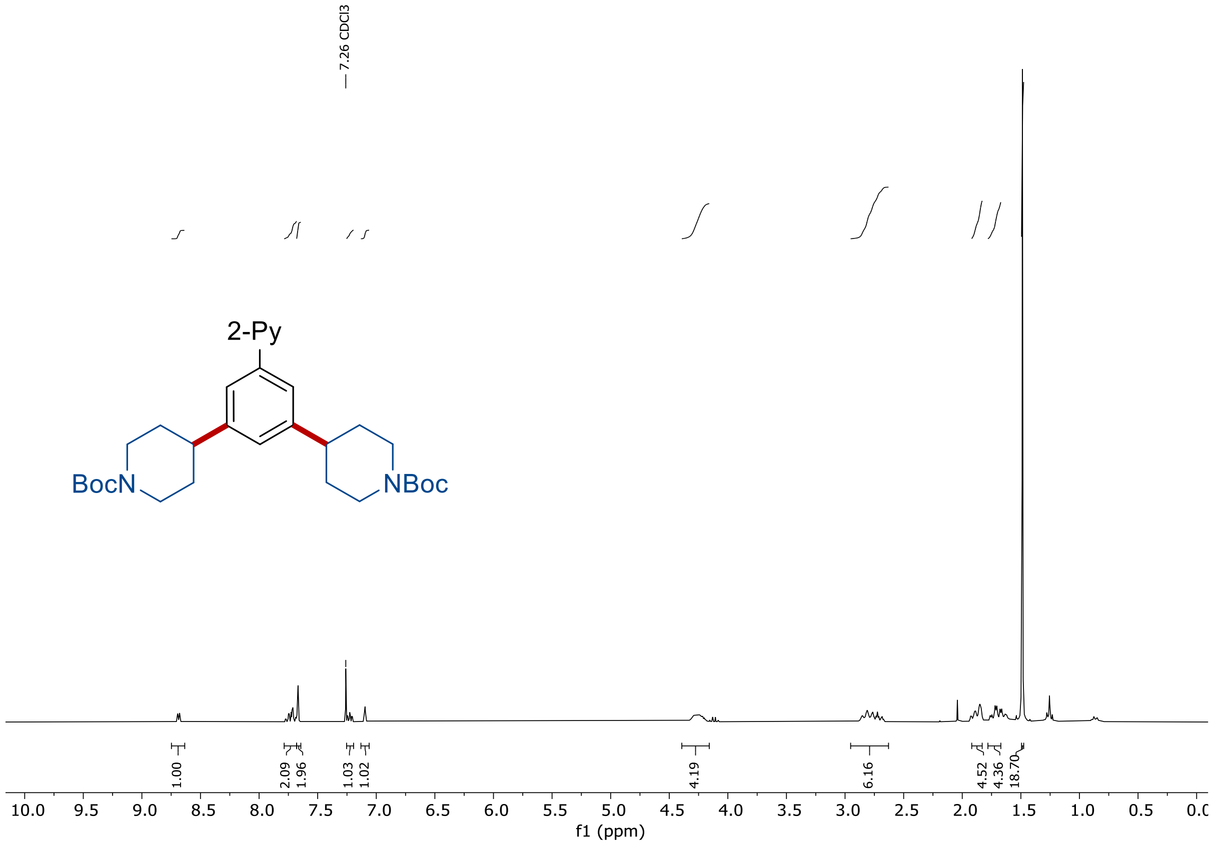

^{13}C NMR of **59b**, 75 MHz, $CDCl_3$, 25 °C.

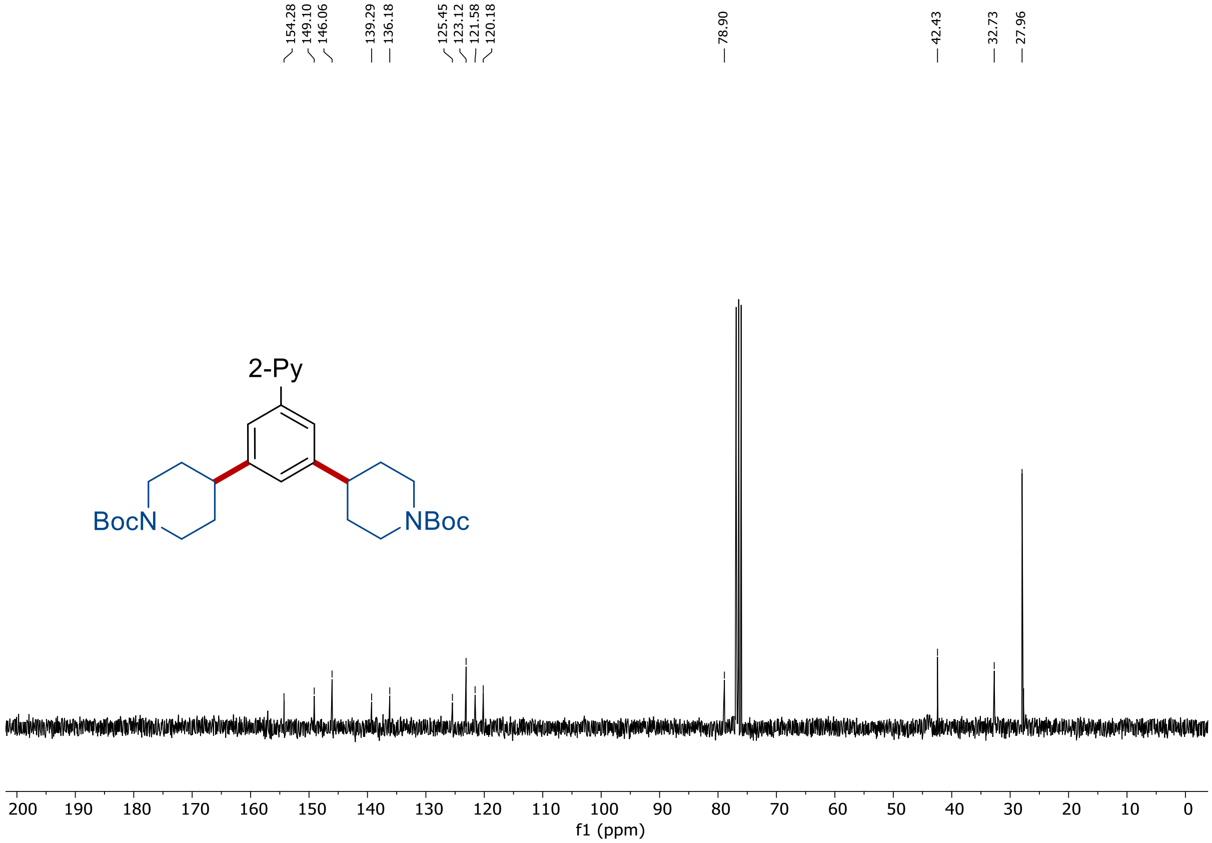

^{1}H NMR of **60a**, 500 MHz, $CDCl_3$, 25 °C.

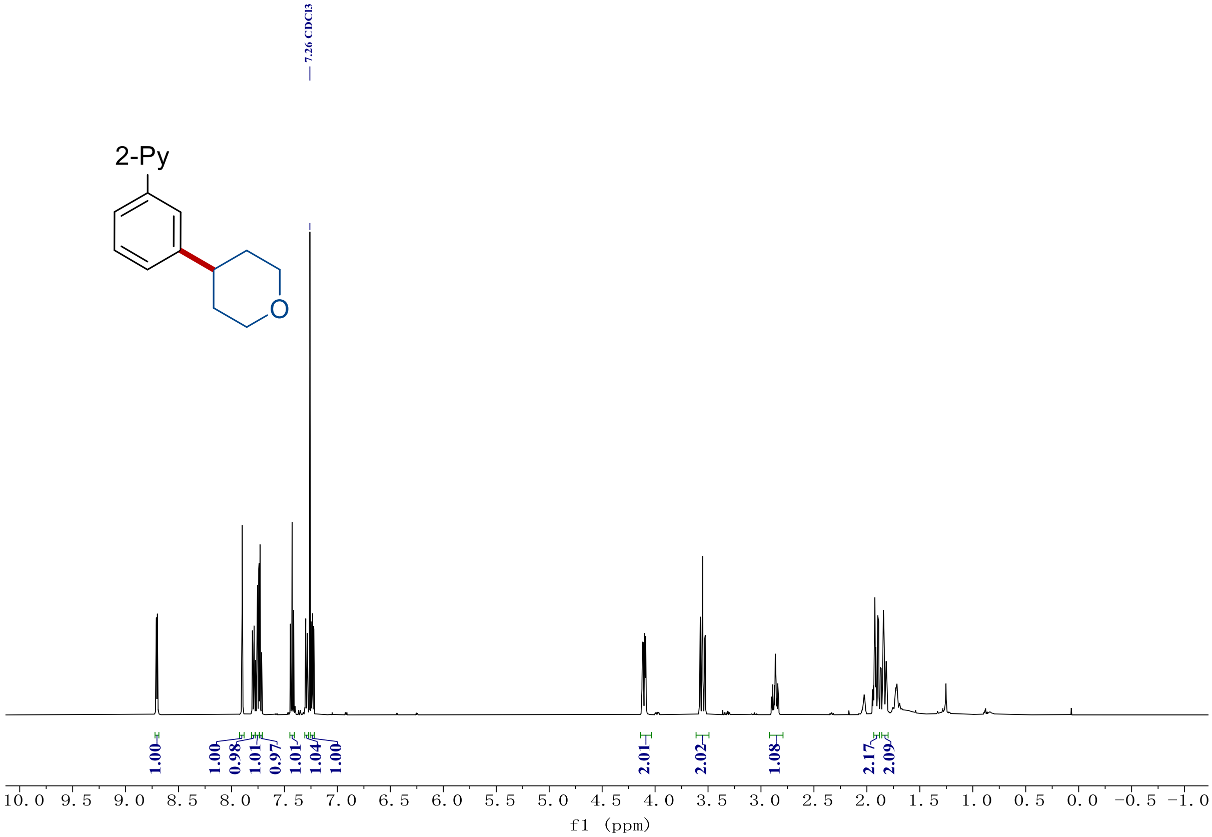

^{13}C NMR of **60a**, 126 MHz, $CDCl_3$, 25 °C.

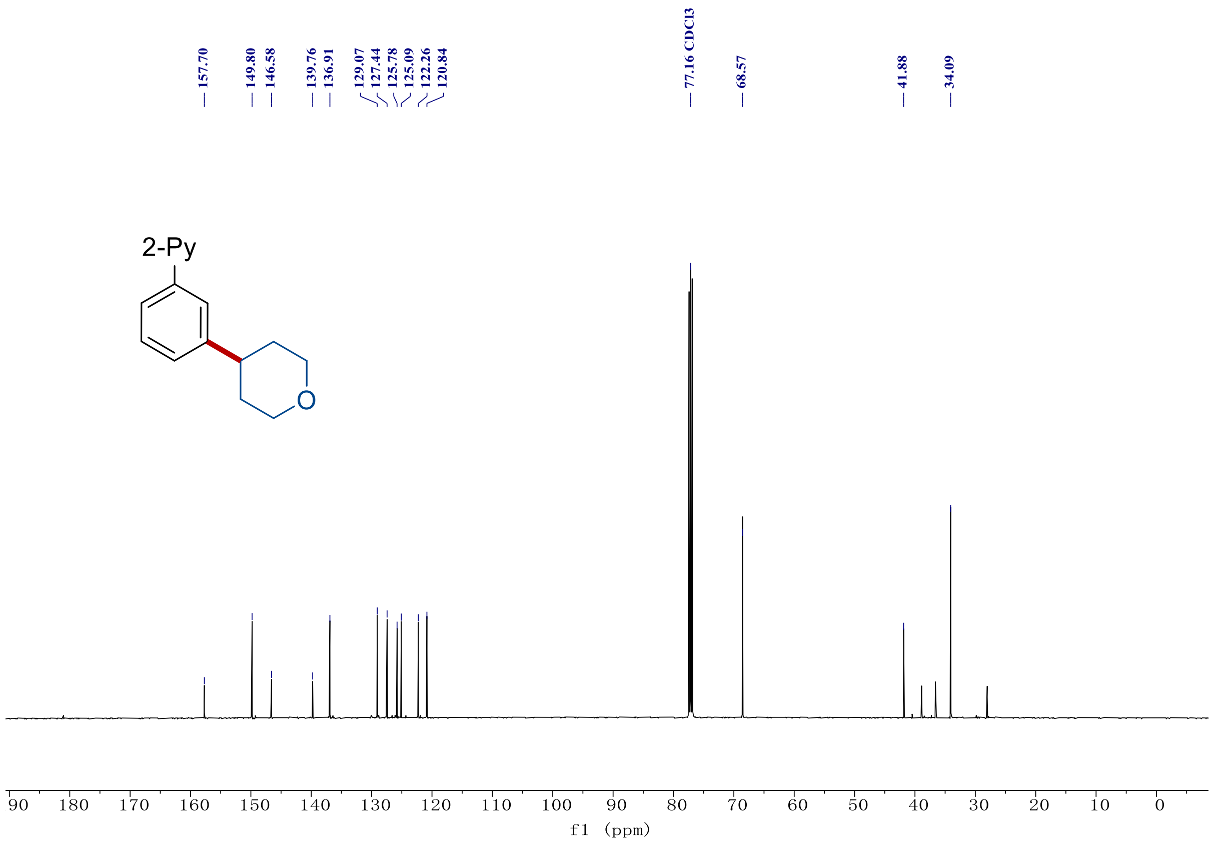

^{1}H NMR of **60b**, 500 MHz, $CDCl_3$, 25 °C.

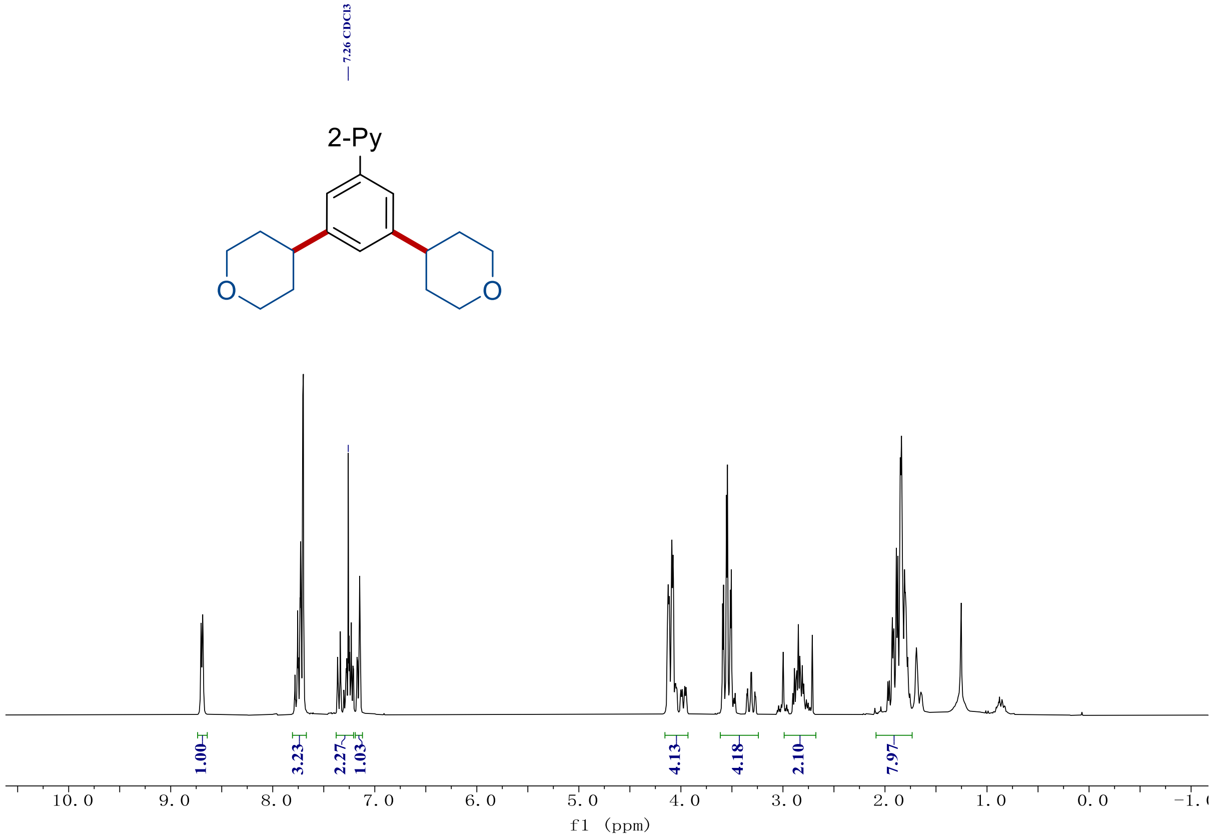

^{13}C NMR of **60b**, 126 MHz, $CDCl_3$, 25 °C.

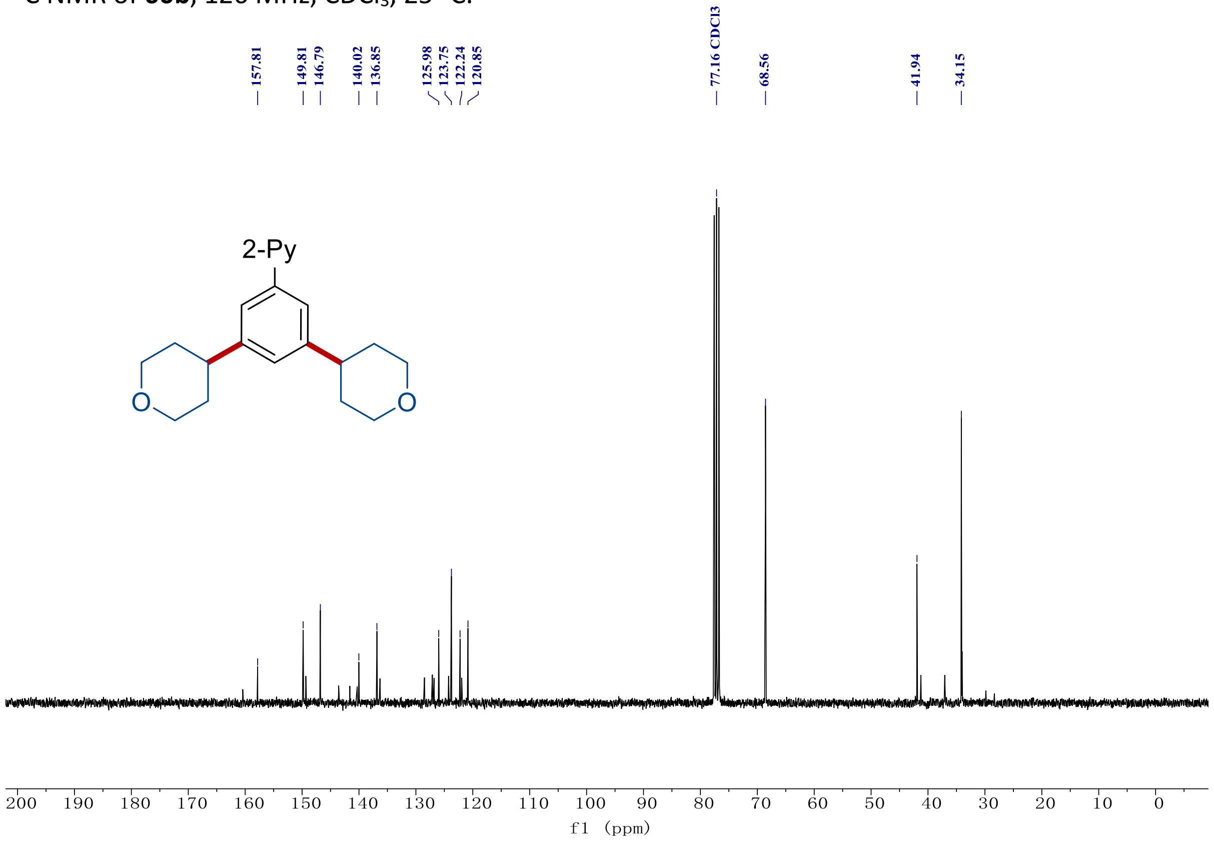

^{1}H NMR of **61a**, 500 MHz, $CDCl_3$, 25 °C.

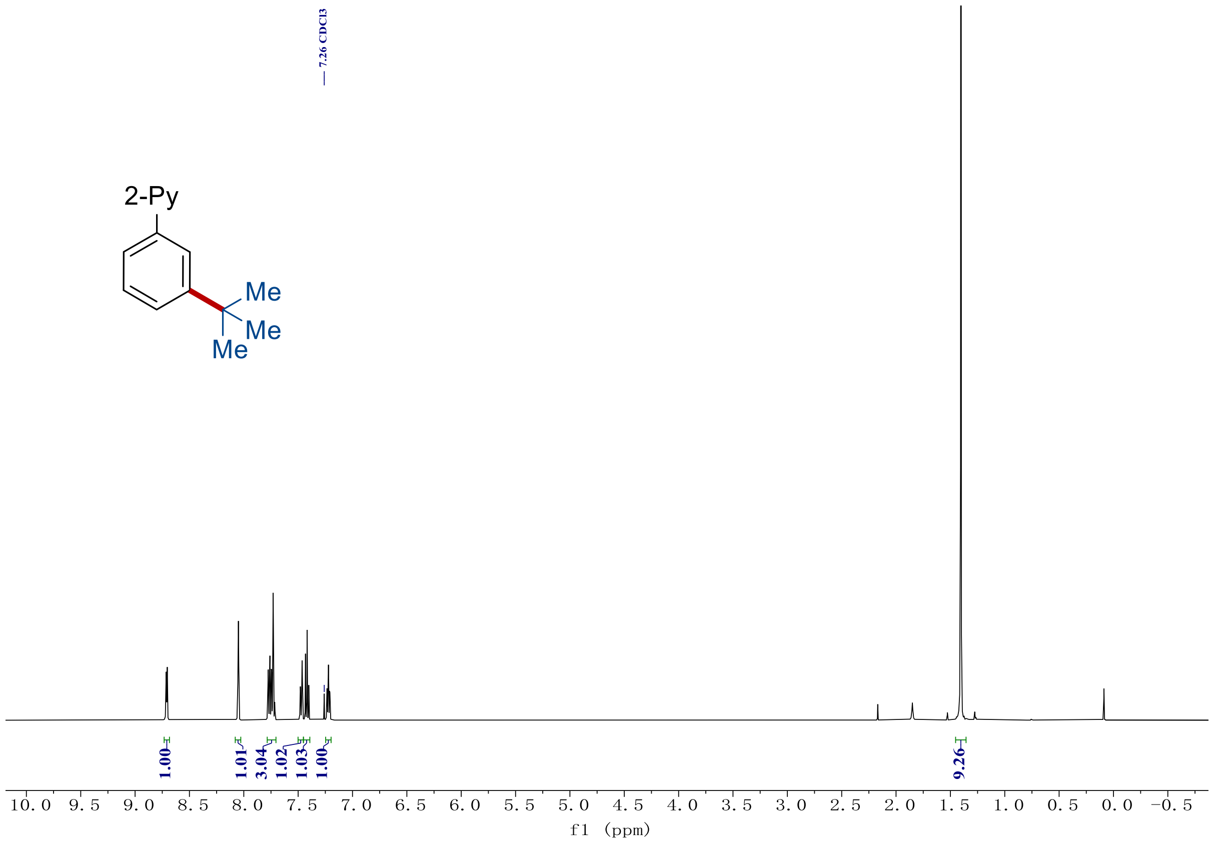

^{13}C NMR of **61a**, 126 MHz, $CDCl_3$, 25 °C.

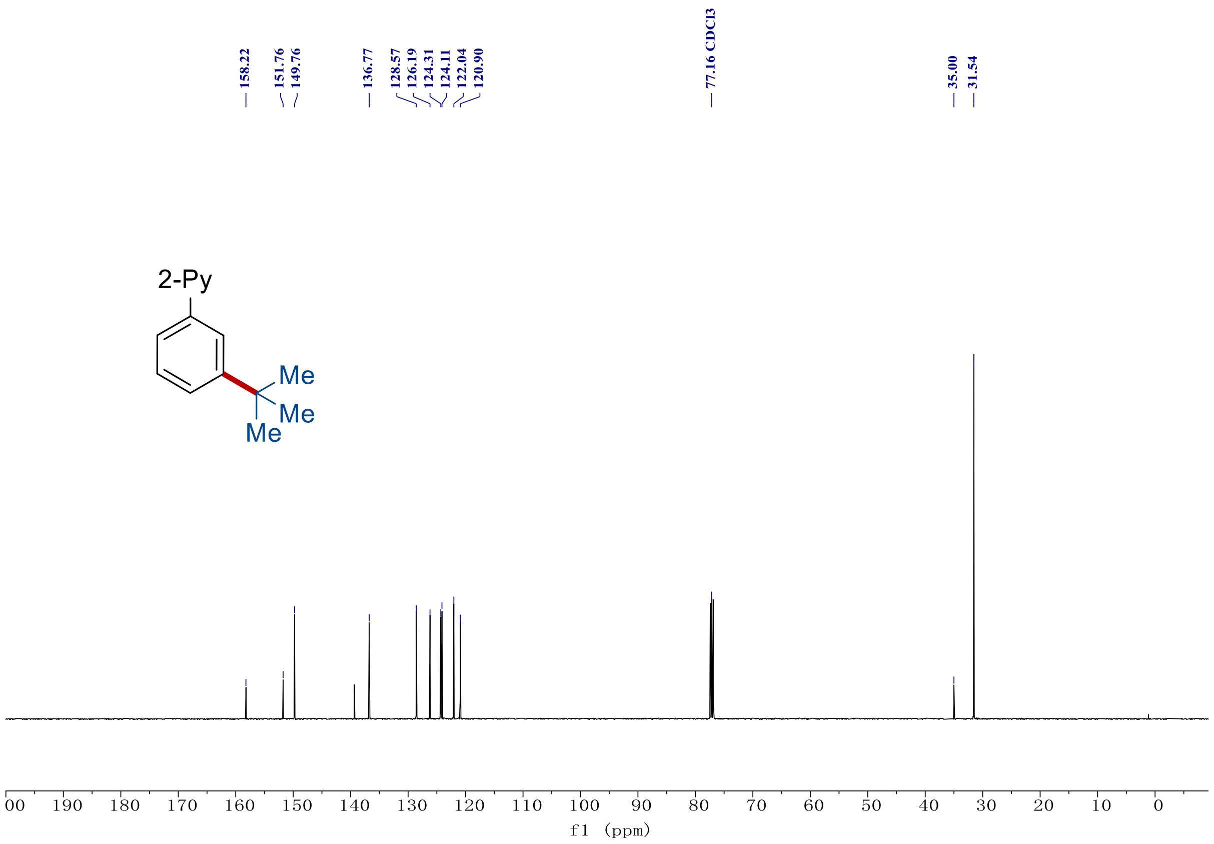

^{1}H NMR of **62a**, 400 MHz, $CDCl_3$, 25 °C.

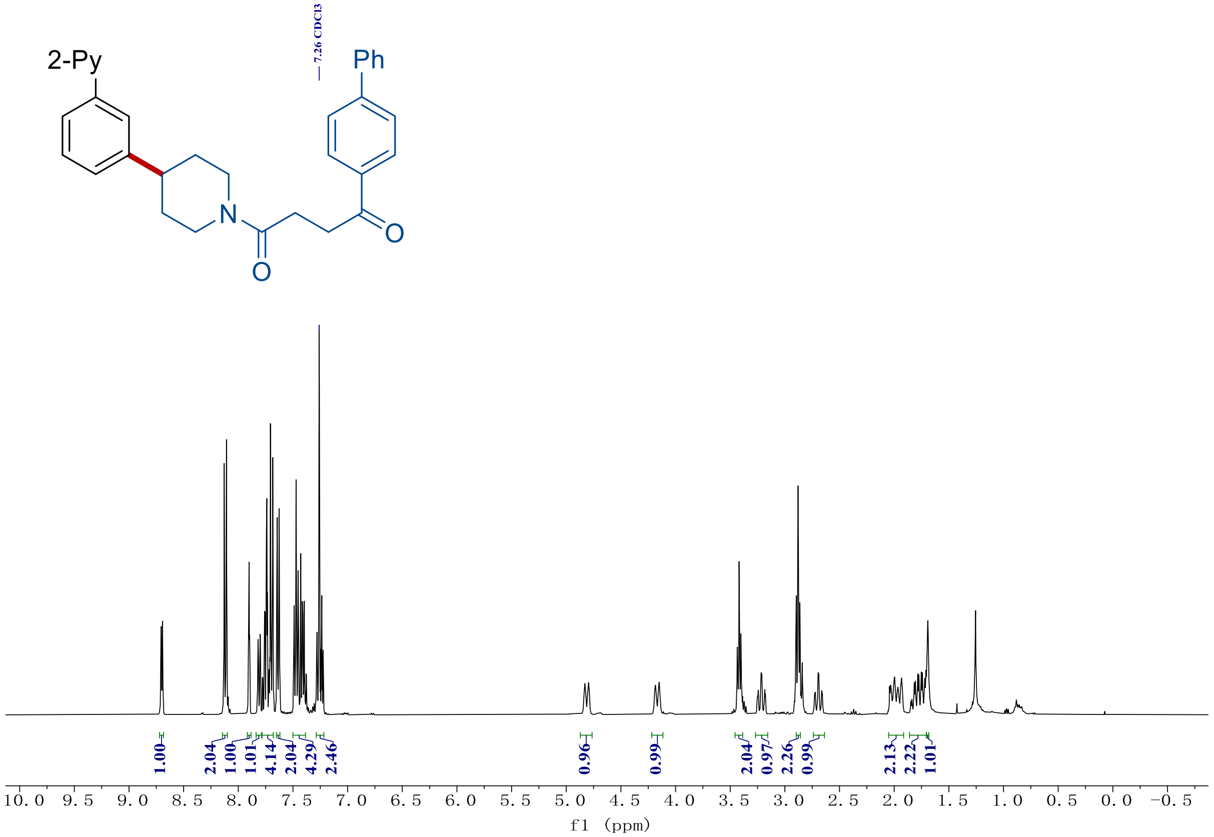

^{13}C NMR of **62a**, 101 MHz, $CDCl_3$, 25 °C.

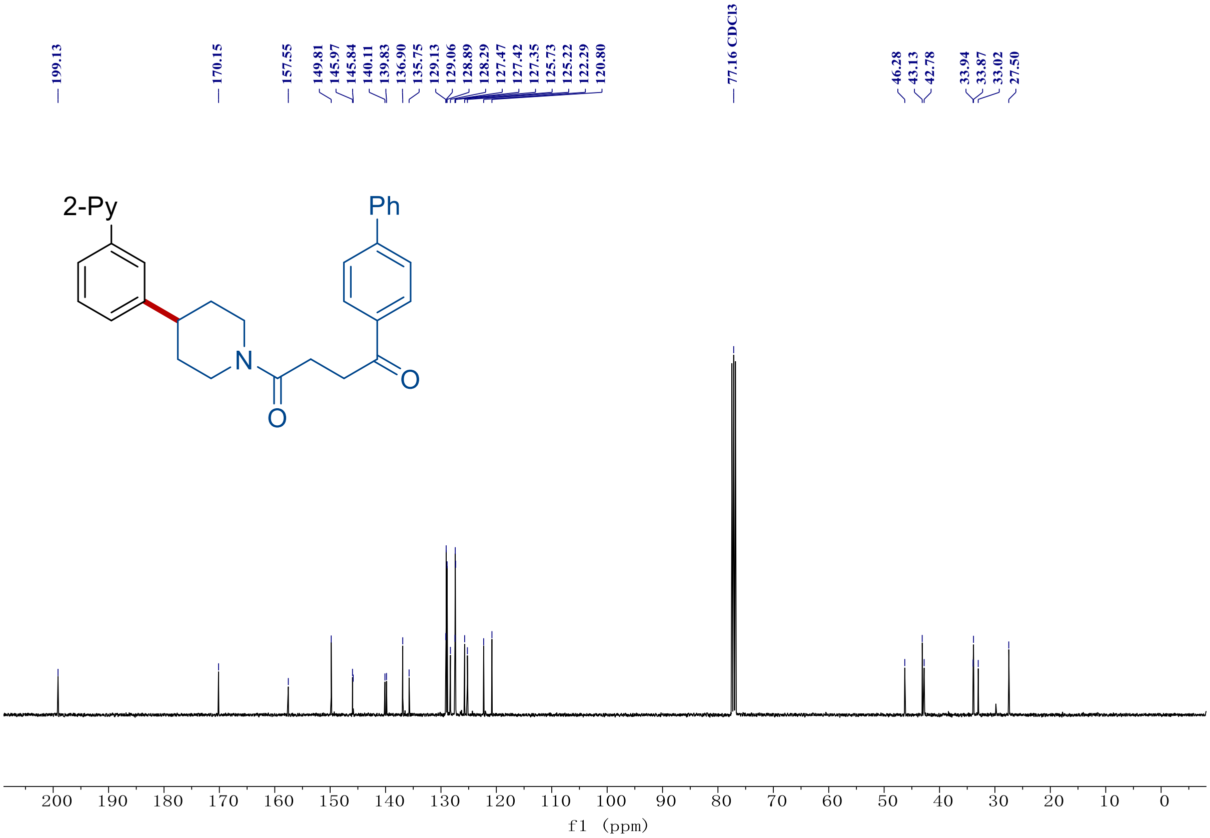

^{1}H NMR of **63a**, 400 MHz, $CDCl_3$, 25 °C.

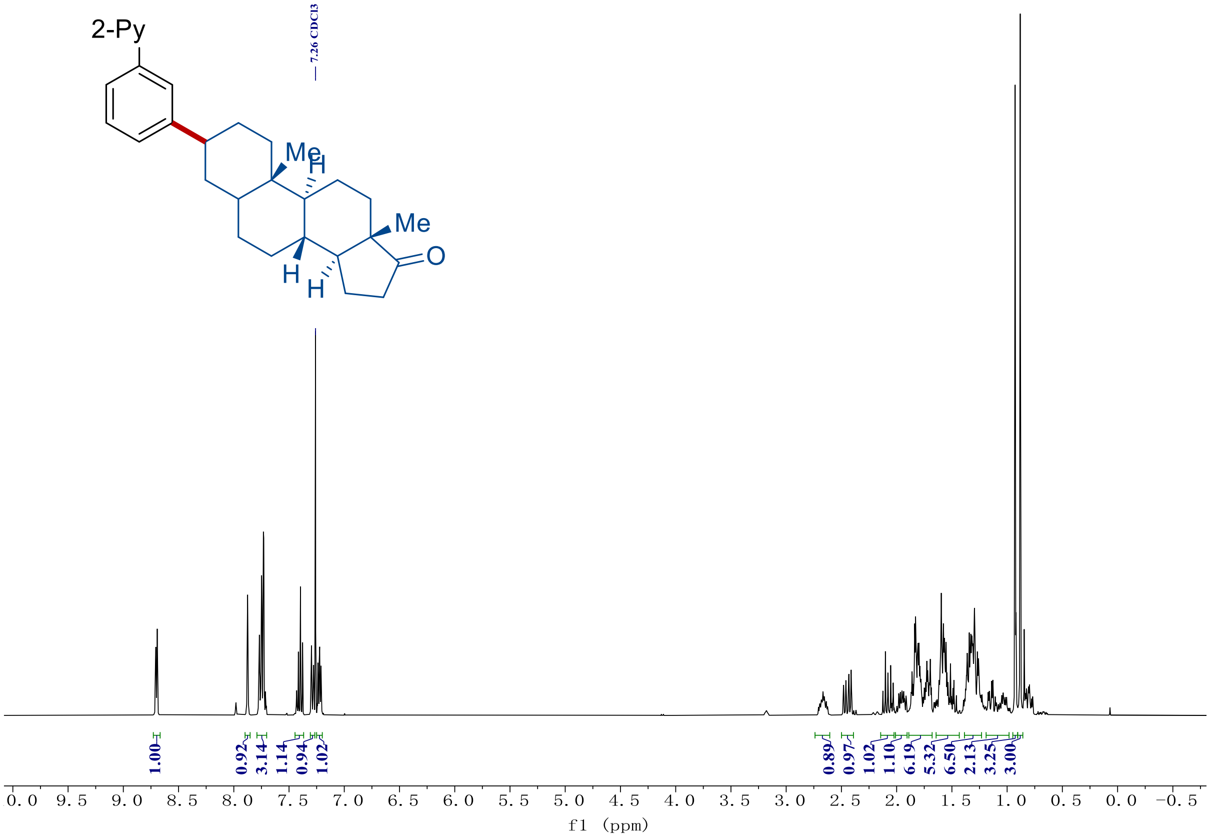

^{13}C NMR of **63a**, 101 MHz, $CDCl_3$, 25 °C.

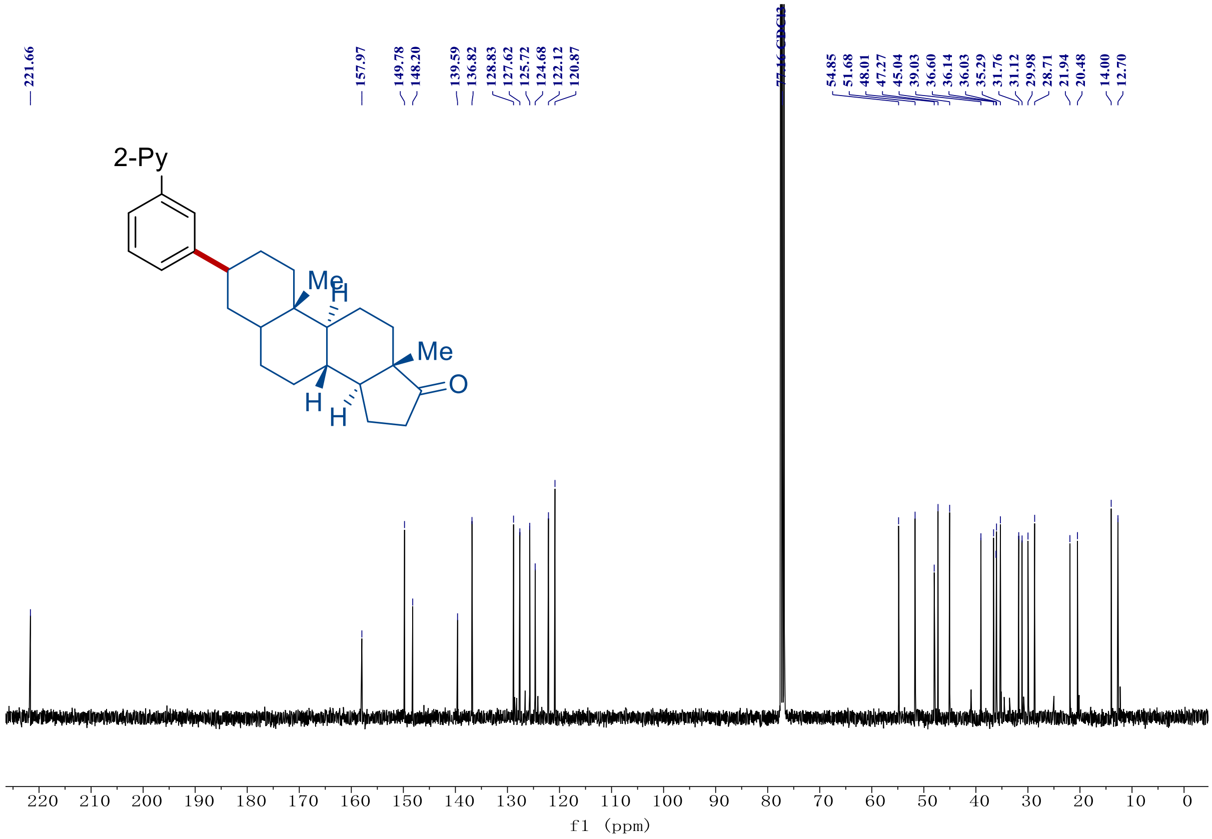

^{1}H NMR of **64a**, 300 MHz, $CDCl_3$, 25 °C.

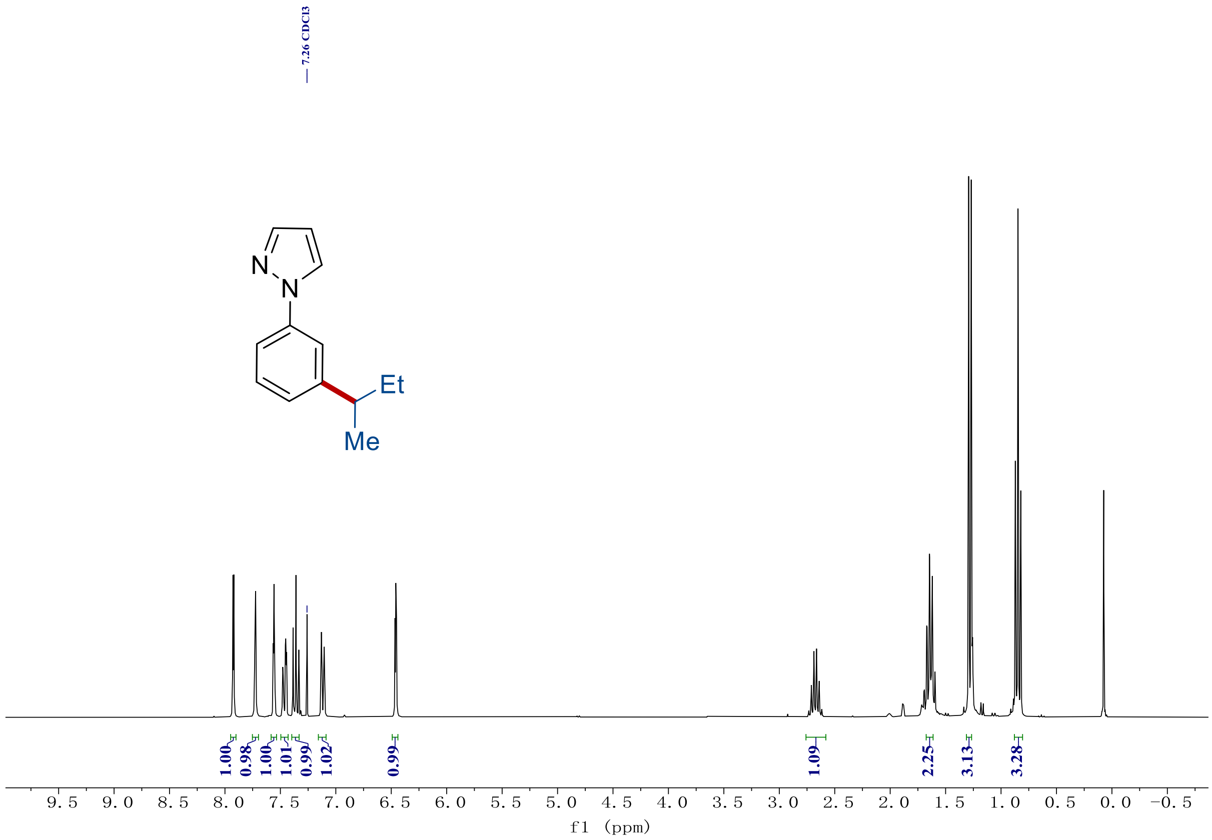

^{13}C NMR of **64a**, 101 MHz, $CDCl_3$, 25 °C.

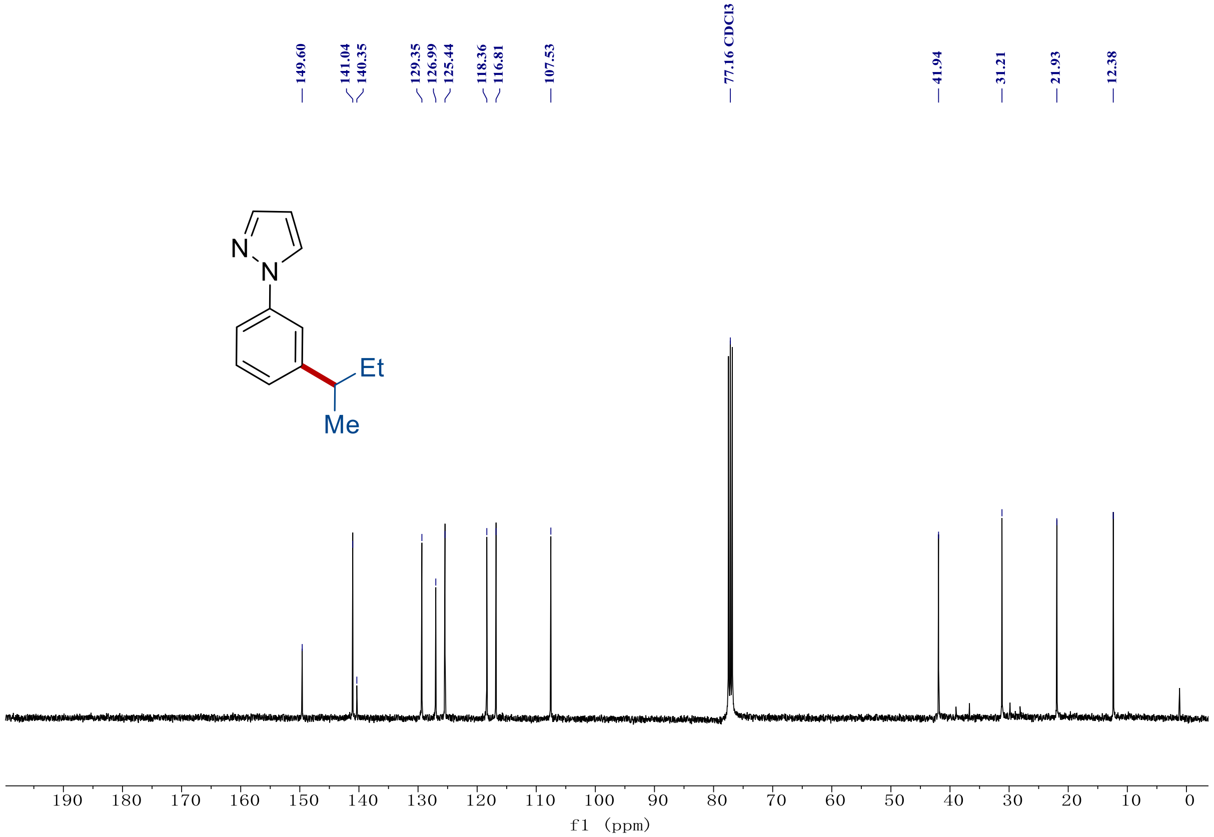

^{1}H NMR of **65a**, 300 MHz, $CDCl_3$, 25 °C.

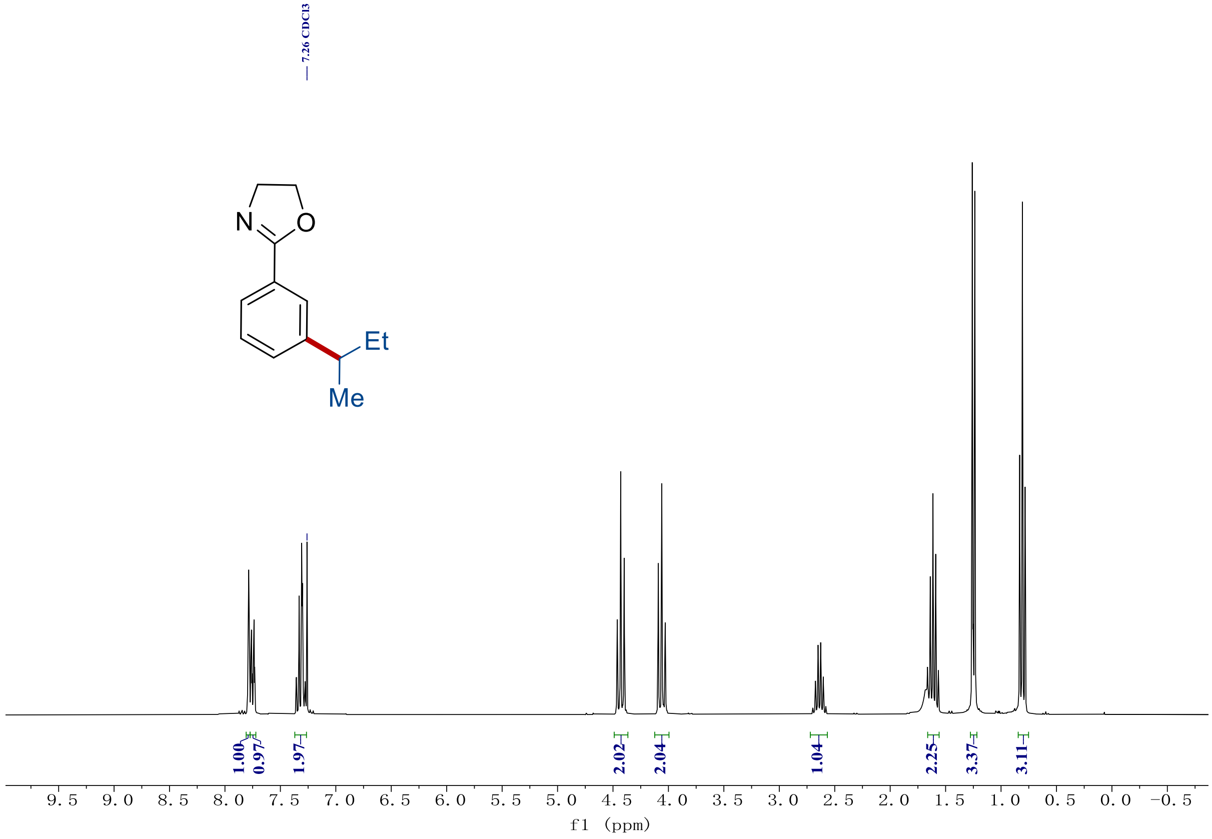

^{13}C NMR of **65a**, 75 MHz, $CDCl_3$, 25 °C.

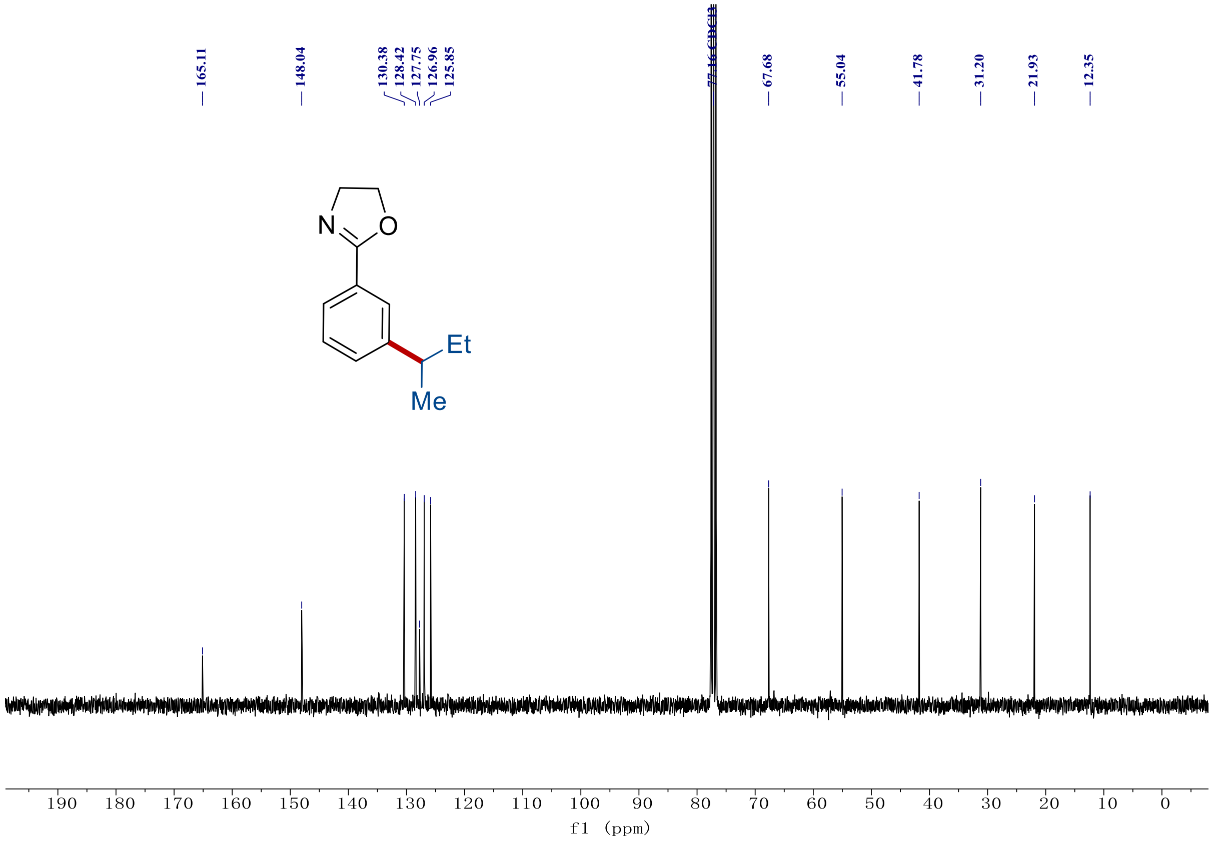

^{1}H NMR of **68**, 400 MHz, $CDCl_3$, 25 °C.

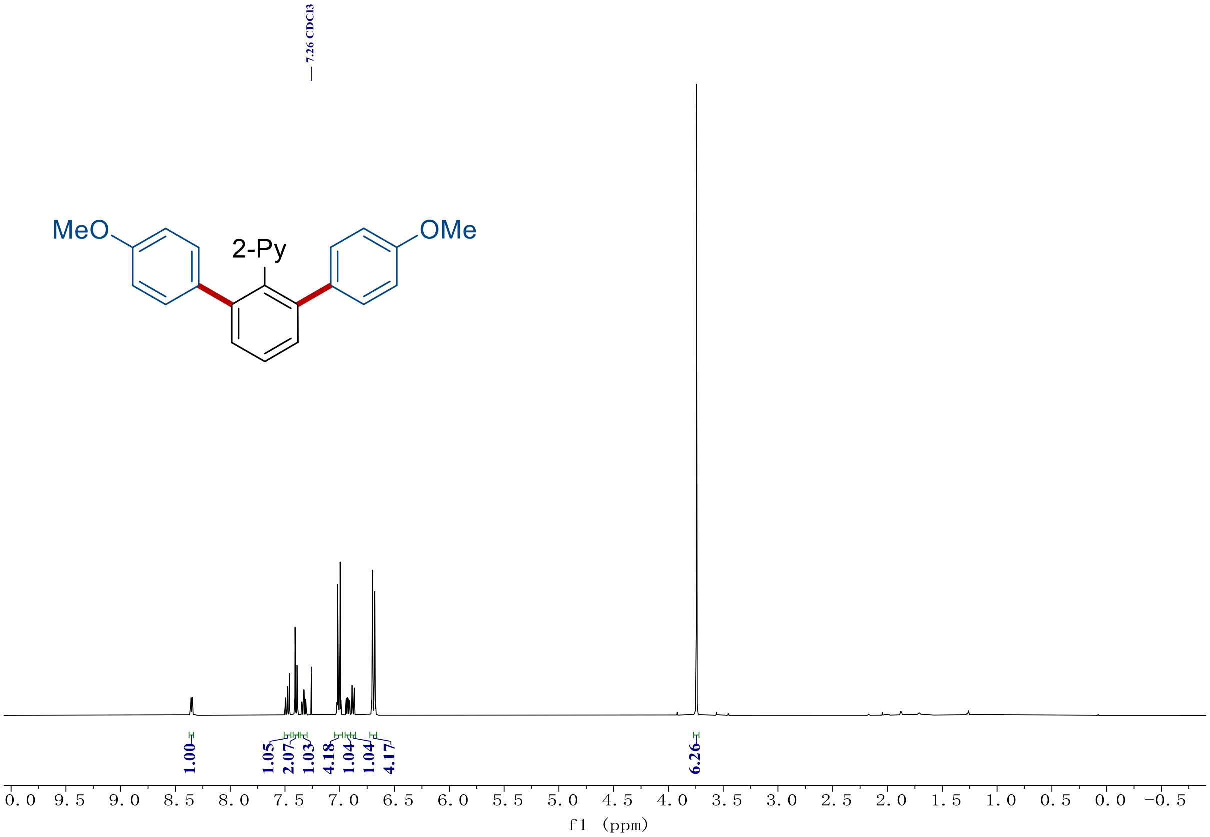

^{13}C NMR of **68**, 101 MHz, $CDCl_3$, 25 °C.

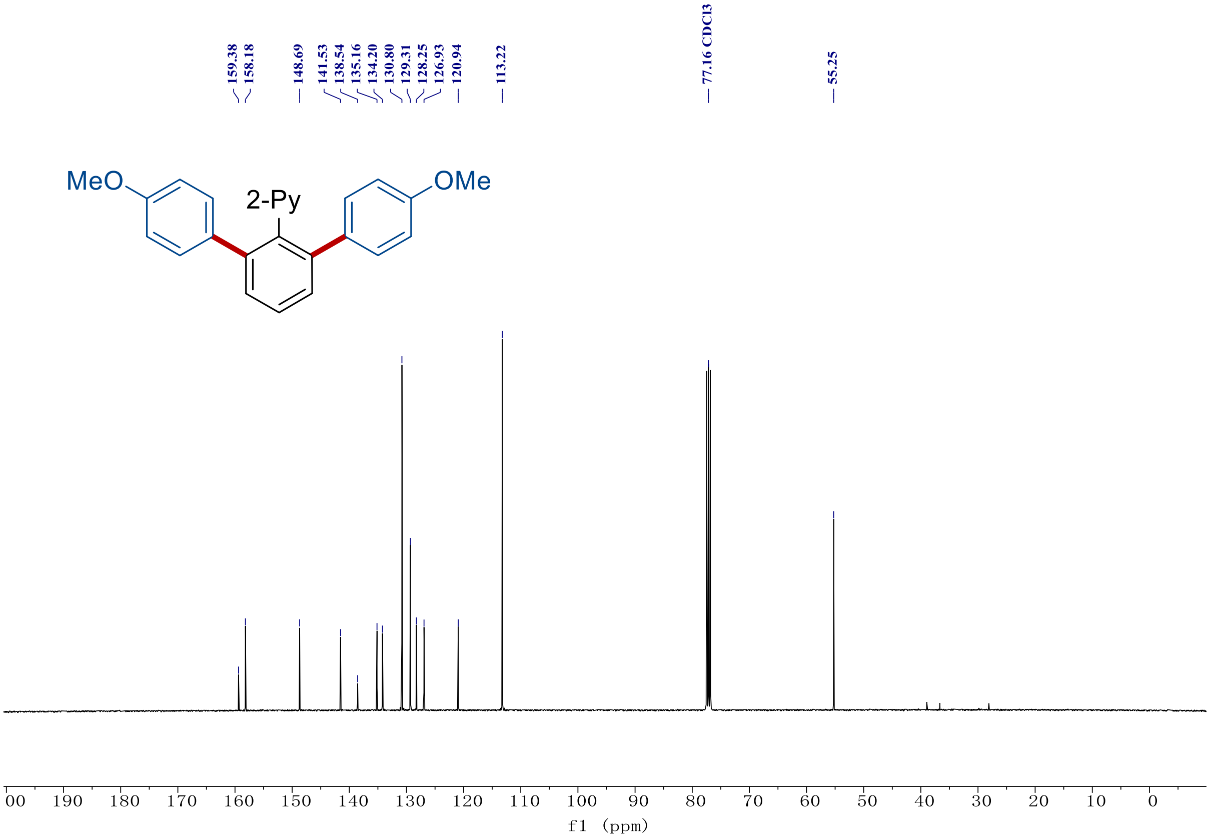

^{1}H NMR of **69**, 500 MHz, $CDCl_3$, 25 °C.

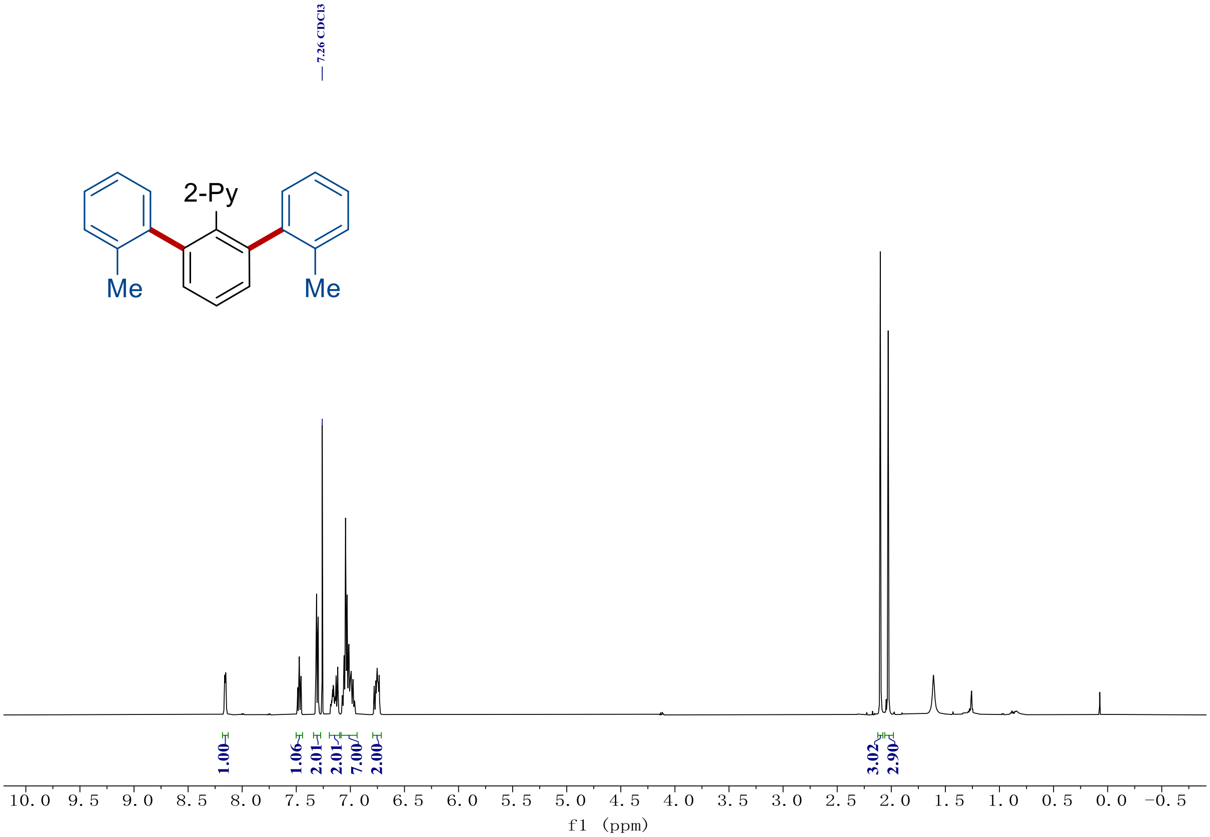

^{13}C NMR of **69**, 126 MHz, $CDCl_3$, 25 °C.

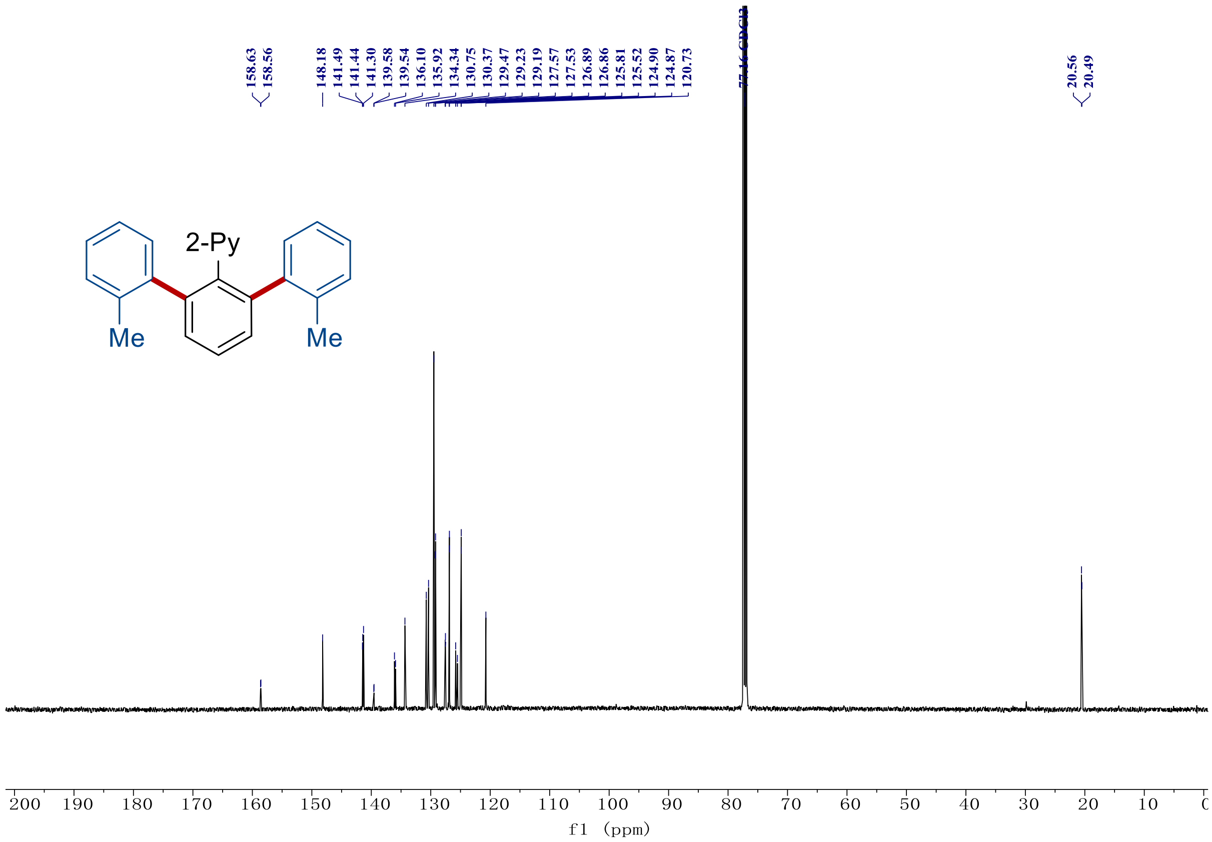

^{1}H NMR of **70**, 500 MHz, $CDCl_3$, 25 °C.

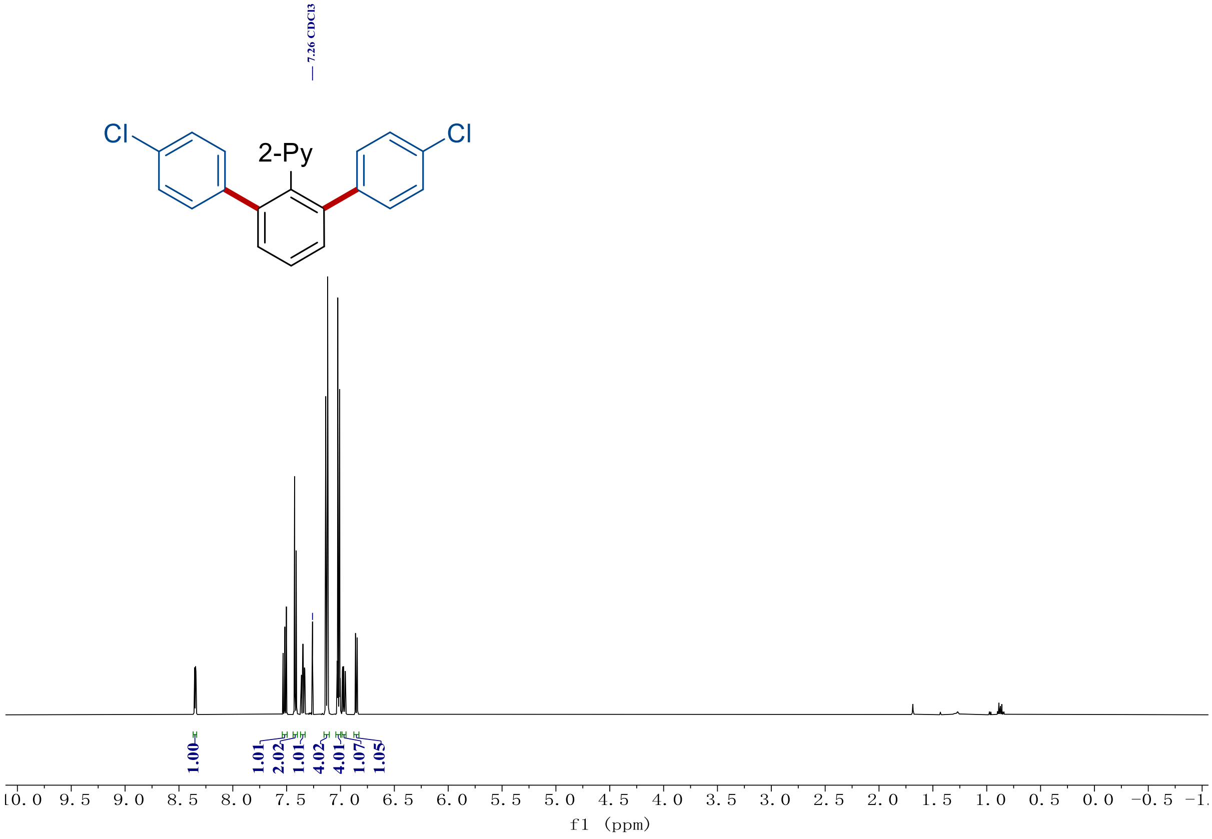

^{13}C NMR of **70**, 126 MHz, $CDCl_3$, 25 °C.

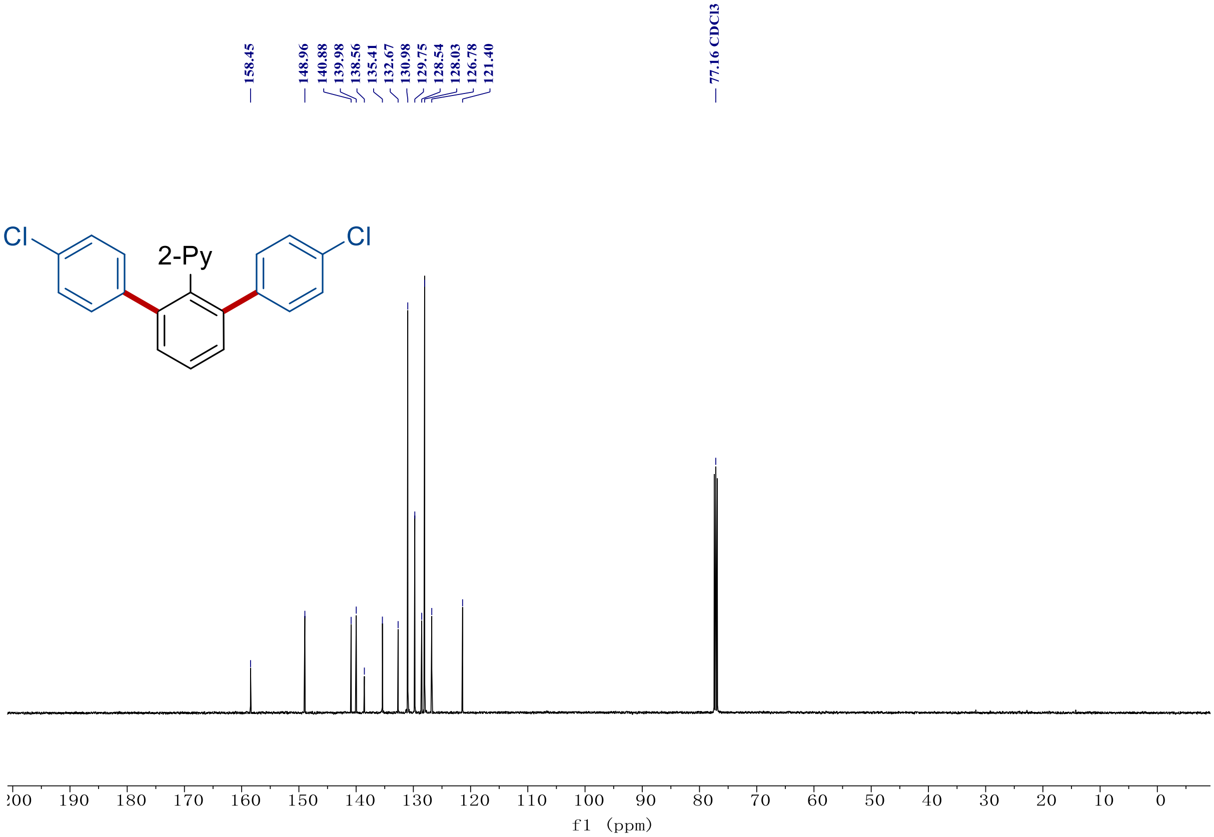

^{1}H NMR of **71**, 300 MHz, $CDCl_3$, 25 °C.

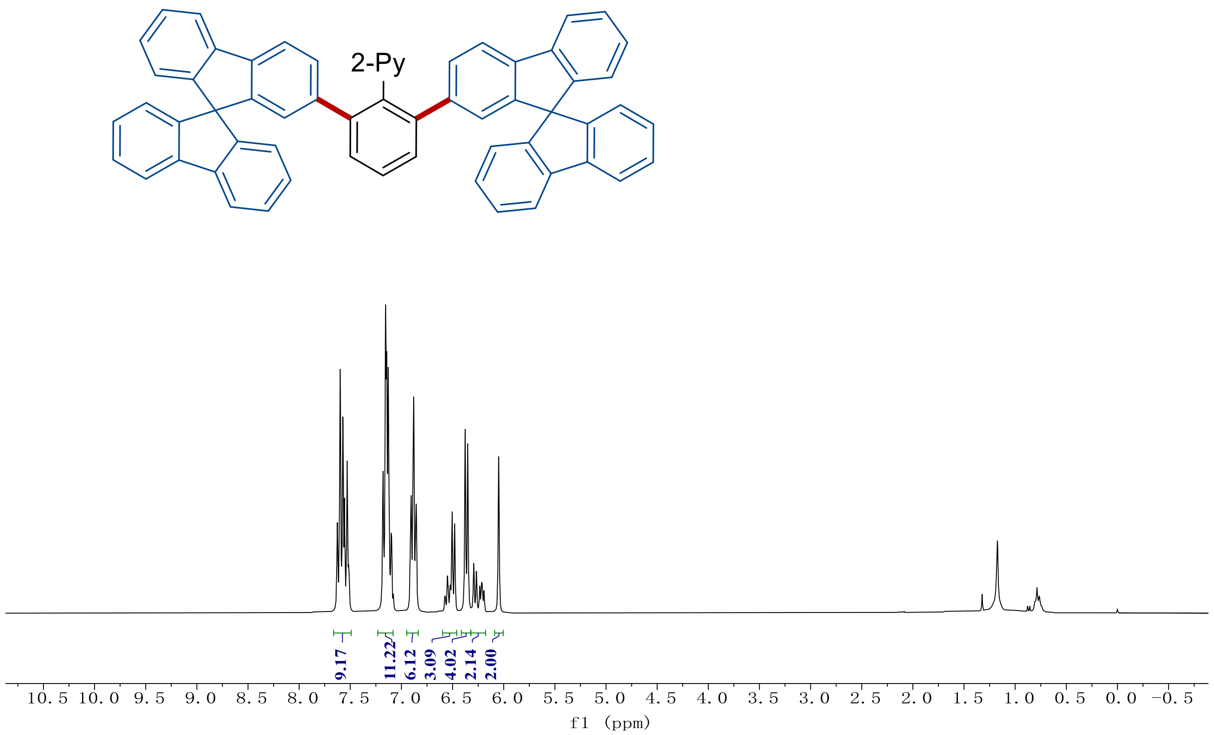

^{13}C NMR of **71**, 75 MHz, $CDCl_3$, 25 °C.

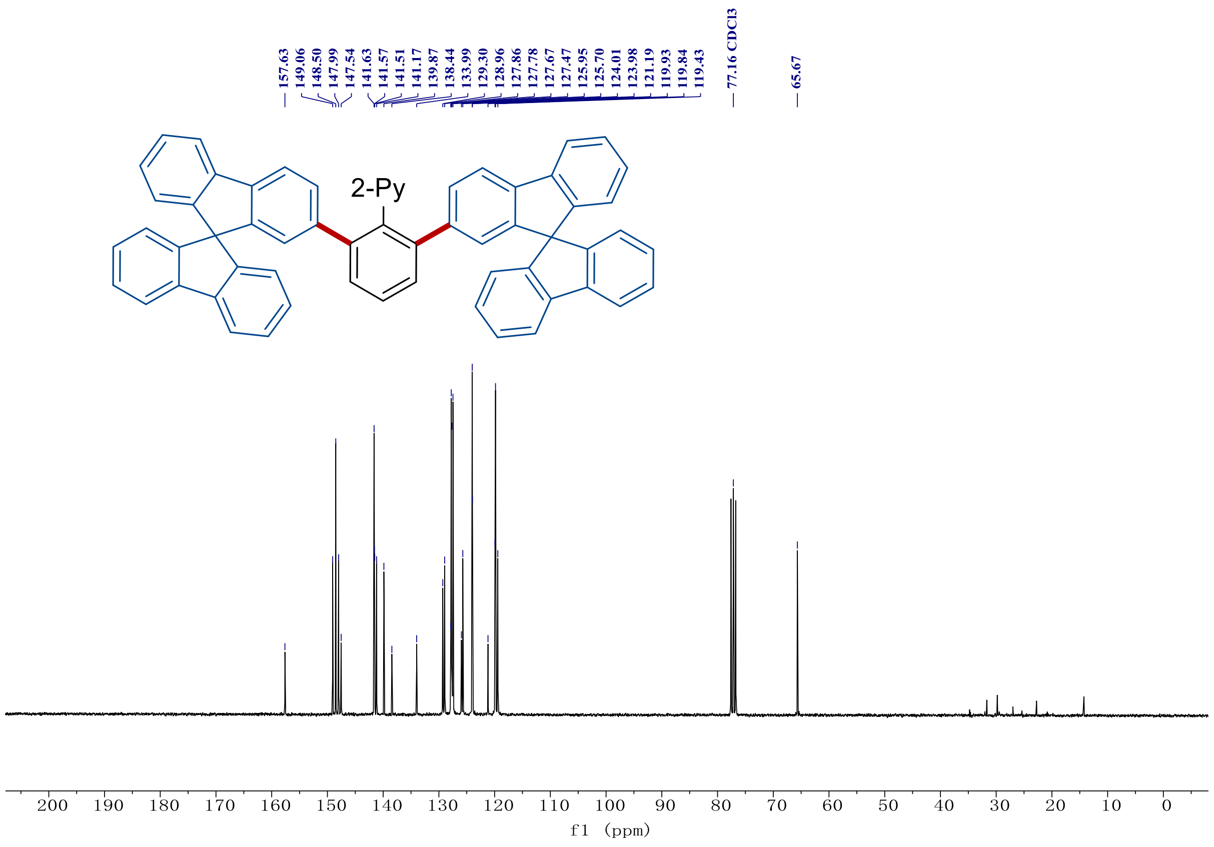

^{1}H NMR of **72a**, 300 MHz, $CDCl_3$, 25 °C.

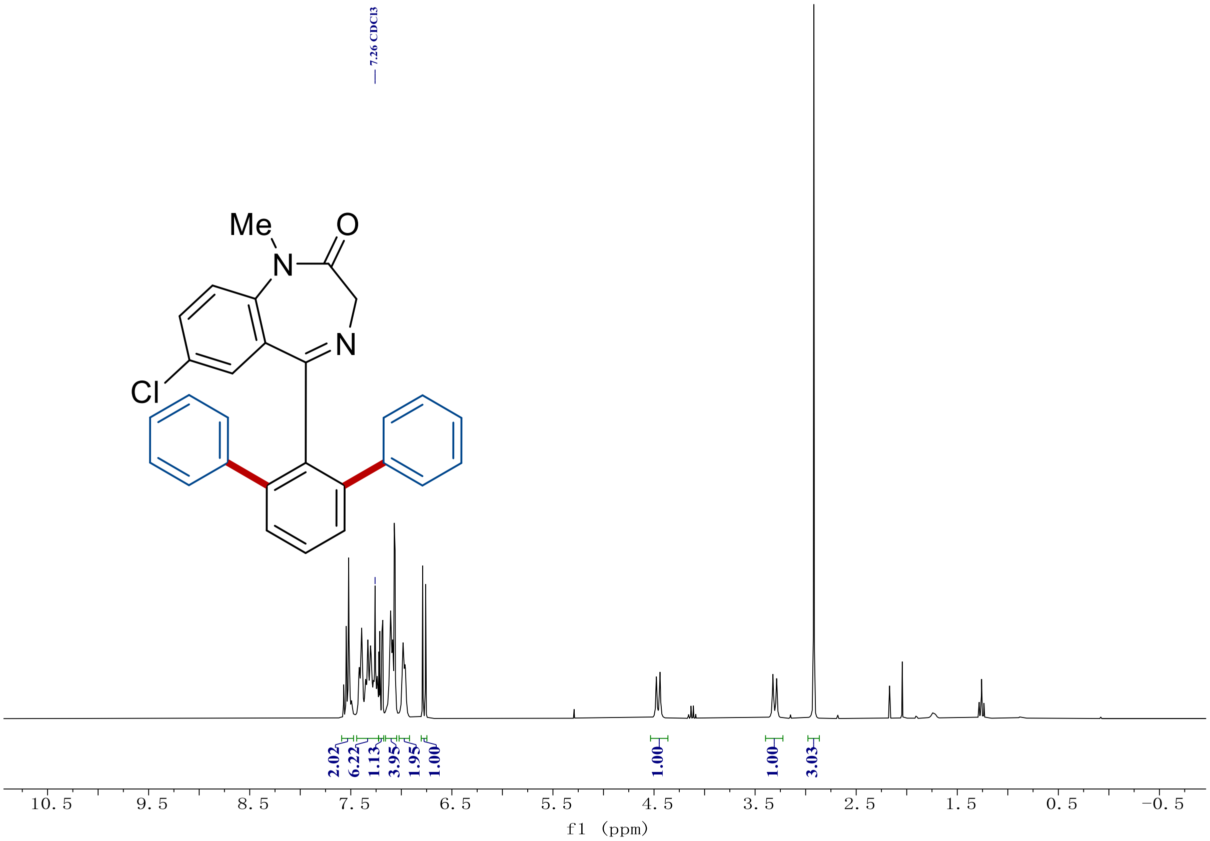

^{13}C NMR of **72a**, 75 MHz, $CDCl_3$, 25 °C.

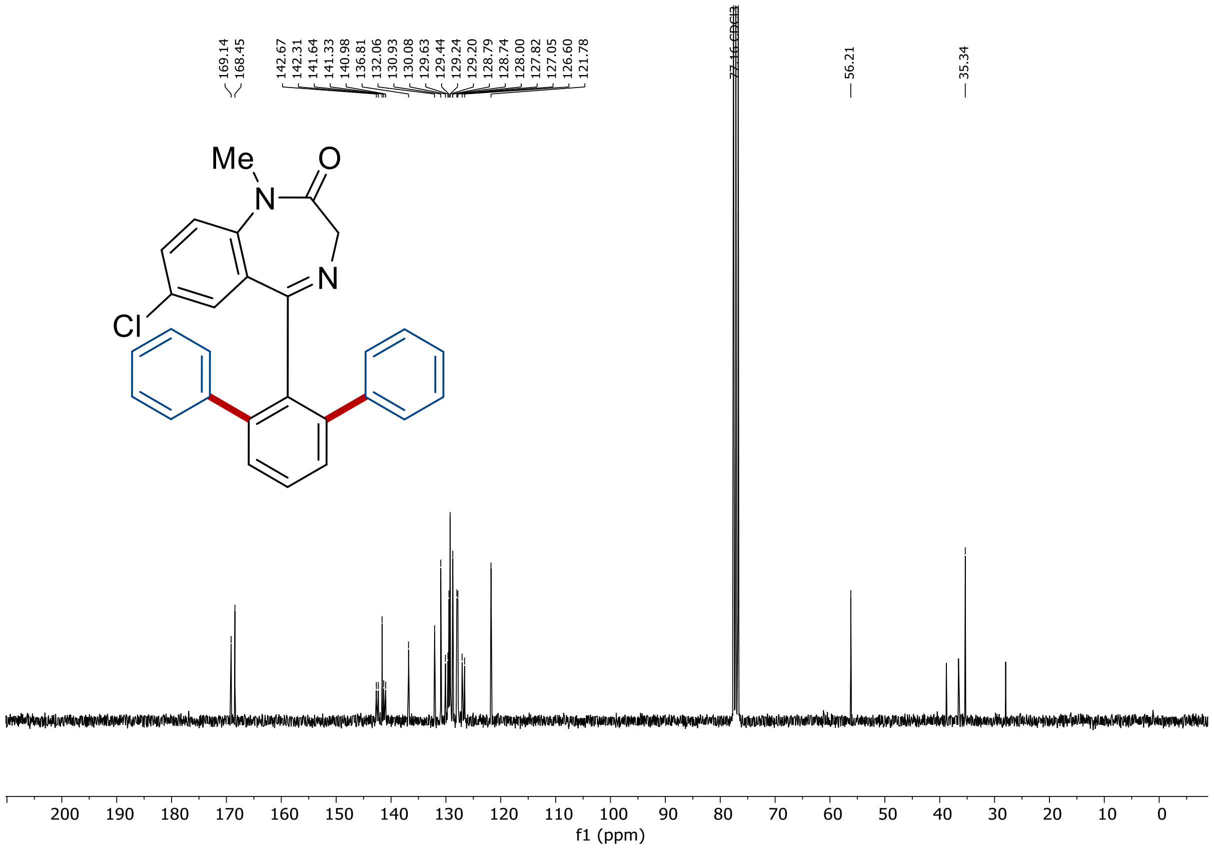

^{1}H NMR of **72b**, 300 MHz, $CDCl_3$, 25 °C.

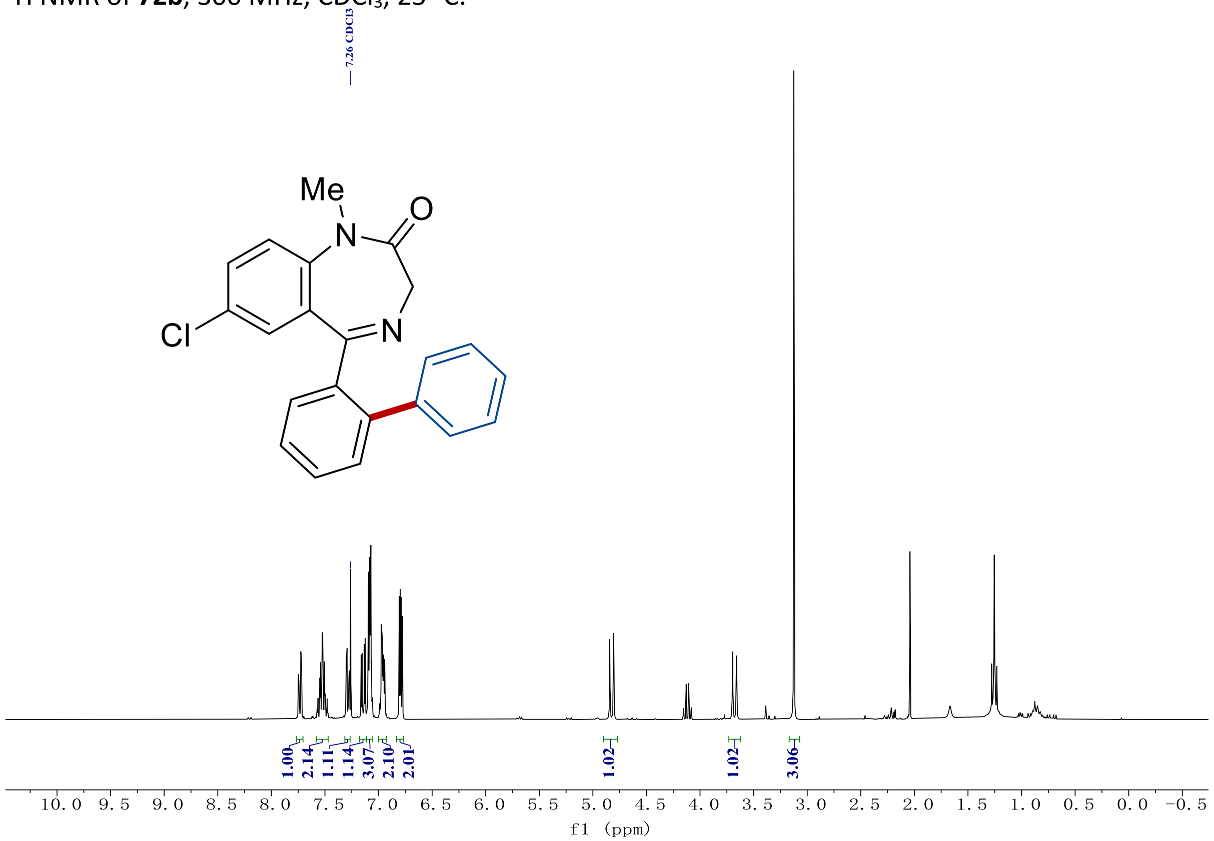

^{13}C NMR of **72b**, 75 MHz, $CDCl_3$, 25 °C.

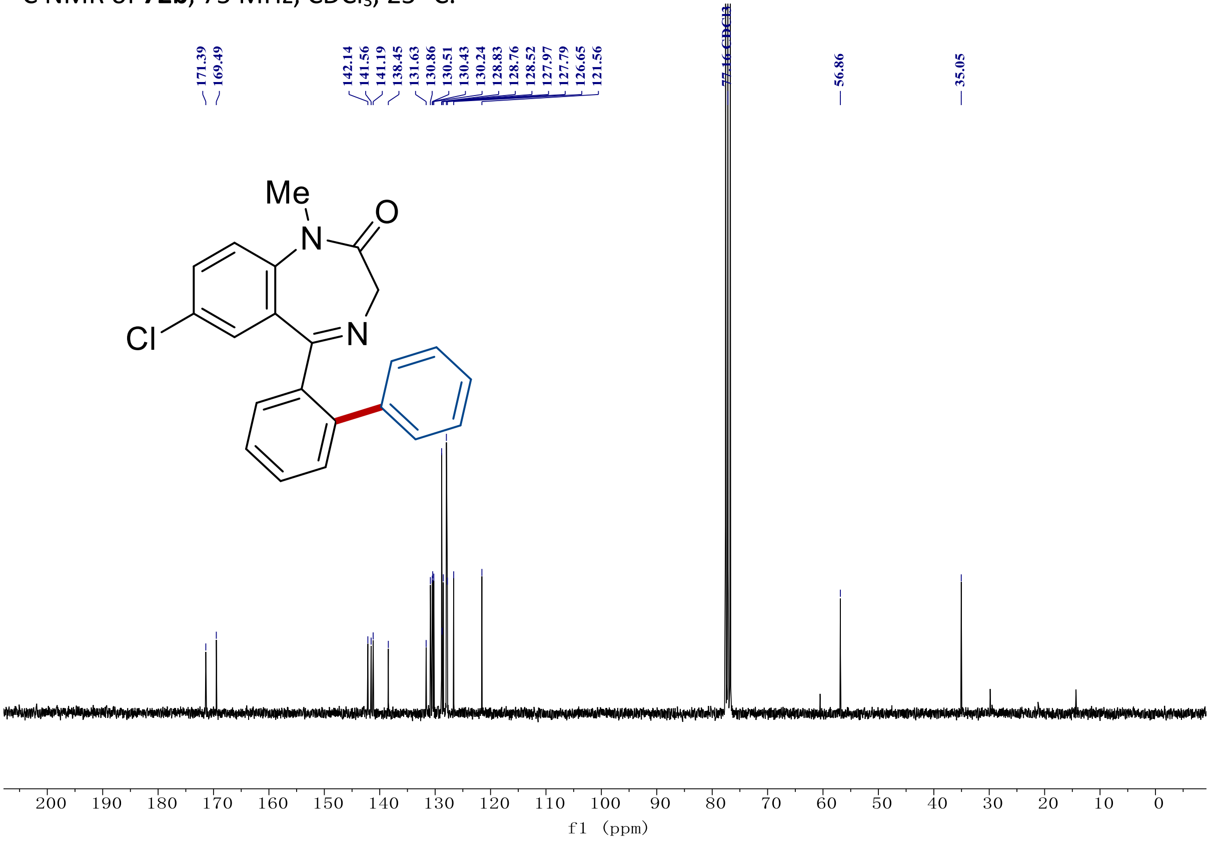

^{1}H NMR of **73**, 300 MHz, $CDCl_3$, 25 °C.

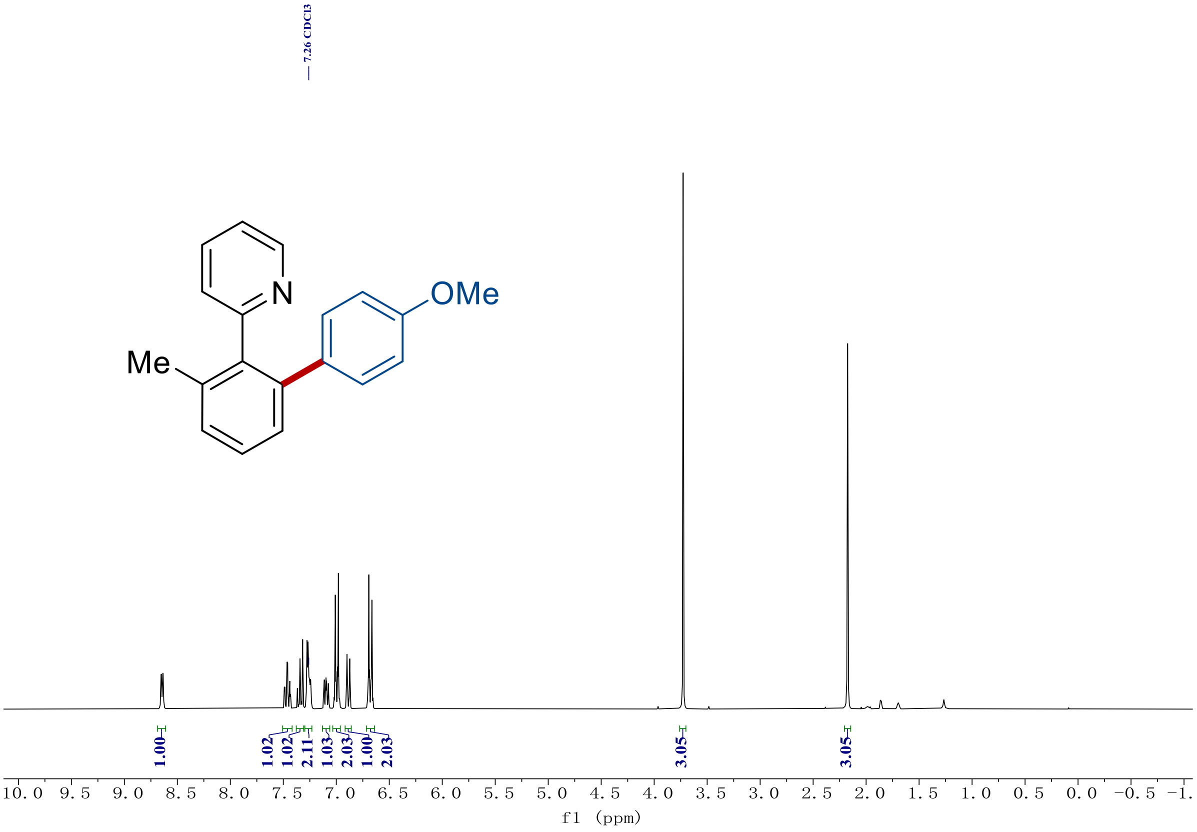

^{13}C NMR of **73**, 75 MHz, $CDCl_3$, 25 °C.

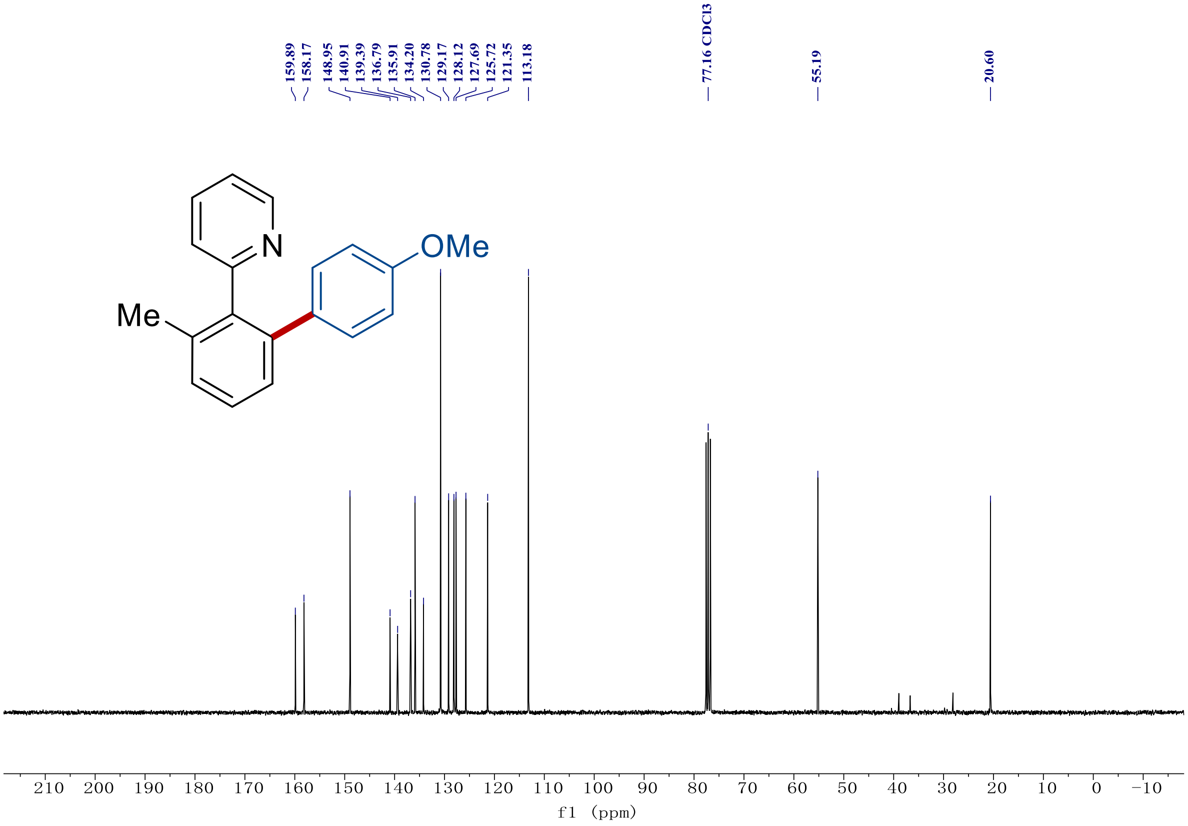

^{1}H NMR of **74**, 300 MHz, $CDCl_3$, 25 °C.

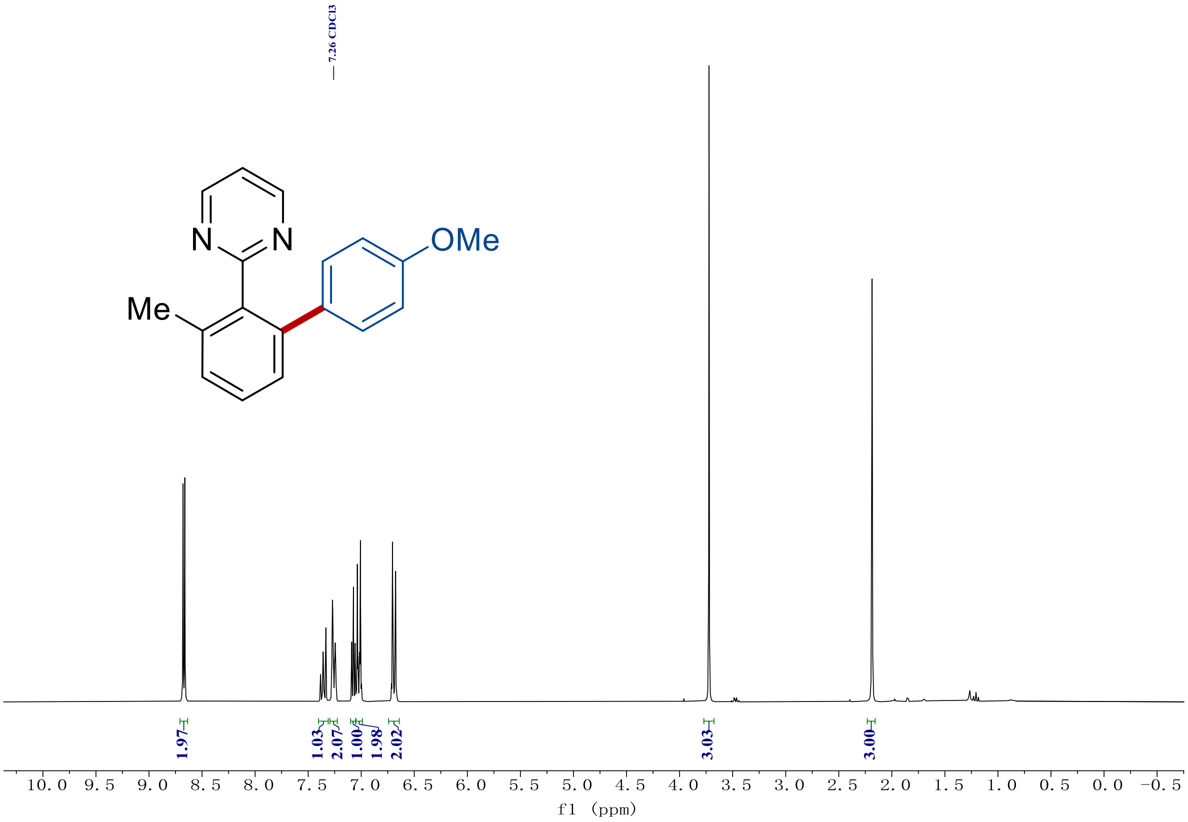

^{13}C NMR of **74**, 75 MHz, $CDCl_3$, 25 °C.

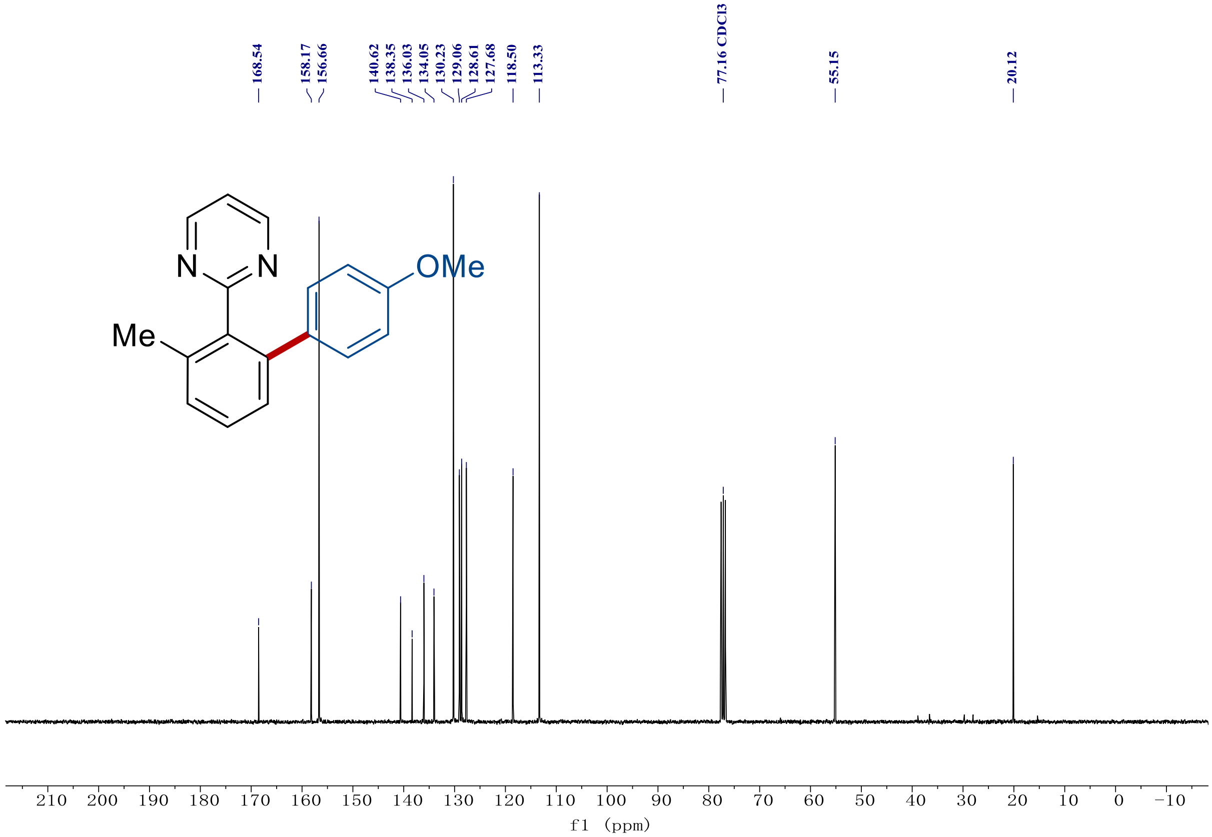

^{1}H NMR of **75**, 300 MHz, $CDCl_3$, 25 °C.

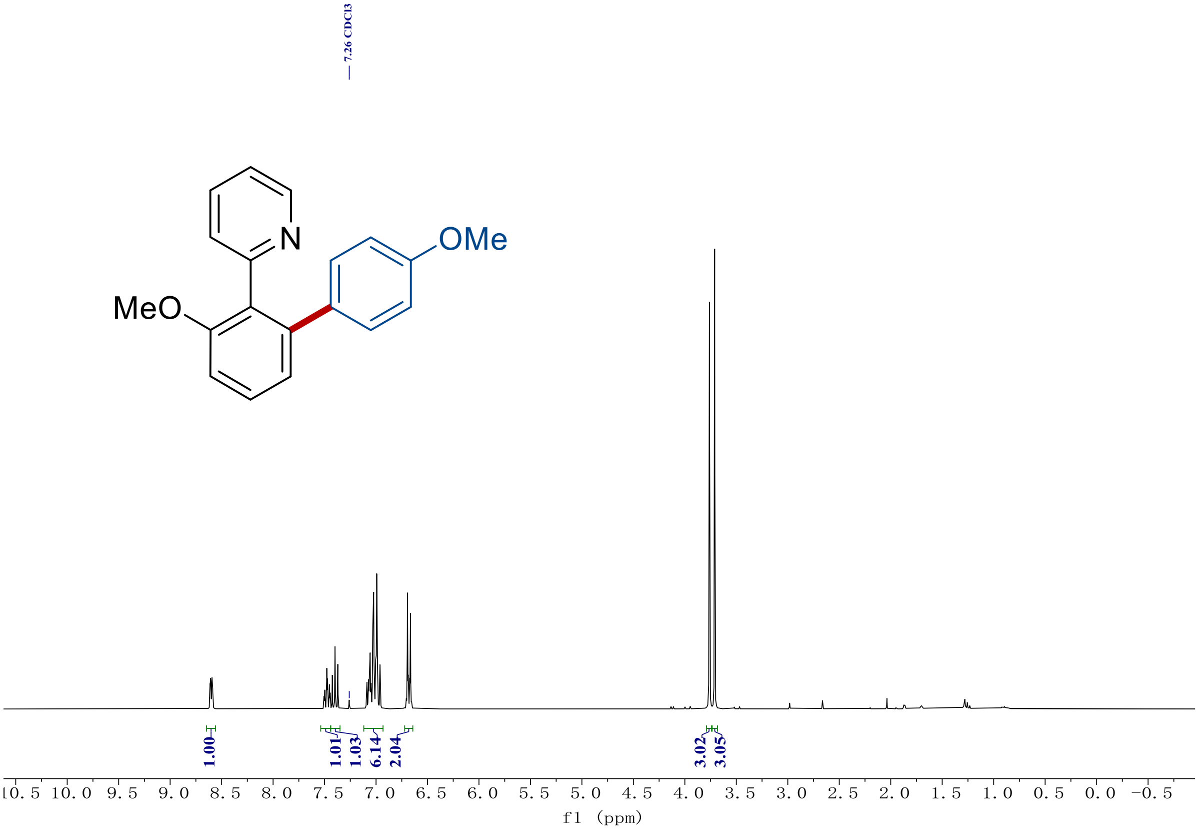

^{13}C NMR of **75**, 75 MHz, $CDCl_3$, 25 °C.

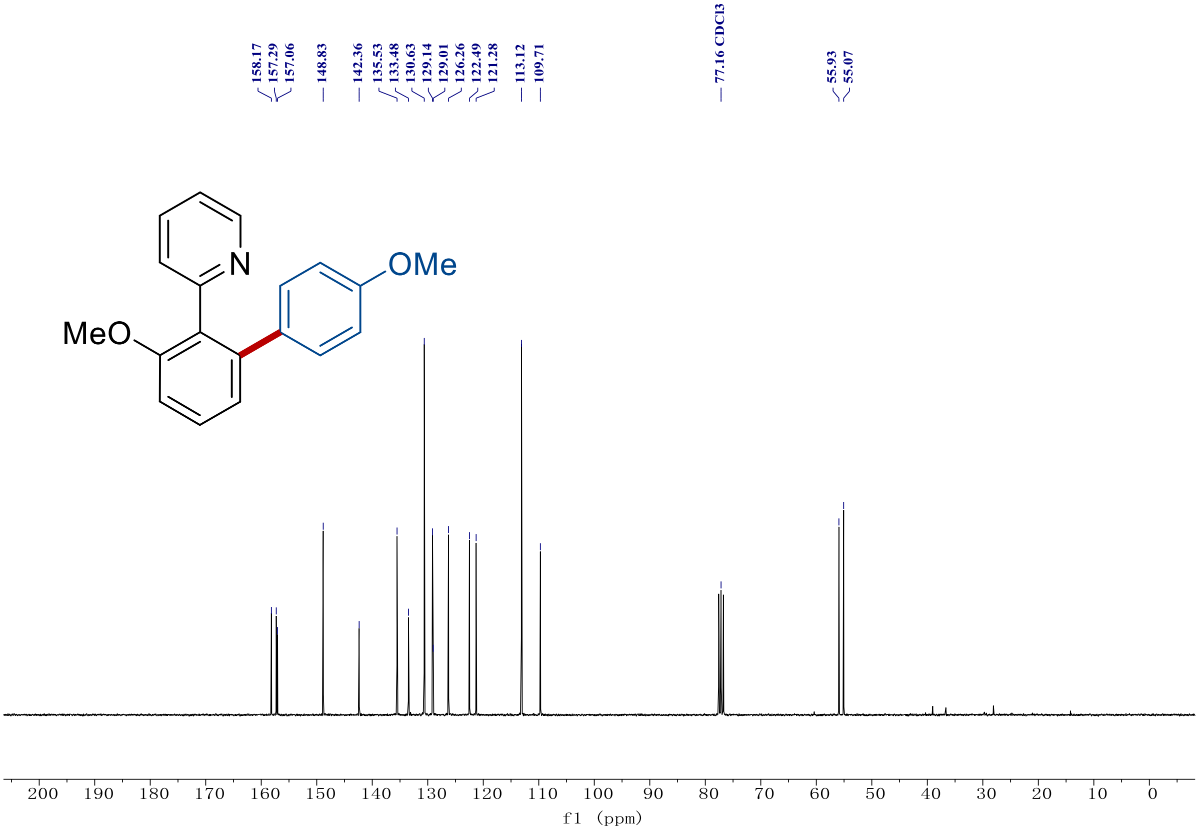

^{1}H NMR of **76**, 300 MHz, $CDCl_3$, 25 °C.

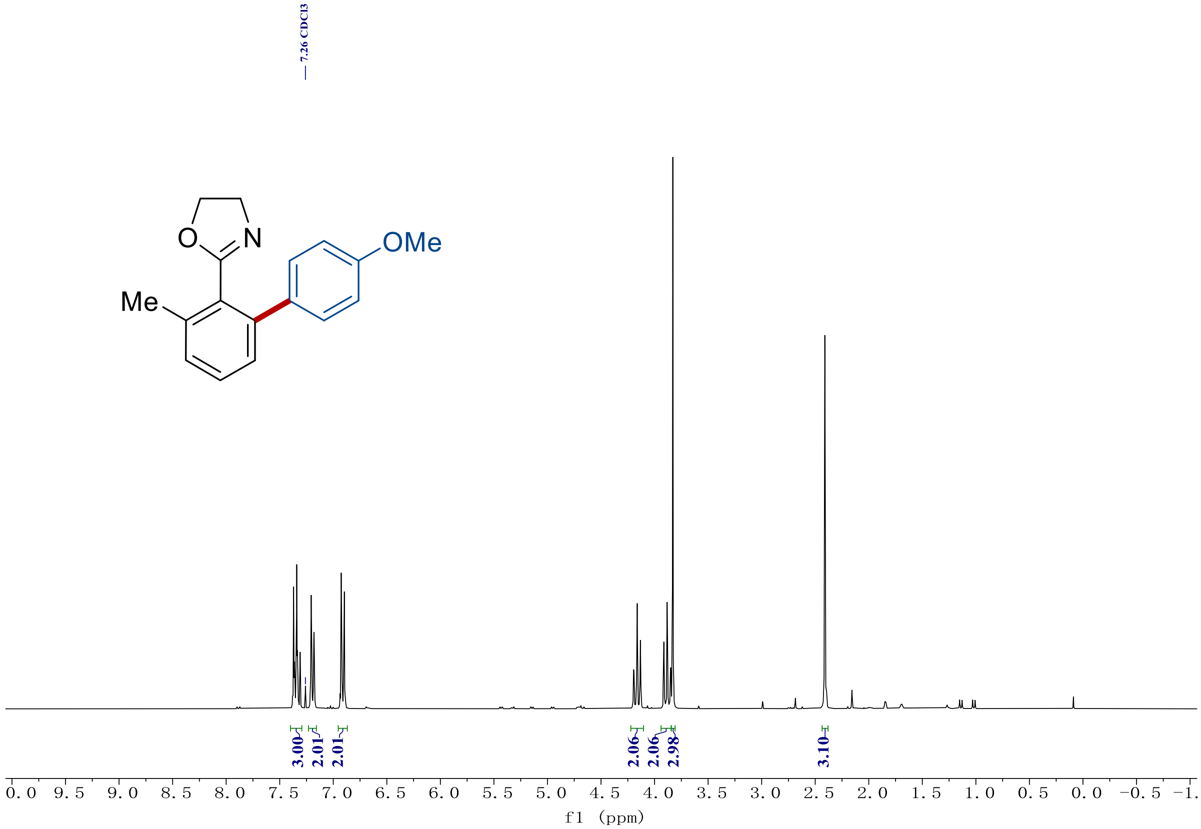

^{13}C NMR of **76**, 75 MHz, $CDCl_3$, 25 °C.

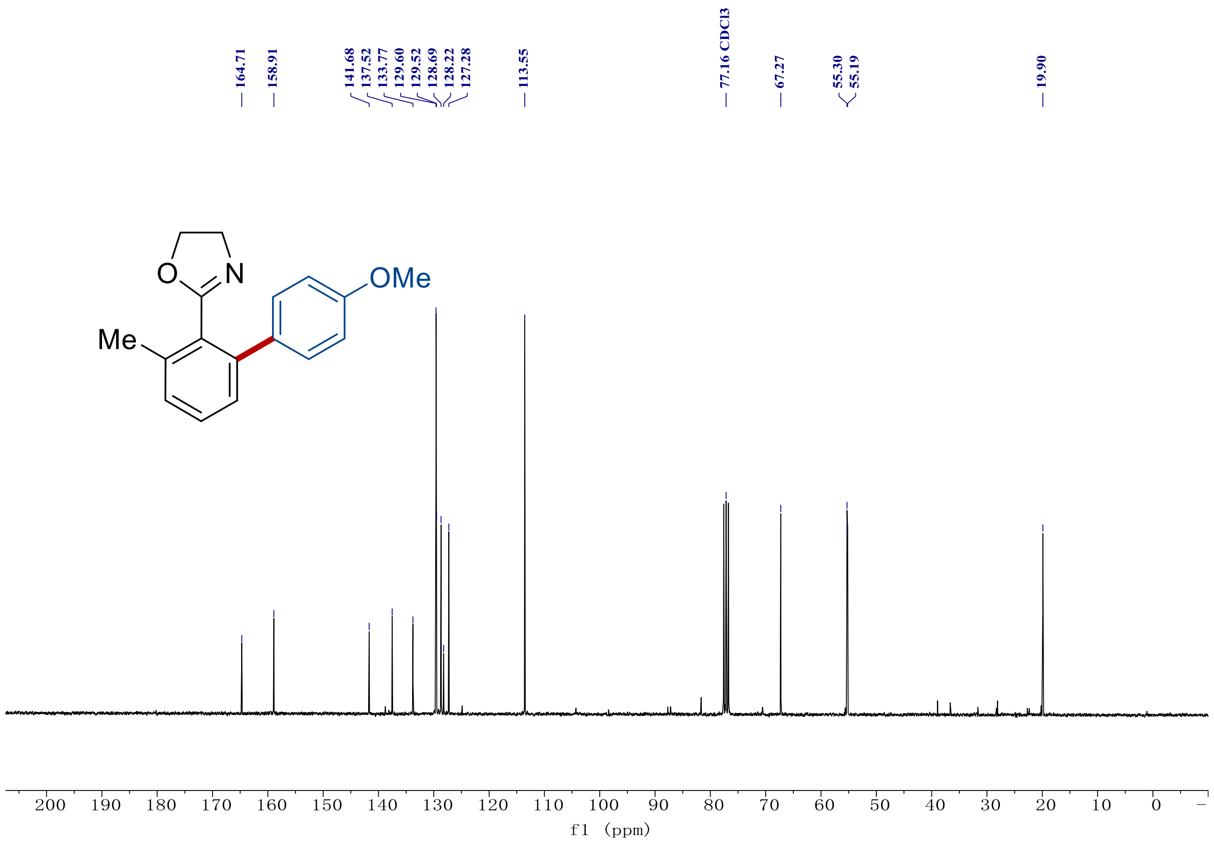

^{1}H NMR of **7**, 400 MHz, CD_2Cl_2, 25 °C.

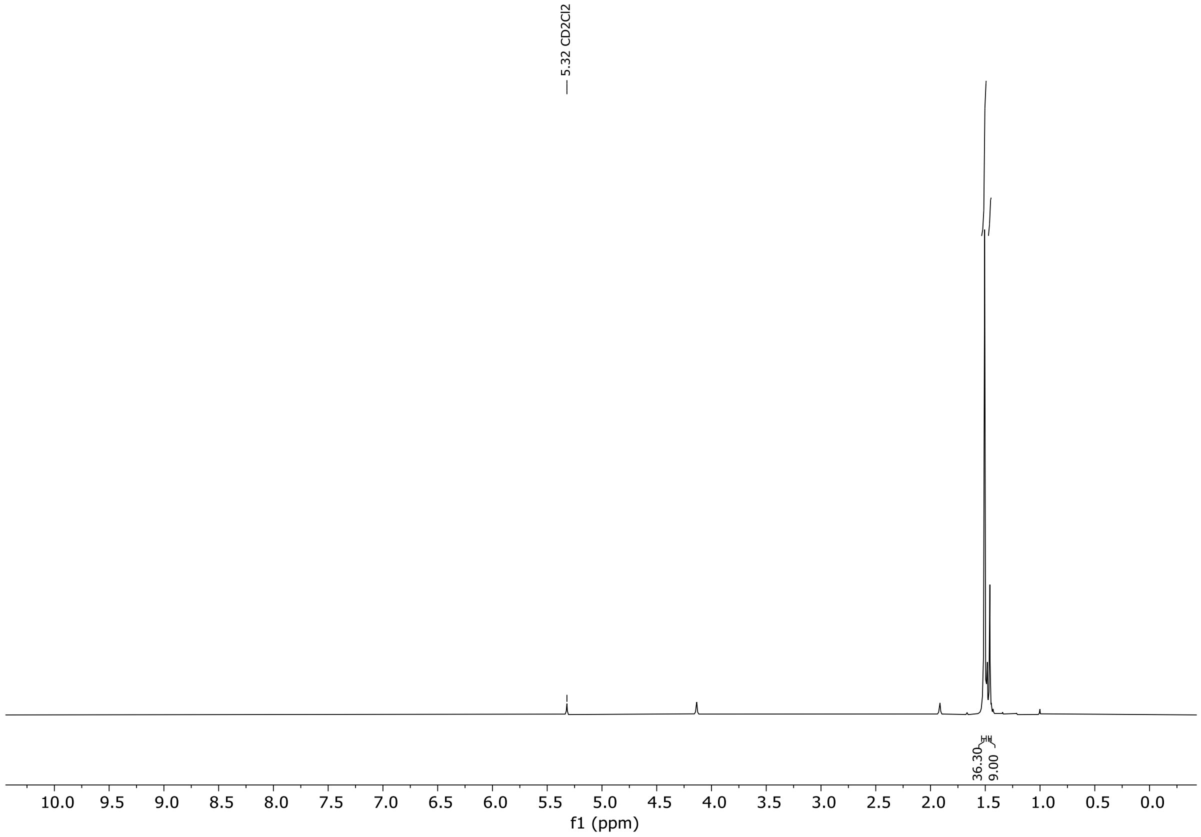

^{13}C NMR of **7**, 100 MHz, $CDCl_3$, 25 °C.

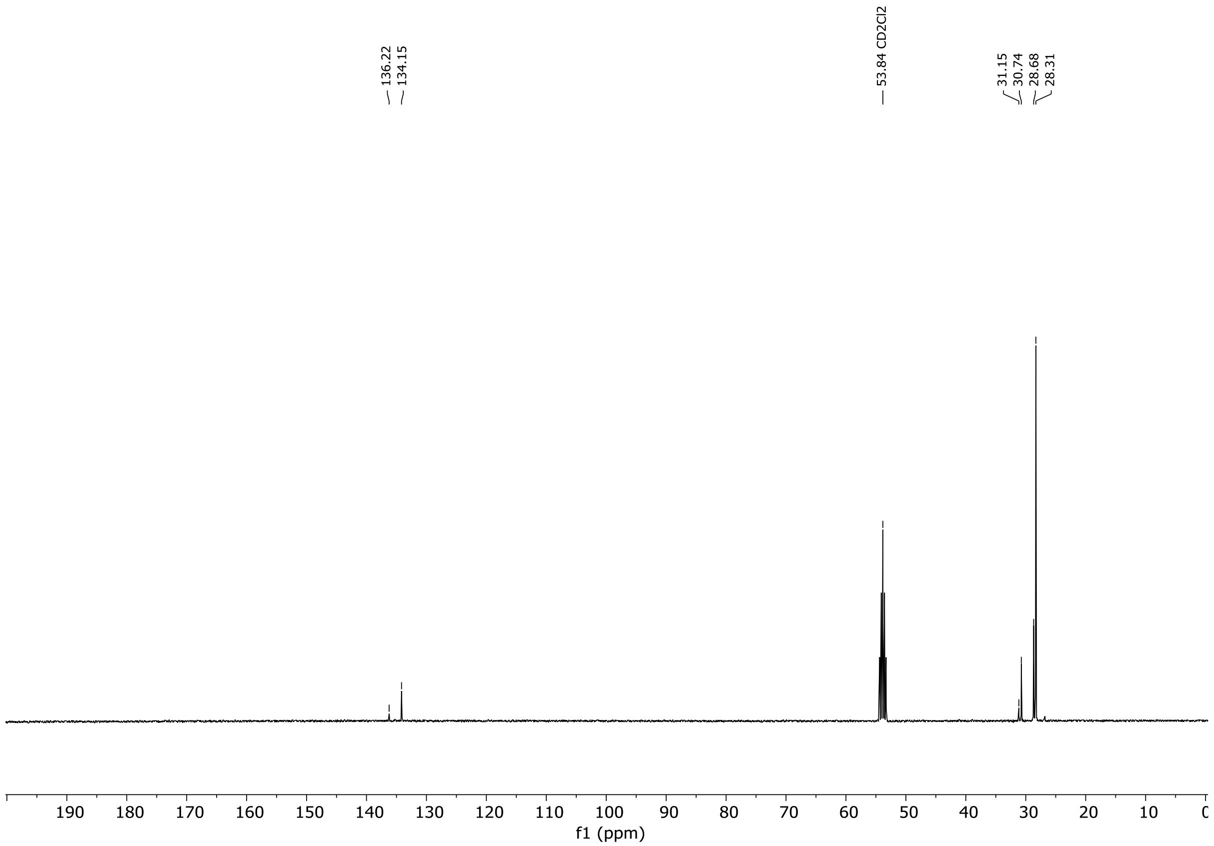

^{19}F NMR of **7**, 377 MHz, CD_2Cl_2, 25 °C.

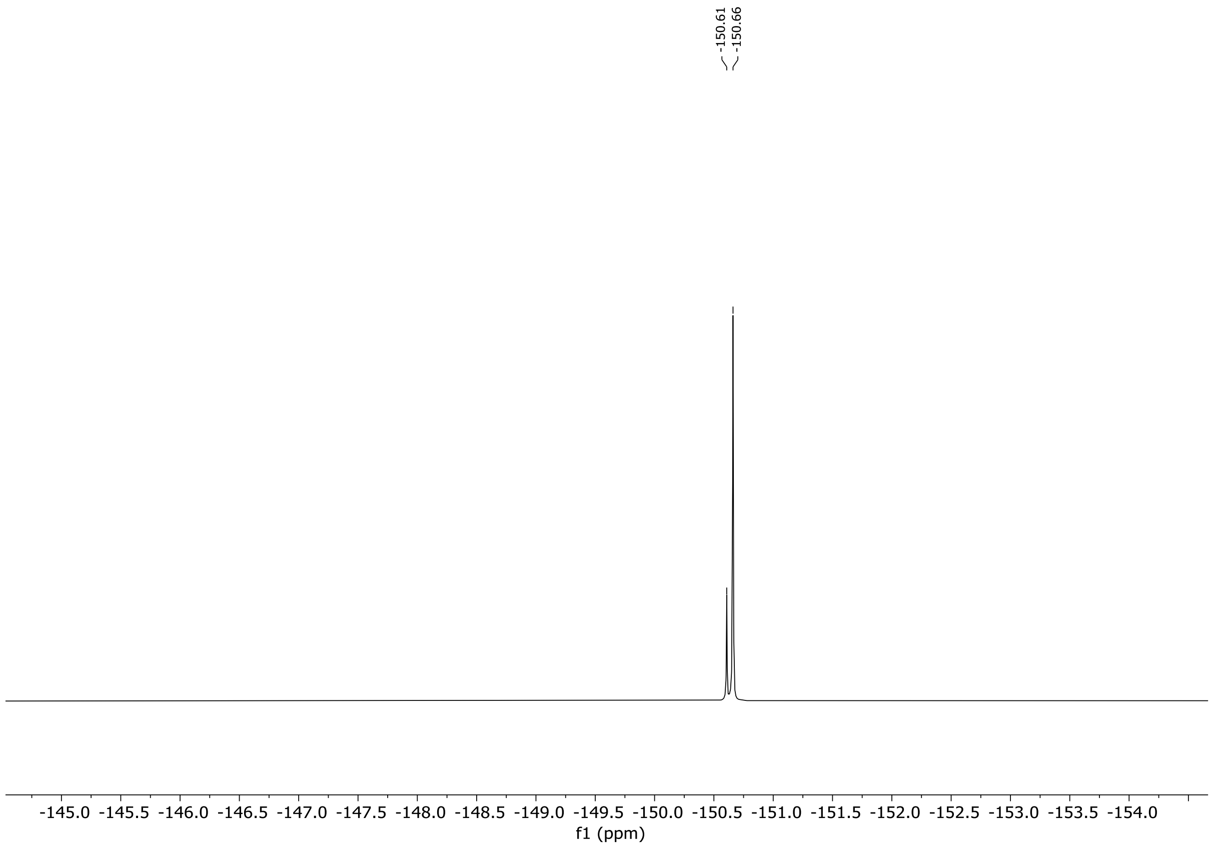

^{1}H NMR of **15**, 300 MHz, DMSO-d_6, 25 °C.

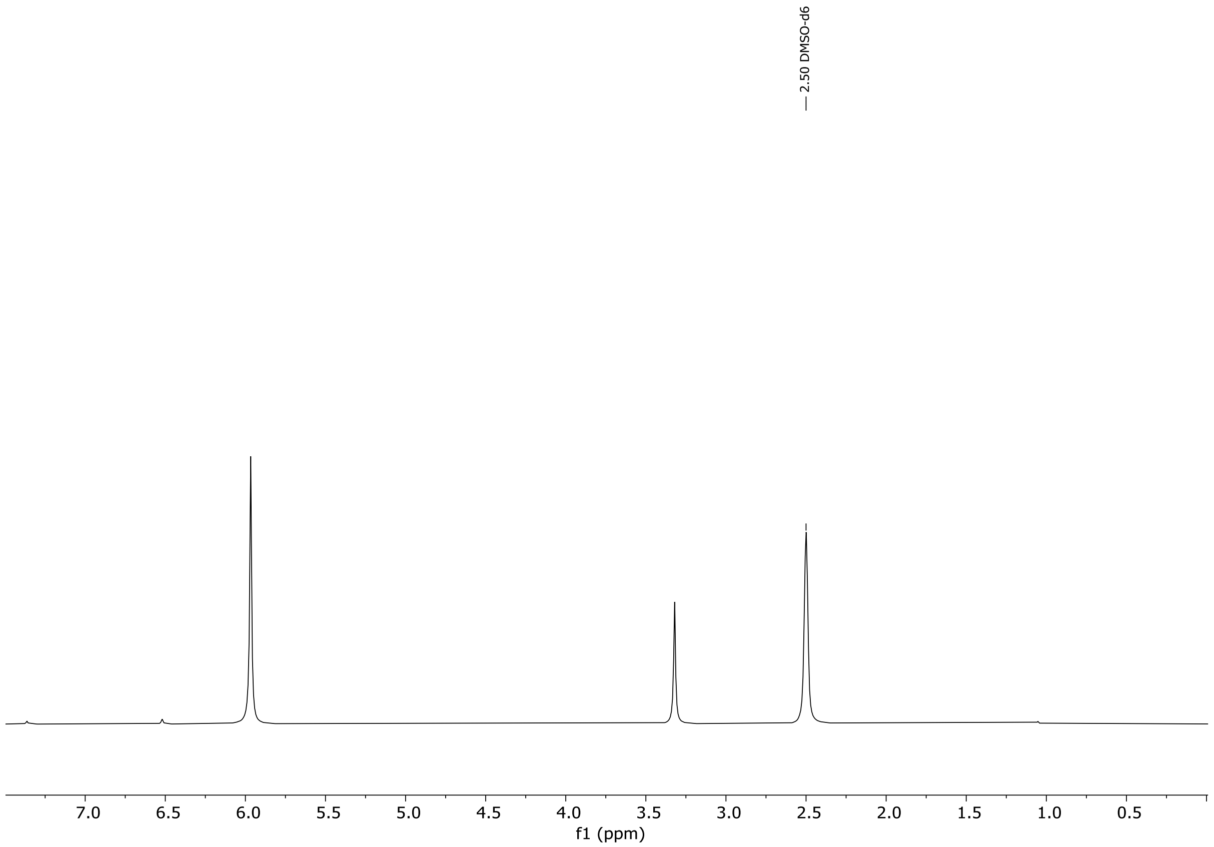

^{13}C NMR of **15**, 75 MHz, DMSO-d_6, 25 °C.

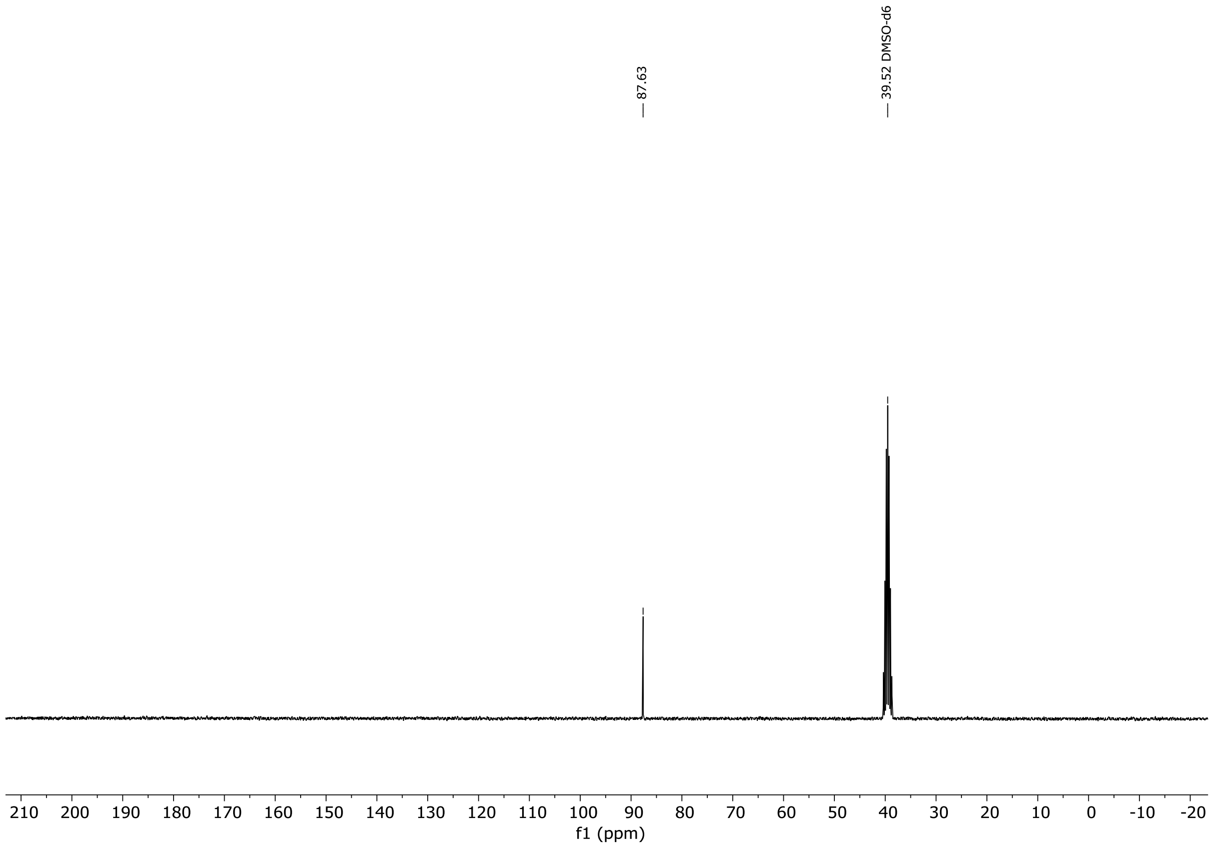

^{1}H NMR of **17**, 300 MHz, CD_3CN, 25 °C.

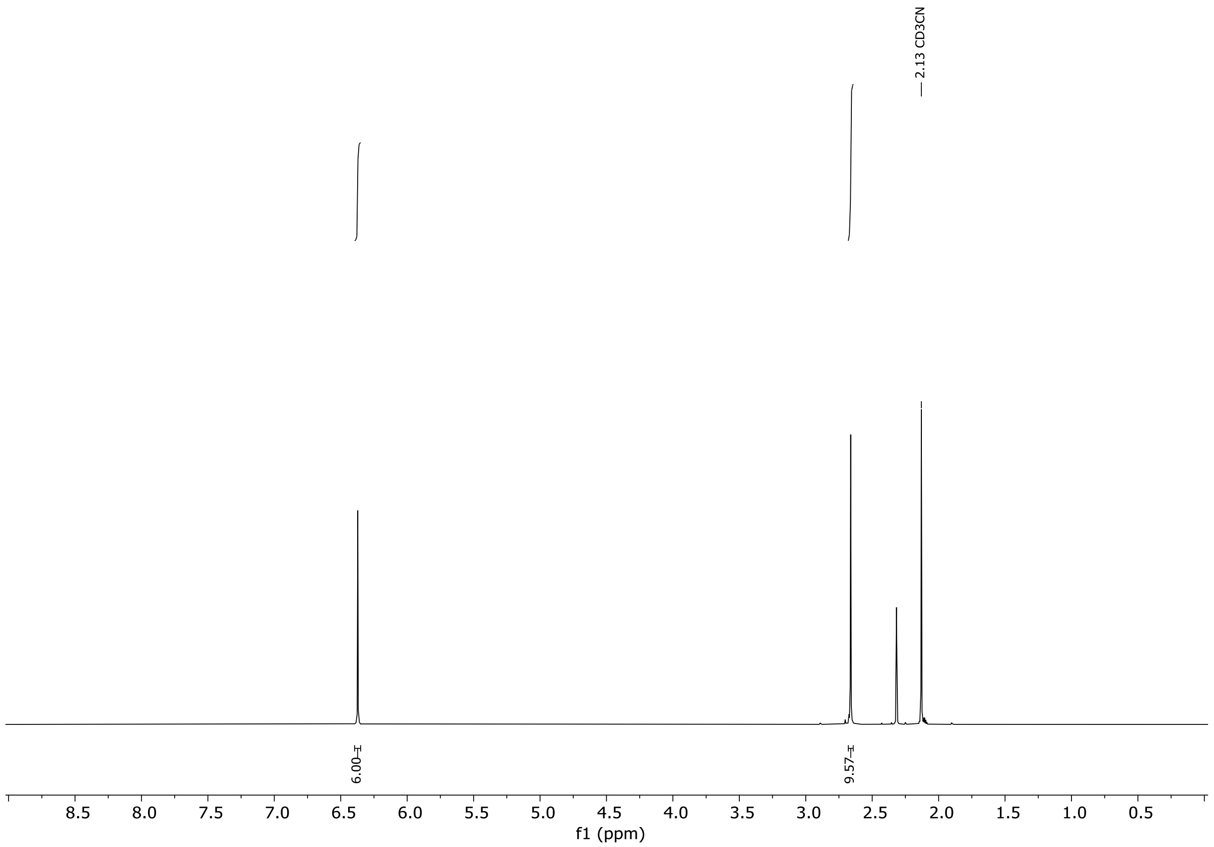

^{19}F NMR of **17**, 282 MHz, CD_3CN, 25 °C.

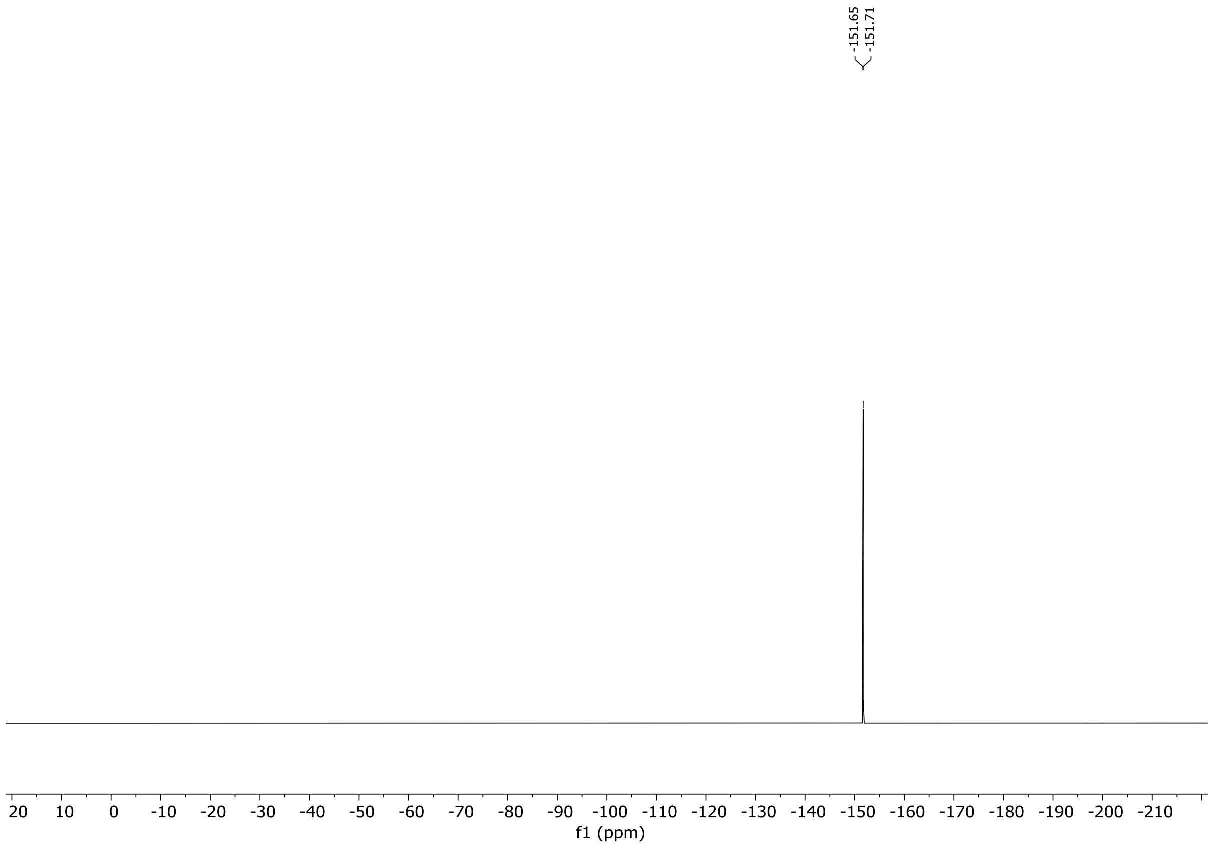

^{13}C NMR of **17**, 75 MHz, CD_3CN, 25 °C.

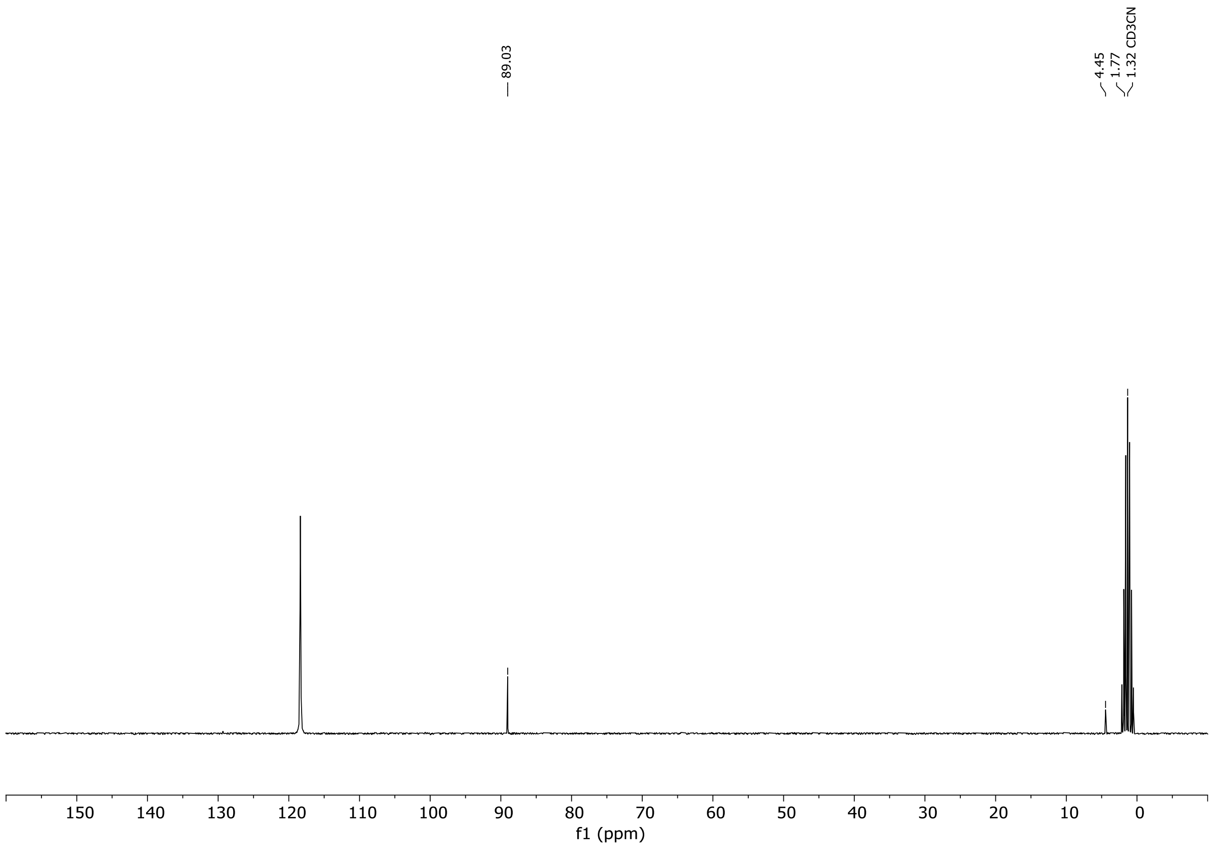

^{1}H NMR of **8**-[D_5], 300 MHz, $CDCl_3$, 25 °C.

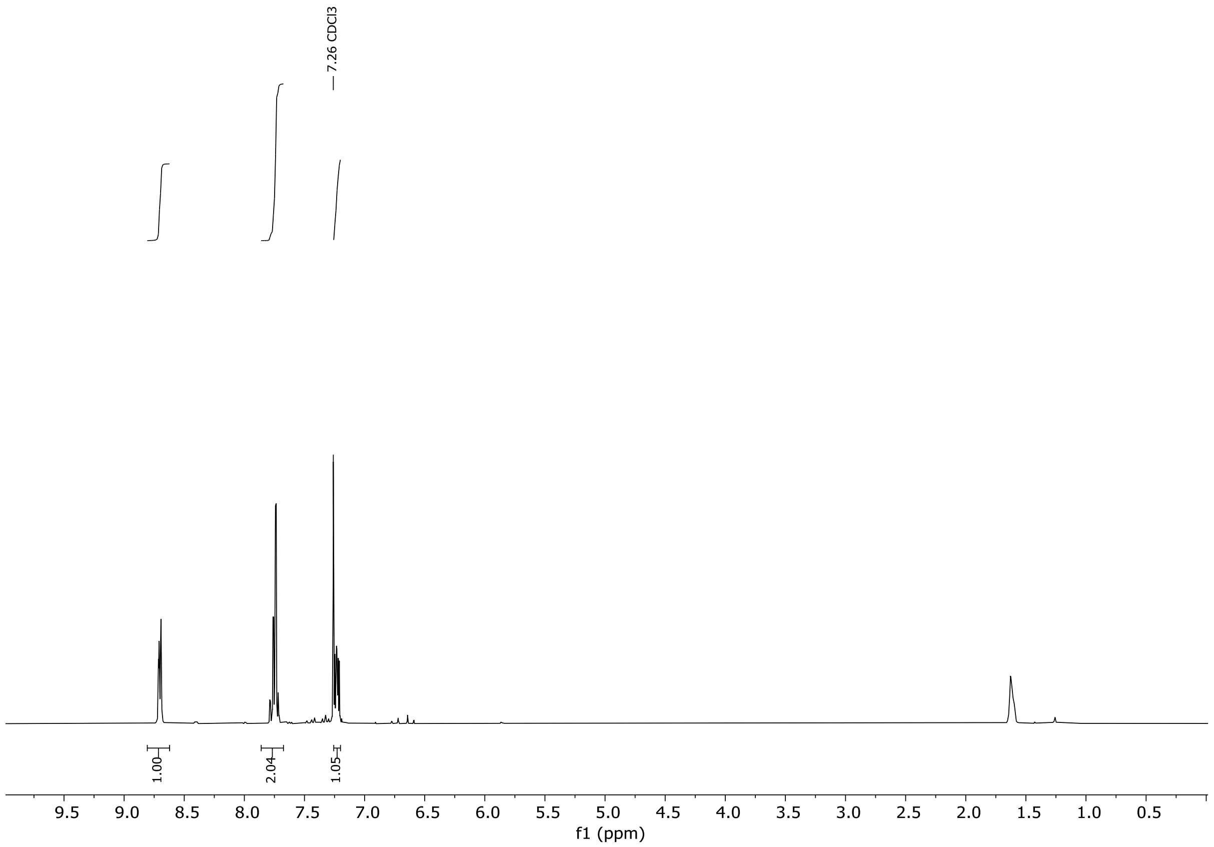

^{13}C NMR of **8**-[D_5], 75 MHz, $CDCl_3$, 25 °C.

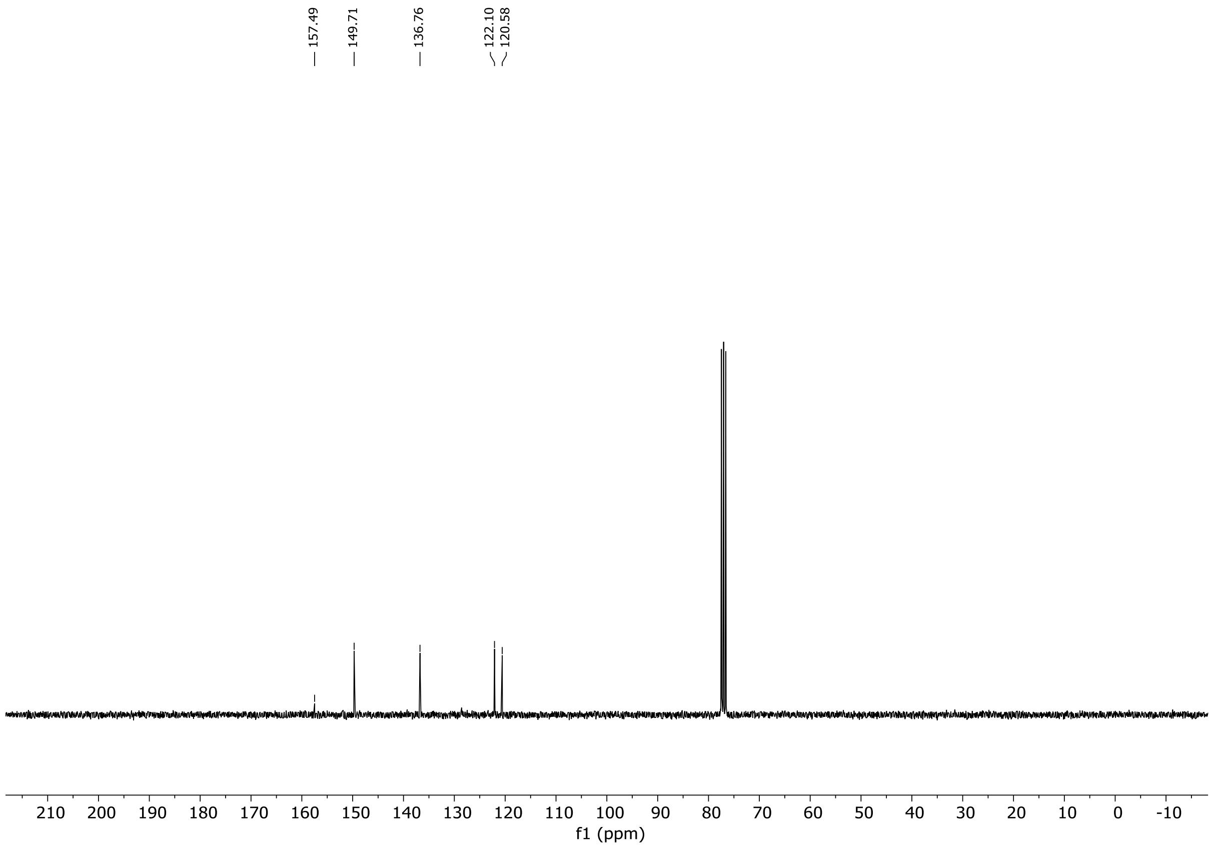

^{1}H NMR of **8**-[D_1], 300 MHz, $CDCl_3$, 25 °C.

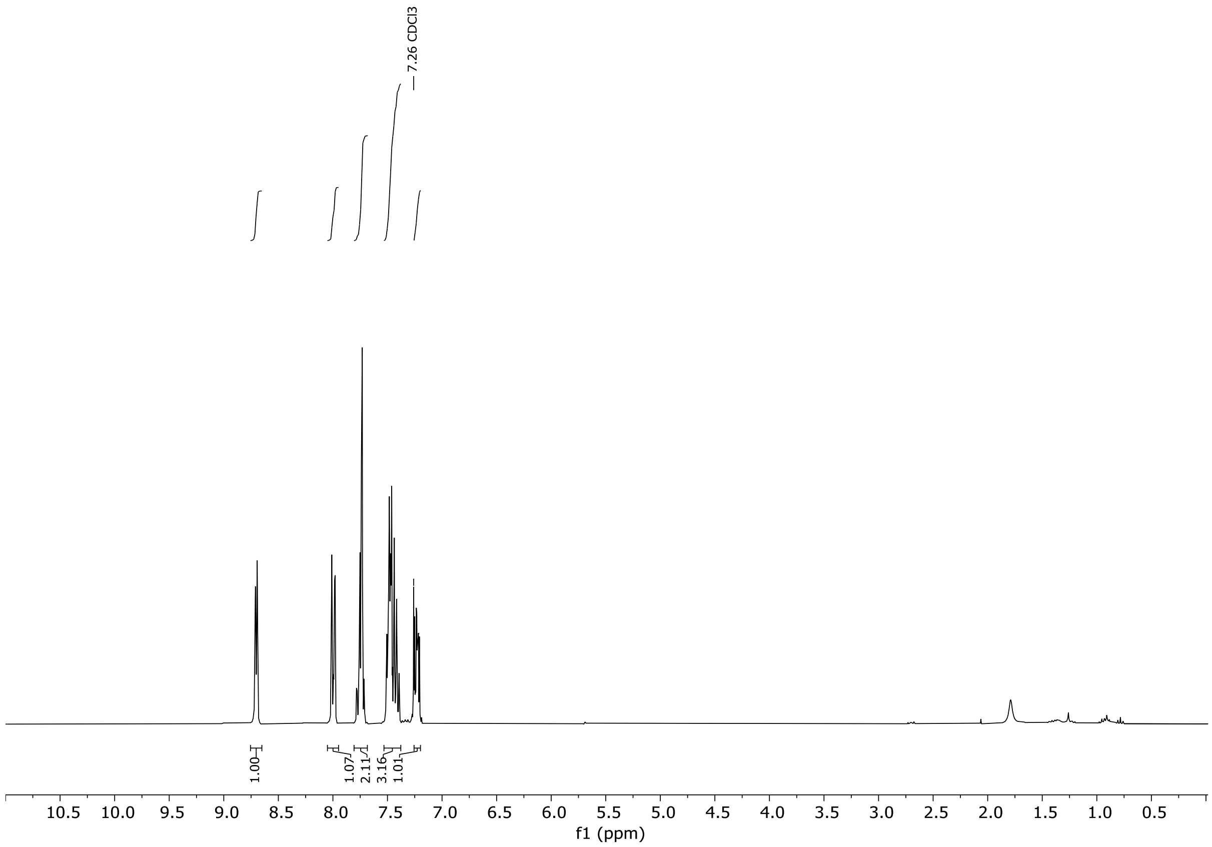

^{1}H NMR of **51f**, 300 MHz, $CDCl_3$, 25 °C.

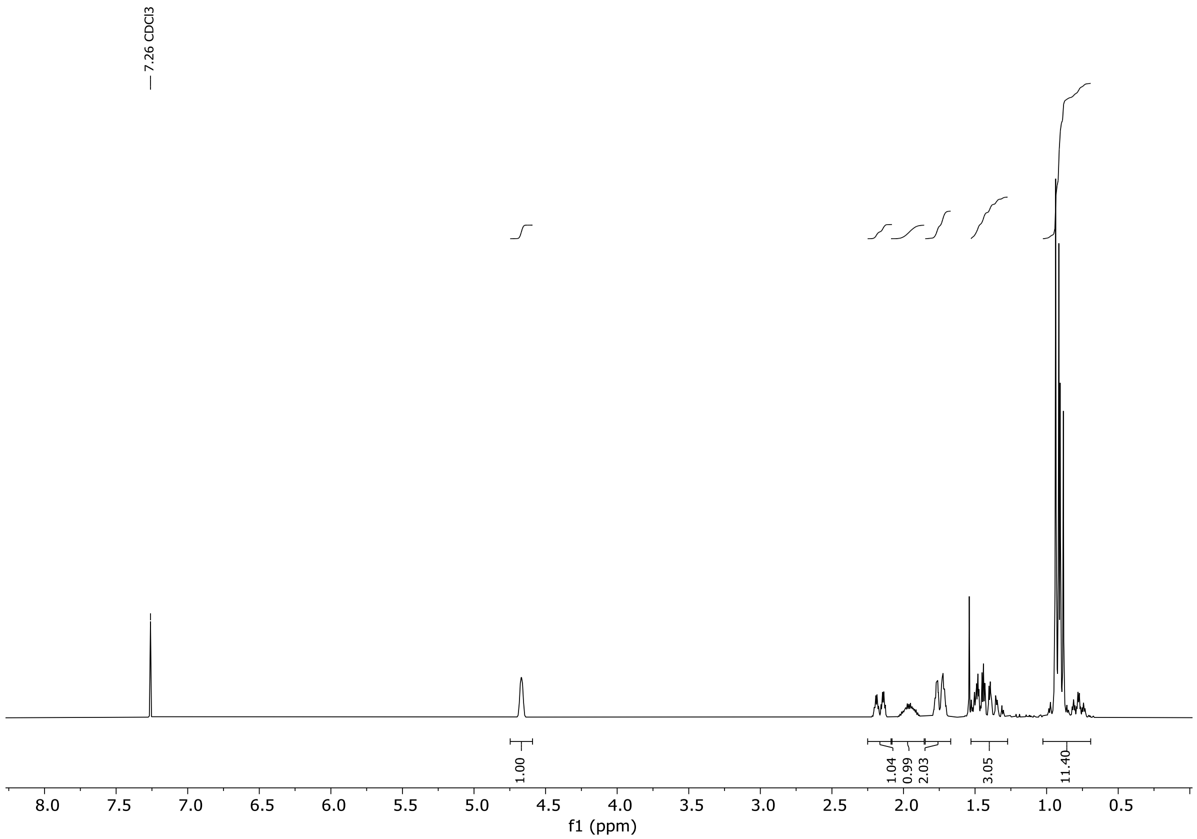

^{13}C NMR of **51f**, 75 MHz, $CDCl_3$, 25 °C.

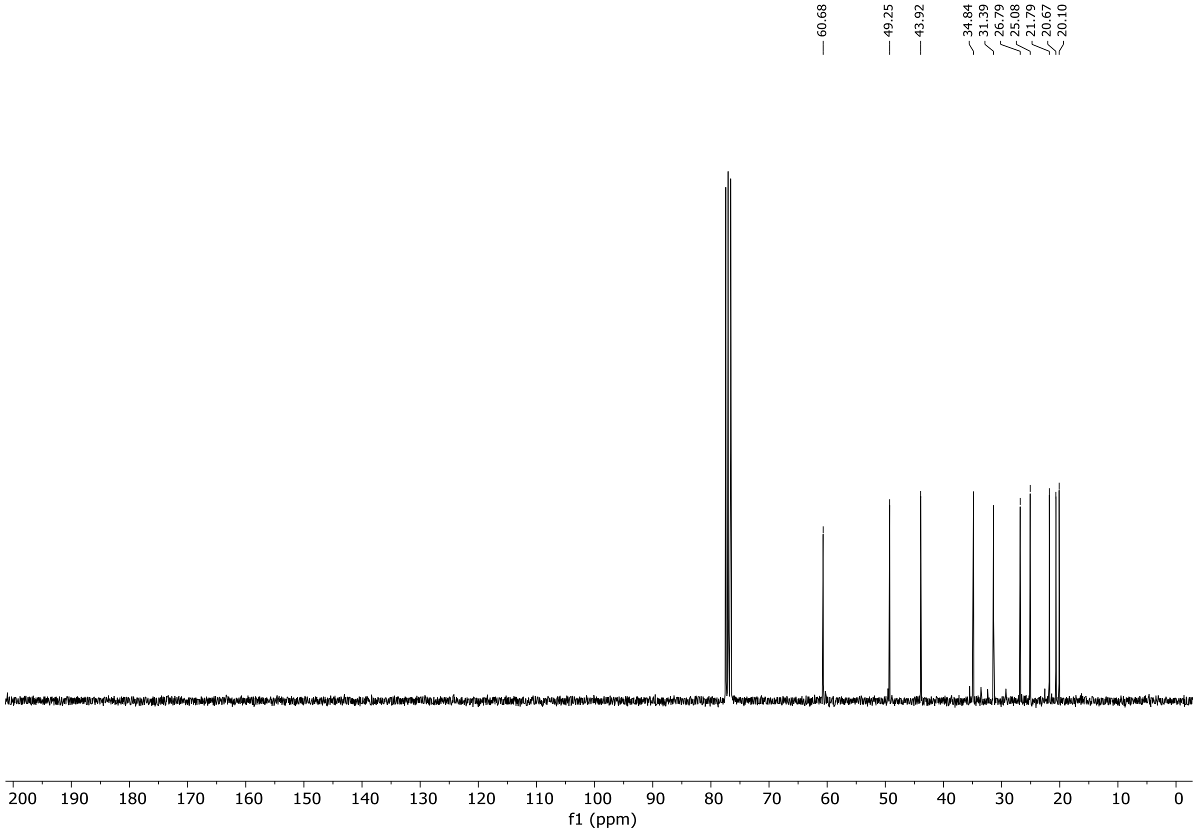

^{1}H NMR of **51g**, 300 MHz, $CDCl_3$, 25 °C.

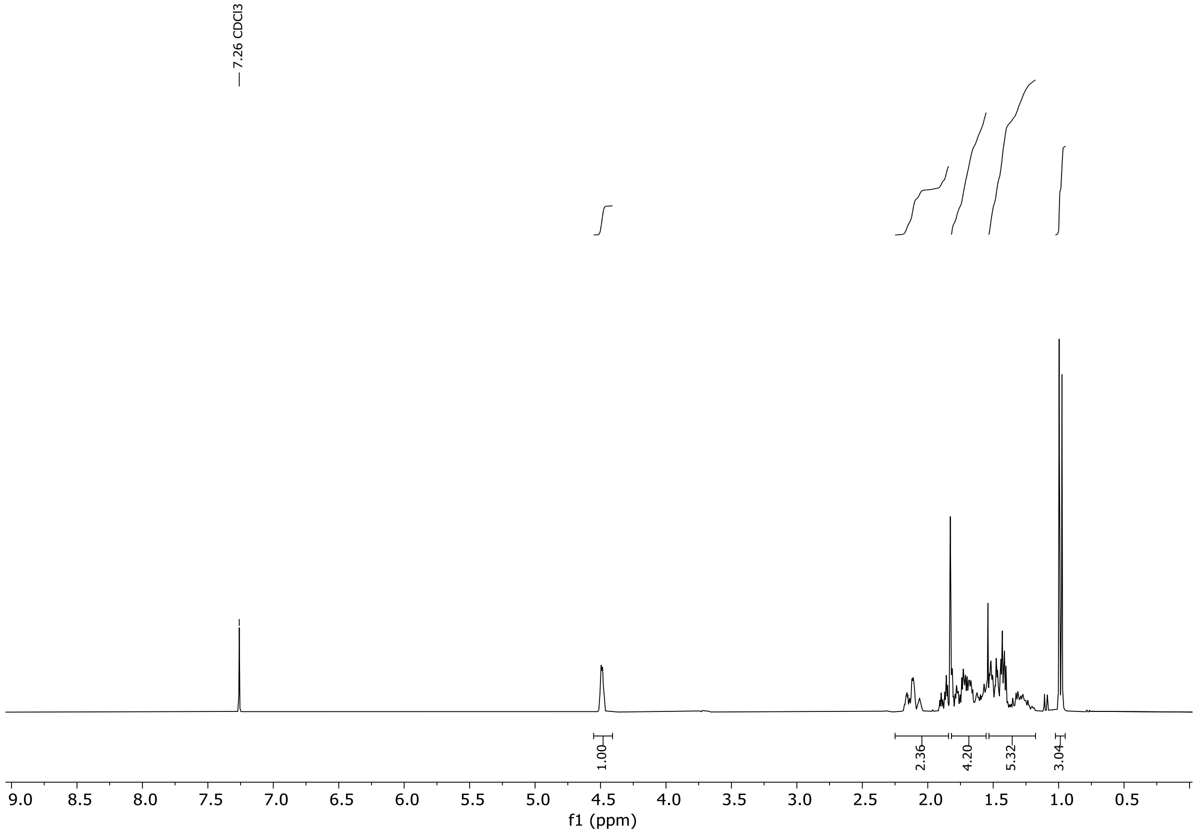

^{1}H NMR of **Diazepam**, 300 MHz, $CDCl_3$, 25 °C.

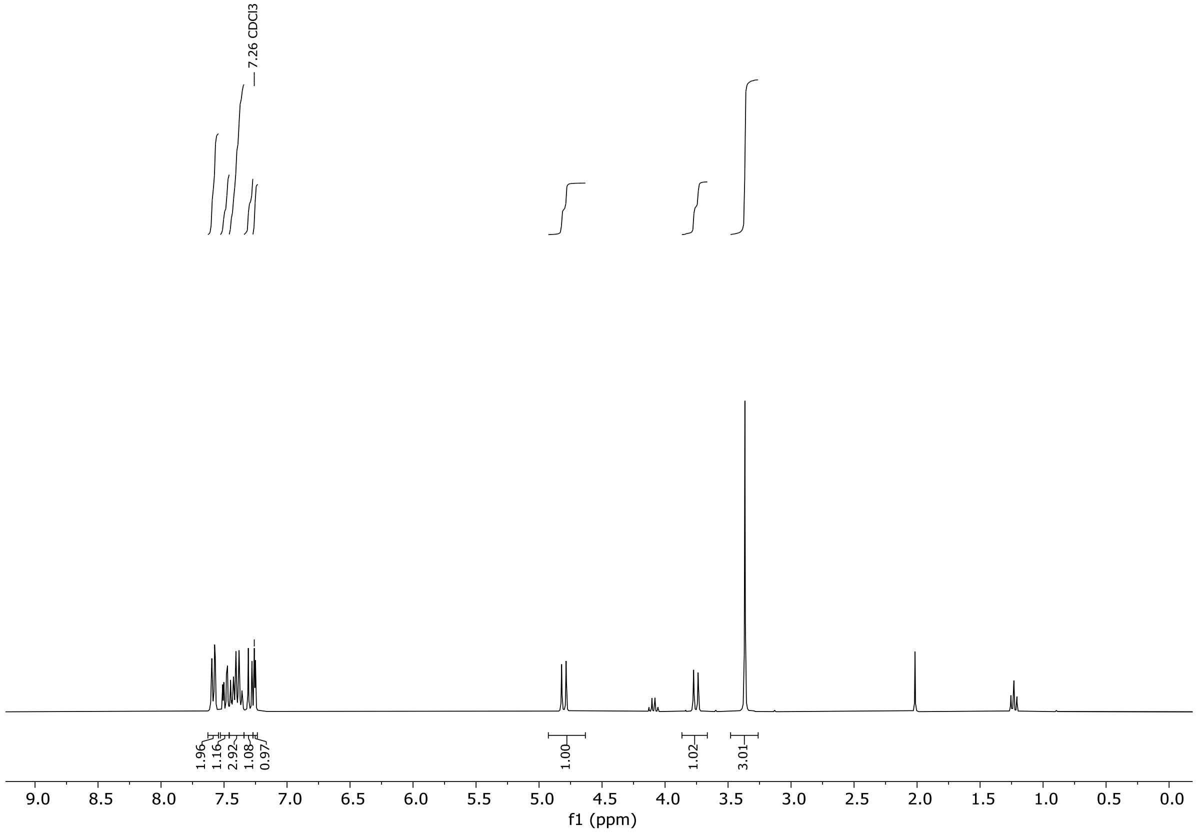

^{13}C NMR of **Diazepam**, 75 MHz, $CDCl_3$, 25 °C.

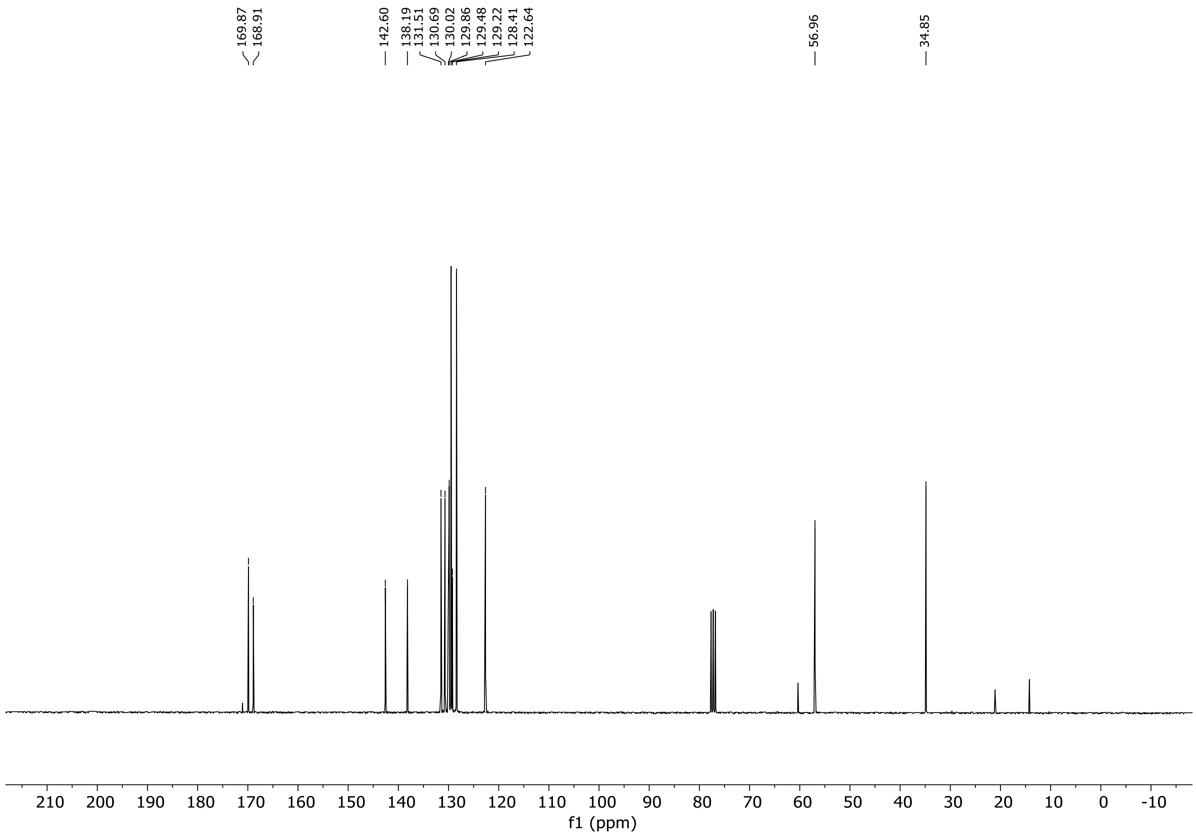

8 References

(1) Wübbolt, S.; Oestreich, M. Catalytic Electrophilic C–H Silylation of Pyridines Enabled by Temporary Dearomatization. *Angew. Chem. Int. Ed.* **2015**, *54*, 15876-15879.

(2) Ackermann, L.; Kozhushkov, S. I.; Yufit, D. S. Ruthenium-Catalyzed Hydroarylation of Methylenecyclopropanes through C–H Bond Cleavage: Scope and Mechanism. *Chem. Eur. J.* **2012**, *18*, 12068-12077.

(3) Yuan, Y.; Liang, Y.; Shi, S.; Liang, Y.-F.; Jiao, N. Efficient Pd-Catalyzed C–H Oxidative Bromination of Arenes with Dimethyl Sulfoxide and Hydrobromic Acid. *Chin. J. Chem.* **2020**, *38*, 1245-1251.

(4) Xu, C.; Shen, Q. Palladium-Catalyzed Trifluoromethylthiolation of Aryl C–H Bonds. *Org. Lett.* **2014**, *16*, 2046-2049.

(5) Zhang, W.; Lin, S. Electroreductive Carbofunctionalization of Alkenes with Alkyl Bromides via a Radical-Polar Crossover Mechanism. *J. Am. Chem. Soc.* **2020**, *142*, 20661-20670.

(6) Ahlburg, A.; Lindhardt, A. T.; Taaning, R. H.; Modvig, A. E.; Skrydstrup, T. An Air-Tolerant Approach to the Carbonylative Suzuki–Miyaura Coupling: Applications in Isotope Labeling. *J. Org. Chem.* **2013**, *78*, 10310-10318.

(7) McArthur, G.; Docherty, J. H.; Hareram, M. D.; Simonetti, M.; Vitorica-Yrezabal, I. J.; Douglas, J. J.; Larrosa, I. An air- and moisture-stable ruthenium precatalyst for diverse reactivity. *Nat. Chem.* **2024**, *16*, 1141-1150.

(8) Zelonka, R. A.; Baird, M. C. Reactions of benzene complexes of ruthernium(II). *J. Organomet. Chem.* **1972**, *35*, C43-C46.

(9) Ackermann, L.; Novák, P.; Vicente, R.; Hofmann, N. Ruthenium-Catalyzed Regioselective Direct Alkylation of Arenes with Unactivated Alkyl Halides through C–H Bond Cleavage. *Angew. Chem. Int. Ed.* **2009**, *48*, 6045-6048.

(10) Ackermann, L.; Hofmann, N.; Vicente, R. Carboxylate-Assisted Ruthenium-Catalyzed Direct Alkylations of Ketimines. *Org. Lett.* **2011**, *13*, 1875-1877.
(11) Korvorapun, K.; Moselage, M.; Struwe, J.; Rogge, T.; Messinis, A. M.; Ackermann, L. Regiodivergent C−H and Decarboxylative C−C Alkylation by Ruthenium Catalysis: *ortho* versus *meta* Position-Selectivity. *Angew. Chem. Int. Ed.* **2020**, *59*, 18795-18803.
(12) (a) Shen, Q.; Peloquin, J. M. The molecular structures and conformations of chlorocyclohexane and bromocyclohexane as determined by gas-phase electron diffraction. *Acta Chem. Scand* **1988**, *42*, 367. (b) Castro, C. R.; Dutler, R.; Rauk, A.; Wieser, H. The structure of bicyclo[2.2.1]heptanes: Norbornane, nornornene, norbornadiene, 7-oxanorbornane, and 7-thianorbornane. *Journal of Molecular Structure: THEOCHEM* **1987**, *152*, 241-253. (c) Kahn, R.; Fourme, R.; André, D.; Renaud, M. Crystal structure of cyclohexane I and II. *Acta Crystallographica Section B* **1973**, *29*, 131-138. (d) Parkin, A.; Oswald, I. D. H.; Parsons, S. Structures of piperazine, piperidine and morpholine. *Acta Crystallographica Section B* **2004**, *60*, 219-227. (e) Breed, H. E.; Gundersen, G.; Seip, R.; Strand, K. A.; Hoyer, E.; Spiridonov, V. P.; Strand, T. G. The Molecular Structure of Gaseous Tetrahydropyran. *Acta Chem. Scand.* **1979**, 225-233. (f) Saebø, S.; Cordell, F. R.; Boggs, J. E. Structures and conformations of cyclopentane, cyclopentene, and cyclopentadiene. *Journal of Molecular Structure: THEOCHEM* **1983**, *104*, 221-232.
(13) Wheatley, M.; Findlay, M. T.; López-Rodríguez, R.; Cannas, D. M.; Simonetti, M.; Larrosa, I. Ru-catalyzed room-temperature alkylation and late-stage alkylation of arenes with primary alkyl bromides. *Chem Catal.* **2021**, *1*, 691-703.
(14) Chen, X.; Goodhue, C. E.; Yu, J.-Q. Palladium-Catalyzed Alkylation of sp2 and sp3 C−H Bonds with Methylboroxine and Alkylboronic Acids: Two Distinct C−H Activation Pathways. *J. Am. Chem. Soc.* **2006**, *128*, 12634-12635.
(15) Hofmann, N.; Ackermann, L. meta-Selective C–H Bond Alkylation with Secondary Alkyl Halides. *J. Am. Chem. Soc.* **2013**, *135*, 5877-5884.
(16) Wang, X.; Ji, X.; Shao, C.; Zhang, Y.; Zhang, Y. Palladium-catalyzed C–H alkylation of 2-phenylpyridines with alkyl iodides. *Org. Biomol. Chem.* **2017**, *15*, 5616-5624.
(17) (a) Tang, J.; Zhao, C.; Li, S.; Zhang, J.; Zheng, X.; Yuan, M.; Fu, H.; Li, R.; Chen, H. Tandem Ring-Contraction/Regioselective C–H Iodination Reaction of Pyridinium Salts. *J. Org. Chem.* **2023**, *88*, 2809-2821. (b) Tang, J.; Li, S.; Zhang, J.; Yan, M.-x.; Shi, Y.-l.; Zheng, X.-l.; Yuan, M.-l.; Fu, H.-y.; Li, R.-x.; Chen, H. Copper-Mediated and Palladium-Catalyzed Cross-Coupling of Indoles and N-Methylpyridinium Salts: A Practical Way to Prepare 3-(Pyridin-2-yl)indoles. *Org. Lett.* **2023**, *25*, 5203-5208.
(18) Li, G.; Ma, X.; Jia, C.; Han, Q.; Wang, Y.; Wang, J.; Yu, L.; Yang, S. Ruthenium-catalyzed meta/ortho-selective C–H alkylation of azoarenes using alkyl bromides. *Chem. Commun.* **2017**, *53*, 1261-1264.
(19) Sagadevan, A.; Greaney, M. F. meta-Selective C−H Activation of Arenes at Room Temperature Using Visible Light: Dual-Function Ruthenium Catalysis. *Angew. Chem. Int. Ed.* **2019**, *58*, 9826-9830.
(20) Yi, W.; Chen, W.; Xu, H.; Chen, K.; Zhong, X.; Zhou, Z. Os(II)-catalyzed γ-C(sp^3)–H amidation and *meta*-C(sp^2)–H alkylation by fine-tuning the characteristics of in-situ-generated C–Os σ bonds. *Cell Reports Physical Science* **2023**, *4*.
(21) Gandeepan, P.; Koeller, J.; Korvorapun, K.; Mohr, J.; Ackermann, L. Visible-Light-Enabled Ruthenium-Catalyzed meta-C−H Alkylation at Room Temperature. *Angew. Chem. Int. Ed.* **2019**, *58*, 9820-9825.
(22) Arockiam, P.; Poirier, V.; Fischmeister, C.; Bruneau, C.; Dixneuf, P. H. Diethyl carbonate as a solvent for ruthenium catalysed C–H bond functionalisation. *Green Chem.* **2009**, *11*, 1871-1875.
(23) Li, W.; Arockiam, P. B.; Fischmeister, C.; Bruneau, C.; Dixneuf, P. H. C–H bond functionalisation with [RuH(codyl)2]BF4 catalyst precursor. *Green Chem.* **2011**, *13*, 2315-2319.
